# Detection and Characterization of Rapidly Equilibrating Glycosylation Reaction Intermediates Using Exchange NMR

**DOI:** 10.1021/jacs.3c08709

**Published:** 2023-11-27

**Authors:** Frank F. J. de Kleijne, Floor ter Braak, Dimitrios Piperoudis, Peter H. Moons, Sam J. Moons, Hidde Elferink, Paul B. White, Thomas J. Boltje

**Affiliations:** Institute for Molecules and Materials (IMM), Synthetic Organic Chemistry, Radboud University, 6525 AJ Nijmegen, The Netherlands

## Abstract

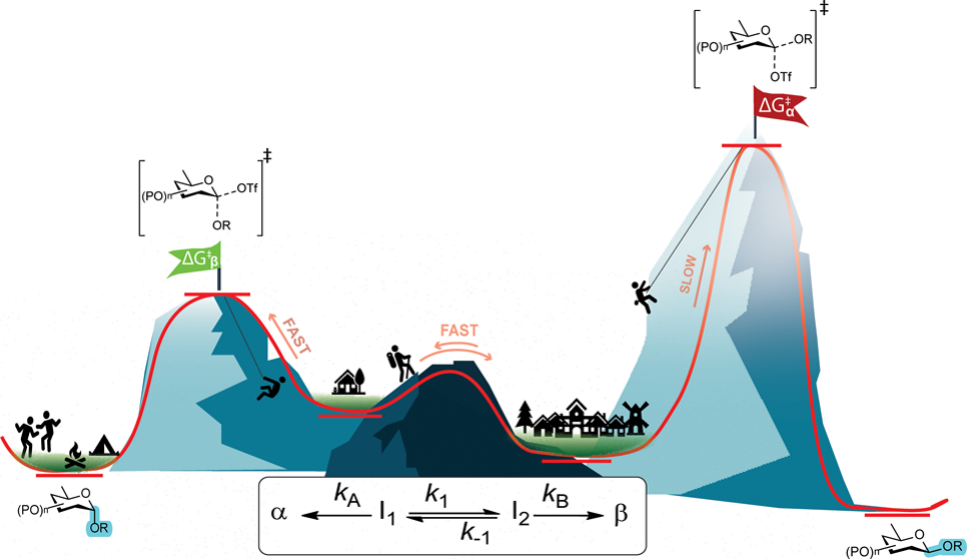

The stereoselective introduction of glycosidic bonds (glycosylation) is one of the main challenges in the chemical synthesis of carbohydrates. Glycosylation reaction mechanisms are difficult to control because, in many cases, the exact reactive species driving product formation cannot be detected and the product outcome cannot be explained by the primary reaction intermediate observed. In these cases, reactions are expected to take place via other low-abundance reaction intermediates that are in rapid equilibrium with the primary reaction intermediate via a Curtin–Hammett scenario. Despite this principle being well-known in organic synthesis, mechanistic studies investigating this model in glycosylation reactions are complicated by the challenge of detecting the extremely short-lived reactive species responsible for product formation. Herein, we report the utilization of the chemical equilibrium between low-abundance reaction intermediates and the stable, readily observed α-glycosyl triflate intermediate in order to infer the structure of the former species by employing exchange NMR. Using this technique, we enabled the detection of reaction intermediates such as β-glycosyl triflates and glycosyl dioxanium ions. This demonstrates the power of exchange NMR to unravel reaction mechanisms as we aim to build a catalog of kinetic parameters, allowing for the understanding and eventual prediction of glycosylation reactions.

## Introduction

The stereoselective introduction of glycosidic bonds (glycosylation) is one of the main challenges in the chemical synthesis of carbohydrates. In a chemical glycosylation reaction, an electrophile (the glycosyl donor) is activated by a chemical promoter and reacts with a nucleophile (the glycosyl acceptor). The nucleophile can add to the α- or β-face of reactive intermediates, thereby leading to the formation of α- or β-diastereoisomers, respectively. Controlling the diastereoselectivity of glycosylation reactions can be achieved by the application of two main strategies.

**Figure 1:**
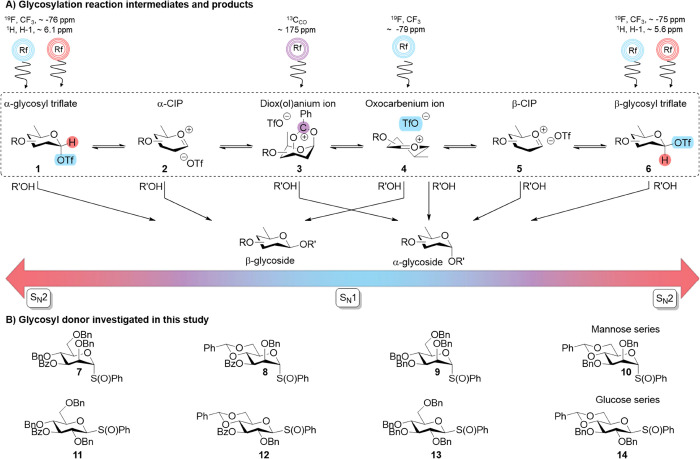
(A) Glycosylation reaction intermediates and their characteristic resonances monitored by exchange NMR. (B) Glycosyl donors used in this study.

First, a stereo-directing group present on the donor molecule can be employed to stabilize the glycosyl cation formed upon activation. An example of this principle is neighboring group participation (NGP) of an acyl group at the C-2 position affording a bicyclic dioxolanium ion intermediate **3** that reacts in a stereospecific manner with a glycosyl acceptor to afford a 1,2-*trans* product (Figure 1A).^1−4^ Extension of this principle to acyl functionalities positioned on the C-3, C-4, or C-6 hydroxyl groups via NGP has also been suggested to direct the stereoselectivity of glycosylation reactions. However, whether selectivity can be attributed to NGP of the acyl group or other stereoelectronic effects is a subject of much debate.^4−19^ The second main strategy utilizes glycosyl donors that contain protecting groups that are less capable of neighboring group participation, e.g., benzyl ethers. In this case, the glycosyl cation is trapped by the promotor system counterion or a solvent additive to afford *quasi-*stable intermediates that can be displaced in an S_N_2-like reaction pathway to afford a glycosylation product.^20−22^ Most modern promotor systems give rise to the formation of glycosyl triflates and since these covalent adducts can exist in the α- (**1**) or β-form (**6**), reactions proceeding via these intermediates can in principle form the β- or α-product via an S_N_2-like reaction pathway, respectively.^22,23^ The nucleophilic displacement of α-glycosyl triflates **1** is likely to take place via an intermediate α-contact ion pair (CIP) **2** which maintains its stereochemical memory to form the β-product.^24^ Full dissociation of the triflate would lead to the solvent-separated ion pair (SSIP) **4**, which can afford glycosylation products via an S_N_1-like pathway.^25−27^ The conformation of monosaccharide-derived SSIPs is dictated by the relative stereochemistry of its substituents^10^ and is a crucial determinant of their stereoselectivity in glycosylation reactions.^25^ Finally, β-glycosyl triflate CIP **5** and its corresponding covalent adduct **6** can form during glycosylation reactions from the SSIP or S_N_2-like displacement of the α-glycosyl triflate **1** by another triflate anion.^20^ The equatorial β-glycosyl triflates (on d-sugars) are not stabilized by the anomeric effect and hence are less stable and more reactive. Since glycosylation reactions take place in a mechanistic continuum between these S_N_1- and S_N_2-like reaction pathways, they are difficult to predict and control and very sensitive to parameters such as solvent,^28,29^ type of monosaccharide,^17,30^ strength of the nucleophile,^31^ reaction solvent,^32^ and temperature.^33,34^

To address this challenge, the characterization of reaction intermediates that drive product formation in glycosylation reactions is required. Glycosylation reaction intermediates can be characterized by employing nuclear magnetic resonance (NMR) spectroscopy and infrared-ion spectroscopy (IRIS), for example.^26,35,36^ These techniques have allowed for the characterization of intermediates such as glycosyl triflates,^22,23,35^ dioxolanium ions,^18,37,38^ dioxanium ions,^17^ and even oxocarbenium ions.^25,26,37,39^ However, IRIS is performed in the gas phase, and the structure of reaction intermediates under these conditions may not always be relevant to those formed in solution. NMR can be used to detect reaction intermediates in solution under relevant reaction conditions and is a powerful tool for studying reaction mechanisms.^40^ However, a challenge in studying glycosylation reactions is that observing a reaction intermediate does not automatically mean it is a reactive intermediate and, hence, relevant to product formation. In many cases, the exact reactive species driving glycoside product formation remains unknown as the product outcome cannot be explained by the primary reaction intermediate observed. In these cases, glycosylation reactions are expected to take place via other low-abundance reaction intermediates that are in rapid equilibrium with the primary reaction intermediate via a Curtin–Hammett scenario (Figure 1).^20,41^ Therefore, the stereochemical outcome of the reaction does not necessarily depend on the population of the intermediate leading to a given product but rather the overall barrier height when the barrier to intermediate interconversion is lower than the irreversible product-forming step.^20^ Despite this principle being well-known in organic synthesis, mechanistic studies investigating this model in glycosylation reactions are complicated by the challenge of detecting the extremely short-lived reactive species responsible for product formation and measuring their exchange kinetics. The low abundance and short lifetime of these intermediates, such as **2**–**6**, complicate their characterization as they readily equilibrate back to form the more stable, readily observable but less reactive α-glycosyl triflate intermediate **1**. Elegant studies by Taylor and co-workers, and Asensio and co-workers, have employed exchange NMR spectroscopy (EXSY) to characterize the dynamic equilibrium of α- and β-glycosyl sulfonate intermediates.^23,42^ The use of EXSY is unfortunately limited to readily observed species and hence applies only to rather stable β-glycosyl sulfonates. We have recently overcome this challenge by utilizing the chemical equilibrium between low-abundance reaction intermediates and the stable, readily observed α-glycosyl triflate intermediate in order to infer the structure of the former species.^17^ Using chemical exchange saturation transfer (CEST) NMR, we demonstrated that high-energy or “invisible” mannosyl dioxanium ions, which are formed by intramolecular stabilization of a C-3 ester, are in chemical exchange with the highly populated α-glycosyl triflate intermediate.^17^

Herein, we report the application of this principle to detect the presence of other virtually undetectable high-energy reaction intermediates relevant to product formation such as β-triflates and dioxanium ions formed by internal stabilization. We characterized the reactive intermediates for a systematic set of eight frequently used glycosyl donors **7–14** (Figure 1B). Not only the presence of reactive intermediates but also their exchange kinetics were measured, thereby providing valuable quantitative data to elucidate the formation mechanism of the reactive intermediates. We report an integrated exchange NMR workflow to measure the reactivity of α-glycosyl triflates by monitoring the dissociation of the triflate ion using ^19^F exchange NMR spectroscopy (EXSY NMR). In addition, we established the mechanism of triflate dissociation with the same technique. A clear difference between mannose and glucose monosaccharides was observed in their triflate dissociation kinetics and mechanism. Mannosides were able to form dioxanium ions **7**_**d**_ and **8**_**d**_ via the participation of a C-3 acyl group, whereas their glucoside counterparts were not and formed β-glycosyl triflates **11**_**βOTf**_ and **12**_**βOTf**_ instead. We were able to indirectly detect the presence of these low-population intermediates via their chemical equilibrium with the observable α-glycosyl triflate using ^13^C CEST, ^1^H CEST, and ^19^F CEST NMR. Finally, we were also able to characterize selected examples of the dioxanium ion and β-glycosyl triflate using more classical NMR techniques to unequivocally establish their structure. These results demonstrate the power of chemical exchange NMR to detect fleeting reaction intermediates to build a catalog of kinetic parameters that allow for the understanding and ultimately prediction of glycosylation reactions. We expect this technique to be applicable to various other types of glycosylation reaction intermediates such as additives and solvents commonly used in glycosylation reactions, both of which tend to be rich in NMR-active nuclei that are sensitive to changes in the chemical environment. Finally, the application of the workflow laid out herein should be applicable to other types of reactions that are under Curtin–Hammett control.

## Results and Discussion

**Figure 2:**
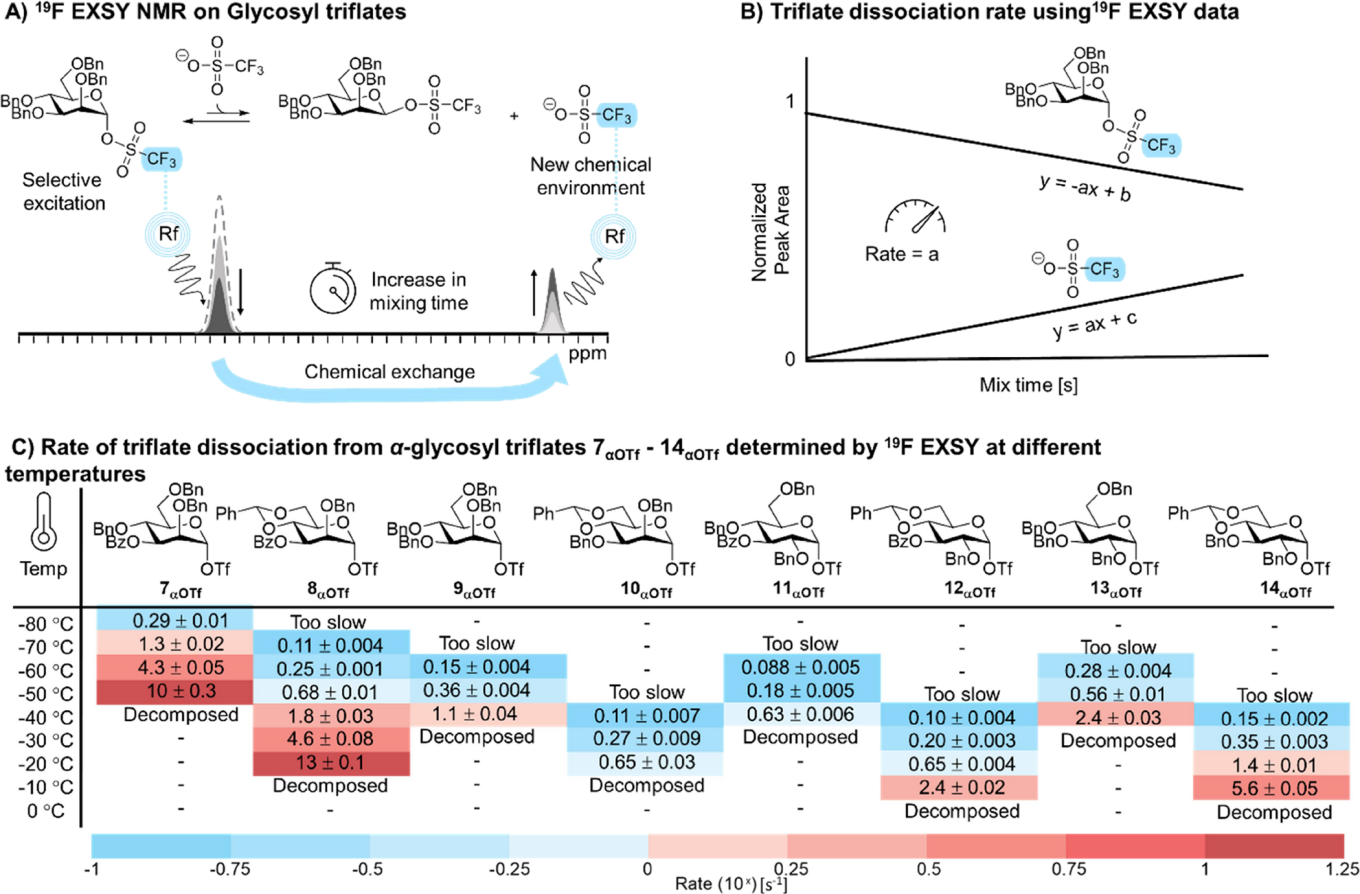
(A) Working principle behind ^19^F EXSY NMR to monitor triflate dissociation. The α-glycosyl triflate is selectively excited and then observed over time to transform into free triflate. (B) Determination of the initial rate of triflate dissociation. (C) Summary of normalized triflate dissociation rates (s^–1^) across the studied glycoside series as a function of temperature.

We started by investigating the stability and reactivity of glycosyl triflates derived from **7** to **14** to assess their likelihood of acting as reactive intermediates in glycosylation reactions. Previous experiments have investigated the decomposition temperature of glycosyl triflates as a measure of their stability/reactivity. While indicative of their thermal stability, such metrics do not necessarily speak to their relevance in product formation during a glycosylation event.^43^ In addition, ^1^H EXSY NMR has been used to monitor the interconversion of α- and β-glycosyl triflates^23^ and mesylates^42^ providing kinetics of their interconversion. However, a major limitation of ^1^H EXSY is that it requires both interconverting species to be visible in 1D NMR. This means that for the vast majority of glycosyl donors, intermediate exchange kinetics cannot be recorded due to the low-populated state of the highly reactive β-triflate intermediates. Another means of measuring α-glycosyl triflate stability and reactivity is by observing the kinetics of triflate dissociation in the absence of an acceptor using ^19^F EXSY NMR. Since both the α-glycosyl triflate and unbound triflate anion, which results from the activation step (*vide infra*), are always observed and exist as strong signals, we reasoned that if we could observe the interconversion between bound and free triflate, then that would indicate the presence of an unobserved intermediate. Hence, we started by investigating the stability and reactivity of eight glycosyl triflates derived from **7**–**14** using ^19^F EXSY NMR, which revealed that all eight species underwent triflate exchange. The preparation of the thioglycoside precursors used to generate glycosyl triflates **7**_**αOTf**_–**14**_**αOTf**_ is described in the Supporting Information (SI pages S12–S31). The glycosyl triflates were generated by activating the corresponding glycosyl sulfoxide donor with triflic anhydride (Tf_2_O) in the presence of the non-nucleophilic base 2,4,6-tri*tert*-butyl-pyrimidine (TTBP) in CD_2_Cl_2_ at −80 °C inside an NMR tube (please see pages S7–S10).^44^ Clean formation of glycosyl triflates (**7**_**αOTf**_–**14**_**αOTf**_) was observed in all cases (Figures S4–S11). Dissociation of the anomeric triflate to unbound triflate could be monitored using selective ^19^F EXSY NMR (SI pages S2–S4, S7). Magnetization of the selectively excited CF_3_ resonance of the α-glycosyl triflate is transferred to the unbound triflate ion upon triflate dissociation during the ZZ-exchange mix time (Figure 2A). By plotting the mix time vs the extent of magnetization transfer, the rate of triflate dissociation was measured, which we propose is an indicator of α-glycosyl triflate stability (Figure 2B). In order to compare measurements across different sugars and samples while also taking into account multiple mechanisms, the reported rates in Figure 2 are normalized by dividing the measured rate by the initial α-triflate concentration (see SI pages S2–S4). By subsequently repeating this process at different temperatures, the normalized rate of triflate dissociation as a function of temperature was established (Figures 2C and S50–S86). A few important considerations are needed to ensure that reliable kinetic data emerge from the ^19^F EXSY experiments. First, the exchanging system needs to be in the slow-exchange regime which is defined by the rate of exchange being much lower than the frequency difference between the interconverting species (*k*_1_ + *k*_–1_ ≪ Δω_A-B_). Second, to reduce the complexity of modeling the kinetics, we chose to measure initial rates for the exchange processes, which puts a rough limit on the maximum normalized rate of ∼100 s^–1^ (10% conversion at 1 ms mix time). Furthermore, the slowest measurable rate is related to the *T*_1_ of the observed nucleus. Hence, the *T*_1_ of the nuclei, and more importantly, the difference in *T*_1_ between the resonances of interest, should be taken into consideration when setting the maximum mix time. For ^19^F, the *T*_1_s of the α-triflate **10**_**αOTf**_ and soluble triflate salt were measured at −80 °C where the exchange was frozen and found to be roughly identical (0.31 and 0.42 s, respectively, Figure S49). This allowed us to make the approximation that *T*_1_ losses for each were roughly equivalent and hence could be ignored thereby allowing us to extend our mix range past *T*_1_. Therefore, we were able to measure normalized rates accurately down to ∼0.1 s^–1^. Third, the population of the α-glycosyl triflate and free triflate should not significantly change from the beginning to the end of the experiment. Therefore, once thermal decomposition begins to take hold, these experiments become unreliable and thus limit the upper limit of the temperature window.

Keeping in mind these considerations, the rates of dissociation of triflate from the parent α-glycosyl triflates **7**_**αOTf**_–**14**_**αOTf**_ at various temperatures were obtained (Figure 2C). Interestingly, clear differences in triflate dissociation were observed related to the relative stereochemistry of the monosaccharide (mannose vs glucose) and the protecting group pattern. Mannosyl triflates **7**_**αOTf**_ and **8**_**αOTf**_ carrying a single benzoate ester at C-3 showed the fastest triflate dissociation. Interestingly, the benzylidene-protected mannosyl analogue **8**_**αOTf**_ carrying a C-3 benzoate ester was the second fastest in the mannose series. Benzylidene-protected monosaccharides are typically classified as disarmed due to the torsional^45,46^ and electronic effects^47^ induced by the fused benzylidene ring system.^43^ Indeed triflate dissociation for **8**_**αOTf**_ was slower than that of the corresponding benzylated analogue **7**_**αOTf**_ but still faster than that of the fully benzylated compound **9**_**αOTf**_. This suggests a role of the C-3 ester in driving triflate release, overpowering the disarming effect induced by the benzylidene. This is confirmed by the fact that the benzylidene-protected mannosyl triflate containing benzyl ethers at C-2 and C-3 was the slowest in the mannose series in triflate dissociation. Interestingly, the opposite trend was observed in the glucose series. The difference in rate for the C-3 benzoyl **11**_**αOTf**_ and **12**_**αOTf**_ vs C-3 benzyl **13**_**αOTf**_ and **14**_**αOTf**_ analogues was much smaller with the former being slower, particularly at higher temperatures. As expected, the benzylidene-protected analogues **12**_**αOTf**_ and **14**_**αOTf**_ showed slower triflate dissociation compared to that of their benzylated counterparts **11**_**αOTf**_ and **13**_**αOTf**_. Overall, these results indicate that the presence of a benzylidene acetal compared to the benzylated analogue slows down triflate dissociation for both the mannose and glucose series. However, the introduction of a C-3 benzoyl group speeds up triflate dissociation in the mannose series and slows down triflate release in the glucose series.

**Figure 3:**
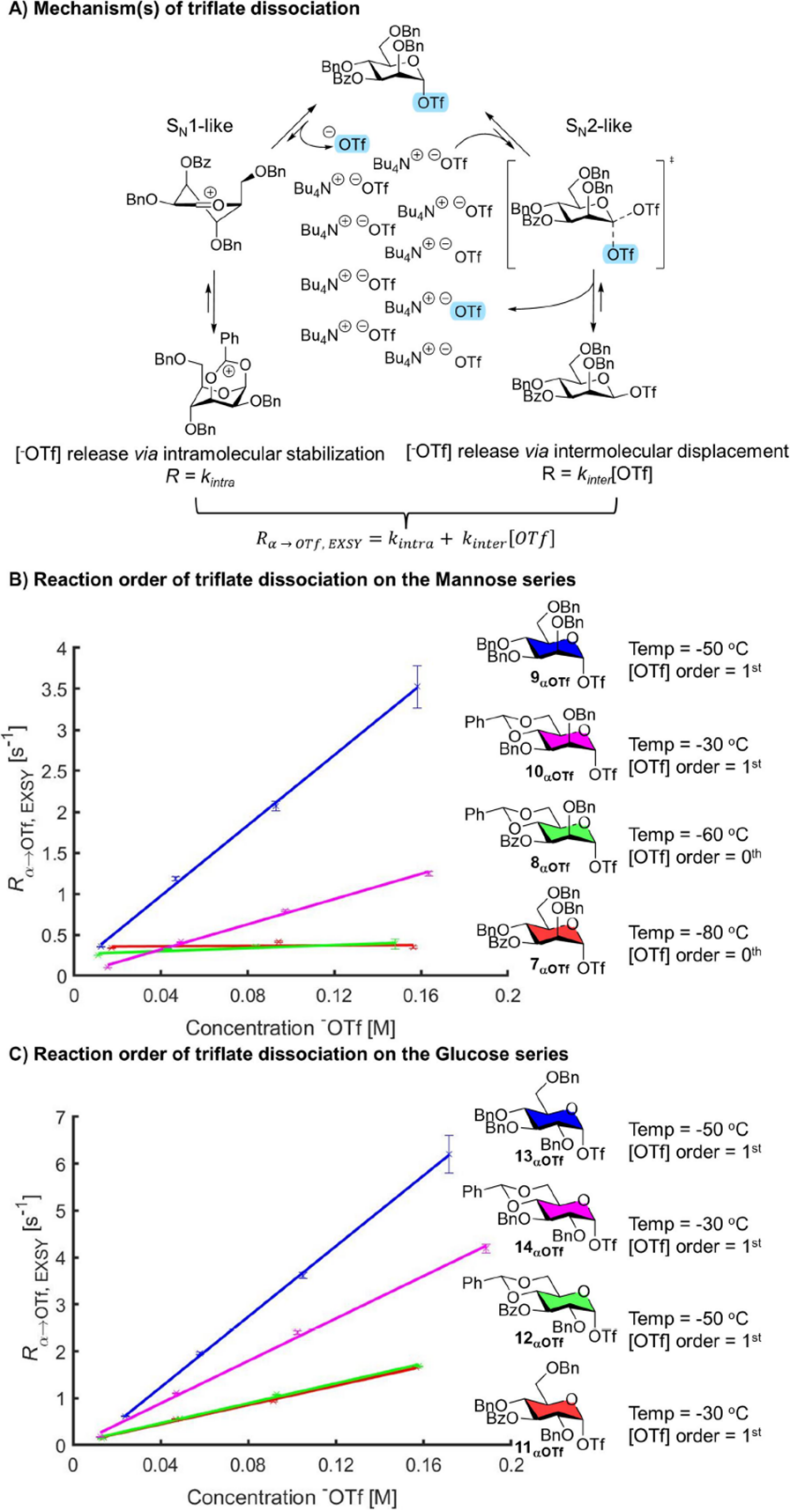
(A) S_N_1 and S_N_2-like triflate dissociation mechanisms. (B) Reaction order determination for triflate dissociation in the mannose series and (C) glucose series.

The striking differences in triflate dissociation rates are likely the result of a different mechanism of triflate dissociation, but these cannot be established from the rates alone. Hence, we set out to investigate the mechanism of triflate dissociation of **7**_**αOTf**_–**14**_**αOTf**_ to explain their dissociation rate differences. We foresee three main equilibria that would lead to the dissociation of the triflate anion (Figure 3A). First, dissociation of the triflate anion could lead to an SSIP. Second, NGP of the C-3 acyl substituent present in molecules **7**_**αOTf**_–**8**_**αOTf**_ could take place to form a dioxanium ion with or without an intermediary SSIP. Third, a triflate anion could displace the α-glycosyl triflate in an S_N_2-like manner to form the corresponding β-triflate. To dissect the mechanism(s) responsible for triflate dissociation, we investigated the rate of this process as a function of the triflate anion concentration. The rate of triflate dissociation from the α-glycosyl triflate via SSIP or dioxanium ion formation should be insensitive to the triflate concentration. The rate of triflate dissociation from the α-glycosyl triflate via S_N_2-like displacement to form the corresponding β-glycosyl triflate should be first order with respect to the triflate concentration. In case both processes operate simultaneously, the sum of the rates would constitute the overall rate of triflate dissociation (Figure 3A). The temperature for the study was chosen so that if a first-order dependence was found, it would not move the kinetics outside the window for EXSY and at the same time would be suitably fast enough to study if no dependence was discovered. Next, using the aforementioned ^19^F EXSY scheme, we measured the triflate dissociation as a function of triflate concentration at a single temperature by the addition of tetrabutylammonium triflate (TBAT).

To this end, the triflate concentration was increased in steps by taking the sample out of the probe and adding 1 M TBAT solution to dry CD_2_Cl_2_ at −80 °C to approximately double the triflate concentration in each step. The exact triflate concentration was determined by comparison to the trimethyl(4-(trifluoromethyl)-phenyl)silane internal standard. The results are plotted in Figure 3B,C and show a clear dichotomy in the mechanism of triflate dissociation, consistent with the observed temperature-dependent rate differences listed in Figure 2C and Tables S1–S4. C-3 Benzoyl-containing mannosides **7**_**αOTf**_–**8**_**αOTf**_ showed a zeroth-order rate dependence with respect to the triflate concentration. These results indicate that internal stabilization of the C-3 benzoyl, which forms the dioxanium ion, drives triflate dissociation. In contrast, all glucosides (**11**_**αOTf**_–**14**_**αOTf**_) and both mannosides lacking the C-3 benzoyl group (**9**_**αOTf**_–**10**_**αOTf**_) show a first-order triflate concentration dependence for the rate of triflate dissociation (Figure 3B, C). These data suggest that these molecules likely undergo an S_N_2-like triflate displacement to form the β-glycosyl triflate. Furthermore, this demonstrates that internal stabilization by the C-3 acyl group is reserved for the mannose donors, while the glucose counterparts likely form β-glycosyl triflates.

**Figure 4:**
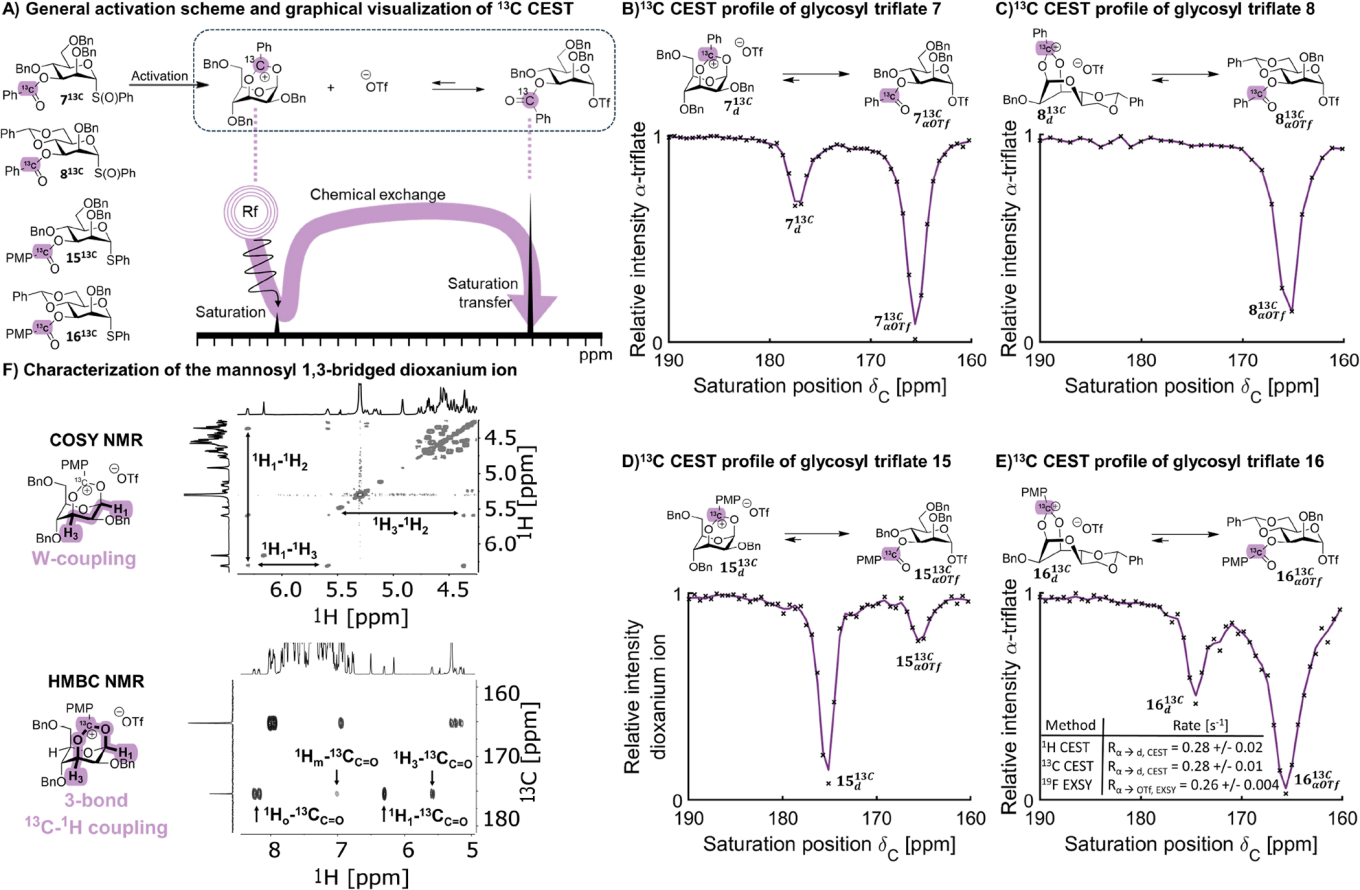
(A) Mechanism of ^13^C CEST NMR to detect neighboring group participation. (B–E) CEST profiles for ^13^C-labeled C-3 acyl-protected mannosyl and glucosyl donors. (F) Characterization of the mannosyl dioxanium ion using HMBC and COSY.

The above ^19^F EXSY studies established the rates of triflate dissociation as well as provided evidence for its mechanism but did not confirm the presence of the intermediates proposed in such mechanisms. Since EXSY can only be applied if both intermediates are readily visible in 1D NMR, it cannot be used to study the expected dioxanium ions and/or β-glycosyl triflate intermediates due to their low population at equilibrium. Hence, to solve this challenge, a different technique is needed. As mentioned above, CEST NMR is well suited to detect a very low population of “invisible” reaction intermediates that are in a chemical equilibrium with a visible reaction intermediate. This technique, originally developed as a contrasting approach in MRI,^48^ has been applied by Gschwind and co-workers to detect iminium ions and by us to detect mannosyl dioxanium ions, for example.^17,49^ The main advantage of CEST NMR is that no prior information about the chemical shift of the low-populated intermediate resonance is required since the experiment scans a given frequency domain by incrementing the saturation offset frequency while monitoring the transfer of saturation to the main observable species via chemical exchange (Figure 4A). To detect the formation of dioxanium ions, we used the ^13^C-labeled C-3 benzoyl signal belonging to the α-triflate species as the reporter peak. By plotting the degree of saturation transfer toward this reporter peak at a given frequency offset, a CEST profile is obtained (Figure 4A). In the case where saturation transfer is observed at a frequency offset different to that of the reporter peak, it shows up as a dip in the CEST profile and indicates the presence of a species that is in chemical exchange with the reporter species. Notably, the degree of saturation transfer is dependent on the observed nucleus, resonance frequency, frequency difference between the two species, the exchange rates between exchanging species, the field strength, saturation width, and saturation time. Hence, by plotting the saturation time versus the degree of saturation transfer, the interconversion kinetics of the equilibrating intermediates can be quantified.^50^ The limitations of CEST are similar to EXSY: the chemical exchange is slow on the NMR time scale (*k*_1_ + *k*_–1_ ≪ Δω_A-B_), and the detection window is defined by a combination of relative population exchanging species and longitudinal relaxation rate (*R*_1_).^48,51^ Since the dioxanium ions can only form from the C-3 benzoyl-containing compounds **7**–**8**, we investigated this set using ^13^C CEST NMR. To this end, ^13^C-labeled substrates **7**^**13C**^ and **8**^**13C**^ were prepared to enable the sensitive detection of the carbonyl quaternary carbon (SI pages S12–S31). By incrementing the saturation offset frequency while monitoring the transfer of saturation to a reporter peak (^13^C=O) on the main observable species (α-glycosyl triflate) via a chemical exchange, we investigated the detection of mannosyl and glucosyl dioxanium ion formation (Figures 4A and S13–S17). Apart from the use of a ^13^C-labeled starting material, the CEST NMR experiments were performed under identical conditions as the EXSY experiments. As expected, the mannosyl triflate **7**^**13C**^_**αOTf**_ showed saturation transfer at the chemical shift expected for the dioxanium ion (δ_C_ = 177 ppm, Figure 4B). In contrast, the mannosyl benzylidene derivative **8**^**13C**^_**αOTf**_ did not (Figure 4C), even though the formation of a dioxanium ion was expected based on an observed zeroth-order triflate dependence.

**Figure 5:**
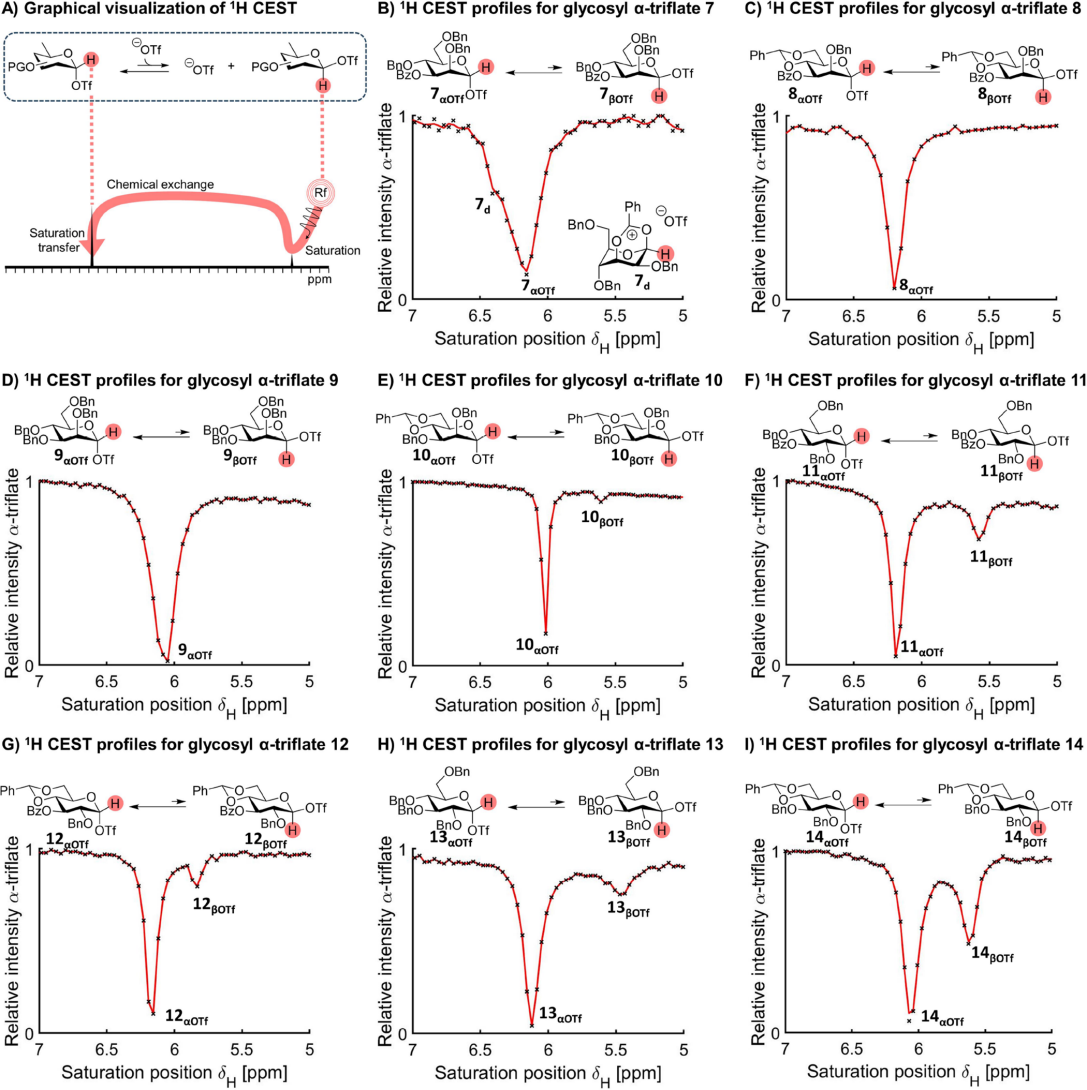
(A) Mechanism of ^1^H CEST NMR to detect β-glycosyl triflates. (B–I) ^1^H CEST profiles for **7**_**αOTf**_–**14**_**αOTf.**_.

Due to the presence of the benzylidene, the molecule is less flexible and requires the formation of a tricyclic dioxanium ion which is likely much slower and less stable than the nonbenzylidene derivative **7**_**d**_. This may explain why CEST NMR was unable to pick up the dioxanium ion as the CEST NMR technique is limited by the population of the minor exchanging species and the rates of dioxanium ion formation and consumption (*k*_α→d_ and *k*_d→α_, respectively). If the population of the intermediate is well below 1% or the exchange has moved into the intermediate or fast regime (*k*_α→d_ + *k*_d→α_ > Δϖ_dα_), then the minor exchanging species cannot be detected. Due to the intrinsically high reactivity of a tricyclic dioxanium ion, it is likely that dioxanium ion consumption (*k*_d→α_) is exceedingly fast, hence limiting detection of the ion by ^13^C CEST NMR. The corresponding glucose series (**11**^**13C**^ and **12**^**13C**^) did not show dioxanium ion formation via CEST (Figures S14 and S16), fully consistent with the first-order triflate dependence (Figure 3C). In light of the conflicting CEST results with the triflate order in the case of mannosyl triflate **8**_**αOTf**_, we reasoned that we could approach the limitation of the CEST NMR viewing window outlined above. Therefore, we wanted to boost the observable population of the dioxanium ion by preparing the corresponding *p*-anisoyl derivatives **15**^**13C**^ and **16**^**13C**^ of the mannose series, as the electron-donating methoxy group should increase the stability of the dioxanium ion and slow down the α-glycosyl triflate formation. Additionally, we performed the activation of **15**^**13C**^ and **16**^**13C**^ in the absence of TTBP to minimize the free triflate anion concentration in an effort to further boost the population of dioxanium ions.^17^ Under these conditions, saturation transfer of the dioxanium ion was observed in both cases (Figure 4D, E) at ∼175 ppm. For benzylidene derivative **16**^**13C**^, it was now possible to detect the dioxanium ion with ^13^C CEST NMR, which aligned with its zeroth-order triflate dependence. Furthermore, mannosyl donor **15**^**13C**^ showed a much higher population (∼45%) of dioxanium ion compared to benzoyl derivative **7**. Due to the very high population of dioxanium upon activating donor **15**^**13C**^, we were able to use the dioxanium ion signal as the reporter peak of the CEST NMR experiment in order to detect if there were additional intermediates in chemical exchange with it. Identical to the benzoyl derivative, the α-glycosyl triflate was the only detectable species in chemical exchange with the dioxanium ion. Fortunately, due to the high population of ion **15**^**13C**^_**d**_, we could fully characterize it using ^1^H–^13^C HMBC and ^1^H–^1^H COSY experiments (Figure 4F). We had previously reported the characterization of a similar 3-benzoyl-2,4,6-trimethylated mannosyl dioxanium ion, but these results now demonstrate its formation with relevant protecting groups, which are routinely used in oligosaccharide synthesis. Hence, although not directly characterized, we expect the characteristic saturation transfer at δ_C_ of 175 ppm for benzylidene derivative **16**^**13C**^ to correspond to the tricyclic mannosyl dioxanium ion **16**^**13C**^_**d**_. Calculations were performed to compute the chemical shift of the ^13^C-labeled dioxanium bridge which was in very good agreement with the observed chemical shift (computational chemical shift: δ_C_ 173.7 ppm, experimental chemical shift: δ_C_ 174.5 ppm, Figure S35). To further solidify this hypothesis, we measured the triflate dissociation of **16**^**13C**^_**αOTf**_ with ^19^F EXSY and dioxanium ion formation using ^1^H and ^13^C CEST NMR (pages S4 and S5). The measured rate from these techniques was 0.28 ± 0.02, 0.28 ± 0.01, and 0.26 ± 0.004 s^–1^, respectively, indicating that the processes for forming the dioxanium and dissociating the triflate group are clearly coupled (Figure 4E). From these results, it is clear that mannoside **15**^**13C**^ and **16**^**13C**^ showed the formation of the C-3 dioxanium ion, which is consistent with the α-selective glycosylation behavior of **7** and **8** and the observation that their triflate dissociation rates are irrespective of the triflate anion concentration. The C-3 acylated mannosides are a privileged class of glycosides, as all of the other cases (**9**_**αOTf**_–**14**_**αOT**_) displayed a first-order triflate anion concentration dependence for triflate dissociation. These observations are consistent with an equilibrium between an α- and β-glycosyl triflate. β-glycosyl triflates are exceedingly difficult to detect and have only been observed in the case of benzylidene-protected methylated glucose and allose donors using ^13^C-labeled monosaccharides.^23^ Only in the case of the allose, a large enough population of β-glycosyl triflate was formed that allowed for the investigation of its exchange kinetics using ^1^H EXSY.^23^ Furthermore, an equatorial triflate not stabilized by the anomeric effect formed via a conformational ring flip was observed by Van der Marel and co-workers.^52^ To the best of our knowledge, these two reports form the summative collection of observed glycosyl triflates not stabilized by the anomeric effect. To enable the detection of unstable and very-low-populated β-glycosyl triflates, we again applied CEST NMR but focused on the ^1^H and ^19^F nuclei. For ^1^H CEST NMR, the H-1 signal belonging to the α-glycosyl triflate was used as a reporter peak, and the saturation offset frequency was scanned while monitoring saturation transfer to this peak (Figure 5A). Upon detection of a possible β-glycosyl triflate, saturation transfer from an upfield peak position (δ_H_ ∼ 5.5–5.7 ppm) would be expected to be observed. None of the mannoside donors displayed a chemical equilibrium with an upfield resonance with the exception of benzylidene derivative **10**, which we tentatively assigned as β-glycosyl triflate **10**_**βOTf**_ (Figures 5B–I and S27–S34). These results are consistent with the triflate orders obtained for three of these compounds, **7** and **8** (zeroth order, no β-glycosyl triflate detected) and **10** (first order, β-glycosyl triflate detected). Only for perbenzyl mannosyl donor **9,** we did not detect a β-glycosyl triflate even though its triflate dissociation rate is on the order of first in triflate concentration. This could be due to a very low population of β-glycosyl triflate at equilibrium or the system has moved out of the slow-exchange regime, in which case it would be difficult or impossible to detect with ^1^H CEST NMR. In contrast, all of the glucose series donors (**11**–**14**) showed clear saturation transfer via an upfield peak that we presume is a β-glycosyl triflate. These results are fully consistent with their first-order triflate dependence (Figure 3B, C). We validated these results by repeating β-glycosyl triflate detection using the ^19^F channel. For ^19^F CEST NMR, the CF_3_ signal belonging to the triflate ion was used as a reporter peak, and the saturation offset frequency was scanned while monitoring saturation transfer to this peak (Figures S19–S26).

As a consequence, all ^19^F CEST NMR spectra identify chemical exchange with the “free” triflate anion pool, which can arise from either the α- or β-glycosyl triflate. The ^19^F CEST NMR results are fully consistent with those obtained from the ^1^H CEST NMR experiments. For the mannose series, the chemical exchange of the α-glycosyl triflate with the unbound triflate anion could be clearly observed via saturation transfer monitored from the α-glycosyl triflate at −75.9 ppm. Only for the benzylidene derivative **10**, an additional saturation transfer from a presumed β-glycosyl triflate was observed at δ_F_ −75.0 ppm, similar to the ^1^H CEST NMR. Moreover, in the case of the glucose series, saturation transfer from β-glycosyl triflates could be detected in all cases using ^19^F CEST NMR as was the case for the corresponding ^1^H experiments.

**Figure 6:**
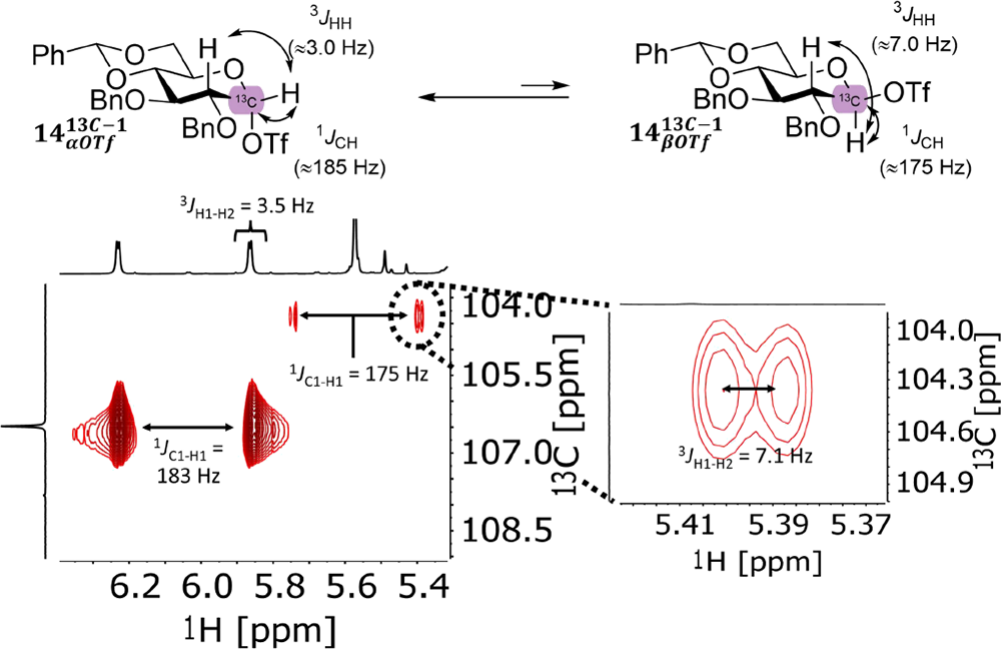
Characterization of β-glycosyl triflate **14** using HSQC without ^13^C decoupling in order to measure ^1^*J*_C1–H1_ and ^3^*J*_H1–H2_.

To obtain additional proof that the saturation transfer originates from exchange with the β-glycosyl triflate, we set out to further characterize this low-population species. From the glucose set, benzylidene derivative **14** displayed the clearest formation of this species, and we thus investigated this molecule further. The only reported direct spectroscopic evidence of a β-glycosyl triflate was obtained for a very similar compound, 4,6-benzylidene-2,3-di-*O*-methyl glucosyl-β-triflate, by Asensio and co-workers.^23^ We prepared the corresponding benzyl protected compound with a C-1 ^13^C isotope label (**14**^**13C-1**^) in order to measure the ^1^*J*_C1–H1_ and ^3^*J*_H1–H2_ coupling constants, which are indicative of the stereochemistry at C-1. Upon activation, this derivative formed a small (≈1%, SI, Figures S46 and S47) population of β-glucosyl triflate that could be characterized using a ^1^H–^13^C HSQC NMR experiment, when the ^13^C decoupler was not applied during the acquisition period (Figure 6). This experiment serves as a simple method for measuring the ^1^*J*_CH_ as well as allowing for long *F_2_* acquisition times to obtain high-resolution peaks in the ^1^H dimension suitable for measuring ^1^H–^1^H couplings. The ^1^*J*_C1–H1_ coupling constant for the minor species was determined to be 175 Hz compared to 183 Hz for the corresponding α-derivative (Figure 6). This 8 Hz decrease in coupling constant is indicative of an axial H-1 found in β-configured molecules.^53^ Most importantly, the ^3^*J*_H1–H2_ coupling constant was measured to be 7.1 Hz, which is consistent with the axial–axial coupling expected for a β-glycosyl triflate intermediate. Lastly, the chemical shifts of C-1 and H-1 are also consistent with a glycosyl triflate species, and the two ^13^C resonances at δ_C_ 106.4 ppm (α-triflate) and δ_C_ 104.3 ppm (β-triflate) as determined from the HSQC were shown to undergo chemical exchange via ^13^C CEST NMR (Figure S48).

The systematic set of exchange NMR experiments described above allows for the development of a rationale for the reaction pathways leading to the stereoselective formation of glycosylation product(s) for each of the eight glycosyl donors **7**–**14** studied. For all eight examples, the main reaction intermediate formed after activation is α-glycosyl triflate. A baseline of S_N_2-like displacement pathway leading to the β-product can therefore be expected in all cases. The SSIPs involved in the reactions could not be detected due to their instability, but both the mannose^10,25^ and glucose^25^ series are expected to react via their α-selective ^4^*H*_3_ half-chair conformers. Additional pathways proceeding via C-3 participation and the β-glycosyl triflate intermediate can also lead to α-selective product formation. The applied exchange NMR techniques have enabled the (indirect) detection of the unstable, reactive, and low-population mannosyl dioxanium ion and β-glucosyl triflate reaction intermediates. Even though these are low-population reaction intermediates and their exact abundance at equilibrium remains unknown, they can be the main product-forming species if the barrier to reaction intermediate interconversion is smaller than the barrier to product formation according to the Curtin–Hammett principle. Therefore, the rate differences of reaction intermediate interconversion and the ensuing product-forming step dictate the stereospecific outcome as outlined by Lemieux and co-workers.^20^ The stereoselectivity for all eight glycosyl donors **7**–**14** as a function of acceptor reactivity was investigated separately.^18,54^

**Figure 7:**
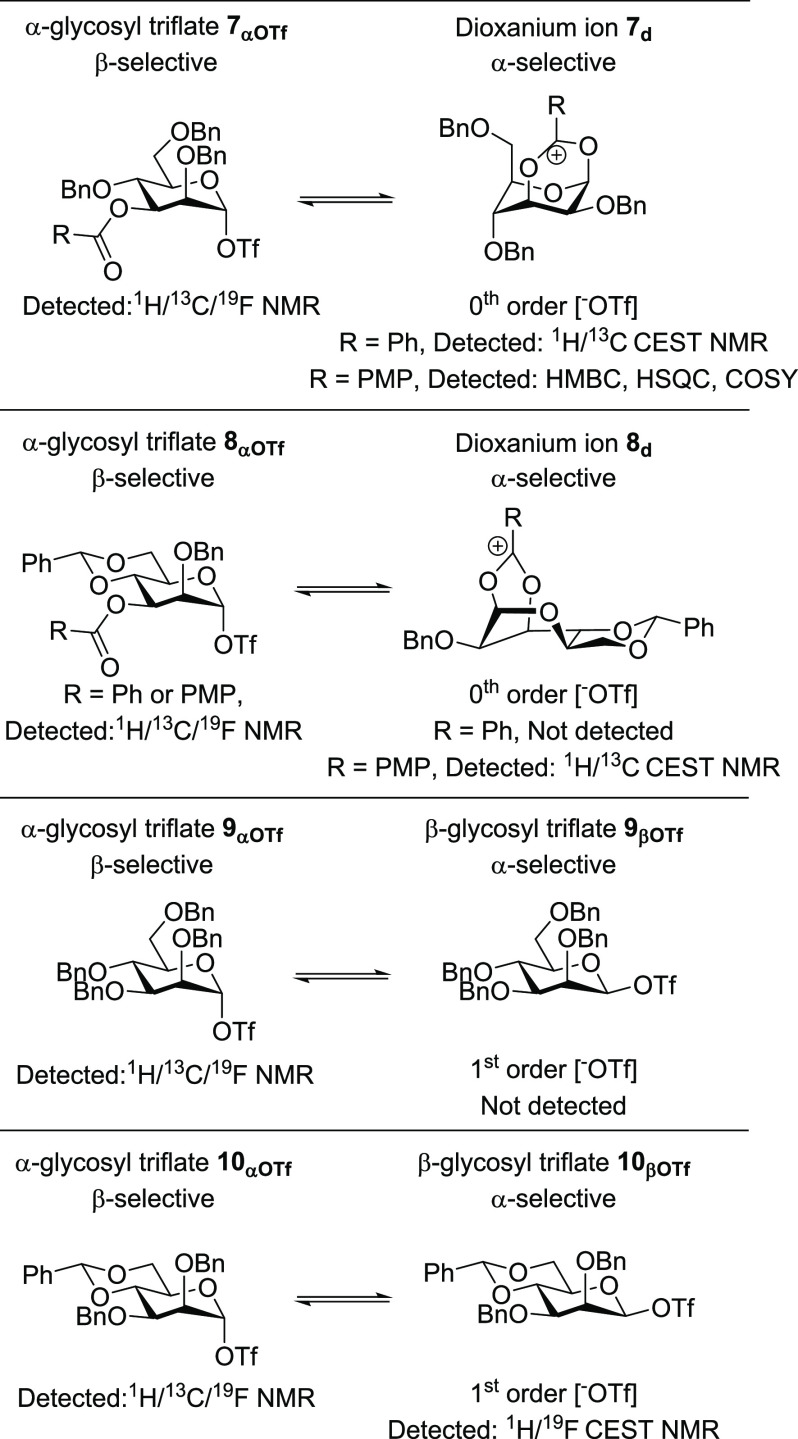
Spectroscopic summary of mannosyl intermediates.

Starting with the mannose series, C-3 benzoyl mannosyl donor **7** provides α-mannosides upon glycosylation.^17,18^ The corresponding α-glycosyl triflate **7**_**αOTf**_ is the main observable reaction intermediate but does not give direct access to the α-product via an S_N_2-like pathway. Hence, glycosylation likely takes place via rapidly equilibrating dioxanium ion **7**_**d**_ and/or β-glycosyl triflate **7**_**βOTf**_ which would afford the α-product. Dioxanium ion **7**_**d**_ was observed via CEST NMR, while the β-glycosyl triflate was not nor was it expected to be based on its zeroth-order triflate dependence. Therefore, we confidently propose that the reactive intermediate in this case is the dioxanium ion and that the system is under Curtin–Hammett control (Figure 7).

The corresponding C-3 benzoyl-4,6-benzylidene mannoside **8** shows a similar profile. The α-mannoside is formed upon glycosylation which cannot be explained via the observable α-glycosyl triflate intermediate.^55^ CEST NMR was unable to confirm the presence of dioxanium ion **8**_**d**_ or β-triflate **8**_**βOTf**_. However, the rate of triflate dissociation from **8**_**αOTf**_ was independent of the triflate concentration pointing toward triflate dissociation via C-3 participation. By substituting the C-3 benzoyl for anisoyl in order to further stabilize the cation, the resulting dioxanium ion was detected for the first time. The existence of this intermediate has long been debated but was never characterized.^5,6^^5,14,55^ However, the experiments reported herein now support the formation of **8**_**d**_ as the chemical shift, triflate order, and glycosylation stereoselectivity all support its existence and role as a product-forming intermediate (Figure 7).

Perbenzyl mannoside **9** is a much less selective glycosylation donor. The first order of the triflate concentration on triflate dissociation supports the formation of a β-glycosyl triflate although this species could not be detected via the exchange NMR experiments. We hypothesize that a dynamic equilibrium of α- and β-glycosyl triflates and possibly the SSIP is present leading to various product-forming pathways and hence mixed stereoselectivity.

4,6-Benzylidene mannoside **10** is known to provide β-products upon glycosylation.^41^ This can be explained by an S_N_2-like displacement of the corresponding α-contact ion pair.^24^ Hence, in this case, the main observed reaction intermediate is assigned to the reactive intermediate. Interestingly, CEST NMR did show the presence of an equilibrium with the β-triflate intermediate, which is consistent with its first-order triflate dependence. This equilibrium is not relevant for product formation with most nucleophiles and therefore represents a situation in which the β-triflate is the less reactive species (Figure 7).

**Figure 8:**
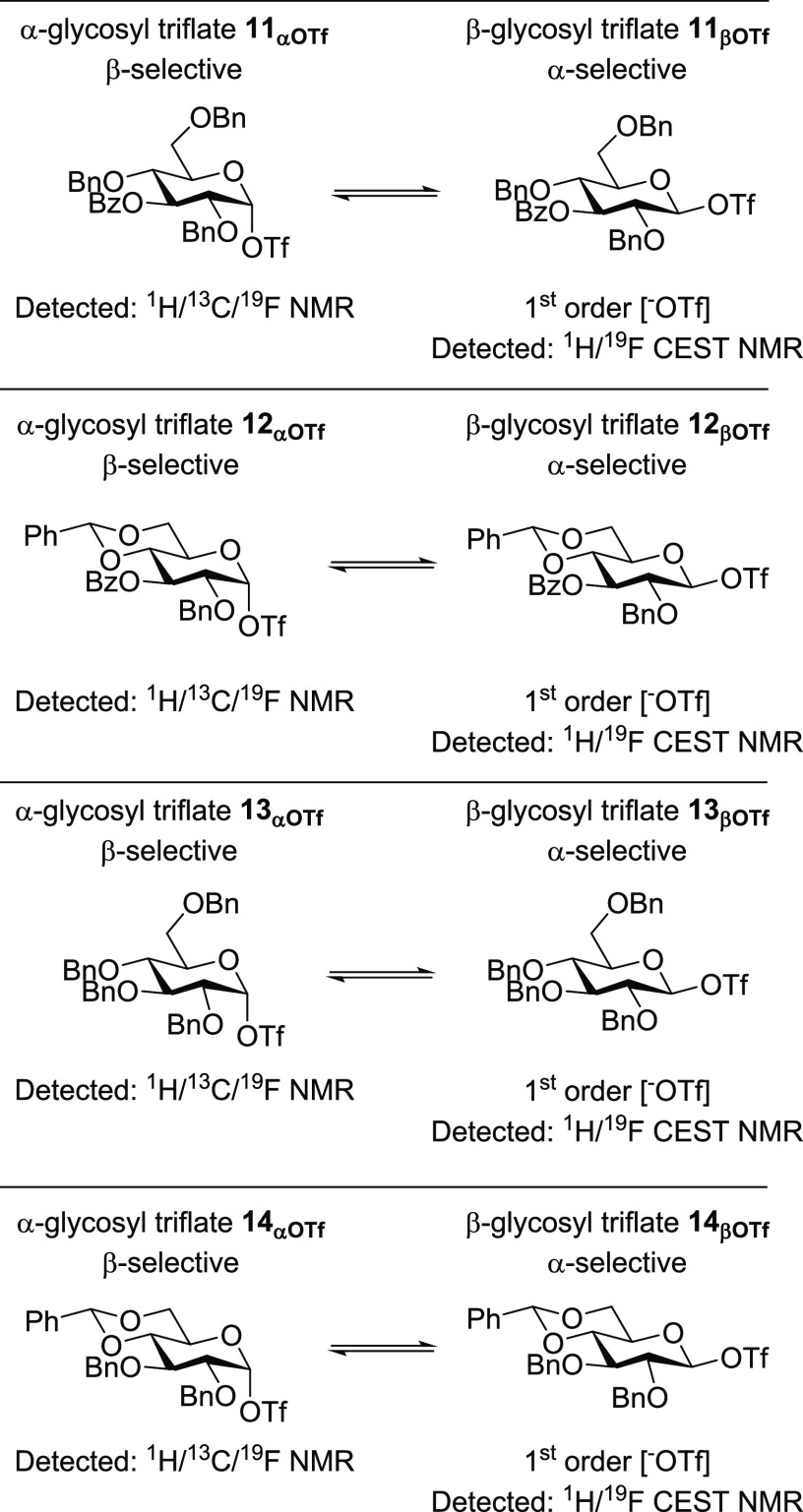
Spectroscopic summary of glucosyl intermediates.

The glucose series showed very different stereoselectivity in glycosylations compared to the corresponding mannose series as noted earlier by Crich and co-workers.^30^ In sharp contrast to the mannose series, no evidence for dioxanium ion formation by a C-3 acyl neighboring group in **11** and **12** was found (Figure 8). The origin of this striking difference was investigated further in a separate study.^54^ Both molecules form β-glycosyl triflates, whereas the mannosides do not, but these lead to a lower α-selectivity than the dioxanium ions in the case of their mannose counterparts.^18^ Also the origin of this clear divergence was investigated further in a separate study.^54^

A role for the SSIP, which is expected to adopt an ^4^*H*_3_ half-chair conformer and drives the formation of α-product as reported by Codée and co-workers, cannot be excluded.^25^ The simultaneous operation of multiple product-forming pathways likely leads to lower stereoselectivity in these cases. The two glucosides **13** and **14** lacking the C-3 benzoyl group show a similar, moderate stereoselectivity compared to their C-3 benzoyl counterparts (Figure 8).^18^ This underscores the lack of stereo-directing capability of the C-3 substituent in glucose.^18^ The benzylidene derivative **14** has been demonstrated to be α-selective in contrast to its mannose derivative.^30^ Since **14** also clearly showed to be in equilibrium with the β-glycosyl triflate, we assign this as a major reactive intermediate driving α-product formation (Figure 8).

## Conclusions

We were able to directly and indirectly detect the presence of low-population reaction intermediates via their chemical equilibrium with the readily observable α-glycosyl triflate for a collection of eight gluco- and mannosides bearing either C-3 acyl or benzyl substituent. The stability of α-glycosyl triflates and their mechanism for dissociation could be readily measured by using ^19^F EXSY NMR. This allowed for their role in glycosylation reactions to be ascertained. Furthermore, equilibria with low-population intermediates such as dioxanium ions and β-glycosyl triflates could be detected using ^13^C CEST, ^1^H CEST, and ^19^F CEST NMR. Finally, selected examples of the dioxanium ion and β-glycosyl triflate were characterized using classical NMR techniques to unequivocally establish their identity. The results provide the first mechanistic proof for the existence of mannosyl dioxanium ions and β-glucosyl triflates utilizing relevant protecting groups under actual reaction conditions. These observations allow for the rationalization of their observed α-selectivity and demonstrate the power of chemical exchange NMR to detect transient intermediates. Ultimately, we aim to build a catalog of kinetic parameters, allowing for the understanding and eventual prediction of glycosylation reactions. We expect this technique to be applicable to various other types of glycosylation reaction intermediates such as additives (e.g., DMF, PPh_3_O) and coordinating solvents (e.g., Et_2_O, MeCN). Finally, the application of the workflow laid out herein should be applicable to other types of reactions that are under Curtin–Hammett control.

## Supporting Information Available

Synthetic procedures for the synthesis of donors **7**-**14**, **15**^13^C, **16**^13^C, and the precursor for **14**_αOTf_(^13^C-1). Theoretical and practical description of the EXSY and CEST experiments, VT-NMR procedures, and additional variable temperature NMR spectra and raw ^19^F EXSY, ^13^C CEST, and ^1^H CEST NMR data (PDF)

## References

[ref1] Boltje T. J.; Buskas T.; Boons G.-J. Opportunities and challenges in synthetic oligosaccharide and glycoconjugate research. Nature Chem. 2009, 1 (8), 611–622. 10.1038/nchem.399. 20161474 PMC2794050

[ref2] Lemieux R. U. Some implications in carbohydrate chemistry of theories relating to the mechanisms of replacement reactions. Adv. Carbohydr. Chem. 1954, 9, 1–57. 10.1016/S0096-5332(08)60371-9. 13217906

[ref3] Frush H. L.; Isbell H. S. Sugar acetates, acetylglycosyl halides and orthoacetates in relation to the Walden inversion. Journal of research of the National Bureau of Standards 1941, 27, 413 10.6028/jres.027.028.

[ref4] Hettikankanamalage A. A.; Lassfolk R.; Ekholm F. S.; Leino R.; Crich D. Mechanisms of Stereodirecting Participation and Ester Migration from Near and Far in Glycosylation and Related Reactions. Chem. Rev. 2020, 120 (15), 7104–7151. 10.1021/acs.chemrev.0c00243. 32627532 PMC7429366

[ref5] Crich D.; Hu T.; Cai F. Does neighboring group participation by non-vicinal esters play a role in glycosylation reactions? Effective probes for the detection of bridging intermediates. Journal of organic chemistry 2008, 73 (22), 8942–8953. 10.1021/jo801630m. 18939876 PMC2669227

[ref6] Baek J. Y.; Lee B.-Y.; Jo M. G.; Kim K. S. β-Directing Effect of Electron-Withdrawing Groups at O-3, O-4, and O-6 Positions and α-Directing Effect by Remote Participation of 3-O-Acyl and 6-O-Acetyl Groups of Donors in Mannopyranosylations. J. Am. Chem. Soc. 2009, 131 (48), 17705–17713. 10.1021/ja907252u. 19908841

[ref7] Lei J.-C.; Ruan Y.-X.; Luo S.; Yang J.-S. Stereodirecting Effect of C3-Ester Groups on the Glycosylation Stereochemistry of L-Rhamnopyranose Thioglycoside Donors: Stereoselective Synthesis of α- and β-L-Rhamnopyranosides. Eur. J. Org. Chem. 2019, 2019 (37), 6377–6382. 10.1002/ejoc.201901186.

[ref8] Demchenko A. V.; Rousson E.; Boons G.-J. Stereoselective 1,2-cis-galactosylation assisted by remote neighboring group participation and solvent effects. Tetrahedron Lett. 1999, 40 (36), 6523–6526. 10.1016/S0040-4039(99)01203-4.

[ref9] Baek J. Y.; Kwon H.-W.; Myung S. J.; Park J. J.; Kim M. Y.; Rathwell D. C. K.; Jeon H. B.; Seeberger P. H.; Kim K. S. Directing effect by remote electron-withdrawing protecting groups at O-3 or O-4 position of donors in glucosylations and galactosylations. Tetrahedron 2015, 71 (33), 5315–5320. 10.1016/j.tet.2015.06.014.

[ref10] Ayala L.; Lucero C. G.; Romero J. A.; Tabacco S. A.; Woerpel K. A. Stereochemistry of nucleophilic substitution reactions depending upon substituent: evidence for electrostatic stabilization of pseudoaxial conformers of oxocarbenium ions by heteroatom substituents. J. Am. Chem. Soc. 2003, 125 (50), 15521–8. 10.1021/ja037935a. 14664599

[ref11] Ma Y.; Lian G.; Li Y.; Yu B. Identification of 3,6-di-O-acetyl-1,2,4-O-orthoacetyl-α-d-glucopyranose as a direct evidence for the 4-O-acyl group participation in glycosylation. Chem. Commun. 2011, 47 (26), 7515–7517. 10.1039/c1cc11680k. 21625694

[ref12] Komarova B. S.; Orekhova M. V.; Tsvetkov Y. E.; Nifantiev N. E. Is an acyl group at O-3 in glucosyl donors able to control α-stereoselectivity of glycosylation? The role of conformational mobility and the protecting group at O-6. Carbohydr. Res. 2014, 384, 70–86. 10.1016/j.carres.2013.11.016. 24368161

[ref13] Dejter-juszynski M.; Flowers H. M. Studies on the koenigs-knorr reaction: Part IV: The effect of participating groups on the stereochemistry of disaccharide formation. Carbohydr. Res. 1973, 28 (1), 61–74. 10.1016/S0008-6215(00)82857-8.

[ref14] Mcmillan T. F.; Crich D. Influence of 3-Thio Substituents on Benzylidene-Directed Mannosylation. Isolation of a Bridged Pyridinium Ion and Effects of 3-O-Picolyl and 3-S-Picolyl Esters. Eur. J. Org. Chem. 2022, 2022 (20), e202200320 10.1002/ejoc.202200320. PMC9632450 36340645

[ref15] Elferink H.; Remmerswaal W. A.; Houthuijs K. J.; Jansen O.; Hansen T.; Rijs A. M.; Berden G.; Martens J.; Oomens J.; Codée J. D. C.; Boltje T. J. Competing C-4 and C-5-Acyl Stabilization of Uronic Acid Glycosyl Cations. Chem. – Eur. J. 2022, 28 (63), e202201724 10.1002/chem.202201724. 35959853 PMC9825916

[ref16] Remmerswaal W. A.; Houthuijs K. J.; Van de ven R.; Elferink H.; Hansen T.; Berden G.; Overkleeft H. S.; Van der marel G. A.; Rutjes F. P. J. T.; Filippov D. V.; Boltje T. J.; Martens J.; Oomens J.; Codée J. D. C. Stabilization of Glucosyl Dioxolenium Ions by “Dual Participation” of the 2,2-Dimethyl-2-(ortho-nitrophenyl)acetyl (DMNPA) Protection Group for 1,2-cis-Glucosylation. Journal of Organic Chemistry 2022, 87 (14), 9139–9147. 10.1021/acs.joc.2c00808. 35748115 PMC9295149

[ref17] De kleijne F. F. J.; Elferink H.; Moons S. J.; White P. B.; Boltje T. J. Characterization of Mannosyl Dioxanium Ions in Solution Using Chemical Exchange Saturation Transfer NMR Spectroscopy. Angew. Chem., Int. Ed. 2022, 61 (6), e202109874 10.1002/anie.202109874. PMC9305821 34519403

[ref18] Hansen T.; Elferink H.; Van hengst J. M. A.; Houthuijs K. J.; Remmerswaal W. A.; Kromm A.; Berden G.; Van der vorm S.; Rijs A. M.; Overkleeft H. S.; Filippov D. V.; Rutjes F. P. J. T.; Van der marel G. A.; Martens J.; Oomens J.; Codée J. D. C.; Boltje T. J. Characterization of glycosyl dioxolenium ions and their role in glycosylation reactions. Nat. Commun. 2020, 11 (1), 2664 10.1038/s41467-020-16362-x. 32471982 PMC7260182

[ref19] Elferink H.; Mensink R. A.; Castelijns W. W. A.; Jansen O.; Bruekers J. P. J.; Martens J.; Oomens J.; Rijs A. M.; Boltje T. J. The Glycosylation Mechanisms of 6,3-Uronic Acid Lactones. Angew. Chem., Int. Ed. 2019, 58 (26), 8746–8751. 10.1002/anie.201902507. 31017713

[ref20] Lemieux R. U.; Hendriks K. B.; Stick R. V.; James K. Halide ion catalyzed glycosidation reactions. Syntheses of.alpha.-linked disaccharides. J. Am. Chem. Soc. 1975, 97 (14), 4056–4062. 10.1021/ja00847a032.

[ref21] Lu S.-R.; Lai Y.-H.; Chen J.-H.; Liu C.-Y.; Mong K.-K. T. Dimethylformamide: An Unusual Glycosylation Modulator. Angew. Chem., Int. Ed. 2011, 50 (32), 7315–7320. 10.1002/anie.201100076. 21688356

[ref22] Crich D.; Sun S. Are Glycosyl Triflates Intermediates in the Sulfoxide Glycosylation Method? A Chemical and 1H, 13C, and 19F NMR Spectroscopic Investigation. J. Am. Chem. Soc. 1997, 119 (46), 11217–11223. 10.1021/ja971239r.

[ref23] Santana A. G.; Montalvillo-jiménez L.; Díaz-casado L.; Corzana F.; Merino P.; Cañada F. J.; Jiménez-osés G.; Jiménez-barbero J.; Gómez A. M.; Asensio J. L. Dissecting the Essential Role of Anomeric β-Triflates in Glycosylation Reactions. J. Am. Chem. Soc. 2020, 142 (28), 12501–12514. 10.1021/jacs.0c05525. 32579343

[ref24] Crich D.; Chandrasekera N. S. Mechanism of 4,6-O-Benzylidene-Directed β-Mannosylation as Determined by α-Deuterium Kinetic Isotope Effects. Angew. Chem., Int. Ed. 2004, 43 (40), 5386–5389. 10.1002/anie.200453688. 15468095

[ref25] Hansen T.; Lebedel L.; Remmerswaal W. A.; Van der vorm S.; Wander D. P. A.; Somers M.; Overkleeft H. S.; Filippov D. V.; Désiré J.; Mingot A.; Bleriot Y.; Van der marel G. A.; Thibaudeau S.; Codée J. D. C. Defining the SN1 Side of Glycosylation Reactions: Stereoselectivity of Glycopyranosyl Cations. ACS Central Science 2019, 5 (5), 781–788. 10.1021/acscentsci.9b00042. 31139714 PMC6535769

[ref26] Franconetti A.; Ardá A.; Asensio J. L.; Blériot Y.; Thibaudeau S.; Jiménez-barbero J. Glycosyl Oxocarbenium Ions: Structure, Conformation, Reactivity, and Interactions. Acc. Chem. Res. 2021, 54 (11), 2552–2564. 10.1021/acs.accounts.1c00021. 33930267 PMC8173606

[ref27] Huang M.; Retailleau P.; Bohé L.; Crich D. Cation Clock Permits Distinction Between the Mechanisms of α- and β-O- and β-C-Glycosylation in the Mannopyranose Series: Evidence for the Existence of a Mannopyranosyl Oxocarbenium Ion. J. Am. Chem. Soc. 2012, 134 (36), 14746–14749. 10.1021/ja307266n. 22920536 PMC3448556

[ref28] Chatterjee S.; Moon S.; Hentschel F.; Gilmore K.; Seeberger P. H. An Empirical Understanding of the Glycosylation Reaction. J. Am. Chem. Soc. 2018, 140 (38), 11942–11953. 10.1021/jacs.8b04525. 30125122

[ref29] Satoh H.; Hansen H. S.; Manabe S.; Van gunsteren W. F.; Hünenberger P. H. Theoretical Investigation of Solvent Effects on Glycosylation Reactions: Stereoselectivity Controlled by Preferential Conformations of the Intermediate Oxacarbenium-Counterion Complex. J. Chem. Theory Comput. 2010, 6 (6), 1783–1797. 10.1021/ct1001347. 26615839

[ref30] Crich D.; Cai W. Chemistry of 4,6-O-Benzylidene-d-glycopyranosyl Triflates: Contrasting Behavior between the Gluco and Manno Series. Journal of Organic Chemistry 1999, 64 (13), 4926–4930. 10.1021/jo990243d. 11674572

[ref31] Van der vorm S.; Hansen T.; Van hengst J. M. A.; Overkleeft H. S.; Van der marel G. A.; Codée J. D. C. Acceptor reactivity in glycosylation reactions. Chem. Soc. Rev. 2019, 48 (17), 4688–4706. 10.1039/C8CS00369F. 31287452

[ref32] Andreana P. R.; Crich D. Guidelines for O-Glycoside Formation from First Principles. ACS Central Science 2021, 7 (9), 1454–1462. 10.1021/acscentsci.1c00594. 34584944 PMC8461634

[ref33] Crich D. En route to the transformation of glycoscience: A chemist’s perspective on internal and external crossroads in glycochemistry. J. Am. Chem. Soc. 2021, 143 (1), 17–34. 10.1021/jacs.0c11106. 33350830 PMC7856254

[ref34] Adero P. O.; Amarasekara H.; Wen P.; Bohé L.; Crich D. The Experimental Evidence in Support of Glycosylation Mechanisms at the S(N)1-S(N)2 Interface. Chem. Rev. 2018, 118 (17), 8242–8284. 10.1021/acs.chemrev.8b00083. 29846062 PMC6135681

[ref35] Frihed T. G.; Bols M.; Pedersen C. M. Mechanisms of glycosylation reactions studied by low-temperature nuclear magnetic resonance. Chem. Rev. 2015, 115 (11), 4963–5013. 10.1021/cr500434x. 25923428

[ref36] Braak F. t.; Elferink H.; Houthuijs K. J.; Oomens J.; Martens J.; Boltje T. J. Characterization of Elusive Reaction Intermediates Using Infrared Ion Spectroscopy: Application to the Experimental Characterization of Glycosyl Cations. Acc. Chem. Res. 2022, 55, 1669–1679. 10.1021/acs.accounts.2c00040. 35616920 PMC9219114

[ref37] Elferink H.; Severijnen M. E.; Martens J.; Mensink R. A.; Berden G.; Oomens J.; Rutjes F. P. J. T.; Rijs A. M.; Boltje T. J. Direct Experimental Characterization of Glycosyl Cations by Infrared Ion Spectroscopy. J. Am. Chem. Soc. 2018, 140 (19), 6034–6038. 10.1021/jacs.8b01236. 29656643 PMC5958338

[ref38] Mucha E.; Marianski M.; Xu F.-F.; Thomas D. A.; Meijer G.; Von helden G.; Seeberger P. H.; Pagel K. Unravelling the structure of glycosyl cations via cold-ion infrared spectroscopy. Nat. Commun. 2018, 9 (1), 4174 10.1038/s41467-018-07184-z. 30301896 PMC6177480

[ref39] Martin A.; Arda A.; Désiré J.; Martin-mingot A.; Probst N.; Sinaÿ P.; Jiménez-barbero J.; Thibaudeau S.; Blériot Y. Catching elusive glycosyl cations in a condensed phase with HF/SbF_5_ superacid. Nat. Chem. 2016, 8 (2), 186–91. 10.1038/nchem.2399. 26791903

[ref40] Ben-tal Y.; Boaler P. J.; Dale H. J. A.; Dooley R. E.; Fohn N. A.; Gao Y.; García-domínguez A.; Grant K. M.; Hall A. M. R.; Hayes H. L. D.; Kucharski M. M.; Wei R.; Lloyd-jones G. C. Mechanistic analysis by NMR spectroscopy: A users guide. Prog. Nucl. Magn. Reson. Spectrosc. 2022, 129, 28–106. 10.1016/j.pnmrs.2022.01.001. 35292133

[ref41] Crich D. Mechanism of a chemical glycosylation reaction. Accounts of chemical research 2010, 43 (8), 1144–1153. 10.1021/ar100035r. 20496888

[ref42] D’angelo K. A.; Taylor M. S. Borinic Acid Catalyzed Stereo- and Regioselective Couplings of Glycosyl Methanesulfonates. J. Am. Chem. Soc. 2016, 138 (34), 11058–11066. 10.1021/jacs.6b06943. 27533523

[ref43] Aubry S.; Sasaki K.; Sharma I.; Crich D. Influence of protecting groups on the reactivity and selectivity of glycosylation: chemistry of the 4,6-o-benzylidene protected mannopyranosyl donors and related species. Top Curr. Chem. 2010, 301, 141–88. 10.1007/128_2010_102. 21240602

[ref44] Kahne D.; Walker S.; Cheng Y.; Van engen D. Glycosylation of unreactive substrates. J. Am. Chem. Soc. 1989, 111 (17), 6881–6882. 10.1021/ja00199a081.

[ref45] Fraser-reid B.; Wu Z.; Andrews C. W.; Skowronski E.; Bowen J. P. Torsional effects in glycoside reactivity: saccharide couplings mediated by acetal protecting groups. J. Am. Chem. Soc. 1991, 113 (4), 1434–1435. 10.1021/ja00004a066.

[ref46] Andrews C. W.; Rodebaugh R.; Fraser-reid B. A Solvation-Assisted Model for Estimating Anomeric Reactivity. Predicted versus Observed Trends in Hydrolysis of n-Pentenyl Glycosides1. Journal of Organic Chemistry 1996, 61 (16), 5280–5289. 10.1021/jo9601223.

[ref47] Jensen H. H.; Nordstro̷m L. U.; Bols M. The Disarming Effect of the 4,6-Acetal Group on Glycoside Reactivity: Torsional or Electronic?. J. Am. Chem. Soc. 2004, 126 (30), 9205–9213. 10.1021/ja047578j. 15281809

[ref48] Van zijl P. C.; Yadav N. N. Chemical exchange saturation transfer (CEST): what is in a name and what isn’t?. Magn Reson Med. 2011, 65 (4), 927–48. 10.1002/mrm.22761. 21337419 PMC3148076

[ref49] Lokesh N.; Seegerer A.; Hioe J.; Gschwind R. M. Chemical Exchange Saturation Transfer in Chemical Reactions: A Mechanistic Tool for NMR Detection and Characterization of Transient Intermediates. J. Am. Chem. Soc. 2018, 140 (5), 1855–1862. 10.1021/jacs.7b12343. 29336150 PMC6301330

[ref50] Serianni A. S.; Pierce J.; Huang S. G.; Barker R. Anomerization of furanose sugars: kinetics of ring-opening reactions by proton and carbon-13 saturation-transfer NMR spectroscopy. J. Am. Chem. Soc. 1982, 104 (15), 4037–4044. 10.1021/ja00379a001.

[ref51] Woods M.; Woessner D. E.; Sherry A. D. Paramagnetic lanthanide complexes as PARACEST agents for medical imaging. Chem. Soc. Rev. 2006, 35 (6), 500–511. 10.1039/b509907m. 16729144 PMC2718840

[ref52] Walvoort M. T. C.; Lodder G.; Mazurek J.; Overkleeft H. S.; Codée J. D. C.; Van der marel G. A. Equatorial Anomeric Triflates from Mannuronic Acid Esters. J. Am. Chem. Soc. 2009, 131 (34), 12080–12081. 10.1021/ja905008p. 19663422

[ref53] Bock K.; Pedersen C. A study of 13CH coupling constants in hexopyranoses. J. Chem. Soc., Perkin Trans. 1974, 3, 293–297. 10.1039/p29740000293.

[ref54] Remmerswaal W.; Elferink H.; Houthuijs K.; Hansen T.; Ter braak F.; Berden G.; Van der vorm S.; Martens J.; Oomens J.; Van der marel G.; Boltje T.; Codée J. Anomeric Triflates vs Dioxanium ions: Different Product-Forming Intermediates from 1-Thiophenyl-2-O-Benzyl-3-O-Benzoyl-4, 6-O-Benzylidene-Mannose and Glucose. ChemRxiv 2023, 10.26434/chemrxiv-2023-t45q6. PMC10845153 38235652

[ref55] Crich D.; Cai W.; Dai Z. Highly Diastereoselective α-Mannopyranosylation in the Absence of Participating Protecting Groups. Journal of Organic Chemistry 2000, 65 (5), 1291–1297. 10.1021/jo9910482. 10814088

