# Supplementary material for: Detection and Characterization of Rapidly Equilibrating Glycosylation Reaction Intermediates Using Exchange NMR
Source: J Am Chem Soc. 2023 Nov 27;145(48):26190–201. doi: 10.1021/jacs.3c08709 (PMC10704605; doi:10.1021/jacs.3c08709)
Supplement: Supplementary file 1 — ja3c08709_si_001.pdf [file ja3c08709_si_001.pdf]

Detection and Characterization of Rapidly Equilibrating Glycosylation Reaction Intermediates Using Exchange NMR

Supporting information

Frank F. J. de Kleijne[‡], Floor ter Braak[‡], Dimitrios Piperoudis, Peter H. Moons, Sam J. Moons, Hidde Elferink, Paul B. White*, Thomas J. Boltje*

Radboud University, Institute for Molecules and Materials (IMM), Synthetic Organic Chemistry, 6525 AJ, Nijmegen, The Netherlands

[‡]These authors contributed equally
*Corresponding authors
Email: Paul.White@ru.nl, Thomas.Boltje@ru.nl

Table of Contents

Theoretical background S2
- Initial rate approximation S2
- Chemical Exchange Saturation Transfer NMR (CEST NMR) kinetic experiments S4

Experimental details S7
- Instrumentation S7
- Chemical Exchange Saturation Transfer NMR (CEST NMR) spectroscopy S7
- Selective ^{19}F Exchange NMR (EXSY NMR) spectroscopy S7
- Sample preparation variable temperature NMR (VT NMR) S8
- EXSY Acquisition S9
- CEST Profile Acquisition S9
- CEST Kinetics Acquisition S9

General synthetic methods S10

Chemical synthesis S10

Supporting VT NMR experimental data S32
- Activation spectra for glycosyl donors S32
- Supporting rates for triflate dependant α-triflate dissociation S40
- Effect of benzyl- and methyl-2,4,6-tri-*O*-protecting groups on mannosyl dioxanium ion formation and α-triflate stability S41
- Supporting CEST profiles overlaid with 1D NMR spectra S42
  - ^{13}C S42
  - ^{19}F S45
  - ^{1}H S49
- Computed NMR Chemical Shifts of $\mathbf{8_d}$, $\mathbf{8_{\alpha OTf}}$, $\mathbf{16_d}$, and $\mathbf{16_{\alpha OTf}}$ S54
- CEST kinetic experiments on 3-*O*-(4-methoxybenzoyl-α-^{13}C)-4,6-benzylidene-2-OBn mannosyl α-triflate S55
- Characterization experiment mannosyl dioxanium ion S60
- Characterization experiment glucosyl β-triflate S62
- T1 measurement at -80 °C S64

References S65

Data
- EXSY kinetic data at variable temperatures S66
- EXSY kinetic data at variable tetrabutylammonium triflate concentrations S85
- Synthetic spectra S106
- Data CEST profiles S141
- Optimized Computation Coordinates for $\mathbf{8_d}$, $\mathbf{8_{\alpha OTf}}$, $\mathbf{16_d}$, and $\mathbf{16_{\alpha OTf}}$. S170

Theoretical background

Initial rate approximation: As outlined in the main text, the *α*-triflate dissociates to form triflate anion in two possible mechanisms (Eq. S1 and S2). The rate law of the corresponding reactions are described in equation S3 and S4 for the intramolecular glycosyl stabilization and intermolecular glycosyl stabilization respectively. Both processes can proceed simultaneously, hence the overall rate law could be a combination of both (Eq. S5).

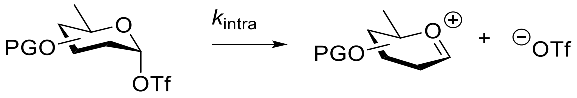

Eq. S1

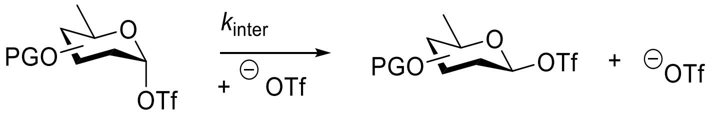

Eq. S2

$$\frac{d[OTf]}{dt} = -\frac{d[\alpha]}{dt} = R_{a\rightarrow OTf} = k_{inter}[\alpha]$$ Eq. S3

$$\frac{d[OTf]}{dt} = -\frac{d[\alpha]}{dt} = R_{a\rightarrow OTf} = k_{intra}[\alpha][^{-}OTf]$$ Eq. S4

$$\frac{d[OTf]}{dt} = -\frac{d[\alpha]}{dt} = R_{a\rightarrow OTf} = k_{inter}[\alpha] + k_{intra}[\alpha][^{-}OTf]$$ Eq. S5

Selective 1D ^{19}F EXSY NMR is a suitable method to study the exchange. By applying a selective excitation pulse on the α-triflate resonance, formation of triflate anion can be measured despite the high population triflate already present in the reaction mixture. This is possible for two main reasons: 1) the resonances of both the *α*-triflate and triflate anion are sufficiently separated to selectively excite the α-triflate in ^{19}F NMR, and, 2) only excited-state nuclei are detected in EXSY NMR. Varying the delay (mix time, τ_m) between the excitation of the *α*-triflate resonance and the spectrum acquisition allows to measure different degrees of conversion for the *α*-triflate into the triflate anion resonance. Eventually, by applying an 'infinitely long' mix time, the equilibrium ratio of *α*-triflate and triflate anion is obtained Figure S1).

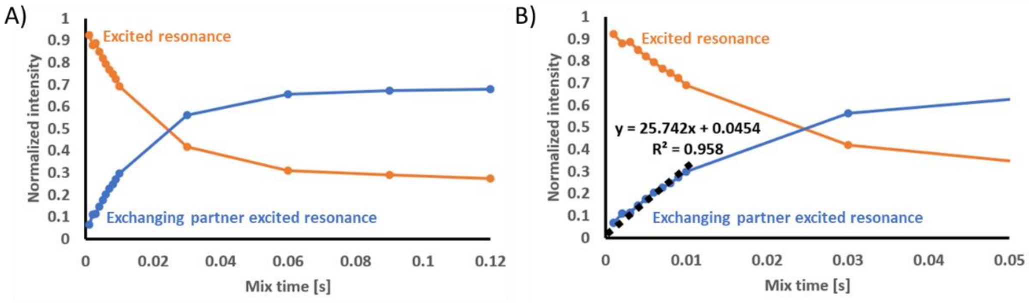

Figure S1: A) Correlation of EXSY mix time to intensity selected/excited resonance and its exchanging partner that is forming. B) Displaying the linear initial rate approximation.

The initial triflate formation is linear and kinetics could be described according to the initial rate approximation. Over the initial linear interval, the reaction rate is the α-triflate consumption and triflate anion formation (Eq. S6 and S7). Herein, $[\alpha]_t$ = concentration excited state α-triflate a set mix time after applying the excitation pulse on the α-triflate

resonance; [**α**]$_0$ = concentration excited state α-triflate directly after applying a selective excitation pulse on the α-triflate resonance; [$^-$**OTf**]$_t$ = concentration excited state $^-$OTf a set mix time after applying the excitation pulse on the α-triflate resonance; [$^-$**OTf**]$_0$ = concentration excited state $^-$OTf directly after applying a selective excitation pulse on the α-triflate resonance (hence, [$^-$**OTf**]$_0$ = 0). Substituting equation S5 into equation S6 gives the concentration excited state α-triflate in terms of concentration and mix time (Eq. S8).

$$\frac{[\alpha]_t - [\alpha]_0}{\tau_m} = -R_{\alpha \to OTf} \quad \text{Eq. S6}$$

$$\frac{[^-OTf]_t - [^-OTf]_0}{\tau_m} = R_{\alpha \to OTf} \quad \text{Eq. S7}$$

$$[\alpha]_t = [\alpha]_0 - (k_{inter}[\alpha] + k_{intra}[\alpha][^-OTf]) \times \tau_m \quad \text{Eq. S8}$$

At the very start of the reaction, the concentration *α*-triflate deviates only marginally compared to the starting concentration ([**α**]$_0$). In accordance to the initial rate approximation, equation S8 becomes equation S9. Additionally, within the initial rate approximation, only an α-triflate conversion of about 5-15% is recorded. Therefore, the concentration excited state triflate ([$^-$**OTf**]) is sufficiently small (especially compared to the bulk concentration non-excited triflate anion ([$^-$OTf])) such that the backwards reaction can be neglected. Subsequently, dividing the equation by [**α**]$_0$ simplifies the equation to S10.

$$[\alpha]_t = [\alpha]_0 - (k_{inter}[\alpha]_0 + k_{intra}[\alpha]_0[^-OTf]) \times \tau_m \quad \text{Eq. S9}$$

$$\frac{[\alpha]_t}{[\alpha]_0} = 1 - (k_{inter} + k_{intra}[^-OTf]) \times \tau_m \quad \text{Eq. S10}$$

Within NMR spectroscopy, the concentration is proportional (with a constant, c) related to the absolute integral ($\int I_x$) of the observed resonances (Eq. S11 and S12). Substituting equation S10 with S11 and S12 gives the absolute integral of the excited state *α*-triflate resonance as function of mix time (Eq. S13).

$$\int I_{\alpha,t} = c \times [\alpha]_t \quad \text{Eq. S11}$$

$$\int I_{\alpha,0} = c \times [\alpha]_0 \quad \text{Eq. S12}$$

$$\frac{\int I_{\alpha,t}}{\int I_{\alpha,0}} = 1 - (k_{inter} + k_{intra}[^-OTf]) \times \tau_m \quad \text{Eq. S13}$$

One complication is that T^1 relaxation occurs during the mixing time, which will reduce the absolute integral or intensity of the selected and exchanged resonances over time and plotting S13 will result in a multiexponential decay process if $k \leq T_1^{-1}$. This can be easily taken into account if the T_1 is known for each in the absence of exchange. However, if the T_1s for the species are very similar and thus experience similar rates of relaxation, then an approximation can be made where the integral of the α-triflate at t=0 ($I_{\alpha,0}$) is the sum of the integrals of the α-triflate and triflate anion at a given mix time (Eq. S14). This then allows Equation S13 to be rewritten in an internally-consistent manner where the decay is normalized by the measurable peaks for each given mix

time. The slope of the plot (Eq. S16) is directly related to the rate constants of both exchange processes (Eq. S1 and S2).

$$\int I_{\alpha,0} = \int I_{\alpha,t} + \int I_{OTf,t} \qquad \text{Eq. S14}$$

$$\frac{\int I_{\alpha,t}}{\int I_{\alpha,t} + \int I_{OTf,t}} = 1 - (k_{inter} + k_{intra}[^{-}OTf]) \times \tau_m \qquad \text{Eq. S15}$$

$$Slope = k_{inter} + k_{intra}[^{-}OTf] \qquad \text{Eq. S16}$$

The rates measured and determined by selective ^{19}F EXSY spectroscopy is, as described above, directly the slope of the normalised absolute integral of the α-triflate ($\int I_{\alpha,t}$) versus mixing time. Hence, equation S16 is in the main text referred to as $R_{\alpha\rightarrow OTf,EXSY}$ (Eq. S17).

$$R_{\alpha\rightarrow OTf,EXSY} = Slope = k_{inter} + k_{intra}[^{-}OTf] \qquad \text{Eq. S17}$$

Chemical Exchange Saturation Transfer NMR (CEST NMR) kinetic experiments: Saturation transfer experiments to derive the exchange constant from the *α*-triflate to dioxanium ion were conducted as reported by Serianni et. al.[1] The system is considered as in Scheme S1, and their corresponding formation rates are described in eq. S18 and S19.

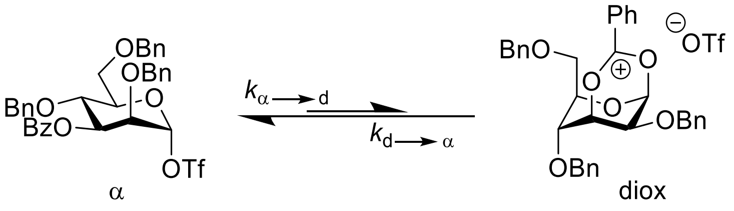

Scheme S1: Equilibrium considered for determining the reaction rate constant of dioxanium ion formation ($k_{\alpha\rightarrow d}$).

$$\frac{d[\alpha]}{dt} = R_{diox\rightarrow a} = k_{d\rightarrow\alpha}[diox][OTf] \qquad \text{Eq. S18}$$

$$\frac{d[diox]}{dt} = R_{a\rightarrow diox} = k_{\alpha\rightarrow d}[\alpha] \qquad \text{Eq. S19}$$

Equation S20 describes the modified Bloch equation that accounts for change in longitudinal magnetization (M_z) due to T_1 relaxation and chemical exchange as a function of time (t).

$$\frac{dM_z^{trif}(t)}{dt} = \frac{-\left(M_z^{trif}(t) - M_z^{trif}(0)\right)}{T_{1,trif}} - \frac{M_z^{trif}(t)}{\tau_{trif}} + \frac{M_z^{diox}(t)}{\tau_{diox}} \qquad \text{Eq. S20}$$

Herein: $T_{1,trif}$ is the spin-lattice relaxation time of the *α*-triflate resonance, τ_{trif} is the lifetime of the triflate, and τ_{diox} is the lifetime of the dioxanium ion. Due to saturation of the dioxanium resonance, the magnetization of the dioxanium (M_z^{diox}) becomes zero, and, as a result, M_z^{diox}/τ_{diox} also goes to zero. The resulting equation can then be integrated to yield equation S21.

$$M_z^{trif}(\tau) = M_z^{trif}(0) \times \left(\frac{\tau_{1,trif}}{\tau_{trif}} e^{\frac{-\tau}{\tau_{1,trif}}} + \frac{\tau_{1,trif}}{T_{1,trif}} \right) \quad \text{Eq. S21}$$

Here, $M_z^{trif}(\tau)$ and $M_z^{trif}(0)$ are the intensities of the α-triflate resonance after applying a saturation pulse for the duration of τ at the dioxanium ion resonance ($M_z^{trif}(\tau)$), and the intensity of the α-triflate resonance after applying a saturation pulse for the duration of 0 seconds at the dioxanium ion resonance ($M_z^{trif}(0)$). Subtracting the peak intensity of the α-triflate with 'infinitely long' saturation at the dioxanium ion ($M_z^{trif}(\infty)$) from $M_z^{trif}(\tau)$ gives the net saturation effect on the peak decay. Typically, $M_z^{trif}(\infty)$ is determined by saturating the dioxanium ion for five times the relaxation time of the carbonyl. Plotting the natural logarithm of the net peak decay resulting from saturation ($\ln(M_z^{trif}(\tau) - M_z^{trif}(\infty))$) against the saturation time (τ) gives a linear correlation where the slope is related to $\tau_{1,trif}$ according to equation S22.

$$slope = \frac{-1}{\tau_{1,trif}} \quad \text{Eq. S22}$$

The observed lifetime ($\frac{1}{\tau_{1,trif}}$, Equation S23) describes the relation between the α-triflate lifetime (τ_{trif}) and its T1. $T_{1,trif}$, can be determined from the acquired data according to equation S24.

$$\frac{1}{\tau_{1,trif}} = \frac{1}{\tau_{trif}} + \frac{1}{T_{1,trif}} \quad \text{Eq. S23}$$

$$T_{1,trif} = \frac{M_z^{trif}(0)}{M_z^{trif}(\infty)} \times \frac{1}{\tau_{1,trif}} \quad \text{Eq. S24}$$

Applying equations S22, S23, and S24 yields the lifetime of the α-triflate. As described by McConnell,[2] the lifetime is to be related to pseudo first-order rate constants. As a consequence, in the system as described by Scheme S1, the lifetimes of the α-triflate and dioxanium ion are given by equations S25 and S26. Hence, the rate constant for the formation of dioxanium ion from the α-triflate ($k_{\alpha \to d}$) is directly obtained from τ_{trif} (Eq. 25).

$$\frac{1}{\tau_{trif}} = k_{\alpha \to d} \quad \text{Eq. S25}$$

$$\frac{1}{\tau_{diox}} = k_{d \to \alpha}[^{-}OTf] \quad \text{Eq. S26}$$

Finally, in the main article $R_{\alpha \to d, CEST}$ is used to describe equation S26. Hence, $R_{\alpha \to d, CEST}$ is described according to equation S27.

$$R_{\alpha \to d,CEST} = \frac{1}{\tau_{trif}} = k_{\alpha \to d} \quad \text{Eq. S27}$$

Standard deviations: The rates determined by EXSY are directly the slope of the normalized α-triflate peak integral against mix time (Eq. S17). The corresponding graph is based on the measured data analysed with the least square regression method. Equations S28 is used to determine the error in the determined EXSY rate based on the measured input data.

$$s_x = \sqrt{\frac{\sum(X_i - \bar{X})^2}{n-1}} \quad \text{Eq. S28}$$

Rates determined by CEST are based on a linear relationship between the natural logarithm of the net saturation effect versus saturation time. The slope represents ${\tau_{1trif}}^{-1}$ and the standard deviation is calculated using equation S28. The relative error of ${\tau_{1trif}}^{-1}$ is determined according to equation S29 and the resulting relative error is applied to the determined rate (Eq. S30).

$$Relative\ error\ slope = \frac{e_{Slope}}{Slope} \quad \text{Eq. S29}$$

$$e_{R_{\alpha\rightarrow d,CEST}} = \frac{e_{Slope}}{Slope} \times R_{\alpha\rightarrow d,CEST} \quad \text{Eq. S30}$$

Experimental details

Instrumentation: Variable temperature NMR (VT NMR) experiments were conducted on a Bruker 300 MHz Avance III HD nanobay equipped with a BBFO probe and on the JEOL 500 ECZ-R spectrometer equipped with a ROYAL-HFX or ROYAL probe. Low temperature VT operations were achieved with the aid of LN_2 evaporator to supply the cold gas, which the probe heated to the desired temperature. The temperature for VT experiments was calibrated against a pure MeOH standard to accurately determine the probe temperature.

Chemical Exchange Saturation Transfer NMR (CEST NMR) spectroscopy: CEST NMR was utilized in ^{1}H, ^{13}C, and ^{19}F NMR spectroscopy. CEST NMR spectra were recorded by incrementing the saturation over a domain of interest. For ^{1}H the domain of interest is typically between 9 and 4 ppm; ^{13}C CEST was typically performed in a window between 190 and 160 ppm; and ^{19}F CEST was performed in a range of -72 to -80 ppm. Before each experiment, the 90-degree pulse was calibrated. A saturation field strength was chosen with respect to experimental duration, resolution, and signal intensity (eg. high resolution (small saturation field strengths) leads to weak signals and long experimental times, in contrast, low resolution (large saturation field strength) leads to stronger signals, faster experiments but low resolution). Typically, saturation field strengths were chosen to be 20 Hz (^{1}H CEST), 80 Hz (^{13}C CEST), and 30 Hz (^{19}F CEST). The saturation was achieved either by CW saturation (Bruker) or by pulsed saturation using laminar pulses (JEOL). Finally, typical saturation times were set to 2-3 seconds; the relaxation delay was set one second longer then the saturation time (3-4 seconds); number of scans were typically 2-4 per frequency; Two dummy scans (Bruker) or 2 (JEOL) were executed.

Selective ^{19}F Exchange NMR (EXSY NMR) spectroscopy: EXSY NMR was utilized in ^{19}F NMR spectroscopy. Before each experiment, the 90-degree pulse was calibrated and then the selective excitation offset was set to the resonance of interest. The selection pulse typically spanned either 0.1 or 0.2 ppm and was on-resonance with the glycosyl *α*-triflate ($\delta_F \approx -75.9$ ppm). The power levels of the excitation pulse were calculated against the actual 90-degree pulse and the selection window. Relaxation delays were typically set to $5xT_1$ of the triflates, and the number of scans were set to 8 with 2 dummy scans. The longest mix times were determined empirically so that they fit within the initial rate approximation (~10% conversion). For experiments where the shortest mix time was > 80 ms, a pulse sequence that contains a Z-gradient element to crush zero-quantum magnetization and clean up artifacts was used (Bruker: selnogp, JEOL: noesy_1d). However, frequently the shortest mix time was << 80 ms, therefore the versions of the above experiments without the Z-gradient element were utilized.

Sample preparation variable temperature NMR (VT NMR): Glycosyl sulfoxide donor (1.0 eq., typically 15 mg) and TTBP (2.5 eq.) were dissolved in dried DCM-d_2 (500 μL). Two spherical molecular sieves (4 or 5 Á) were added to the NMR tube and the tube was transferred to an analytical scale where internal standard (trimethyl(4-trifluoromethylphenyl)silane) was added. A stock solution of Tf_2O was prepared in DCM-d_2 such that upon addition of stock solution (50 μL), the desired amount Tf_2O (1.3 eq.) could be added. When the NMR sample and Tf_2O stock solution were ready, the NMR tube was cooled to -80 °C (dry ice/acetone bath) and to the cold tube was added the freshly prepared Tf_2O stock solution (50 μL). The solution generally becomes (light) yellow upon addition of Tf_2O, was shaken quickly (3x) and was carefully transferred to the NMR. In the probe, the temperature was heated to either -60 °C, -50 °C, -40 °C, or -30 °C for 1h depending on the stability of the *α*-triflate (typically at least 20 °C colder than the reported decomposition temperature, main text, Figure 3). The temperature was decreased to -80 °C and characterization/kinetic experiments were performed at variable temperatures.

Two experiments were performed under base-free conditions as previously reported.[3] Under these conditions: Glycosyl thioether donor (1.0 eq., typically 15 mg) and Ph_2SO (1.1 eq.) were weighted and dissolved in dried DCM-d_2 (500 μL). Two spherical molecular sieves (4 or 5 Á) were added to the NMR tube and the tube was transferred to an analytical scale where internal standard (trimethyl(4-trifluoromethylphenyl)silane) was added. A stock solution of Tf_2O was prepared in DCM-d_2 such that upon addition of stock solution (0.1 mL), the desired amount Tf_2O (5.0 eq.) could be added. The access Tf_2O was added to assure full consumption of Ph_2SO. When the NMR sample and Tf_2O stock solution were ready, the NMR tube was cooled to -80 °C (dry ice/acetone bath) and to the cold tube was added the freshly prepared Tf_2O stock solution (50 μL). The solution generally becomes (light) yellow upon addition of Tf_2O, was shaken quickly (3x) and was carefully transferred to the NMR. In the probe the temperature was heated to -60 ºC for non-strained donors and -30 ºC for strained donors. The samples were activated for 2h at their corresponding temperatures after which the temperature was cooled to -80 °C and (kinetic) experiments were performed at variable temperatures.

A 1.0 M solution of tetra butyl ammonium triflate (TBAT) was prepared in DCM-d_2. To the solution was added activated molecular sieves (4 Á) and the solution was stored under argon at -80 °C. This solution was removed from the -80 °C fridge 1h before the NMR experiment.

NMR experiments at various concentrations triflate anion were executed as described above with respect to sample preparation. After activation at the desired temperature, the probe was heated to the temperature where the sample displayed an exchange ($R_{\alpha \rightarrow OTf,\ EXSY}$) of about 0.2 s^{-1} to allow sufficient exchange at the lowest concentration and sufficient opportunity to increase as a consequence of the increased triflate concentration before falling out the window of EXSY NMR (see main text). After recording the triflate dissociation under standard conditions, the sample was removed from the probe, quickly stored in a dry ice/acetone bath (-80 °C) and the TBAT solution was added (20 μL). The sample was quickly shaken to homogenize the solution (3x)

and was carefully transferred to the probe. The sample was locked to DCM-d_2, tuned, and shimmed before performing NMR experiments. After finishing the EXSY experiment, the cycle was repeated for two more time (by adding 30 μL and 50 μL TBAT solution). In the data workup, the internal standard was used to accurately correct the concentration to volume and TBAT added.

EXSY Acquisition: ^{19}F EXSY rates were determined according to kinetics from the initial rates approximation. Typically 8-10 EXSY spectra were recorded with varying mixing times between the selection/excitation pulse and the acquisition. Typically a maximum conversion for the selected resonance (*α*-triflate) to the newly formed species ($^{-}$OTf) was between 10-15%. All acquired spectra were phased and baseline correction was applied. The peak areas from both the *α*-triflate resonance and triflate anion were normalized against the sum of the integrals for their corresponding spectrum. The normalized intensities were then plotted against the mixing time to yield a linear plot where the absolute slope of each graph represents the rate of *α*-triflate being converted to the $^{-}$OTf resonance.

CEST Profile Acquisition: CEST profiles were constructed using 1D ^{13}C spectra with saturation at variable positions. A spectrum with no saturation or with off-resonance saturation to both the major and minor observable signal is required as reference named $M_z(0)$ (typically a 1D spectrum with saturation at δ_C = 190 ppm; δ_H = 9.0 ppm; or δ_F = -72 ppm). The peak intensity for the *α*-triflate (^{13}C and ^{1}H CEST) and $^{-}$OTf (^{19}F) resonances were determined for all individual spectra after phasing and applying baseline correction. The peak intensity of every spectrum was divided by the peak intensity of the unaltered spectrum ($M_z(0)$) to obtain the relative peak intensity of the main observable species as function of saturation frequency. Plotting the relative intensity of the *α*-triflate as function of the saturation frequency gives the CEST spectrum.

CEST Kinetics Acquisition: Dioxanium ion formation rates ($R_{\alpha \rightarrow d,\ CEST}$, Scheme S1, page S4) were determined by using saturation-transfer. The saturation frequency offset was set to be on-resonance with the minor exchangeable species (e.g. the dioxanium C=O 13C resonance) as discovered by first obtaining a CEST profile. The saturation offset frequency was held constant while the saturation duration was varied. Deriving the exchange rate required a spectrum having no saturation ($M_z(0)$), a spectrum where the exchange and relaxation rates are in equilibrium ($M_z(inf)$), and a number of spectra where a decay of the main observable is visible as function of saturation time ($M_z(\tau)$). Typically the saturation times were set to 0 seconds, 0.1 seconds incrementing to 3 seconds, and 4 seconds. The resulting 1D ^{13}C spectra were phased and baseline correction was applied. The maximum peak intensity was determined for all individual 1D ^{13}C NMR spectra and was plotted against the saturation time to yield the kinetic build-up curves. The crude 1D ^{13}C peak intensities were converted as described above (page S4-5) to yield the exchange rate.

Applying the same strategy in ^{1}H spectroscopy by fixing the saturation transmitter to either the dioxanium ion resonance ($\delta_H \approx$ 6.5 ppm) or β-triflate resonance ($\delta_H \approx$ 5.6 ppm) allows to determine the normalized rate at which the *α*-triflate is converted to the

dioxanium ion ($R_{\alpha \rightarrow OTf, CEST}$) or rate at which the α-triflate becomes β-triflate ($R_{\alpha \rightarrow \beta, CEST}$) respectively.

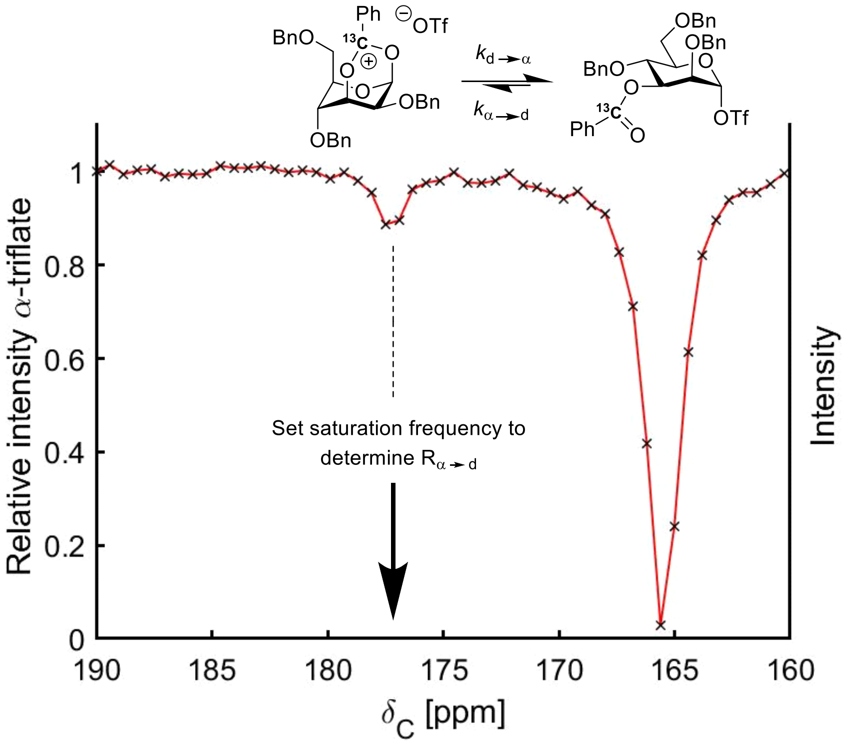

Figure S2: Rate of *α*-triflate becoming dioxanium ion is determined by setting the saturation frequency to the minimum of the (minor) dioxanium ion signal in ^{13}C CEST NMR and subsequently applying variable saturation times on-resonance.

General synthetic methods: ^{1}H, $^{13}C\{^{1}H\}$, COSY, ^{1}H-^{13}C HSQC (coupled and decoupled), and ^{1}H-^{13}C HMBC spectra were acquired on either a Bruker 500 MHz AVANCE III spectrometer, or JEOL 500 ECZ-R spectrometers. The Bruker 500 MHz Avance III spectrometer is equipped with a Prodigy BB cryoprobe. The JEOL 500 ECZ-R spectrometers were equipped with either a SuperCOOL broadband probe, ROYAL broadband probe, or ROYAL HFX broadband probe. Chemical shifts are reported in parts per million (ppm) relative to tetramethylsilane (TMS), or residual solvents as the internal standard. NMR data is presented as follows: chemical shift, multiplicity (s = singlet, d = doublet, t = triplet, dd = doublet of doublets, m = multiplet and/or multiple resonances), coupling constant (J) in hertz (Hz), integration. All NMR signals were assigned on the basis of 1H NMR, 13C NMR, COSY, HSQC, and TOCSY experiments.

Mass spectra were recorded on an JEOL AccuTOF CS JMST100CS mass spectrometer. Automatic flash column chromatography was performed using Biotage Isolera Spektra One, using SNAP cartridges (Biotage, 30− 100 μm, 60 Å), 10−50 g. TLC analysis was conducted on silica gel F254 (Merck KGaA) with detection by UV absorption (254 nm) where applicable; by spraying with 10% sulfuric acid in methanol followed by charring at ≈300 °C or by spraying with $KMnO_4$ stain consisting of (0.06M $KMnO_4$, 0.5M K_2CO_3 and 0.02M NaOH in water) after gently heating of the plate.

All reagents were commercially purchased unless stated differently. All reagents were used without purification apart for dry DCM, THF, and toluene which were freshly distilled. DCM-d_2 was dried by adding activated molecular sieves (4 Å) directly to the bottle as obtained from Deutero GmbH. Molecular sieves (3 Å, 4 Å, or 5 Å) were flame-activated under a vacuum prior to use. All dry reactions were carried out under an argon atmosphere using flame-dried flasks.

Chemical Synthesis:

General procedure I: S-oxidation of thioglycosides

To a solution of the thioglycoside (1.0 eq.) in DCM (0.1 M) at -78 °C was added *m*-CPBA (1.1 eq., 70% Wt) in DCM dropwise. The reaction mixture was stirred for 3h after which it was then quenched by the addition of sat. aq. $Na_2S_2O_3$ and diluted using DCM. The organic layer was washed with sat. aq. $NaHCO_3$ (2x) and brine, dried over $MgSO_4$ and concentrated *in vacuo*. The residue was purified by silica gel flash column chromatography (0-25 Et_2O/Toluene, v/v).

Phenyl 2,3,4,6-tetra-*O*-benzyl-1-thio-α-D-mannopyranoside

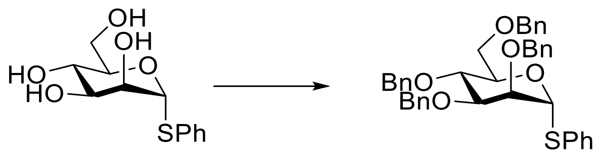

To a solution of phenyl 1-thio-α-D-mannopyranoside[4] (300 mg, 1 Eq, 1.10 mmol) in DMF (11.0 mL, 0.1 M) was added NaH (264 mg, 60% Wt, 6 Eq, 6.61 mmol) at 0 °C. After 15 minutes benzyl bromide (1.13 g, 786 µL, 6 Eq, 6.61 mmol) was added and the reaction mixture was stirred for 12h at rt. The reaction mixture was quenched with the addition of MeOH (5 mL) and H_2O (10 mL) and thereafter diluted with EtOAc (20 mL). Subquently, the water layer was extracted with EtOAc (3 x 20 mL). The combined organic layers were washed with $NaHCO_3$ (sat. aq., 10 mL), brine (10 mL) and dried $MgSO_4$ and concentrated *in vacuo*. The crude oil was then purified using silica gel flash column chromatography (0→20% EtOAc/*n*-heptane, v/v) to obtain phenyl 2,3,4,6-tetra-*O*-benzyl-1-thio-α-D-mannopyranoside (690 mg, 99%) as a colorless syrup.

TLC: R_f 0.72 (EtOAc/*n*-heptane, 1:1, v/v); **^{1}H NMR** (500 MHz, CHLOROFORM-*D*) δ 7.43 (dd, *J* = 6.5, 3.0 Hz, 2H), 7.37 – 7.17 (m, 23H), 5.60 (d, *J* = 2.1 Hz, 1H, H-1), 4.89 (d, *J* = 10.8 Hz, 1H), 4.72 (d, *J* = 12.4 Hz, 1H), 4.66 – 4.54 (m, 4H), 4.52 (d, *J* = 10.8 Hz, 1H), 4.47 (d, *J* = 11.9 Hz, 1H), 4.27 (ddd, *J* = 9.9, 5.2, 2.0 Hz, 1H, H-5), 4.06 (td, *J* = 9.6, 2.1 Hz, 1H, H-4), 4.00 – 3.96 (m, 1H, H-2), 3.85 (dd, *J* = 9.6, 3.0 Hz, 1H, H-3), 3.82 (d, *J* = 5.1 Hz, 1H, H-6), 3.73 (dd, *J* = 10.9, 2.1 Hz, 1H, H-6).**^{13}C NMR** (126 MHz, CHLOROFORM-*D*) δ 138.6, 138.5, 138.3, 138.1, 134.5 (all quarternary), 131.8, 129.1, 128.6, 128.5, 128.5, 128.4, 128.1, 128.1, 128.0, 127.9, 127.8, 127.8, 127.6, 127.5 (all aromatic), 85.9 (C-1), 80.3 (C-3), 76.4 (C-2), 75.3, 75.1 (C-4), 73.4, 72.9 (C-5), 72.2, 72.0, 69.3 (C-6).**HRMS** [M + Na]$^+$ calcd. for $C_{40}H_{40}O_5S$ 655.24941, found 655.24673.

phenyl 2,3,4,6-tetra-*O*-benzyl-1-thiosulfinyl-α-D-mannopyranoside

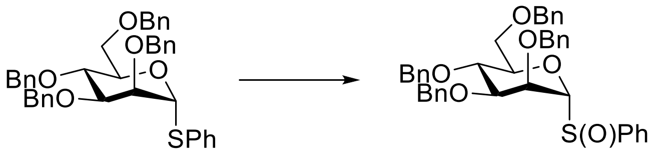

Via general S-oxidation procedure starting with phenyl 2,3,4,6-tetra-*O*-benzyl-1-thio-α-D-mannopyranoside (500 mg, 1 Eq, 790 µmol) to afford phenyl 2,3,4,6-tetra-*O*-benzyl-1-thiosulfinyl-α-D-mannopyranoside (415 mg, 81%) as a white powder.

TLC: R_f 0.45 (EtOAc/*n*-heptane, 1:1 v/v);**^{1}H NMR** (500 MHz, CHLOROFORM-*D*) δ 7.62 – 7.57 (m, 2H), 7.51 – 7.44 (m, 1H), 7.42 – 7.38 (m, 2H), 7.37 – 7.26 (m, 18H), 7.23 – 7.19 (m, 2H), 4.93 (d, *J* = 10.9 Hz, 1H), 4.65 (s, 2H), 4.59 – 4.57 (m, 3H, H-1), 4.56 (s, 1H), 4.53 (d, *J* = 10.9 Hz, 1H), 4.48 (s, 1H), 4.46 (t, *J* = 2.6 Hz, 1H, H-2), 4.21 (dd, *J* = 9.2, 3.3 Hz, 1H, H-3), 4.14 (ddd, *J* = 9.9, 5.8, 1.9 Hz, 1H, H-5), 4.01 (t, *J* = 9.5 Hz, 1H, H-4), 3.75 (dd, *J* = 10.8, 2.0 Hz, 1H, H-6), 3.68 (dd, *J* = 10.8, 5.8 Hz, 1H, H-6).**^{13}C NMR** (126 MHz, CHLOROFORM-*D*) δ 142.0, 138.3, 138.2, 138.2, 137.7 (all quaternary), 131.4, 129.3, 128.6, 128.5, 128.5, 128.2, 128.1, 128.0, 127.9, 127.9, 127.8, 124.6 (all aromatic), 96.1 (C-1), 79.7 (C-3), 77.9 (C-5), 75.3, 74.1 (C-4), 73.6, 72.7, 72.3, 71.8 (C-2), 69.6 (C-6). **HRMS** [M + Na]$^+$ calcd. for $C_{40}H_{40}O_6S$ 671.24433, found 671.24200.

Phenyl 2,4,6-tri-*O*-benzyl-1-thio-α-D-mannopyranoside

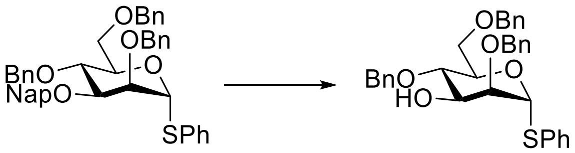

To a solution of phenyl 2,4,6-tri-*O*-benzyl- 3-*O*-(2-naphthylmethyl)-1-thio-α-D-mannopyranoside* (340 mg, 1 Eq, 498 µmol) in in DCM/H_2O (9:1 v/v, 5 mL) was added DDQ (170 mg, 1.5 Eq, 747 µmol) and the resulting mixture was stirred in the dark for 2.5 h. The reaction mixture was diluted using DCM (20 mL). The organic phase was washed twice using an aqueous mixture of ascorbic acid (0.7%), citric acid (1.5%) and NaOH (0.9%) (w/v, 2 x 15 mL). The organic layer was dried over $MgSO_4$, filtered and concentrated *in vacuo*. The residue was purified using silica gel flash column chromatography (0→25% EtOAc/pentane, v/v) to afford phenyl 2,4,6-tri-*O*-benzyl-1-thio-α-D-mannopyranoside (190 mg, 70%) as a waxy solid.

TLC: R_f 0.60 (EtOAc/*n*-heptane, 1:1 v/v);**^{1}H NMR** (499 MHz, CHLOROFORM-*D*) δ 7.52 – 7.47 (m, 2H), 7.38 – 7.22 (m, 18H), 5.69 (d, *J* = 1.3 Hz, 1H, H-1), 4.88 (d, *J* = 11.1 Hz, 1H), 4.77 (d, *J* = 11.6 Hz, 1H), 4.66 (d, *J* = 11.9 Hz, 1H), 4.56 (d, *J* = 11.0 Hz, 1H), 4.53 (d, *J* = 11.6 Hz, 1H), 4.50 (d, *J* = 12.0 Hz, 1H), 4.30 (ddd, *J* = 9.8, 4.9, 1.9 Hz, 1H, H-5), 4.03 – 4.01 (m, 1H, H-2), 4.01 – 3.97 (m, 1H, H-3), 3.85 (dd, *J* = 11.0, 4.9 Hz, 1H, H-6), 3.86 – 3.76 (m, 1H, H-4), 3.75 (dd, *J* = 10.9, 2.0 Hz, 1H, H-6), 2.42 (d, *J* = 9.2 Hz, 1H, OH-3). **^{13}C NMR** (126 MHz, CHLOROFORM-*D*) δ 138.5, 138.3, 137.5, 134.2 (all quaternary), 131.8, 129.1, 128.7, 128.5, 128.4, 128.2, 128.1, 128.0, 127.8, 127.8, 127.6, 127.6 (all aromatic), 85.1 (C-1), 79.8 (C-2), 76.9 (C-4), 75.0, 73.4, 72.4 (C-3), 72.2, 72.1 (C-5), 69.2 (C-6).**HRMS** $[M + Na]^+$ calcd. for $C_{33}H_{34}O_5S$ 565.20246, found 565.19989.

*Prepared via the same route as reported by Boltje and co-workers[3] using benzyl bromide instead of methyl iodide to benzylated instead of methylation.

Phenyl 3-*O*-benzoyl-2,4,6-tri-*O*-benzyl-1-thio-α-D-mannopyranoside

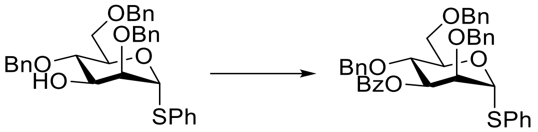

To a solution of phenyl 2,4,6-tri-*O*-benzyl-1-thio-α-D-mannopyranoside (50 mg, 1 Eq, 92 µmol) in pyridine (0.92 mL, 0.1 M), benzoyl chloride (16 mg, 13 µL, 1.25 Eq, 0.12 mmol) was added at 0 °C, and the mixture was stirred for 12h at rt. Upon completion the solvent was removed *in vacuo* after which the residue was redissolved in DCM (10 mL). The organic layer was washed with $NaHCO_3$ (2 mL) and brine (10 mL). The solution was dried over Na_2SO_4 and concentrated to get the benzoyl derivative as a solid, which was purified using silica gel flash column chromatography (0→15% EtOAc/pentane, v/v) to obtain phenyl 3-*O*-benzoyl-2,4,6-tri-*O*-benzyl-1-thio-α-D-mannopyranoside (55 mg, 92%) as a waxy solid.

TLC: R_f 0.60 (EtOAc/*n*-heptane, 1:1, v/v); **^{1}H NMR** (500 MHz, CHLOROFORM-*D*) δ 8.12 – 8.02 (m, 2H), 7.65 – 7.55 (m, 1H), 7.55 – 7.48 (m, 2H), 7.46 (t, *J* = 7.8 Hz, 3H), 7.39 – 7.22 (m, 7H), 7.22 – 7.05 (m, 10H), 5.66 (d, *J* = 1.9 Hz, 1H, H-1), 5.48 (dd, *J* = 9.3, 3.2 Hz, 1H, H-3), 4.75 – 4.66 (m, 3H), 4.58 – 4.48 (m, 3H),4.41 (ddd, *J* = 9.8, 4.6, 1.8 Hz, 1H, H-5), 4.30 (t, *J* = 9.5 Hz, 1H, H-4), 4.25 (dd, *J* = 3.3, 1.9 Hz, 1H, H-2), 3.89 (dd, *J* = 11.0, 4.6 Hz, 1H, H-6), 3.75 (dd, *J* = 11.0, 1.9 Hz, 1H, H-6).**^{13}C NMR** (126 MHz, CHLOROFORM-*D*) δ 165.8 (carbonyl), 138.4, 138.0, 137.7, 134.4 (all quaternary), 133.3, 131.9, 130.0 (quarternary), 129.9, 129.1, 128.6, 128.4, 128.1, 128.0, 127.9, 127.9, 127.8, 127.7, 127.6 (all aromatic), 85.7 (C-1), 77.4 (C-2), 75.0, 74.7 (C-3), 73.7 (C-4), 73.6, 72.7 (C-5), 72.5, 69.1 (C-6).**HRMS** [M + Na]$^+$ calcd. for $C_{40}H_{38}O_6S$ 669.22868, found 669.22641.

phenyl 3-*O*-benzoyl-2,4,6-tri-*O*-benzyl-1-thiosulfinyl-α-D-mannopyranoside

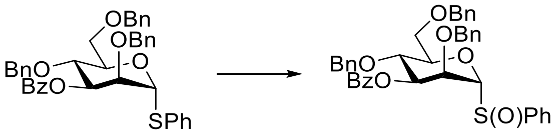

Via general S-oxidation procedure starting with phenyl 3-*O*-benzoyl-2,4,6-tri-*O*-benzyl-1-thio-α-D-mannopyranoside (30 mg, 1 Eq, 46 µmol) to afford phenyl 3-*O*-benzoyl-2,4,6-tri-*O*-benzyl-1-thiosulfinyl-α-D-mannopyranoside (21 mg, 68%) as an off-white powder.

TLC: R_f 0.44 (EtOAc/*n*-heptane, 1:1 v/v);**^{1}H NMR** (499 MHz, CHLOROFORM-*D*) δ 8.02 (dd, *J* = 8.3, 1.4 Hz, 2H), 7.72 – 7.64 (m, 2H), 7.60 – 7.52 (m, 1H), 7.50 – 7.36 (m, 5H), 7.36 – 7.01 (m, 16H), 5.80 (dd, *J* = 8.3, 3.4 Hz, 1H, H-3), 4.73 (d, *J* = 11.1 Hz, 1H), 4.65 (d, *J* = 2.7 Hz, 1H, H-1), 4.62 – 4.56 (m, 2H, H-2), 4.55 – 4.49 (m, 2H), 4.46 (d, *J* = 11.9 Hz, 1H), 4.39 (d, *J* = 11.8 Hz, 1H), 4.32 – 4.21 (m, 1H, H-5), 4.16 (dd, *J* = 9.6, 8.3 Hz, 1H, H-4), 3.70 (dd, *J* = 3.5, 2.1 Hz, 2H, H-6).**^{13}C NMR** (126 MHz, CHLOROFORM-*D*) δ 165.5 (carbonyl), 141.6, 138.1, 137.7, 137.2 (all quaternary), 133.3, 131.4, 130.0, 130.0, 129.4, 128.6, 128.5, 128.5, 128.4, 128.2, 128.1, 128.0, 128.0, 127.9, 127.8, 124.8 (all aromatic), 96.2 (C-1), 77.5 (C-5), 74.6, 73.7, 73.3 (C-4), 73.3 (C-3), 72.6, 72.2 (C-2), 69.3 (C-6). **HRMS** [M + Na]$^+$ calcd. for $C_{40}H_{38}O_7S$ 685.22359, found 685.22039.

phenyl 3-*O*-(benzoyl-α- ^{13}C)-2,4,6-tri-*O*-benzyl-1-thio-α-D-mannopyranoside

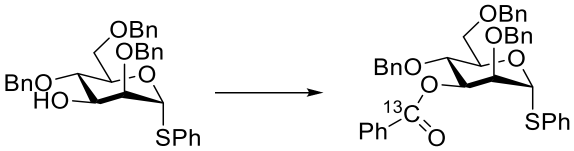

To a solution of benzoic acid-α-^{13}C (71.5 mg, 2.1 Eq, 580 µmol) in dry ACN (2.76 mL, 0.1 M), was added CDI (44.8 mg, 1 Eq, 276 µmol) under N_2. The reaction mixture was stirred at 70 °C for 2h before phenyl 2,4,6-tri-*O*-benzyl-1-thio-α-D-mannopyranoside (150 mg, 1 Eq, 276 µmol) ACN (0.5 mL) was added dropwise followed by DBU (88.4 mg, 87.5 µL, 2.1 Eq, 580 µmol). The reaction mixture was stirred at 60 °C overnight, cooled and poured into $NaHCO_3$ (sat. aq., 3 mL). The water layer was extracted with DCM (3 x 15 mL). The combined organic phases were washed with brine (5 mL), dried over $MgSO_4$ and concentrated *in vacuo*. The residue was purified using silica gel flash

column chromatography (0→20% EtOAc/*n*-heptane, v/v) to afford phenyl 3-*O*-(benzoyl-α- ^{13}C)-2,4,6-tri-*O*-benzyl-1-thio-α-D-mannopyranoside as a clear oil (105 mg, 59%).

TLC: R_f 0.60 (EtOAc/*n*-heptane, 1:1 v/v);**^{1}H NMR** (500 MHz, CHLOROFORM-*D*) δ 8.11 – 8.01 (m, 2H), 7.61 (t, *J* = 7.5 Hz, 1H), 7.55 – 7.52 (m, 2H), 7.47 (t, *J* = 7.6 Hz, 2H), 7.39 – 7.36 (m, 2H), 7.35 – 7.24 (m, 8H), 7.21 – 7.09 (m, 8H), 5.68 (d, *J* = 1.9 Hz, 1H), 5.49 (dt, *J* = 9.5, 3.2 Hz, 1H), 4.80 – 4.65 (m, 3H), 4.58 – 4.49 (m, 3H), 4.43 (ddd, *J* = 9.8, 4.6, 1.8 Hz, 1H, H-5), 4.33 (t, *J* = 9.6 Hz, 1H, H-4), 4.27 (dd, *J* = 3.3, 1.9 Hz, 1H, H-2), 3.91 (dd, *J* = 11.0, 4.5 Hz, 1H, H-6), 3.77 (dd, *J* = 10.9, 1.9 Hz, 1H, H-6).**^{13}C NMR** (126 MHz, CHLOROFORM-*D*) δ 165.7 (carbonyl), 138.3, 137.9, 137.6, 134.4 (all quaternary), 133.3, 131.7, 129.9, 129.9, 129.1, 128.6, 128.6, 128.4, 128.4, 128.0, 128.0, 127.9, 127.8, 127.7, 127.5 (all aromatic), 85.6 (C-1), 77.3 (C-2), 75.0, 74.6 (C-3), 73.6 (C-4), 73.5, 72.6 (C-5), 72.4, 69.0 (C-6).**HRMS** [M + Na]$^+$ calcd. for $C_{39}{}^{13}CH_{38}O_6S$ 670.23203, found 670.23053.

Phenyl 3-*O*-(benzoyl-α- ^{13}C)-2,4,6-tri-*O*-benzyl-1-thiosulfinyl-α-D-mannopyranoside

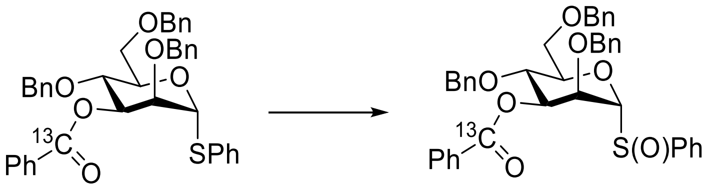

Via general S-oxidation procedure starting with phenyl 3-*O*-(benzoyl-α- ^{13}C)-2,4,6-tri-*O*-benzyl-1-thio-α-D-mannopyranoside (70 mg, 1 Eq, 0.11 mmol) to afford phenyl 3-O-(benzoyl-α- ^{13}C)-2,4,6-tri-O-benzyl-1-thiosulfinyl-α-D-mannopyranoside as a white powder 55 mg, 77%).

TLC: R_f 0.35 (EtOAc/*n*-heptane, 1:1 v/v);**^{1}H NMR** (500 MHz, CHLOROFORM-*D*) δ 8.03 (ddd, *J* = 8.4, 4.1, 1.4 Hz, 2H), 7.73 – 7.62 (m, 2H), 7.58 (td, *J* = 7.3, 1.4 Hz, 1H), 7.51 – 7.40 (m, 2H), 7.32 (d, *J* = 4.9 Hz, 5H), 7.23 – 7.04 (m, 10H), 5.80 (dt, *J* = 8.2, 3.3 Hz, 1H, H-3), 4.73 (d, *J* = 11.1 Hz, 1H), 4.64 (d, *J* = 2.6 Hz, 1H, H-1), 4.62 – 4.57 (m, 2H, H-2), 4.55 – 4.49 (m, 2H), 4.46 (d, *J* = 11.8 Hz, 1H), 4.39 (d, *J* = 11.9 Hz, 1H), 4.27 (ddd, *J* = 9.6, 4.4, 2.6 Hz, 1H, H-5), 4.17 (dd, *J* = 9.6, 8.3 Hz, 1H, H-4), 3.74 – 3.58 (m, 2H, H-6).**^{13}C NMR** (126 MHz, CHLOROFORM-*D*) δ 165.5 (carbonyl), 141.6, 138.0, 137.7, 137.2 (all quaternary), 133.3, 131.4, 130.0, 130.0, 129.3, 128.6, 128.5, 128.5, 128.5, 128.4, 128.1, 128.1, 128.0, 127.9, 127.9, 127.8, 124.8 (all aromatic), 96.2 (C-1), 74.6, 73.6, 73.3 (C-3), 72.6, 72.2 (C-2), 69.3 (C-6). **HRMS** [M + Na]$^+$ calcd. for $C_{39}{}^{13}CH_{38}O_7S$ 686.22695, found 686.22455.

4-methoxy-[α-^{13}C]-benzoic acid

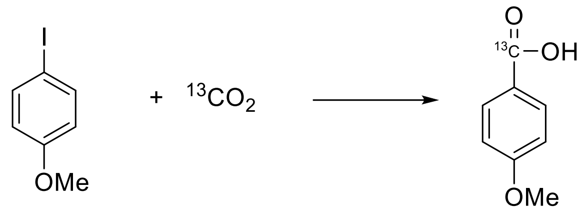

p-iodoanisole (1.0 gram, 4.3 mmol) was dissolved in dry THF (10 mL). The solution was cooled to -78 °C and a solution of s-BuLi (1.4 M in cyclohexane, 6.6 mL, 8.6 mmol, 2.0 eq.) was added. The solution was stirred for 2h after which a cylinder of $^{13}CO_2$ was connected to the reaction (p = 1.5 atm., Figure S3) and the needle reached the bottom of the flask to ensure bubbling of $^{13}CO_2$ through the reaction solution upon opening the valve. The cylinder was opened for 5x 5 seconds and product formation was immediately observed by the formation of white solids. The reaction was heated to rt, diluted with THF (10 mL), acidified with a few droplets of aqueous 1.0 M HCl, and further diluted with Et_2O (25 mL). The organic phase was washed with water (25 mL) followed by brine (25 mL). The combined aqueous phases were extracted with Et_2O (2x 50 mL) and the combined organic phases were dried over MgSO4, filtrated, and the solvent was evaporated *in vacuo*. The product purified by silica gel flash column chromatography (20→40% EtOAc/*n*-heptane, v/v) and was obtained as white solid (560 mg, 86%).

TLC: R_f = 0.42 (EtOAc/*n*-heptane, 3/2 v/v); **^{1}H NMR** (500 MHz, DMSO-d_6): δ_H 7.95 – 7.86 (m, 2H), 7.05 – 7.00 (m, 2H), 3.83 (s, 3H); **^{13}C NMR** (126 MHz, DMSO-d_6): δ_C 167.0 (quaternary), 162.8 (quaternary), 131.3 (d, *J* = 3.1 Hz, aromatic), 122.9 (d, *J* = 73.9 Hz, quaternary), 113.8 (d, *J* = 4.9 Hz, aromatic), 55.43.

NMR data in good agreement to literature.[5]

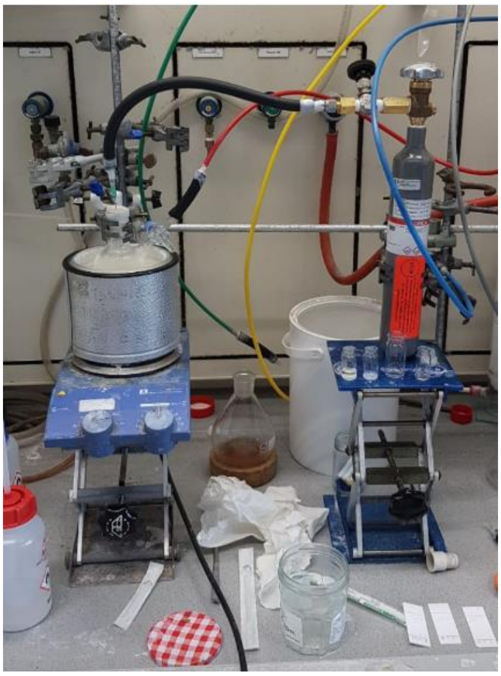

Figure S3: Reaction setup used to introduce $^{13}CO_2$ to the reaction mixture at -78 °C

Phenyl 3-*O*-(4-methoxybenzoyl-α- ^{13}C)-2,4,6-tri-*O*-benzyl-1-thio-α-D-mannopyranoside

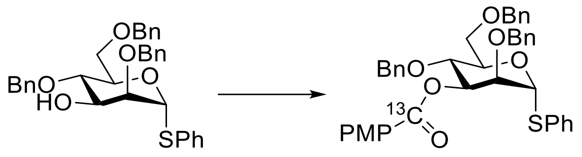

To phenyl 2,4,6-tri-*O*-benzyl-1-thio-α-D-mannopyranoside (70 mg, 1 Eq, 0.13 mmol) dissolved in DCM (1.3 mL, 0.1 M) was added 4-methoxybenzoic acid-α- ^{13}C (22 mg, 1.1 Eq, 0.14 mmol). The mixture was activated by the addition of DIC (24 mg, 30 µL, 1.5 Eq, 0.19 mmol) and DMAP (1.6 mg, 0.1 Eq, 13 µmol) whereafter it was stirred for 60h at rt. The reaction mixture was then filtered and concentrated *in vacuo*. The crude material was purified using column purified using silica gel flash column chromatography (0→20% EtOAc/*n*-heptane, v/v) to afford phenyl 3-*O*-(4-methoxybenzoyl-α-^{13}C)-2,4,6-tri-*O*-benzyl-1-thio-α-D-mannopyranoside (59 mg, 68%) as a clear oil.

TLC: R_f 0.84 (EtOAc/*n*-heptane, 1:1, v/v); **^{1}H NMR** (500 MHz, CHLOROFORM-*D*) δ 8.05 – 7.98 (m, 2H), 7.58 – 7.44 (m, 2H), 7.39 – 7.07 (m, 18H), 6.98 – 6.90 (m, 2H), 5.65 (d, *J* = 1.9 Hz, 1H, H-1), 5.46 (dt, *J* = 9.3, 3.2 Hz, 1H, H-3), 4.73 (d, *J* = 10.9 Hz, 1H), 4.70 (d, *J* = 12.0 Hz, 1H), 4.68 (d, *J* = 12.2 Hz, 1H), 4.54 (d, *J* = 10.9 Hz, 1H), 4.54 (d, *J* = 12.2 Hz, 1H), 4.51 (d, *J* = 12.0 Hz, 1H), 4.41 (ddd, *J* = 9.8, 4.6, 1.9 Hz, 1H, H-5), 4.29 (t, *J* = 9.6 Hz, 1H, H-4), 4.24 (dd, *J* = 3.3, 1.9 Hz, 1H, H-2), 3.89 (dd, *J* = 11.0, 4.5 Hz, 1H, H-6), 3.80 (s, 3H), 3.74 (dd, *J* = 10.9, 1.9 Hz, 1H, H-6).**^{13}C NMR** (126 MHz, CHLOROFORM-*D*) δ 165.5 (carbonyl), 163.7, 138.4, 138.0, 137.7, 134.5 (all quaternary), 132.0, 132.0, 131.8, 129.1, 128.4, 128.1, 127.9, 127.8, 127.7, 127.5, 122.4 (d, *J* = 80.0 Hz, quaternary), 113.9 (all aromatic), 85.8 (C-1), 77.5 (C-2), 75.0, 74.4 (C-3), 73.8 (C-4), 73.6, 72.7 (C-5), 72.5, 69.1 (C-6), 55.6.**HRMS** $[M + Na]^+$ calcd. for $C_{40}{}^{13}CH_{40}O_7S$ 700.24260, found 700.23808.

Phenyl 2,3-di-*O*-benzyl-4,6-*O*-benzylidene-1-thio-α-D-mannopyranoside

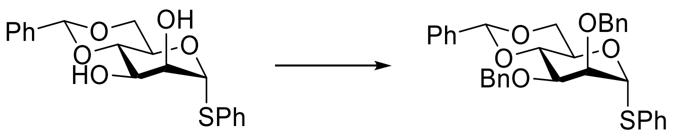

To a solution of phenyl 4,6-*O*-benzylidene-1-thio-α-D-mannopyranoside[4] (150 mg, 1 Eq, 416 µmol) in DMF (4.16 mL, 0.1 M) was added NaH (0.10 g, 60% Wt, 6 Eq, 2.50 mmol) at 0 °C. After 15 minutes benzyl bromide (178 mg, 124 µL, 2.5 Eq, 1.04 mmol) was added and the reaction mixture was stirred for 12h at rt. The reaction mixture was quenched with the addition of MeOH (5 mL) and H_2O (10 mL) and thereafter diluted with EtOAc (10 mL). Subquently, the water layer was extracted with EtOAc (3 x 10 mL). The combined organic layers were washed with $NaHCO_3$ (sat. aq., 5 mL), brine (5 mL) and dried $MgSO_4$ and concentrated *in vacuo*. The crude oil was then purified using silica gel flash column chromatography (0→20% EtOAc/*n*-heptane, v/v) to obtain phenyl 2,3-di-*O*-benzyl-4,6-*O*-benzylidene-1-thio-α-D-mannopyranoside (220 mg, 98%) as a white powder.

TLC: R_f 0.34 (EtOAc/*n*-heptane, 1:1 v/v);**^{1}H NMR** (499 MHz, CHLOROFORM-*D*) δ 7.53 (dd, *J* = 7.9, 1.8 Hz, 2H), 7.41 – 7.28 (m, 18H), 5.66 (s, 1H), 5.52 (d, *J* = 1.4 Hz, 1H, H-1), 4.84 (d, *J* = 12.2 Hz, 1H), 4.74 (s, 2H), 4.67 (d, *J* = 12.2 Hz, 1H), 4.36 – 4.32 (m, 1H, H-4), 4.31 – 4.27 (m, 1H, H-5), 4.23 (dd, *J* = 10.2, 4.4 Hz, 1H, H-6), 4.06 (dd, *J* = 3.2, 1.5 Hz, 1H, H-2), 3.98 (dd, *J* = 9.4, 3.2 Hz, 1H, H-3), 3.90 (t, *J* = 9.9 Hz, 1H, H-6).**^{13}C NMR** (126 MHz, CHLOROFORM-*D*) δ 142.4, 141.8, 141.7, 137.8 (all quaternary), 135.7, 133.2, 132.9, 132.5, 132.4, 132.2, 132.2, 131.9, 131.7, 131.7, 130.2, 105.6 (all aromatic), 91.2 (C-1), 83.2 (C-4), 82.2 (C-2), 80.3 (C-3), 77.2, 77.1, 72.6 (C-6), 69.5 (C-5). **HRMS** $[M + Na]^+$ calcd. for $C_{33}H_{32}O_5S$ 563.18681, found 563.18544.

Phenyl 2,3-di-*O*-benzyl-4,6-*O*-benzylidene-1-thiosulfinyl-α-D-mannopyranoside

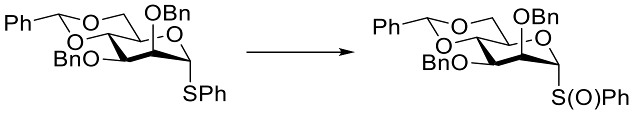

Via general *S*-oxidation procedure starting with phenyl 2,3-di-*O*-benzyl-4,6-*O*-benzylidene-1-thio-α-D-mannopyranoside (400 mg, 1 Eq, 740 µmol) to afford phenyl 2,3-di-*O*-benzyl-4,6-*O*-benzylidene-1-thiosulfinyl-α-D-mannopyranoside (300 mg, 73%) as a white powder.

TLC: R_f 0.34 (EtOAc/*n*-heptane, 1:1 v/v); **^{1}H NMR** (500 MHz, CHLOROFORM-*D*) δ 7.55 – 7.48 (m, 7H), 7.41 – 7.19 (m, 13H), 5.63 (s, 1H), 4.82 (d, *J* = 12.0 Hz, 1H), 4.67 (d, *J* = 12.0 Hz, 1H), 4.62 – 4.54 (m, 2H), 4.50 (d, *J* = 1.4 Hz, 1H, H-1), 4.39 (dd, *J* = 3.2, 1.4 Hz, 1H, H-2), 4.34 – 4.30 (m, 1H, H-4), 4.28 (dd, *J* = 10.0, 3.2 Hz, 1H, H-3), 4.21 (dd, *J* = 10.3, 4.9 Hz, 1H, H-6), 4.10 (td, *J* = 9.4, 4.8 Hz, 1H, H-5), 3.75 (t, *J* = 10.1 Hz, 1H, H-6).**^{13}C NMR** (126 MHz, CHLOROFORM-*D*) δ 141.7, 138.4, 137.5, 137.4 (all quaternary), 131.8, 129.6, 129.1, 128.5, 128.5, 128.4, 128.0, 127.9, 127.8, 126.2, 124.5 (all aromatic), 101.8, 97.8 (C-1), 78.2 (C-4), 76.4 (C-3), 73.7, 73.4, 73.0 (C-2), 70.2 (C-5), 68.3 (C-6). **HRMS** $[M + Na]^+$ calcd. for $C_{33}H_{32}O_6S$ 579.18173, found 579.18386.

Phenyl 2-*O*-benzyl-4,6-*O*-benzylidene-1-thio-α-D-mannopyranoside

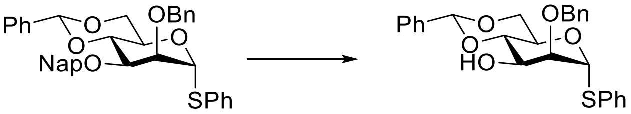

To a solution of phenyl 2-*O*-benzyl-4,6-*O*-benzylidene-3-*O*-(2-naphthylmethyl)-1-thio-α-D-mannopyranoside[6] (200 mg, 1 Eq, 339 µmol) in DCM/H_2O (9:1 v/v, 4 mL) was added DDQ (115 mg, 1.5 Eq, 508 µmol)) and the resulting mixture was stirred in the dark for 2.5 h. The reaction mixture was diluted using DCM (15 mL). The organic phase was washed twice using an aqueous mixture of ascorbic acid (0.7%), citric acid (1.5%) and NaOH (0.9%) (w/v, 2 x 10 mL). The organic layer was dried over $MgSO_4$, filtered and concentrated *in vacuo*. The residue was purified using silica gel flash column chromatography (0→20% Et_2O/toluene, v/v) to afford phenyl 2-*O*-benzyl-4,6-*O*-benzylidene-1-thio-α-D-mannopyranoside (130 mg, 85%) as a translucent crystalline solid.

TLC: R_f 0.51 (EtOAc/*n*-heptane, 1:1 v/v); **^{1}H NMR** (499 MHz, CHLOROFORM-*D*) δ 7.56 – 7.48 (m, 2H), 7.46 – 7.40 (m, 2H), 7.40 – 7.29 (m, 11H), 5.59 (s, 1H), 5.58 (s, 1H, H-1), 4.76 (d, *J* = 11.6 Hz, 1H), 4.65 (d, *J* = 11.6 Hz, 1H), 4.31 (td, *J* = 9.8, 4.9 Hz, 1H, H-5), 4.23 (dd, *J* = 10.3, 4.9 Hz, 1H, H-6), 4.15 – 4.12 (m, 1H, H-3), 4.12 – 4.10 (m, 1H, H-2), 4.00 (t, *J* = 9.5 Hz, 1H, H-4), 3.84 (t, *J* = 10.2 Hz, 1H, H-6), 2.44 (d, *J* = 7.9 Hz, 1H, OH-3). **^{13}C NMR** (126 MHz, CHLOROFORM-*D*) δ 137.4, 133.8 (all quaternary), 132.0, 129.3, 129.3, 128.8, 128.4, 128.4, 128.2, 127.9, 126.5 (all aromatic), 102.3, 86.4 (C-1), 80.2 (C-2), 79.7 (C-4), 73.3, 69.2 (C-3), 68.6 (C-6), 64.9 (C-5). **HRMS** [M + Na]$^+$ calcd. for $C_{26}H_{26}O_5S$ 473.13986, found 473.13908.

Phenyl 3-*O*-benzoyl-2-*O*-benzyl-4,6-*O*-benzylidene-1-thio-α-D-mannopyranoside

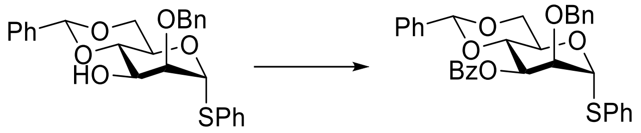

To a solution of phenyl 2-*O*-benzyl-4,6-*O*-benzylidene-1-thio-α-D-mannopyranoside (125 mg, 1 Eq, 277 µmol) in pyridine (2.77 mL, 0.1 M) benzoyl chloride (48.7 mg, 40.3 µL, 1.25 Eq, 347 µmol) was added at 0 °C. The resulting mixture was stirred at rt overnight. Water (5 mL) was added and extracted with DCM (3 x 10 mL). The combined organic layers were washed with $NaHCO_3$ (sat. aq., 5 mL), water (5 mL) and brine (5 mL). The solution was dried over $MgSO_4$ and concentrated *in vacuo* to get a crude oil which was purified using silica gel flash column chromatography (0→20% EtOAc/*n*-heptane, v/v) to obtain phenyl 3-*O*-benzoyl-2-*O*-benzyl-4,6-*O*-benzylidene-1-thio-α-D-mannopyranoside (110 mg, 72%) as a milky oil.

TLC: R_f 0.61 (EtOAc/*n*-heptane, 1:1 v/v); **^{1}H NMR** (500 MHz, CHLOROFORM-*D*) δ 8.12 – 8.03 (m, 2H), 7.63 – 7.55 (m, 1H), 7.51 – 7.42 (m, 6H), 7.37 – 7.29 (m, 5H), 7.27 – 7.23 (m, 2H), 7.22 – 7.15 (m, 3H), 5.65 (s, 1H), 5.59 (d, *J* = 1.5 Hz, 1H, H-1), 5.56 (dd, *J* = 9.9, 3.4 Hz, 1H, H-3), 4.67 (d, *J* = 11.9 Hz, 1H), 4.56 (d, *J* = 11.9 Hz, 1H), 4.52 – 4.47 (m, 1H, H-5), 4.47 – 4.42 (m, 1H, H-4), 4.38 (dd, *J* = 3.4, 1.4 Hz, 1H, H-2), 4.28 (dd, *J* = 10.4, 4.6 Hz, 1H, H-6), 3.94 (t, *J* = 10.0 Hz, 1H, H-6).**^{13}C NMR** (126 MHz, CHLOROFORM-*D*) δ 165.9 (carbonyl), 137.4, 137.3, 133.8 (all quaternary), 133.3, 132.0, 130.0, 130.0 (quaternary), 129.3, 129.2, 128.6, 128.5, 128.4, 128.1, 128.1, 127.9, 126.3 (all aromatic), 101.9, 86.7 (C-1), 77.9 (C-2), 76.5 (C-4), 73.3, 71.3 (C-3), 68.7 (C-6), 65.5 (C-5).**HRMS** [M + Na]$^+$ calcd. for $C_{33}H_{30}O_6S$ 577.16608, found 577.16359.

Phenyl 3-*O*-benzoyl-2-*O*-benzyl-4,6-*O*-benzylidene-1-thiosulfinyl-α-D-manno-pyranoside

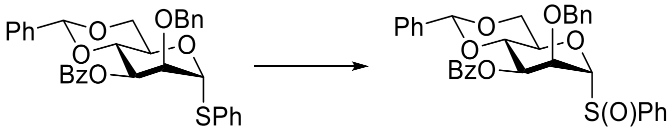

Via general S-oxidation procedure starting with phenyl 3-*O*-benzoyl-2-*O*-benzyl-4,6-*O*-benzylidene-1-thio-α-D-mannopyranoside (90 mg, 1 Eq, 0.16 mmol) to afford phenyl 3-*O*-benzoyl-2-*O*-benzyl-4,6-*O*-benzylidene-1-thiosulfinyl-α-D-mannopyranoside (61 mg, 66%) as a white solid.

TLC: R_f 0.33 (EtOAc/*n*-heptane, 1:1 v/v);**^{1}H NMR** (500 MHz, CHLOROFORM-*D*) δ 8.05 (dd, *J* = 8.3, 1.4 Hz, 2H), 7.74 – 7.67 (m, 2H), 7.58 (dt, *J* = 4.9, 2.5 Hz, 4H), 7.47 – 7.41 (m, 4H), 7.33 (dd, *J* = 5.1, 2.0 Hz, 3H), 7.20 – 7.14 (m, 1H), 7.13 – 7.05 (m, 4H), 5.87 (dd, *J* = 10.4, 3.8 Hz, 1H, H-3), 5.62 (s, 1H), 4.64 (dd, *J* = 3.8, 1.3 Hz, 1H, H-2), 4.55 (d, *J* = 1.3 Hz, 1H, H-1), 4.49 (d, *J* = 11.8 Hz, 1H), 4.43 (dd, *J* = 10.5, 9.2 Hz, 1H, H-4), 4.35 (d, *J* = 11.7 Hz, 1H), 4.34 – 4.29 (m, 1H, H-5), 4.27 (t, *J* = 5.1 Hz, 1H, H-6), 3.79 (t, *J* = 9.9 Hz, 1H, H-6).**^{13}C NMR** (126 MHz, CHLOROFORM-*D*) δ 165.6 (carbonyl), 141.4, 137.1, 136.8 (all quaternary), 133.3, 131.9, 130.1, 129.9 (quaternary), 129.7, 129.2, 128.5, 128.5, 128.4, 128.2, 128.1 (all aromatic), 102.0, 97.5 (C-1), 75.7 (C-4), 73.4, 72.7 (C-2), 71.1 (C-3), 70.1 (C-5), 68.4 (C-6).**HRMS** $[M + Na]^+$ calcd. for $C_{33}H_{30}O_7S$ 593.16099, found 593.15889.

Phenyl 3-*O*-(benzoyl-α- ^{13}C)-2-*O*-benzyl-4,6-*O*-benzylidene-1-thio-α-D-manno-pyranoside

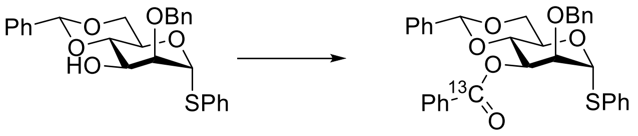

To phenyl 2-*O*-benzyl-4,6-*O*-benzylidene-1-thio-α-D-mannopyranoside (120 mg, 1 Eq, 266 µmol) dissolved in DCM (2.66 mL, 0.1 M) was added benzoic acid-α-^{13}C (36.1 mg, 28.4 µL, 1.1 Eq, 293 µmol). The mixture was activated by the addition of DIC (50.4 mg, 62.6 µL, 1.5 Eq, 400 µmol) and DMAP (3.25 mg, 0.1 Eq, 26.6 µmol) whereafter it was stirred overnight at rt. The reaction mixture was then filtered and concentrated *in vacuo*. The crude material was purified using silica gel flash column chromatography (0→20% EtOAc/*n*-heptane, v/v) to afford phenyl 3-*O*-(benzoyl-α- ^{13}C)-2-*O*-benzyl-4,6-*O*-benzylidene-1-thio-α-D-mannopyranoside (102 mg, 69%) as a milky oil.

TLC: R_f 0.61 (EtOAc/*n*-heptane, 1:1 v/v); **^{1}H NMR** (500 MHz, CHLOROFORM-*D*) δ 8.10 – 8.03 (m, 2H), 7.59 (td, *J* = 7.3, 1.4 Hz, 1H), 7.51 – 7.43 (m, 6H), 7.36 – 7.29 (m, 6H), 7.28 – 7.23 (m, 2H), 7.23 – 7.15 (m, 3H), 5.65 (s, 1H), 5.59 (d, *J* = 1.4 Hz, 1H, H-1), 5.56 (dt, *J* = 9.9, 3.2 Hz, 1H, H-3), 4.72 – 4.54 (m, 2H), 4.49 (dt, *J* = 9.6, 4.8 Hz, 1H, H-5), 4.46 – 4.42 (m, 1H, H-4), 4.38 (dd, *J* = 3.4, 1.4 Hz, 1H, H-2), 4.28 (dd, *J* = 10.3, 4.5 Hz, 1H, H-6), 3.94 (t, *J* = 10.0 Hz, 1H, H-6).**^{13}C NMR** (126 MHz, CHLOROFORM-*D*) δ 165.9 (carbonyl), 137.4, 137.3, 133.8 (all quaternary), 133.3, 132.0, 130.1, 130.0, 129.3, 129.1, 128.6, 128.5, 128.5, 128.4, 128.1, 128.1, 127.9, 126.3 (all aromatic), 101.9, 86.7 (C-1), 77.9 (C-2), 76.5 (C-4), 73.3, 71.3 (d, *J* = 1.8 Hz, C-3), 68.7 (C-6), 65.5 (C-5).**HRMS** $[M + Na]^+$ calcd. for $C_{32}{}^{13}CH_{30}O_6S$ 578.16943, found 578.16719.

Phenyl 3-*O*-(benzoyl-α- ^{13}C)-2-*O*-benzyl-4,6-*O*-benzylidene-1-thiosulfinyl-α-D-mannopyranoside

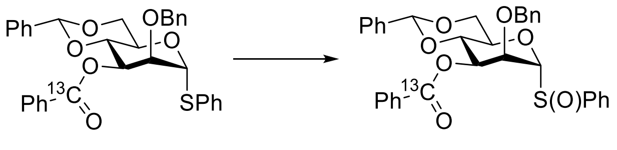

Via general S-oxidation procedure starting with phenyl 3-*O*-(benzoyl-α-^{13}C)-2-*O*-benzyl-4,6-*O*-benzylidene-1-thio-α-D-mannopyranoside (80 mg, 1 Eq, 0.14 mmol) to

afford phenyl 3-*O*-(benzoyl-α- ^{13}C)-2-*O*-benzyl-4,6-*O*-benzylidene-1-thiosulfinyl-α-D-mannopyranoside (69 mg, 84%) as an off-white solid.

TLC: R_f 0.33 (EtOAc/*n*-heptane, 1:1 v/v); **^{1}H NMR** (500 MHz, CHLOROFORM-*D*) δ 8.14 – 8.01 (m, 2H), 7.75 – 7.64 (m, 2H), 7.61 – 7.53 (m, 4H), 7.48 – 7.39 (m, 4H), 7.33 (dd, *J* = 5.2, 2.0 Hz, 3H), 7.20 – 7.14 (m, 2H), 7.14 – 7.01 (m, 4H), 5.87 (ddd, *J* = 10.5, 3.8, 3.0 Hz, 1H, H-3), 5.62 (s, 1H), 4.65 (dd, *J* = 3.8, 1.3 Hz, 1H, H-2), 4.56 (d, *J* = 1.2 Hz, 1H, H-1), 4.49 (d, *J* = 11.8 Hz, 1H), 4.43 (dd, *J* = 10.4, 9.2 Hz, 1H, H-4), 4.35 (d, *J* = 11.8 Hz, 1H), 4.33 – 4.30 (m, 1H, H-5), 4.30 – 4.25 (m, 1H, H-6), 3.79 (t, *J* = 9.9 Hz, 1H, H-6).**^{13}C NMR** (126 MHz, CHLOROFORM-*D*) δ 165.6 (carbonyl), 141.4, 137.1, 136.8 (all quaternary), 133.3, 131.9, 130.1, 130.0, 129.7, 129.2, 128.5, 128.5, 128.4, 128.4, 128.2, 128.1, 126.3, 124.7 (all aromatic), 102.0, 97.4 (C-1), 75.7 (C-4), 73.4, 72.7 (C-2), 71.1 (d, *J* = 2.4 Hz, C-3), 70.1 (C-5), 68.4 (C-6). **HRMS** [M + Na]$^+$ calcd. for $C_{32}{}^{13}CH_{30}O_7S$ 594.16435, found 594.16198.

Phenyl 3-*O*-(4-methoxybenzoyl -α- ^{13}C)-2-*O*-benzyl-4,6-*O*-benzylidene-1-thio-α-D-mannopyranoside

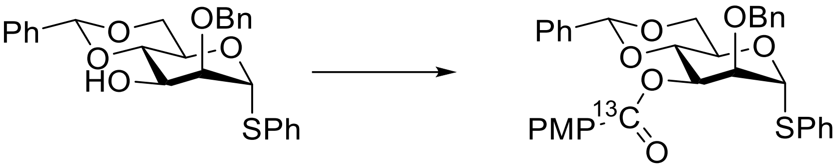

To phenyl 2-*O*-benzyl-4,6-*O*-benzylidene-1-thio-α-D-mannopyranoside(25 mg, 1 Eq, 55 µmol) dissolved in DCM (0.55 mL, 0.1 M) was added 4-methoxybenzoic acid-α- ^{13}C (9.3 mg, 1.1 Eq, 61 µmol). The mixture was activated by the addition of DIC (11 mg, 13 µL, 1.5 Eq, 83 µmol) and DMAP (0.68 mg, 0.1 Eq, 5.5 µmol) whereafter it was stirred for 60h at rt. The reaction mixture was then filtered and concentrated *in vacuo*. The crude material was purified using column purified using silica gel flash column chromatography (0→20% EtOAc/*n*-heptane, v/v) to afford phenyl 3-*O*-(4-methoxybenzoyl-α-^{13}C)-2-*O*-benzyl-4,6-*O*-benzylidene-1-thiosulfinyl-α-D-mannopyranoside (22 mg, 68%) as a clear oil.

TLC: R_f 0.56 (EtOAc/*n*-heptane, 1:1 v/v); **^{1}H NMR** (500 MHz, CHLOROFORM-*D*) δ 8.03 (dd, *J* = 8.9, 3.9 Hz, 2H), 7.46 (dt, *J* = 6.0, 1.4 Hz, 4H), 7.32 (dtd, *J* = 6.6, 3.5, 2.2 Hz, 6H), 7.25 – 7.16 (m, 5H), 6.97 – 6.83 (m, 2H), 5.64 (s, 1H), 5.58 (d, *J* = 1.5 Hz, 1H, H-1), 5.53 (dt, *J* = 10.1, 3.1 Hz, 1H, H-3), 4.66 (d, *J* = 11.9 Hz, 1H), 4.57 (d, *J* = 11.9 Hz, 1H), 4.47 (td, *J* = 9.6, 4.6 Hz, 1H, H-5), 4.42 (t, *J* = 9.8 Hz, 1H, H-4), 4.37 (dd, *J* = 3.4, 1.4 Hz, 1H, H-2), 4.27 (dd, *J* = 10.3, 4.7 Hz, 1H, H-6), 3.93 (t, *J* = 10.1 Hz, 1H, H-6), 3.88 (s, 3H). **^{13}C NMR** (126 MHz, CHLOROFORM-*D*) δ 165.7, 163.7, 137.4, 137.4, 133.9 (all quaternary), 132.1, 132.1, 131.9, 129.3, 129.1, 128.6, 128.4, 128.1, 128.1, 127.8, 126.3, 122.4 (d, J = 78.9 Hz, quaternary), 113.8, 113.8 (all aromatic), 101.9, 86.8 (C-1), 78.0 (C-2), 76.5 (d, *J* = 2.4 Hz, C-4), 73.4, 71.0 (d, *J* = 2.4 Hz, C-3), 68.7 (C-6), 65.5 (C-5), 55.6.**HRMS** [M + Na]$^+$ calcd. for $C_{33}{}^{13}CH_{32}O_7S$ 608.18000, found 608.18015.

Phenyl 2,3,4,6-tetra-*O*-benzyl-1-thio-β-D-glucopyranoside

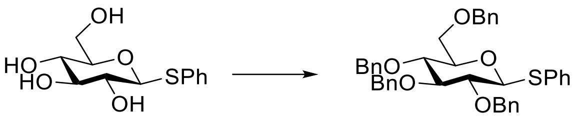

Phenyl 1-thio-α-D-glucopyranoside[7] (300 mg, 1.1 mmol, 1.0 eq.) was dissolved in dry DMF (10 mL) under inert atmosphere. The solution was cooled on ice (0 °C) and successively NaH (320 mg, 7.7 mmol, 7.0 eq., 60% on paraffin oil) and benzyl bromide (0.79 mL, 6.6 mmol, 6.0 eq.) were added. The reaction was stirred on ice for 15 minutes, heated to rt and stirred for 16h. The reaction was quenched with MeOH, diluted in Et_2O (50 mL), washed with water (3x 50 mL), and brine (50 mL). The combined aqueous phases were extracted with DCM (2x 20 mL). The combined organic phases was dried over MgSO4, filtrated, and the solvent was evaporated *in vacuo.* The crude was purified by silica gel flash column chromatography (0 →15% EtOAc/*n*-heptane, v/v) to yield the product (594 mg, 85%).

TLC: R_f 0.71 (EtOAc/*n*-heptane, 1:1 v/v);**^{1}H NMR** (500 MHz, CHLOROFORM-*D*) δ 7.73 – 7.49 (m, 2H), 7.49 – 7.04 (m, 23H), 4.95 – 4.79 (m, 4H), 4.74 (d, *J* = 10.2 Hz, 1H), 4.68 (d, *J* = 9.8 Hz, 1H), 4.64 – 4.58 (m, 2H), 4.55 (d, *J* = 11.9 Hz, 1H), 3.80 (dd, *J* = 10.9, 2.0 Hz, 1H, H-6), 3.77 – 3.72 (m, 1H, H-6), 3.73 – 3.69 (m, 1H, H-3), 3.66 (t, *J* = 9.3 Hz, 1H, H-4), 3.56 – 3.47 (m, 2H, H-2; H-5).**^{13}C NMR** (126 MHz, CHLOROFORM-*D*) δ 138.5, 138.4, 138.1, 133.9 (all quaternary), 132.1, 129.0, 128.6, 128.6, 128.5, 128.4, 128.1, 128.0, 128.0, 128.0, 127.9, 127.8, 127.7, 127.6 (all aromatic), 87.5 (C-1), 86.9 (C-3), 80.9 (C-2), 79.2 (C-5), 77.9 (C-4), 76.0, 75.6, 75.2, 73.5, 69.1 (C-6).**HRMS** [M + Na]$^+$ calcd. for $C_{40}H_{40}O_5S$ 655.24941, found 655.24675.

Phenyl 2,3,4,6-tetra-*O*-benzyl-1-thiosulfinyl-β-D-glucopyranoside

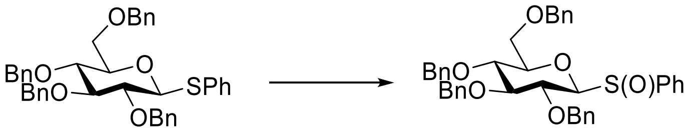

Via general S-oxidation procedure starting with phenyl 2,3,4,6-tetra-*O*-benzyl-1-thio-β-D-glucopyranoside (700 mg, 1 Eq, 1.11 mmol) to afford phenyl 2,3,4,6-tetra-*O*-benzyl-1-thiosulfinyl-β-D-glucopyranoside (605 mg, 84%) as an off-white solid (mixture of diasterioisomers, major/minor 2:1).

TLC: R_f 0.54 major, 0.46 minor (EtOAc/*n*-heptane 1:1 v/v); **^{1}H NMR** (500 MHz, CHLOROFORM-*D*) Major: δ 7.69 – 7.65 (m, 2H), 7.49 – 7.39 (m, 3H), 7.39 – 7.27 (m, 15H), 7.21 – 7.16 (m, 5H), 5.04 (d, *J* = 10.3 Hz, 1H), 4.98 (d, *J* = 10.3 Hz, 1H), 4.97 (d, *J* = 11.1 Hz, 1H), 4.93 (d, *J* = 11.1 Hz, 1H), 4.81 (d, *J* = 10.4 Hz, 1H), 4.59 (d, *J* = 11.0 Hz, 1H), 4.33 (d, *J* = 12.0 Hz, 1H), 4.23 (d, *J* = 12.1 Hz, 1H), 4.12 (dd, *J* = 9.8, 9.0 Hz, 1H, H-2), 3.99 (d, *J* = 9.8 Hz, 1H, H-1), 3.81 (t, *J* = 9.0 Hz, 1H, H-3), 3.59 (t, *J* = 9.5 Hz, 1H, H-4), 3.54 – 3.51 (m, 2H, H-6), 3.33 (ddd, *J* = 9.9, 5.0, 2.3 Hz, 1H, H-5).**^{13}C NMR** (126 MHz, CHLOROFORM-*D*) Major: δ 139.7, 138.4, 138.4, 137.8, 137.7 (all quaternary), 131.1, 128.9, 128.7, 128.6, 128.6, 128.6, 128.5, 128.4, 128.2, 128.0, 127.9, 127.8, 127.8, 127.8, 127.6, 125.4 (all aromatic), 93.6 (C-1), 86.7 (C-3), 80.8 (C-5), 77.7 (C-4), 76.9 (C-2), 76.0, 75.8, 75.2, 73.6, 68.9 (C-6). Minor: δ_H = 4.49 (d, *J* = 8.8 Hz, 1H, H-1), δ_C = 95.6 (C-1). **HRMS** [M + Na]$^+$ calcd. for $C_{33}H_{34}O_5S$ 671.24433, found 671.24302.

Phenyl 2,4,6-tri-*O*-benzyl-1-thio- β-D-glucopyranoside

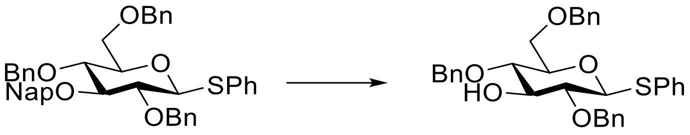

To a solution of phenyl 2,3,4,6-tetra-*O*-benzyl-1-thiosulfinyl-β-D-glucopyranoside[8] (900 mg, 1 Eq, 1.32 mmol) in in DCM/H_2O (9:1 v/v, 14.7 mL) was added DDQ (449 mg, 1.5 Eq, 1.98 mmol) and the resulting mixture was stirred in the dark for 2.5 h. The reaction mixture was diluted using DCM (40 mL). The organic phase was washed twice using an aqueous mixture of ascorbic acid (0.7%), citric acid (1.5%) and NaOH (0.9%) (w/v, 2 x 25 mL). The organic layer was dried over $MgSO_4$, filtered and concentrated *in vacuo*. The residue was purified using silica gel flash column chromatography (0→20% EtOAc/*n*-heptane, v/v) to afford phenyl 2,4,6-tri-*O*-benzyl-1-thio- β-D-glucopyranoside (570 mg, 80%) as a clear oil.

TLC: R_f 0.67 (EtOAc/*n*-heptane, 1:1 v/v);**^{1}H NMR** (500 MHz, CHLOROFORM-*D*) δ 7.65 – 7.52 (m, 2H), 7.42 – 7.17 (m, 18H), 4.96 (d, *J* = 11.0 Hz, 1H), 4.79 (d, *J* = 11.3 Hz, 1H), 4.68 (d, *J* = 11.3 Hz, 1H), 4.65 (d, *J* = 9.8 Hz, 1H, H-1), 4.63 (d, *J* = 1.6 Hz, 1H), 4.61 (s, 1H), 4.55 (d, *J* = 12.0 Hz, 1H), 3.80 (dd, *J* = 11.0, 1.9 Hz, 1H, H-6), 3.78 – 3.75 (m, 1H, H-3), 3.74 – 3.71 (m, 1H, H-6), 3.54 (dd, *J* = 9.8, 8.5 Hz, 1H, H-4), 3.49 (ddd, *J* = 9.8, 4.5, 1.9 Hz, 1H, H-5), 3.42 – 3.33 (m, 1H, H-2), 2.39 (d, *J* = 2.6 Hz, 1H, OH-3).**^{13}C NMR** (126 MHz, CHLOROFORM-*D*) δ 138.4, 138.4, 138.2, 134.0 (all quaternary), 131.9, 129.1, 128.8, 128.6, 128.5, 128.4, 128.2, 128.1, 128.0, 127.9, 127.7, 127.6 (all aromatic), 87.2 (C-1), 80.8 (C-2), 79.0 (C-5), 78.8 (C-3), 77.5 (C-4), 75.3, 74.8, 73.6, 69.2 (C-6).**HRMS** $[M + Na]^+$ calcd. for $C_{33}H_{34}O_5S$ 565.20246, found 565.20476.

Phenyl 3-*O*-benzoyl-2,4,6-tri-*O*-benzyl -1-thio- β-D-glucopyranoside

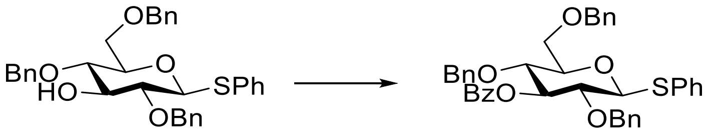

To a solution of phenyl 2,4,6-tri-*O*-benzyl-1-thio- β-D-glucopyranoside (200 mg, 1 Eq, 369 µmol) in pyridine (3.69 mL, 0.1 M), benzoyl chloride (64.8 mg, 53.5 µL, 1.25 Eq, 461 µmol) was added at 0°C, and the mixture was stirred overnight at rt. Subsequently, the solvent was removed *in vacuo* after which the residue was redissolved in DCM (30 mL). The organic layer was washed with sat. aq. $NaHCO_3$ (sat. aq., 10 mL) and brine (10 mL). The solution was dried over Na_2SO_4 and concentrated to get the benzoyl derivative as an oil, which was purified using silica gel flash column chromatography (0→20% EtOAc/*n*-heptane, v/v) to obtain phenyl 3-*O*-benzoyl-2,4,6-tri-*O*-benzyl -1-thio- β-D-glucopyranoside (178 mg, 75%) as a clear oil.

TLC: R_f 0.69 (EtOAc/*n*-heptane 1:1 v/v); **^{1}H NMR** (500 MHz, CHLOROFORM-*D*) δ 8.04 – 7.91 (m, 2H), 7.64 – 7.53 (m, 3H), 7.42 (t, *J* = 7.8 Hz, 2H), 7.40 – 7.34 (m, 5H), 7.30 – 7.24 (m, 4H), 7.17 – 7.09 (m, 7H), 7.07 – 6.99 (m, 2H), 5.58 (t, *J* = 9.2 Hz, 1H, H-3), 4.78 (d, *J* = 9.6 Hz, 1H, H-1), 4.78 (d, *J* = 10.7 Hz, 1H), 4.65 (d, *J* = 11.9 Hz, 1H), 4.56 (d, *J* = 12.1 Hz, 1H), 4.53 (d, *J* = 10.8 Hz, 1H), 4.50 – 4.48 (m, 2H), 3.87 – 3.81 (m, 1H, H-4), 3.79 – 3.75 (m, 2H, H-6), 3.65 – 3.61 (m, 1H, H-2), 3.61 – 3.58 (m, 1H, H-

5)**^{13}C NMR** (126 MHz, CHLOROFORM-*D*) δ 165.6 (carbonyl), 138.3, 137.6, 137.5, 133.7 (all quaternary), 133.2, 132.2, 130.1 (quaternary), 129.9, 129.1, 128.5, 128.5, 128.5, 128.4, 128.3, 128.2, 128.0, 127.9, 127.9, 127.8, 127.7 (all aromatic), 87.6 (C-1), 79.0 (C-5), 78.7 (C-2), 78.1 (C-3), 76.0 (C-4), 74.9, 74.6, 73.7, 68.8 (C-6). **HRMS** $[M + Na]^+$ calcd. for $C_{40}H_{38}O_6S$ 669.22868, found 669.22693.

Phenyl 3-*O*-benzoyl-2,4,6-tri-*O*-benzyl -1-thiosulfinyl- β-D-glucopyranoside

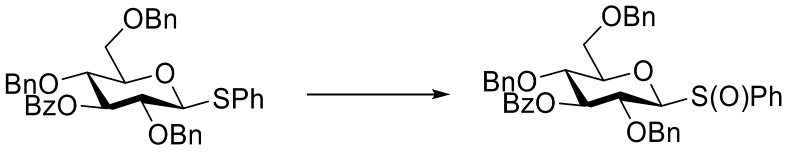

Via general S-oxidation procedure starting with phenyl 3-*O*-benzoyl-2,4,6-tri-*O*-benzyl -1-thio- β-D-glucopyranoside (150 mg, 1 Eq, 232 µmol) to afford phenyl 3-*O*-benzoyl-2,4,6-tri-*O*-benzyl -1-thiosulfinyl- β-D-glucopyranoside (95 mg, 62%) as an off-white solid (mixture of diasteroisomers 5:2).

TLC: R_f 0.50 (EtOAc/*n*-heptane, 1:1 v/v);**^{1}H NMR** (500 MHz, CHLOROFORM-*D*) Major: δ 8.01 (td, *J* = 8.4, 1.4 Hz, 3H), 7.69 – 7.64 (m, 2H), 7.58 – 7.53 (m, 1H), 7.50 – 7.43 (m, 6H), 7.38 – 7.35 (m, 3H), 7.27 – 7.21 (m, 6H), 7.14 – 7.03 (m, 4H), 5.68 (t, *J* = 9.3 Hz, 1H, H-3), 4.90 (d, *J* = 10.4 Hz, 1H), 4.70 (d, *J* = 10.5 Hz, 1H), 4.49 (s, 2H), 4.41 (d, *J* = 12.1 Hz, 1H), 4.31 (d, *J* = 12.1 Hz, 1H), 4.27 (t, *J* = 9.5 Hz, 1H, H-2), 4.08 (d, *J* = 9.7 Hz, 1H, H-1), 3.80 (t, *J* = 9.5 Hz, 1H, H-4), 3.59 (dd, *J* = 11.6, 4.7 Hz, 1H, H-6), 3.48 (dd, *J* = 11.6, 1.8 Hz, 1H, H-6), 3.41 (ddd, *J* = 9.8, 4.7, 1.7 Hz, 1H, H-5).**^{13}C NMR** (126 MHz, CHLOROFORM-*D*) δ 165.6 (carbonyl), 139.4, 138.3, 137.2, 137.0 (all quaternary), 133.5, 131.3, 129.9, 129.8 (quaternary), 129.0, 128.6, 128.5, 128.5, 128.4, 128.4, 128.2, 127.9, 127.7, 125.5 (all aromatic), 93.3 (C-1), 80.4 (C-5), 78.1 (C-3), 75.7, 75.6 (C-4), 75.2, 74.7, 73.7, 68.5 (C-6). Minor: δ_H = 4.57 (d, *J* = 7.9 Hz, 1H, H-1), δ_C = 95.8 (C-1). **HRMS** $[M + Na]^+$ calcd. for $C_{40}H_{38}O_7S$ 685.22359, found 685.22021.

Phenyl 3-*O*-(benzoyl-α- ^{13}C)-2,4,6-tri-*O*-benzyl -1-thio- β-D-glucopyranoside

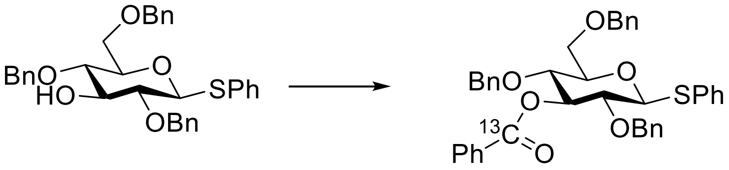

To phenyl 2,4,6-tri-*O*-benzyl-1-thio- β-D-glucopyranoside (200 mg, 1 Eq, 369 µmol) dissolved in DCM (3.69 mL, 0.1 M) was added benzoic acid-α-^{13}C (68.1 mg, 1.5 Eq, 553 µmol). The mixture was activated by the addition of EDCl (212 mg, 3 Eq, 1.11 mmol) and and DMAP (9.00 mg, 0.2 Eq, 73.7 µmol) whereafter it was stirred overnight at rt. The reaction mixture was then filtered and concentrated *in vacuo*. The crude material was purified using silica gel flash column chromatography (0→20% EtOAc/*n*-heptane, v/v) to obtain phenyl 3-*O*-(benzoyl-α- ^{13}C)-2,4,6-tri-*O*-benzyl -1-thio- β-D-glucopyranoside as an off white solid (161 mg, 67%).

TLC: R_f 0.69 (EtOAc/*n*-heptane, 1:1 v/v);**^{1}H NMR** (500 MHz, CHLOROFORM-*D*) δ 8.03 – 7.92 (m, 2H), 7.66 – 7.51 (m, 3H), 7.47 – 7.27 (m, 9H), 7.15 – 7.08 (m, 9H),

7.03 (dd, *J* = 7.1, 2.5 Hz, 2H), 5.57 (td, *J* = 9.2, 3.7 Hz, 1H, H-3), 4.81 – 4.75 (m, 2H, H-1), 4.64 (d, *J* = 12.0 Hz, 1H), 4.56 (d, *J* = 12.0 Hz, 1H), 4.52 (d, *J* = 10.7 Hz, 1H), 4.49 (d, *J* = 1.8 Hz, 2H), 3.88 – 3.78 (m, 1H, H-4), 3.80 – 3.74 (m, 2H, H-6), 3.65 – 3.61 (m, 1H, H-2), 3.61 – 3.58 (m, 1H, H-5).**^{13}C NMR** (126 MHz, CHLOROFORM-*D*) δ 165.6 (carbonyl), 138.3, 137.6, 137.5, 133.7 (all quaternary), 133.2, 132.2, 129.9, 129.9, 129.1, 128.5, 128.5, 128.5, 128.4, 128.3, 128.3, 128.0, 127.9, 127.9, 127.8, 127.8 (all aromatic), 87.7 (C-1), 79.0 (C-5), 78.7 (C-2), 76.0 (C-4), 74.9, 74.6, 73.7, 68.8 (C-6). **HRMS** $[M + Cs]^+$ calcd. for $C_{39}{}^{13}CH_{38}O_6S$ 670.23203, found 670.22895.

Phenyl 3-*O*-(benzoyl-α- ^{13}C)-2,4,6-tri-*O*-benzyl -1-thiosulfinyl- β-D-glucopyrano-side

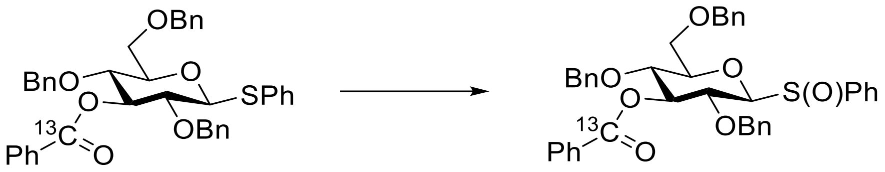

Via general S-oxidation procedure starting with phenyl 3-*O*-(benzoyl-α- ^{13}C)-2,4,6-tri-*O*-benzyl -1-thio- β-D-glucopyranoside (150 mg, 1 Eq, 232 µmol) to afford phenyl 3-*O*-(benzoyl-α- ^{13}C)-2,4,6-tri-*O*-benzyl -1-thiosulfinyl- β-D-glucopyranoside (102 mg, 66%) as an off-white solid (mixture of diastereoisomers 4:1).

TLC: R_f 0.39 major, 0.26 minor (EtOAc/*n*-heptane, 1:1 v/v); **^{1}H NMR** (500 MHz, CHLOROFORM-*D*) δ 8.06 – 7.97 (m, 2H), 7.70 – 7.64 (m, 2H), 7.59 – 7.54 (m, 1H), 7.49 – 7.44 (m, 4H), 7.37 – 7.33 (m, 3H), 7.25 – 7.22 (m, 4H), 7.20 – 7.17 (m, 3H), 7.13 – 7.09 (m, 4H), 7.05 – 7.01 (m, 2H), 5.68 (td, *J* = 9.3, 3.6 Hz, 1H, H-3), 4.90 (d, *J* = 10.4 Hz, 1H), 4.70 (d, *J* = 10.4 Hz, 1H), 4.49 (s, 2H), 4.41 (d, *J* = 12.1 Hz, 1H), 4.31 (d, *J* = 12.1 Hz, 1H), 4.27 (t, *J* = 9.5 Hz, 1H, H-2), 4.08 (d, *J* = 9.7 Hz, 1H, H-1), 3.80 (t, *J* = 9.5 Hz, 1H, H-4), 3.59 (dd, *J* = 11.6, 4.7 Hz, 1H, H-6), 3.48 (dd, *J* = 11.6, 1.8 Hz, 1H, H-6), 3.41 (ddd, *J* = 9.8, 4.7, 1.8 Hz, 1H, H-5).**^{13}C NMR** (126 MHz, CHLOROFORM-*D*) δ 165.6 (carbonyl), 139.4, 138.3, 137.2, 137.0 (all quaternary), 133.4, 131.3, 129.9 (d, *J* = 2.4 Hz), 129.0, 128.6, 128.5, 128.5, 128.4, 128.4, 128.2, 128.0, 127.9, 127.7, 125.5 (all aromatic), 93.3 (C-1), 80.5 (C-5), 78.1 (d, *J* = 3.0 Hz, C-3), 75.7, 75.6 (C-4), 75.2 (C-2), 74.7, 73.7, 68.5 (C-6). Minor: δ_H = 4.57 (d, *J* = 8.0 Hz, 1H, H-1), δ_C = 95.8 (C-1). **HRMS** $[M + Na]^+$ calcd. for $C_{39}{}^{13}CH_{38}O_7S$ 796.14262, found 796.14008.

Phenyl 2,3-di-*O*-benzyl-4,6-*O*-benzylidene-1-thio- β-D-glucopyranoside

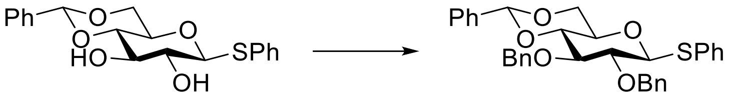

To a solution of phenyl 4,6-*O*-benzylidene-1-thio- β-D-glucopyranoside[9] (400 mg, 1 Eq, 1.11 mmol) in anhydrous DMF (11.1 mL, 0.1 M), NaH (266 mg, 60% Wt, 6 Eq, 6.66 mmol) was added at 0 °C. After stirring for 15 min, benzyl bromide (475 mg, 330 µL, 2.5 Eq, 2.77 mmol) was added dropwise. The reaction mixture was then stirred overnight at rt after which it quenched with MeOH (5 mL) and subsequently diluted with water (40 mL). Subsequently, the water layer was extracted with EtOAc (3 x 20 mL). The combined organic layers were washed with $NaHCO_3$ (sat. aq., 10 mL), brine (10 mL) and dried $MgSO_4$ and concentrate *in vacuo*. The resulting solid was then recrystallized from a mixture of Et_2O/pentane (v/v) to obtain phenyl 2,3-di-*O*-benzyl-4,6-*O*-benzylidene-1-thio- β-D-glucopyranoside (450 mg, 75%) as a white fluffy powder.

TLC: R_f 0.60 (EtOAc/*n*-heptane, 1:1 v/v);**^{1}H NMR** (500 MHz, CHLOROFORM-*D*) δ 7.58 – 7.51 (m, 2H), 7.51 – 7.46 (m, 2H), 7.44 – 7.27 (m, 16H), 5.59 (s, 1H), 4.94 (d, *J* = 11.2 Hz, 1H), 4.87 (d, *J* = 10.3 Hz, 1H), 4.82 (d, *J* = 10.3 Hz, 1H), 4.80 – 4.77 (m, 1H), 4.76 (d, *J* = 9.9 Hz, 1H, H-1), 4.39 (dd, *J* = 10.5, 5.0 Hz, 1H, H-6), 3.87 – 3.83 (m, 1H, H-3), 3.83 – 3.79 (m, 1H, H-6), 3.71 (t, *J* = 9.4 Hz, 1H, H-4), 3.54 – 3.51 (m, 1H, H-2), 3.50 – 3.44 (m, 1H, H-5).**^{13}C NMR** (126 MHz, CHLOROFORM-*D*) δ 138.4, 138.2, 137.4, 133.2 (all quaternary), 132.5, 129.2, 129.1, 128.5, 128.4, 128.4, 128.3, 128.0, 127.9, 126.1 (all aromatic), 101.3, 88.4 (C-1), 83.2 (C-3), 81.6 (C-4), 80.6 (C-2), 76.1, 75.5, 70.4 (C-5), 68.9 (C-6).**HRMS** $[M + Na]^+$ calcd. for $C_{33}H_{32}O_5S$ 563.18681, found 563.18732.

Phenyl 2,3-di-*O*-benzyl-4,6-*O*-benzylidene-1-thiosulfinyl- β-D-glucopyranoside

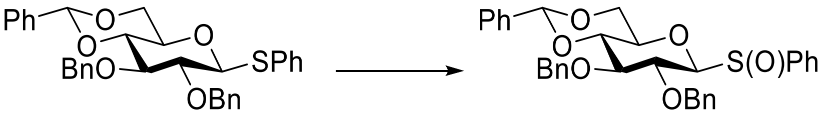

Via general S-oxidation procedure starting with phenyl 2,3-di-*O*-benzyl-4,6-*O*-benzylidene-1-thio- β-D-glucopyranoside (300 mg, 1 Eq, 555 µmol) to afford phenyl 2,3-di-*O*-benzyl-4,6-*O*-benzylidene-1-thiosulfinyl- β-D-glucopyranoside (210 mg, 68%) as an off-white solid (mixture of diastereoisomers 5:3).

TLC: R_f 0.48 major, 0.41 minor (EtOAc/*n*-heptane, 1:1 v/v);**^{1}H NMR** (500 MHz, CHLOROFORM-*D*) Major: δ 7.63 – 7.60 (m, 2H), 7.55 – 7.49 (m, 3H), 7.47 – 7.42 (m, 3H), 7.40 – 7.27 (m, 12H), 5.53 (s, 1H), 5.00 (d, *J* = 11.5 Hz, 1H), 4.92 (d, *J* = 11.1 Hz, 1H), 4.83 (d, *J* = 11.5 Hz, 1H), 4.71 (d, *J* = 11.1 Hz, 1H), 4.02 (d, *J* = 9.9 Hz, 1H, H-1), 4.02 (dd, *J* = 10.4, 5.0 Hz, 1H, H-6), 3.94 – 3.90 (m, 1H, H-3), 3.83 – 3.79 (m, 1H, H-2), 3.79 – 3.77 (m, 1H, H-4), 3.74 (d, *J* = 10.4 Hz, 1H, H-6), 3.29 (td, *J* = 9.7, 4.9 Hz, 1H, H-5). **^{13}C NMR** (126 MHz, CHLOROFORM-*D*) δ 139.5, 138.3, 137.6, 137.1 (all quaternary), 131.2, 129.1, 129.0, 128.6, 128.5, 128.3, 128.2, 128.1, 127.9, 127.7, 126.1, 125.2 (all aromatic), 101.3, 93.8 (C-1), 82.7 (C-3), 81.2 (C-4), 75.1 (C-2), 71.1

(C-5), 68.1 (C-6). Minor: δ_H = 4.60 (d, *J* = 9.1 Hz, 1H, H-1), δ_C = 95.7 (C-1). **HRMS** [M + Na]$^+$ calcd. for $C_{33}H_{32}O_5S$ 579.18173, found 579.18195.

Phenyl 2-*O*-benzyl-4,6-*O*-benzylidene-1-thio- β-D-glucopyranoside

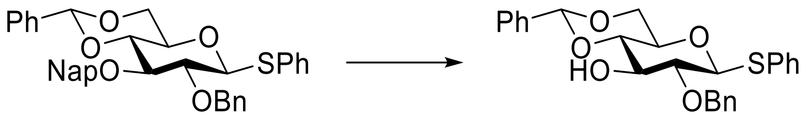

Phenyl 4,6-O-benzylidene-3-O-(2-methylnaphtyl)-1-thio-α-D-glucopyranoside[6] (350 mg, 0.59 mmol, 1.0 eq.) was dissolved in DCM (6 mL) and water (2.0 mL) was added. The suspension was stirred vigorously in the dark and DDQ (160 mg, 0.71 mmol, 1.2 eq.) was added. The reaction was stirred for 2h in the dark after which TLC displayed complete consumption of the starting material. The reaction mixture was diluted with DCM (34 mL); washed (3x 50 mL) with an aqueous solution containing 0.7% ascorbic acid, 0.9% NaOH, and 1.5% citric acid; followed by extraction of the combined aqueous phases with DCM (2x 50 mL). The combined organic phases were dried over MgSO4, filtrated, and the solvent was evaporated *in vacuo.* The crude product was purified by silica gel flash column chromatography (0→40% EtOAc/*n*-heptane, v/v) to obtain the product (220 mg, 82%).

TLC: R_f = 0.57 (EtOAc/*n*-heptane, 1/4 v/v);**^{1}H NMR** (500 MHz, CHLOROFORM-*D*) δ 7.59 – 7.51 (m, 2H), 7.50 – 7.45 (m, 2H), 7.45 – 7.39 (m, 2H), 7.40 – 7.30 (m, 9H), 5.53 (s, 1H), 4.94 (d, *J* = 10.9 Hz, 1H), 4.81 (d, *J* = 10.9 Hz, 1H), 4.76 (d, *J* = 9.8 Hz, 1H, H-1), 4.37 (dd, *J* = 10.5, 4.9 Hz, 1H, H-6), 3.98 – 3.85 (m, 1H, H-3), 3.79 (t, *J* = 10.2 Hz, 1H, H-6), 3.54 (t, *J* = 9.3 Hz, 1H, H-4), 3.51 – 3.46 (m, 1H, H-5), 3.46 – 3.41 (m, 1H, H-2), 2.52 (d, *J* = 2.4 Hz, 1H, OH-3).**^{13}C NMR** (126 MHz, CHLOROFORM-*D*) δ 138.1, 137.0, 133.2 (all quaternary), 132.3, 129.4, 129.2, 128.7, 128.5, 128.4, 128.2, 128.1, 126.4 (all aromatic), 101.9, 88.1 (C-1), 80.8 (C-2), 80.3 (C-4), 75.6, 75.5 (C-3), 70.2 (C-5), 68.8 (C-6). **HRMS** [M + Na]$^+$ calcd. for $C_{26}H_{26}O_5S$ 473.13986, found 473.14016.

Phenyl 3-*O*-benzoyl-2-*O*-benzyl-4,6-*O*-benzylidene-1-thio- β-D-glucopyranoside

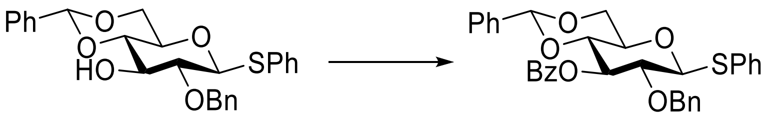

To a solution of benzoic acid (56.9 mg, 2.1 Eq, 466 µmol) in dry ACN (2.22 mL, 0.1 M), was added CDI (36.0 mg, 1 Eq, 222 µmol) under N_2. The reaction mixture was stirred at 70 °C for 2h before phenyl 2-*O*-benzyl-4,6-*O*-benzylidene-1-thio- β-D-glucopyranoside (100 mg, 1 Eq, 222 µmol) in CH_3CN (0.5 mL) was added dropwise followed by DBU (71.0 mg, 70.3 µL, 2.1 Eq, 466 µmol). The reaction mixture was stirred at 60 °C overnight, cooled and poured into $NaHCO_3$ (sat. aq., 1 mL). The water layer was extracted with DCM (3 x 10 mL). The combined organic phases were washed with brine (2 mL), dried over $MgSO_4$ and concentrated *in vacuo*. The residue was purified using silica gel flash column chromatography (0→20% EtOAc/*n*-heptane, v/v) to afford phenyl 3-*O*-benzoyl-2-*O*-benzyl-4,6-*O*-benzylidene-1-thio- β-D-glucopyranoside as a clear sticky solid (65 mg, 53%).

TLC: R_f 0.72 (EtOAc/*n*-heptane, 1:1, v/v); **^{1}H NMR** (500 MHz, CHLOROFORM-*D*) δ 8.12 – 8.01 (m, 2H), 7.67 – 7.53 (m, 3H), 7.49 – 7.28 (m, 10H), 7.21 – 7.13 (m, 5H), 5.70 (dd, *J* = 9.9, 8.6 Hz, 1H, H-3), 5.51 (s, 1H), 4.93 (d, *J* = 9.7 Hz, 1H, H-1), 4.88 (d, *J* = 10.6 Hz, 1H), 4.64 (d, *J* = 10.5 Hz, 1H), 4.42 (dd, *J* = 10.6, 5.0 Hz, 1H, H-6), 3.89 – 3.82 (m, 1H, H-6), 3.82 – 3.78 (m, 1H, H-4), 3.77 – 3.71 (m, 1H, H-2), 3.68 – 3.60 (m, 1H, H-5). **^{13}C NMR** (126 MHz, CHLOROFORM-*D*) δ 165.5 (carbonyl), 137.3, 136.9 (all quaternary), 133.2, 133.1 (quaternary), 132.4, 130.0 (quaternary), 129.9, 129.3, 129.1, 128.5, 128.4, 128.4, 128.3, 128.1, 128.0, 126.2 (all aromatic), 101.5, 88.7 (C-1), 79.4 (C-2), 78.8 (C-4), 75.5, 75.2 (C-3), 70.6 (C-5), 68.8 (C-6).**HRMS** [M + Na]$^+$ calcd. for $C_{33}H_{30}O_6S$ 577.16608, found 577.16760.

phenyl 3-*O*-benzoyl-2-*O*-benzyl-4,6-*O*-benzylidene-1-thiosulfinyl- β-D-glucopyranoside

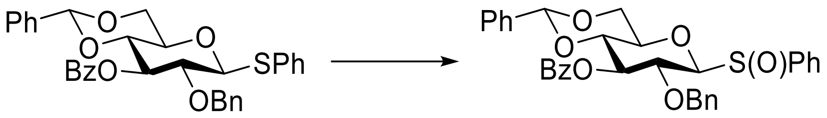

Via general S-oxidation procedure starting with phenyl 3-*O*-benzoyl-2-*O*-benzyl-4,6-*O*-benzylidene-1-thio- β-D-glucopyranoside (50 mg, 1 Eq, 90 µmol) to afford phenyl 3-*O*-benzoyl-2-*O*-benzyl-4,6-*O*-benzylidene-1-thiosulfinyl- β-D-glucopyranoside as a white powder (25 mg, 49%).

TLC: R_f 0.35 (EtOAc/*n*-heptane, 1:1 v/v);**^{1}H NMR** (500 MHz, CHLOROFORM-*D*) δ 8.11 – 8.01 (m, 2H), 7.69 – 7.62 (m, 2H), 7.60 – 7.56 (m, 1H), 7.56 – 7.52 (m, 4H), 7.49 – 7.44 (m, 2H), 7.36 – 7.32 (m, 2H), 7.30 – 7.20 (m, 7H), 5.77 (dd, *J* = 9.7, 8.8 Hz, 1H, H-3), 5.44 (s, 1H), 4.93 (d, *J* = 10.5 Hz, 1H), 4.78 (d, *J* = 10.5 Hz, 1H), 4.38 (dd, *J* = 9.6, 8.9 Hz, 1H, H-2), 4.16 (d, *J* = 9.6 Hz, 1H, H-1), 4.04 (dd, *J* = 10.6, 4.9 Hz, 1H, H-6), 3.84 (t, *J* = 9.6 Hz, 1H, H-4), 3.76 (t, *J* = 10.3 Hz, 1H, H-6), 3.44 (td, *J* = 9.8, 5.0 Hz, 1H, H-5).**^{13}C NMR** (126 MHz, CHLOROFORM-*D*) δ 165.5 (carbonyl), 139.1, 136.9, 136.7 (all quaternary), 133.5, 131.5, 130.0, 129.7 (quaternary), 129.2, 128.7, 128.6, 128.6, 128.3, 128.3, 126.2, 125.3 (all aromatic), 101.6, 93.6 (C-1), 78.4 (C-4), 76.1, 75.5 (C-2), 75.2 (C-3), 71.2 (C-5), 68.1 (C-6). **HRMS** [M + Na]$^+$ calcd. for $C_{33}H_{30}O_7S$ 593.16099, found 593.16019.

Phenyl 3-*O*-(benzoyl-α- ^{13}C)-2-*O*-benzyl-4,6-*O*-benzylidene-1-thio- β-D-glucopyranoside

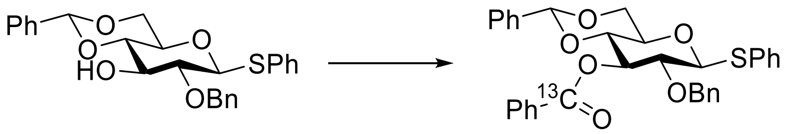

To a solution of benzoic acid-α-^{13}C (57.4 mg, 2.1 Eq, 466 µmol) in dry ACN (2.22 mL, 0.1 M), was added CDI(36.0 mg, 1 Eq, 222 µmol) under N_2. The reaction mixture was stirred at 70 °C for 2h before phenyl 2-*O*-benzyl-4,6-*O*-benzylidene-1-thio- β-D-glucopyranoside (100 mg, 1 Eq, 222 µmol) in ACN (0.5 mL) was added dropwise followed by DBU (71.0 mg, 70.3 µL, 2.1 Eq, 466 µmol). The reaction mixture was stirred at 60 °C overnight, cooled and poured into $NaHCO_3$ (sat. aq., 1 mL). The water layer was extracted with DCM (3 x 10 mL). The combined organic phases were washed with brine (2 mL), dried over $MgSO_4$ and concentrated *in vacuo*. The residue was purified

using silica gel flash column chromatography (0→20% Et_2O/toluene, v/v) to afford phenyl 3-*O*-benzoyl-2-*O*-benzyl-4,6-*O*-benzylidene-1-thio- β-D-glucopyranoside as an off-white solid (65 mg, 53%).

TLC: R_f 0.72 (EtOAc/*n*-heptane, 1:1 v/v); **^{1}H NMR** (500 MHz, CHLOROFORM-*D*) δ 8.08 – 7.97 (m, 2H), 7.65 – 7.52 (m, 3H), 7.48 – 7.25 (m, 10H), 7.22 – 7.13 (m, 5H), 5.76 – 5.61 (m, 1H, H-3), 5.51 (s, 1H), 4.91 (d, *J* = 9.7 Hz, 1H, H-1), 4.86 (d, *J* = 10.6 Hz, 1H), 4.62 (d, *J* = 10.5 Hz, 1H), 4.41 (dd, *J* = 10.5, 5.0 Hz, 1H, H-6), 3.88 – 3.81 (m, 1H, H-6), 3.82 – 3.75 (m, 1H, H-4), 3.75 – 3.70 (m, 1H, H-2), 3.63 (td, *J* = 9.7, 4.9 Hz, 1H, H-5). **^{13}C NMR** (126 MHz, CHLOROFORM-*D*) δ 165.5 (carbonyl), 137.3, 137.0 (all quaternary), 133.2, 133.2 (quaternary), 132.4, 129.9, 129.9, 129.3, 129.1, 128.5, 128.5, 128.4, 128.4, 128.3, 128.2, 128.0, 126.2 (all aromatic), 101.5, 88.7 (C-1), 79.4 (C-2), 78.8 (C-4), 75.5, 75.2 (d, *J* = 2.4 Hz, C-3), 70.6 (C-5), 68.8 (C-6). **HRMS** [M + Na]$^+$ calcd. for $C_{32}{}^{13}CH_{30}O_6S$ 578.16943, found 578.16922.

Phenyl 3-*O*-(benzoyl-α- ^{13}C)-2-*O*-benzyl-4,6-*O*-benzylidene-1-thiosulfinyl- β-D-glucopyranoside

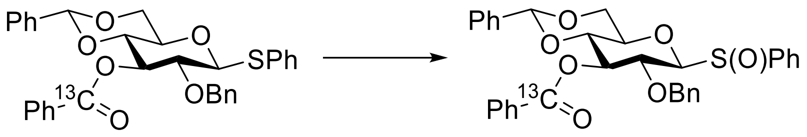

Via general S-oxidation procedure starting with phenyl 3-*O*-(benzoyl-α- ^{13}C)-2-*O*-benzyl-4,6-*O*-benzylidene-1-thio- β-D-glucopyranoside (65 mg, 1 Eq, 0.12 mmol) to afford phenyl 3-*O*-(benzoyl-α- ^{13}C)-2-*O*-benzyl-4,6-*O*-benzylidene-1-thiosulfinyl- β-D-glucopyranoside as a white powder (35 mg, 52 %)

TLC: R_f 0.35 (EtOAc/*n*-heptane, 1:1 v/v);**^{1}H NMR** (500 MHz, CHLOROFORM-*D*) δ 8.14 – 8.02 (m, 2H), 7.68 – 7.62 (m, 2H), 7.60 – 7.51 (m, 4H), 7.46 (td, *J* = 7.9, 1.2 Hz, 2H), 7.36 – 7.31 (m, 2H), 7.29 – 7.23 (m, 8H), 5.77 (ddd, *J* = 9.8, 8.9, 3.6 Hz, 1H, H-3), 5.44 (s, 1H), 4.93 (d, *J* = 10.5 Hz, 1H), 4.78 (d, *J* = 10.5 Hz, 1H), 4.39 (dd, *J* = 9.7, 8.9 Hz, 1H, H-2), 4.17 (d, *J* = 9.6 Hz, 1H, H-1), 4.04 (dd, *J* = 10.5, 4.9 Hz, 1H, H-6), 3.84 (t, *J* = 9.6 Hz, 1H, H-4), 3.76 (t, *J* = 10.3 Hz, 1H, H-6), 3.45 (td, *J* = 9.7, 4.9 Hz, 1H, H-5). **^{13}C NMR** (126 MHz, CHLOROFORM-*D*) δ 165.5 (carbonyl), 139.1, 136.8, 136.7 (all quaternary), 133.5, 131.5, 130.0, 130.0, 129.4 (quaternary), 129.1, 128.7, 128.6, 128.6, 128.6, 128.3, 128.3, 126.2, 125.3 (all aromatic), 101.6, 93.6 (C-1), 78.4 (C-4), 76.1, 75.5 (C-2), 75.2 (d, *J* = 3.0 Hz, C-3), 71.2 (C-5), 68.1 (C-6). **HRMS** [M + Na]$^+$ calcd. for $C_{32}{}^{13}CH_{30}O_7S$ 594.16435, found 594.16652.

p-methylphenyl 4,6-*O*-benzylidene-1-^{13}C-thio- β-D-glucopyranoside

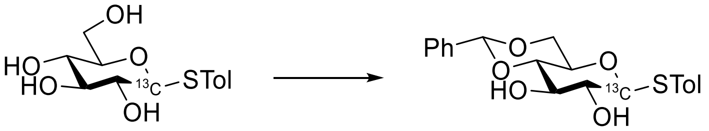

To a solution of *p*-methylphenyl 1-thio-^{13}C- β -D-glucopyranoside (250 mg, 1 Eq, 873 µmol) and $PhCH(OMe)_2$ (266 mg, 262 µL, 2 Eq, 1.75 mmol) in DMF (4.37 mL, 0.2 M) was added *p*-TsOH (until the pH of the solution reached 2–3). The resulting mixture, was stirred at 60°C under reduced pressure for 3h. Upon completion the solution was neutralized with triethylamine and diluted using EtOAc (30 mL). The organic layer was

washed with brine (15 mL), dried over $MgSO_4$ and concentrated *in vacuo*. The residue was purified using silica gel flash column chromatography (0→20% Et_2O/toluene, v/v) to afford phenyl 4,6-*O*-benzylidene-1-^{13}C-thio- β-D-glucopyranoside as a white solid (180 mg, 55%).

TLC: R_f 0.52 (EtOAc/*n*-heptane, 1:1 v/v); **^{1}H NMR** (499 MHz, CHLOROFORM-*D*) δ 7.51 – 7.46 (m, 2H), 7.45 – 7.41 (m, 2H), 7.38 – 7.33 (m, 3H), 7.20 – 7.10 (m, 2H), 5.52 (s, 1H), 4.56 (dd, *J* = 155.8, 9.7 Hz, 1H, H-1), 4.39 – 4.35 (m, 1H, H-6), 3.83 (t, *J* = 8.2 Hz, 1H. H-3), 3.79 – 3.74 (m, 1H, H-6), 3.52 – 3.47 (m, 2H, H-4, H-5), 3.46 – 3.38 (m, 1H, H-2), 2.85 (d, *J* = 2.2 Hz, 1H, OH-3), 2.70 (t, *J* = 2.8 Hz, 1H, OH-2), 2.36 (s, 3H).**^{13}C NMR** (126 MHz, CHLOROFORM-*D*) δ 139.0, 137.0 (all quaternary), 133.9, 130.1, 129.5, 128.5, 127.3 (quaternary), 126.4, 102.1, 88.9 (C-1), 80.4 (C-4), 74.7 (C-3), 72.6 (d, *J* = 39.5 Hz, C-2), 70.7 (d, *J* = 1.8 Hz, C-5), 68.7 (d, *J* = 5.4 Hz, C-6), 21.3. **HRMS** $[M + Na]^+$ calcd. for $C_{19}{}^{13}CH_{22}O_5S$ 398.11192, found 398.11137.

***p*-methylphenyl 2,3-di-*O*-benzyl-4,6-*O*-benzylidene-1-^{13}C -thio- β-D-glucopyranoside**

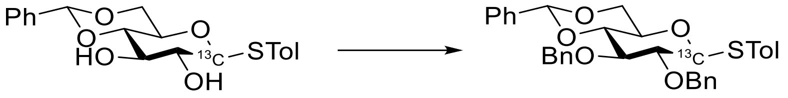

To a solution of *p*-methylphenyl 4,6-*O*-benzylidene-1-^{13}C-thio- β-D-glucopyranoside (130 mg, 1 Eq, 347 μmol) in anhydrous DMF (3.47 mL, 0.1 M), NaH (83.3 mg, 60% Wt, 6 Eq, 2.08 mmol) was added at 0 °C. After stirring for 15 min, benzyl bromide (148 mg, 103 µL, 2.5 Eq, 868 µmol) was added dropwise. The reaction mixture was then stirred overnight at rt after which it quenched with MeOH (5 mL) and subsequently diluted with water (40 mL). Subsequently, the water layer was extracted with EtOAc (3 x 20 mL). The combined organic layers were washed with $NaHCO_3$ (sat. aq., 10 mL), brine (10 mL) and dried $MgSO_4$ and concentrate *in vacuo*. The resulting solid was then recrystallized from recrystallized from a mixture of Et_2O/pentane to obtain *p*-methylphenyl 2,3-di-*O*-benzyl-4,6-*O*-benzylidene-1-^{13}C -thio- β-D-glucopyranoside (140 mg, 73%) as a white fluffy powder.

TLC: R_f 0.68 (EtOAc/*n*-heptane, 1:1 v/v); **^{1}H NMR** (499 MHz, CHLOROFORM-*D*) δ 7.57 – 7.26 (m, 17H), 7.15 – 7.08 (m, 2H), 5.58 (s, 1H), 4.93 (d, *J* = 11.1 Hz, 1H), 4.87 (d, *J* = 10.3 Hz, 1H), 4.81 (d, *J* = 10.3 Hz, 1H), 4.77 (d, *J* = 11.1 Hz, 1H), 4.69 (dd, *J* = 156.9, 9.8 Hz, 1H, H-1), 4.38 (dd, *J* = 10.5, 5.0 Hz, 1H, H-6), 3.86 – 3.80 (m, 1H, H-3), 3.85 – 3.75 (m, 1H, H-6), 3.68 (t, *J* = 9.4 Hz, 1H, H-4), 3.52 – 3.47 (m, 1H, H-2), 3.47 – 3.40 (m, 1H, H-5), 2.34 (s, 3H).**^{13}C NMR** (126 MHz, CHLOROFORM-*D*) δ 138.5, 138.4, 138.3, 137.4 (all quaternary), 133.2, 129.9, 129.1, 128.6, 128.4, 128.4, 128.3, 128.0, 127.9, 126.1 (all aromatic), 101.3, 88.7 (C-1), 83.2 (d, *J* = 3.0 Hz, C-3), 81.6 (C-4), 80.5 (d, *J* = 40.0 Hz, C-2), 76.0, 75.5, 70.4 (C-5), 68.9 (d, *J* = 5.5 Hz, C-6), 21.3. **HRMS** $[M + Na]^+$ calcd. for $C_{33}{}^{13}CH_{34}O_5S$ 578.20582, found 578.20303.

_p_-methylphenyl 2,3-di-_O_-benzyl-4,6-_O_-benzylidene-1-^{13}C -thiosulfinyl- β-D-gluco-pyranoside

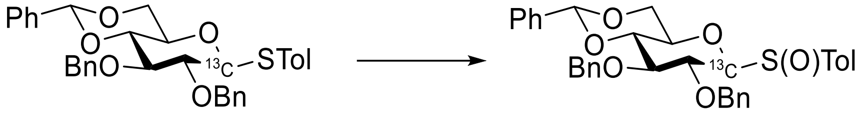

Via general S-oxidation procedure starting with *p*-methylphenyl 2,3-di-*O*-benzyl-4,6-*O*-benzylidene-1-^{13}C -thio- β-D-glucopyranoside (95 mg, 1 Eq, 0.17 mmol) to afford *p*-methylphenyl 2,3-di-*O*-benzyl-4,6-*O*-benzylidene-1-^{13}C -thiosulfinyl- β-D-glucopyranoside (70 mg, 72%) as a white powder (as mixture of isomers 5:4).

TLC: R_f 0.56 major; 0.48 minor (EtOAc/*n*-heptane, 1:1 v/v); Major: **^{1}H NMR** (500 MHz, CHLOROFORM-*D*) δ 7.51 (d, *J* = 8.2 Hz, 2H), 7.47 – 7.26 (m, 15H), 7.20 – 7.16 (m, 2H), 5.54 (s, 1H), 5.05 (d, *J* = 10.4 Hz, 1H), 5.00 (d, *J* = 11.5 Hz, 1H), 4.97 (d, *J* = 10.4 Hz, 1H), 4.83 (d, *J* = 11.5 Hz, 1H), 4.20 – 4.12 (m, 1H, H-2), 4.07 (dd, *J* = 10.5, 5.0 Hz, 1H, H-6), 4.00 (dd, *J* = 165.3, 9.8 Hz, 1H, H-1), 3.96 – 3.88 (m, 1H, H-3), 3.82 – 3.79 (m, 1H, H-4), 3.78 – 3.74 (m, 1H, H-6), 3.30 (tdd, *J* = 9.9, 5.0, 2.1 Hz, 1H, H-5), 2.43 (s, 3H). **^{13}C NMR** (126 MHz, CHLOROFORM-*D*) δ 141.8, 138.0, 137.2, 136.3, 136.2 (all quaternary), 129.8, 129.2, 128.6, 128.6, 128.5, 128.4, 128.3, 128.1, 127.9, 127.7, 126.1, 125.4 (all aromatic), 101.4, 93.8 (C-1), 82.8 (d, *J* = 2.4 Hz, C-3), 81.3 (C-4), 76.4 (d, *J* = 40.2 Hz, C-2), 76.3 (d, *J* = 2.4 Hz), 75.1, 71.1 (d, *J* = 2.4 Hz, C-5), 68.2 (d, *J* = 5.0 Hz, C-6), 21.6. Minor: δ_H = 4.59 (dd, *J* = 156.1, 9.9 Hz, 1H, H-1), δ_C = 95.4 (C-1). **HRMS** $[M + Na]^+$ calcd. for $C_{33}{}^{13}CH_{34}O_6S$ 594.20073, found 594.19968.

Phenyl 3-_O_-(benzoyl-α- ^{13}C)-2,4,6-tri-_O_-methyl-1-thiosulfinyl-α-D-mannopyrano-side

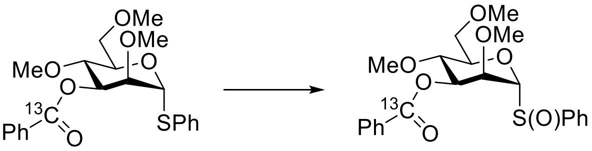

Via general S-oxidation procedure starting with phenyl 3-*O*-(benzoyl-α- ^{13}C)-2,4,6-tri-*O*-1-thio-α-D-mannopyranoside (96 mg, 1 Eq, 0.23 mmol) to afford phenyl 3-*O*-(benzoyl-α- ^{13}C)-2,4,6-tri-*O*-methyl-1-thiosulfinyl-α-D-mannopyrano-side (41 mg, 41%) as a colourless oil.

TLC: R_f 0.25 (Et_2O/toluene, 2/3 v/v); **^{1}H NMR** (500 MHz, $CDCl_3$) δ 8.17 – 8.03 (m, 2H), 7.75 – 7.66 (m, 2H), 7.63 – 7.57 (m, 1H), 7.57 – 7.49 (m, 3H), 7.49 – 7.41 (m, 2H), 5.73 (dt, *J* = 8.1, 3.3 Hz, 1H, H-3), 4.58 (d, *J* = 2.8 Hz, 1H, H-1), 4.31 (t, *J* = 3.2 Hz, 1H, H-2), 4.19 – 4.01 (m, 1H, H-5), 3.81 (dd, *J* = 9.6, 8.2 Hz, 1H, H-4), 3.65 – 3.57 (m, 2H, H-6), 3.50 (s, 3H), 3.39 (s, 3H), 3.25 (s, 3H).**^{13}C NMR** (126 MHz, $CDCl_3$) δ 165.7 (carbonyl), 141.6 (quaternary), 133.4, 131.5, 130.0, 130.0, 128.7, 128.6, 124.7 (all aromatic), 96.0 (C-1), 77.2 (C-5), 74.9 (d, *J* = 1.8 Hz, C-4), 74.3 (C-2), 72.8 (d, *J* = 2.6 Hz, C-3), 71.5 (C-6), 60.3, 59.5, 58.7. **HRMS** $[M + Na]^+$ calcd. for $C_{21}{}^{13}CH_{26}O_7S$ 458.13305, found 458.13224.

Supporting VT NMR experimental data

Activation spectra for glycosyl donors

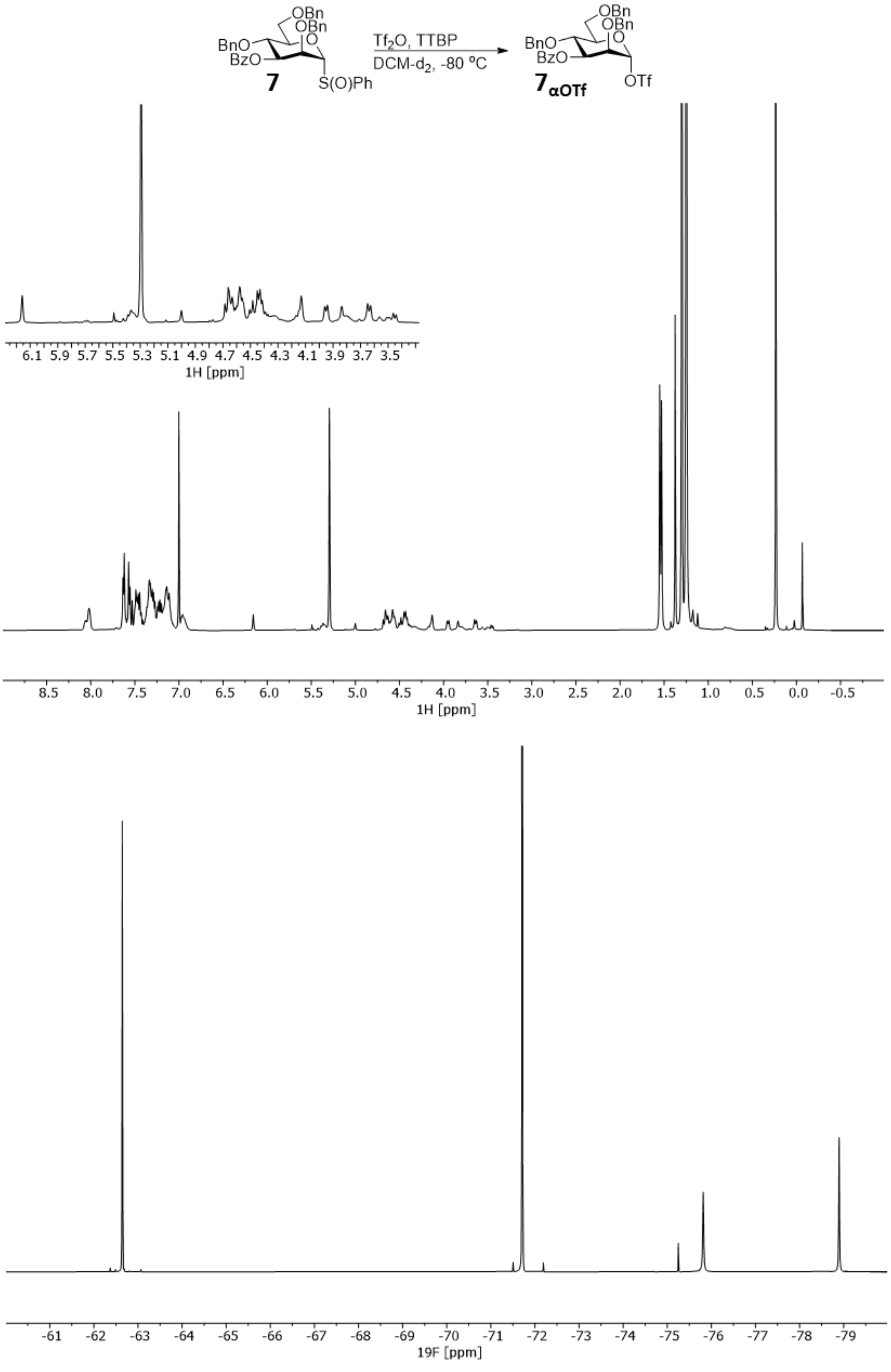

Figure S4: ^{1}H and ^{19}F activation spectra of the α-triflate from the corresponding donor. Minor decomposition was observed due to formation of benzyl triflate at δ_F = 75.2 ppm.

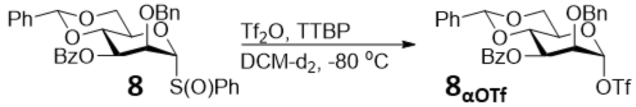

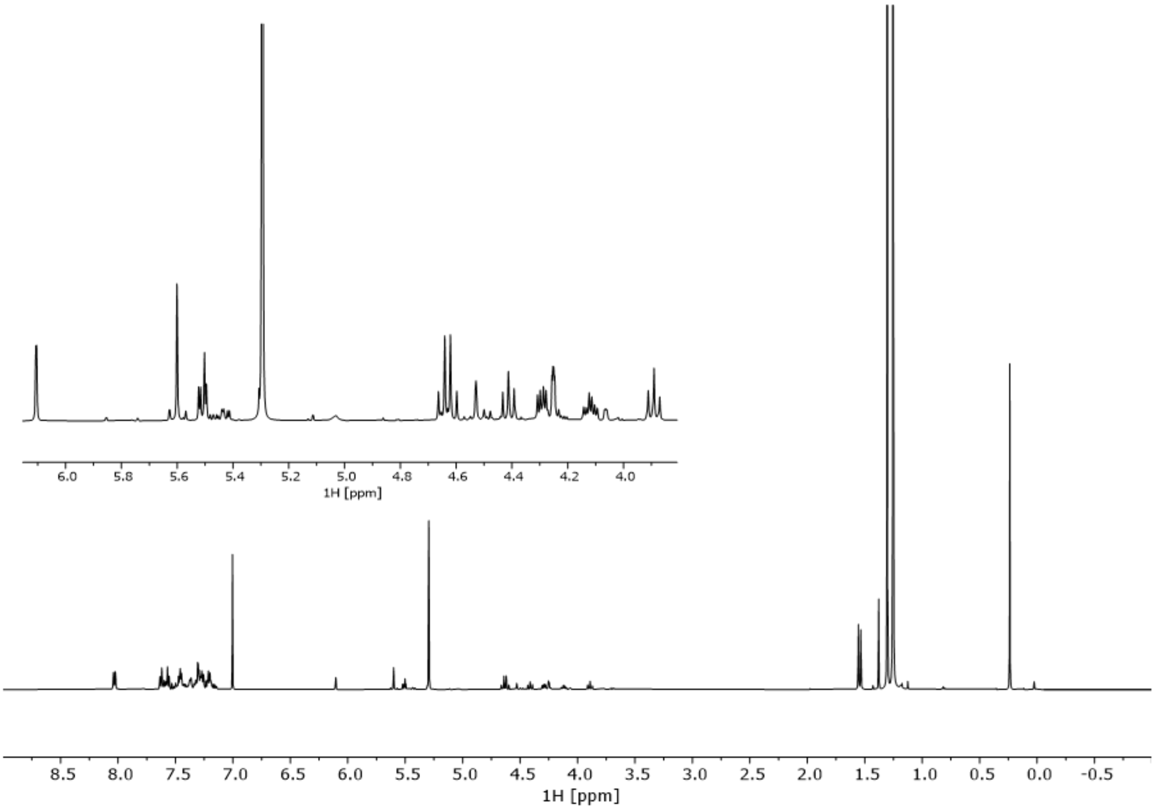

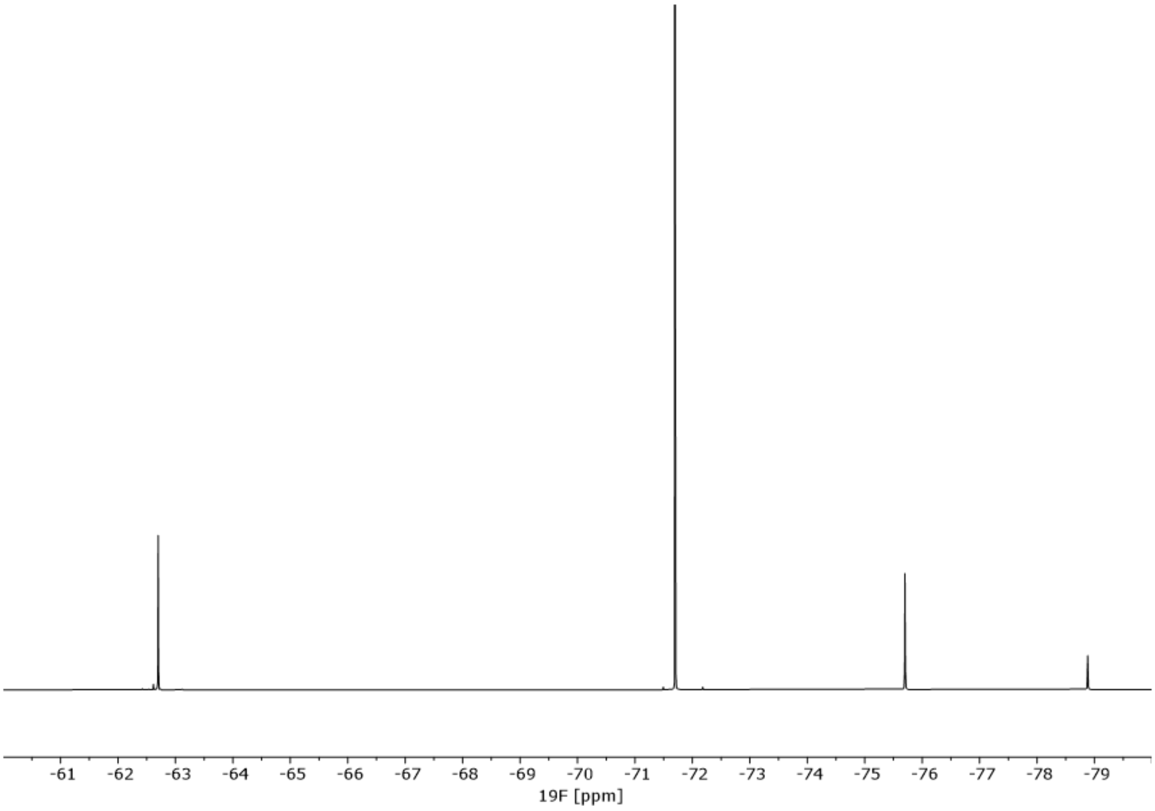

Figure S5: ${}^{1}H$ and ${}^{19}F$ activation spectra of the α-triflate from the corresponding donor.

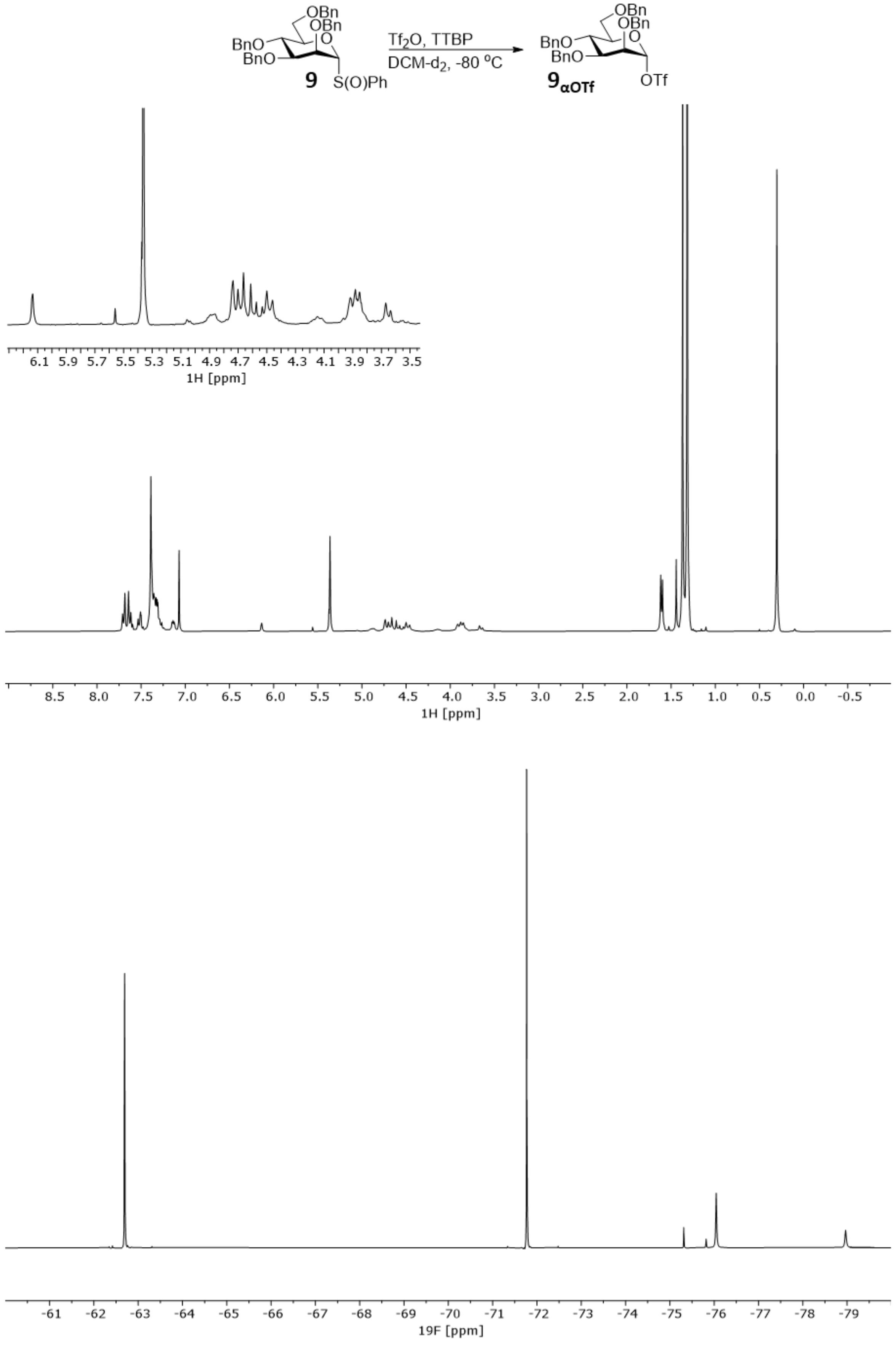

Figure S6: ^{1}H and ^{19}F activation spectra of the α-triflate from the corresponding donor. Minor decomposition was observed due to formation of benzyl triflate at δ_F = 75.2 ppm.

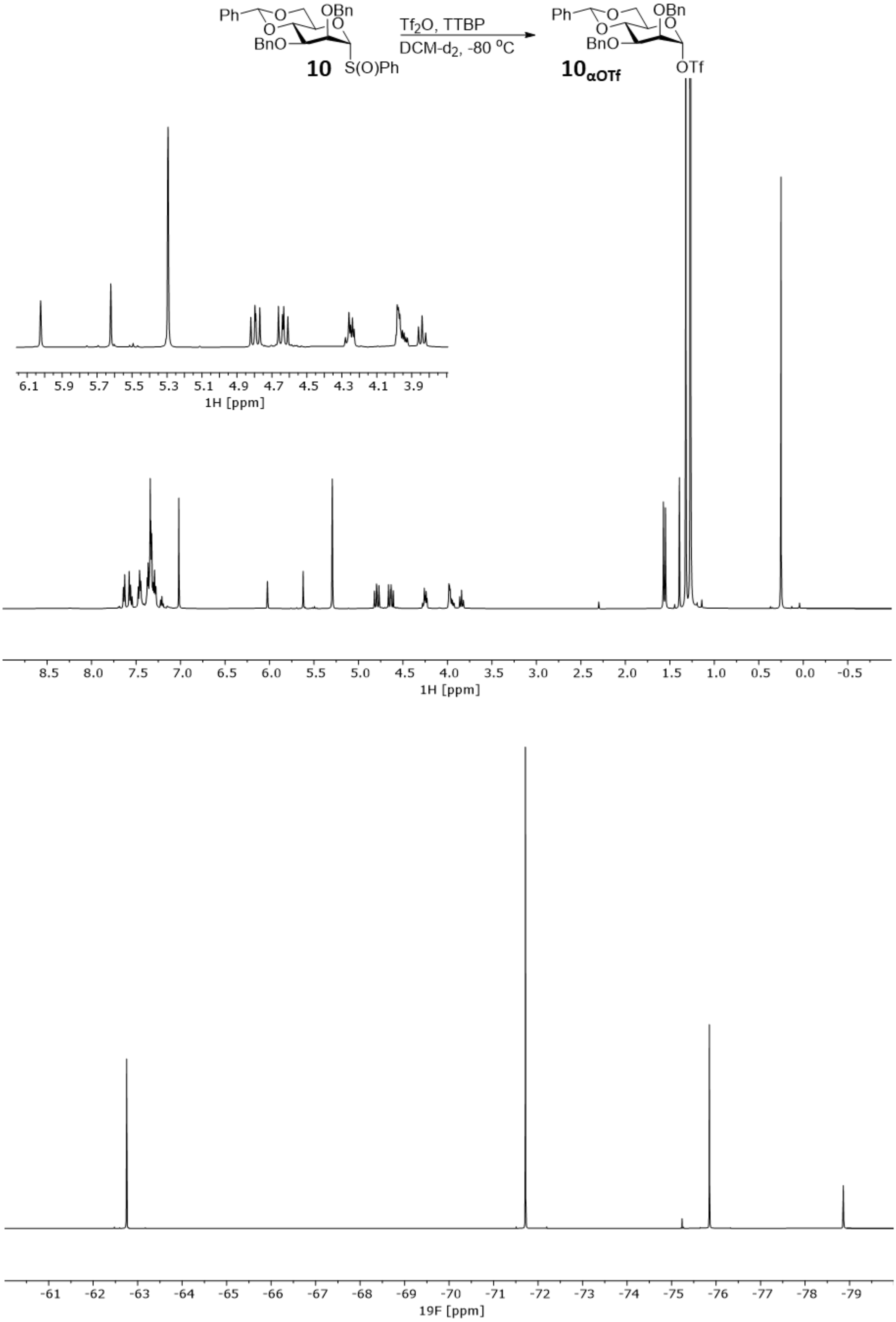

Figure S7: ^{1}H and ^{19}F activation spectra of the α-triflate from the corresponding donor. Very minor decomposition was observed due to formation of benzyl triflate at δ_F = 75.2 ppm.

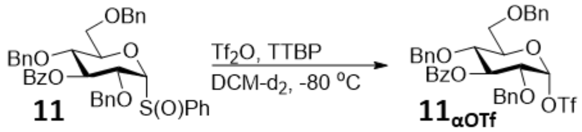

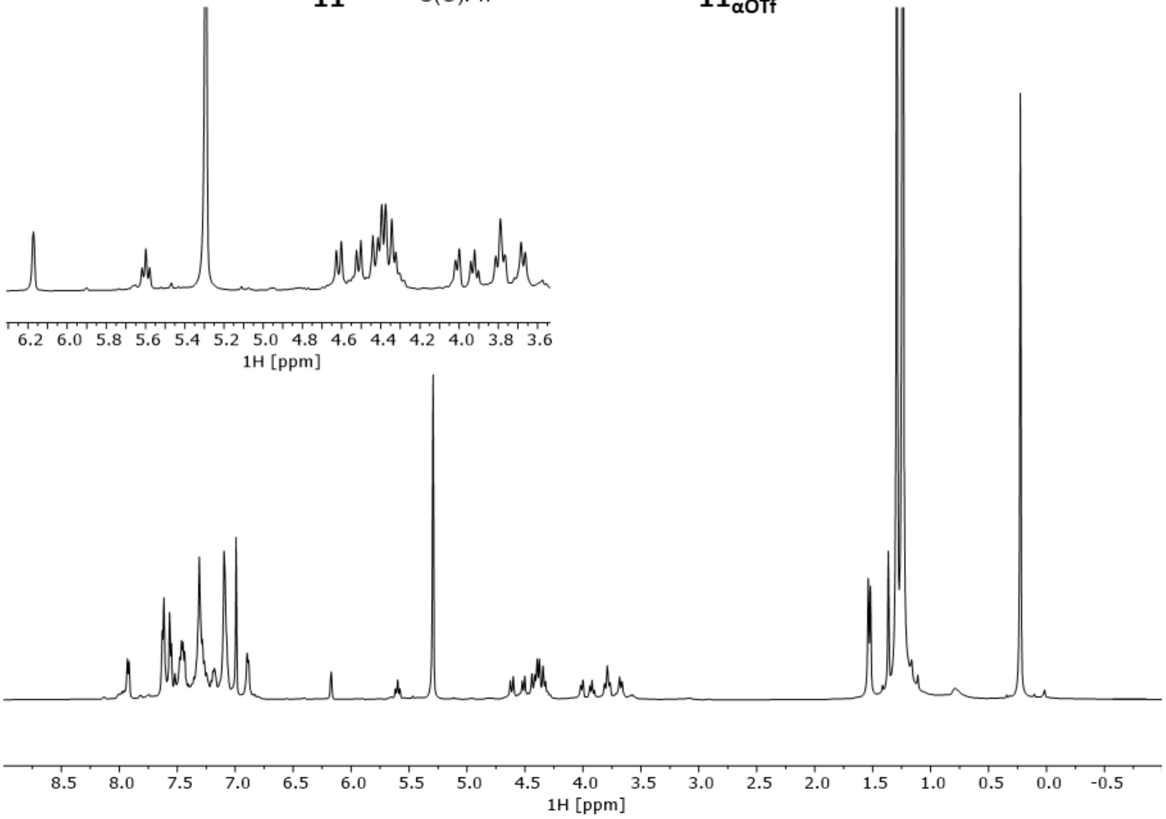

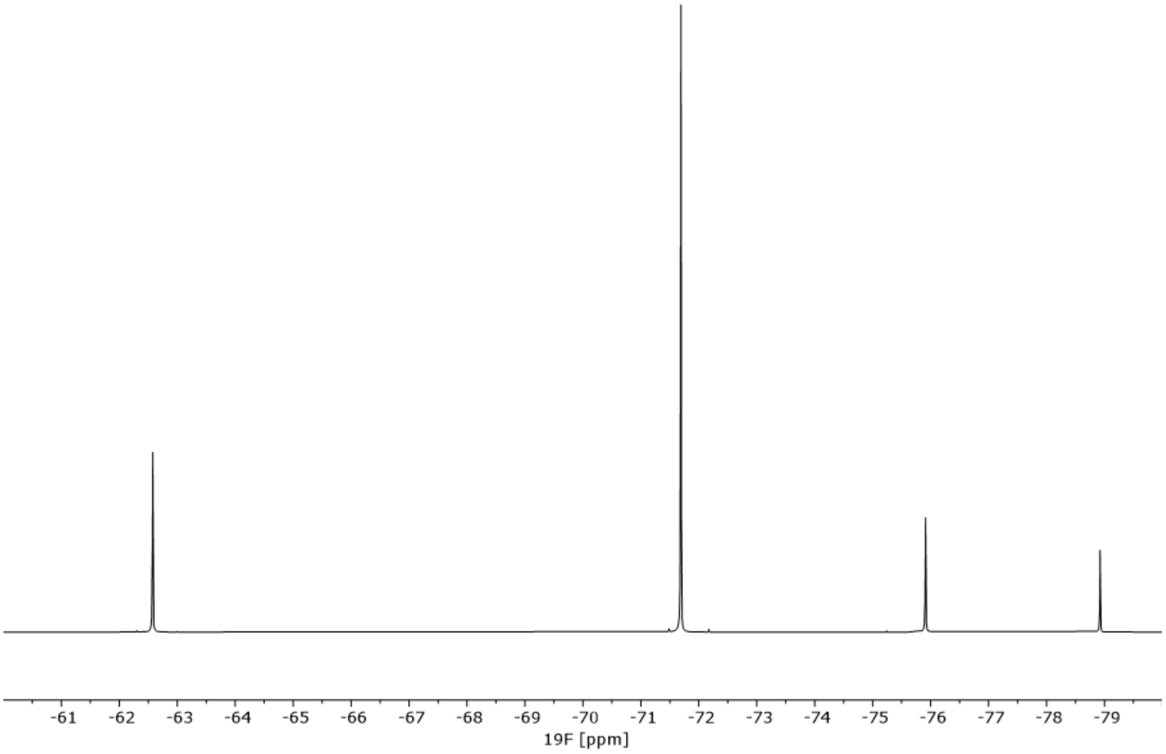

Figure S8: ^{1}H and ^{19}F activation spectra of the α-triflate from the corresponding donor.

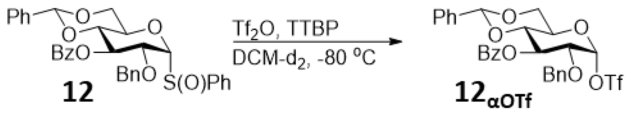

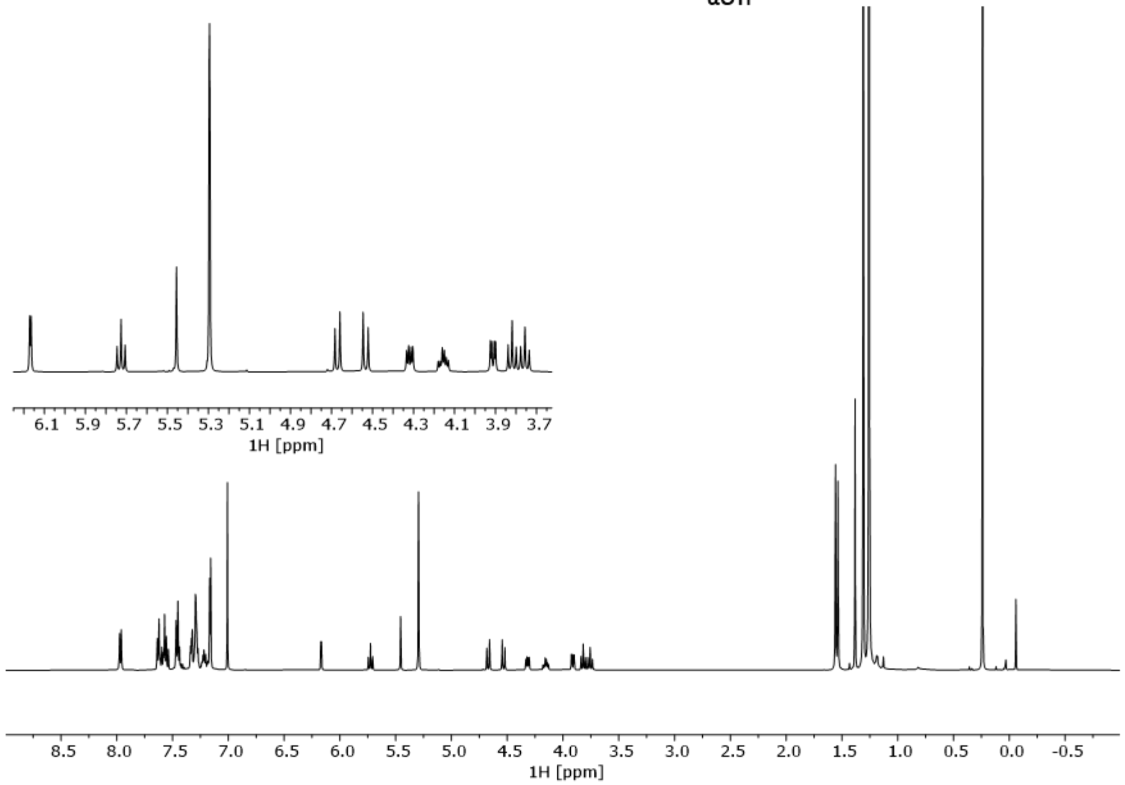

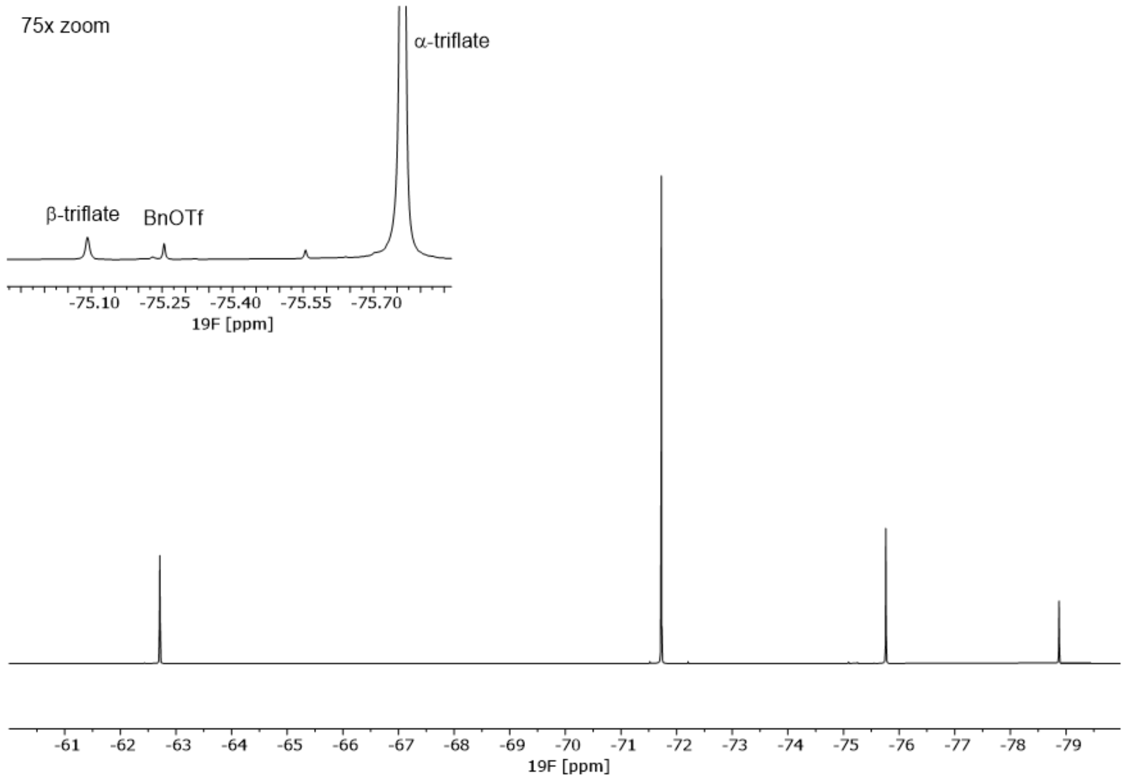

Figure S9: ^{1}H and ^{19}F activation spectra of the α-triflate from the corresponding donor. Very minor decomposition was observed due to formation of benzyl triflate at δ_F = 75.2 ppm. Additionally, zooming to the region where ^{19}F CEST NMR displayed a dip ($\delta_F \approx$ -75.1 ppm) displayed a broad, minor resonance which we assigned to the β-triflate due to its exchange with the α-triflate.

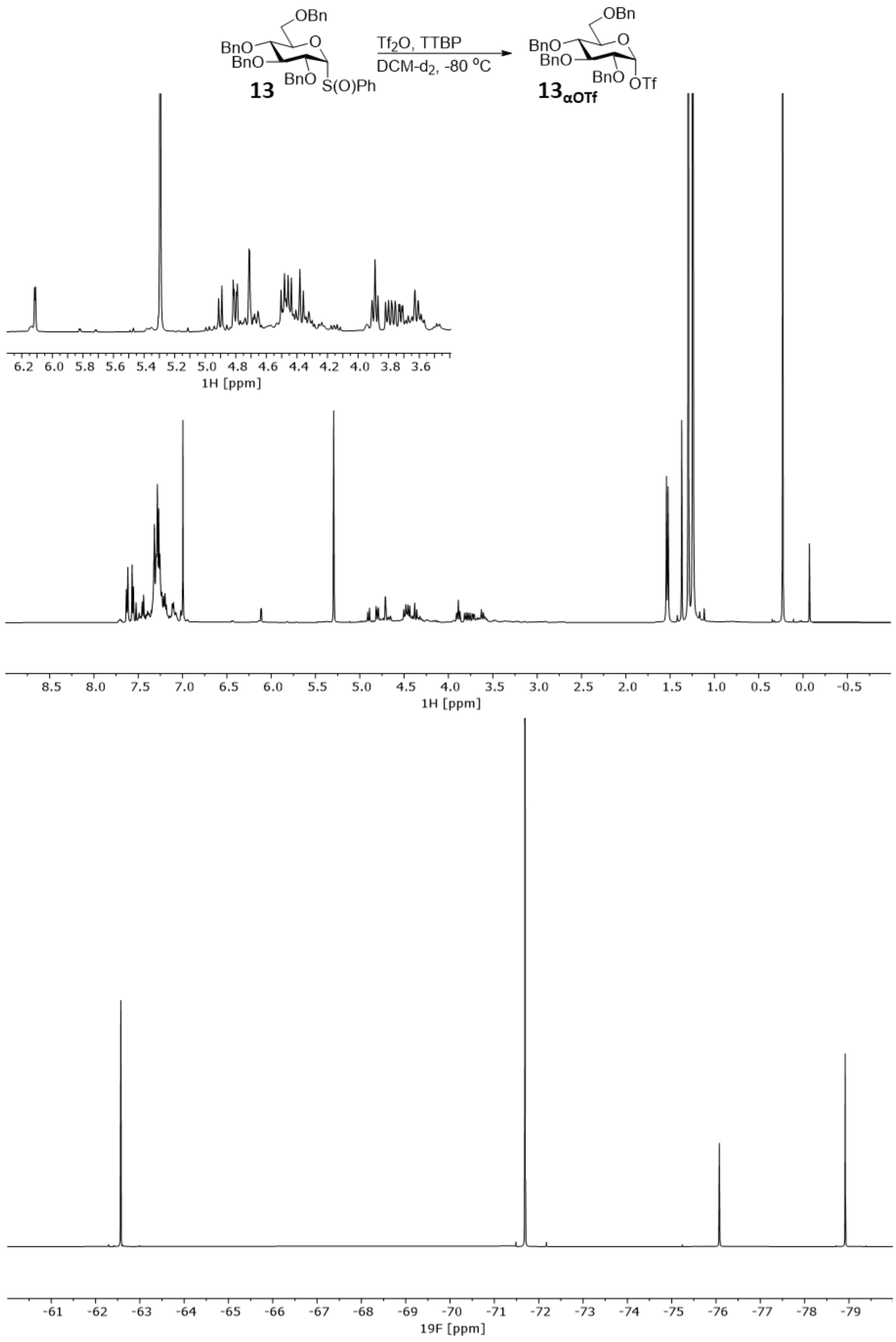

Figure S10: ^{1}H and ^{19}F activation spectra of the α-triflate from the corresponding donor.

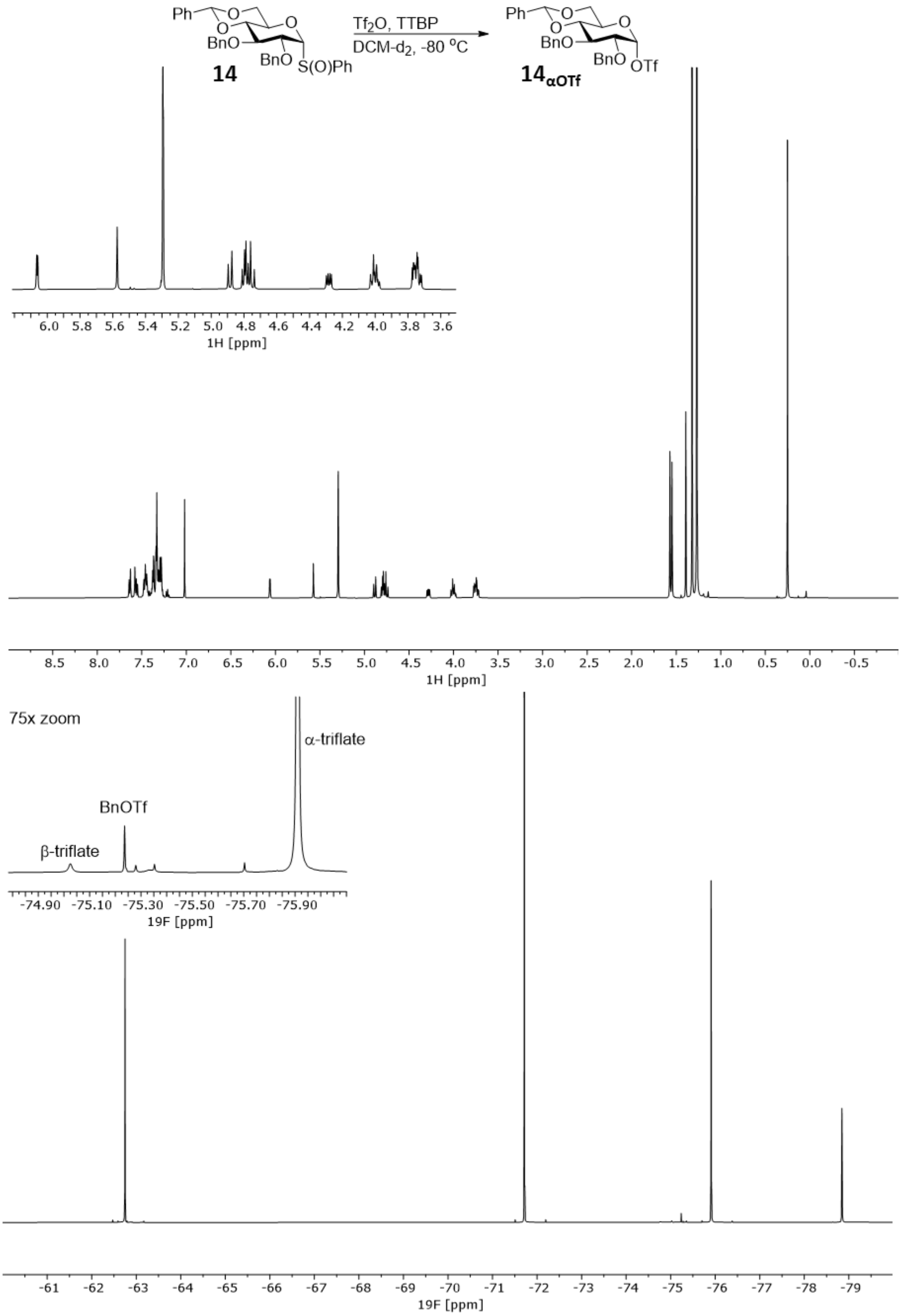

Figure S11: ^{1}H and ^{19}F activation spectra of the α-triflate from the corresponding donor. Very minor decomposition was observed due to formation of benzyl triflate at δ_F = 75.2 ppm. Additionally, zooming to the region where ^{19}F CEST NMR displayed a dip ($\delta_F \approx$ -75.1 ppm) displayed a broad, minor resonance which we assigned to the β-triflate due to its exchange with the α-triflate.

Supporting rates for triflate dependant α-triflate dissociation

Table S1: Supporting rates ($R_{\alpha \rightarrow \mathrm{OTf,\ EXSY}}$) at varying concentrations triflate anion.

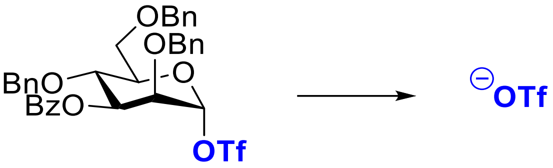

Concentration $^{-}$OTf [M]	$R_{\alpha \rightarrow \mathrm{OTf,\ EXSY}}$ [s^{-1}]
0.017	0.34 +/- 0.002
0.092	0.41 +/- 0.008
0.15	0.35 +/- 0.02

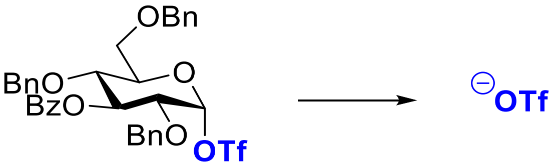

Concentration $^{-}$OTf [M]	$R_{\alpha \rightarrow \mathrm{OTf,\ EXSY}}$ [s^{-1}]
0.014	0.16 +/- 0.004
0.047	0.56 +/- 0.01
0.091	0.94 +/- 0.01
0.16	1.65 +/- 0.01

Table S2: Supporting rates ($R_{\alpha \rightarrow \mathrm{OTf,\ EXSY}}$) at varying concentrations triflate anion.

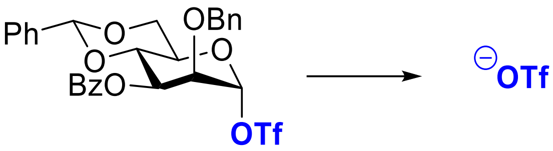

Concentration $^{-}$OTf [M]	$R_{\alpha \rightarrow \mathrm{OTf,\ EXSY}}$ [s^{-1}]
0.011	0.25 +/- 0.003
0.043	0.33 +/- 0.006
0.084	0.35 +/- 0.006
0.15	0.38 +/- 0.007

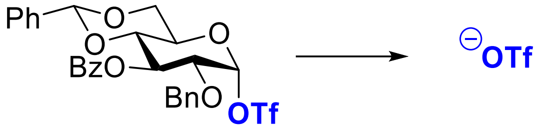

Concentration $^{-}$OTf [M]	$R_{\alpha \rightarrow \mathrm{OTf,\ EXSY}}$ [s^{-1}]
0.013	0.14 +/- 0.003
0.049	0.57 +/- 0.005
0.093	1.08 +/- 0.01
0.16	1.68 +/- 0.06

Table S3: Supporting rates ($R_{\alpha \rightarrow \mathrm{OTf,\ EXSY}}$) at varying concentrations triflate anion.

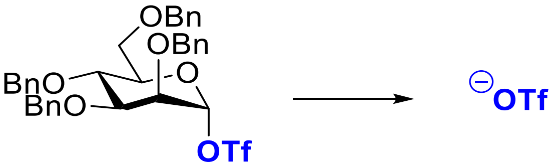

Concentration $^{-}$OTf [M]	$R_{\alpha \rightarrow \mathrm{OTf,\ EXSY}}$ [s^{-1}]
0.012	0.35 +/- 0.003
0.047	1.19 +/- 0.02
0.093	2.07 +/- 0.06
0.16	3.53 +/- 0.3

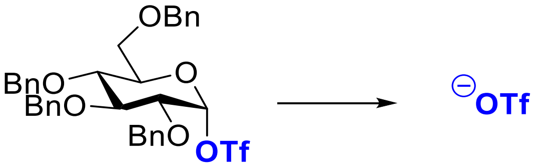

Concentration $^{-}$OTf [M]	$R_{\alpha \rightarrow \mathrm{OTf,\ EXSY}}$ [s^{-1}]
0.024	0.61 +/- 0.004
0.058	1.96 +/- 0.03
0.10	3.61 +/- 0.07
0.17	6.21 +/- 0.4

Table S4: Supporting rates ($R_{\alpha \rightarrow OTf,\ EXSY}$) at varying concentrations triflate anion.

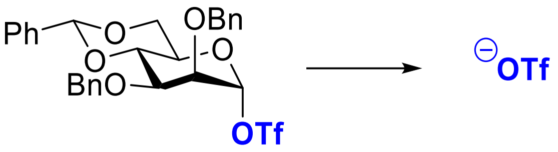

Concentration $^{-}$OTf [M]	$R_{\alpha \rightarrow OTf,\ EXSY}$ [s^{-1}]
0.015	0.10 +/- 0.002
0.049	0.41 +/- 0.003
0.098	0.79 +/- 0.01
0.16	1.25 +/- 0.03

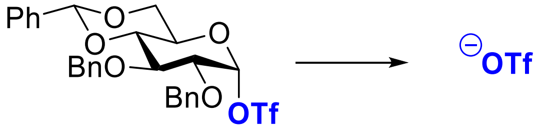

Concentration $^{-}$OTf [M]	$R_{\alpha \rightarrow OTf,\ EXSY}$ [s^{-1}]
0.012	0.17 +/- 0.008
0.047	1.11 +/- 0.007
0.10	2.40 +/- 0.04
0.19	4.20 +/- 0.09

Effect of benzyl- and methyl-2,4,6-tri-*O*-protecting groups on mannosyl dioxanium ion formation and α-triflate stability

Table S5: $R_{\alpha \rightarrow OTf,EXSY}$ for mannosyl triflates equipped with a benzoyl at C3 and either methyl or benzyl protecting groups at C2, C4, and C4, demonstrating the difference in rates.

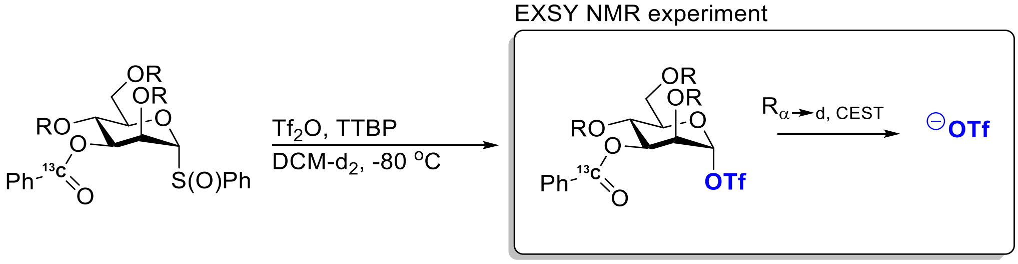

Entry	Concentration $^{-}$OTf [M]	$R_{\alpha \rightarrow OTf,\ EXSY}$ [s^{-1}] R = Me	$R_{\alpha \rightarrow OTf,\ EXSY}$ [s^{-1}] R = Bn
1	0.016		0.33 +/- 0.003
2	0.033	0.87 +/- 0.02	
3	0.092	1.06 +/- 0.02	0.41 +/- 0.01
4	0.15	1.08 +/- 0.02	0.35 +/- 0.02
5	0.24	0.96 +/- 0.05	

Table S6: $R_{\alpha \rightarrow d, CEST}$ for mannosyl triflates equipped with a benzoyl at C3 and either methyl or benzyl protecting groups at C2, C4, and C4, demonstrating the difference in rates.

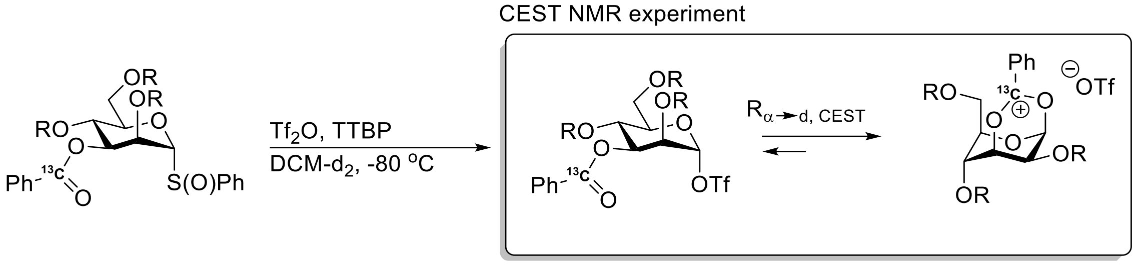

Entry	Concentration $^{-}$OTf [M]	$R_{\alpha \rightarrow d, CEST}$ [s^{-1}] R = Me	$R_{\alpha \rightarrow d, CEST}$ [s^{-1}] R = Bn
1	0.016		0.26 +/- 0.01
2	0.033	0.78 +/- 0.01	
3	0.092	0.88 +/- 0.04	0.30 +/- 0.01
4	0.15	0.74 +/- 0.04	0.34 +/- 0.01

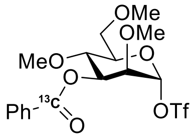

Decomposition temperature = -30 °C

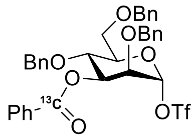

Decomposition temperature = -50 °C

Figure S12: Decomposition temperatures for C2, C4, and C6 methyl and benzyl protected triflates equipped with a benzoyl at C3.

Supporting CEST profiles overlaid with 1D NMR spectra

This section displays CEST profiles for glycosyl α-triflates (**$7_{αOTf}$ - $14_{αOTf}$**) studied in this work. The CEST profiles are recorded on the ^{1}H, ^{19}F, and when possible ^{13}C nuclei. Whilst, for example, the mannosyl dioxanium ion **7_d^{13C}** displays a clear CEST dip at $δ_C$ = 177 ppm, recording a ^{1}H CEST profile for the same molecule only leads to a minor shoulder on top of the major CEST dip resulting from direct saturation of the α-triflate itself. It should be noted that the shape of the CEST profiles and their dip intensities is highly dependent on the observed nucleus (^{1}H, ^{13}C, or ^{19}F) resonance frequency and the frequency difference between the two sites; the exchange rates between exchanging species; the field strength of the NMR; saturation time; and saturation width. For example, a narrow saturation width (15 Hz vs 40 Hz) will yield CEST profiles with higher resolution between the observed dips, although, it also reduced the dip intensity.[10] As a consequence, individual CEST profiles could not be easily overlaid across different molecules or within the same molecule on different nuclei. The main purpose of this section is to demonstrate CEST as tool to detect low-populated intermediates by looking at their CEST profiles and explain the meaning of each CEST dip observed in the profile. Raw data about the CEST spectra are provided in the 'Data' section at the end of this supporting information.

^{13}C

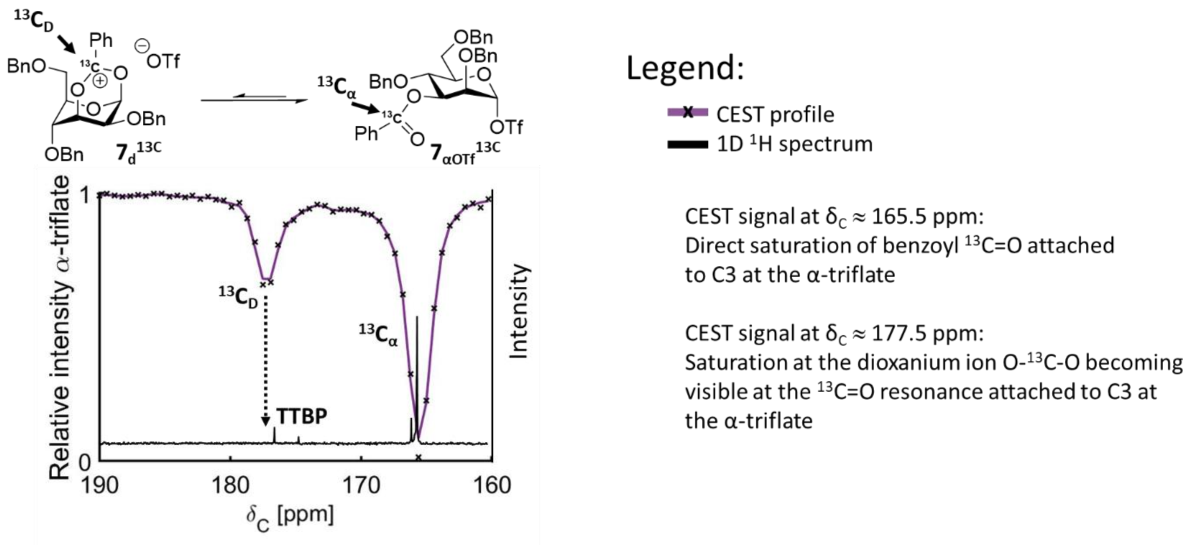

Figure S13: ^{13}C CEST profile overlaid with the 1D ^{13}C NMR spectrum for the corresponding glycosyl α-triflate displaying evidence for a dioxanium ion in ^{13}C CEST NMR spectroscopy.

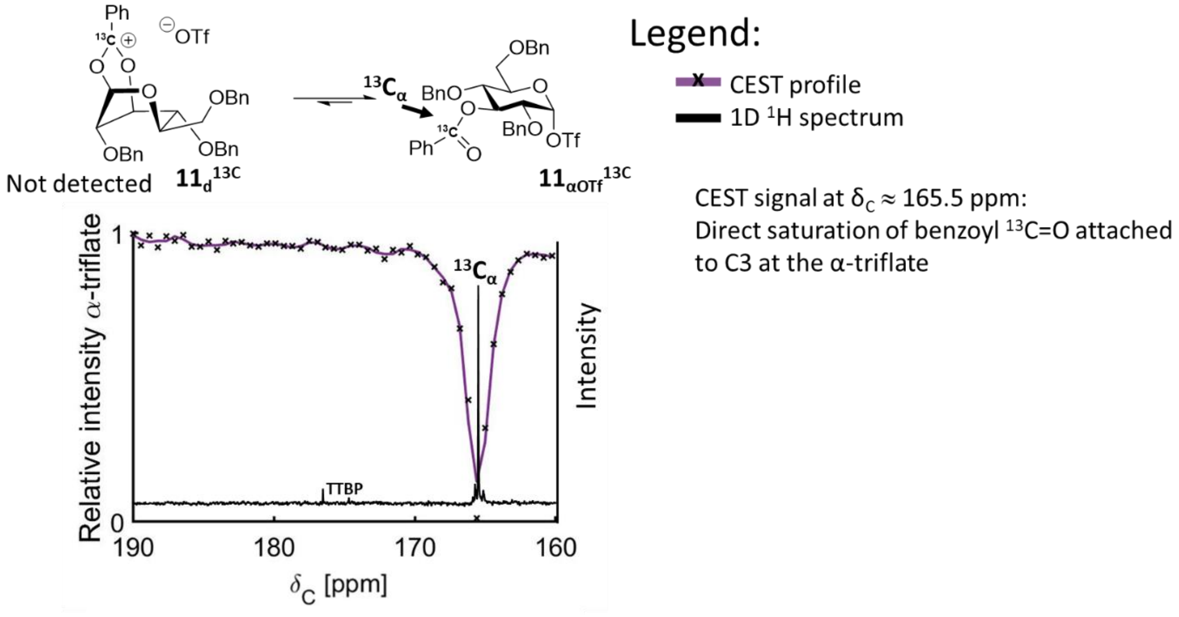

Figure S14: ^{13}C CEST profile overlaid with the 1D ^{13}C NMR spectrum for the corresponding glycosyl α-triflate, displaying no evidence for a dioxanium ion in ^{13}C CEST and 1D ^{13}C NMR spectroscopy.

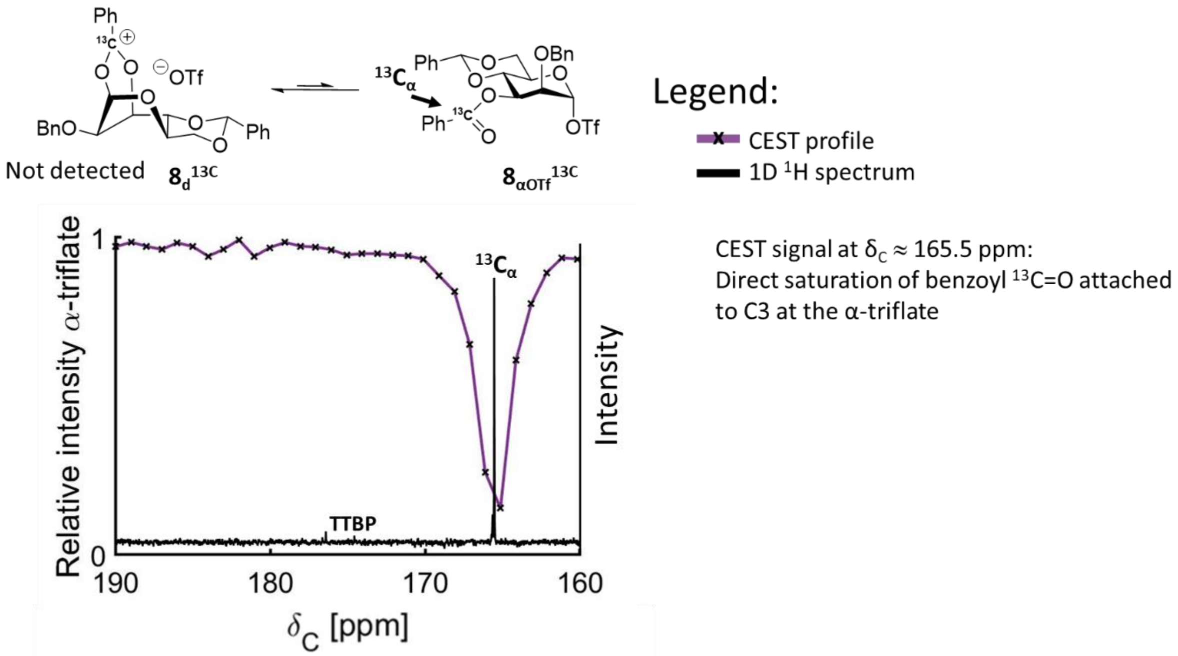

Figure S15: ^{13}C CEST profile overlaid with the 1D ^{13}C NMR spectrum for the corresponding glycosyl α-triflate, displaying no evidence for a dioxanium ion in ^{13}C CEST and 1D ^{13}C NMR spectroscopy.

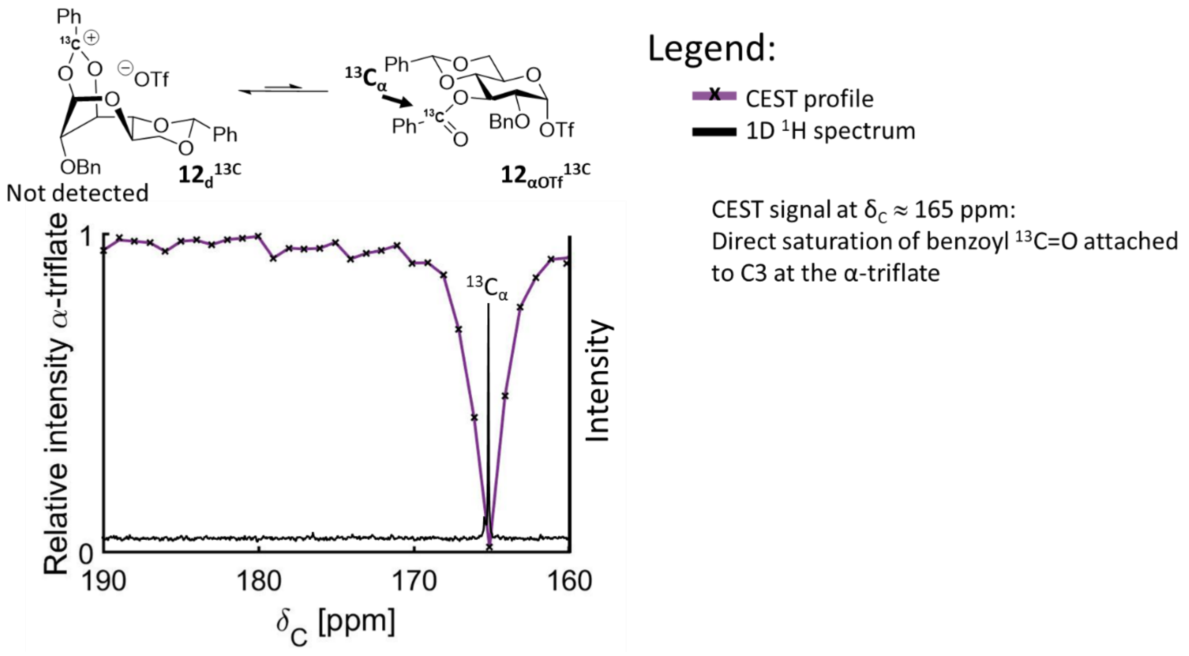

Figure S16: ^{13}C CEST profile overlaid with the 1D ^{13}C NMR spectrum for the corresponding glycosyl α-triflate, displaying no evidence for a dioxanium ion in ^{13}C CEST and 1D ^{13}C NMR spectroscopy.

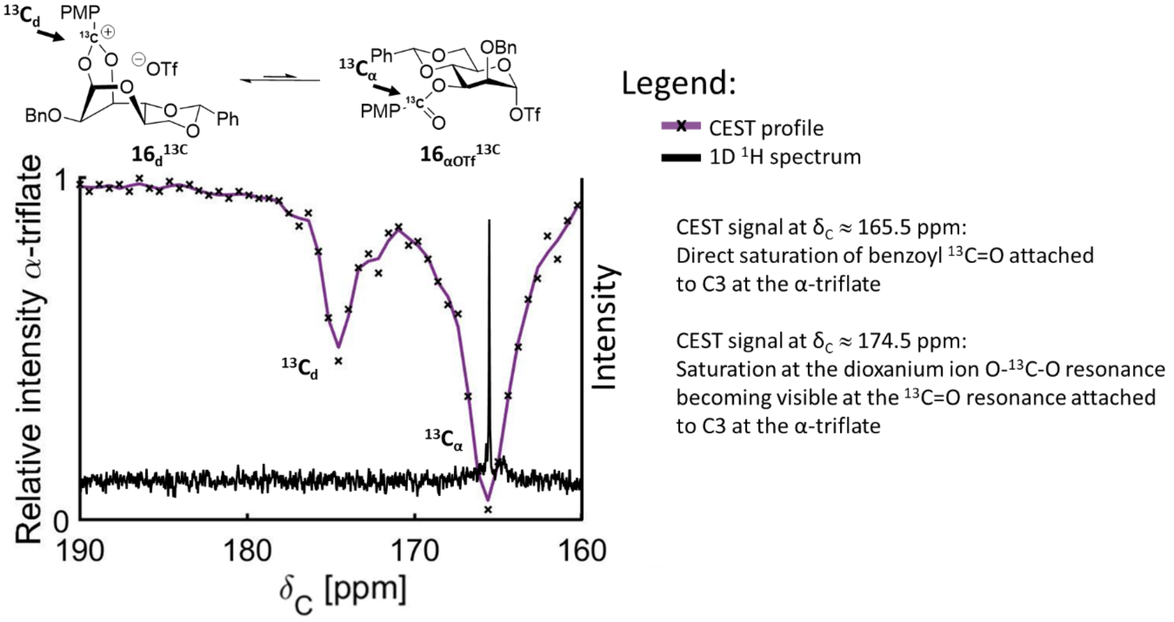

Figure S17: ^{13}C CEST profile overlaid with the 1D ^{13}C NMR spectrum for the corresponding glycosyl α-triflate, displaying evidence for a dioxanium ion only in ^{13}C CEST NMR spectroscopy.

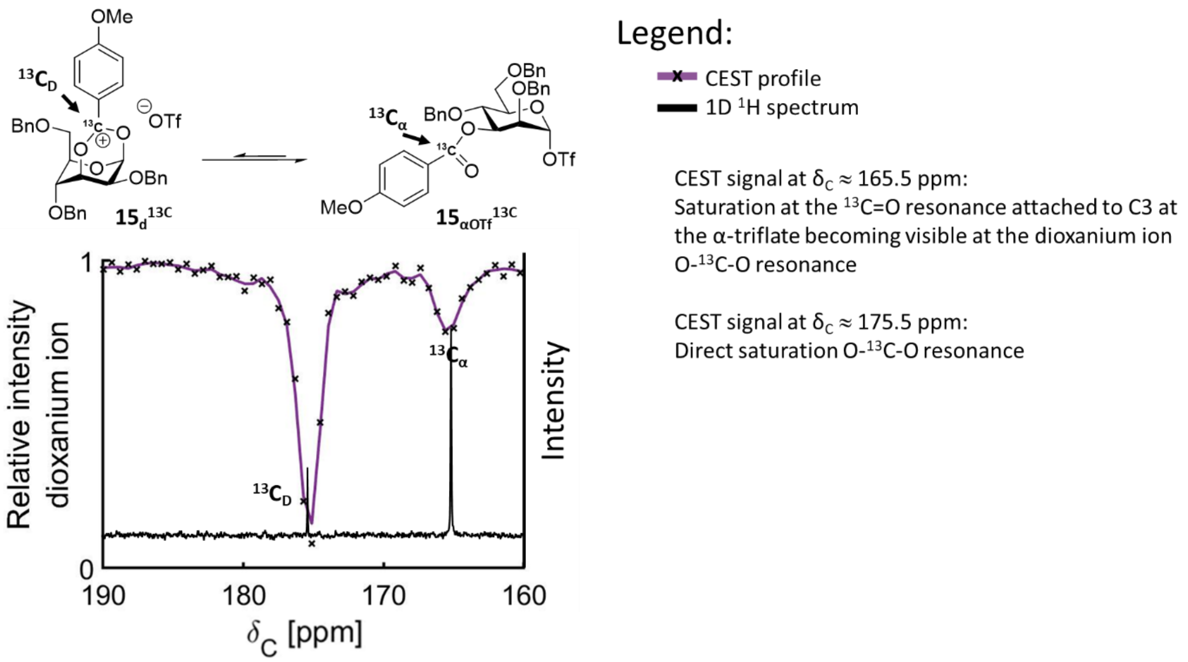

Figure S18: ^{13}C CEST profile overlaid with the 1D ^{13}C NMR spectrum for the corresponding glycosyl α-triflate, displaying evidence for a dioxanium ion in ^{13}C CEST and 1D ^{13}C NMR spectroscopy.

^{19}F

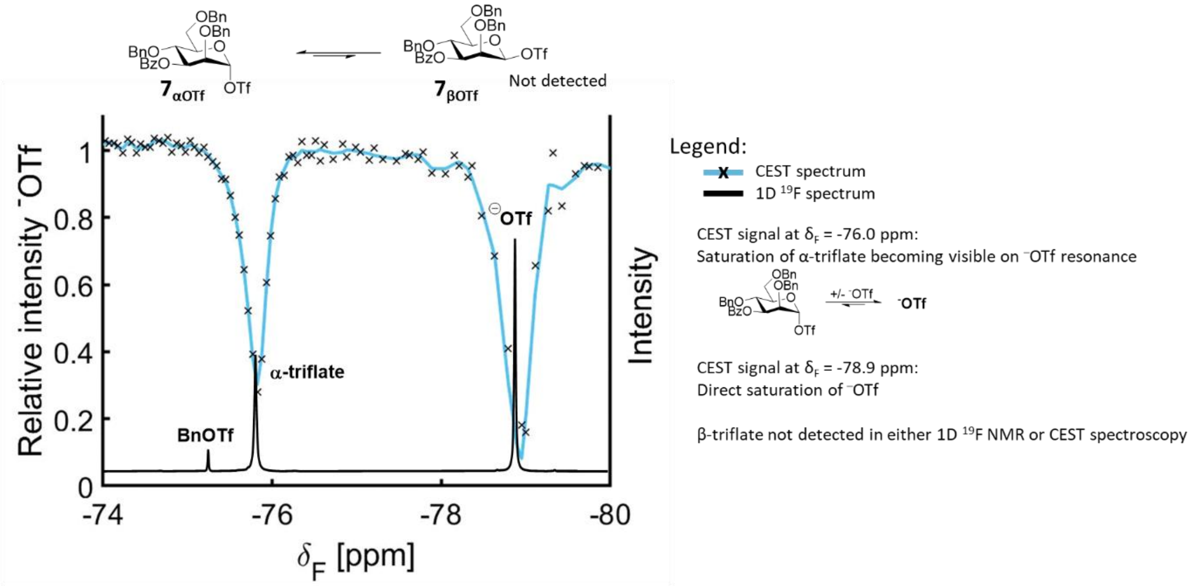

Figure S19: ^{19}F CEST profile overlaid with the 1D ^{19}F NMR spectrum for the corresponding glycosyl α-triflate, not displaying evidence for a β-triflate.

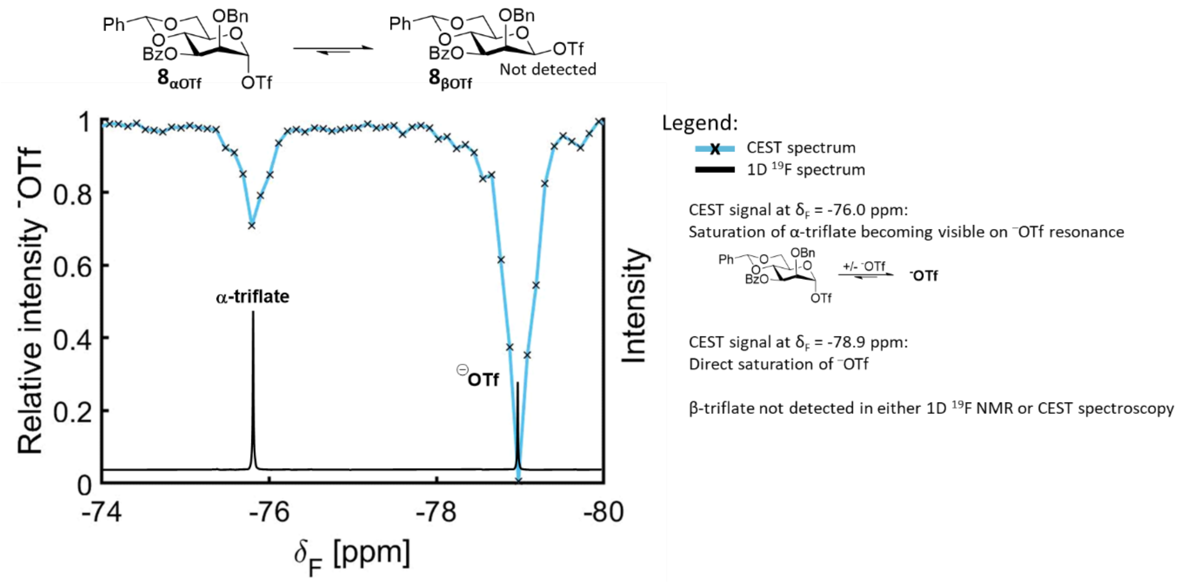

Figure S20: ^{19}F CEST profile overlaid with the 1D ^{19}F NMR spectrum for the corresponding glycosyl α-triflate, not displaying evidence for a β-triflate.

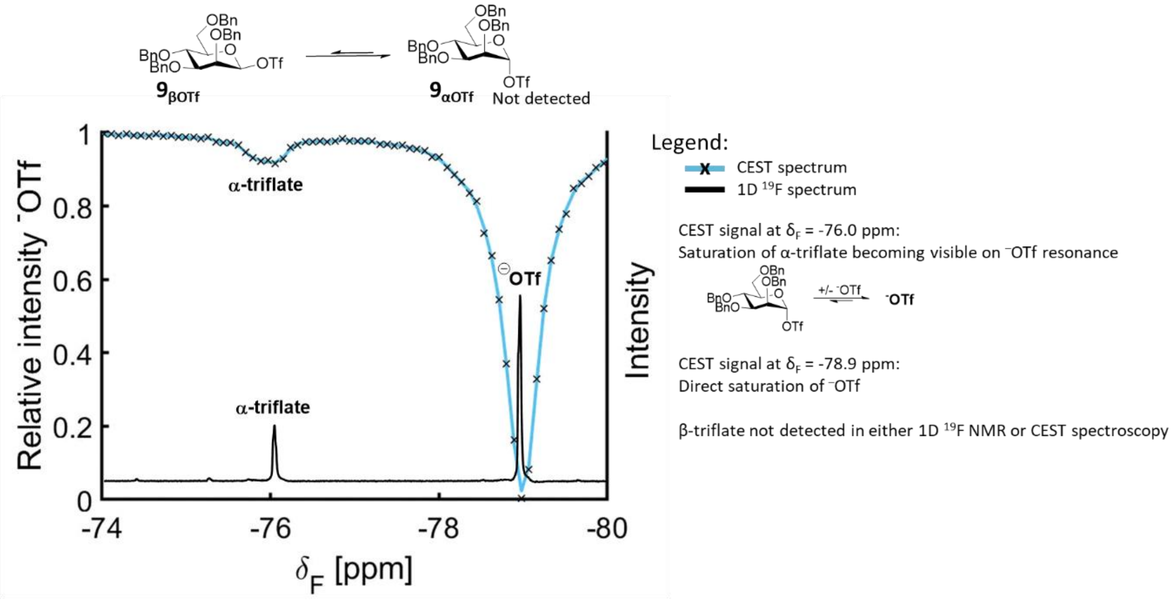

Figure S21: ^{19}F CEST profile overlaid with the 1D ^{19}F NMR spectrum for the corresponding glycosyl α-triflate, not displaying evidence for a β-triflate.

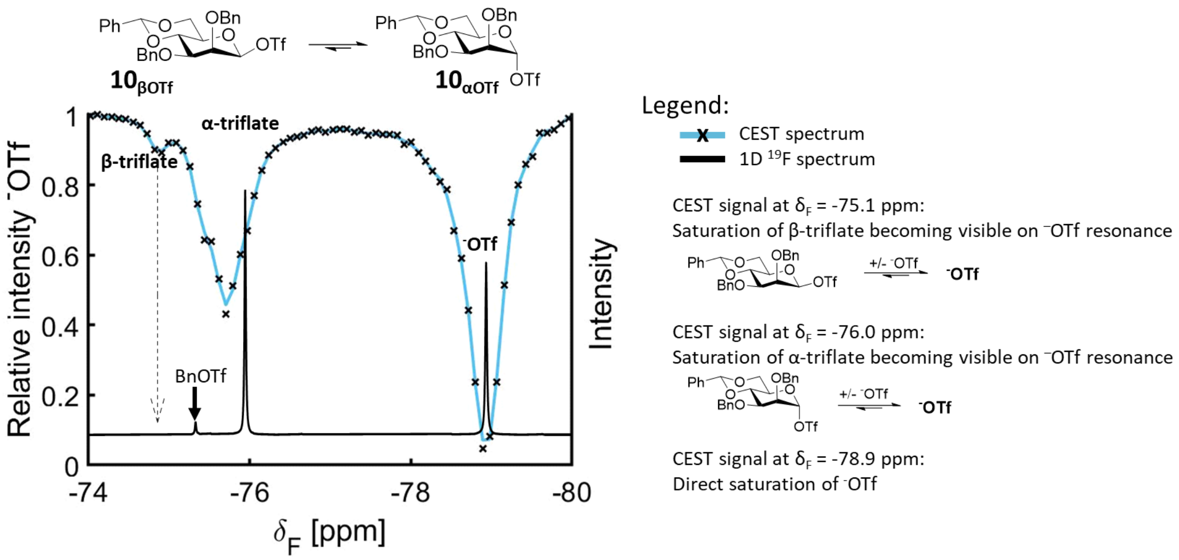

Figure S22: ^{19}F CEST profile overlaid with the 1D ^{19}F NMR spectrum for the corresponding glycosyl α-triflate, displaying evidence for a β-triflate.

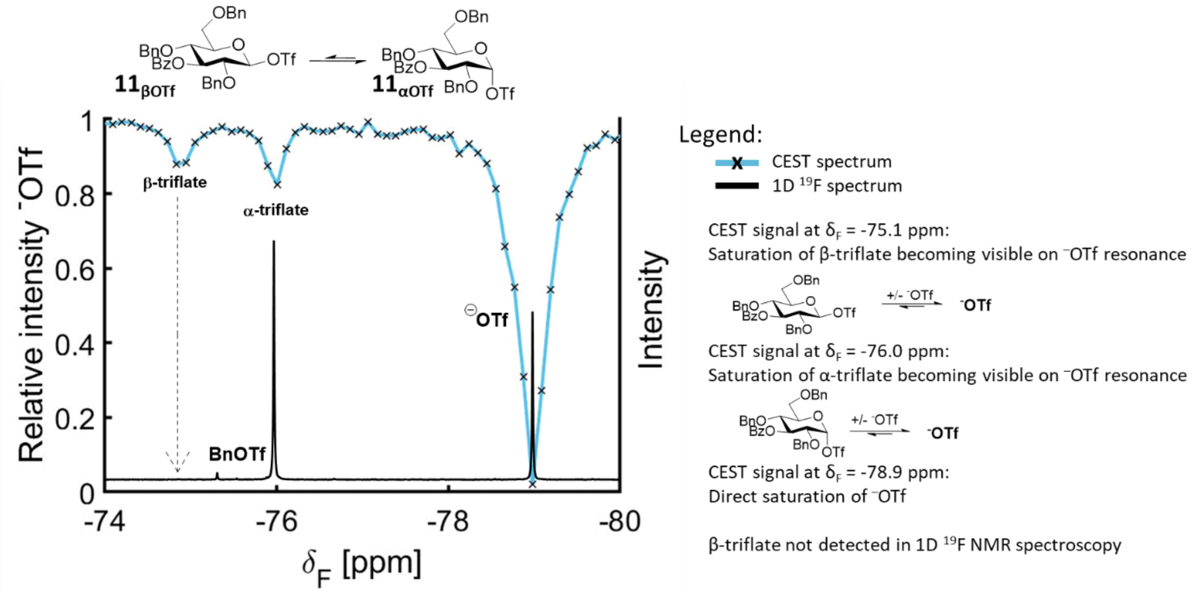

Figure S23: ^{19}F CEST profile overlaid with the 1D ^{19}F NMR spectrum for the corresponding glycosyl α-triflate, displaying evidence for a β-triflate.

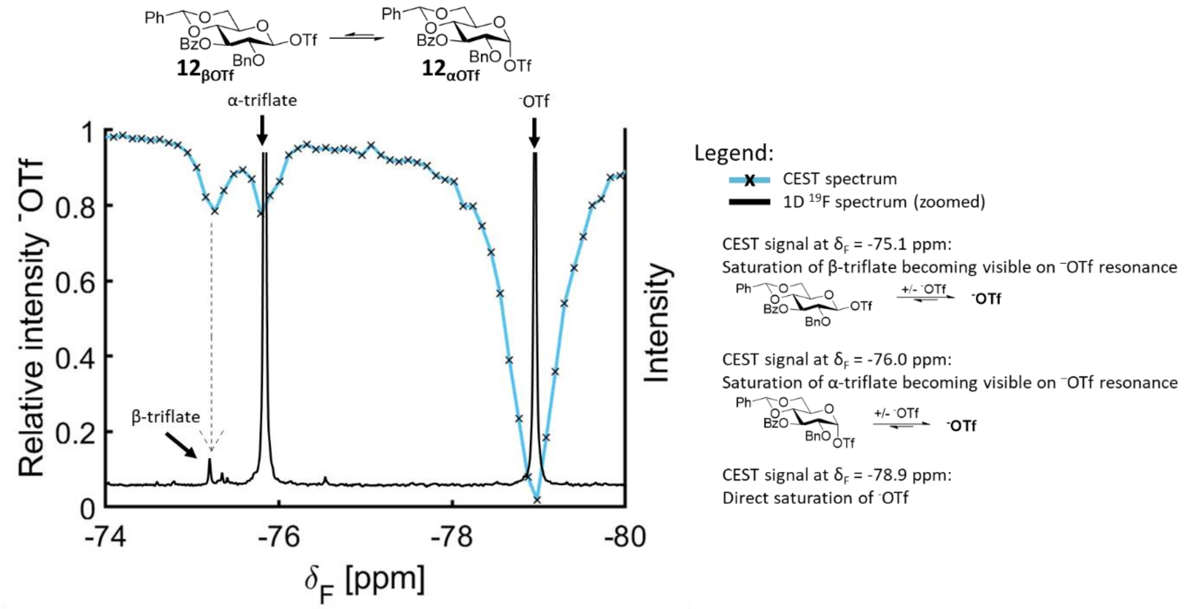

Figure S24: ^{19}F CEST profile overlaid with the 1D ^{19}F NMR spectrum for the corresponding glycosyl α-triflate, displaying evidence for a β-triflate both in ^{19}F CEST and in 1D ^{19}F NMR spectroscopy.

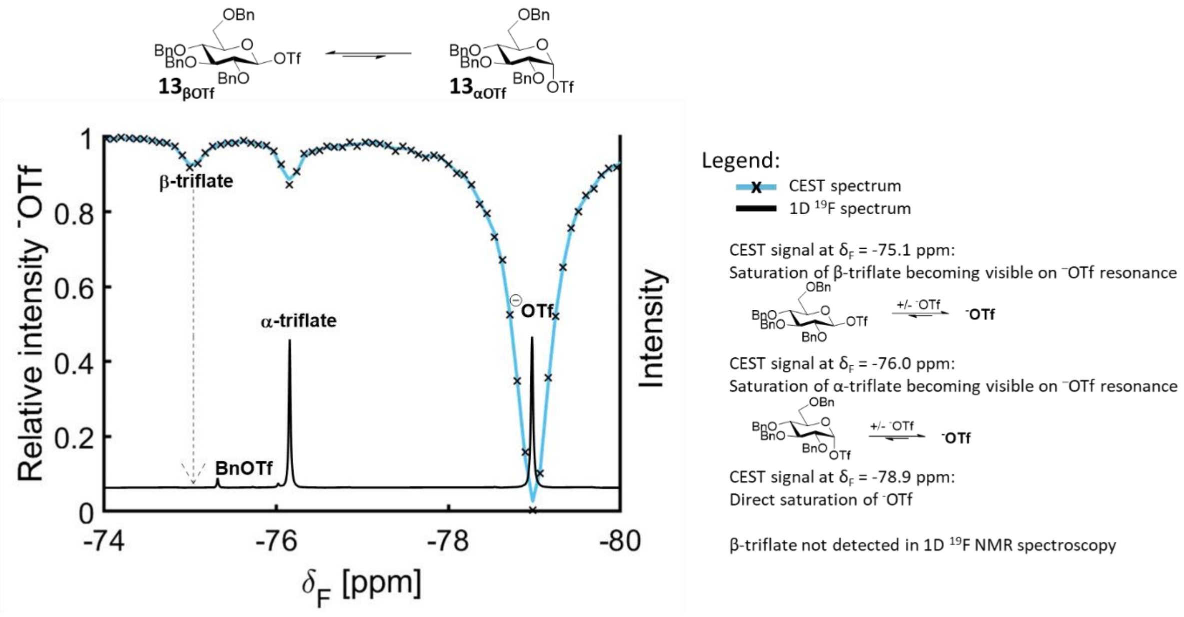

Figure S25: ^{19}F CEST profile overlaid with the 1D ^{19}F NMR spectrum for the corresponding glycosyl α-triflate, displaying evidence for a β-triflate.

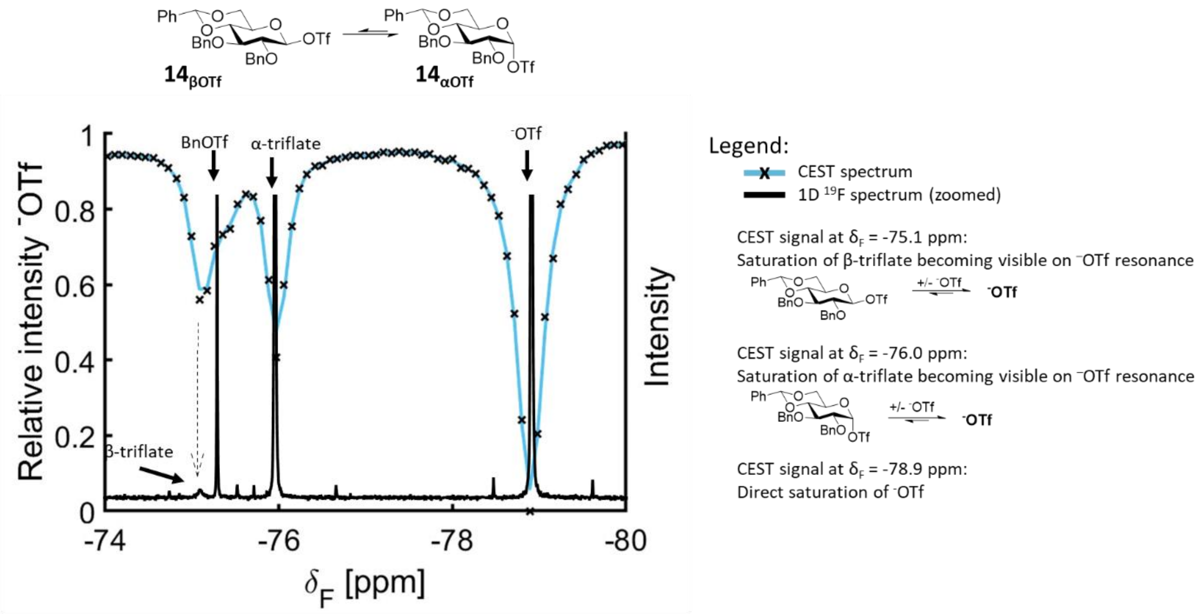

Figure S26: ^{19}F CEST profile overlaid with the 1D ^{19}F NMR spectrum for the corresponding glycosyl α-triflate, displaying evidence for a β-triflate both in ^{19}F CEST and in 1D ^{19}F NMR spectroscopy.

^{1}H

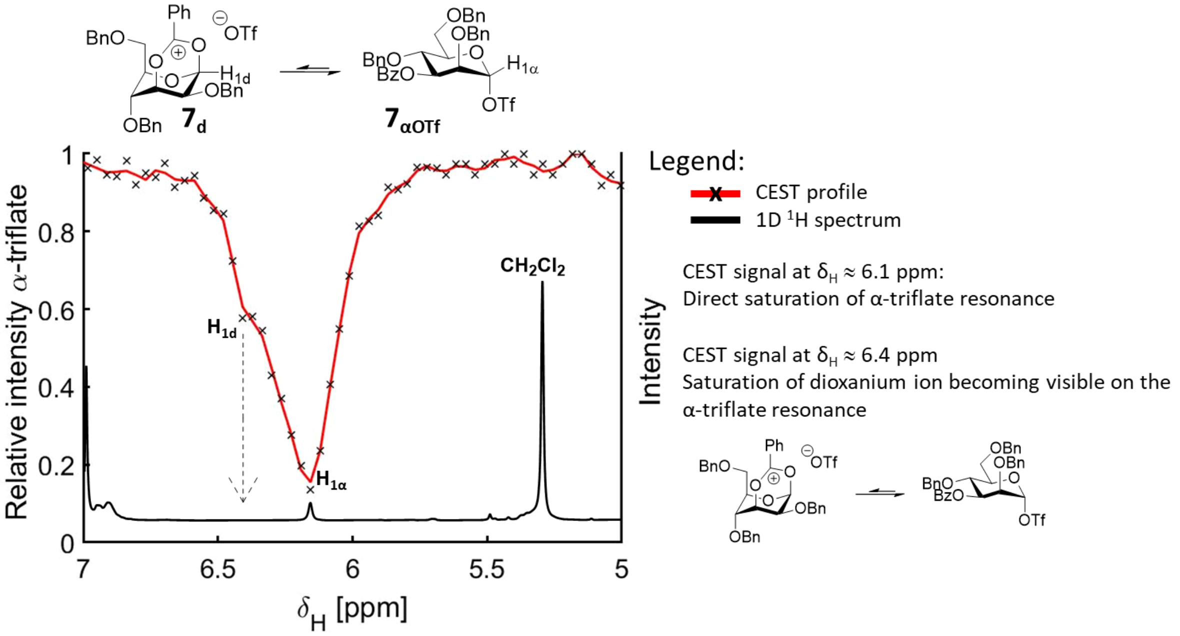

Figure S27: ^{1}H CEST profile overlaid with the 1D ^{1}H NMR spectrum for the corresponding glycosyl α-triflate, displaying evidence for a dioxanium ion in ^{1}H CEST but no evidence for a β-triflate, in 1D ^{1}H NMR spectroscopy no evidence of neither intermediate is presented.

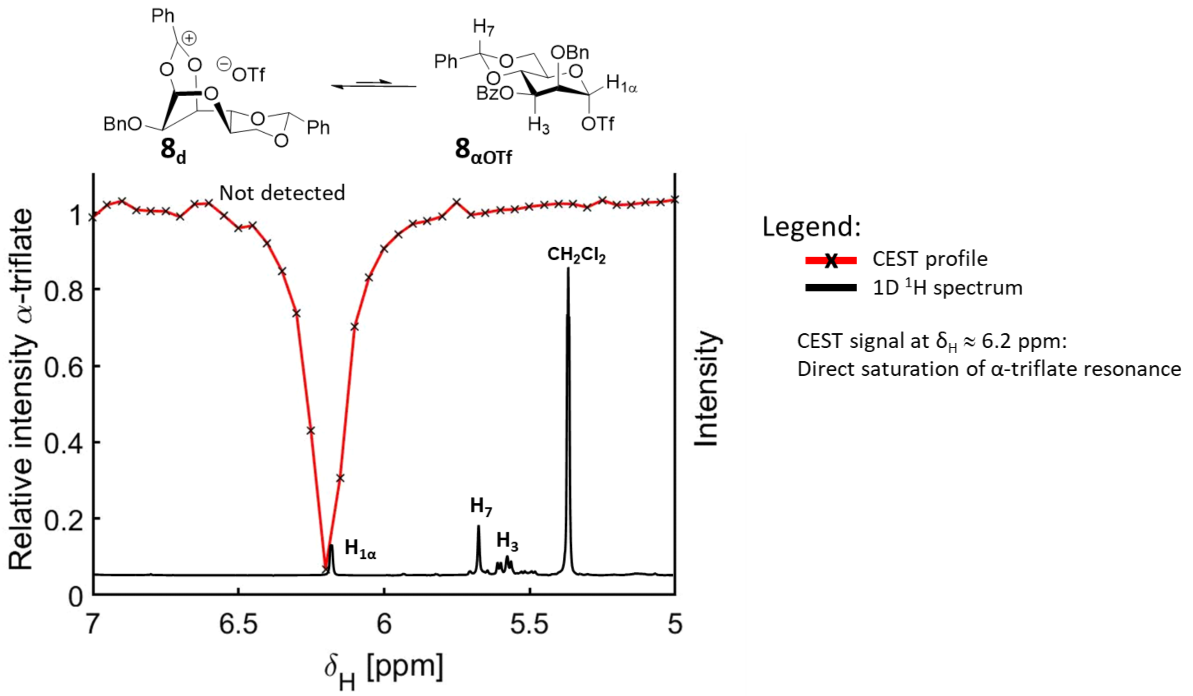

Figure S28: ^{1}H CEST profile overlaid with the 1D ^{1}H NMR spectrum for the corresponding glycosyl α-triflate, displaying no evidence for a dioxanium ion nor a β-triflate in both ^{1}H CEST and 1D ^{1}H NMR spectroscopy.

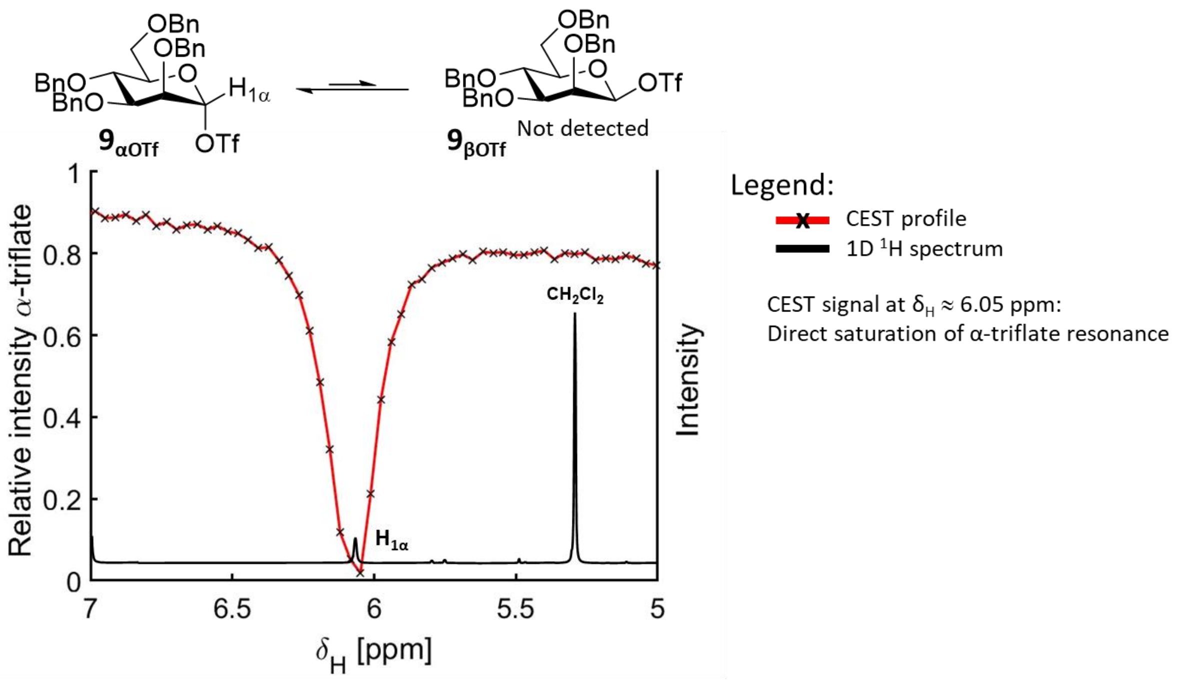

Figure S29: ^{1}H CEST profile overlaid with the 1D ^{1}H NMR spectrum for the corresponding glycosyl α-triflate, displaying no evidence for a β-triflate in both ^{1}H CEST and 1D ^{1}H NMR spectroscopy.

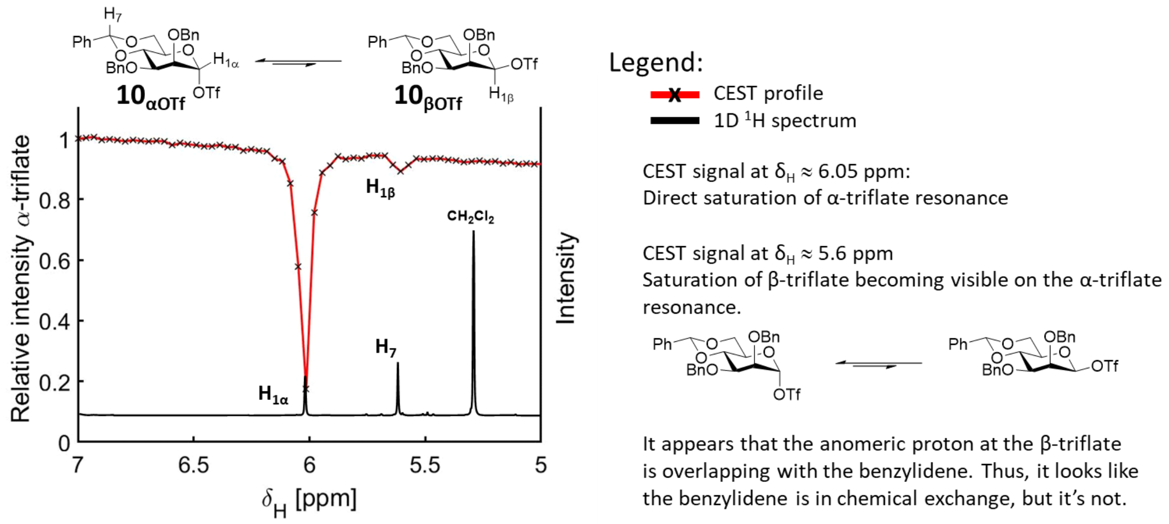

Figure S30: ^{1}H CEST profile overlaid with the 1D ^{1}H NMR spectrum for the corresponding glycosyl α-triflate, displaying evidence for a β-triflate only in ^{1}H CEST NMR spectroscopy.

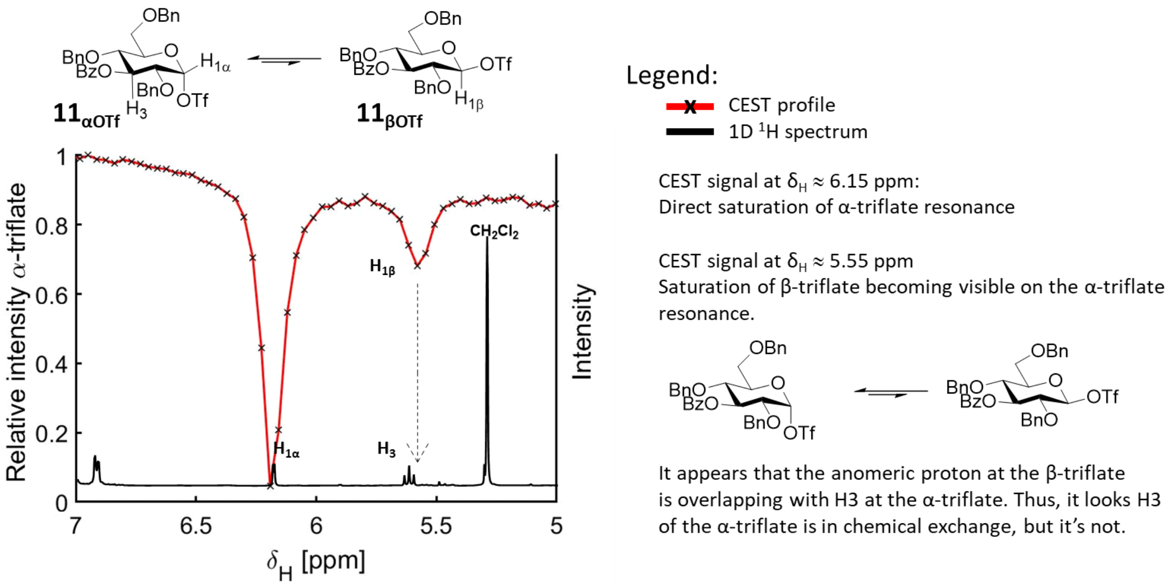

Figure S31: ^{1}H CEST profile overlaid with the 1D ^{1}H NMR spectrum for the corresponding glycosyl α-triflate, displaying evidence for a β-triflate only in ^{1}H CEST NMR spectroscopy.

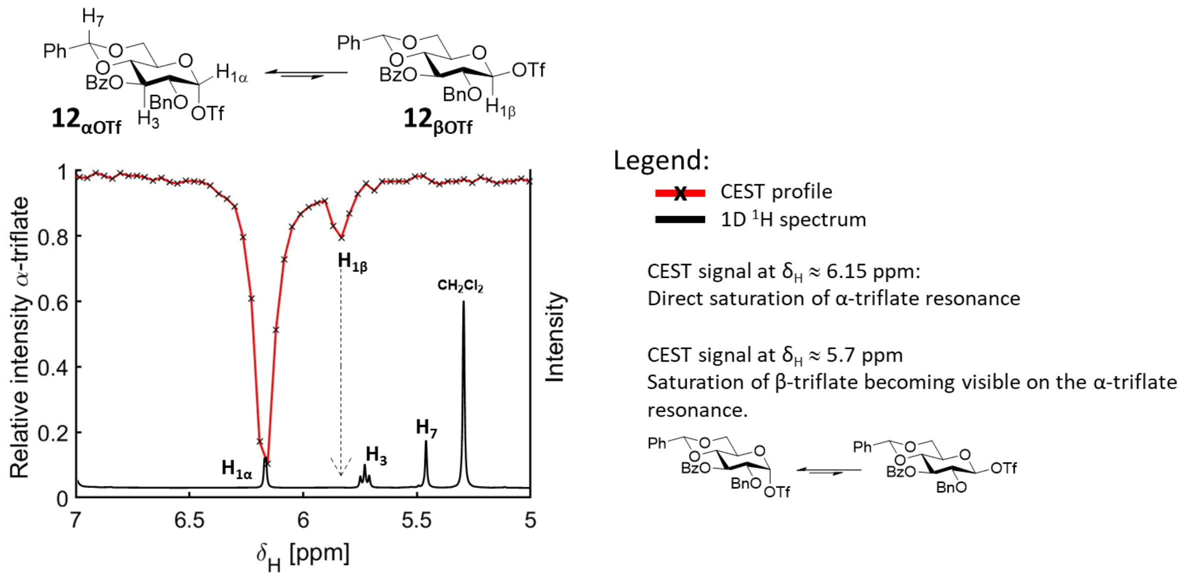

Figure S32: ^{1}H CEST profile overlaid with the 1D ^{1}H NMR spectrum for the corresponding glycosyl α-triflate, displaying evidence for a β-triflate only in ^{1}H CEST NMR spectroscopy.

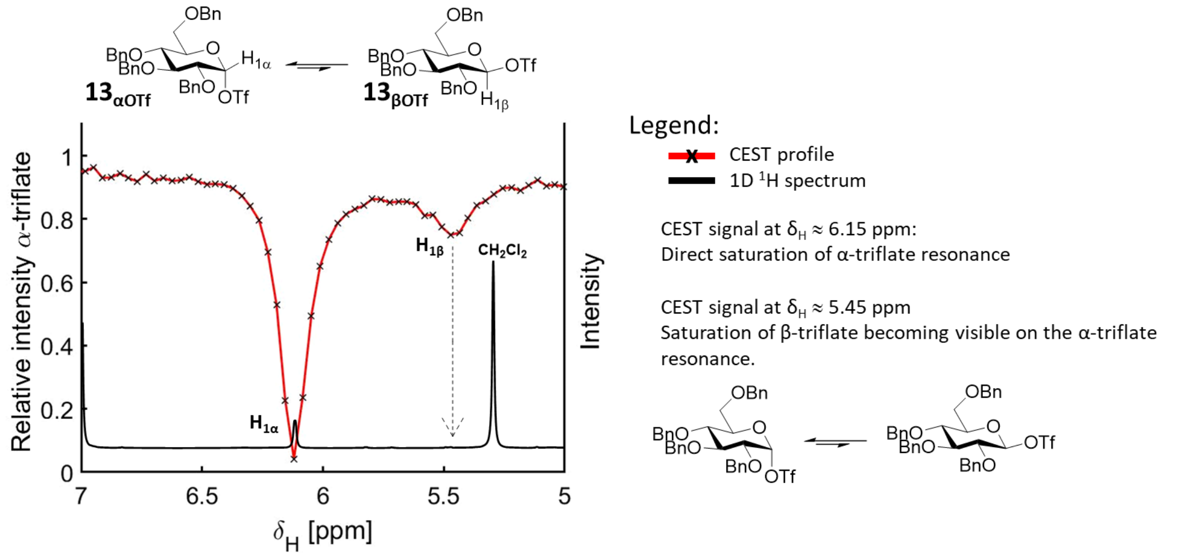

Figure S33: ^{1}H CEST profile overlaid with the 1D ^{1}H NMR spectrum for the corresponding glycosyl α-triflate, displaying evidence for a β-triflate only in ^{1}H CEST NMR spectroscopy.

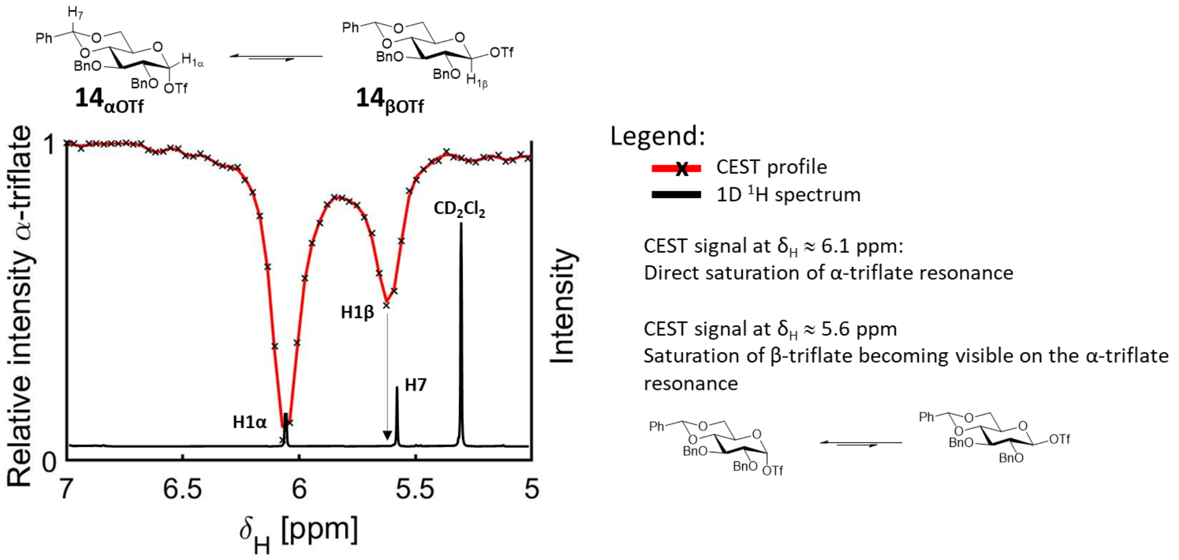

Figure S34: ^{1}H CEST profile overlaid with the 1D ^{1}H NMR spectrum for the corresponding glycosyl α-triflate, displaying evidence for a β-triflate only in ^{1}H CEST NMR spectroscopy.

Computed NMR Chemical Shifts of 8_d, $8_{\alpha OTf}$, 16_d and $16_{\alpha OTf}$

NMR chemical shifts for the benzylidene mannoses with the benzoyl and PMP protecting groups were calculated in order to provide additional support for a tricyclic dioxanium intermediate (**8**$_d$ and **16**$_d$). Geometries of the complete molecules were optimized using B3LYP/6-21+G**. ^{13}C NMR chemical shifts were computed relative to TMS using the Gauge Including Atomic Orbitals (GIAO) formalism with a B3LYP/6-31+g(2df,2pd) functional and basis set. Dichloromethane solvation effects were simulated using CPCM. All calculations were performed using Gaussian 16.

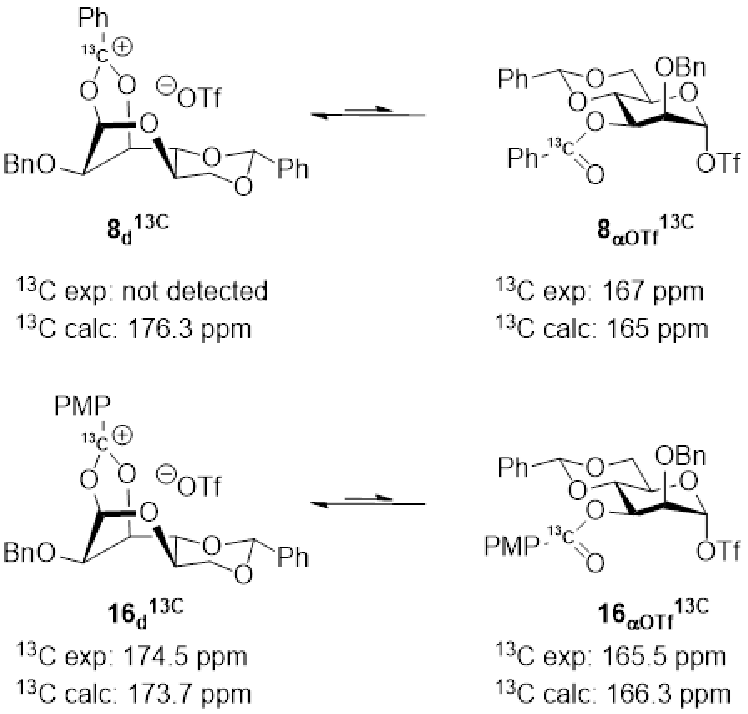

Figure S35: Experimental and calculated chemical shifts found for the acyl carbonyl and dioxanium ion bridge carbon for molecules 8_d^{13C}, $8_{\alpha OTf}^{13C}$, 16_d^{13C}, and $16_{\alpha OTf}^{13C}$.

CEST kinetic experiments on 3-*O*-(4-methoxybenzoyl-α- ^{13}C)-4,6-*O*-benzylidene-2-*O*-Bn mannosyl α-triflate

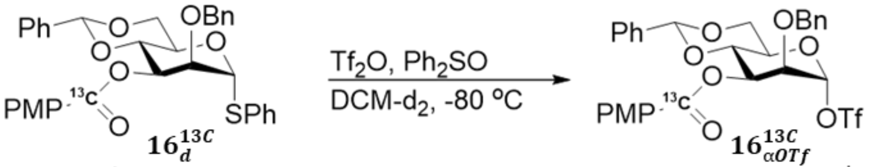

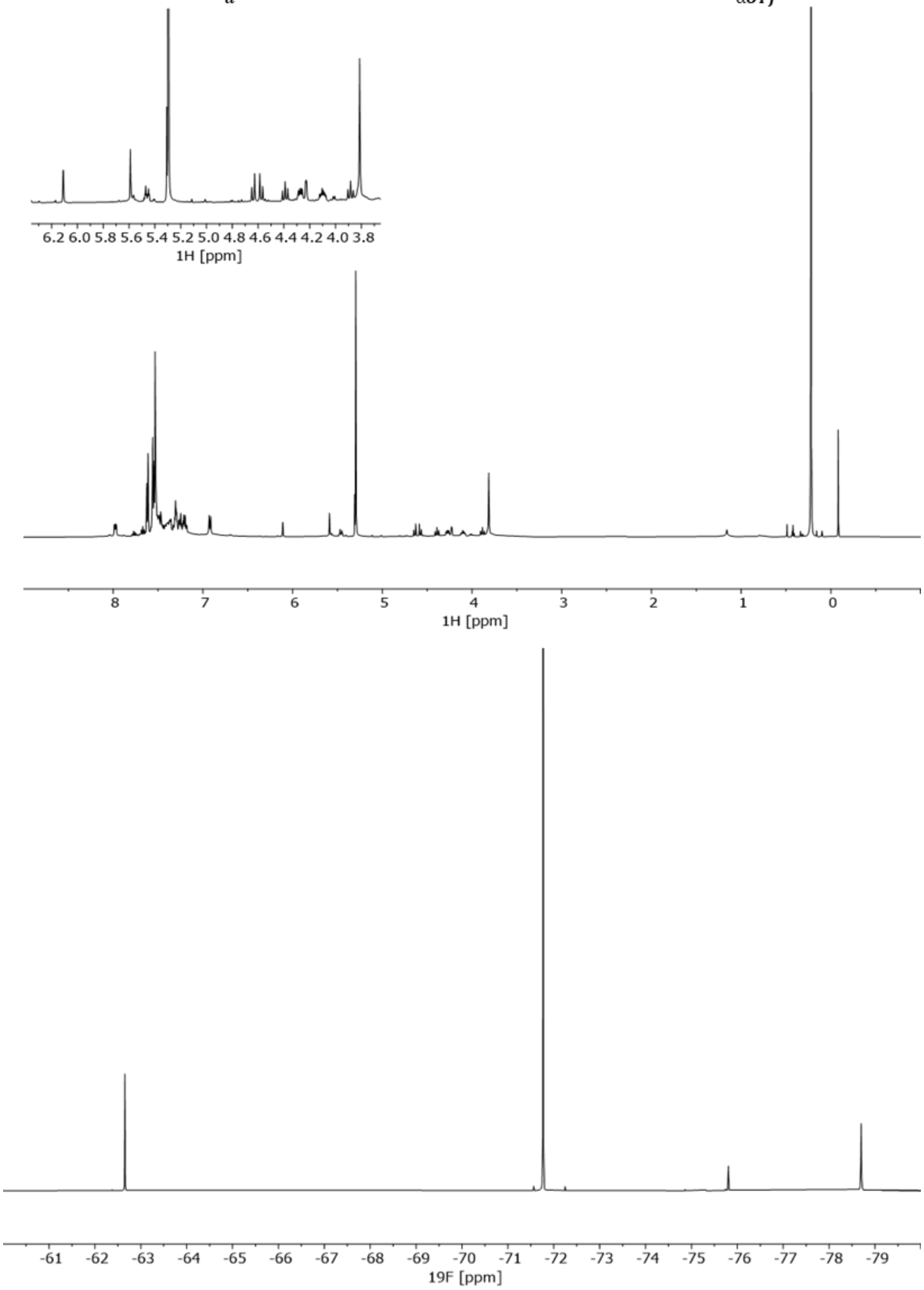

Figure S36: ^{1}H and ^{19}F activation spectra of the α-triflate from the corresponding donor.

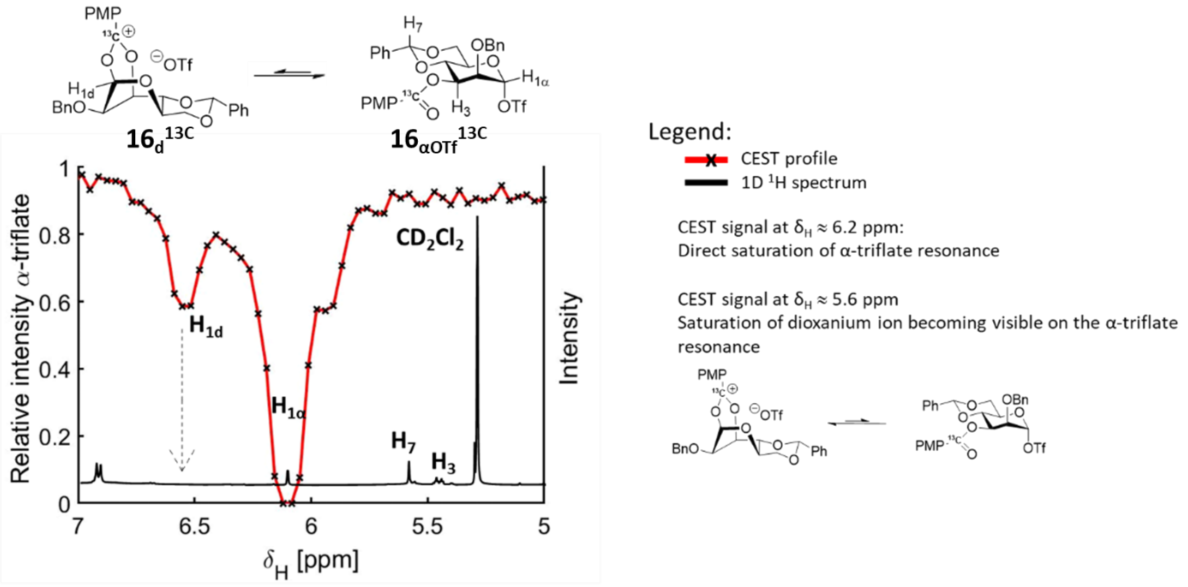

Figure S37: ^{1}H CEST profile overlaid with the 1D ^{1}H NMR spectrum for the corresponding glycosyl α-triflate, displaying evidence for a dioxanium ion only in ^{13}C CEST NMR spectroscopy.

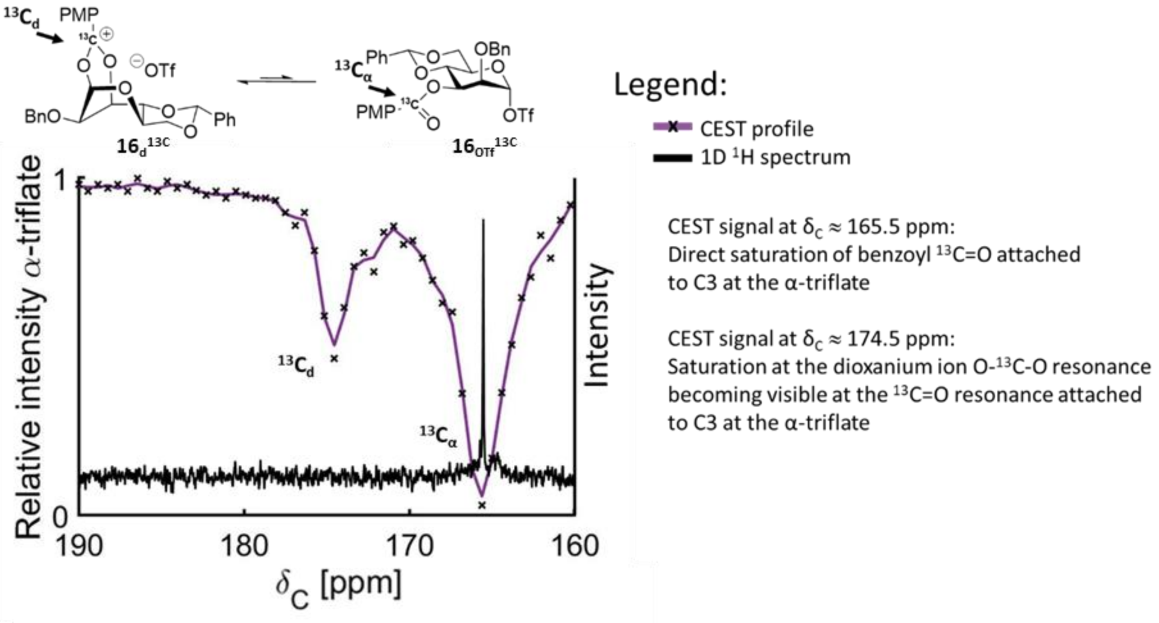

Figure S38: ^{13}C CEST profile overlaid with the 1D ^{13}C NMR spectrum for the corresponding glycosyl α-triflate, displaying evidence for a dioxanium ion only in ^{13}C CEST NMR spectroscopy.

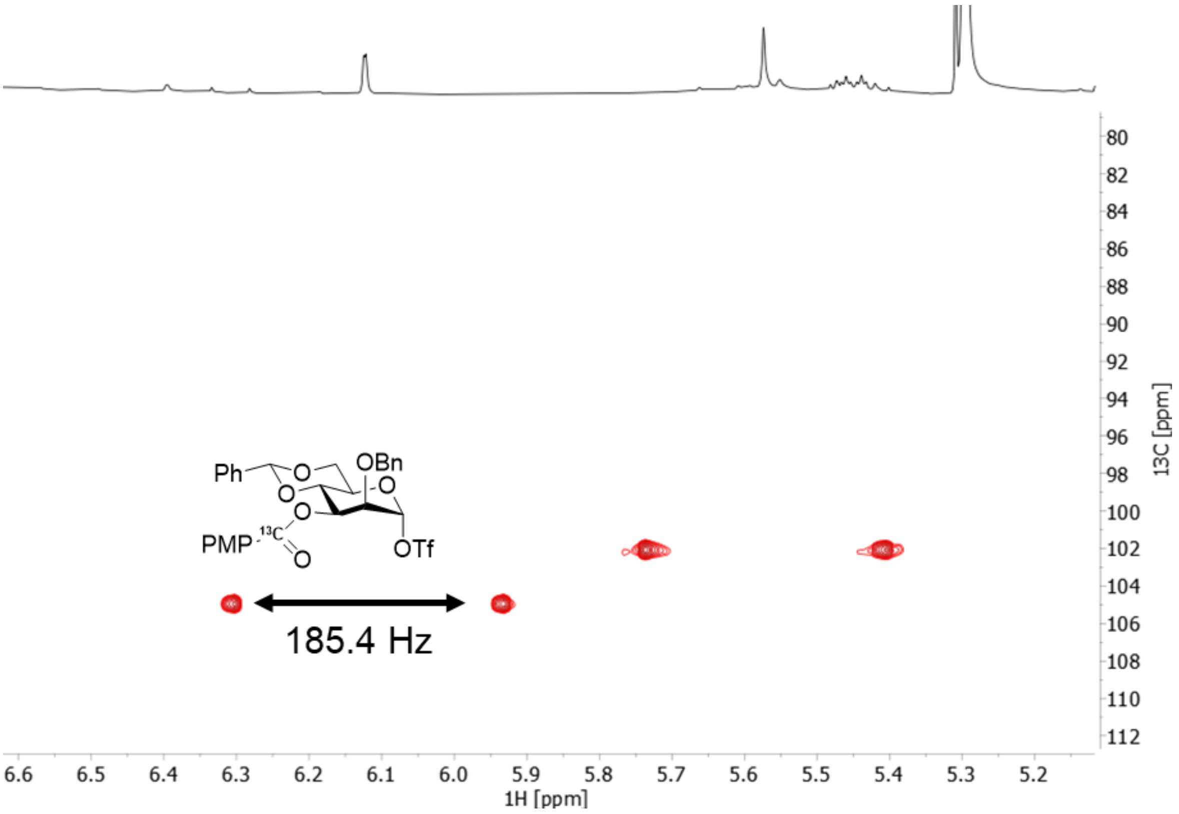

Figure S39: ^{1}H decoupled HSQC zoomed to the anomeric region displaying an equatorial H attached to C1.[11]

^{1}H CEST kinetics to derive the rate of dioxanium ion formation based on ^{1}H CEST experiments

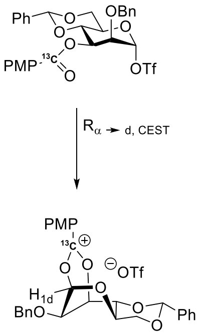

Spectrum number	Saturation time [s]	Intensity H1
1	0	0.007441
2	0.05	0.007182
3	0.1	0.007186
4	0.15	0.007322
5	0.2	0.007169
6	0.27	0.007247
7	0.3	0.007122
8	0.35	0.006997
9	0.4	0.007057
10	0.6	0.006311
11	0.8	0.006718
12	1	0.006381
13	1.5	0.006149
14	2	0.005995
15	3	0.005989
16	4	0.00588
17	6	0.005954

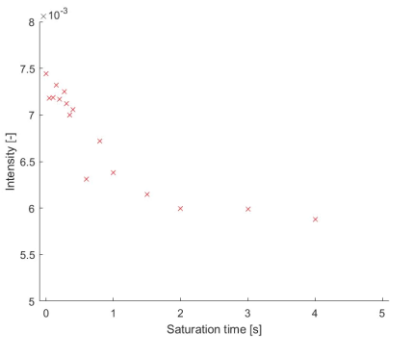

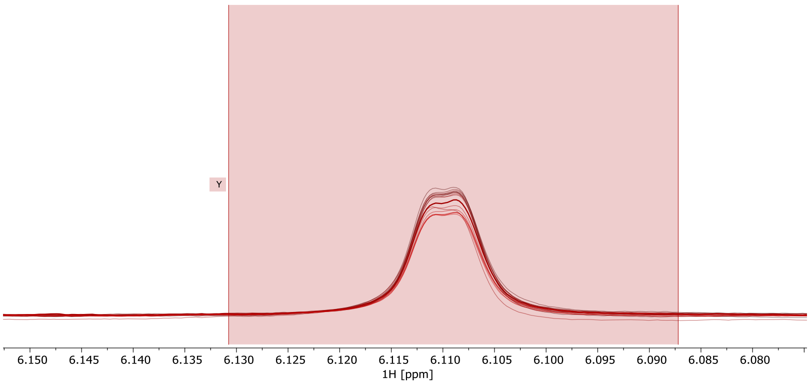

Figure S40: ^{1}H CEST kinetics at -60 °C to determine the rate of dioxanium ion formation.

^{13}C CEST kinetics at -60 ^{o}C to derive the rate of dioxanium ion formation based on ^{13}C CEST experiments

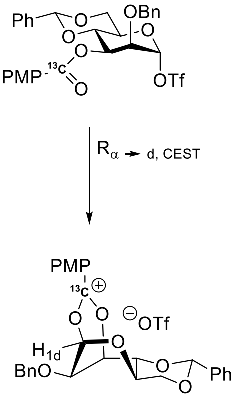

Spectrum number	Saturation time [s]	Intensity ^{13}C=O
1	0	2.92149
2	0.05	2.76368
3	0.1	2.62364
4	0.15	2.50376
5	0.2	2.39772
6	0.27	2.27224
7	0.3	2.22353
8	0.35	2.153
9	0.4	2.09059
10	0.6	1.90787
11	0.8	1.80175
12	1	1.73798
13	1.5	1.67679
14	2	1.67442
15	3	1.68546
16	4	1.68598
17	6	1.68808

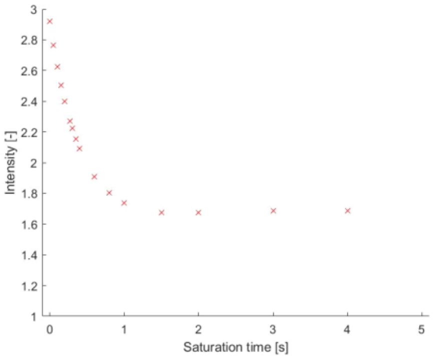

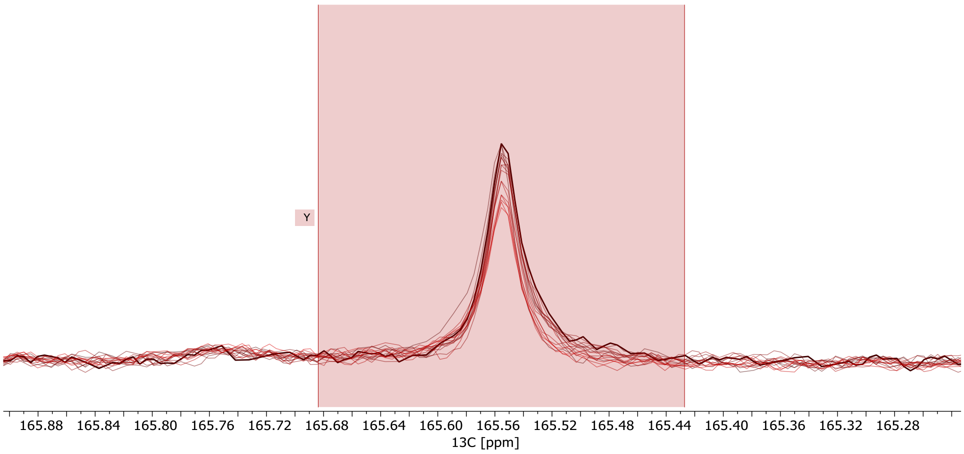

Figure S41: ^{13}C CEST kinetics at -60 °C to determine the rate of dioxanium ion formation.

^{19}F EXSY kinetics at -60 ^{o}C to derive the rate of triflate anion formation

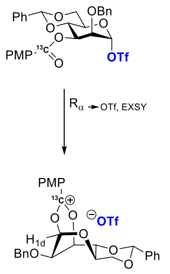

Spectrum number	Mixing Time [s]	*α*-triflate resonance intensity	⁻OTf resonance intensity
1	0.04	3.63921	0.027153
2	0.08	3.15676	0.055879
3	0.12	2.77628	0.078512
4	0.16	2.45887	0.092087
5	0.2	2.14591	0.102104
6	0.24	1.90362	0.115782
7	0.28	1.65596	0.117757
8	0.32	1.4715	0.122603
9	0.36	1.28454	0.127328
10	0.4	1.13699	0.128139

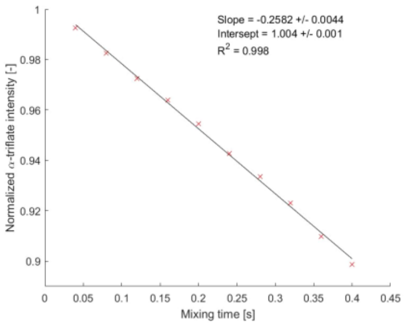

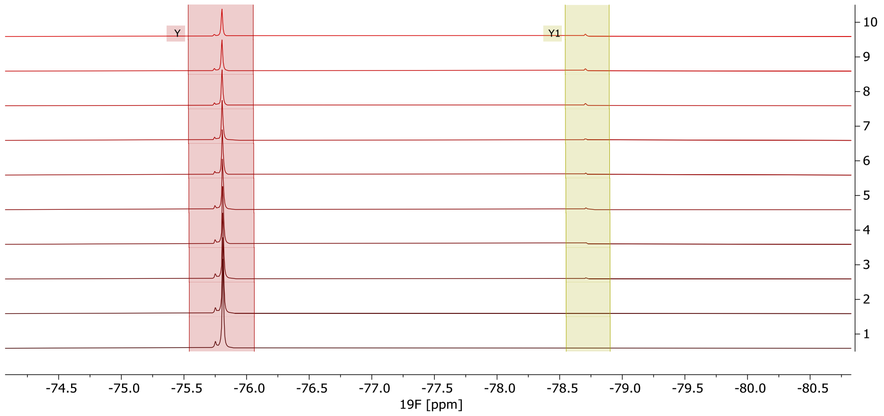

Figure S42: ^{19}F EXSY experiment at -60 °C to determine the α-triflate dissociation rate.

Characterization experiment mannosyl dioxanium ion

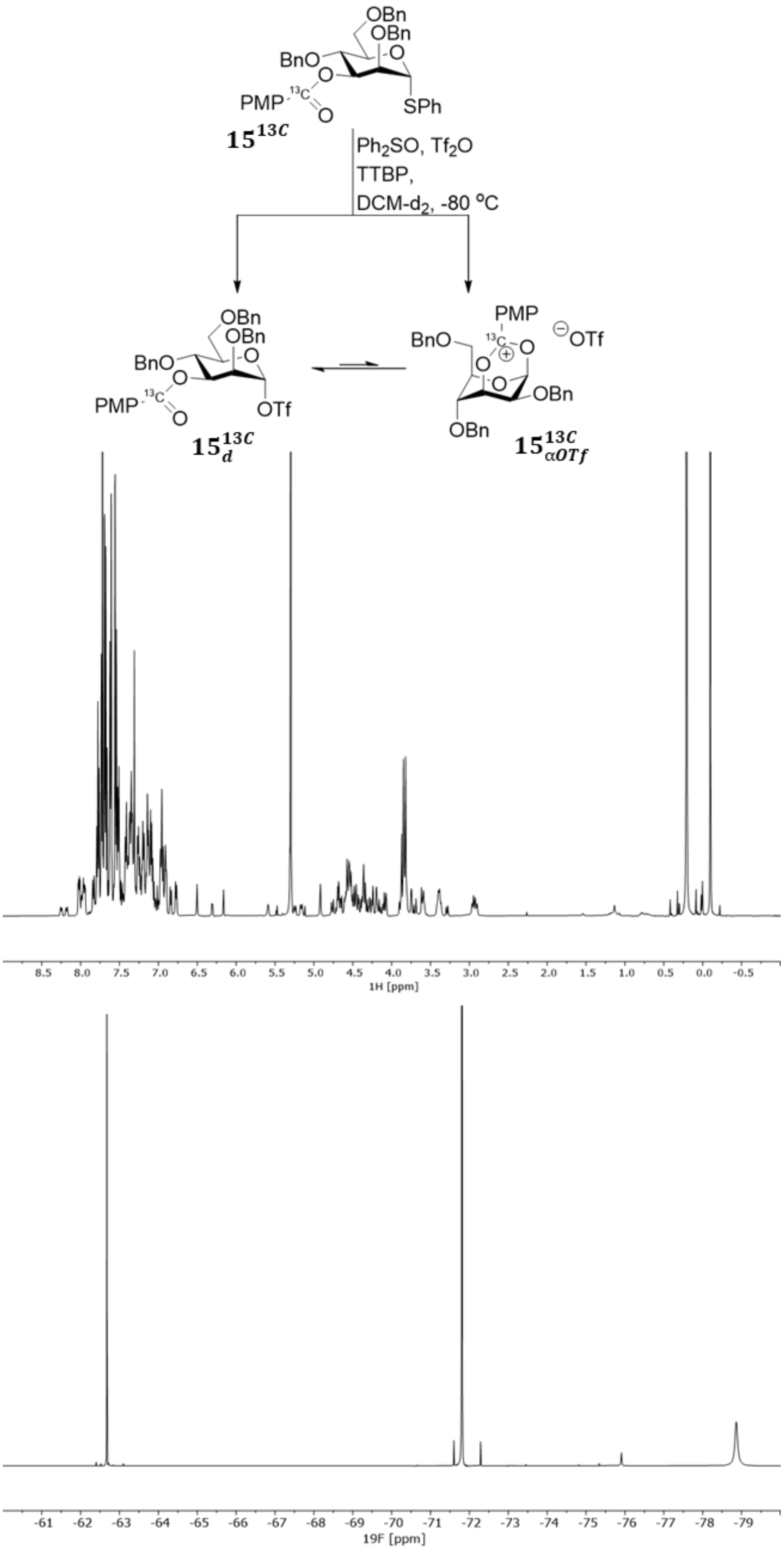

Figure S43: ^{1}H and ^{19}F activation spectra of the α-triflate from the corresponding donor.

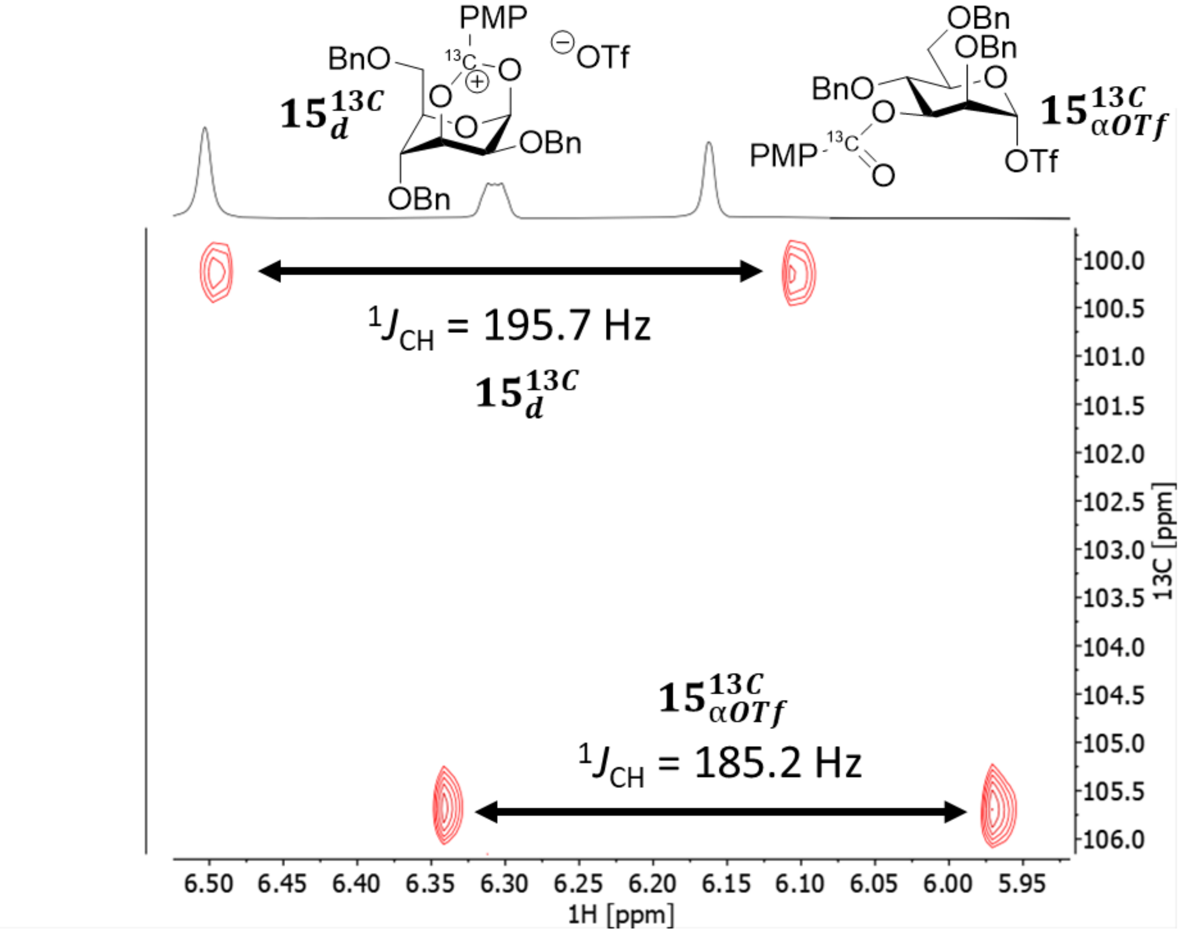

Figure S44: ^{1}H decoupled HSQC zoomed to the anomeric region displaying $^1J_{CH}$-coupling of 195.7 Hz which is in good agreement to observed before for 1,3-bridged mannosyl dioxanium ions.[3]

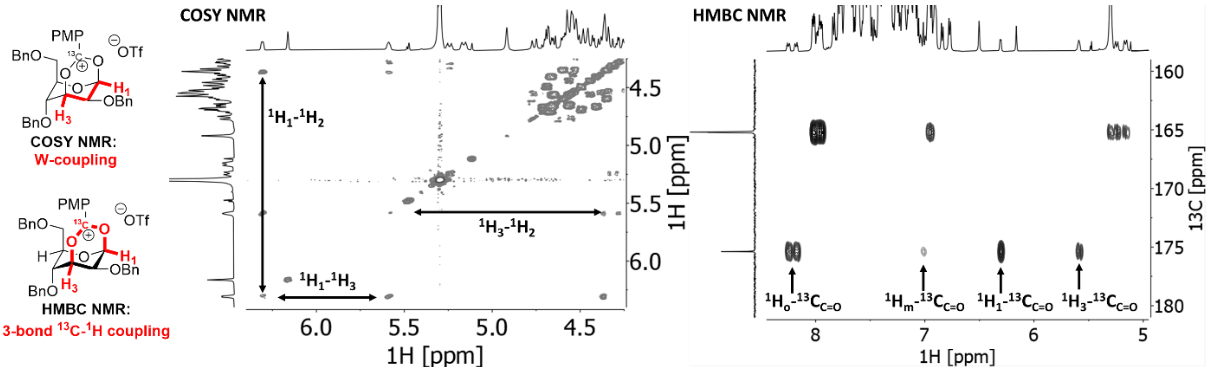

Figure S45: HMBC NMR confirms presence of 1,3-bridge due to cross-peaks to the ^{13}C labelled carbon. COSY NMR confirms the dioxanium ion to be in the $^1C_4$-chair due to a cross-peak between H-1 and H-3 resulting from an ideal angle for long range coupling due to the W-orientation.

Characterization experiment glucosyl β-triflate

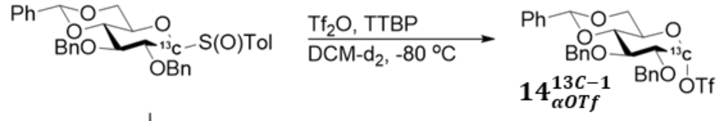

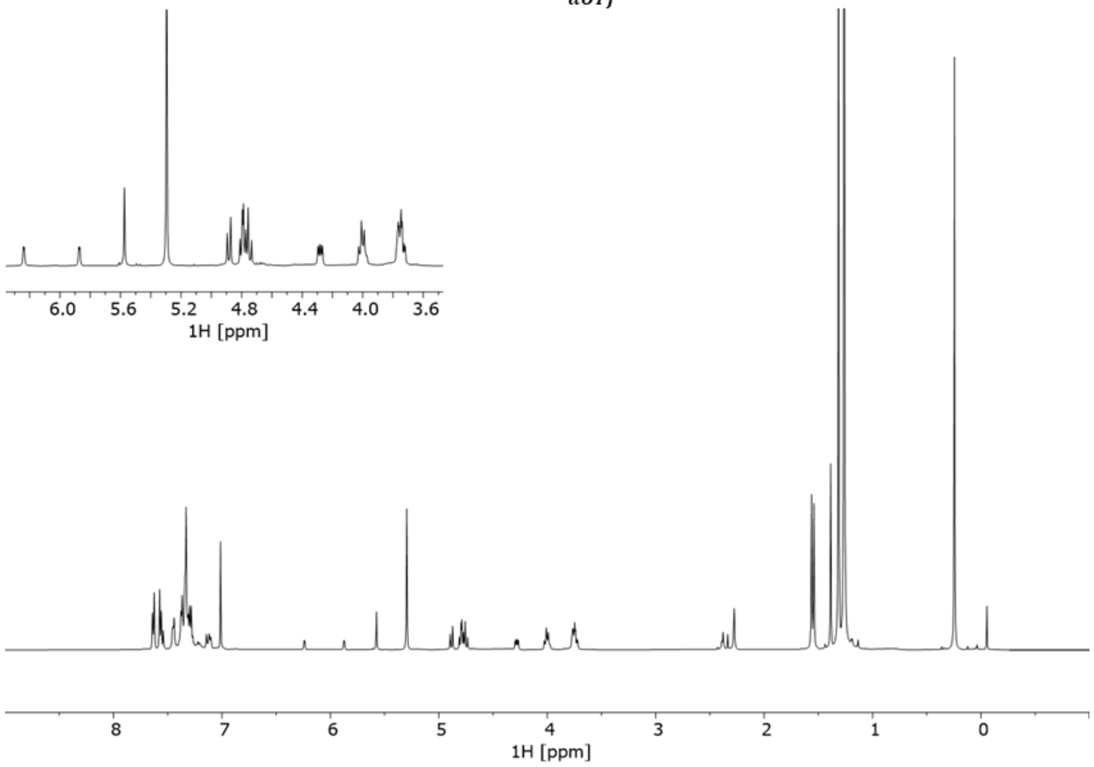

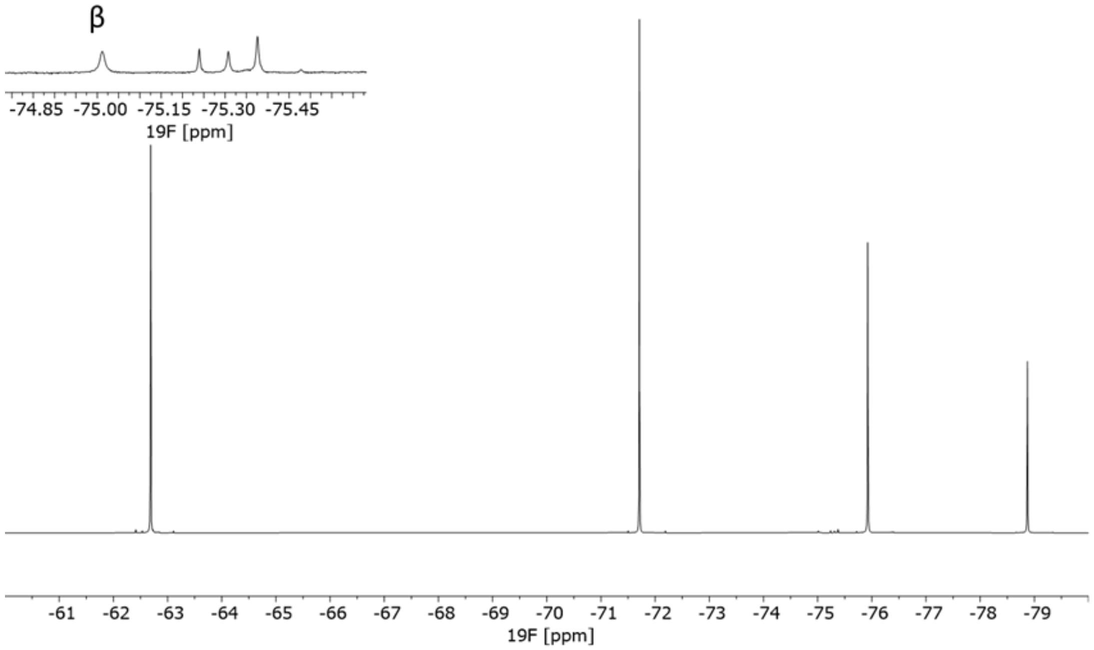

Figure S46: ^{1}H and ^{19}F activation spectra of the α-triflate from the corresponding donor. Minor decomposition was observed but also the β-triflate at $\delta_F \approx 75.0$ ppm as also displayed in Figure S25.

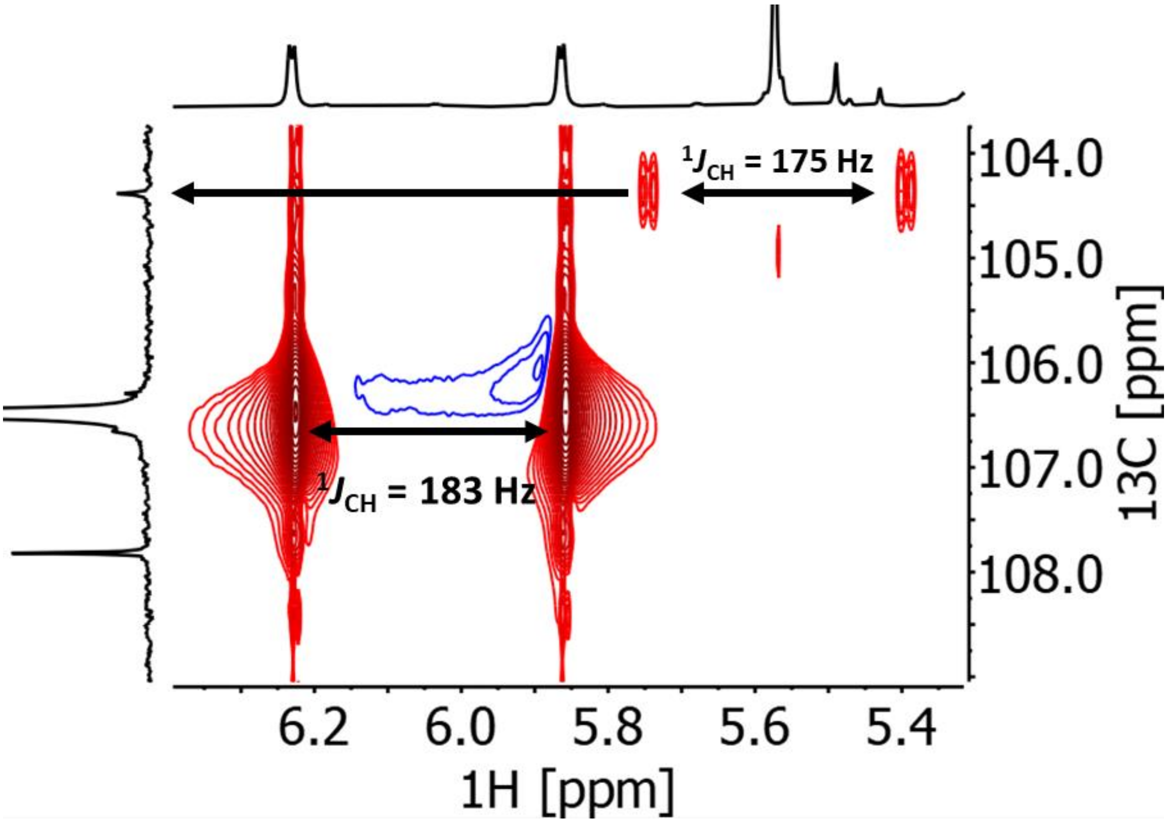

Figure S47: HSQC recorded at -60 °C having sufficiently slow exchange to detect the β-triflate resonance. $^1J_{CH}$ in good agreement as expected based on literature.[11]

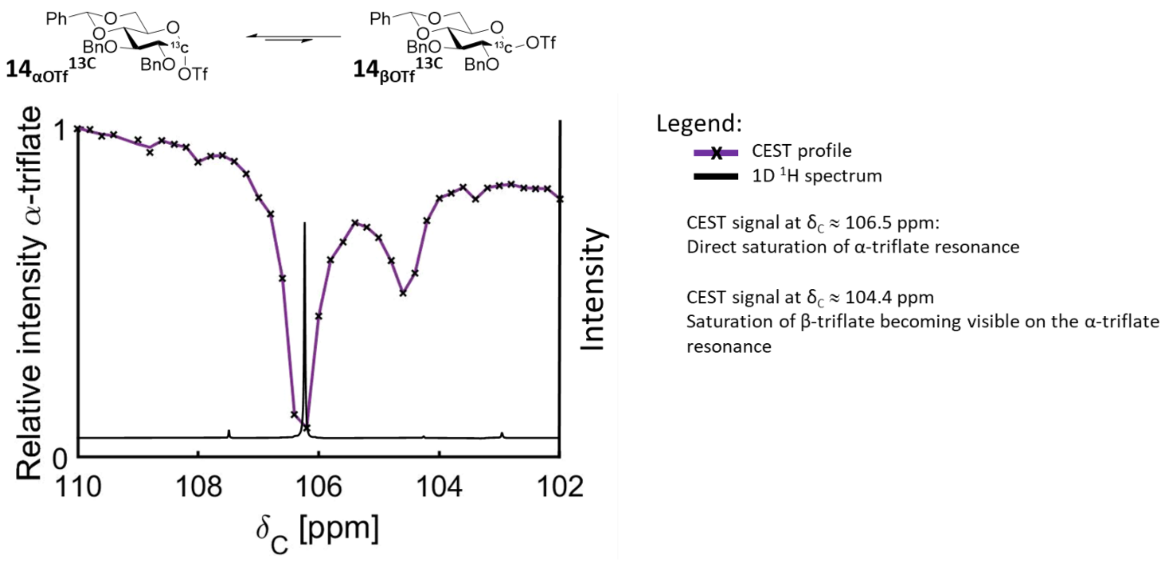

Figure S48: ^{13}C CEST spectrum recorded at -20 °C displaying evidence in ^{13}C CEST NMR.

T1 measurement at -80 °C

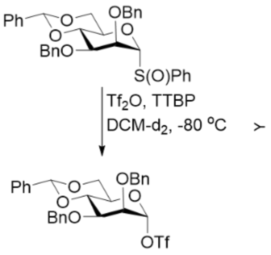

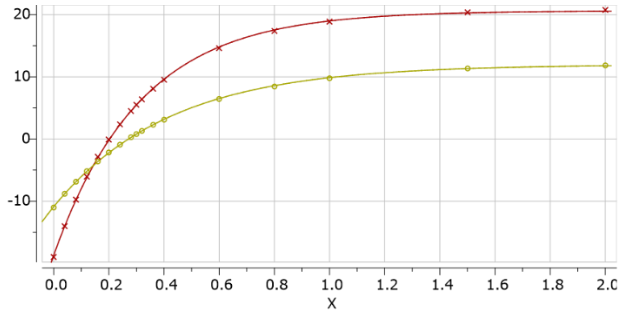

Spec. no.	Delay time [s]	α-triflate	$^{-}$OTf
1	0	-18.6998	-11.003
2	0.04	-13.9953	-8.81765
3	0.08	-9.85381	-6.88291
4	0.12	-6.20787	-5.13871
5	0.16	-2.9982	-3.57015
6	0.2	-0.172611	-2.15973
7	0.24	2.31486	-0.880023
8	0.28	4.50468	0.281698
9	0.3	5.49927	0.812416
10	0.32	6.43246	1.32345
11	0.36	8.12956	2.27757
12	0.4	9.62358	3.13979
13	0.6	14.8033	6.4273
14	0.8	17.542	8.48119
15	1	18.9901	9.79945
16	1.5	20.2846	11.364
17	2	20.5477	11.8782

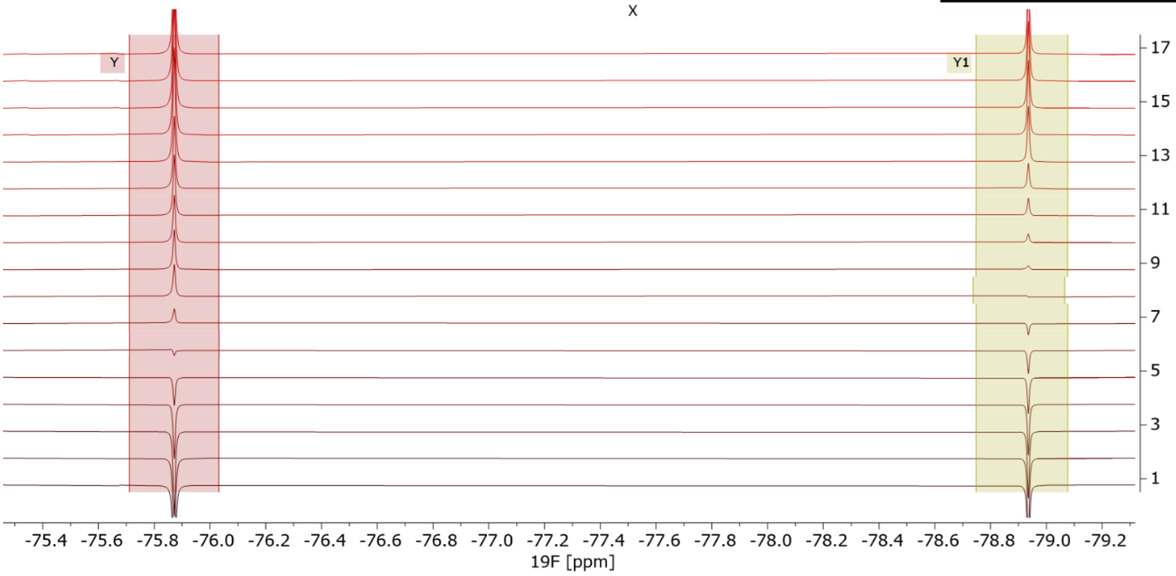

Figure S49: Inversion recovery measurement at -80 °C giving T1 = 0.31 s and 0.42s for the α-triflate and $^{-}$OTf respectively.

References

1 Serianni, A. S., Pierce, J., Huang, S. G. & Barker, R. Anomerization of furanose sugars: kinetics of ring-opening reactions by proton and carbon-13 saturation-transfer NMR spectroscopy. *Journal of the American Chemical Society* **104**, 4037-4044 (1982).

2 McConnell, H. M. Reaction rates by nuclear magnetic resonance. *The Journal of chemical physics* **28**, 430-431 (1958).

3 de Kleijne, F. F., Elferink, H., Moons, S. J., White, P. B. & Boltje, T. J. Characterization of Mannosyl Dioxanium Ions in Solution Using Chemical Exchange Saturation Transfer NMR Spectroscopy. *Angewandte Chemie International Edition* **61**, e202109874 (2022).

4 Elferink, H. *et al.* Synthesis and cellular uptake of carbamoylated mannose derivatives. *Carbohydrate research* **481**, 67-71 (2019).

5 Tortajada, A. *et al.* Catalytic decarboxylation/carboxylation platform for accessing isotopically labeled carboxylic acids. *ACS Catalysis* **9**, 5897-5901 (2019).

6 Elferink, H. *et al.* The Glycosylation Mechanisms of 6, 3-Uronic Acid Lactones. *Angewandte Chemie International Edition* **58**, 8746-8751 (2019).

7 Cheng, M. S. *et al.* Total synthesis of methyl protodioscin: a potent agent with antitumor activity. *The Journal of Organic Chemistry* **68**, 3658-3662 (2003).

8 Remmerswaal, W. A. *et al.* Stabilization of Glucosyl Dioxolenium Ions by “Dual Participation” of the 2, 2-Dimethyl-2-(ortho-nitrophenyl) acetyl (DMNPA) Protection Group for 1, 2-cis-Glucosylation. *The Journal of Organic Chemistry* **87**, 9139-9147 (2022).

9 Nielsen, M. M., Stougaard, B. A., Bols, M., Glibstrup, E. & Pedersen, C. M. Glycosyl Fluorides as Intermediates in BF3· OEt2-Promoted Glycosylation with Trichloroacetimidates. *European Journal of Organic Chemistry* **2017**, 1281-1284 (2017).

10 Bolik-Coulon, N., Hansen, D. F. & Kay, L. E. Optimizing frequency sampling in CEST experiments. *Journal of Biomolecular NMR* **76**, 167-183 (2022).

11 Bock, K. & Pedersen, C. A study of 13 CH coupling constants in hexopyranoses. *Journal of the Chemical Society, Perkin Transactions 2*, 293-297 (1974).

Data

Variable temperature kinetic experiments:

1D Selective ^{19}F EXSY data at -80 °C

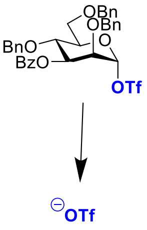

Spectrum number	Mixing Time [s]	α-triflate (-75.90 ppm) resonance intensity	⁻OTf (-79.04 ppm) resonance intensity
1	0.1	8672.3	160.96
2	0.15	7340.96	211.009
3	0.2	6320.28	309.855
4	0.25	5375.66	290.819
5	0.3	4648.48	335.709
6	0.35	3949.38	350.181
7	0.4	3407.61	413.257
8	0.45	2901.93	403.519
9	0.5	2547.82	365.07
10	0.55	2235.97	357.648

[⁻OTf] = 0.01 M

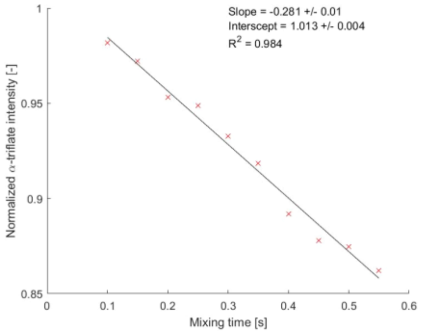

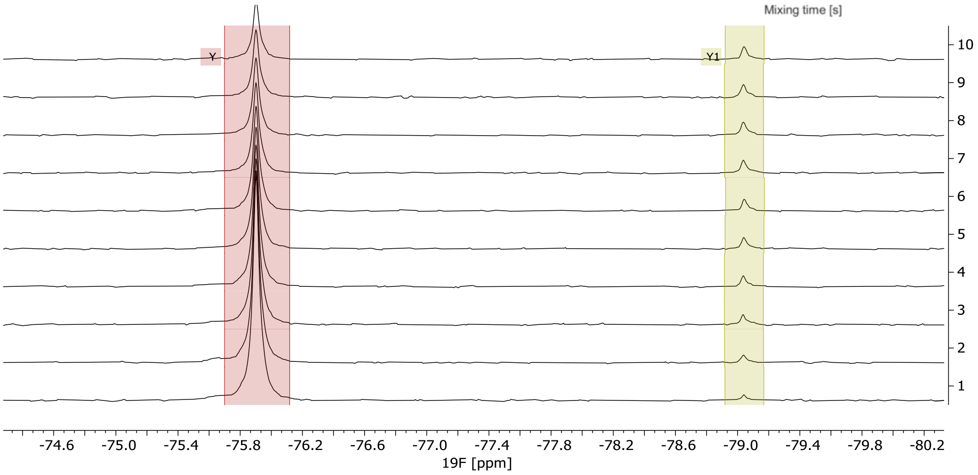

Figure S50: Raw ^{19}F EXSY data for the *α*-triflate dissociation in the corresponding *α*-triflate (left corner) measured at -80 °C.

1D Selective ^{19}F EXSY data at -70 °C

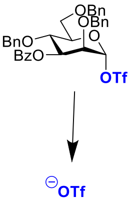

Spectrum number	Mixing Time [s]	α-triflate (-75.90 ppm) resonance intensity	⁻OTf (-79.02 ppm) resonance intensity
1	0.01	11758	192.106
2	0.02	11044	344.723
3	0.03	10780.9	469.318
4	0.04	10238	612.878
5	0.05	9830.21	767.521
6	0.06	9636.13	867.589
7	0.07	9314	1026.67
8	0.08	8947.44	1131.27
9	0.09	8645.12	1209.89

[⁻OTf] = 0.01 M

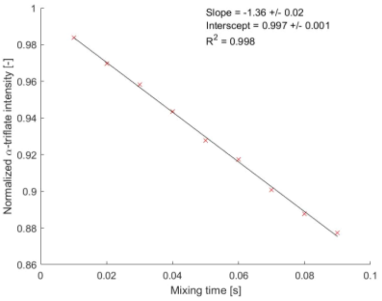

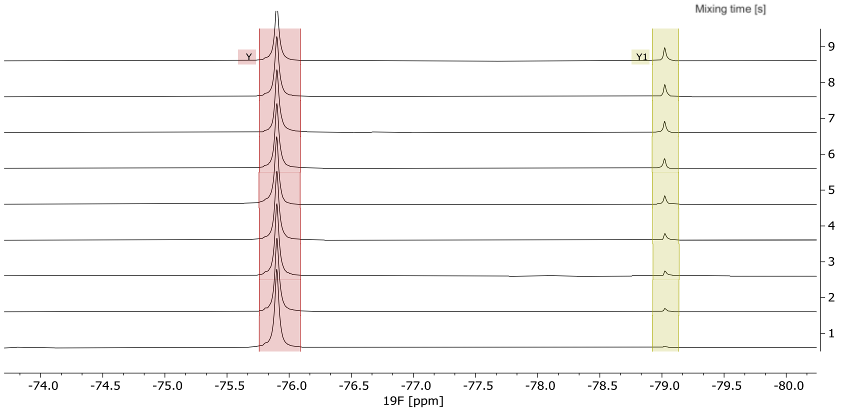

Figure S51: Raw ^{19}F EXSY data for the *α*-triflate dissociation in the corresponding *α*-triflate (left corner) measured at -70 °C.

1D Selective ^{19}F EXSY data at -60 ^{o}C

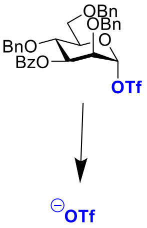

Spectrum number	Mixing Time [s]	*α*-triflate (-75.89 ppm) resonance intensity	$^{-}$OTf (-78.99 ppm) resonance intensity
1	0.005	8081.25	171.477
2	0.008	7914.62	271.955
3	0.011	8099.5	386.62
4	0.014	8276.13	528.669
5	0.017	8054.85	635.573
6	0.02	7802.73	753.938
7	0.023	7912.89	863.316
8	0.025	8126.15	968.876
9	0.026	7983.61	997.231

[$^{-}$OTf] = 0.01 M

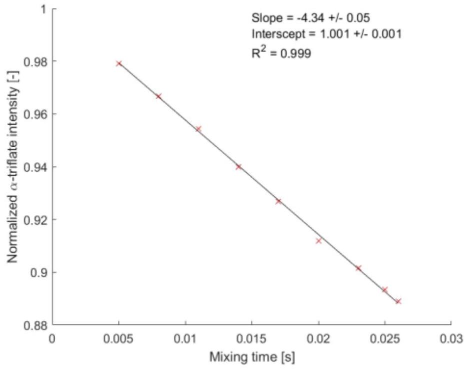

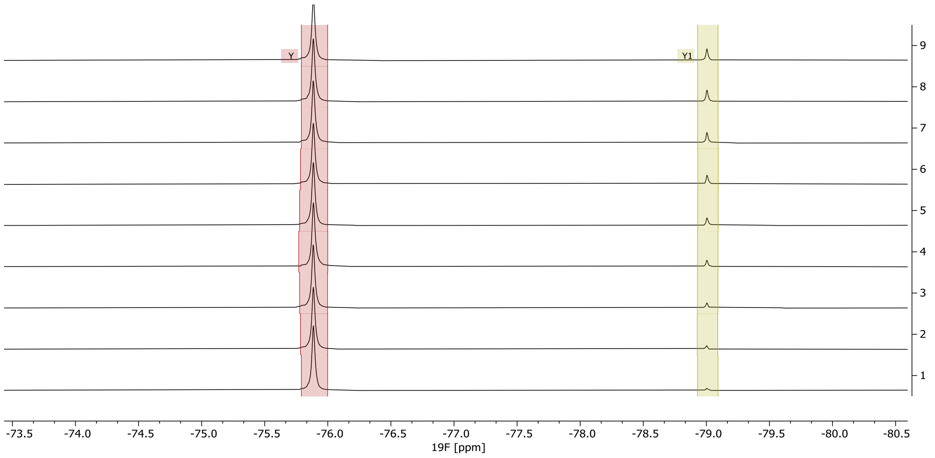

Figure S52: Raw ^{19}F EXSY data for the *α*-triflate dissociation in the corresponding *α*-triflate (left corner) measured at -60 °C.

1D Selective ^{19}F EXSY data at -50 ^{o}C

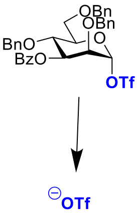

Spectrum number	Mixing Time [s]	*α*-triflate (-75.88 ppm) resonance intensity	$^{-}$OTf (-78.99 ppm) resonance intensity
1	0.001	11892.3	183.371
2	0.002	12341	318.129
3	0.003	12203	510.628
4	0.004	11763.7	621.772
5	0.005	12228.1	807.878
6	0.006	12480.9	967.201
7	0.007	12347.3	1086.38
8	0.008	12099.7	1224.68
9	0.009	12430.3	1380.59
10	0.01	12667.2	1511.98

[$^{-}$OTf] = 0.01 M

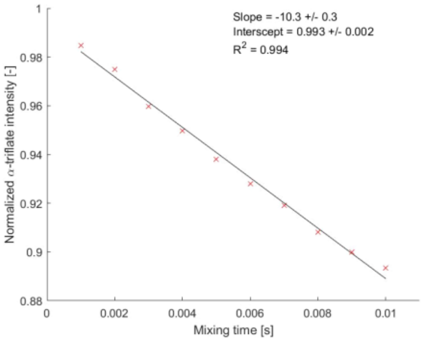

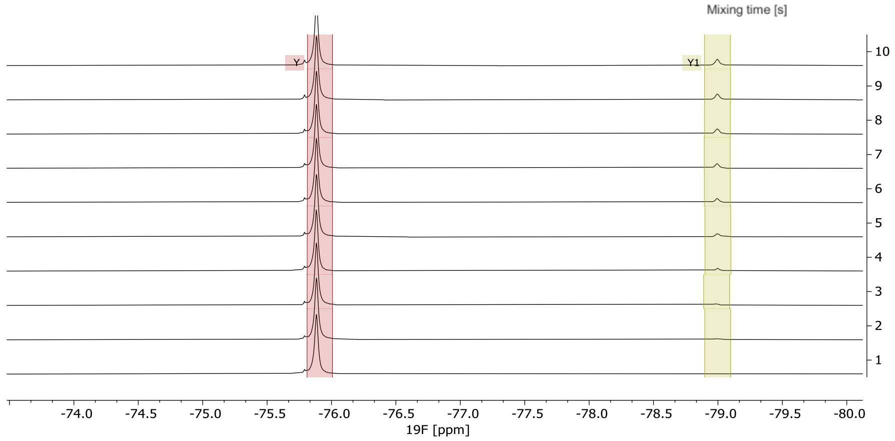

Figure S53: Raw ^{19}F EXSY data for the *α*-triflate dissociation in the corresponding *α*-triflate (left corner) measured at -50 °C.

1D Selective ^{19}F EXSY data at -70 °C

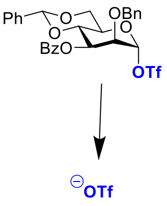

Spectrum number	Mixing Time [s]	α-triflate (-75.73 ppm) resonance intensity	⁻OTf (-78.93 ppm) resonance intensity
1	0.1	4.65494	0.031637
2	0.2	3.41953	0.050364
3	0.3	2.48435	0.057408
4	0.4	1.80536	0.059257
5	0.5	1.31609	0.055782
6	0.6	0.958435	0.049644
7	0.7	0.701957	0.047817
8	0.8	0.515497	0.043772
9	0.9	0.37783	0.038898
10	1	0.277055	0.030944

[⁻OTf] = 0.01 M

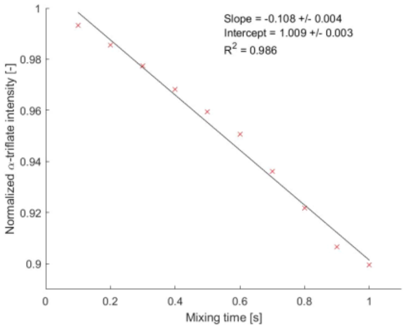

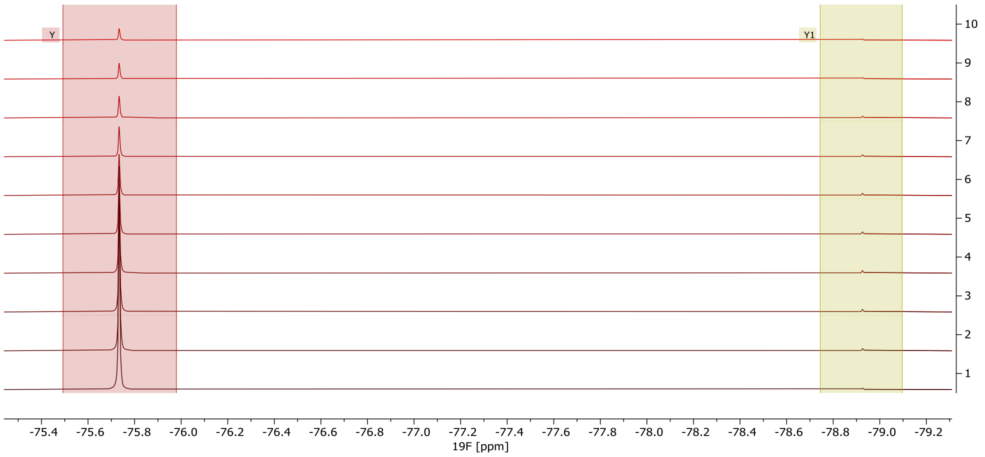

Figure S54: Raw ^{19}F EXSY data for the *α*-triflate dissociation in the corresponding *α*-triflate (left corner) measured at -70 °C.

1D Selective ^{19}F EXSY data at -60 °C

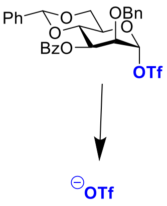

Spectrum number	Mixing Time [s]	α-triflate (-75.72 ppm) resonance intensity	⁻OTf (-78.91 ppm) resonance intensity
1	0.04	7.85278	0.080111
2	0.08	7.01015	0.143408
3	0.12	6.12209	0.194143
4	0.16	5.49092	0.234082
5	0.2	4.87642	0.262122
6	0.24	4.31513	0.281729
7	0.28	3.8663	0.296154
8	0.32	3.43203	0.30398
9	0.36	3.05922	0.307114
10	0.4	2.74691	0.310083

[⁻OTf] = 0.01 M

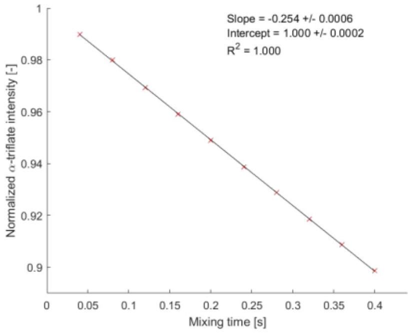

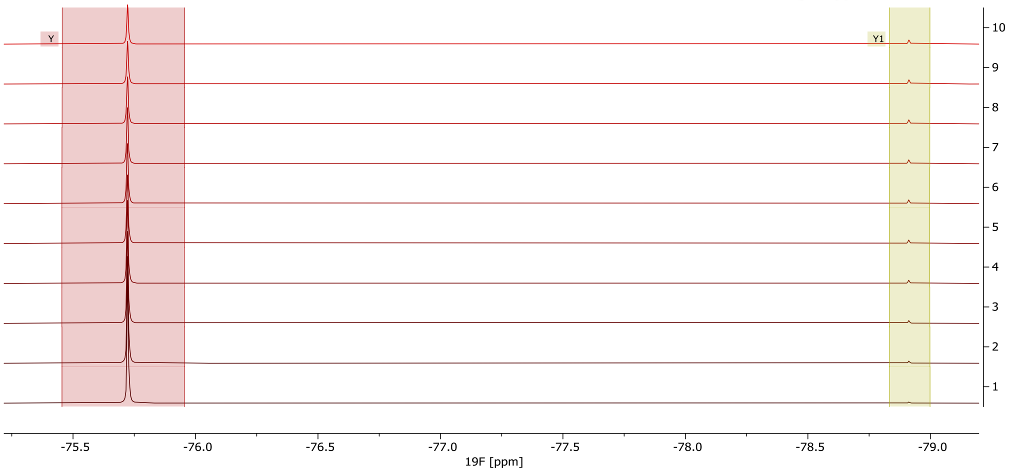

Figure S55: Raw ^{19}F EXSY data for the *α*-triflate dissociation in the corresponding *α*-triflate (left corner) measured at -60 °C.

1D Selective ^{19}F EXSY data at -50 °C

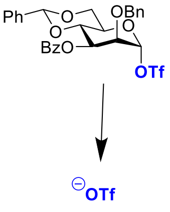

Spectrum number	Mixing Time [s]	α-triflate (-75.71 ppm) resonance intensity	$^{-}$OTf (-78.90 ppm) resonance intensity
1	0.014	8.50086	0.093185
2	0.028	8.09206	0.181079
3	0.042	7.59413	0.259212
4	0.056	7.53269	0.326952
5	0.07	7.02981	0.39229
6	0.084	6.76484	0.441272
7	0.098	6.51588	0.493824
8	0.112	6.16771	0.536466
9	0.126	5.97222	0.57288
10	0.14	5.68342	0.609041

[$^{-}$OTf] = 0.01 M

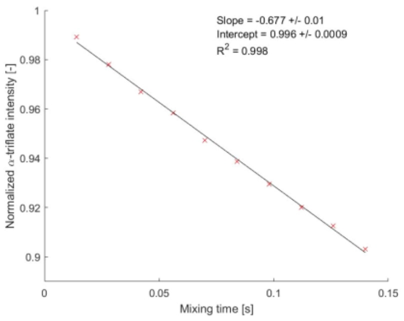

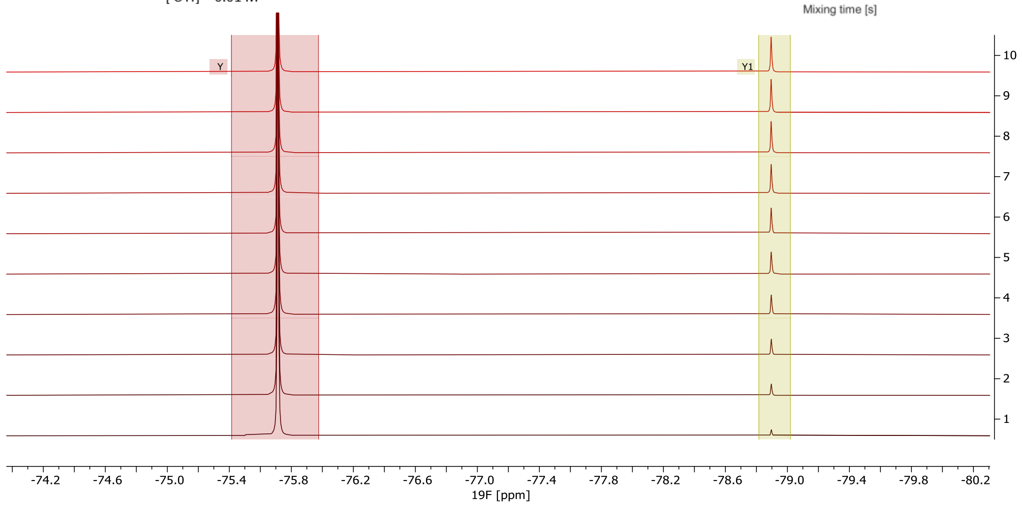

Figure S56: Raw ^{19}F EXSY data for the *α*-triflate dissociation in the corresponding *α*-triflate (left corner) measured at -50 °C.

1D Selective ^{19}F EXSY data at -40 °C

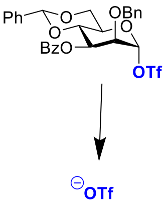

Spectrum number	Mixing Time [s]	α-triflate (-75.70 ppm) resonance intensity	$^{-}$OTf (-78.88 ppm) resonance intensity
1	0.005	6.55851	0.070017
2	0.01	6.26288	0.137419
3	0.015	6.45595	0.207365
4	0.02	6.50411	0.270699
5	0.025	6.36547	0.340854
6	0.03	6.60363	0.402502
7	0.035	6.33688	0.467285
8	0.04	6.50666	0.529717
9	0.045	6.46182	0.584367
10	0.05	6.41051	0.647296

[$^{-}$OTf] = 0.01 M

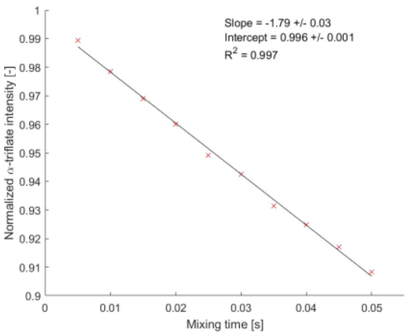

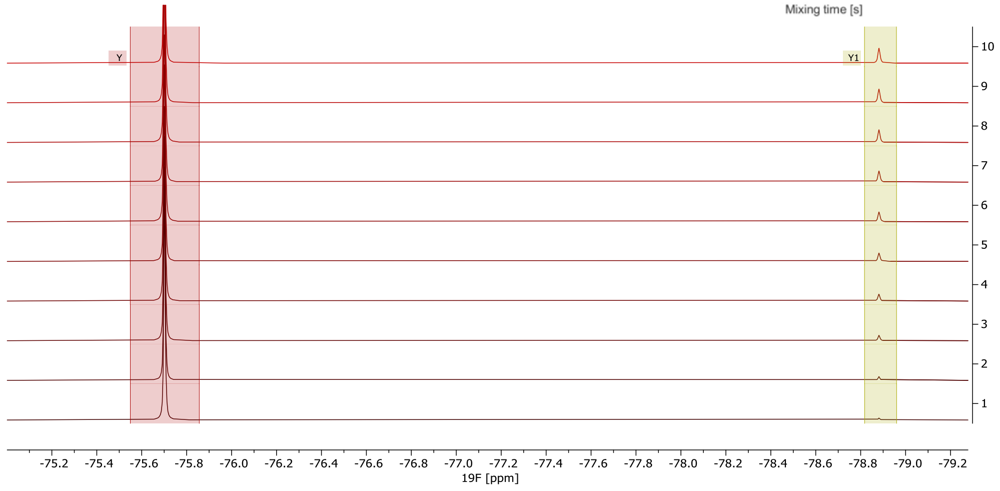

Figure S57: Raw ^{19}F EXSY data for the *α*-triflate dissociation in the corresponding *α*-triflate (left corner) measured at -40 °C.

1D Selective ^{19}F EXSY data at -30 °C

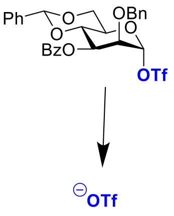

Spectrum number	Mixing Time [s]	α-triflate (-75.69 ppm) resonance intensity	⁻OTf (-78.86 ppm) resonance intensity
1	0.002	7.10625	0.075266
2	0.004	7.0318	0.152567
3	0.006	6.77672	0.223281
4	0.008	6.83942	0.295359
5	0.01	6.67762	0.356993
6	0.012	6.52747	0.4177
7	0.014	6.52453	0.478404
8	0.016	6.32074	0.535555
9	0.018	6.2616	0.588825
10	0.02	6.25014	0.641369

[⁻OTf] = 0.01 M

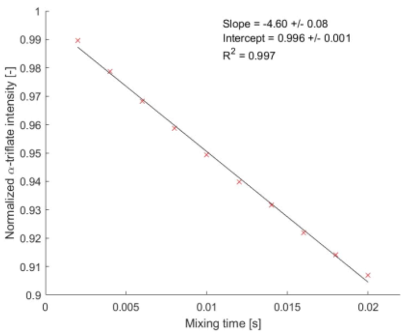

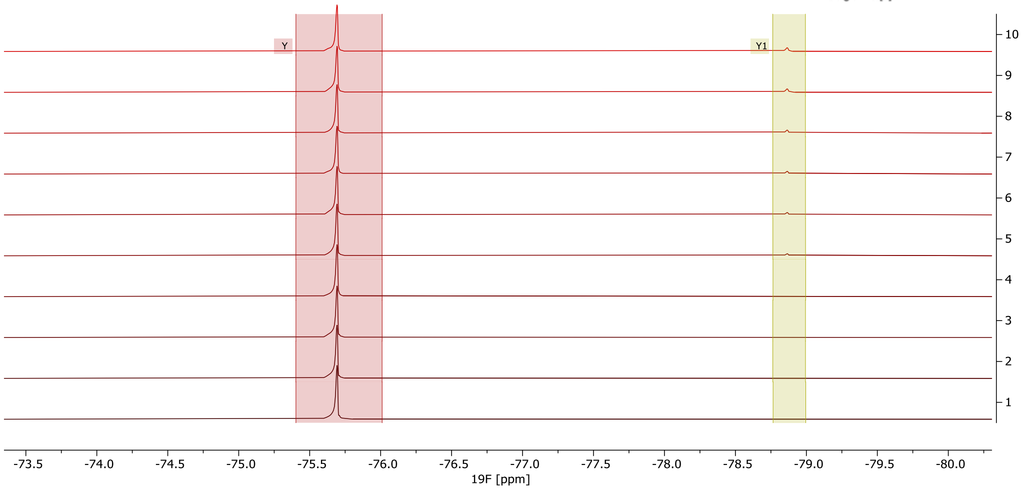

Figure S58: Raw ^{19}F EXSY data for the *α*-triflate dissociation in the corresponding *α*-triflate (left corner) measured at -30 °C.

1D Selective ^{19}F EXSY data at -20 °C

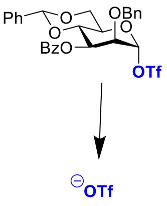

Spectrum number	Mixing Time [s]	α-triflate (-75.67 ppm) resonance intensity	⁻OTf (-78.84 ppm) resonance intensity
1	0.0006	5.18975	0.040792
2	0.0012	5.04417	0.082781
3	0.0018	4.79956	0.122225
4	0.0024	4.94128	0.160588
5	0.003	4.62054	0.19544
6	0.0036	4.72097	0.234154
7	0.0042	4.5261	0.265373
8	0.0048	4.33818	0.300214
9	0.0054	4.40867	0.333461
10	0.006	4.15345	0.362987

[⁻OTf] = 0.01 M

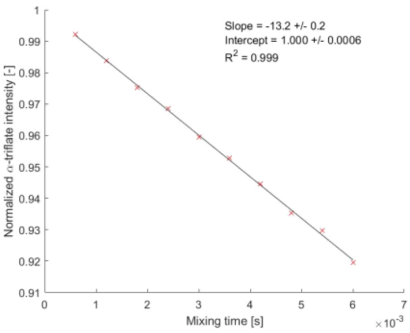

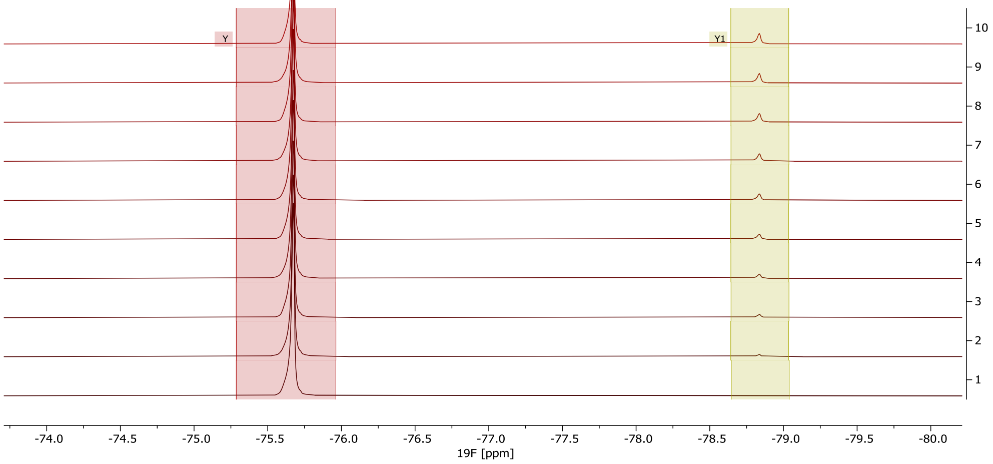

Figure S59: Raw ^{19}F EXSY data for the *α*-triflate dissociation in the corresponding *α*-triflate (left corner) measured at -20 °C.

1D Selective ^{19}F EXSY data at -60 ^{o}C

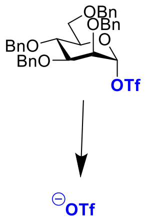

Spectrum number	Mixing Time [s]	*α*-triflate (-75.97 ppm) resonance intensity	$^{-}$OTf (-78.90 ppm) resonance intensity
1	0.05	3.62793	0.0287205
2	0.1	3.13074	0.0401867
3	0.15	2.69219	0.052312
4	0.2	2.33114	0.0626856
5	0.25	1.99635	0.0670015
6	0.3	1.71973	0.070856
7	0.35	1.49745	0.0757945
8	0.4	1.30728	0.0787283
9	0.45	1.11958	0.0772648
10	0.5	0.974104	0.0747759
11	0.55	0.852376	0.0770592
12	0.6	0.732092	0.0710966
13	0.65	0.633619	0.0703663
14	0.7	0.5559	0.0690227

[$^{-}$OTf] = 0.033 M

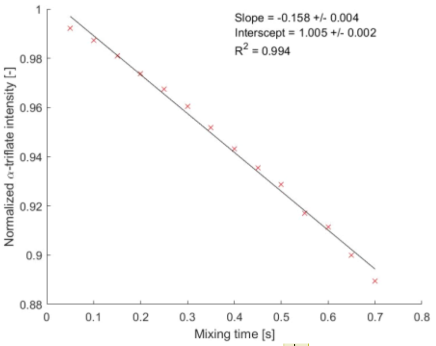

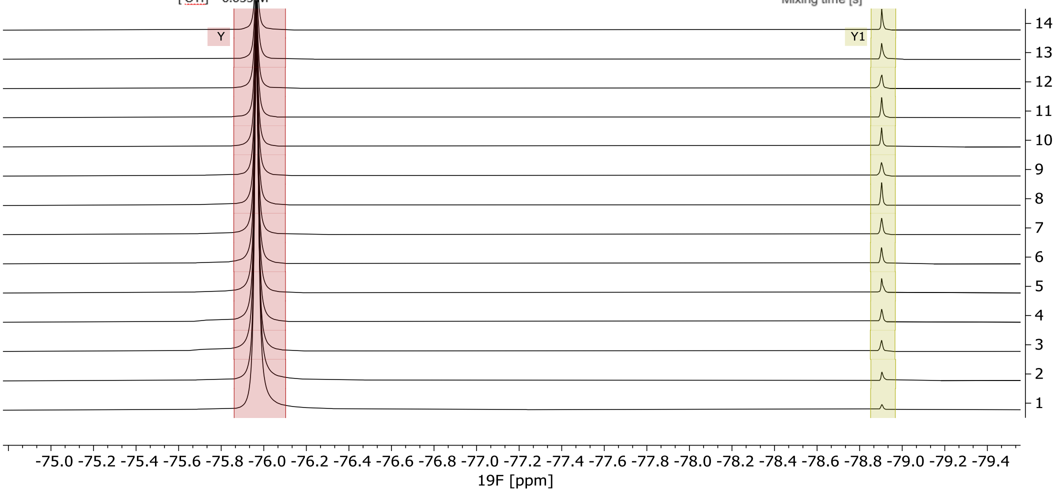

Figure S60: Raw ^{19}F EXSY data for the *α*-triflate dissociation in the corresponding *α*-triflate (left corner) measured at -60 °C.

1D Selective ^{19}F EXSY data at -50 ^{o}C

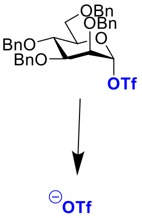

Spectrum number	Mixing Time [s]	*α*-triflate (-75.95 ppm) resonance intensity	$^{-}$OTf (-78.89 ppm) resonance intensity
1	0.025	2.48452	0.0176001
2	0.05	2.30472	0.0329916
3	0.075	2.1373	0.0571924
4	0.1	1.96293	0.0692229
5	0.125	1.82326	0.0804797
6	0.15	1.66324	0.0919814
7	0.175	1.53586	0.101314
8	0.2	1.41769	0.107891
9	0.225	1.30966	0.112914
10	0.25	1.20699	0.116125

[$^{-}$OTf] = 0.034 M

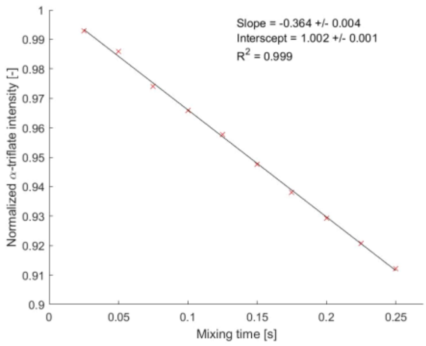

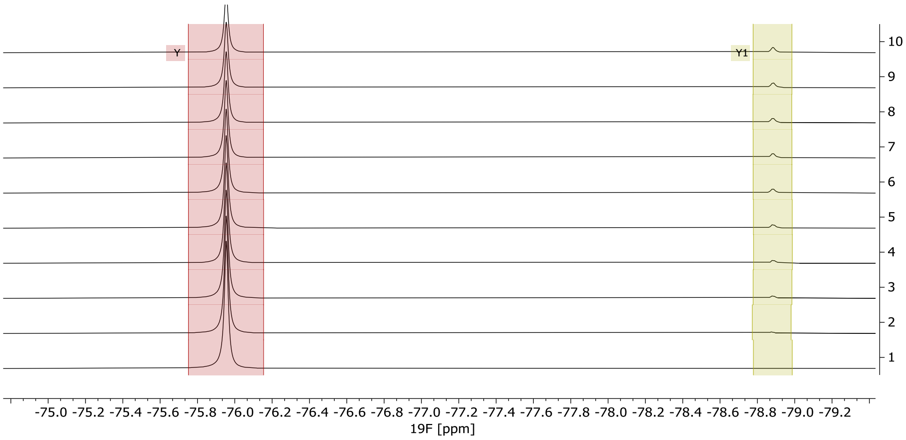

Figure S61: Raw ^{19}F EXSY data for the *α*-triflate dissociation in the corresponding *α*-triflate (left corner) measured at -50 °C.

1D Selective ^{19}F EXSY data at -45 ^{o}C

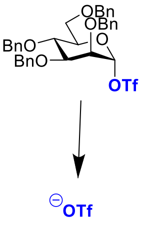

Spectrum number	Mixing Time [s]	α-triflate (-75.95 ppm) resonance intensity	⁻OTf (-78.87 ppm) resonance intensity
1	0.015	1.68769	0.0171614
2	0.03	1.62436	0.0401318
3	0.045	1.54132	0.0454135
4	0.06	1.47231	0.0591101
5	0.075	1.38205	0.0692924
6	0.09	1.29881	0.081299
7	0.105	1.2273	0.0905169
8	0.12	1.15674	0.096672
9	0.135	1.104	0.106574
10	0.15	1.04503	0.114837

[⁻OTf] = 0.039 M

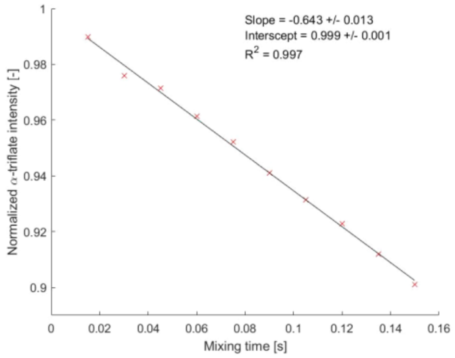

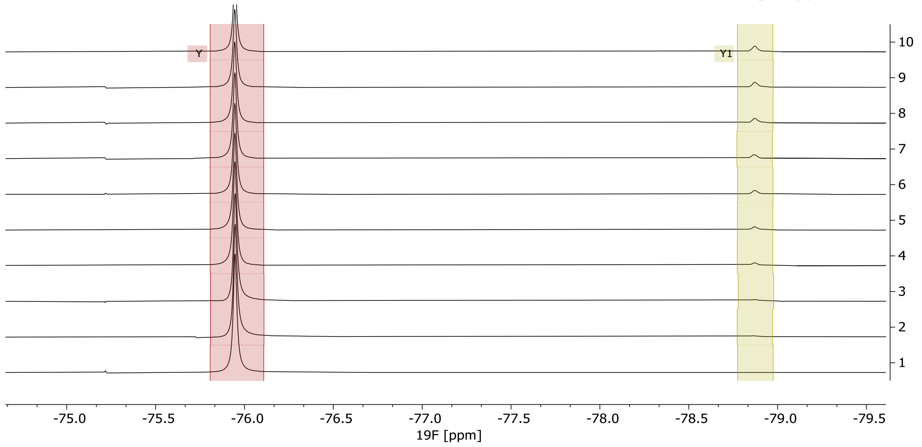

Figure S62: Raw ^{19}F EXSY data for the *α*-triflate dissociation in the corresponding *α*-triflate (left corner) measured at -45 °C.

1D Selective ^{19}F EXSY data at -40 ^{o}C

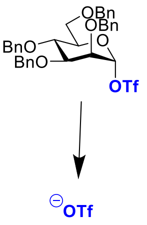

Spectrum number	Mixing Time [s]	α-triflate (-75.94 ppm) resonance intensity	⁻OTf (-78.86 ppm) resonance intensity
1	0.008	0.962537	0.0149878
2	0.016	0.9397	0.0269068
3	0.024	0.904049	0.0313806
4	0.032	0.872208	0.0338309
5	0.04	0.834912	0.0409511
6	0.048	0.800221	0.0492865
7	0.056	0.767243	0.0553768
8	0.064	0.734493	0.0590012
9	0.072	0.698991	0.0698274
10	0.08	0.671098	0.0681194

[⁻OTf] = 0.047 M

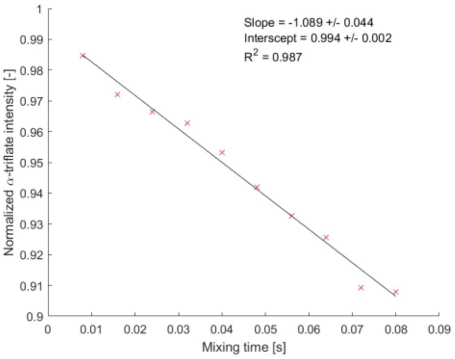

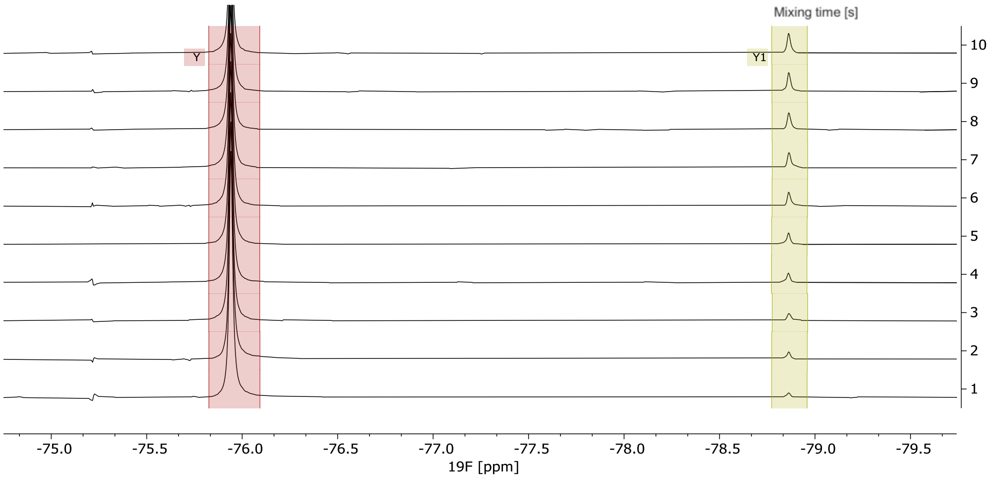

Figure S63: Raw ^{19}F EXSY data for the *α*-triflate dissociation in the corresponding *α*-triflate (left corner) measured at -40 °C.

1D Selective ^{19}F EXSY data at -40 ^{o}C

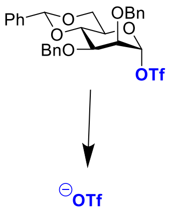

Spectrum number	Mixing Time [s]	*α*-triflate (-75.94 ppm) resonance intensity	$^{-}$OTf (-78.93 ppm) resonance intensity
1	0.05	12714.5	31.26
2	0.1	11022.91	115.57
3	0.2	9468.03	207.29
4	0.3	8069.46	246.79
5	0.4	7201.93	337.43
6	0.5	6077.04	360.11
7	0.6	4964.09	333.26
8	0.7	4434.46	428.77
9	0.8	3692.2	431

[$^{-}$OTf] = 0.05 M

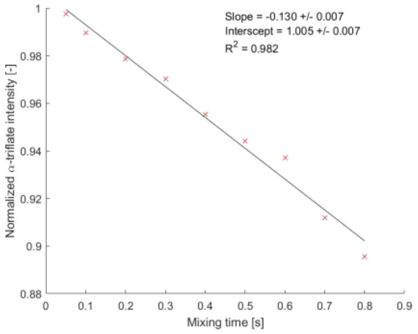

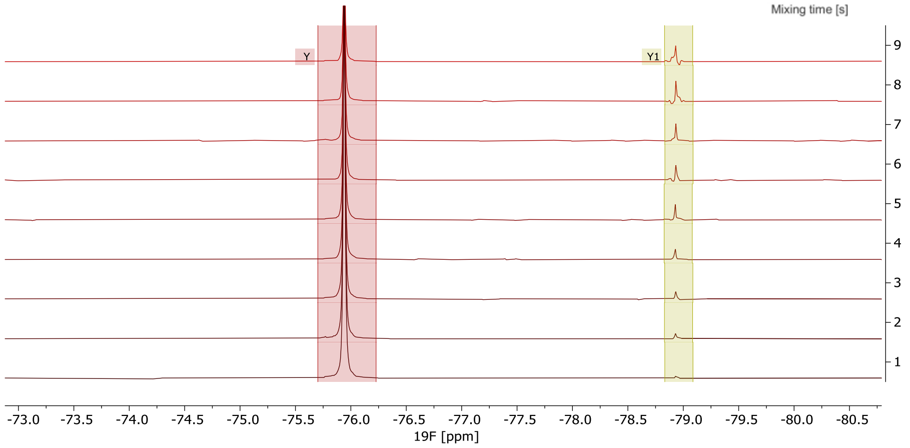

Figure S64: Raw ^{19}F EXSY data for the *α*-triflate dissociation in the corresponding *α*-triflate (left corner) measured at -40 °C.

1D Selective ^{19}F EXSY data at -35 °C

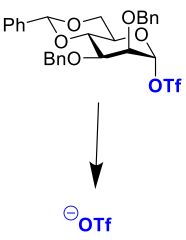

Spectrum number	Mixing Time [s]	*α*-triflate (75.95 ppm) resonance intensity	$^-$OTf (78.92 ppm) resonance intensity
1	0.02	18151.43	104.14
2	0.05	18101.93	240.79
3	0.1	15841.96	470.49
4	0.15	14164.43	651.29
5	0.2	13089.87	797.79
6	0.25	11545.92	930.2
7	0.3	10678.39	926.05
8	0.35	9934.11	1051.43
9	0.4	8561.88	1150.93

[$^-$OTf] = 0.04 M

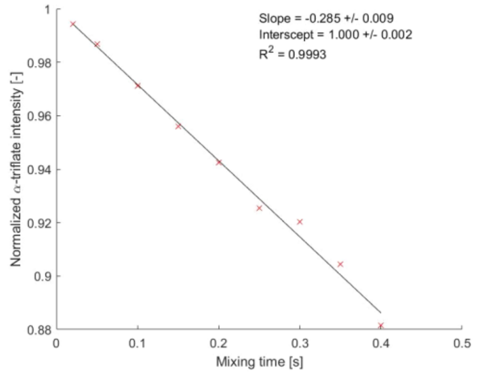

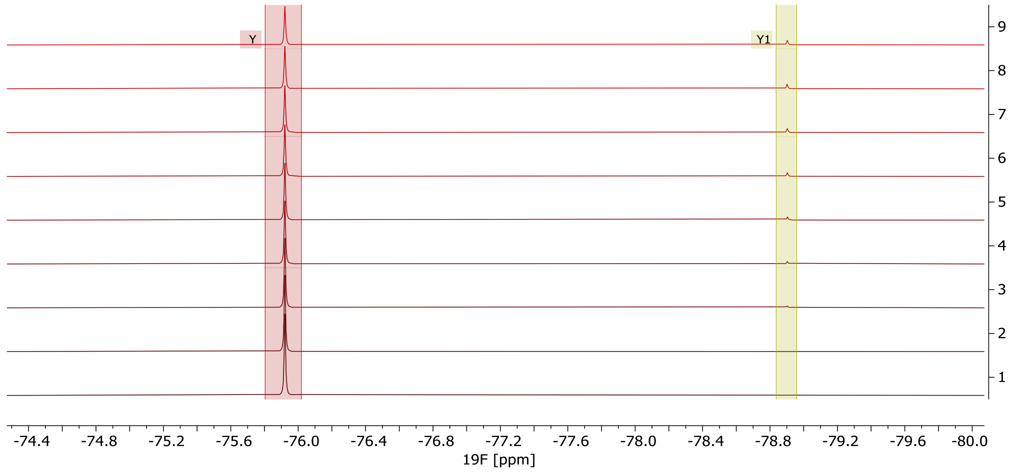

Figure S65: Raw ^{19}F EXSY data for the *α*-triflate dissociation in the corresponding *α*-triflate (left corner) measured at -35 °C.

1D Selective ^{19}F EXSY data at -30 °C

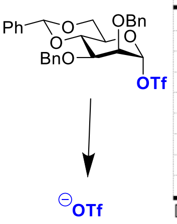

Spectrum number	Mixing Time [s]	*α*-triflate (75.95 ppm) resonance intensity	⁻OTf (78.92 ppm) resonance intensity
1	0.02	18151.43	104.14
2	0.05	18101.93	240.79
3	0.1	15841.96	470.49
4	0.15	14164.43	651.29
5	0.2	13089.87	797.79
6	0.25	11545.92	930.2
7	0.3	10678.39	926.05
8	0.35	9934.11	1051.43
9	0.4	8561.88	1150.93

[⁻OTf] = 0.04 M

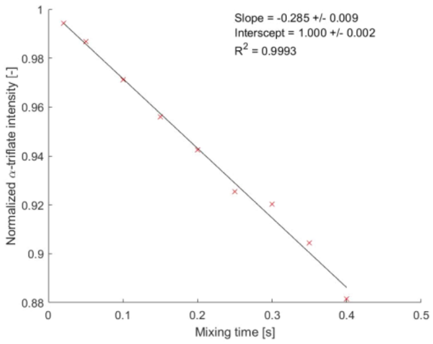

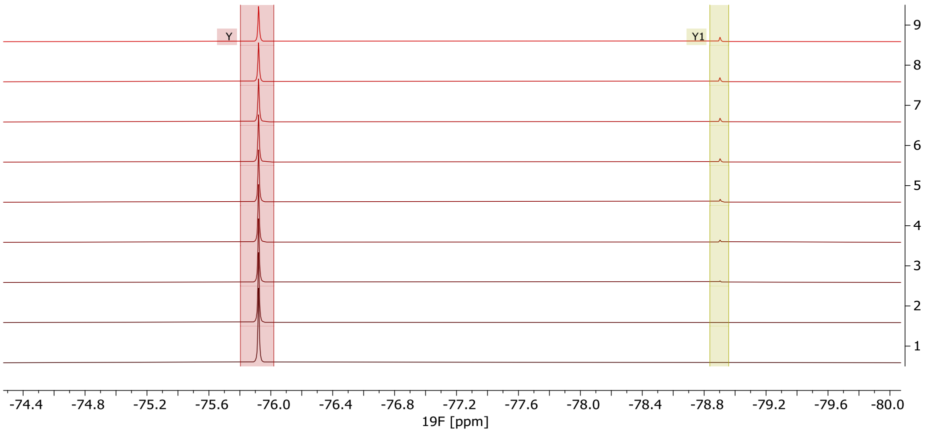

Figure S66: Raw ^{19}F EXSY data for the *α*-triflate dissociation in the corresponding *α*-triflate (left corner) measured at -30 °C.

1D Selective ^{19}F EXSY data at -20 °C

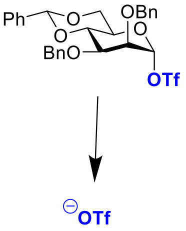

Spectrum number	Mixing Time [s]	*α*-triflate (-75.93 ppm) resonance intensity	⁻OTf (-78.89 ppm) resonance intensity
1	0.02	4173.68	60.31
2	0.04	4024.22	136.41
3	0.06	3988.02	143.74
4	0.08	3526.71	227.74
5	0.11	3269.78	278.78
6	0.14	3187.81	330.52
7	0.17	2861.11	338.58
8	0.2	2710.89	418.37

[⁻OTf] = 0.06 M

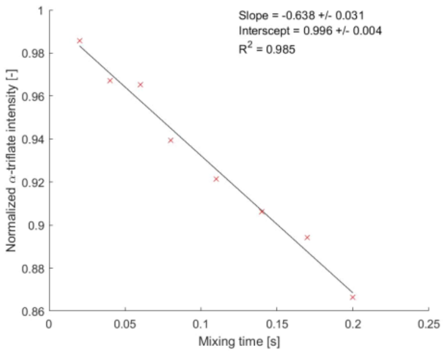

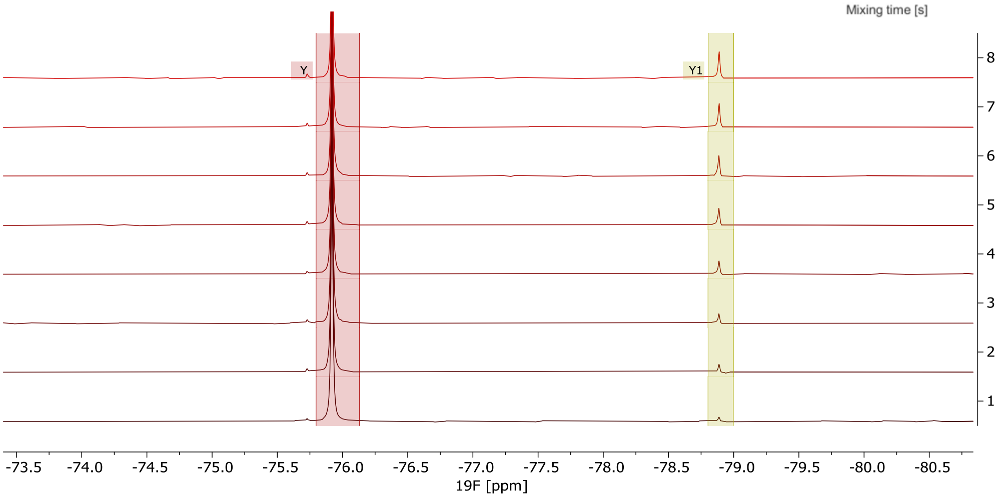

Figure S67: Raw ^{19}F EXSY data for the *α*-triflate dissociation in the corresponding *α*-triflate (left corner) measured at -20 °C.

1D Selective ^{19}F EXSY data at -15 ^{o}C

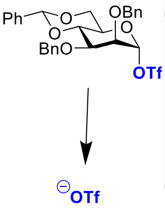

Spectrum number	Mixing Time [s]	*α*-triflate (-75.92 ppm) resonance intensity	$^{-}$OTf (-78.87 ppm) resonance intensity
1	0.005	2867.4	0
2	0.01	2831.24	16.61
3	0.02	2491.54	73.96
4	0.04	2418.21	116.46
5	0.06	2260	149.8
6	0.08	2090.6	201.66
7	0.09	1718.08	168.94
8	0.1	1883.69	222.66

[$^{-}$OTf] = 0.06 M

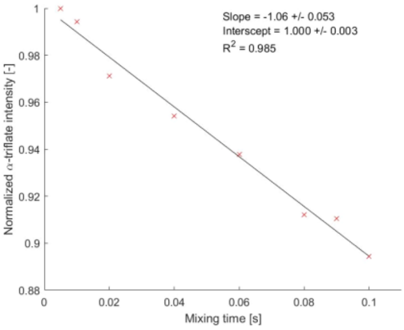

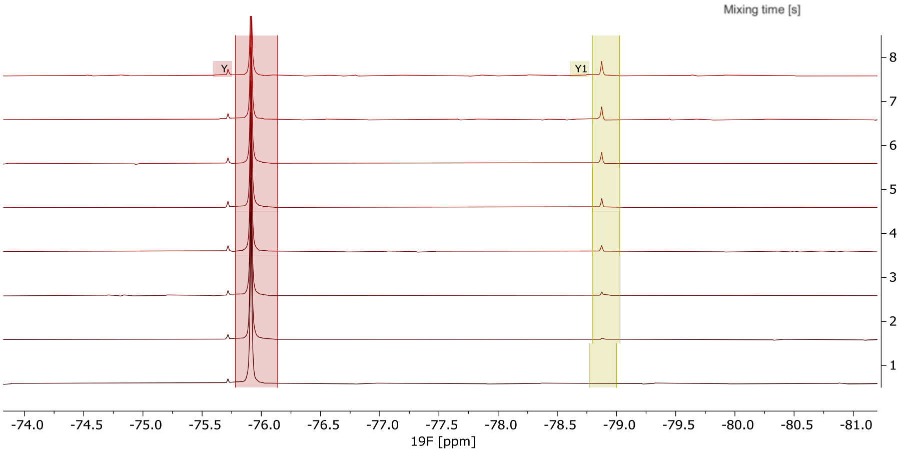

Figure S68: Raw ^{19}F EXSY data for the *α*-triflate dissociation in the corresponding *α*-triflate (left corner) measured at -15 °C.

1D Selective ^{19}F EXSY data at -60 ^{o}C

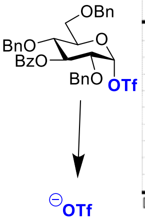

Spectrum number	Mixing Time [s]	*α*-triflate (-75.92 ppm) resonance intensity	$^{-}$OTf (-78.92 ppm) resonance intensity
1	0.1	7.18338	0.03266
2	0.2	5.28993	0.05117
3	0.3	3.85007	0.060531
4	0.4	2.82745	0.06363
5	0.5	2.07052	0.064164
6	0.6	1.51847	0.06146
7	0.7	1.11371	0.056238
8	0.8	0.821113	0.050563
9	0.9	0.610164	0.046966
10	1	0.44959	0.041262

[$^{-}$OTf] = 0.01 M

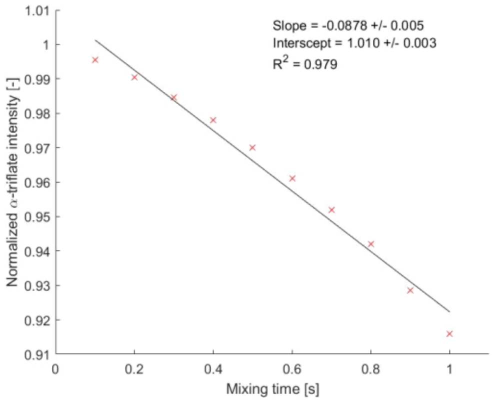

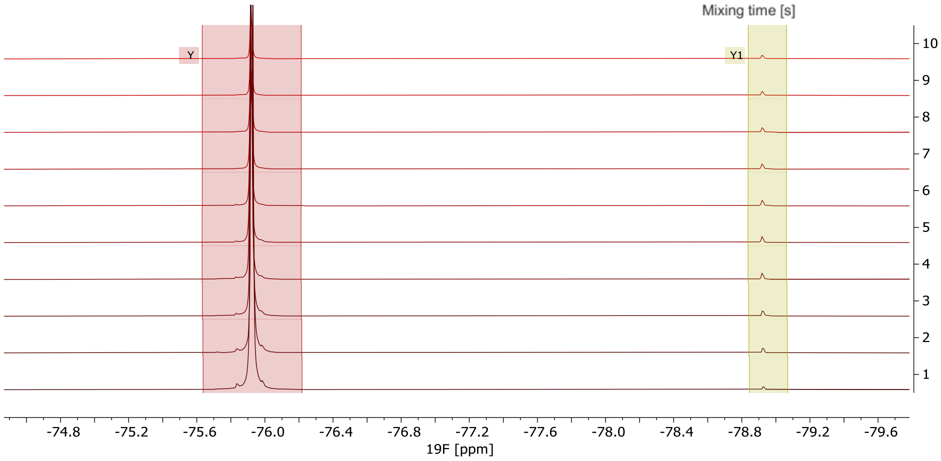

Figure S69: Raw ^{19}F EXSY data for the *α*-triflate dissociation in the corresponding *α*-triflate (left corner) measured at -60 °C.

1D Selective ^{19}F EXSY data at -55 °C

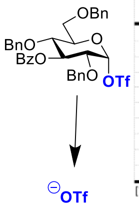

Spectrum number	Mixing Time [s]	α-triflate (-75.91 ppm) resonance intensity	⁻OTf (-78.91 ppm) resonance intensity
1	0.08	8.51462	0.049143
2	0.16	6.79363	0.081478
3	0.24	5.32194	0.100482
4	0.32	4.16009	0.110044
5	0.4	3.2622	0.113732
6	0.48	2.55826	0.112971
7	0.56	2.00189	0.111396
8	0.64	1.57023	0.10372
9	0.72	1.23524	0.097924
10	0.8	0.972992	0.090641

[⁻OTf] = 0.01 M

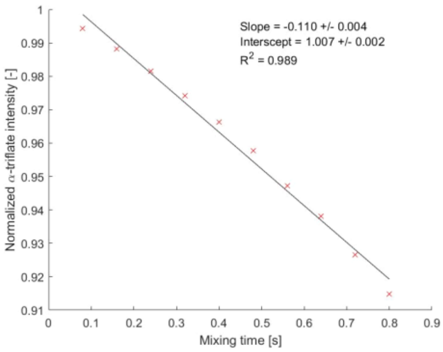

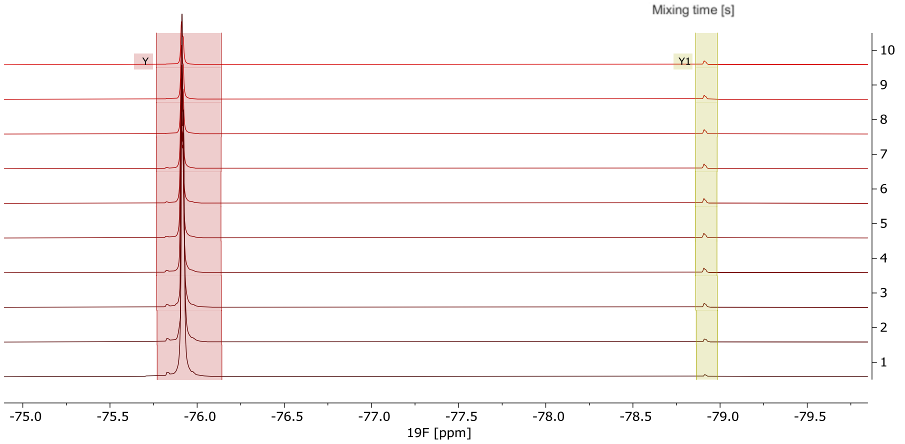

Figure S70: Raw ^{19}F EXSY data for the *α*-triflate dissociation in the corresponding *α*-triflate (left corner) measured at -55 °C.

1D Selective ^{19}F EXSY data at -50 °C

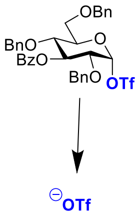

Spectrum number	Mixing Time [s]	α-triflate (-75.91 ppm) resonance intensity	⁻OTf (-78.91 ppm) resonance intensity
1	0.06	8.94225	0.072388
2	0.12	7.4093	0.123406
3	0.18	6.23294	0.158208
4	0.24	5.17662	0.18565
5	0.3	4.30124	0.200762
6	0.36	3.57549	0.208407
7	0.42	2.97169	0.212352
8	0.48	2.47668	0.210578
9	0.54	2.06808	0.207498
10	0.6	1.72057	0.199158

[⁻OTf] = 0.01 M

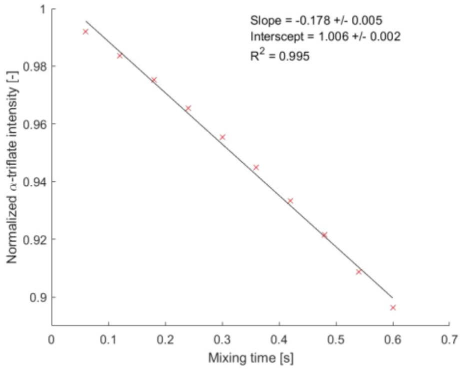

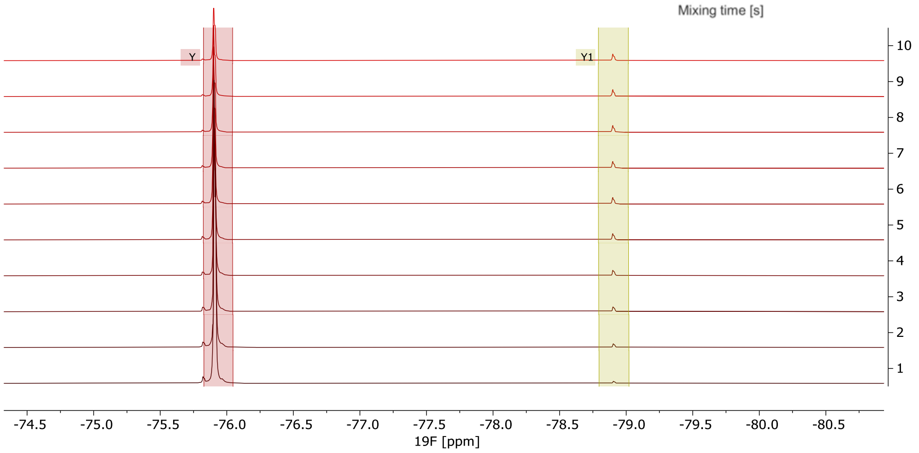

Figure S71: Raw ^{19}F EXSY data for the *α*-triflate dissociation in the corresponding *α*-triflate (left corner) measured at -50 °C.

1D Selective ^{19}F EXSY data at -45 °C

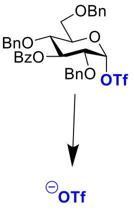

Spectrum number	Mixing Time [s]	α-triflate (-75.89 ppm) resonance intensity	⁻OTf (-78.89 ppm) resonance intensity
1	0.032	8.11976	0.046492
2	0.064	7.16503	0.101011
3	0.096	6.54661	0.168808
4	0.128	5.89123	0.195963
5	0.16	5.2709	0.235886
6	0.192	4.75415	0.263806
7	0.224	4.2469	0.285397
8	0.256	3.84083	0.308781
9	0.288	3.44268	0.323219
10	0.32	3.10713	0.335389

[⁻OTf] = 0.02 M

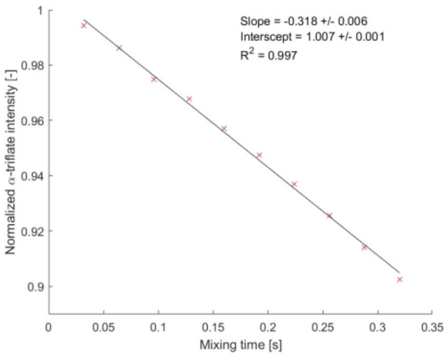

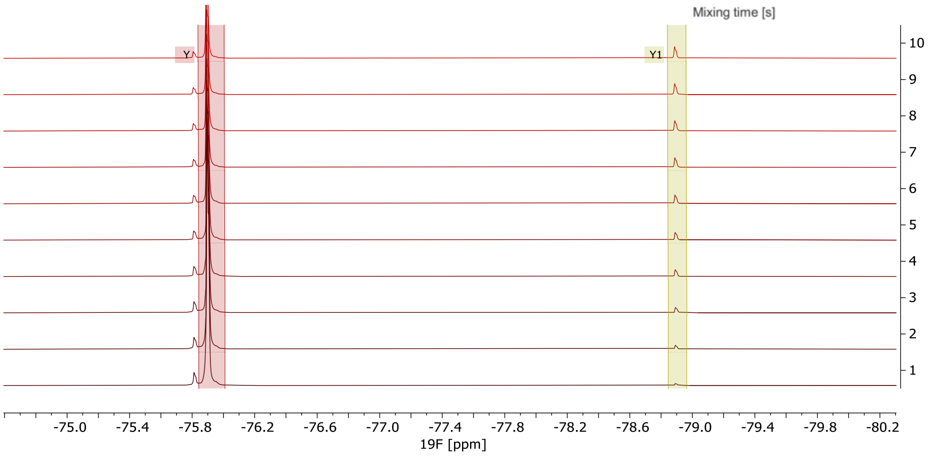

Figure S72: Raw ^{19}F EXSY data for the *α*-triflate dissociation in the corresponding *α*-triflate (left corner) measured at -45 °C.

1D Selective ^{19}F EXSY data at -40 °C

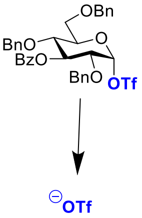

Spectrum number	Mixing Time [s]	α-triflate (-75.89 ppm) resonance intensity	⁻OTf (-78.88 ppm) resonance intensity
1	0.02	5.83222	0.066578
2	0.04	5.36345	0.125615
3	0.06	4.9471	0.179899
4	0.08	4.58302	0.223098
5	0.1	4.21629	0.265826
6	0.12	3.89026	0.300025
7	0.14	3.58799	0.331902
8	0.16	3.30543	0.361653
9	0.18	3.05897	0.383527
10	0.2	2.81281	0.402464

[⁻OTf] = 0.02 M

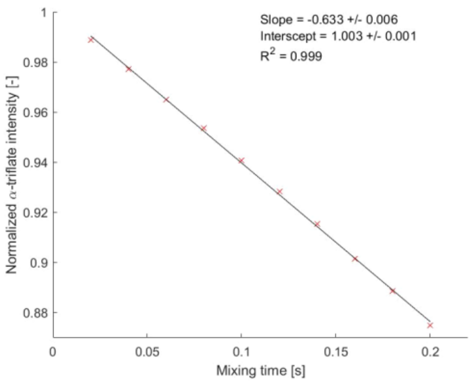

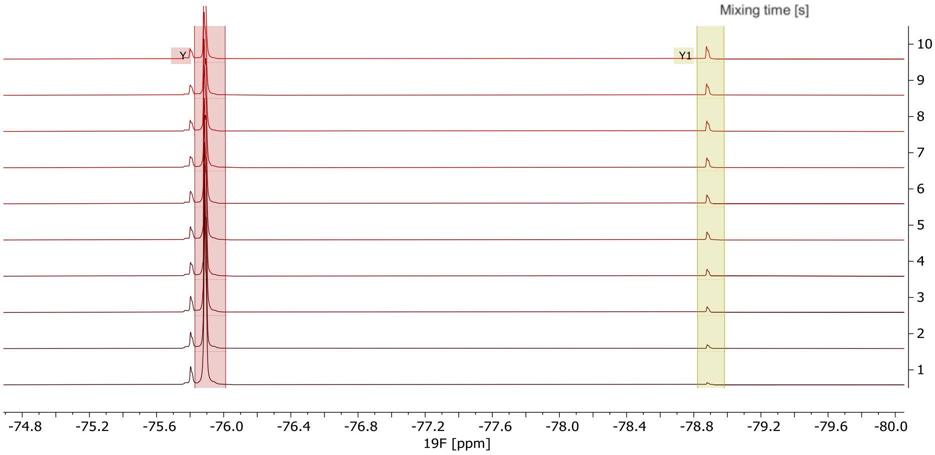

Figure S73: Raw ^{19}F EXSY data for the *α*-triflate dissociation in the corresponding *α*-triflate (left corner) measured at -40 °C.

1D Selective ^{19}F EXSY data at -40 ^{o}C

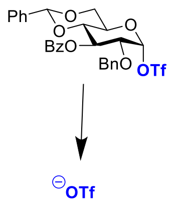

Spectrum number	Mixing Time [s]	*α*-triflate (-75.76 ppm) resonance intensity	$^{-}$OTf (-78.89 ppm) resonance intensity
1	0.1	8.4645	0.052814
2	0.2	6.55746	0.085382
3	0.3	5.09559	0.109116
4	0.4	3.97286	0.118407
5	0.5	3.10884	0.122953
6	0.6	2.42471	0.12262
7	0.7	1.88625	0.11707
8	0.8	1.47981	0.114862
9	0.9	1.14979	0.107589
10	1	0.907179	0.098785

[$^{-}$OTf] = 0.02 M

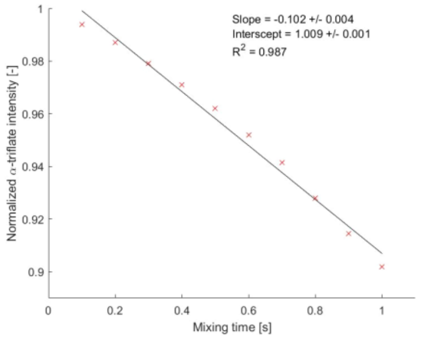

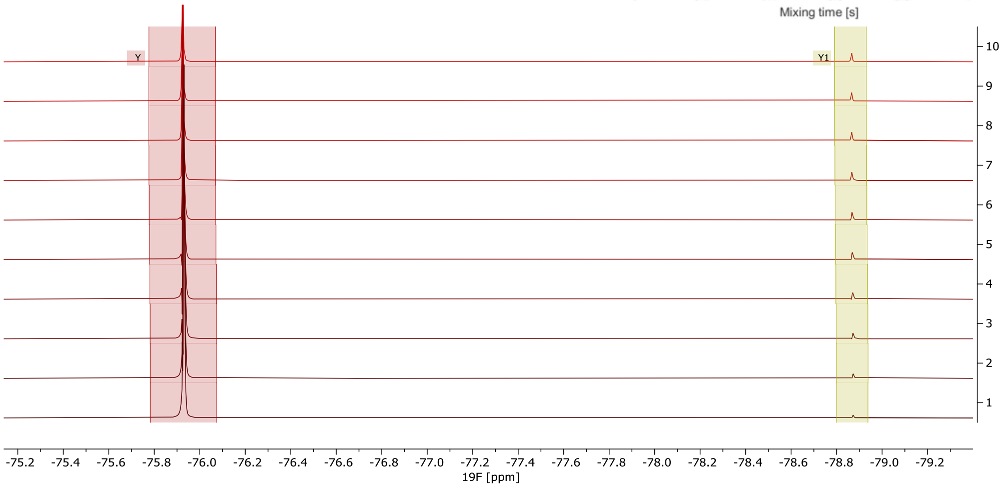

Figure S74: Raw ^{19}F EXSY data for the *α*-triflate dissociation in the corresponding *α*-triflate (left corner) measured at -40 °C.

1D Selective ^{19}F EXSY data at -30 ^{o}C

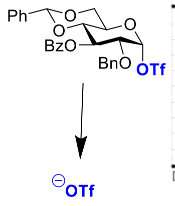

Spectrum number	Mixing Time [s]	*α*-triflate (-75.74 ppm) resonance intensity	$^{-}$OTf (-77.86 ppm) resonance intensity
1	0.045	9.33306	0.075098
2	0.09	8.25215	0.13411
3	0.135	7.43259	0.18819
4	0.18	6.79006	0.229035
5	0.225	6.00092	0.263531
6	0.27	5.46284	0.291409
7	0.315	4.90649	0.312375
8	0.36	4.42386	0.331695
9	0.405	3.9754	0.343393
10	0.45	3.59606	0.353458

[$^{-}$OTf] = 0.02 M

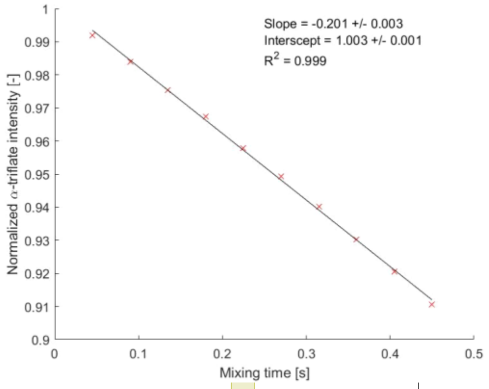

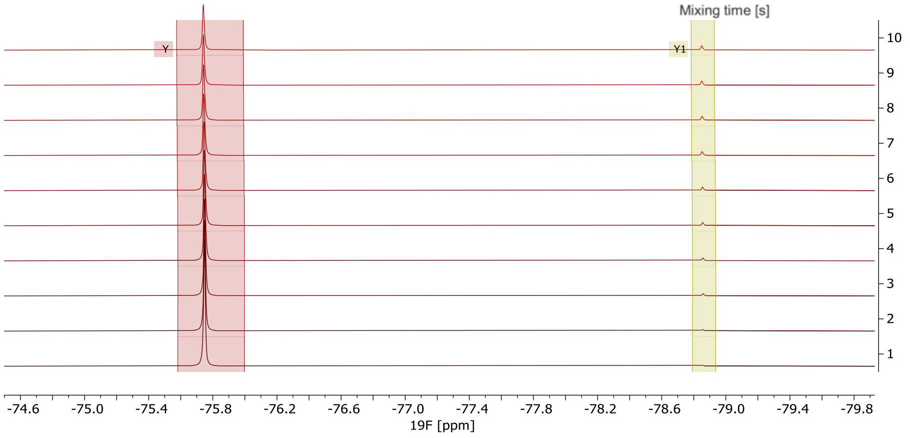

Figure S75: Raw ^{19}F EXSY data for the *α*-triflate dissociation in the corresponding *α*-triflate (left corner) measured at -30 °C.

1D Selective ^{19}F EXSY data at -20 ^{o}C

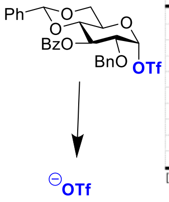

Spectrum number	Mixing Time [s]	*α*-triflate (-75.73 ppm) resonance intensity	$^{-}$OTf (-78.83 ppm) resonance intensity
1	0.016	7.53456	0.080451
2	0.032	7.23809	0.151461
3	0.048	6.93968	0.217912
4	0.064	6.65216	0.279102
5	0.08	6.34754	0.337773
6	0.096	6.03564	0.390601
7	0.112	5.74297	0.444613
8	0.128	5.46599	0.48802
9	0.144	5.21109	0.533055
10	0.16	4.97475	0.57345

[$^{-}$OTf] = 0.03 M

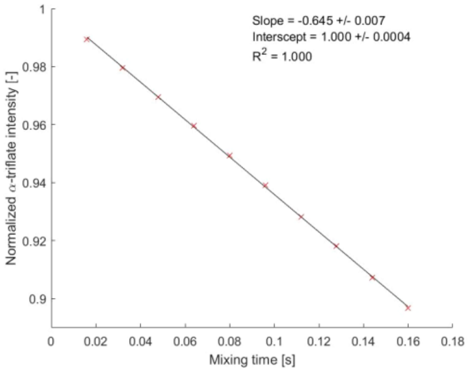

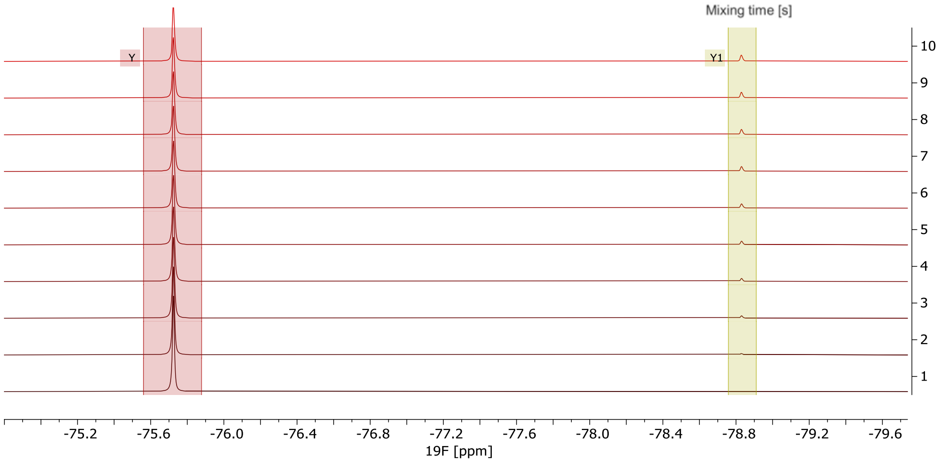

Figure S76: Raw ^{19}F EXSY data for the *α*-triflate dissociation in the corresponding *α*-triflate (left corner) measured at -20 °C.

1D Selective ^{19}F EXSY data at -10 ^{o}C

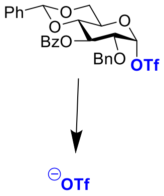

Spectrum number	Mixing Time [s]	*α*-triflate (-75.70 ppm) resonance intensity	$^{-}$OTf (-78.80 ppm) resonance intensity
1	0.0045	3.41044	0.034666
2	0.009	3.23797	0.069755
3	0.0135	3.06932	0.099565
4	0.018	2.908	0.127629
5	0.0225	2.76744	0.152986
6	0.027	2.62841	0.174127
7	0.0315	2.48608	0.204741
8	0.036	2.33857	0.218448
9	0.0405	2.19014	0.236547
10	0.045	2.06184	0.251762

[$^{-}$OTf] = 0.03 M

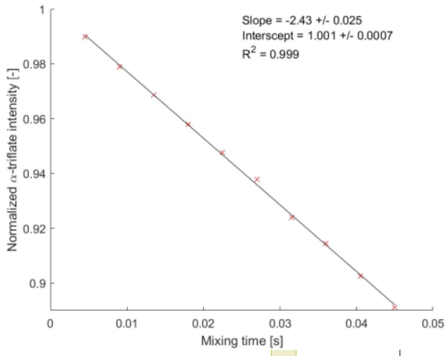

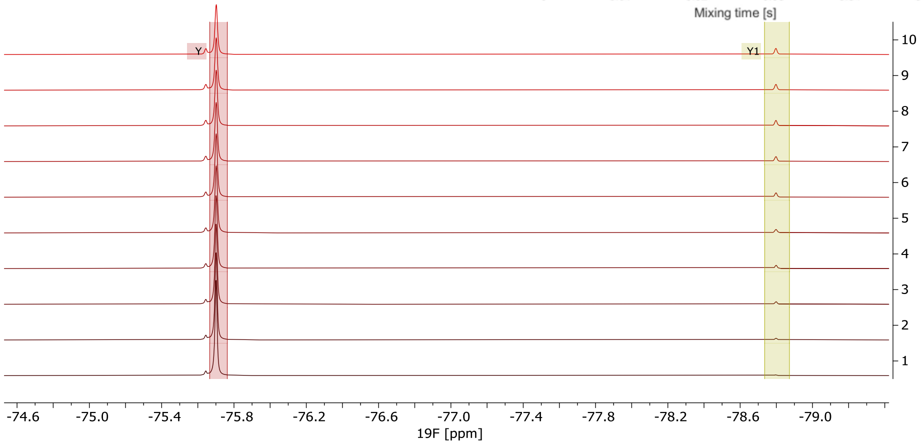

Figure S77: Raw ^{19}F EXSY data for the *α*-triflate dissociation in the corresponding *α*-triflate (left corner) measured at -10 °C.

1D Selective ^{19}F EXSY data at -60 ^{o}C

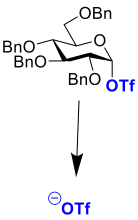

Spectrum number	Mixing Time [s]	α-triflate (-76.08 ppm) resonance intensity	⁻OTf (-78.92 ppm) resonance intensity
1	0.03	5.41019	0.044451
2	0.06	5.06077	0.076597
3	0.09	4.65011	0.110715
4	0.12	4.18142	0.136493
5	0.15	3.80856	0.158996
6	0.18	3.5006	0.178207
7	0.21	3.22318	0.194626
8	0.24	2.96677	0.205488
9	0.27	2.72069	0.226063
10	0.3	2.4781	0.22562

[⁻OTf] = 0.02 M

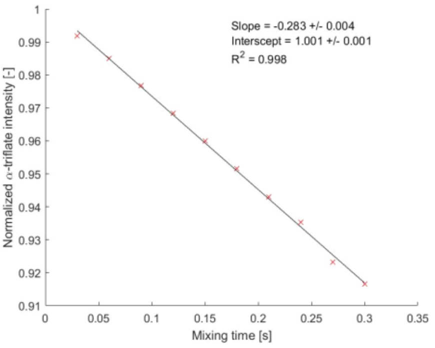

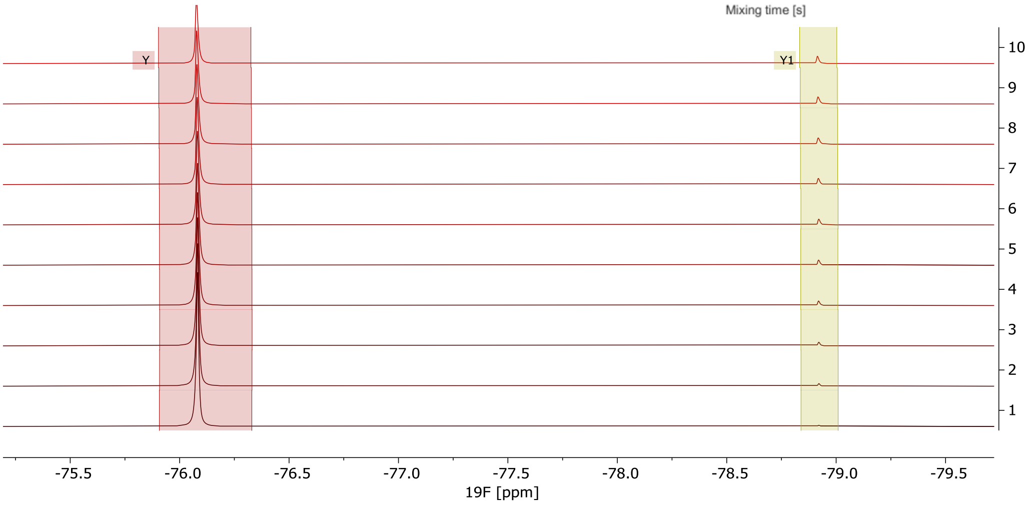

Figure S78: Raw ^{19}F EXSY data for the *α*-triflate dissociation in the corresponding *α*-triflate (left corner) measured at -60 °C.

1D Selective ^{19}F EXSY data at -55 ^{o}C

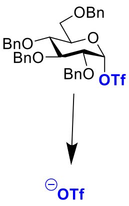

Spectrum number	Mixing Time [s]	α-triflate (-76.06 ppm) resonance intensity	⁻OTf (-78.90 ppm) resonance intensity
1	0.03	10.3697	0.09116
2	0.06	9.55088	0.170079
3	0.09	8.50568	0.236227
4	0.12	7.76823	0.28782
5	0.15	7.10008	0.340285
6	0.18	6.4331	0.375765
7	0.21	5.85645	0.403672
8	0.24	5.32198	0.428126
9	0.27	4.84298	0.445294
10	0.3	4.41724	0.460103

[⁻OTf] = 0.02 M

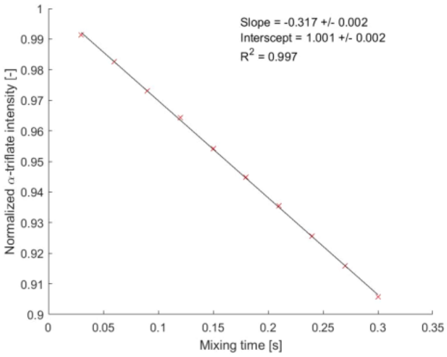

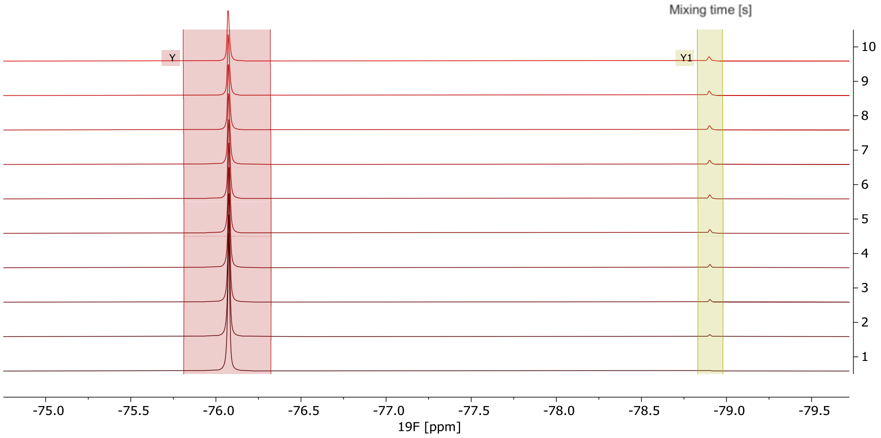

Figure S79: Raw ^{19}F EXSY data for the *α*-triflate dissociation in the corresponding *α*-triflate (left corner) measured at -55 °C.

1D Selective ^{19}F EXSY data at -50 °C

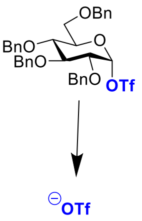

Spectrum number	Mixing Time [s]	α-triflate (-76.06 ppm) resonance intensity	⁻OTf (-78.88 ppm) resonance intensity
1	0.007	4.41173	0.046293
2	0.014	4.24788	0.097634
3	0.021	4.12023	0.142491
4	0.028	3.96573	0.184663
5	0.035	3.85141	0.22414
6	0.042	3.724	0.263587
7	0.049	3.63345	0.305263
8	0.056	3.53154	0.3353
9	0.063	3.39944	0.368015
10	0.07	3.298	0.397847

[⁻OTf] = 0.03 M

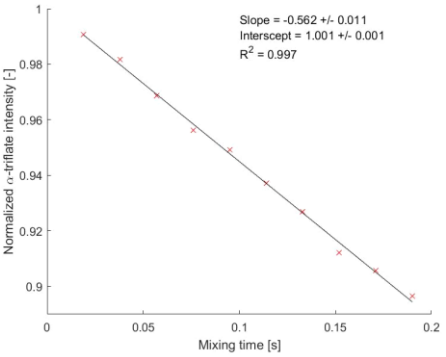

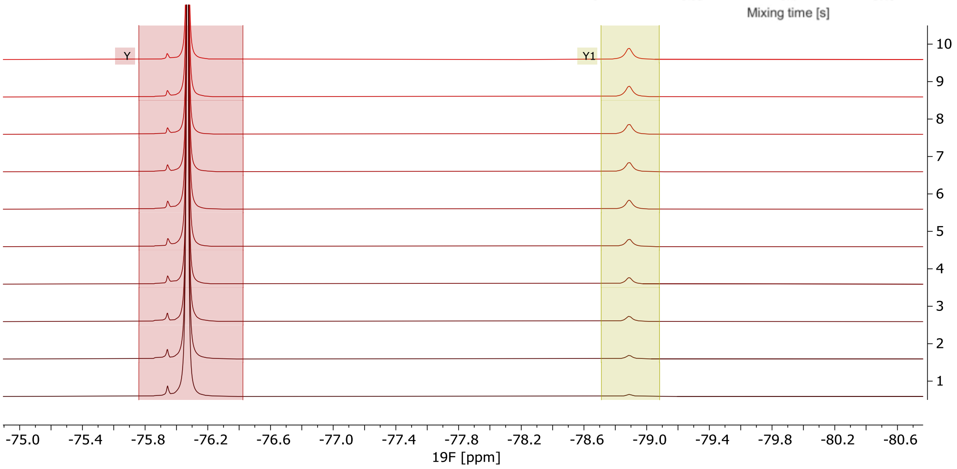

Figure S80: Raw ^{19}F EXSY data for the *α*-triflate dissociation in the corresponding *α*-triflate (left corner) measured at -50 °C.

1D Selective ^{19}F EXSY data at -45 °C

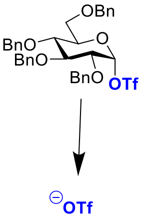

Spectrum number	Mixing Time [s]	α-triflate (-76.06 ppm) resonance intensity	⁻OTf (-78.88 ppm) resonance intensity
1	0.007	4.41173	0.046293
2	0.014	4.24788	0.097634
3	0.021	4.12023	0.142491
4	0.028	3.96573	0.184663
5	0.035	3.85141	0.22414
6	0.042	3.724	0.263587
7	0.049	3.63345	0.305263
8	0.056	3.53154	0.3353
9	0.063	3.39944	0.368015
10	0.07	3.298	0.397847

[⁻OTf] = 0.03 M

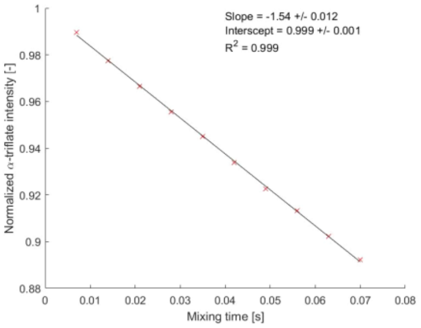

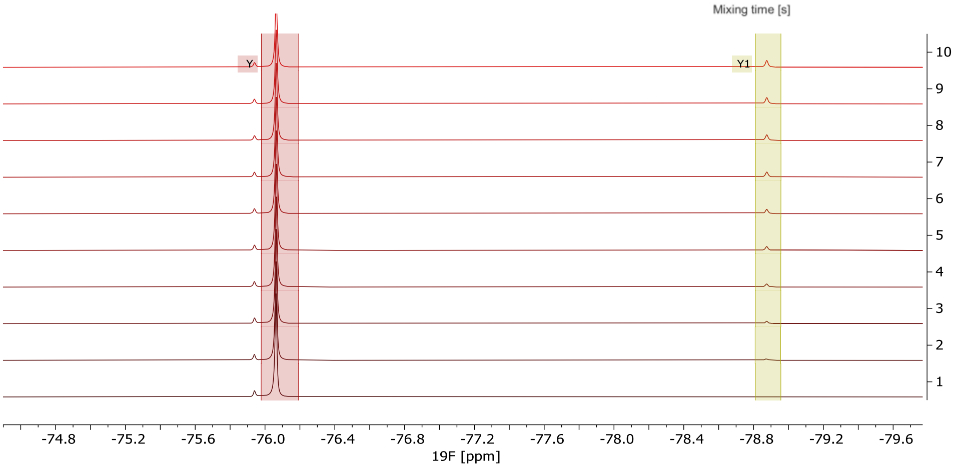

Figure S81: Raw ^{19}F EXSY data for the *α*-triflate dissociation in the corresponding *α*-triflate (left corner) measured at -45 °C.

1D Selective ^{19}F EXSY data at -40 ^{o}C

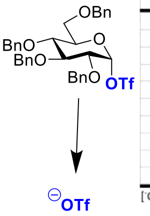

Spectrum number	Mixing Time [s]	α-triflate (-76.05 ppm) resonance intensity	⁻OTf (-78.86 ppm) resonance intensity
1	0.004	5.77992	0.051836
2	0.008	5.43689	0.10201
3	0.012	5.39252	0.160792
4	0.016	5.32338	0.200002
5	0.02	4.94569	0.253556
6	0.024	4.95619	0.295906
7	0.028	4.68124	0.337505
8	0.032	4.55786	0.379373
9	0.036	4.50947	0.417131
10	0.04	4.20437	0.450822

[⁻OTf] = 0.03 M

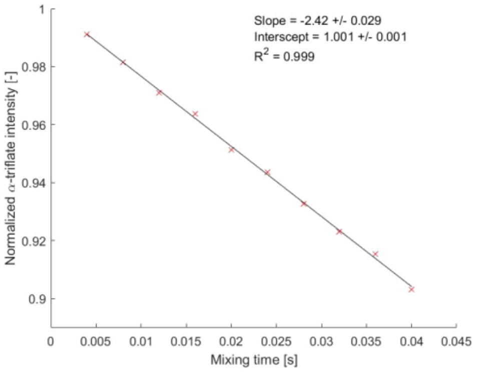

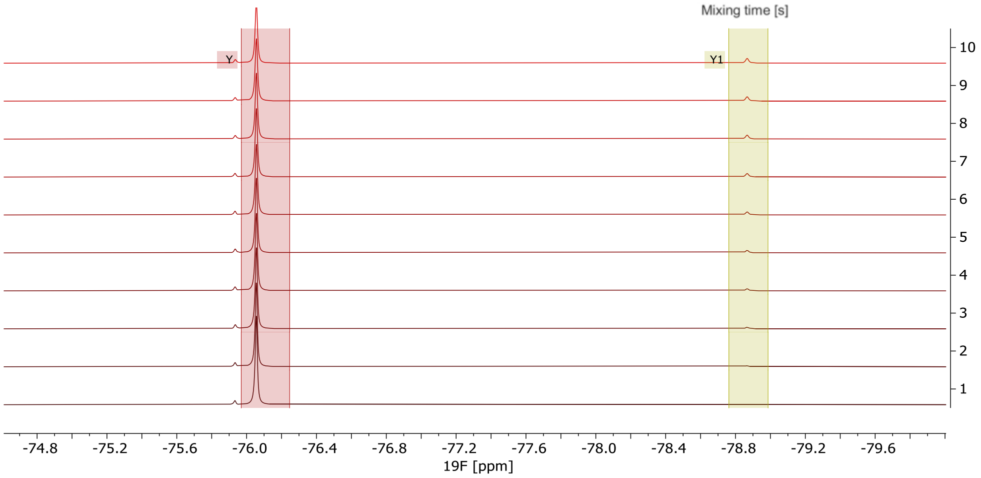

Figure S82: Raw ^{19}F EXSY data for the *α*-triflate dissociation in the corresponding *α*-triflate (left corner) measured at -40 °C.

1D Selective ^{19}F EXSY data at -40 ^{o}C

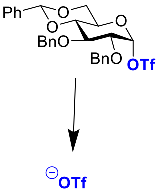

Spectrum number	Mixing Time [s]	α-triflate (-75.92 ppm) resonance intensity	⁻OTf (-78.87 ppm) resonance intensity
1	0.06	11.714	0.10203
2	0.12	10.1922	0.173606
3	0.18	8.78583	0.225102
4	0.24	7.58334	0.260833
5	0.3	6.52068	0.287092
6	0.36	5.618	0.301113
7	0.42	4.82853	0.309705
8	0.48	4.16962	0.316846
9	0.54	3.59117	0.312793
10	0.6	3.09109	0.30818

[⁻OTf] = 0.02 M

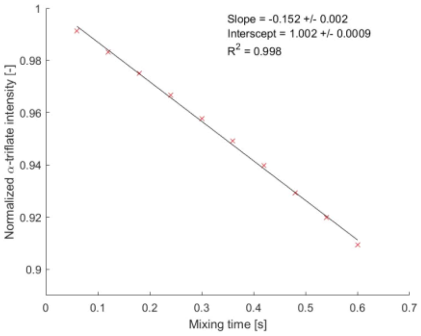

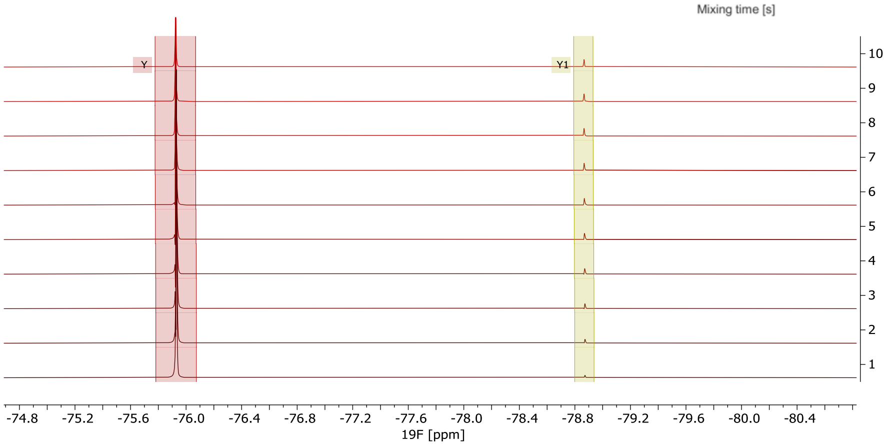

Figure S83: Raw ^{19}F EXSY data for the *α*-triflate dissociation in the corresponding *α*-triflate (left corner) measured at -40 °C.

1D Selective ^{19}F EXSY data at -30 °C

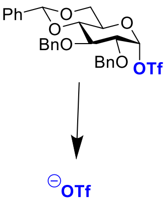

Spectrum number	Mixing Time [s]	α-triflate (-75.92 ppm) resonance intensity	⁻OTf (-78.84 ppm) resonance intensity
1	0.03	12.2404	0.134376
2	0.06	11.5912	0.247277
3	0.09	10.8918	0.34092
4	0.12	10.0854	0.420278
5	0.15	9.28039	0.493271
6	0.18	8.54498	0.557657
7	0.21	7.8597	0.612472
8	0.24	7.28612	0.657324
9	0.27	6.78737	0.699923
10	0.3	6.30957	0.732406

[⁻OTf] = 0.02 M

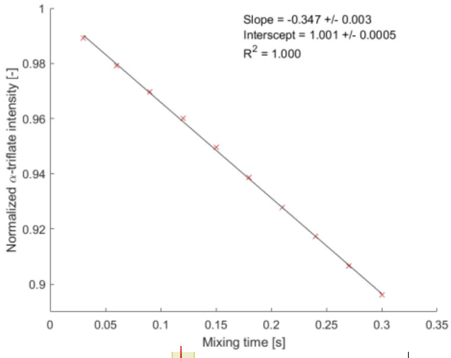

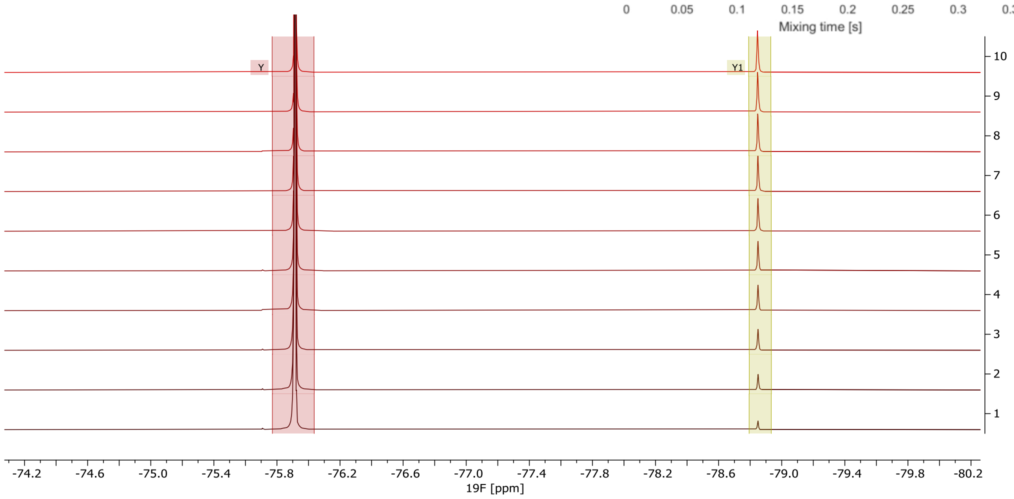

Figure S84: Raw ^{19}F EXSY data for the *α*-triflate dissociation in the corresponding *α*-triflate (left corner) measured at -30 °C.

1D Selective ^{19}F EXSY data at -20 °C

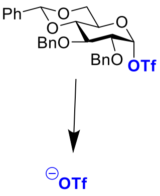

Spectrum number	Mixing Time [s]	α-triflate (-75.89 ppm) resonance intensity	⁻OTf (-78.82 ppm) resonance intensity
1	0.009	10.1519	0.120921
2	0.018	9.81255	0.236736
3	0.027	9.40949	0.347109
4	0.036	9.0213	0.44999
5	0.045	8.64699	0.548455
6	0.054	8.28105	0.643875
7	0.063	7.92225	0.731731
8	0.072	7.55815	0.816637
9	0.081	7.19294	0.892312
10	0.09	6.83547	0.967628

[⁻OTf] = 0.02 M

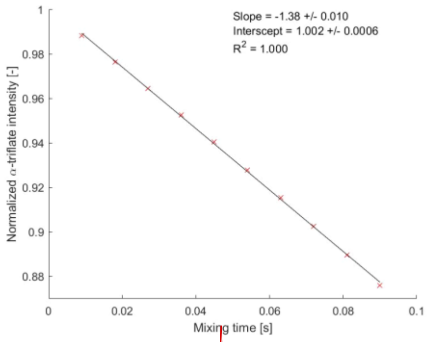

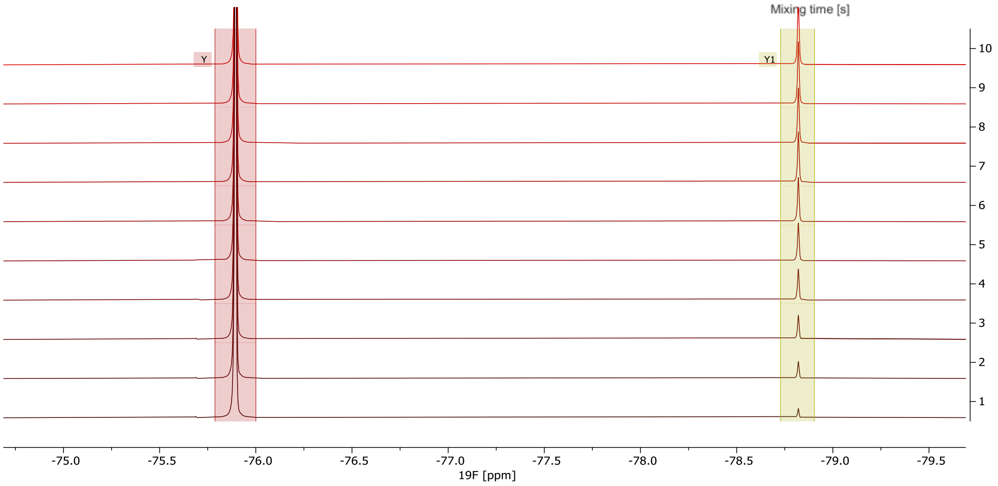

Figure S85: Raw ^{19}F EXSY data for the *α*-triflate dissociation in the corresponding *α*-triflate (left corner) measured at -20 °C.

1D Selective ^{19}F EXSY data at -10 ^{o}C

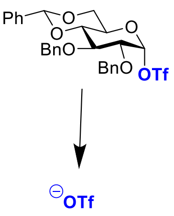

Spectrum number	Mixing Time [s]	*α*-triflate (-75.87 ppm) resonance intensity	^{-}OTf (-78.79 ppm) resonance intensity
1	0.002	3.56681	0.037999
2	0.004	3.26329	0.071633
3	0.006	3.18206	0.103312
4	0.008	2.9357	0.138367
5	0.01	2.81191	0.157652
6	0.012	2.63299	0.185938
7	0.014	2.47166	0.205758
8	0.016	2.35314	0.229034
9	0.018	2.17895	0.243853
10	0.02	2.1039	0.263628

[^{-}OTf] = 0.05 M

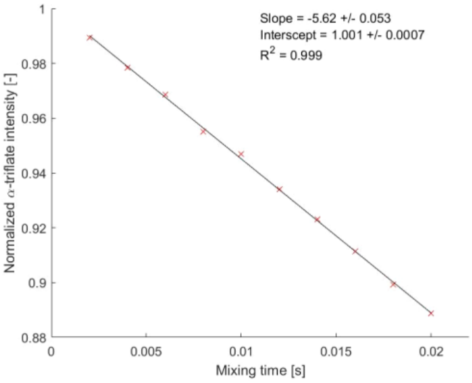

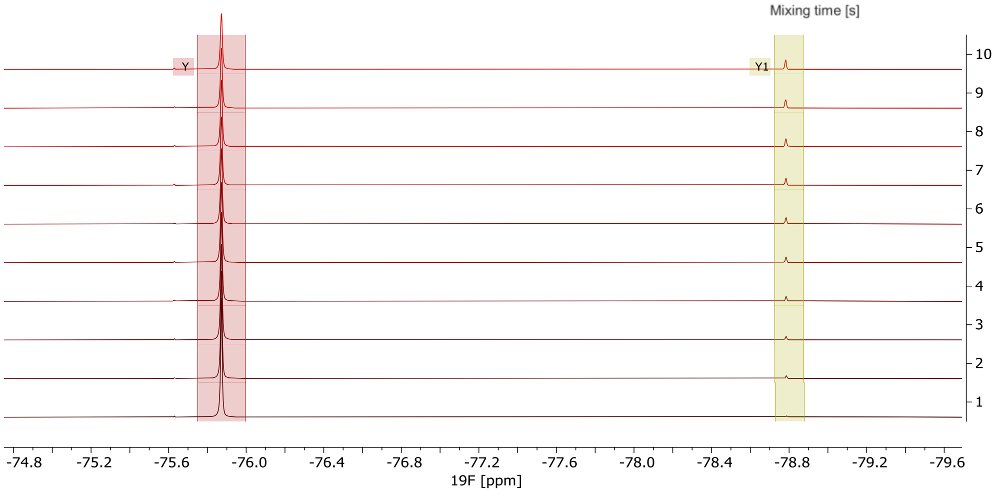

Figure S86: Raw ^{19}F EXSY data for the *α*-triflate dissociation in the corresponding *α*-triflate (left corner) measured at -10 °C.

Variable Bu_4NOTf concentration kinetic experiments:

1D Selective ^{13}C CEST data at [$^{-}$OTf] = 0.017 M

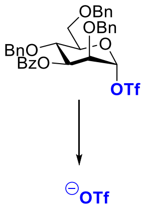

Spectrum number	Mixing Time [s]	α-triflate (-75.91 ppm) resonance intensity	$^{-}$OTf (-79.01 ppm) resonance intensity
1	0.03	30471.8	230.262
2	0.06	27586.3	478.467
3	0.09	25106.8	703.656
4	0.12	22822.4	860.979
5	0.15	20616.8	995.463
6	0.18	18847.3	1160.28
7	0.21	17220.8	1230.78
8	0.24	15614.4	1323.73
9	0.27	14225	1373.01
10	0.3	13071.1	1425.4

Temperature = -80 °C

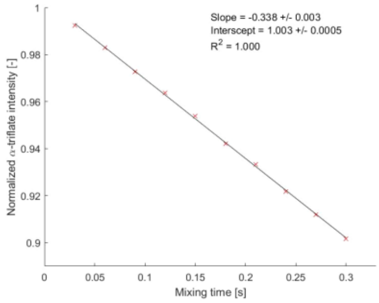

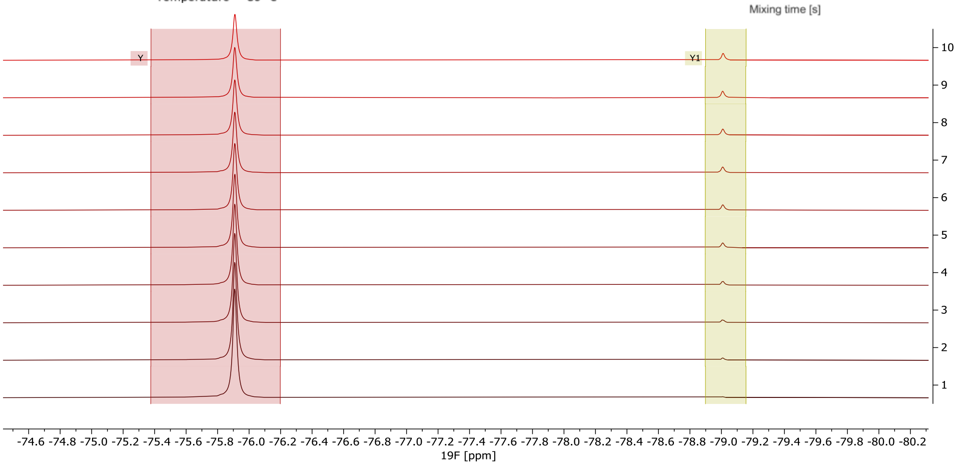

Figure S87: Raw ^{19}F EXSY data for the *α*-triflate dissociation in the corresponding *α*-triflate (left corner) measured at -80 °C at a concentration $^{-}$OTf of 0.017 M.

1D Selective ^{13}C CEST data at [$^{-}$OTf] = 0.094 M

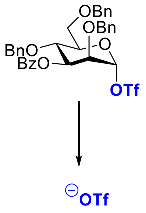

Spectrum number	Mixing Time [s]	α-triflate (-75.92 ppm) resonance intensity	$^{-}$OTf (-79.11 ppm) resonance intensity
1	0.027	17817.2	177.598
2	0.054	16263.9	313.909
3	0.081	14934.6	432.378
4	0.108	13528	525.945
5	0.135	12448.8	635.231
6	0.162	11413	756.613
7	0.189	10425.7	836.025
8	0.216	9603.38	915.791
9	0.243	8757.39	931.424
10	0.27	8071.78	959.686

Temperature = -80 °C

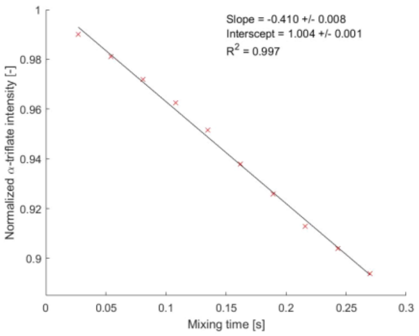

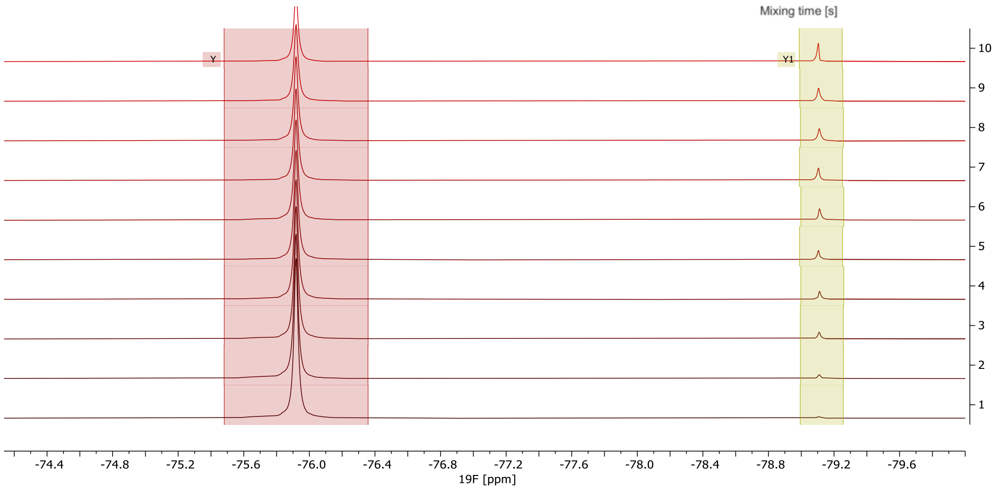

Figure S88: Raw ^{19}F EXSY data for the *α*-triflate dissociation in the corresponding *α*-triflate (left corner) measured at -80 °C at a concentration $^{-}$OTf of 0.094 M.

1D Selective ^{13}C CEST data at [$^{-}$OTf] = 0.16 M

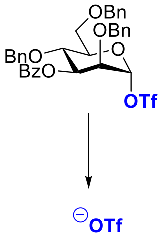

Spectrum number	Mixing Time [s]	α-triflate (-75.93 ppm) resonance intensity	$^{-}$OTf (-79.11 ppm) resonance intensity
1	0.027	12186.6	207.128
2	0.054	11152.1	294.155
3	0.081	10069.1	260.743
4	0.108	9270.05	389.42
5	0.135	8370.38	392.909
6	0.162	7711.66	473.377
7	0.189	7030.54	542.045
8	0.216	6440.67	526.671
9	0.243	5914.98	597.109
10	0.27	5476.32	599.298

Temperature = -80 °C

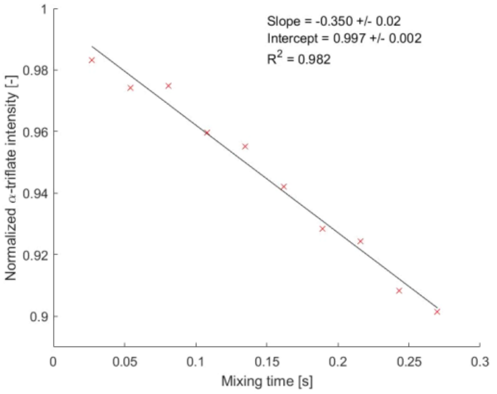

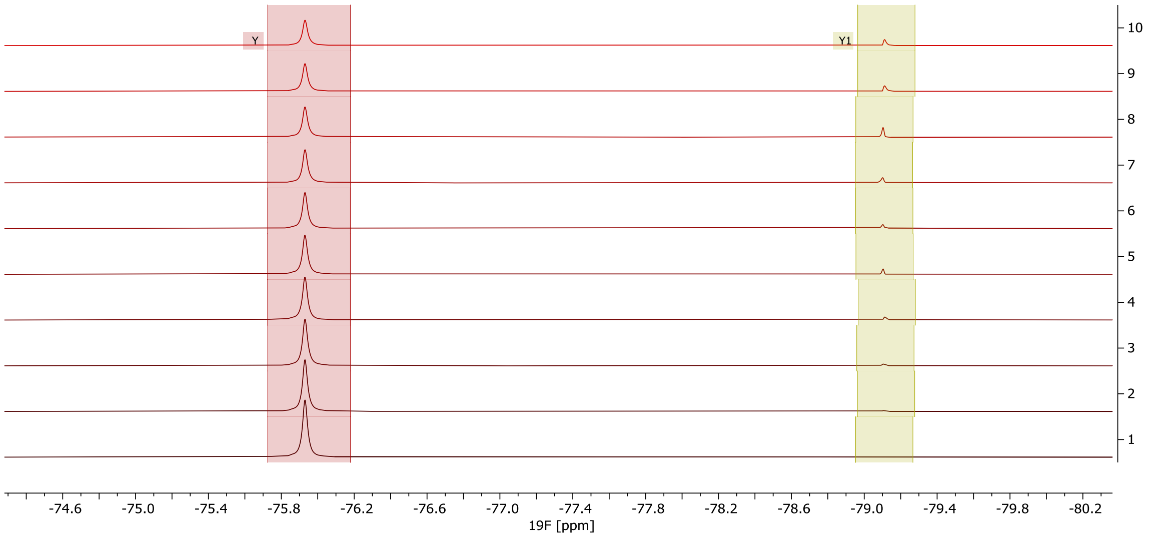

Figure S89: Raw ^{19}F EXSY data for the *α*-triflate dissociation in the corresponding *α*-triflate (left corner) measured at -80 °C at a concentration $^{-}$OTf of 0.16 M.

1D Selective ^{13}C CEST data at [$^{-}$OTf] = 0.017 M

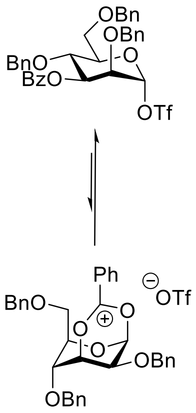

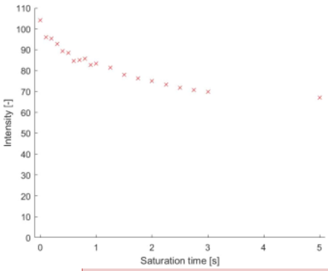

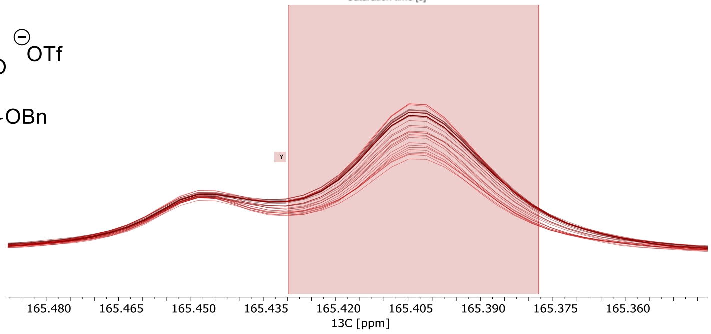

Spectrum number	Saturation time [s]	Intensity ^{13}C=O (165 ppm)
1	0.1	96.0635
2	0.2	95.3734
3	0.3	92.7285
4	0.4	89.4503
5	0.5	88.5271
6	0.6	84.6024
7	0.7	84.9696
8	0.8	85.6847
9	0.9	82.6564
10	1	83.3826
11	1.25	81.4853
12	1.5	77.936
13	1.75	76.2911
14	2	74.9725
15	2.25	73.3497
16	2.5	71.7329
17	2.75	70.7225
18	3	69.9591
19	5	67.1321
20	0	104.032

Temperature = -80 °C

Figure S90: Raw ^{13}C CEST data for the dioxanium ion formation from the corresponding *α*-triflate rate (left corner) measured at -80 °C at a concentration $^{-}$OTf of 0.017 M.

1D Selective ^{13}C CEST data at [$^{-}$OTf] = 0.094 M

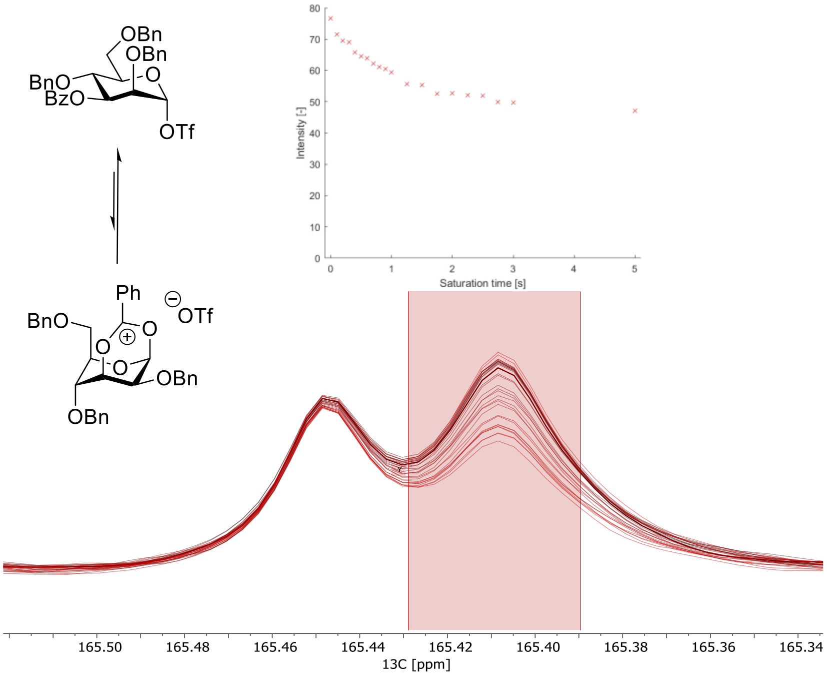

Spectrum number	Saturation time [s]	Intensity ^{13}C=O (165 ppm)
1	0.1	71.4534
2	0.2	69.5089
3	0.3	69.0094
4	0.4	65.6832
5	0.5	64.4924
6	0.6	63.8822
7	0.7	62.235
8	0.8	61.0185
9	0.9	60.4857
10	1	59.3336
11	1.25	55.6619
12	1.5	55.4002
13	1.75	52.468
14	2	52.6213
15	2.25	52.0092
16	2.5	51.971
17	2.75	49.8658
18	3	49.6645
19	5	47.1377
20	0	76.6437

Temperature = -80 °C

Figure S91: Raw ^{13}C CEST data for the dioxanium ion formation from the corresponding *α*-triflate rate (left corner) measured at -80 °C at a concentration $^{-}$OTf of 0.094 M.

1D Selective ^{13}C CEST data at [$^{-}$OTf] = 0.16 M

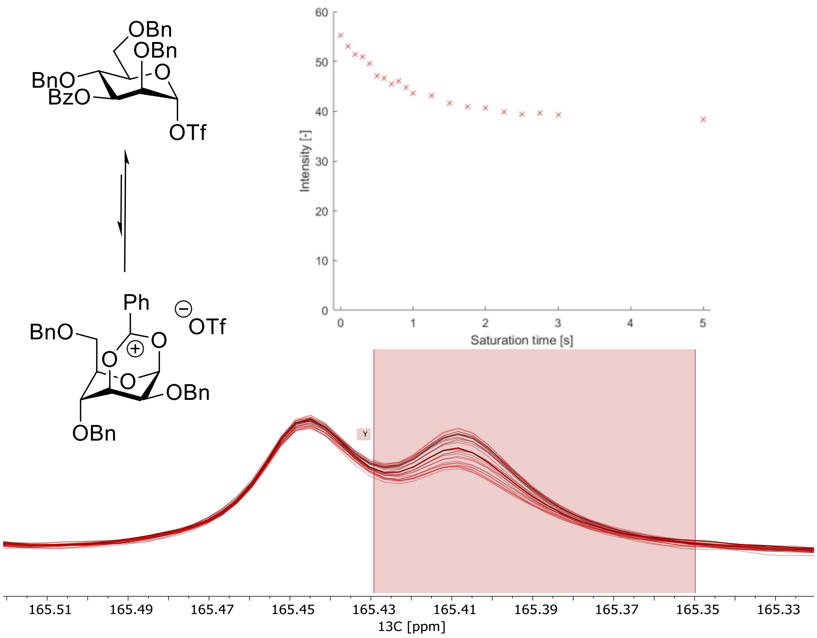

Spectrum number	Saturation time [s]	Intensity ^{13}C=O (165 ppm)
1	0.1	53.0877
2	0.2	51.4592
3	0.3	50.9597
4	0.4	49.4973
5	0.5	47.1302
6	0.6	46.5774
7	0.7	45.4907
8	0.8	46.0655
9	0.9	44.7084
10	1	43.5849
11	1.25	43.0875
12	1.5	41.5709
13	1.75	40.9343
14	2	40.6305
15	2.25	39.8198
16	2.5	39.362
17	2.75	39.5739
18	3	39.2455
19	5	38.3222
20	0	55.2778

Temperature = -80 °C

Figure S92: Raw ^{13}C CEST data for the dioxanium ion formation from the corresponding *α*-triflate rate (left corner) measured at -80 °C at a concentration $^{-}$OTf of 0.16 M.

1D Selective ^{19}F EXSY data at [$^{-}$OTf] = 0.011 M

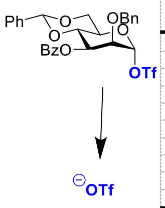

Spectrum number	Mixing Time [s]	α-triflate (-75.81 ppm) resonance intensity	$^{-}$OTf (-78.99 ppm) resonance intensity
1	0.04	35560	363.069
2	0.08	32151.2	593.373
3	0.12	29010.6	821.536
4	0.16	26136.5	1034.7
5	0.2	23667.7	1207.56
6	0.24	21402.7	1308.46
7	0.28	19401	1397
8	0.32	17597.4	1484.02
9	0.36	16034.2	1546.36
10	0.4	14608.2	1623.79

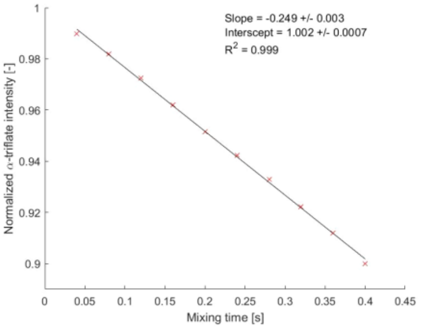

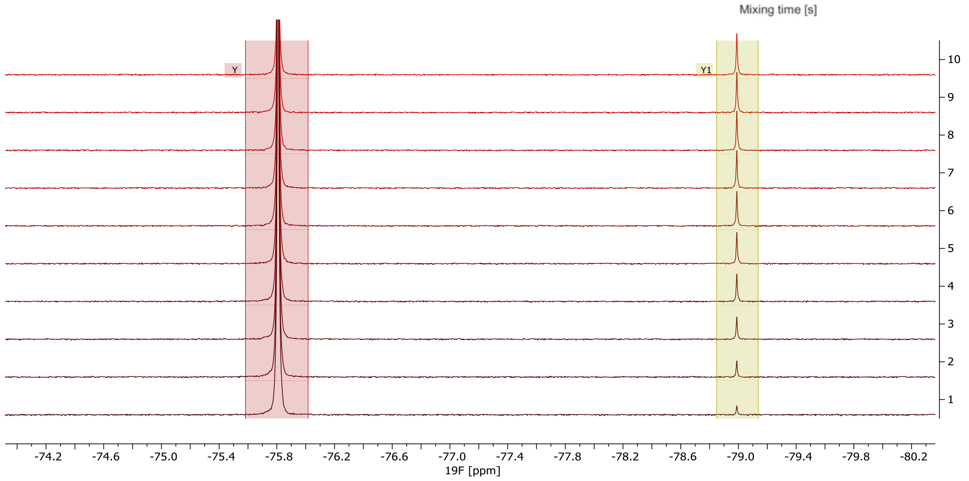

Figure S93: Raw ^{19}F EXSY data for the *α*-triflate dissociation in the corresponding *α*-triflate (left corner) measured at -60 °C at a concentration $^{-}$OTf of 0.011 M.

1D Selective ^{19}F EXSY data at [$^{-}$OTf] = 0.043 M

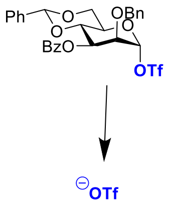

Spectrum number	Mixing Time [s]	α-triflate (-75.81 ppm) resonance intensity	$^{-}$OTf (-79.10 ppm) resonance intensity
1	0.03	33550.8	299.496
2	0.06	31272	549.949
3	0.09	28933	743.605
4	0.12	26633.2	992.253
5	0.15	24568.9	1112.53
6	0.18	22701	1301.76
7	0.21	21023	1439.22
8	0.24	19375.4	1564.02
9	0.27	17948.6	1687.43
10	0.3	16550.7	1780.75

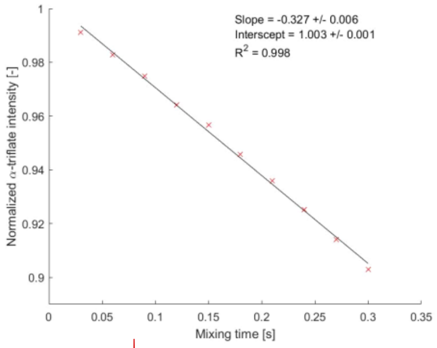

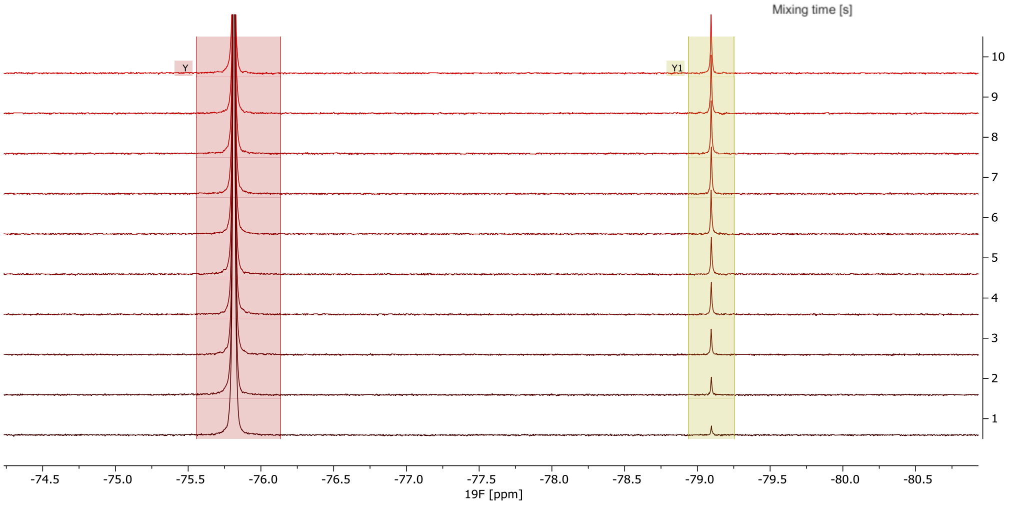

Figure S94: Raw ^{19}F EXSY data for the *α*-triflate dissociation in the corresponding *α*-triflate (left corner) measured at -60 °C at a concentration $^{-}$OTf of 0.043 M.

1D Selective ^{19}F EXSY data at [$^{-}$OTf] = 0.084 M

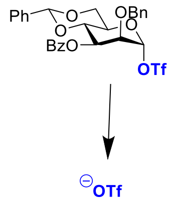

Spectrum number	Mixing Time [s]	*α*-triflate (-75.82 ppm) resonance intensity	$^{-}$OTf (-79.10 ppm) resonance intensity
1	0.03	29872.6	318.431
2	0.06	27706.9	517.769
3	0.09	25529.4	755.397
4	0.12	23420.5	966.743
5	0.15	21606.7	1082.05
6	0.18	19931.2	1245.82
7	0.21	18466.2	1407.62
8	0.24	17003.9	1526.19
9	0.27	15649.9	1591.84
10	0.3	14468.9	1722.53

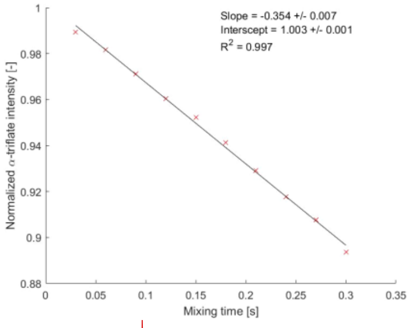

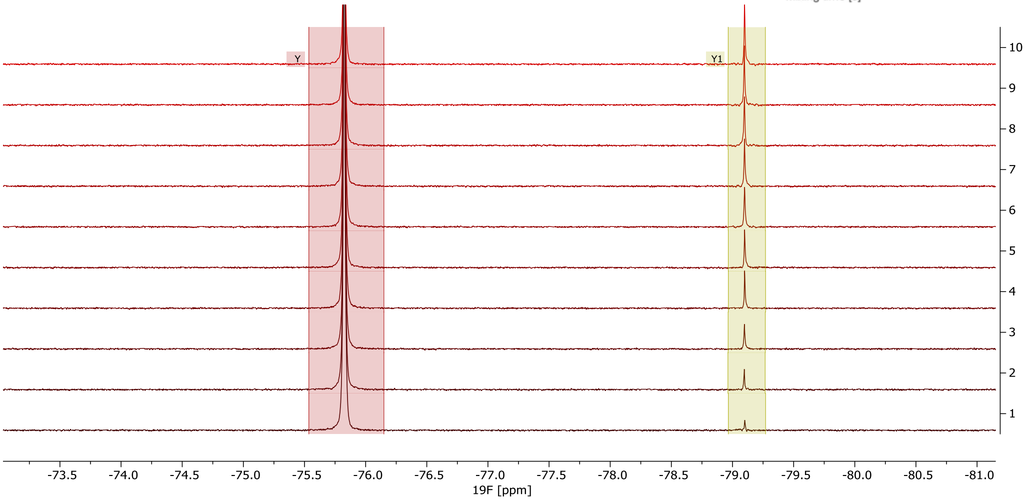

Figure S95: Raw ^{19}F EXSY data for the *α*-triflate dissociation in the corresponding *α*-triflate (left corner) measured at -60 °C at a concentration $^{-}$OTf of 0.084 M.

1D Selective ^{19}F EXSY data at [$^{-}$OTf] = 0.15 M

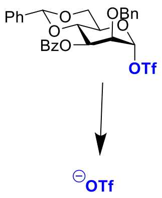

Spectrum number	Mixing Time [s]	*α*-triflate (-75.84 ppm) resonance intensity	$^{-}$OTf (-79.09 ppm) resonance intensity
1	0.03	25998.9	185.123
2	0.06	24003.5	470.733
3	0.09	22020.5	675.303
4	0.12	20207.2	886.726
5	0.15	18506.3	1013.77
6	0.18	17016.5	1169.77
7	0.21	15615.8	1294.84
8	0.24	14416.9	1316.28
9	0.27	13289.5	1489.61
10	0.3	12221.1	1560.7

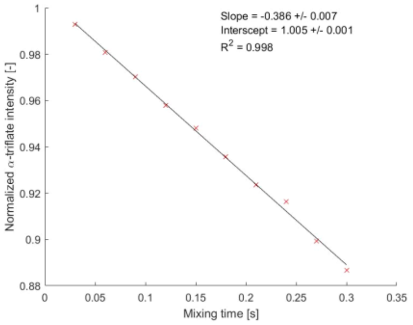

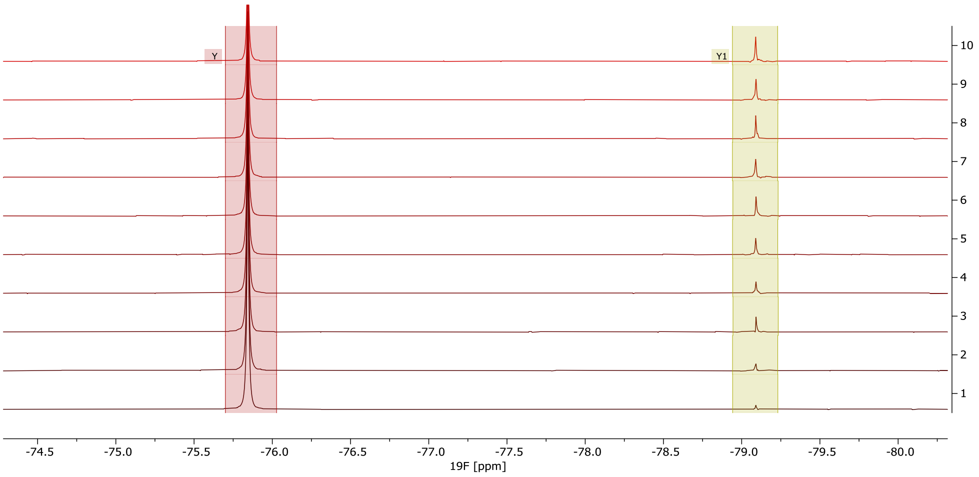

Figure S96: Raw ^{19}F EXSY data for the *α*-triflate dissociation in the corresponding *α*-triflate (left corner) measured at -60 °C at a concentration $^{-}$OTf of 0.15 M.

1D Selective ^{19}F EXSY data at [$^-$OTf] = 0.012 M

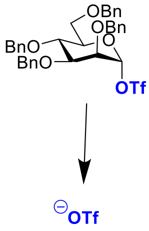

Spectrum number	Mixing Time [s]	*α*-triflate (-76.05 ppm) resonance intensity	$^-$OTf (-78.96 ppm) resonance intensity
1	0.03	26864.6	252.808
2	0.06	24927.1	495.588
3	0.09	23051.1	725.401
4	0.12	21221.5	906.099
5	0.15	19635.2	1050.34
6	0.18	18059.3	1184.34
7	0.21	16719.1	1307.7
8	0.24	15531.9	1405.75
9	0.27	14301.5	1514.33
10	0.3	13215.8	1528.9

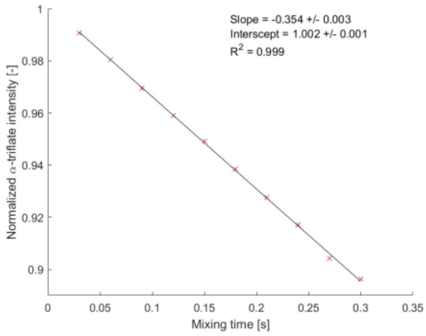

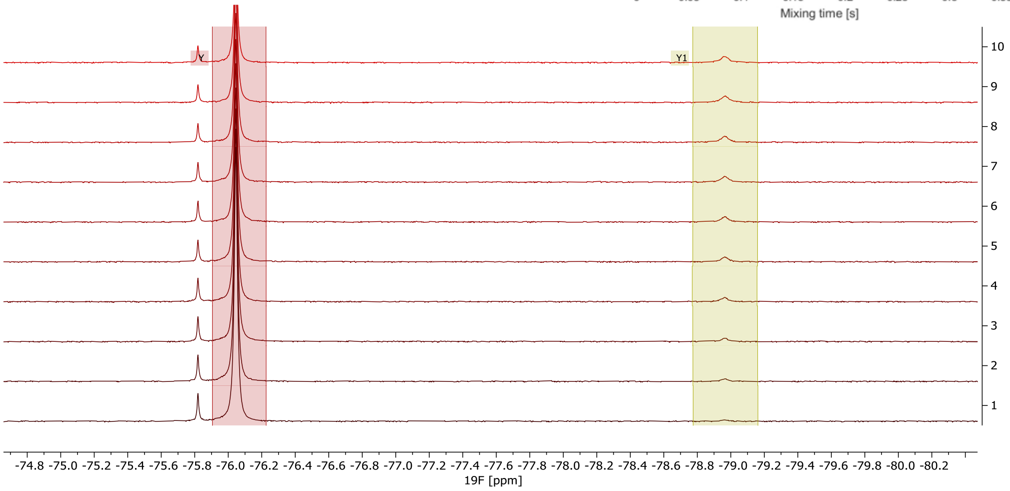

Figure S97: Raw ^{19}F EXSY data for the *α*-triflate dissociation in the corresponding *α*-triflate (left corner) measured at -50 °C at a concentration $^-$OTf of 0.012 M.

1D Selective ^{19}F EXSY data at [$^-$OTf] = 0.046 M

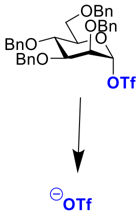

Spectrum number	Mixing Time [s]	*α*-triflate (-76.05 ppm) resonance intensity	$^-$OTf (-79.06 ppm) resonance intensity
1	0.009	14240.2	133.17
2	0.018	13720.1	325.428
3	0.027	12919.4	418.014
4	0.036	12659.4	554.838
5	0.045	12366.2	680.332
6	0.054	11835.4	756.478
7	0.063	11357.2	913.054
8	0.072	10899.3	1016.14
9	0.081	10411	1065.49
10	0.09	10066.3	1231.38

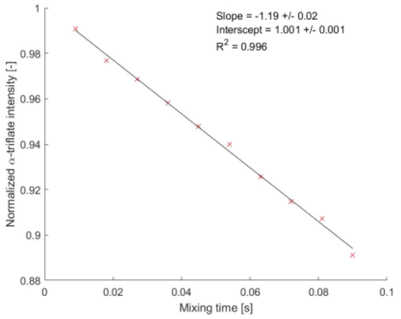

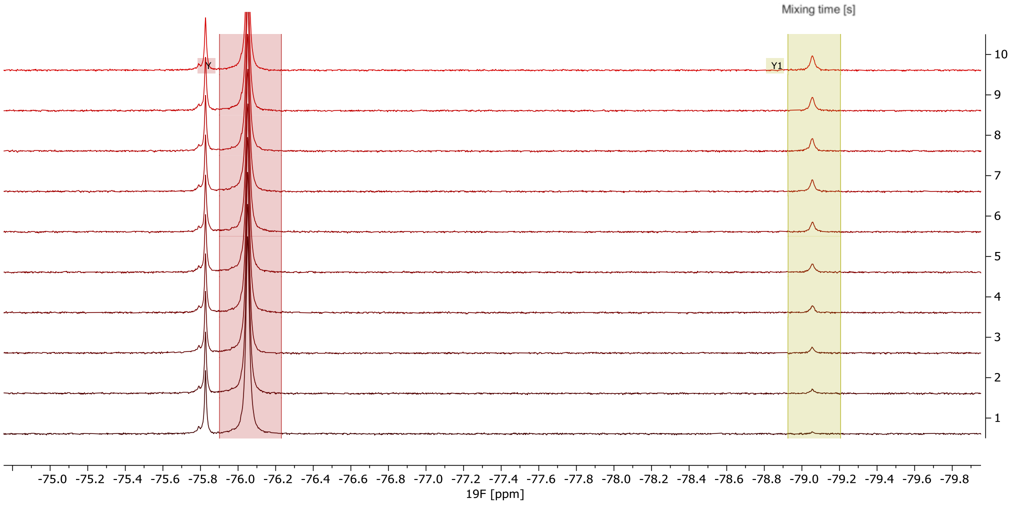

Figure S98: Raw ^{19}F EXSY data for the *α*-triflate dissociation in the corresponding *α*-triflate (left corner) measured at -50 °C at a concentration $^-$OTf of 0.046 M.

1D Selective ^{19}F EXSY data at [$^{-}$OTf] = 0.090 M

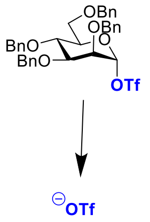

Spectrum number	Mixing Time [s]	*α*-triflate (-76.06 ppm) resonance intensity	$^{-}$OTf (-79.06 ppm) resonance intensity
1	0.005	9378.48	84.6618
2	0.01	8801.98	202.731
3	0.015	8845.2	224.336
4	0.02	8422.25	357.249
5	0.025	8268.76	447.287
6	0.03	7894.29	476.019
7	0.035	7822.71	590.264
8	0.04	7433.38	645.746
9	0.045	7388.29	741.646
10	0.05	7075.81	821.489

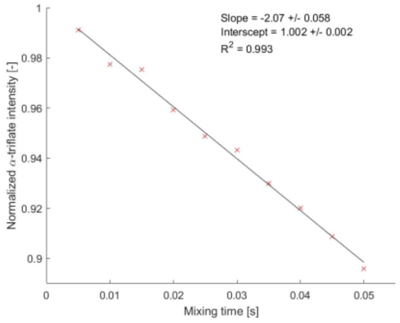

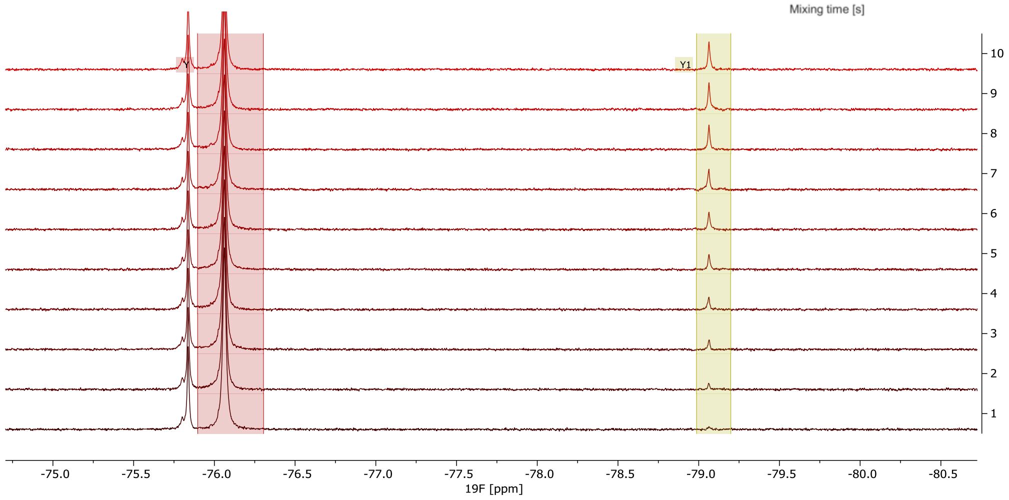

Figure S99: Raw ^{19}F EXSY data for the *α*-triflate dissociation in the corresponding *α*-triflate (left corner) measured at -50 °C at a concentration $^{-}$OTf of 0.090 M.

1D Selective ^{19}F EXSY data at [$^{-}$OTf] = 0.16 M

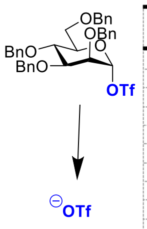

Spectrum number	Mixing Time [s]	*α*-triflate (-76.07 ppm) resonance intensity	$^{-}$OTf (-79.06 ppm) resonance intensity
1	0.003	5025.66	31.8023
2	0.006	4906.06	129.043
3	0.009	4793.72	93.2034
4	0.012	4687	185.931
5	0.015	4686.2	250.494
6	0.018	4628.95	232.458
7	0.021	4395.28	334.529
8	0.024	4258.35	331.3
9	0.027	4114.16	414.55
10	0.03	4055.89	495.485

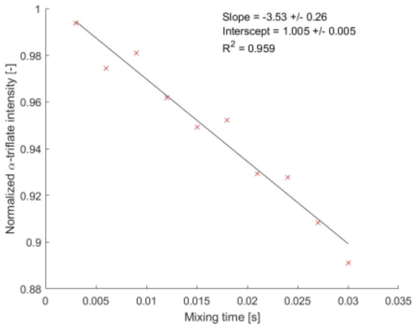

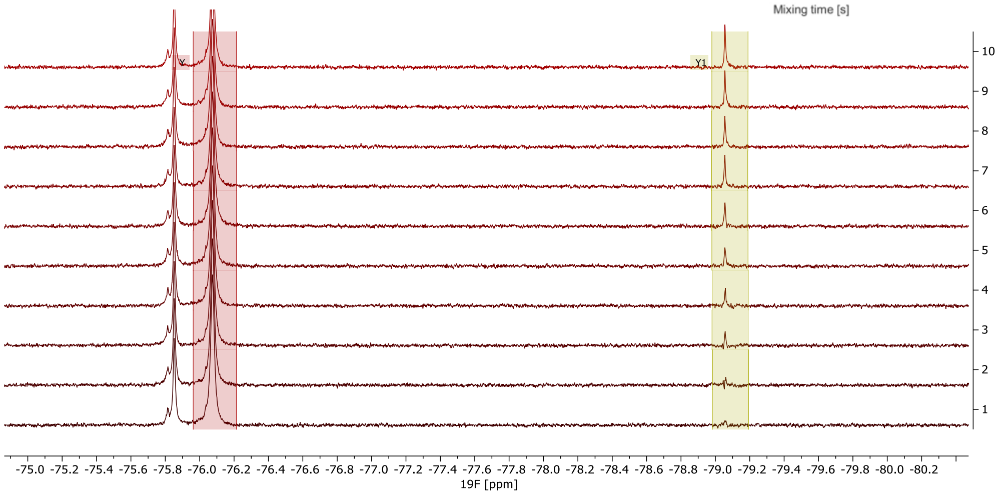

Figure S100: Raw ^{19}F EXSY data for the *α*-triflate dissociation in the corresponding *α*-triflate (left corner) measured at -50 °C at a concentration $^{-}$OTf of 0.16 M.

1D Selective ^{19}F EXSY data at [$^{-}$OTf] = 0.015 M

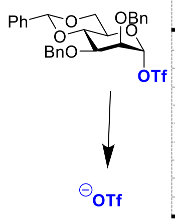

Spectrum number	Mixing Time [s]	α-triflate (-75.92 ppm) resonance intensity	$^{-}$OTf (-78.92 ppm) resonance intensity
1	0.1	45120.7	510.918
2	0.2	38681.7	814.092
3	0.3	33206.4	990.271
4	0.4	28483.1	1123.58
5	0.5	24511.6	1252.35
6	0.6	21072.2	1348.57
7	0.7	18208.1	1394.48
8	0.8	15707.6	1376.88
9	0.9	13565.2	1407.6
10	1	11724.4	1345.47

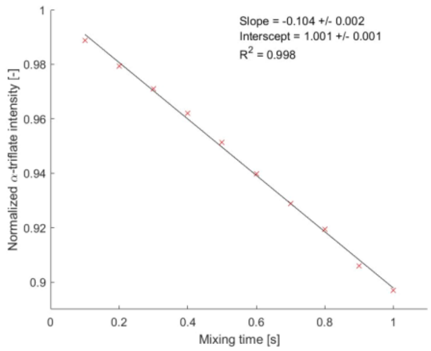

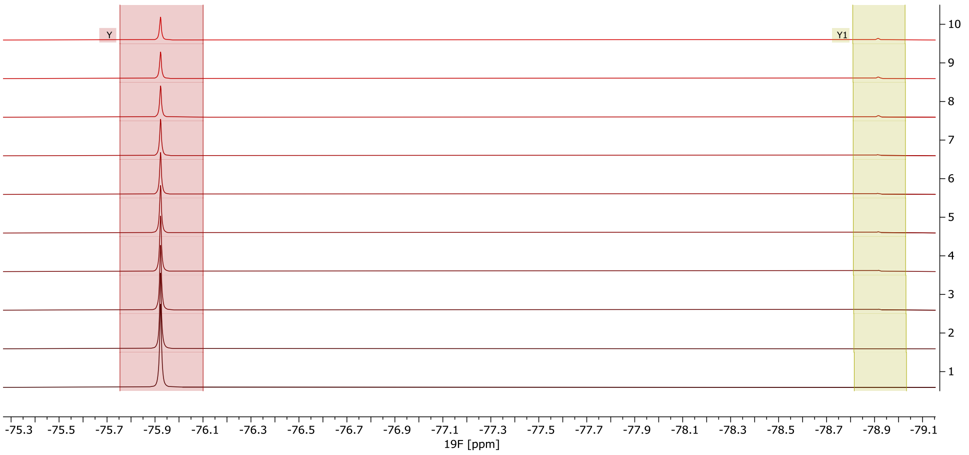

Figure S101: Raw ^{19}F EXSY data for the *α*-triflate dissociation in the corresponding *α*-triflate (left corner) measured at -30 °C at a concentration $^{-}$OTf of 0.015 M.

1D Selective ^{19}F EXSY data at [$^{-}$OTf] = 0.049 M

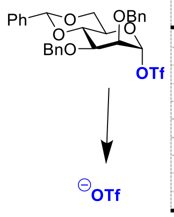

Spectrum number	Mixing Time [s]	α-triflate (-75.93 ppm) resonance intensity	$^{-}$OTf (-78.98 ppm) resonance intensity
1	0.027	35570.9	395.919
2	0.054	33928.3	741.17
3	0.081	32014.2	1136.77
4	0.108	30192.3	1344.44
5	0.135	28671.8	1654.62
6	0.162	26979.1	1893.11
7	0.189	25435.9	2152.87
8	0.216	24123.4	2323.95
9	0.243	22727.9	2514.78
10	0.27	21518.1	2670.32

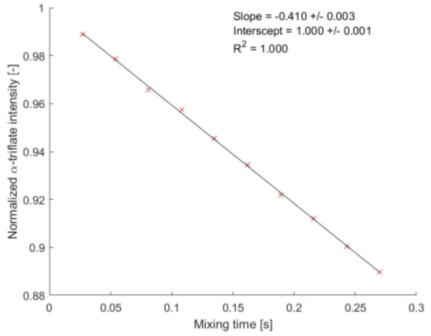

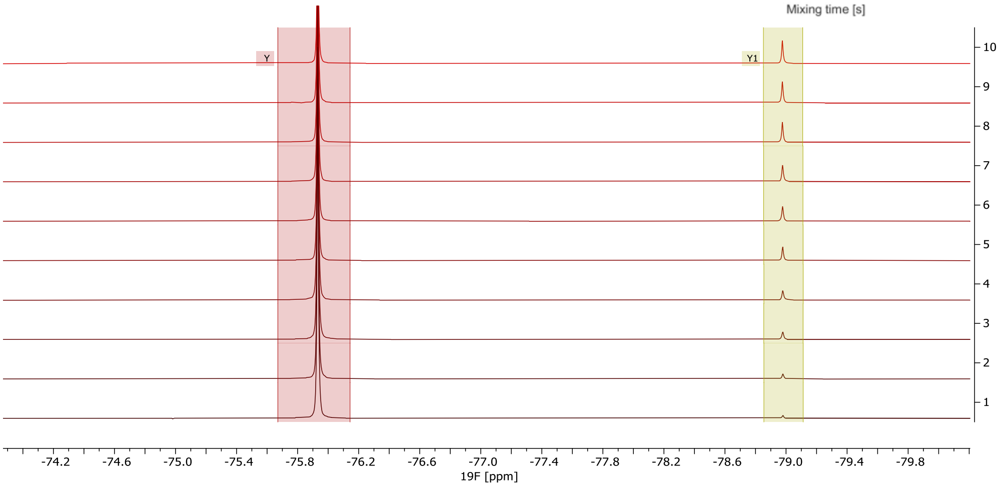

Figure S102: Raw ^{19}F EXSY data for the *α*-triflate dissociation in the corresponding *α*-triflate (left corner) measured at -30 °C at a concentration $^{-}$OTf of 0.049 M.

1D Selective ^{19}F EXSY data at [$^{-}$OTf] = 0.098 M

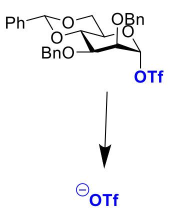

Spectrum number	Mixing Time [s]	α-triflate (-75.94 ppm) resonance intensity	$^{-}$OTf (-78.99 ppm) resonance intensity
1	0.014	25050.7	337.966
2	0.028	24394.5	534.761
3	0.042	22854.9	826.234
4	0.056	21770.7	1061.09
5	0.07	21205.6	1240.48
6	0.084	20280.7	1450.6
7	0.098	19339.2	1676.75
8	0.112	18686	1823.57
9	0.126	17875.1	2071.48
10	0.14	17139.2	2135.18

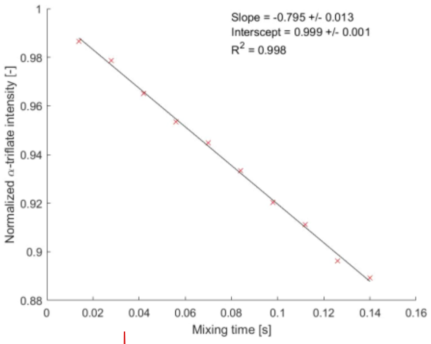

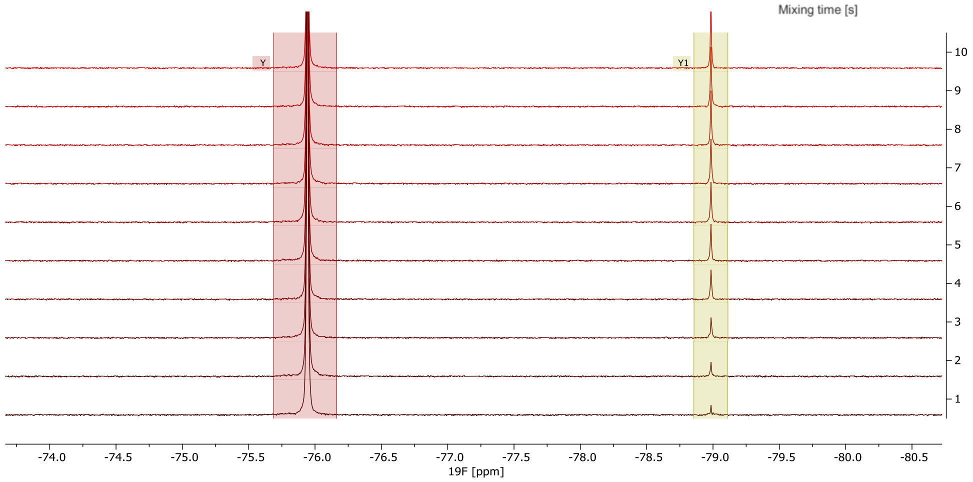

Figure S103: Raw ^{19}F EXSY data for the *α*-triflate dissociation in the corresponding *α*-triflate (left corner) measured at -30 °C at a concentration $^{-}$OTf of 0.098 M.

1D Selective ^{19}F EXSY data at [$^{-}$OTf] = 0.16 M

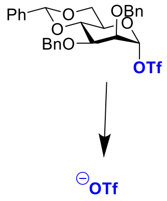

Spectrum number	Mixing Time [s]	α-triflate (-75.96 ppm) resonance intensity	$^{-}$OTf (-78.98 ppm) resonance intensity
1	0.01	16021.4	237.62
2	0.02	15599.3	390.379
3	0.03	14488.8	611.222
4	0.04	13928.5	713.904
5	0.05	13673	947.239
6	0.06	12999.3	1068.25
7	0.07	12438.7	1190.17
8	0.08	12035.6	1272.85
9	0.09	11523.4	1486.42
10	0.1	11084.3	1638.81

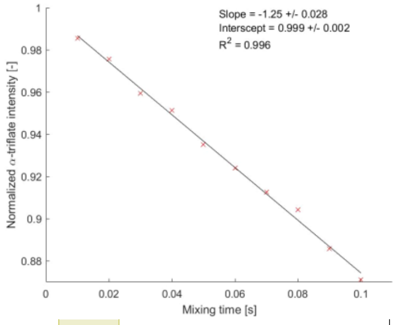

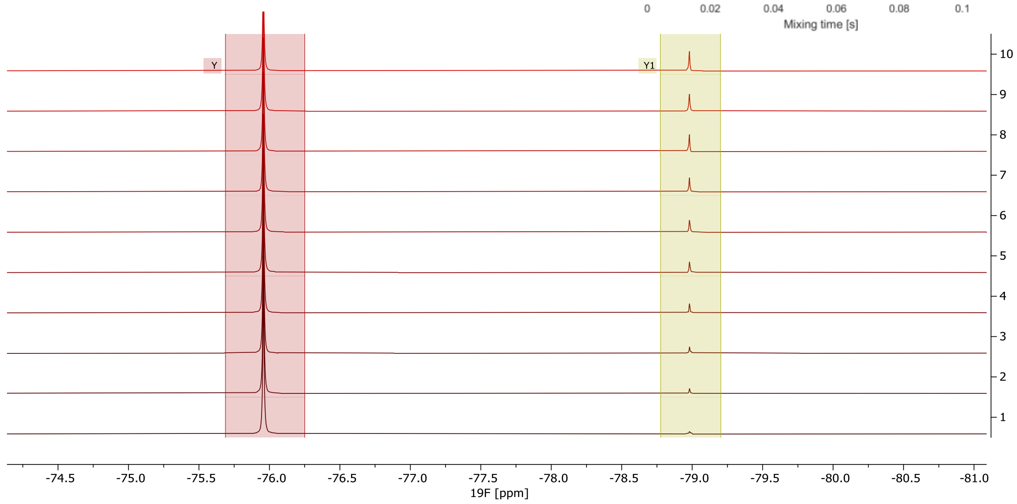

Figure S104: Raw ^{19}F EXSY data for the *α*-triflate dissociation in the corresponding *α*-triflate (left corner) measured at -30 °C at a concentration $^{-}$OTf of 0.16 M.

1D Selective ^{19}F EXSY data at [$^{-}$OTf] = 0.014 M

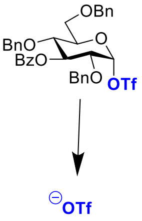

Spectrum number	Mixing Time [s]	α-triflate (-75.97 ppm) resonance intensity	$^{-}$OTf (-78.98 ppm) resonance intensity
1	0.06	31210.5	224.156
2	0.12	26647.9	409.293
3	0.18	23018.5	560.924
4	0.24	19815.9	645.579
5	0.3	17254.9	734.143
6	0.36	14918.4	829.166
7	0.42	12961.6	881.117
8	0.48	11285.6	879.853
9	0.54	9821.35	905.594
10	0.6	8646.78	931.242

Temperature = -50 °C

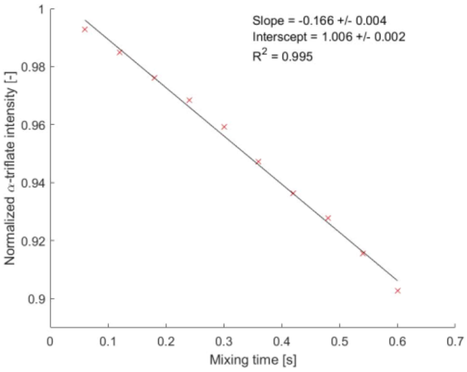

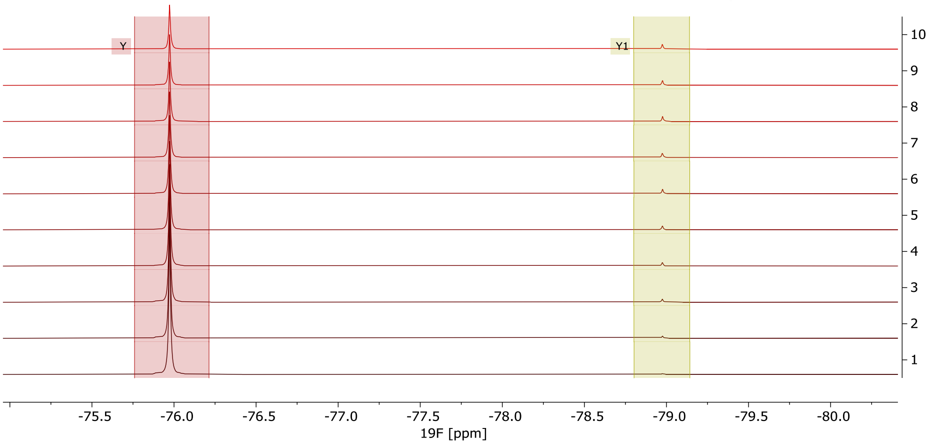

Figure S105: Raw ^{19}F EXSY data for the *α*-triflate dissociation in the corresponding *α*-triflate (left corner) measured at -50 °C at a concentration $^{-}$OTf of 0.014 M.

1D Selective ^{19}F EXSY data at [$^{-}$OTf] = 0.047 M

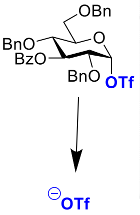

Spectrum number	Mixing Time [s]	α-triflate (-75.97 ppm) resonance intensity	$^{-}$OTf (-79.06ppm) resonance intensity
1	0.02	30609.2	281.468
2	0.04	28235.8	569.579
3	0.06	26876.5	839.458
4	0.08	25259.7	1042.12
5	0.1	23920.9	1276.9
6	0.12	22581.1	1542.65
7	0.14	21370.2	1681.91
8	0.16	20202.5	1920.53
9	0.18	19045.9	1977.19
10	0.2	18042.6	2252.25

Temperature = -50 °C

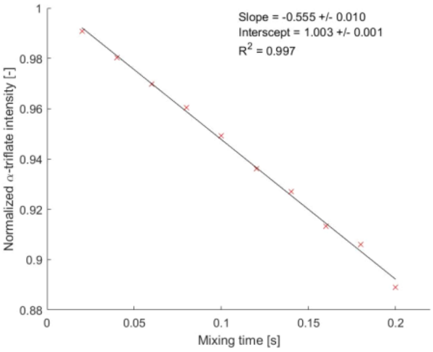

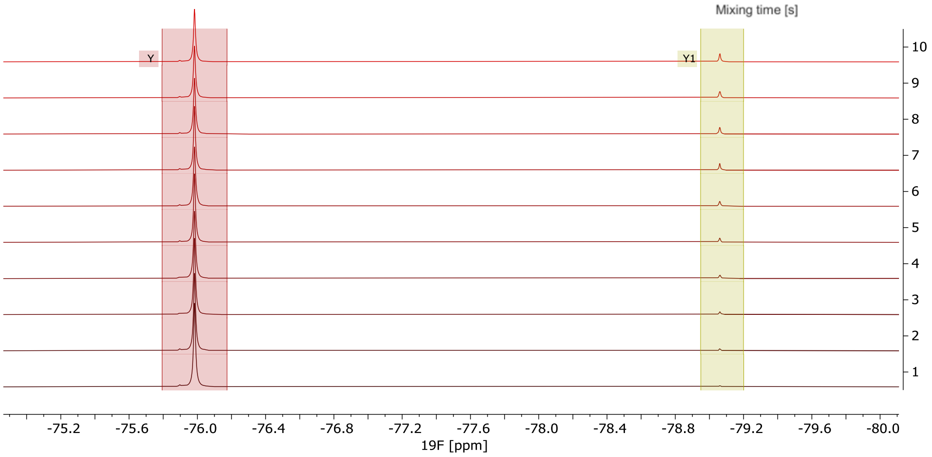

Figure S106: Raw ^{19}F EXSY data for the *α*-triflate dissociation in the corresponding *α*-triflate (left corner) measured at -50 °C at a concentration $^{-}$OTf of 0.047 M.

1D Selective ^{19}F EXSY data at [$^{-}$OTf] = 0.091 M

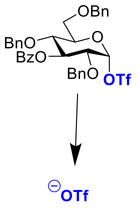

Spectrum number	Mixing Time [s]	*α*-triflate (-76.00 ppm) resonance intensity	$^{-}$OTf (-79.06 ppm) resonance intensity
1	0.012	27400.6	317.492
2	0.024	25783.9	569.295
3	0.036	24705.3	895.021
4	0.048	24153	1073.59
5	0.06	22858.8	1335.74
6	0.072	22085.3	1536.61
7	0.084	21187.6	1850.38
8	0.096	20386.7	2027.02
9	0.108	19628.7	2161.47
10	0.12	18843.2	2391.32

Temperature = -50 °C

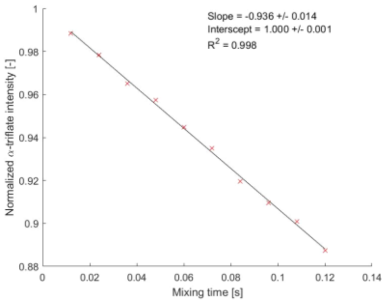

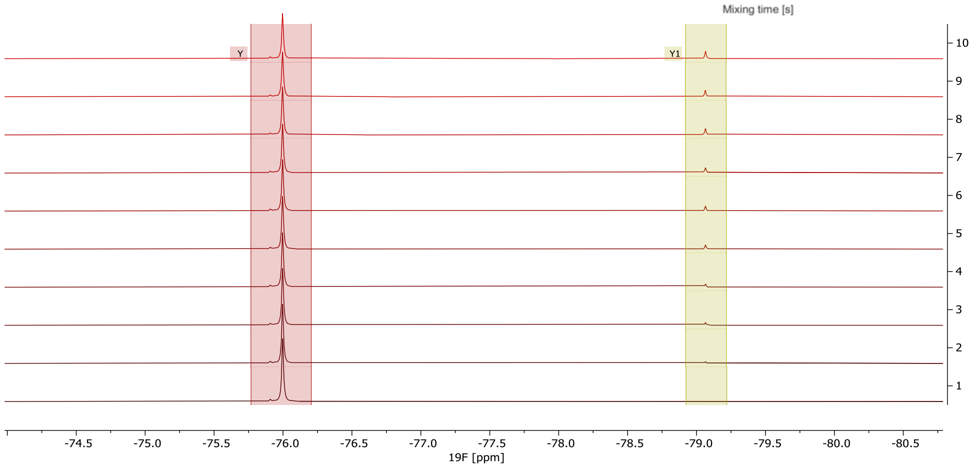

Figure S107: Raw ^{19}F EXSY data for the *α*-triflate dissociation in the corresponding *α*-triflate (left corner) measured at -50 °C at a concentration $^{-}$OTf of 0.091 M.

1D Selective ^{19}F EXSY data at [$^{-}$OTf] = 0.16 M

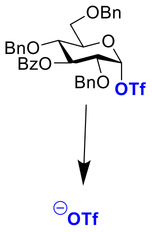

Spectrum number	Mixing Time [s]	*α*-triflate (-76.01ppm) resonance intensity	$^{-}$OTf (-79.06 ppm) resonance intensity
1	0.008	20422.1	256.002
2	0.016	19903.1	542.393
3	0.024	18834.2	763.431
4	0.032	18274.8	1023.88
5	0.04	17850.7	1216.35
6	0.048	17117.9	1445.23
7	0.056	16486	1670.58
8	0.064	16006.4	1907.22
9	0.072	15521	2062.56
10	0.08	14909.3	2275.58

Temperature = -50 °C

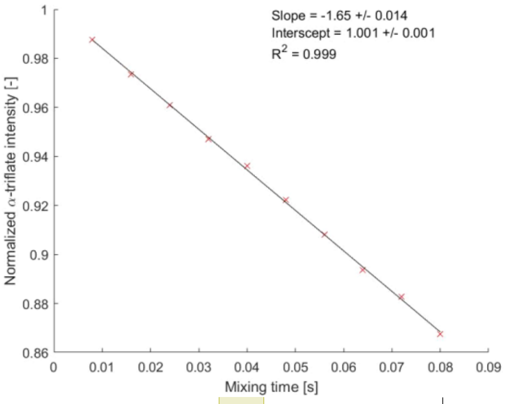

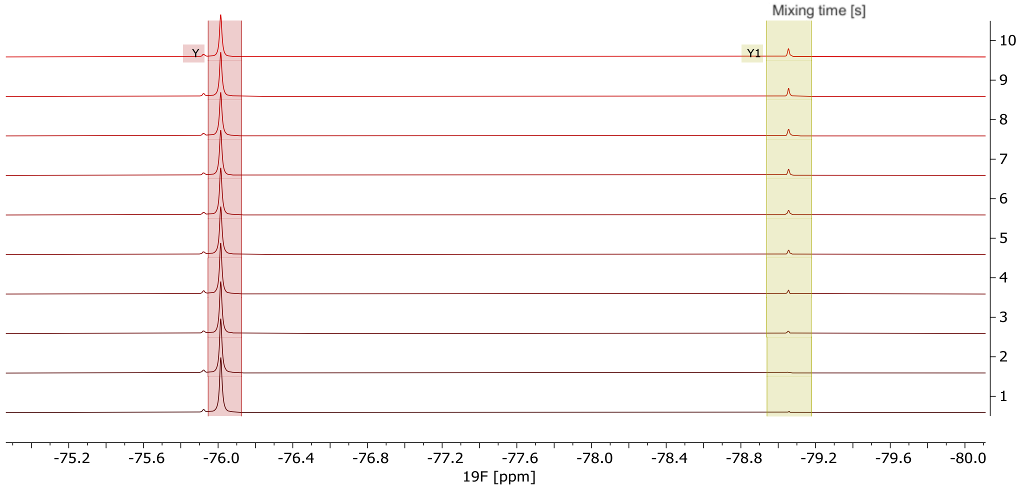

Figure S108: Raw ^{19}F EXSY data for the *α*-triflate dissociation in the corresponding *α*-triflate (left corner) measured at -50 °C at a concentration $^{-}$OTf of 0.16 M.

1D Selective ^{19}F EXSY data at [$^{-}$OTf] = 0.013 M

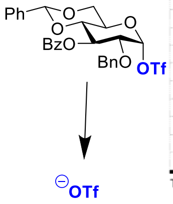

Spectrum number	Mixing Time [s]	α-triflate (-75.82 ppm) resonance intensity	$^{-}$OTf (-78.94ppm) resonance intensity
1	0.08	30880.6	300.918
2	0.16	26980.9	524.794
3	0.24	23589.1	719.876
4	0.32	20859.2	878.276
5	0.4	18199.2	1024.44
6	0.48	16116.7	1070.34
7	0.56	14195.9	1161.26
8	0.64	12551.7	1193.17
9	0.72	11090.8	1246.05
10	0.8	9734.47	1257.76

Temperature = -30 °C

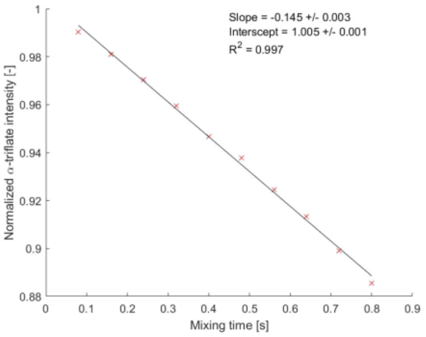

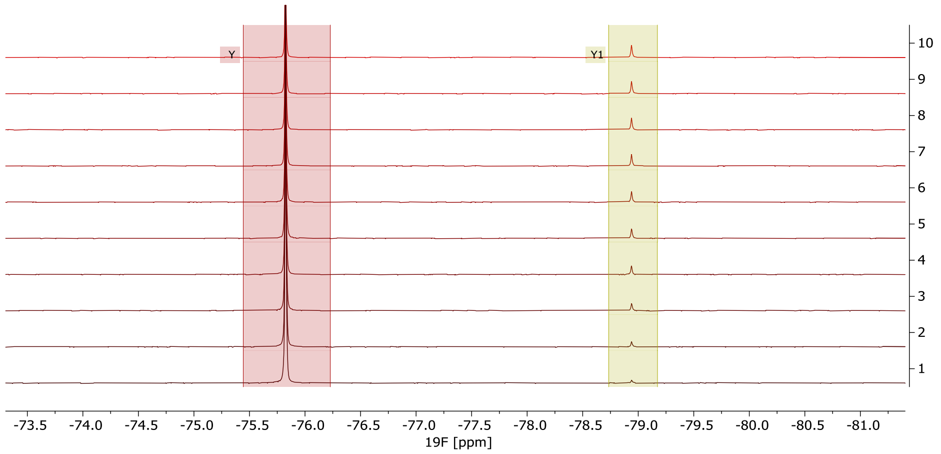

Figure S109: Raw ^{19}F EXSY data for the *α*-triflate dissociation in the corresponding *α*-triflate (left corner) measured at -30 °C at a concentration $^{-}$OTf of 0.013 M.

1D Selective ^{19}F EXSY data at [$^{-}$OTf] = 0.049 M

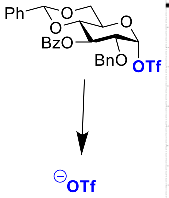

Spectrum number	Mixing Time [s]	α-triflate (-75.84 ppm) resonance intensity	$^{-}$OTf (-79.01 ppm) resonance intensity
1	0.02	25917.6	288.331
2	0.04	25103.4	610.282
3	0.06	23529.7	838.19
4	0.08	22432	1093.98
5	0.1	21375	1275.67
6	0.12	20617.2	1496.17
7	0.14	19667.3	1738.71
8	0.16	18879.7	1887.91
9	0.18	18046	2092.29
10	0.2	17271.9	2239.54

Temperature = -30 °C

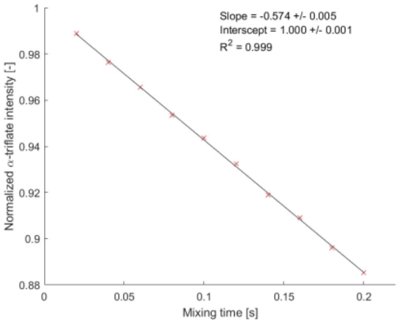

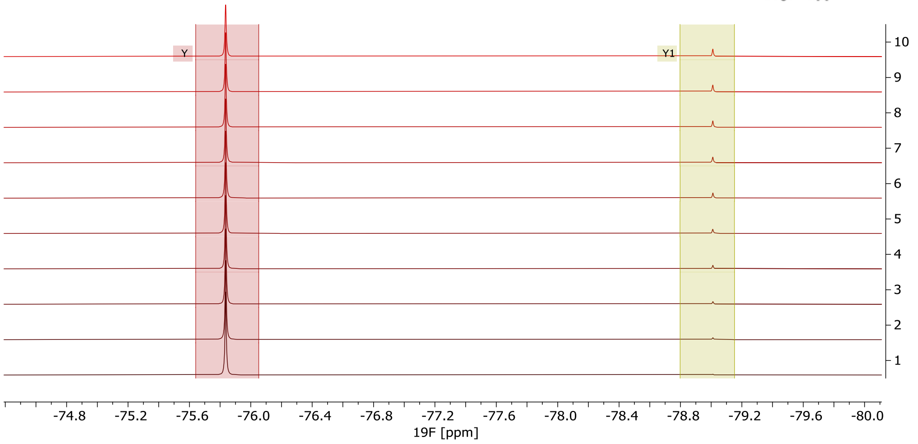

Figure S110: Raw ^{19}F EXSY data for the *α*-triflate dissociation in the corresponding *α*-triflate (left corner) measured at -30 °C at a concentration $^{-}$OTf of 0.049 M.

1D Selective ^{19}F EXSY data at [$^{-}$OTf] = 0.093 M

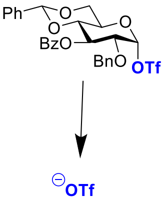

Spectrum number	Mixing Time [s]	α-triflate (-78.85 ppm) resonance intensity	$^{-}$OTf (-79.01 ppm) resonance intensity
1	0.012	20229.8	237.763
2	0.024	19695.5	510.891
3	0.036	19292.8	813.817
4	0.048	18828.2	964.226
5	0.06	18158.8	1232.08
6	0.072	17363.4	1455.1
7	0.084	16621.1	1682.51
8	0.096	15978.9	1868.12
9	0.108	15419.7	1998.13
10	0.12	14982.7	2205.59

Temperature = -30 °C

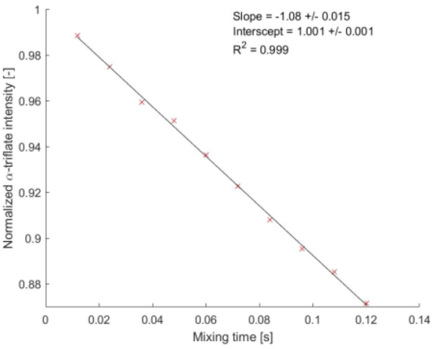

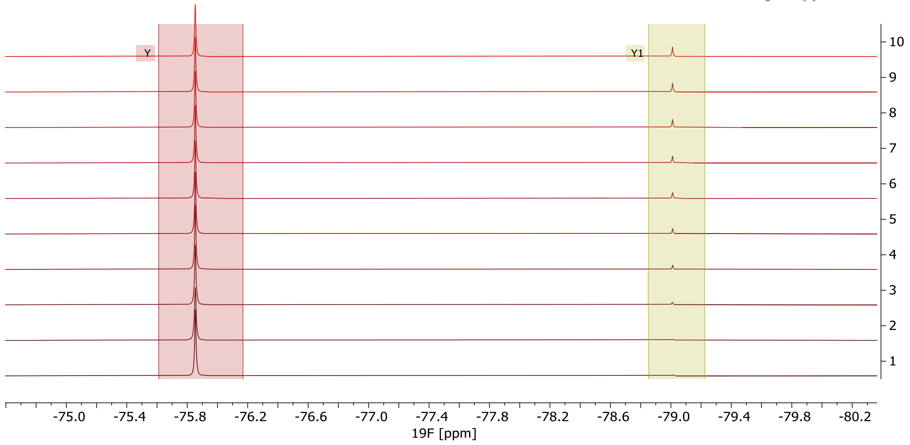

Figure S111: Raw ^{19}F EXSY data for the *α*-triflate dissociation in the corresponding *α*-triflate (left corner) measured at -30 °C at a concentration $^{-}$OTf of 0.093 M.

1D Selective ^{19}F EXSY data at [$^{-}$OTf] = 0.16 M

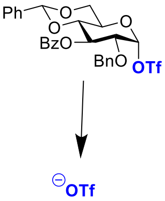

Spectrum number	Mixing Time [s]	α-triflate (-75.88 ppm) resonance intensity	$^{-}$OTf (-79.00 ppm) resonance intensity
1	0.008	15945.4	256.601
2	0.016	15374.7	439.444
3	0.024	14982.3	744.707
4	0.032	14563.4	677.638
5	0.04	14147.9	1005.76
6	0.048	13757	1172.03
7	0.056	13405.4	1439.21
8	0.064	12993.9	1619.24
9	0.072	12658.6	1738.29
10	0.08	12292.2	1941.03

Temperature = -30 °C

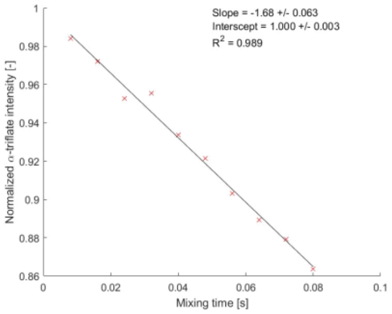

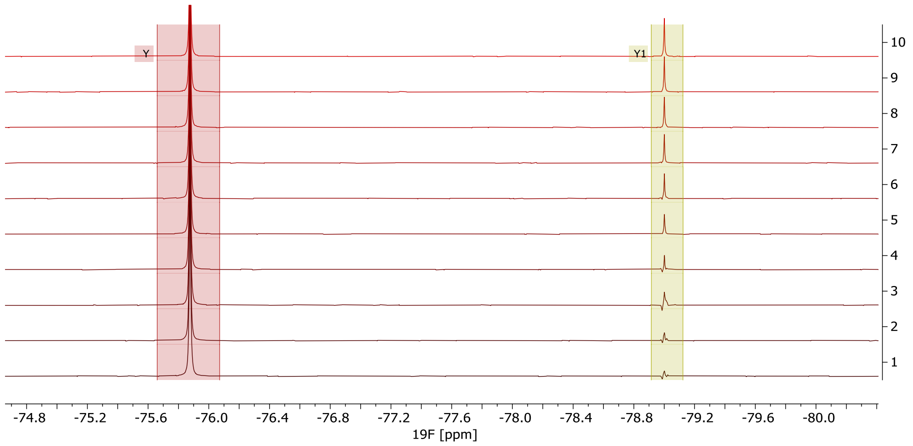

Figure S112: Raw ^{19}F EXSY data for the *α*-triflate dissociation in the corresponding *α*-triflate (left corner) measured at -30 °C at a concentration $^{-}$OTf of 0.16 M.

1D Selective ^{19}F EXSY data at [$^{-}$OTf] = 0.024 M

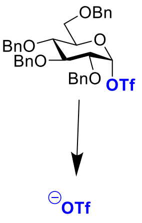

Spectrum number	Mixing Time [s]	α-triflate (-76.13 ppm) resonance intensity	$^{-}$OTf (-78.97 ppm) resonance intensity
1	0.018	24205.8	269.511
2	0.036	23443.8	519.042
3	0.054	22146.6	759.734
4	0.072	21019	959.537
5	0.09	20062.2	1163.42
6	0.108	19171.1	1357.16
7	0.126	18288.5	1534.98
8	0.144	17411.1	1687.16
9	0.162	16623.8	1859.34
10	0.18	15995.8	1964.23

Temperature = -50 °C

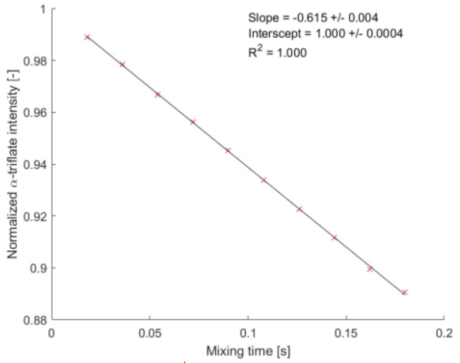

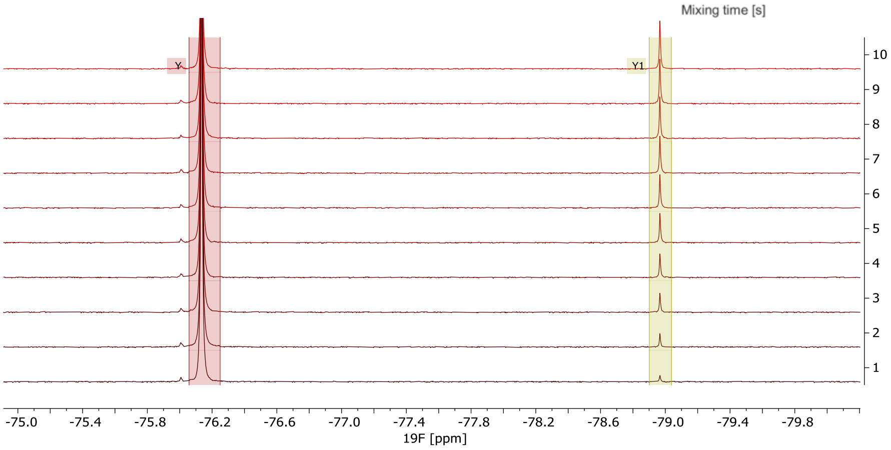

Figure S113: Raw ^{19}F EXSY data for the *α*-triflate dissociation in the corresponding *α*-triflate (left corner) measured at -50 °C at a concentration $^{-}$OTf of 0.024 M.

1D Selective ^{19}F EXSY data at [$^{-}$OTf] = 0.058 M

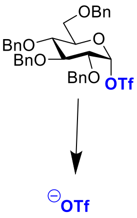

Spectrum number	Mixing Time [s]	α-triflate (-76.15 ppm) resonance intensity	$^{-}$OTf (-79.04 ppm) resonance intensity
1	0.0055	21930.9	214.558
2	0.011	21986.2	470.658
3	0.0165	21420	662.41
4	0.022	20579.8	887.326
5	0.0275	20148	1151.96
6	0.033	20033.4	1276.48
7	0.0385	19783	1582.14
8	0.044	19127.2	1768.33
9	0.0495	18661	1999.27
10	0.055	18457	2198.36

Temperature = -50 °C

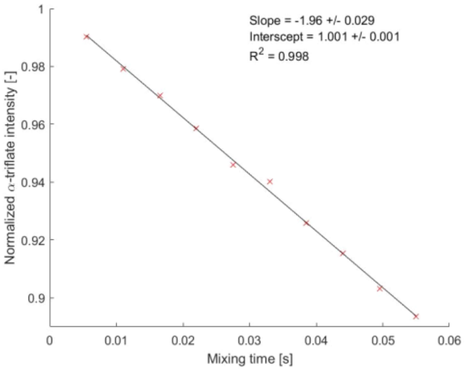

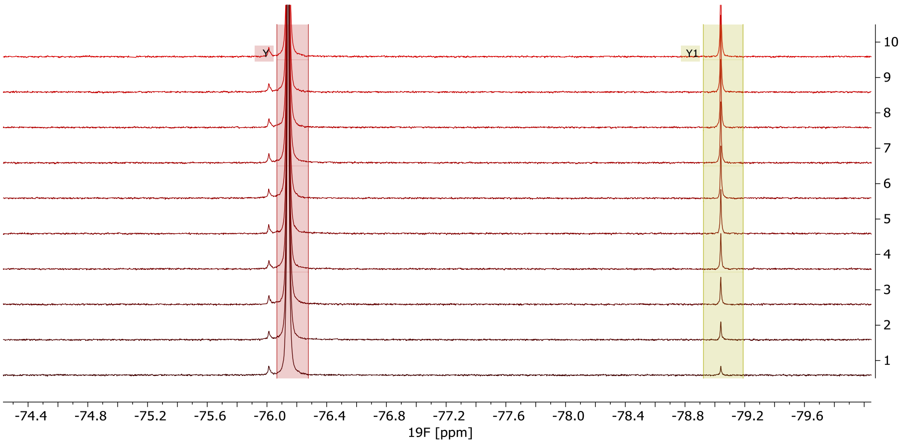

Figure S114: Raw ^{19}F EXSY data for the *α*-triflate dissociation in the corresponding *α*-triflate (left corner) measured at -50 °C at a concentration $^{-}$OTf of 0.058 M.

1D Selective ^{19}F EXSY data at [$^{-}$OTf] = 0.10 M

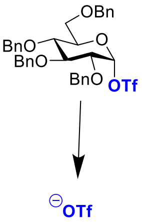

Spectrum number	Mixing Time [s]	α-triflate (-76.15 ppm) resonance intensity	$^{-}$OTf (-79.05 ppm) resonance intensity
1	0.003	17650.3	199.863
2	0.006	17431.8	394.49
3	0.009	17173.4	638.774
4	0.012	16916.6	863.448
5	0.015	16729.5	983.096
6	0.018	16497.5	1229.4
7	0.021	16212.2	1338.9
8	0.024	16038.1	1564.7
9	0.027	15814.6	1715.48
10	0.03	15654.4	1951.17

Temperature = -50 °C

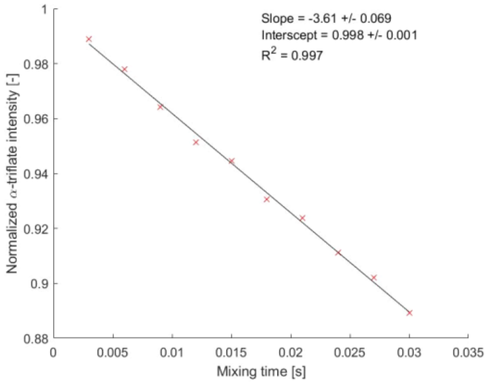

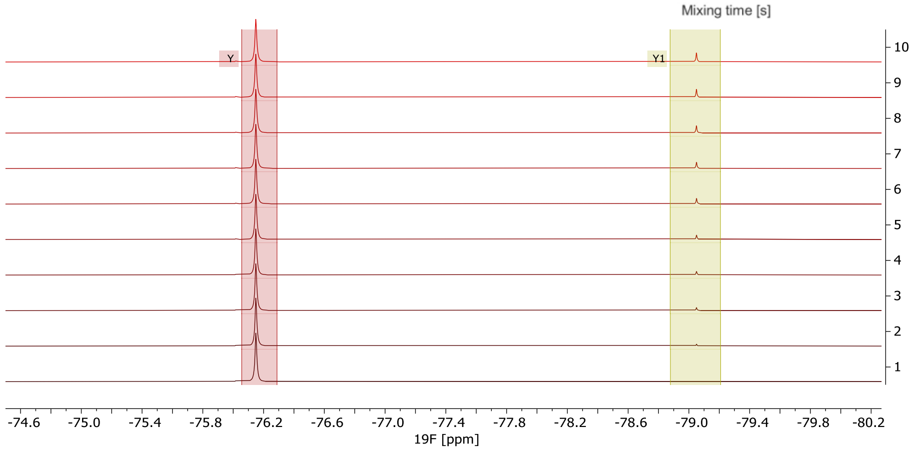

Figure S115: Raw ^{19}F EXSY data for the *α*-triflate dissociation in the corresponding *α*-triflate (left corner) measured at -50 °C at a concentration $^{-}$OTf of 0.010 M.

1D Selective ^{19}F EXSY data at [$^{-}$OTf] = 0.17 M

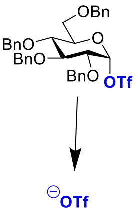

Spectrum number	Mixing Time [s]	α-triflate (-76.15 ppm) resonance intensity	$^{-}$OTf (-79.05 ppm) resonance intensity
1	0.003	17650.3	199.863
2	0.006	17431.8	394.49
3	0.009	17173.4	638.774
4	0.012	16916.6	863.448
5	0.015	16729.5	983.096
6	0.018	16497.5	1229.4
7	0.021	16212.2	1338.9
8	0.024	16038.1	1564.7
9	0.027	15814.6	1715.48
10	0.03	15654.4	1951.17

Temperature = -50 °C

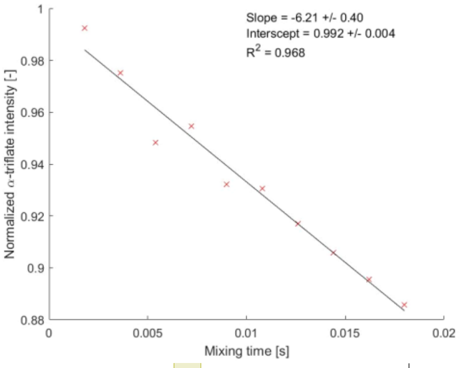

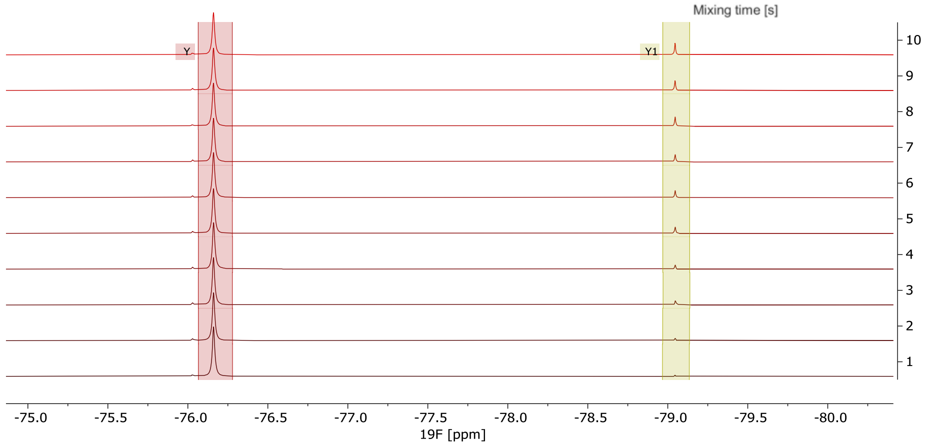

Figure S116: Raw ^{19}F EXSY data for the *α*-triflate dissociation in the corresponding *α*-triflate (left corner) measured at -50 °C at a concentration $^{-}$OTf of 0.17 M.

1D Selective ^{19}F EXSY data at [$^{-}$OTf] = 0.012 M

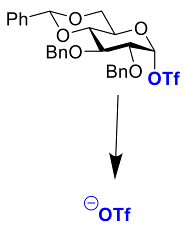

Spectrum number	Mixing Time [s]	α-triflate (-75.99 ppm) resonance intensity	$^{-}$OTf (-78.93 ppm) resonance intensity
1	0.06	50773.6	646.792
2	0.12	46066.4	1095.48
3	0.18	41729.5	1439.4
4	0.24	37977.4	1765.8
5	0.3	34598.3	1980.97
6	0.36	31442.8	2206.25
7	0.42	28745.8	2337.5
8	0.48	26220.2	2439.15
9	0.54	23929.1	2542.83
10	0.6	21819.9	2576.35

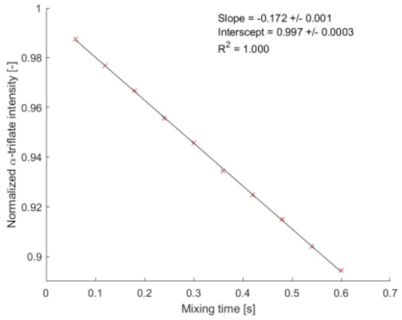

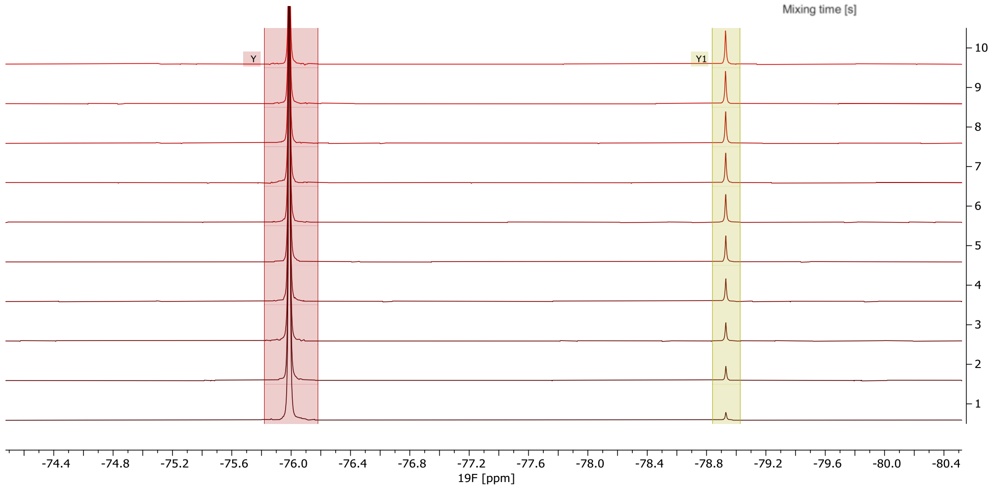

Figure S117: Raw ^{19}F EXSY data for the *α*-triflate dissociation in the corresponding *α*-triflate (left corner) measured at -30 °C at a concentration $^{-}$OTf of 0.012 M.

1D Selective ^{19}F EXSY data at [$^{-}$OTf] = 0.047 M

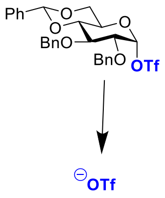

Spectrum number	Mixing Time [s]	α-triflate (-75.99 ppm) resonance intensity	$^{-}$OTf (-79.00 ppm) resonance intensity
1	0.01	43990.9	573.352
2	0.02	43839.1	1080.91
3	0.03	42378.7	1553.37
4	0.04	40409.2	2002.94
5	0.05	38889.5	2446.56
6	0.06	38138.7	2829.7
7	0.07	37321.2	3267.92
8	0.08	36133.7	3611.79
9	0.09	34835	3964.44
10	0.1	33850	4282.25

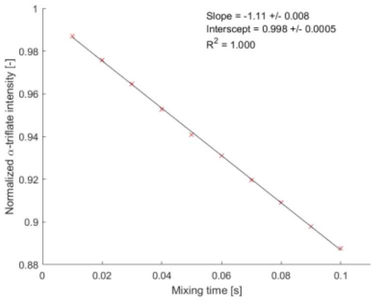

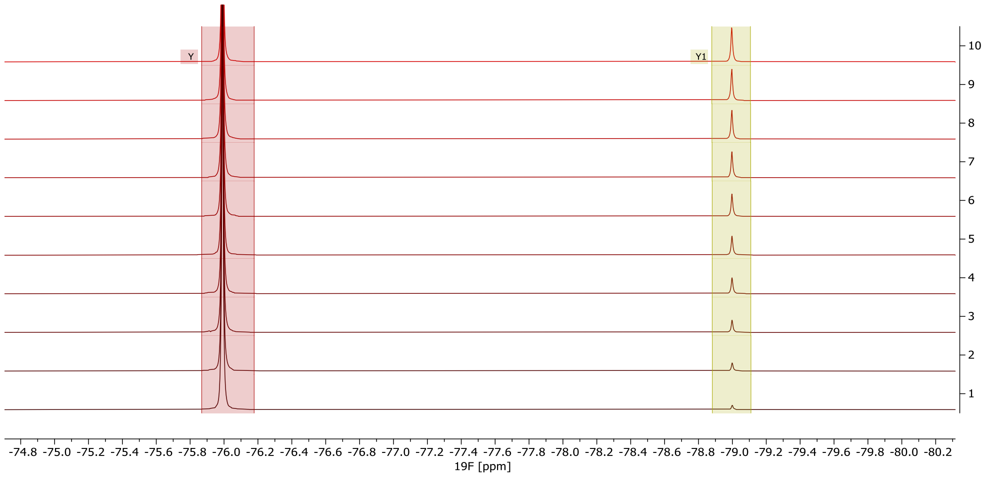

Figure S118: Raw ^{19}F EXSY data for the *α*-triflate dissociation in the corresponding *α*-triflate (left corner) measured at -30 °C at a concentration $^{-}$OTf of 0.047 M.

1D Selective ^{19}F EXSY data at [$^{-}$OTf] = 0.10 M

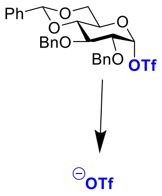

Spectrum number	Mixing Time [s]	α-triflate (-76.00 ppm) resonance intensity	$^{-}$OTf (-79.00 ppm) resonance intensity
1	0.0045	33870.2	435.475
2	0.009	33552.5	837.515
3	0.0135	31865.3	1148.66
4	0.018	31016.9	1489.68
5	0.0225	31126.8	1908.52
6	0.027	30523.4	2241.21
7	0.0315	28984.8	2516.35
8	0.036	28600.3	2873.46
9	0.0405	28532.2	3069.82
10	0.045	27594.6	3411.81

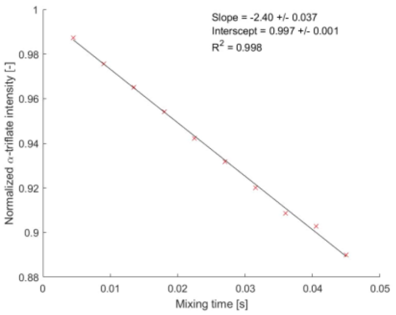

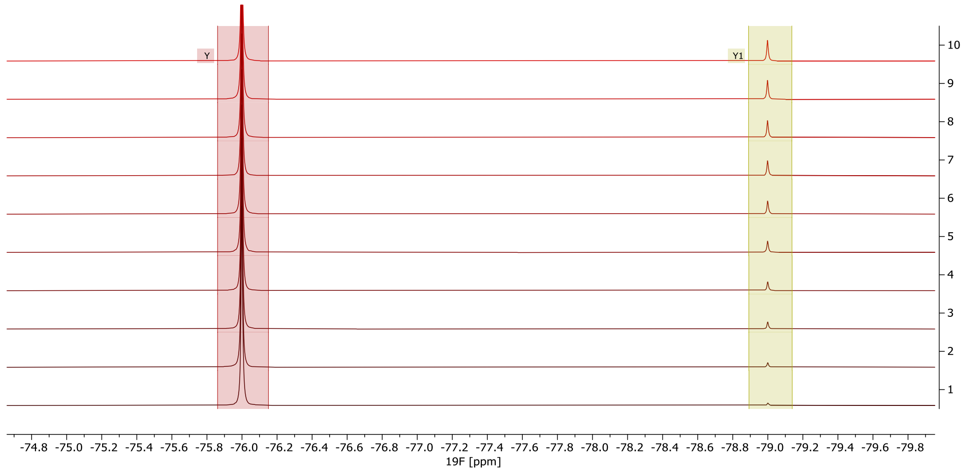

Figure S119: Raw ^{19}F EXSY data for the *α*-triflate dissociation in the corresponding *α*-triflate (left corner) measured at -30 °C at a concentration $^{-}$OTf of 0.10 M.

1D Selective ^{19}F EXSY data at [$^{-}$OTf] = 0.19 M

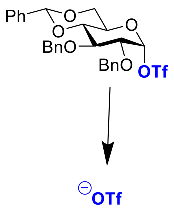

Spectrum number	Mixing Time [s]	α-triflate (-76.01 ppm) resonance intensity	$^{-}$OTf (-78.99 ppm) resonance intensity
1	0.003	24105.4	304.185
2	0.006	24010	720.225
3	0.009	23791.3	994.323
4	0.012	23319.2	1300.11
5	0.015	22450.9	1524.78
6	0.018	21639	1965.15
7	0.021	20927.7	2051.99
8	0.024	20513.9	2401.96
9	0.027	20401	2634.44
10	0.03	20228.8	2927.38

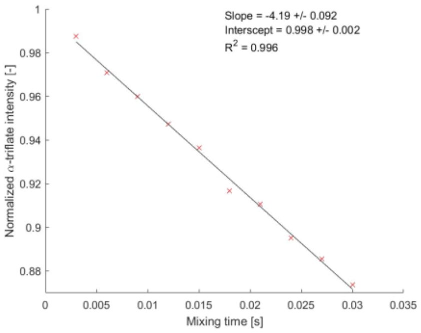

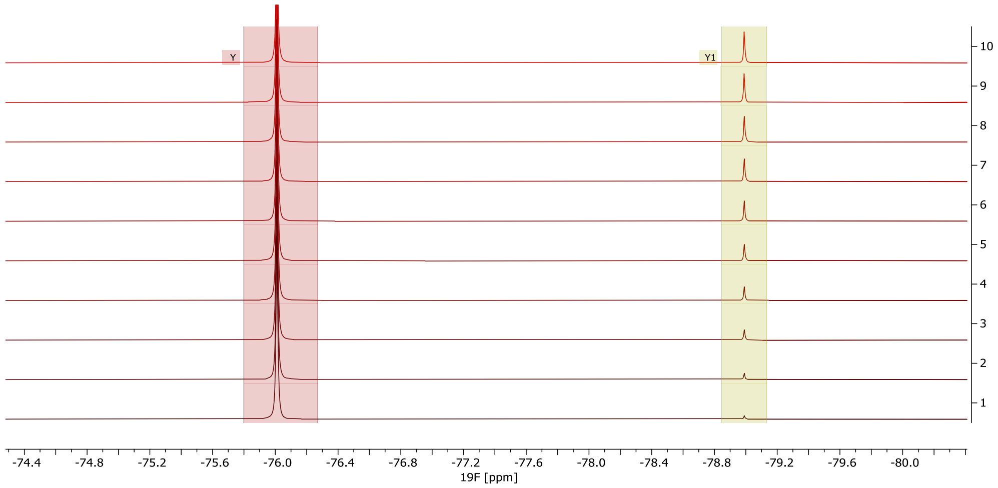

Figure S120: Raw ^{19}F EXSY data for the *α*-triflate dissociation in the corresponding *α*-triflate (left corner) measured at -30 °C at a concentration $^{-}$OTf of 0.19 M.

Selective 1D EXSY at -80 °C at 32.2 mM [⁻OTf]

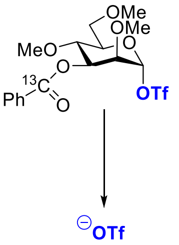

Spectrum number	Mixing Time [s]	Alpha (-76.0 ppm) resonance intensity	⁻OTf (-79.1 ppm) resonance intensity
1	0.011	18588.2	220.796
2	0.022	17988	409.128
3	0.033	17401.1	482.153
4	0.044	16627.4	643.316
5	0.055	15821.8	795.56
6	0.066	15234.9	915.779
7	0.077	14587.5	1109.58
8	0.088	14039.6	1200.97
9	0.099	13382.4	1230.99
10	0.11	12887.2	1391.4

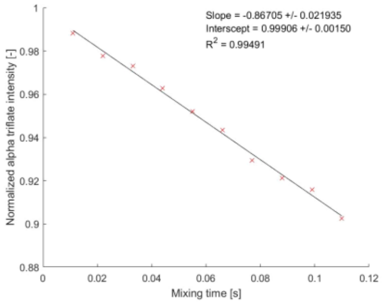

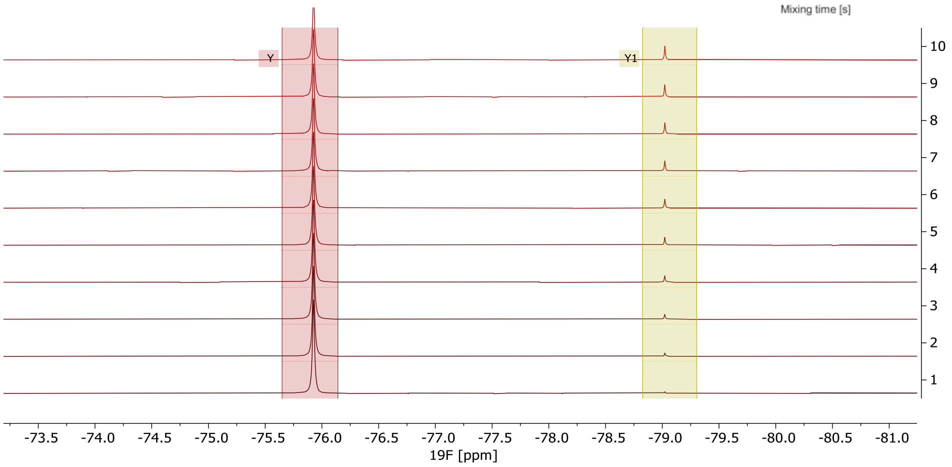

Figure S121: Raw ^{19}F EXSY data for the *α*-triflate dissociation in the corresponding *α*-triflate (left corner) measured at -80 °C at a concentration ⁻OTf of 0.032 M.

Selective 1D EXSY at -80 °C at 98.0 mM [⁻OTf]

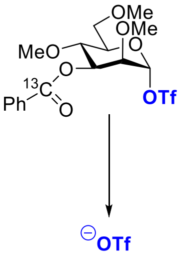

Spectrum number	Mixing Time [s]	Alpha (-76.0 ppm) resonance intensity	⁻OTf (-79.1 ppm) resonance intensity
1	0.01	13888.9	194.886
2	0.02	13174.7	240.236
3	0.03	12507.1	431.563
4	0.04	12158	543.74
5	0.05	11685.5	638.357
6	0.06	11200.8	804.668
7	0.07	10721.5	879.508
8	0.08	10319	967.965
9	0.09	9908.84	1026.96
10	0.1	9521.76	1139.06

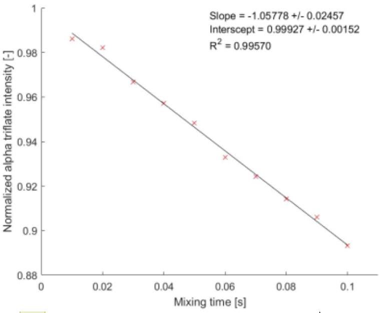

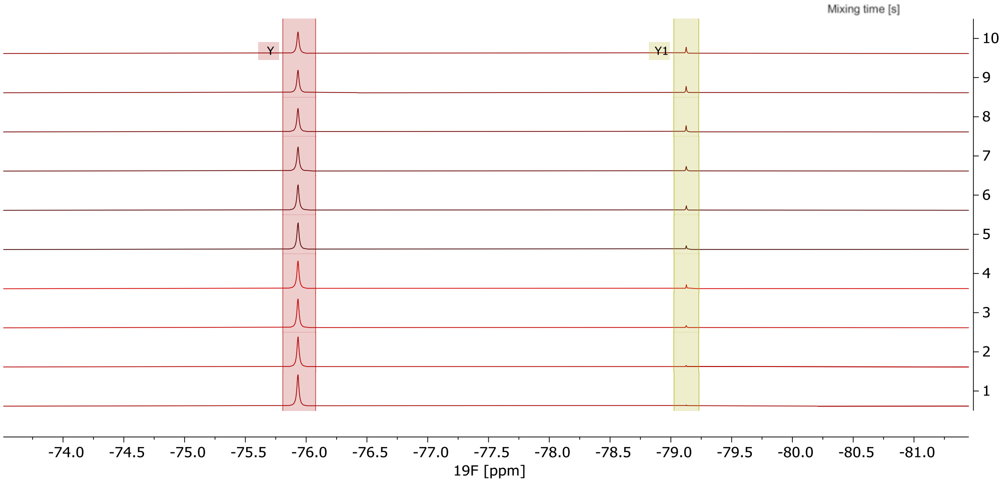

Figure S122: Raw ^{19}F EXSY data for the *α*-triflate dissociation in the corresponding *α*-triflate (left corner) measured at -80 °C at a concentration ⁻OTf of 0.098 M.

Selective 1D EXSY at -80 °C at 153.7 mM [$^{-}$OTf]

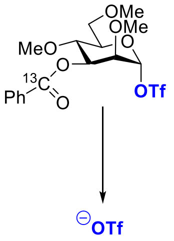

Spectrum number	Mixing Time [s]	Alpha (-76.0 ppm) resonance intensity	$^{-}$OTf (-79.1 ppm) resonance intensity
1	0.01	10821.4	19.352
2	0.02	10389.2	189.473
3	0.03	9916.84	301.145
4	0.04	9513.54	407.158
5	0.05	9148.35	471.908
6	0.06	8736.68	547.38
7	0.07	8409.44	651.559
8	0.08	8068.92	691.058
9	0.09	7756.99	793.072
10	0.1	7456.5	847.732

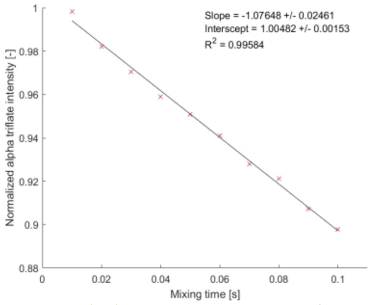

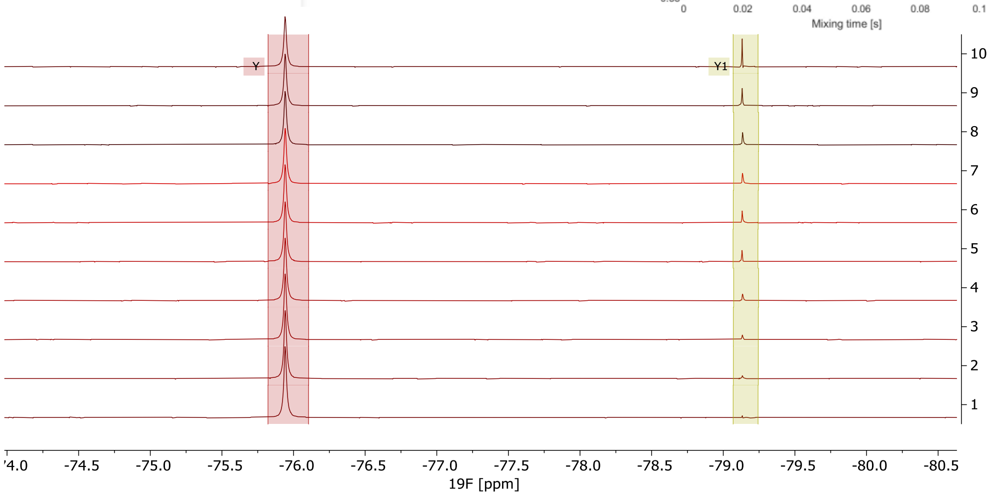

Figure S123: Raw ^{19}F EXSY data for the *α*-triflate dissociation in the corresponding *α*-triflate (left corner) measured at -80 °C at a concentration $^{-}$OTf of 0.15 M.

Selective 1D EXSY at -80 °C at 243.8 mM [$^{-}$OTf]

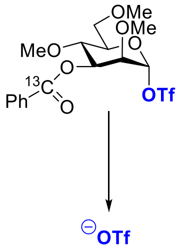

Spectrum number	Mixing Time [s]	Alpha (-76.0 ppm) resonance intensity	$^{-}$OTf (-79.1 ppm) resonance intensity
1	0.02	6882.02	142.483
2	0.03	6417.73	216.597
3	0.04	6304.65	201.853
4	0.05	6048.97	281.765
5	0.06	5808	372.329
6	0.07	5593.59	398.821
7	0.08	5301.84	433.376
8	0.09	5125.4	492.091
9	0.1	4887.17	518.449

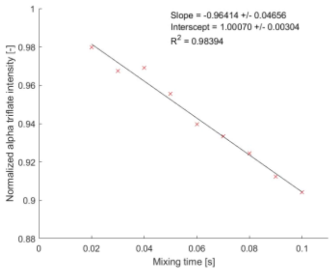

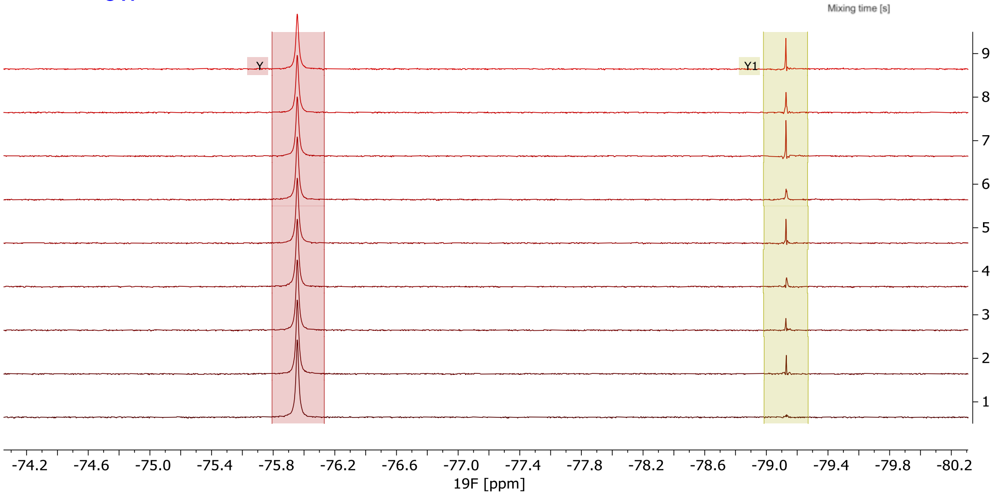

Figure S124: Raw ^{19}F EXSY data for the *α*-triflate dissociation in the corresponding *α*-triflate (left corner) measured at -80 °C at a concentration $^{-}$OTf of 0.24 M.

^{13}C CEST at -80 °C at 32.2 mM [$^{-}$OTf]

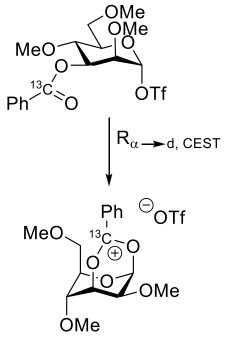

Spectrum number	Saturation time [s]	Intensity ^{13}C=O (165 ppm)
1	0.1	73.371
2	0.2	66.4071
3	0.3	62.7869
4	0.4	58.3101
5	0.5	55.0579
6	0.6	52.1146
7	0.7	49.9214
8	0.8	47.9011
9	0.9	45.8388
10	1	44.4001
11	4	31.6452
12	0	79.7656

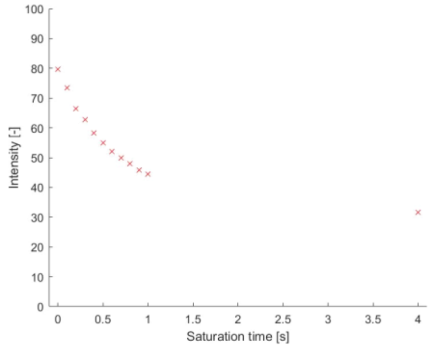

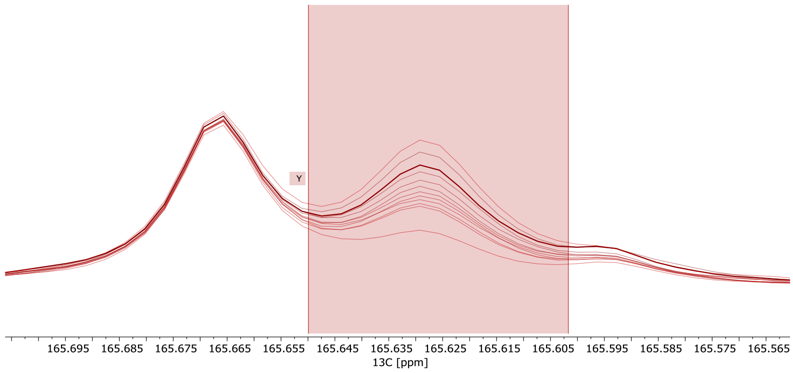

Figure S125: Raw ^{13}C CEST data for the dioxanium ion formation from the corresponding α-triflate (left corner) measured at -80 °C at a concentration $^{-}$OTf of 0.032 M.

^{13}C CEST at -80 °C at 98.0 mM [$^{-}$OTf]

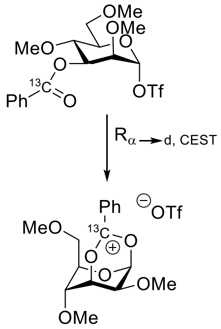

Spectrum number	Saturation time [s]	Intensity ^{13}C=O (165 ppm)
1	0.1	58.7993
2	0.2	54.6272
3	0.3	50.4608
4	0.4	46.7378
5	0.5	45.6735
6	0.6	41.601
7	0.7	39.9795
8	0.8	37.351
9	0.9	37.4448
10	1	33.9365
11	4	26.2765
12	0	63.8645

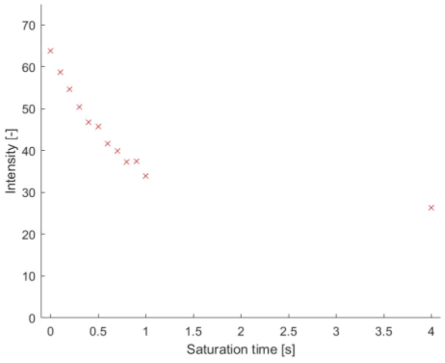

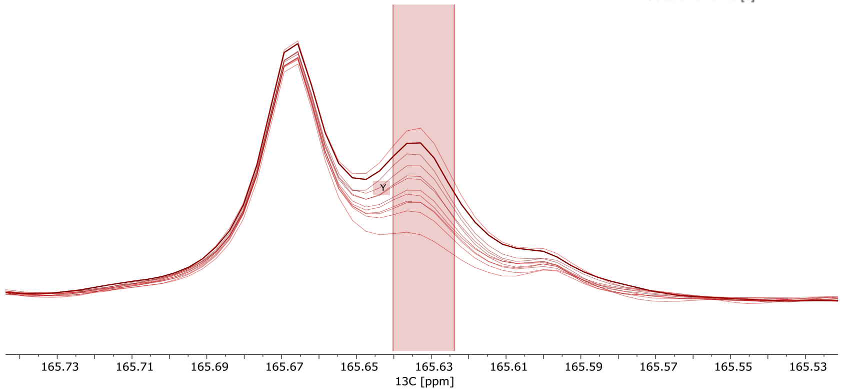

Figure S126: Raw ^{13}C CEST data for the dioxanium ion formation from the corresponding α-triflate (left corner) measured at -80 °C at a concentration $^{-}$OTf of 0.098 M.

^{13}C CEST at -80 ^{o}C at 153.7 mM [$^{-}$OTf]

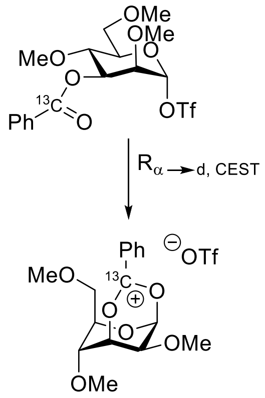

Spectrum number	Saturation time [s]	Intensity ^{13}C=O (165 ppm)
1	0.1	191.561
2	0.2	180.191
3	0.3	166.708
4	0.4	154.144
5	0.5	153.263
6	0.6	141.816
7	0.7	132.63
8	0.8	133.416
9	0.9	128.872
10	1	124.608
11	4	97.8592
12	0	207.775

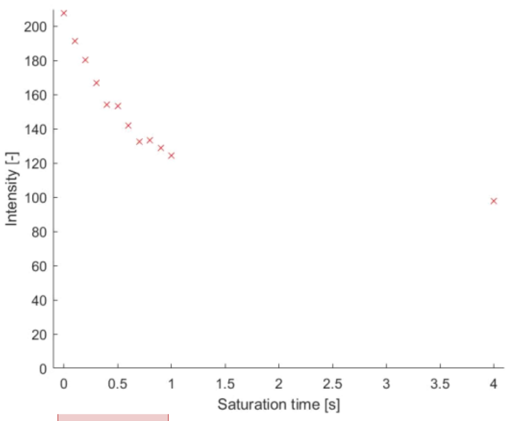

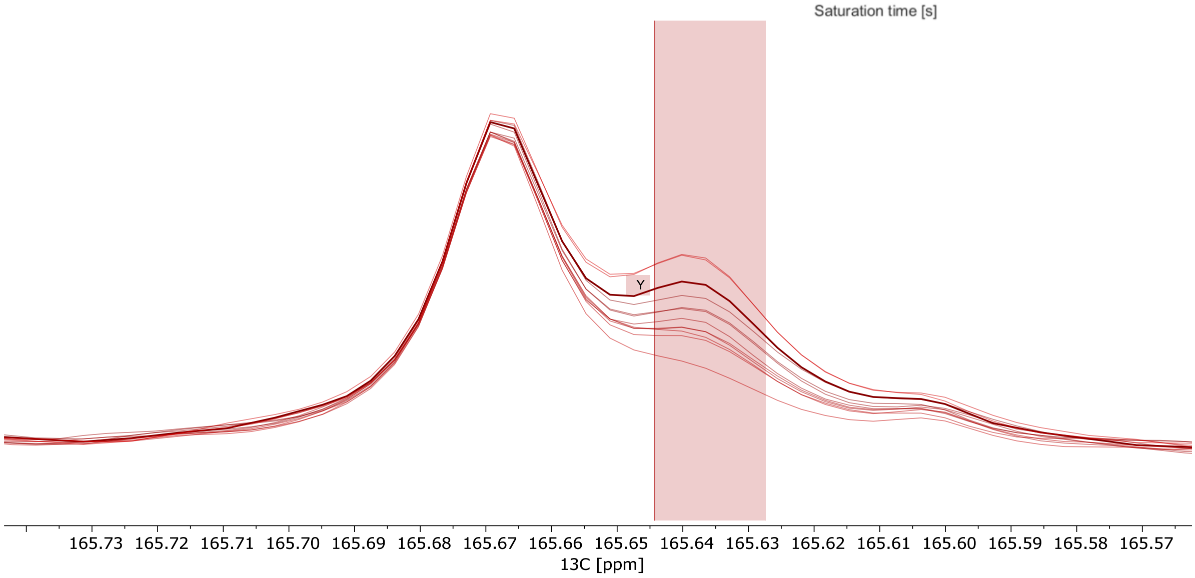

Figure S127: Raw ^{13}C CEST data for the dioxanium ion formation from the corresponding α-triflate (left corner) measured at -80 °C at a concentration $^{-}$OTf of 0.15 M.

Synthesis spectra

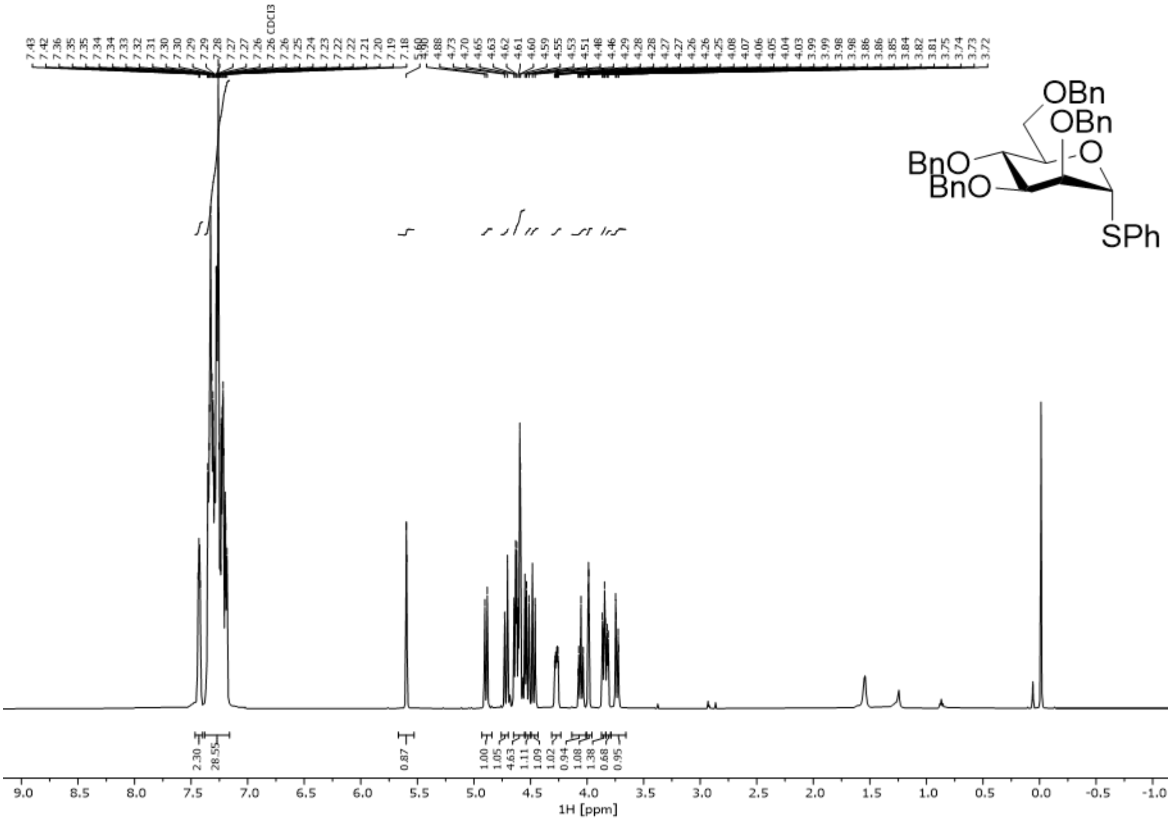

Figure S128: ^{1}H NMR of phenyl 2,3,4,6-tetra-O-benzyl-1-thio-α-D-mannopyranoside.

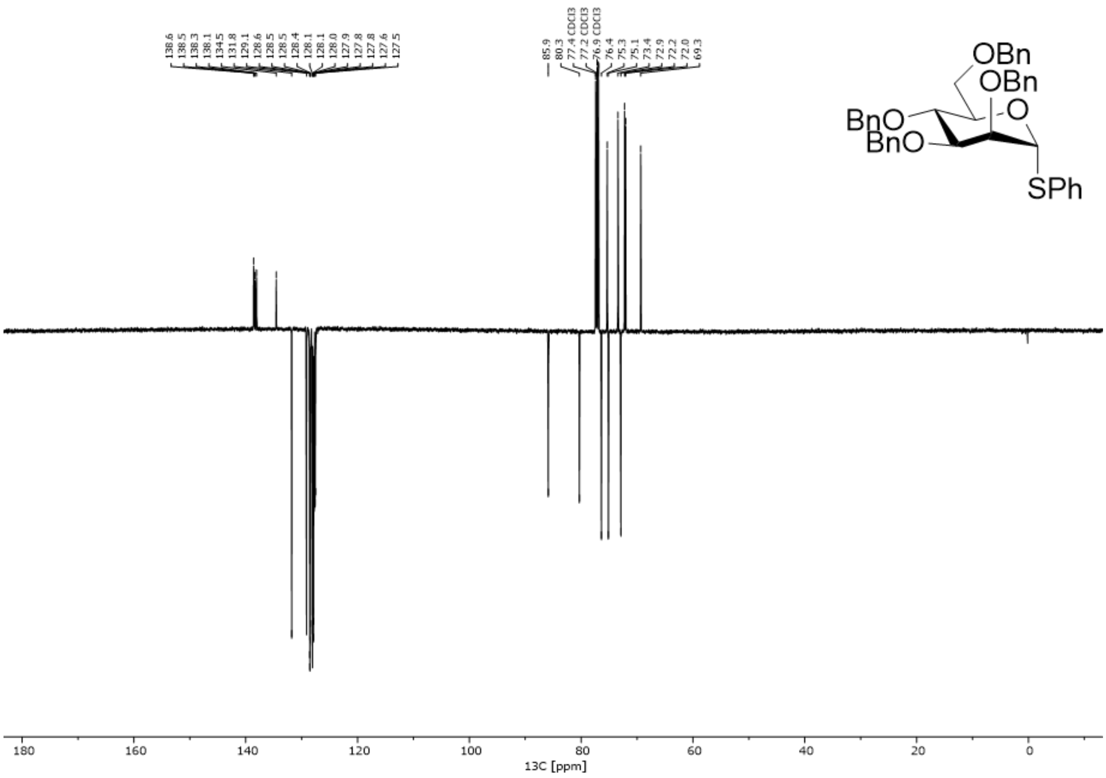

Figure S129: ^{13}C NMR of phenyl 2,3,4,6-tetra-O-benzyl-1-thio-α-D-mannopyranoside.

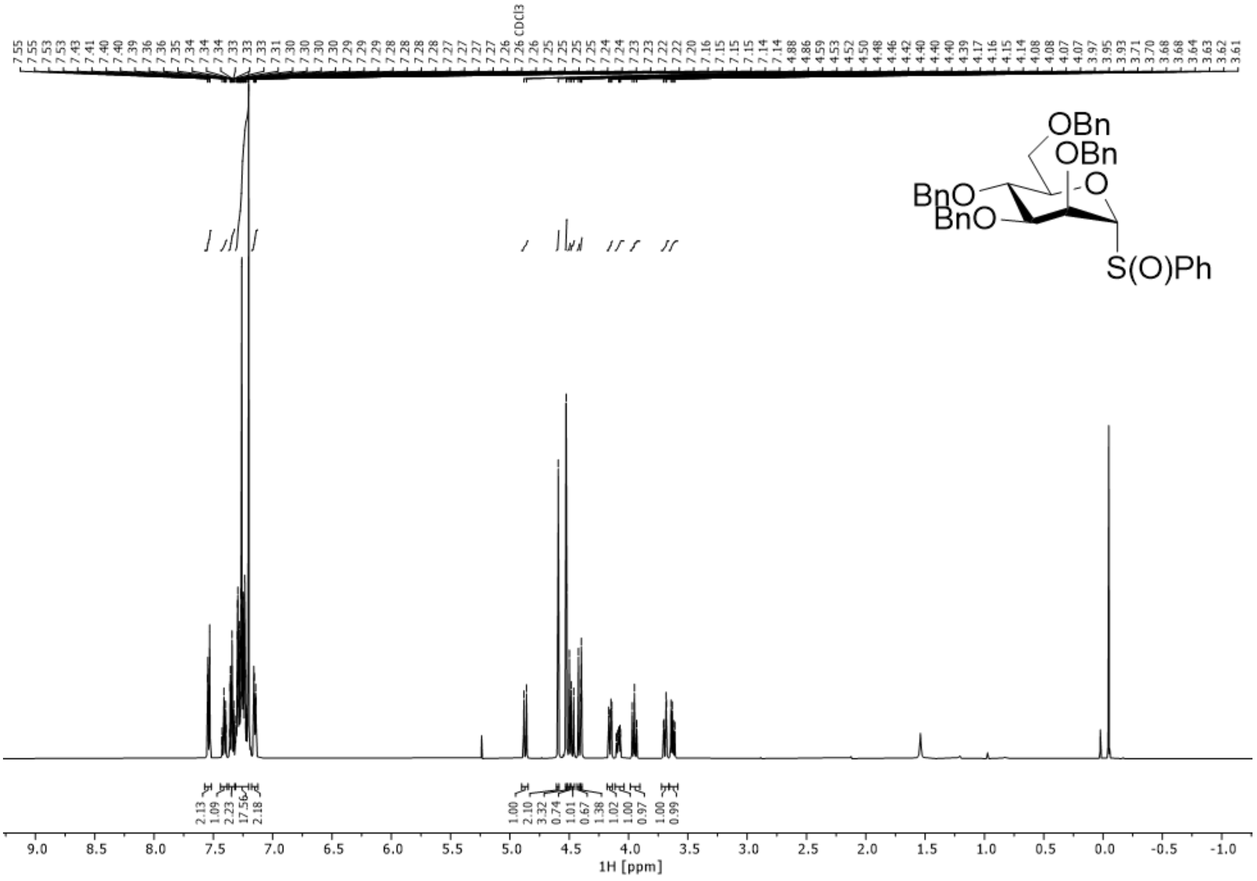

Figure S130: ^{1}H NMR of phenyl 2,3,4,6-tetra-O-benzyl-1-thiosulfinyl-α-D-mannopyranoside**.**

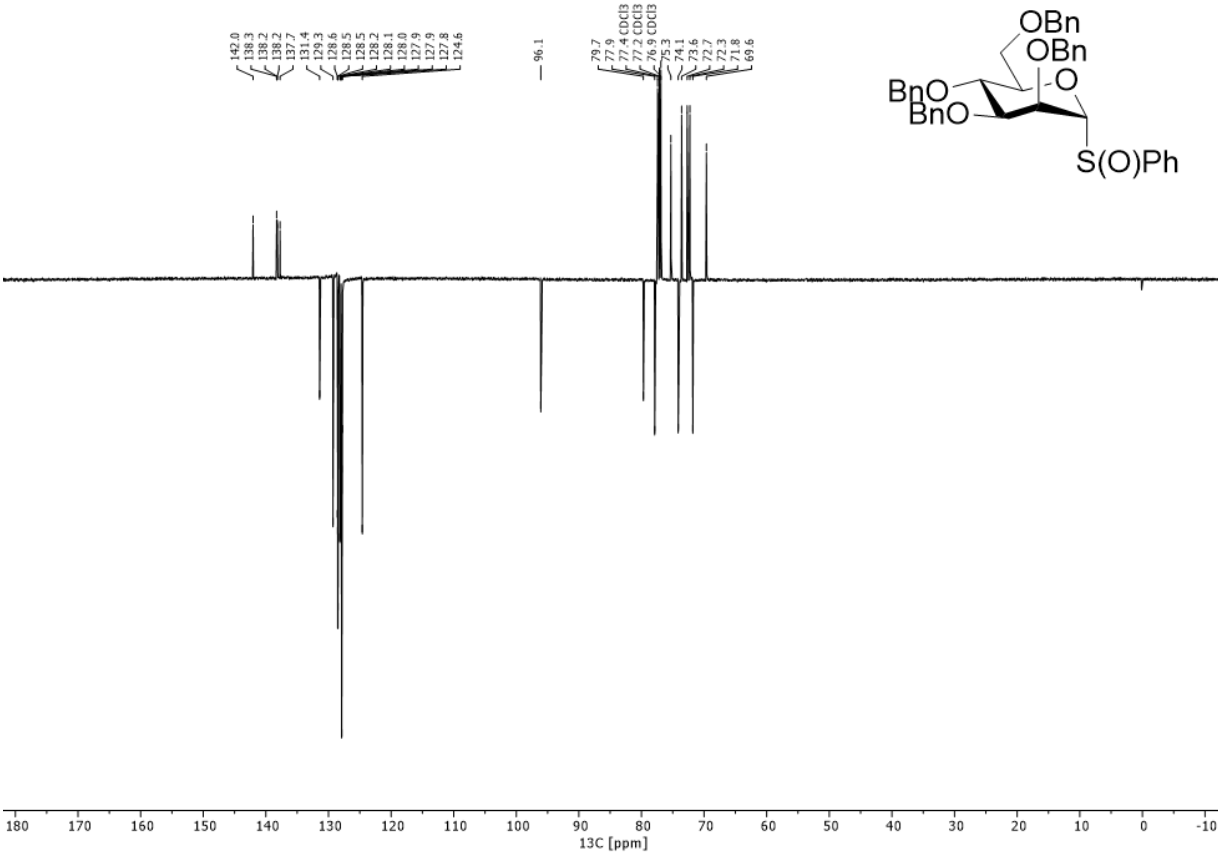

Figure S131: ^{13}C NMR of phenyl 2,3,4,6-tetra-O-benzyl-1-thiosulfinyl-α-D-mannopyranoside.

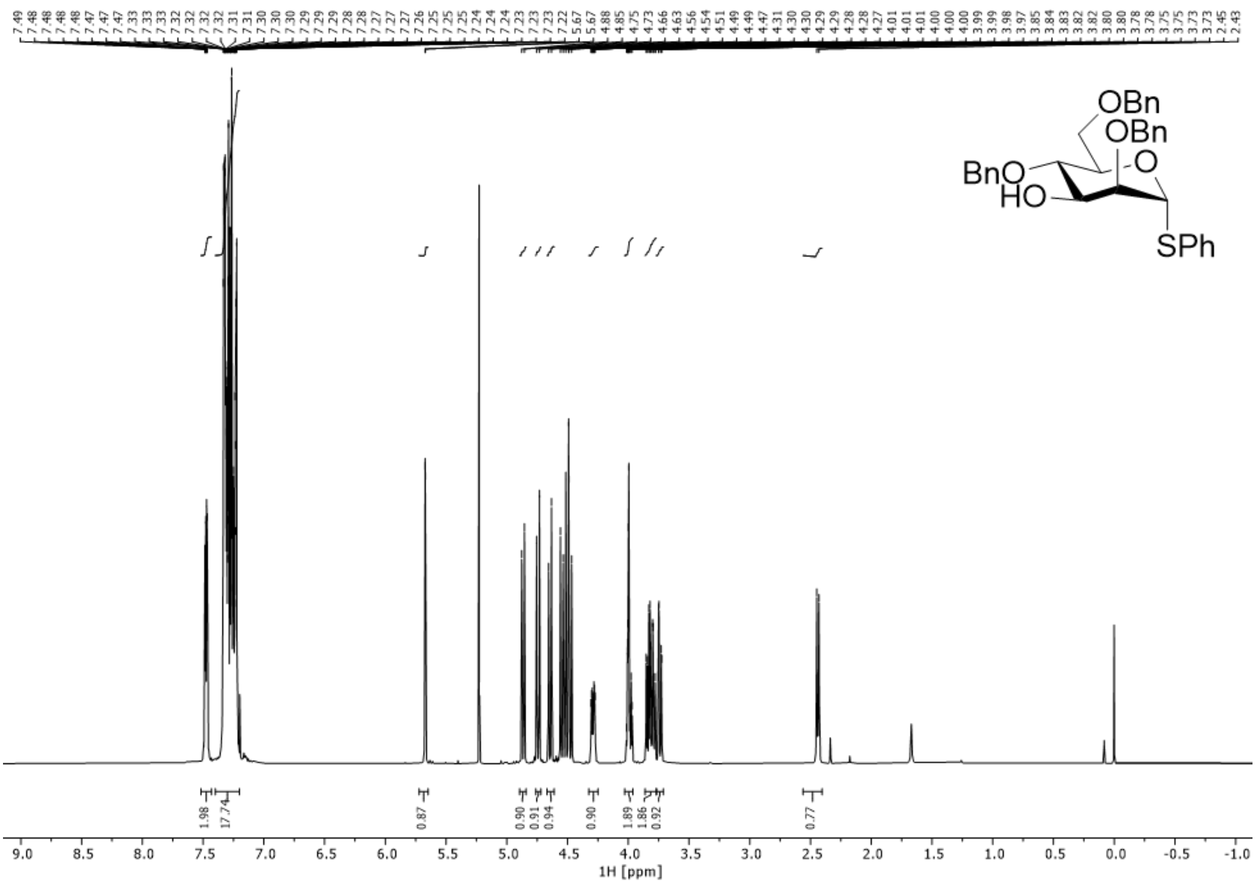

Figure S132: ^{1}H NMR of phenyl 2,4,6-tri-O-benzyl-1-thio-α-D-mannopyranoside.

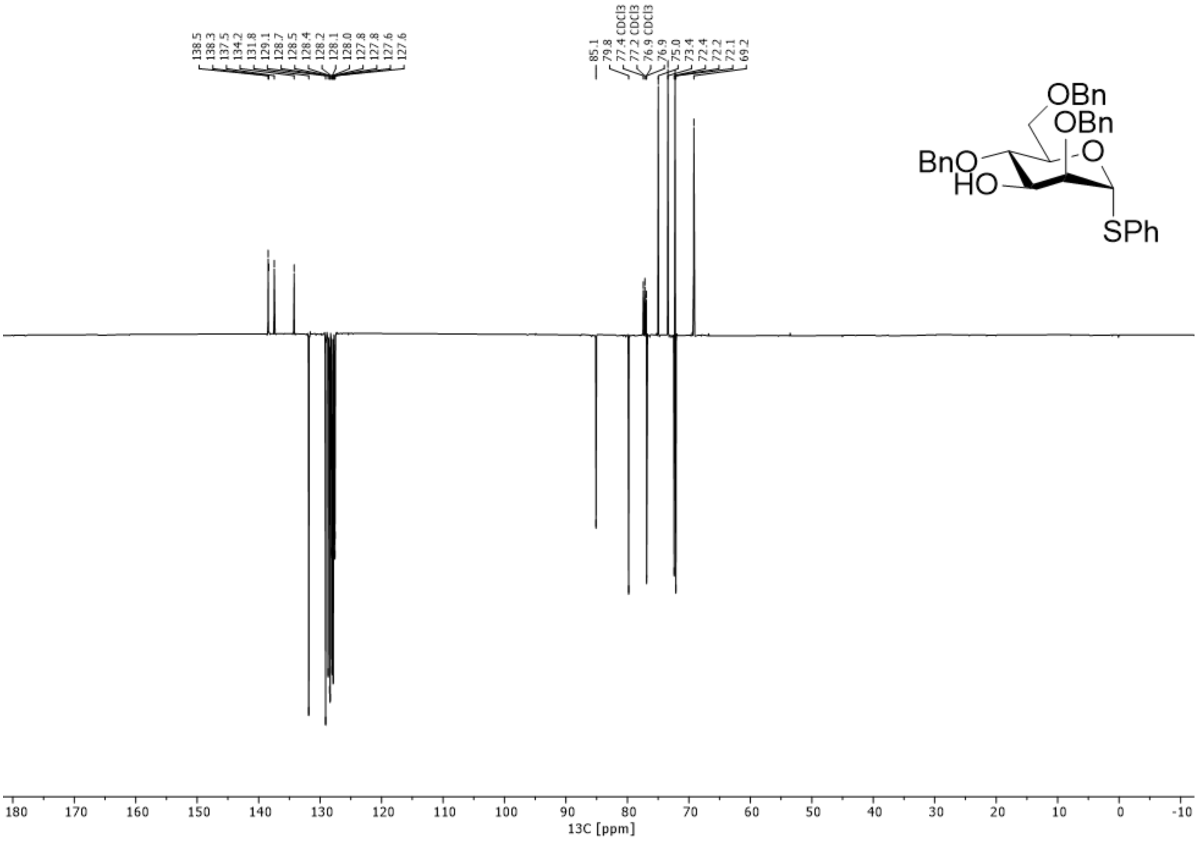

Figure S133: ^{13}C NMR of phenyl 2,4,6-tri-O-benzyl-1-thio-α-D-mannopyranoside.

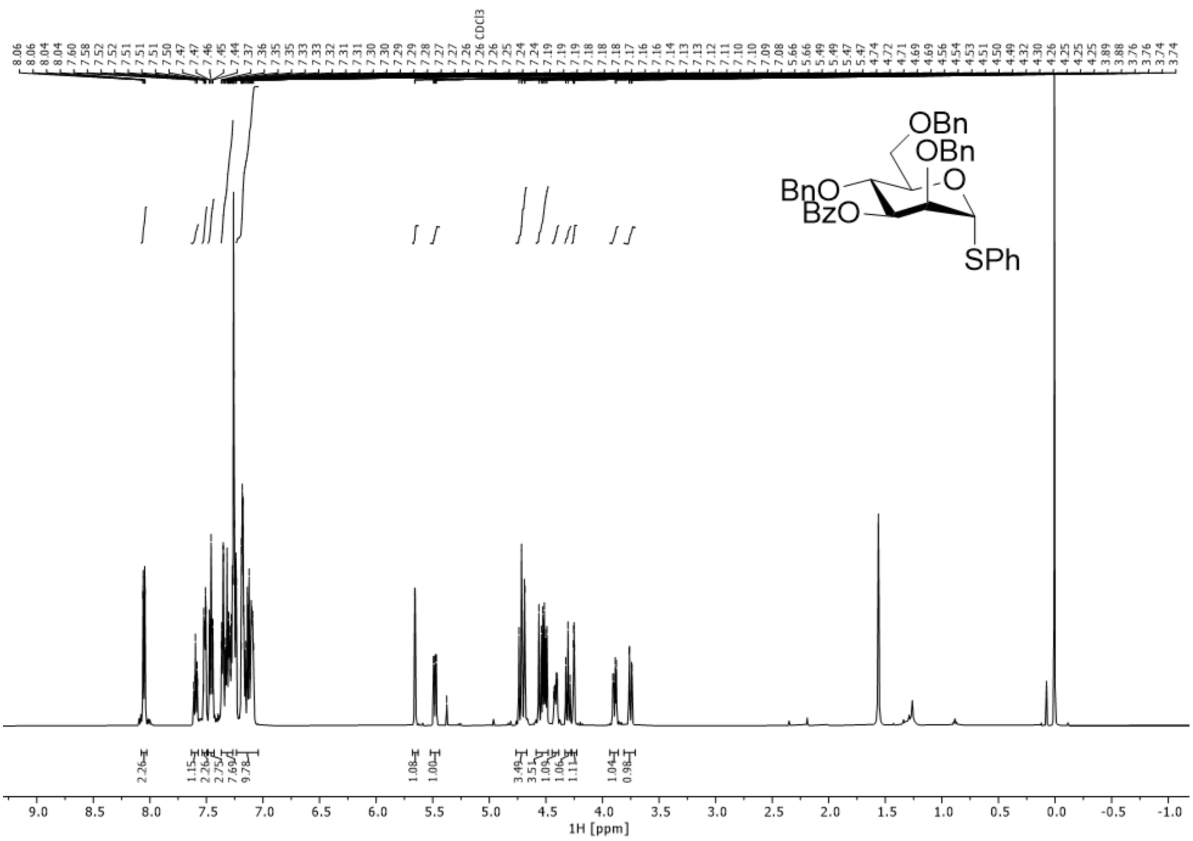

Figure S134: ^{1}H NMR of phenyl 3-O-benzoyl-2,4,6-tri-O-benzyl-1-thio-α-D-mannopyranoside.

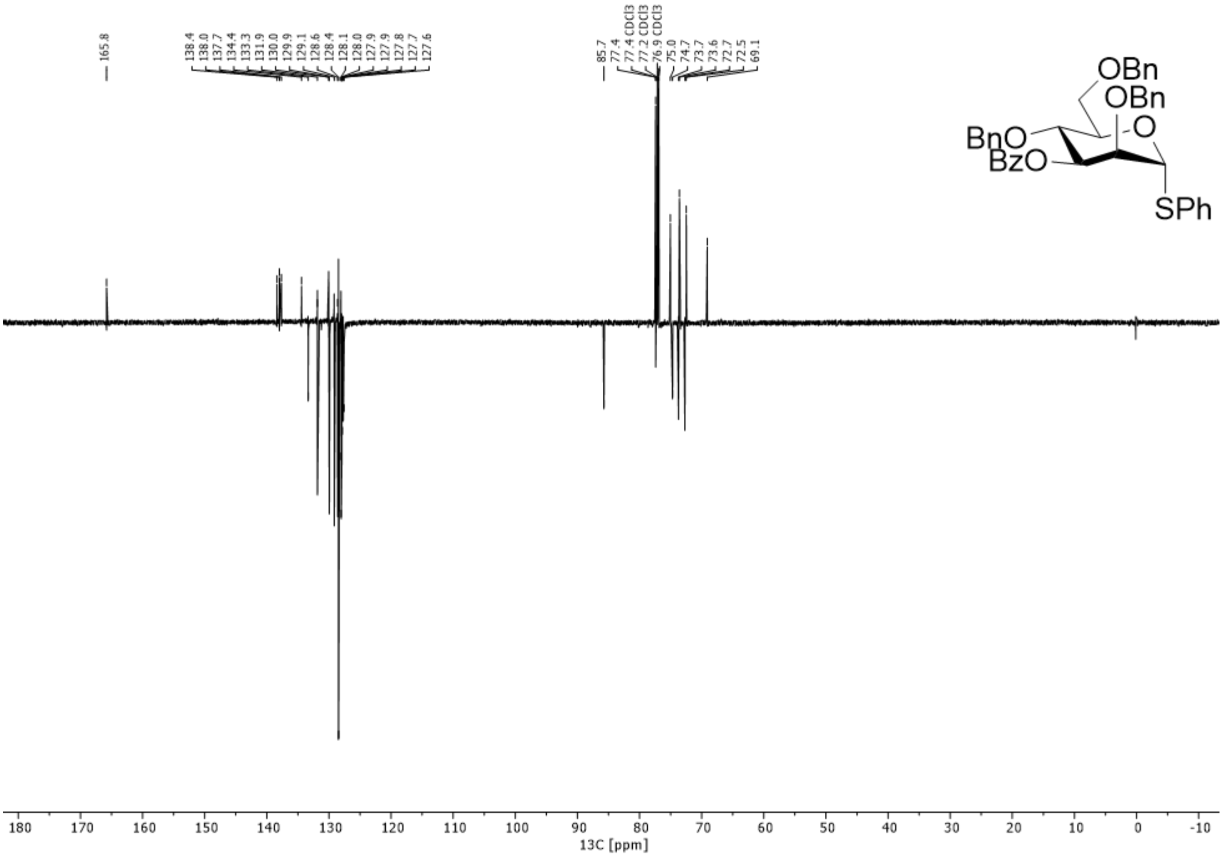

Figure S135: ^{13}C NMR of phenyl 3-O-benzoyl-2,4,6-tri-O-benzyl-1-thio-α-D-mannopyranoside.

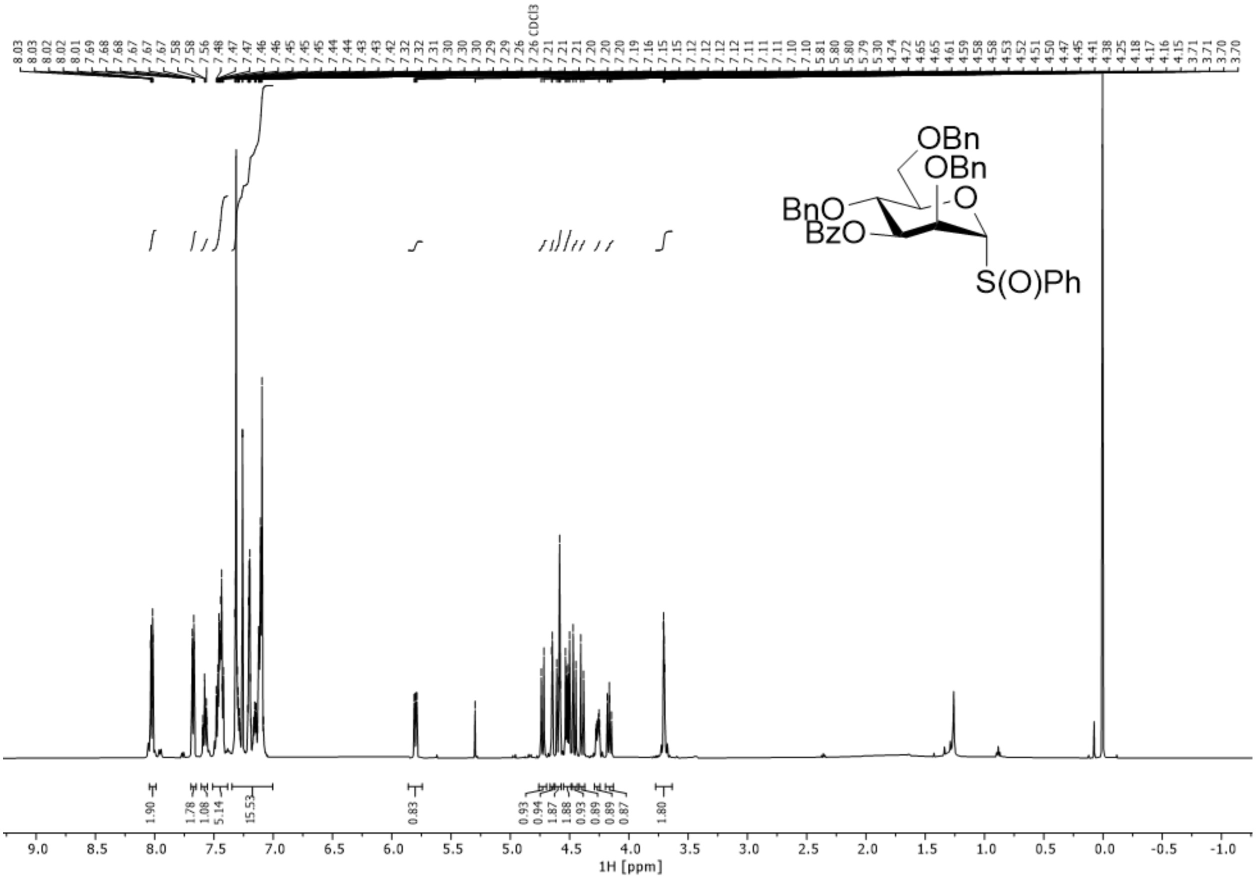

Figure S136: ^{1}H NMR of phenyl 3-O-benzoyl-2,4,6-tri-O-benzyl-1-thiosulfinyl-α-D-mannopyranoside.

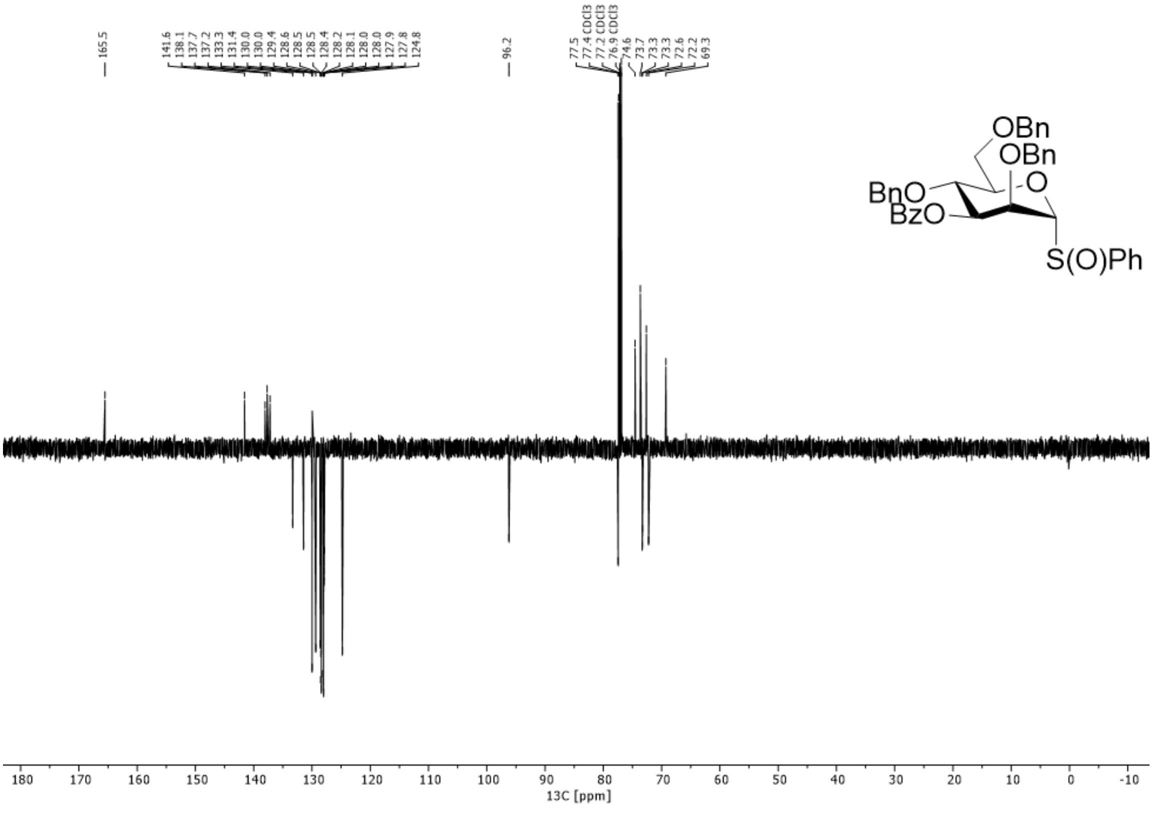

Figure S137: ^{13}C NMR of phenyl 3-O-benzoyl-2,4,6-tri-O-benzyl-1-thiosulfinyl-α-D-mannopyranoside.

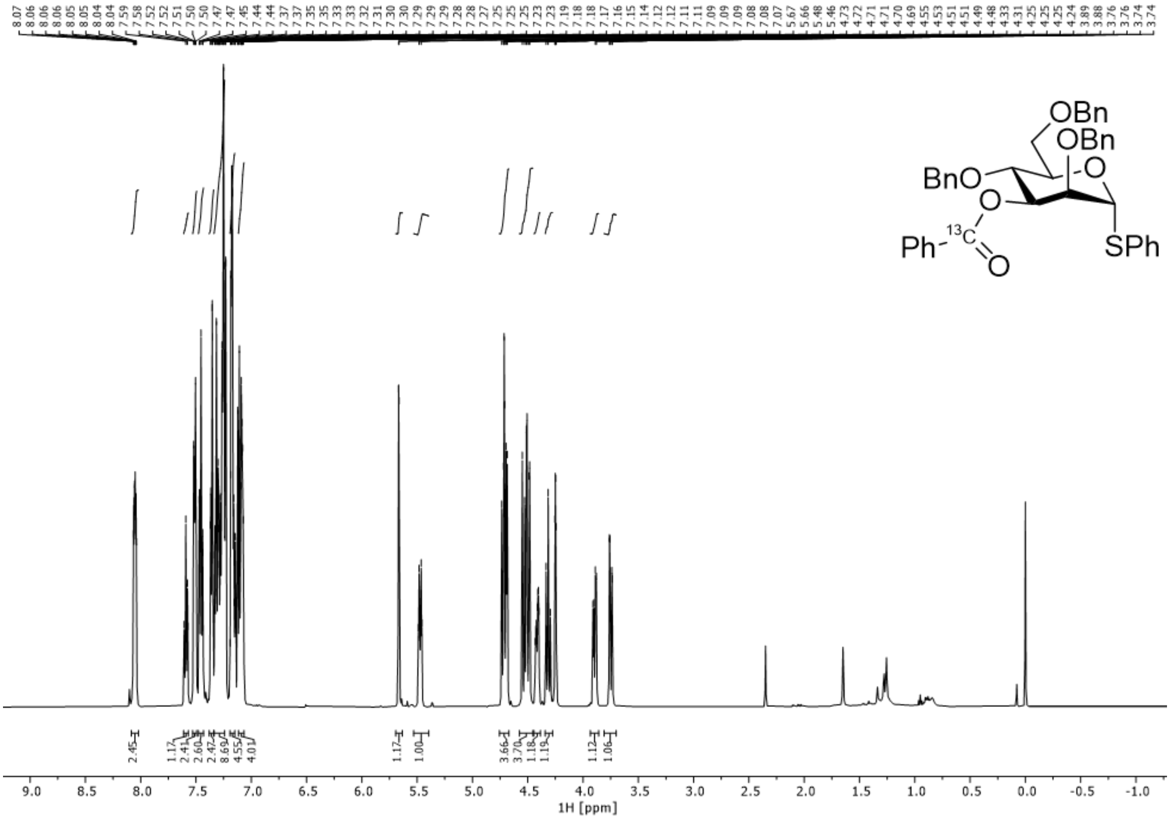

Figure S138: ^{1}H NMR of phenyl 3-O-(benzoyl-α- ^{13}C)-2,4,6-tri-O-benzyl-1-thio-α-D-mannopyranoside.

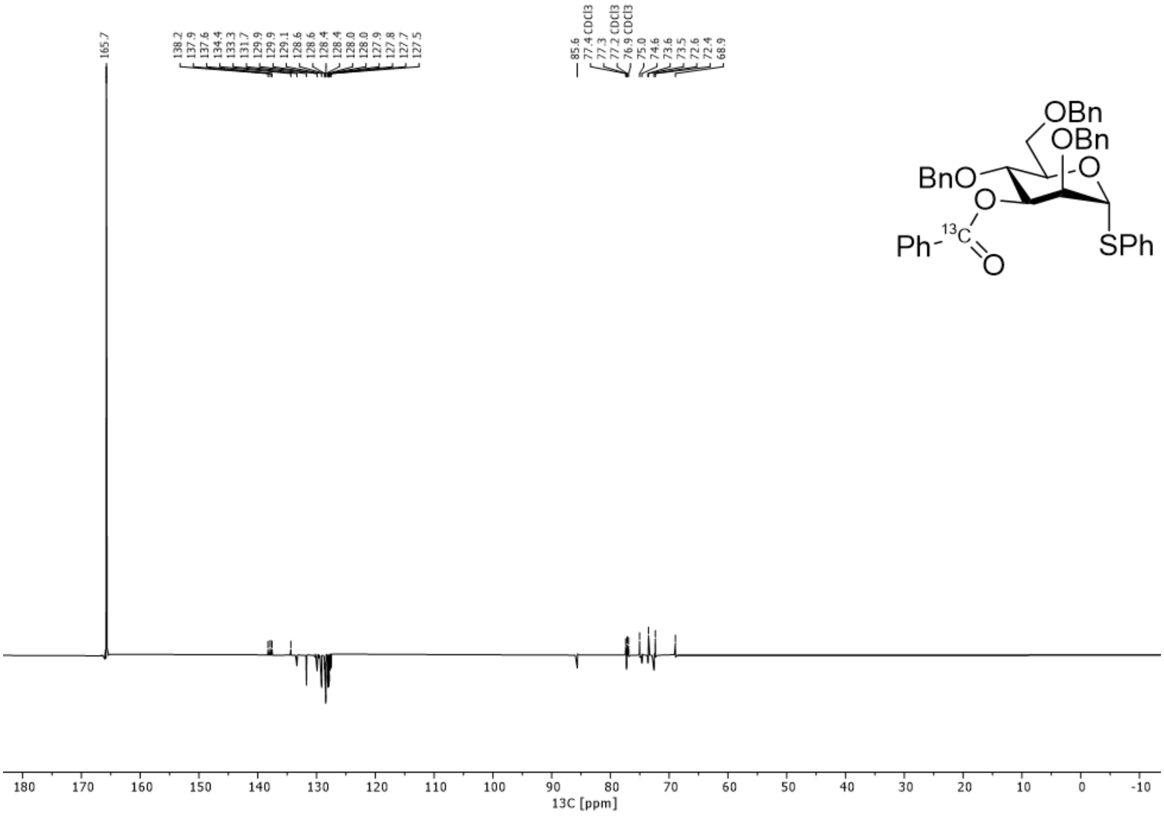

Figure S139: ^{13}C NMR of phenyl 3-O-(benzoyl-α- ^{13}C)-2,4,6-tri-O-benzyl-1-thio-α-D-mannopyranoside.

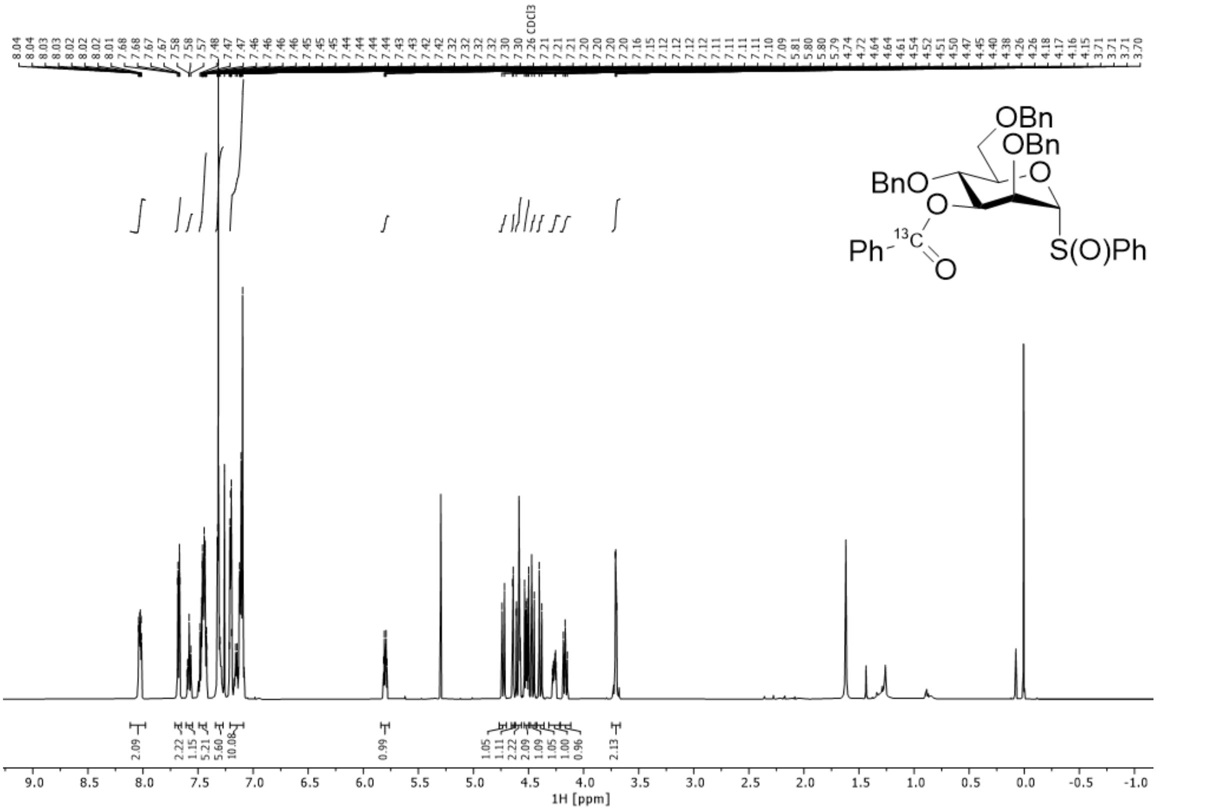

Figure S140: ^{1}H NMR of phenyl 3-O-(benzoyl-α-^{13}C)-2,4,6-tri-O-benzyl-1-thiosulfinyl-α-D-mannopyranoside.

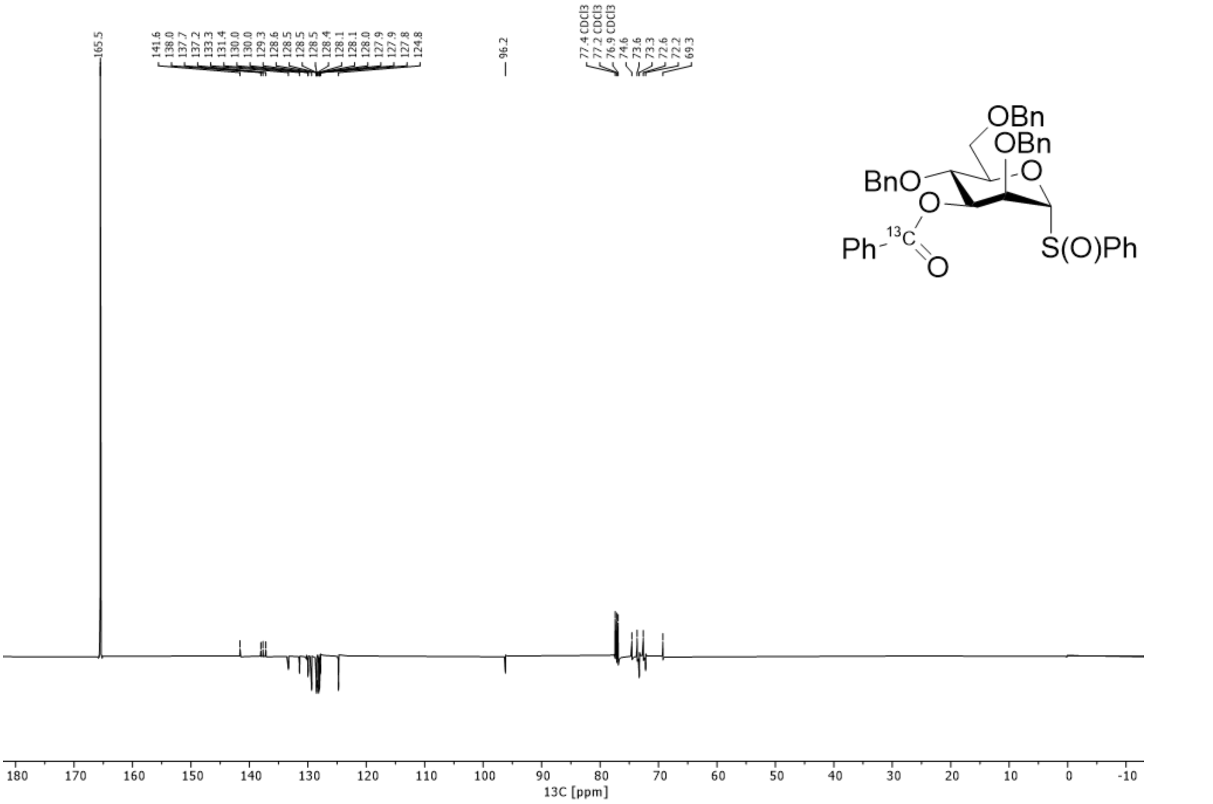

Figure S141: ^{13}C NMR of phenyl 3-O-(benzoyl-α-^{13}C)-2,4,6-tri-O-benzyl-1-thiosulfinyl-α-D-mannopyranoside.

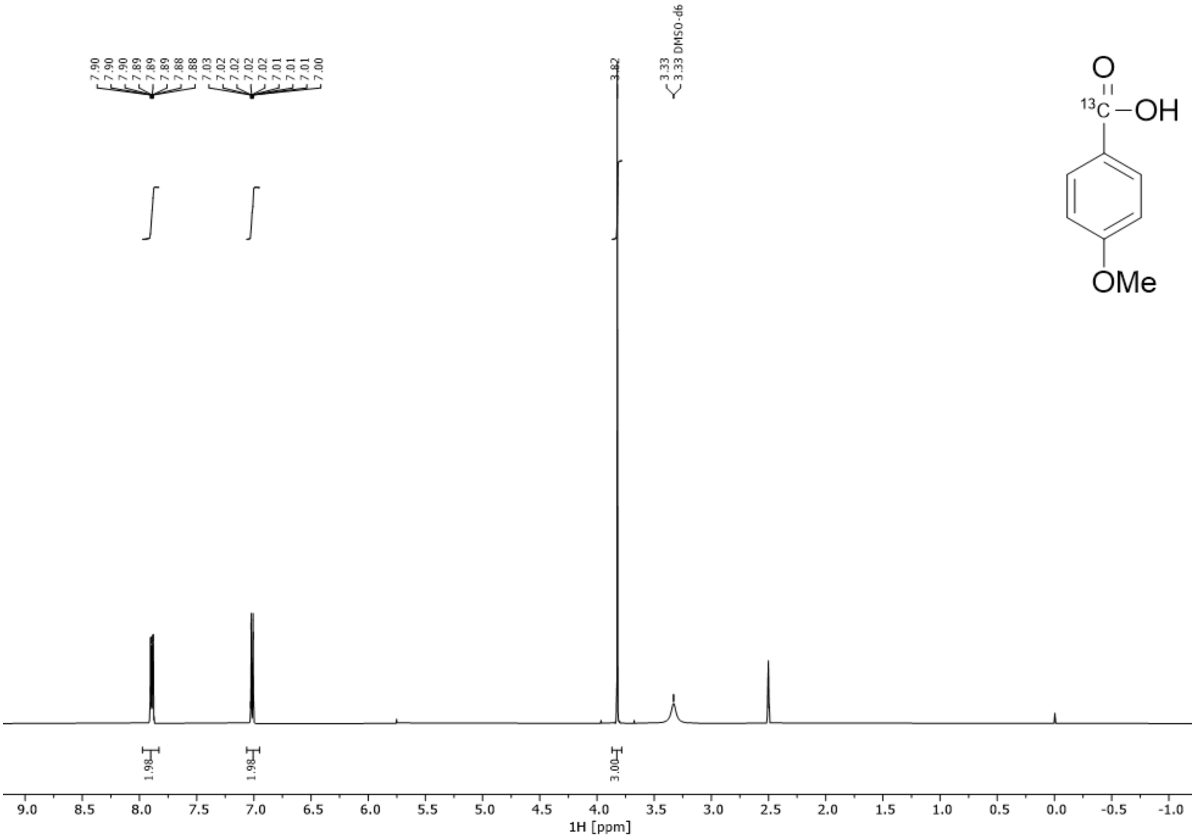

Figure S142: ^{1}H NMR of 4-methoxy-[α-^{13}C]-benzoic acid.

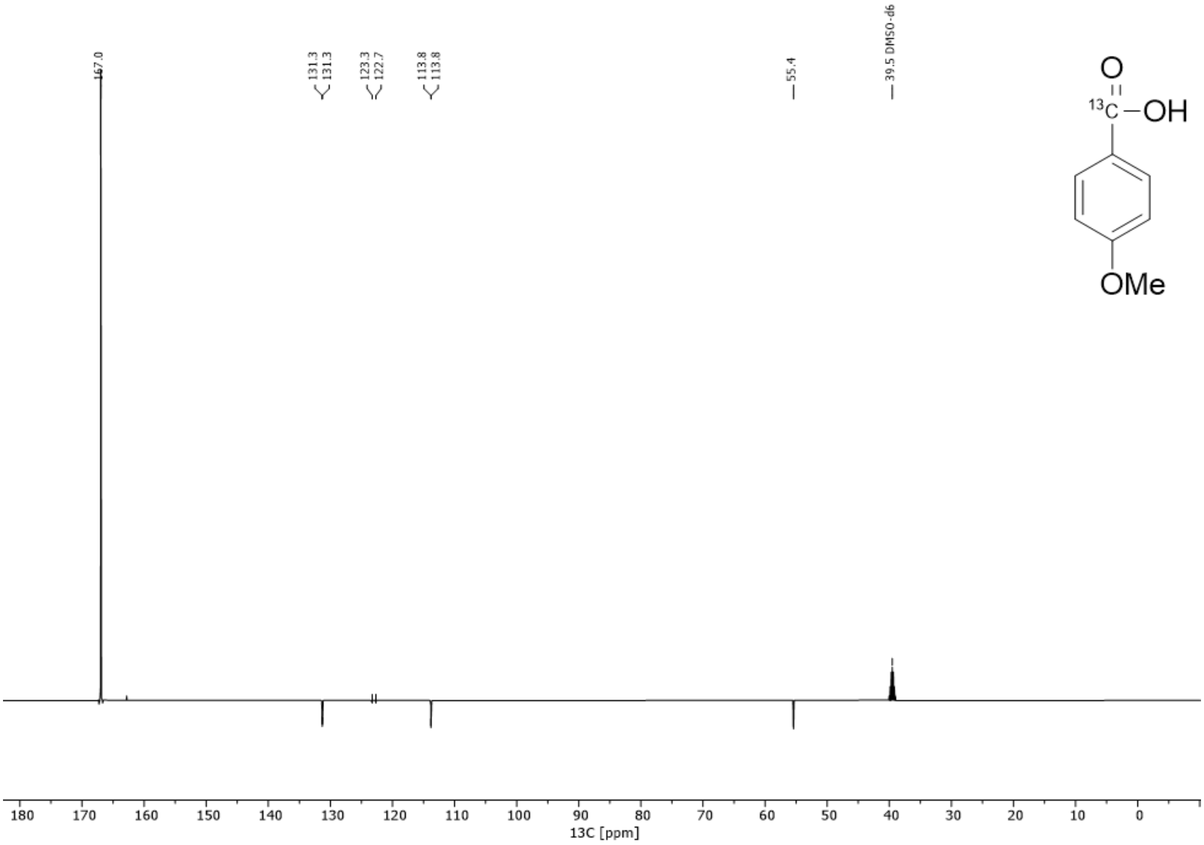

Figure S143: ^{13}C NMR of 4-methoxy-[α-^{13}C]-benzoic acid.

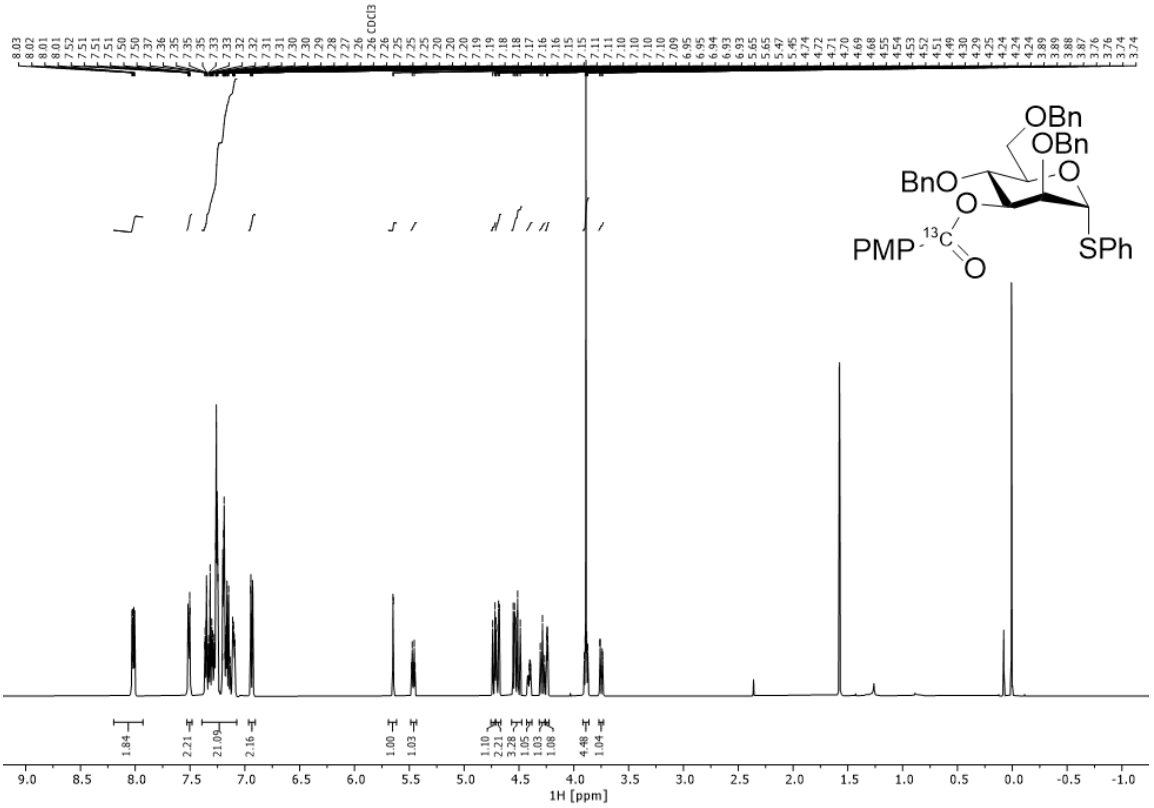

Figure S144: ^{1}H NMR of phenyl 3-*O*-(4-methoxybenzoyl-α-^{13}C)-2,4,6-tri-*O*-benzyl-1-thio-α-D-mannopyranoside.

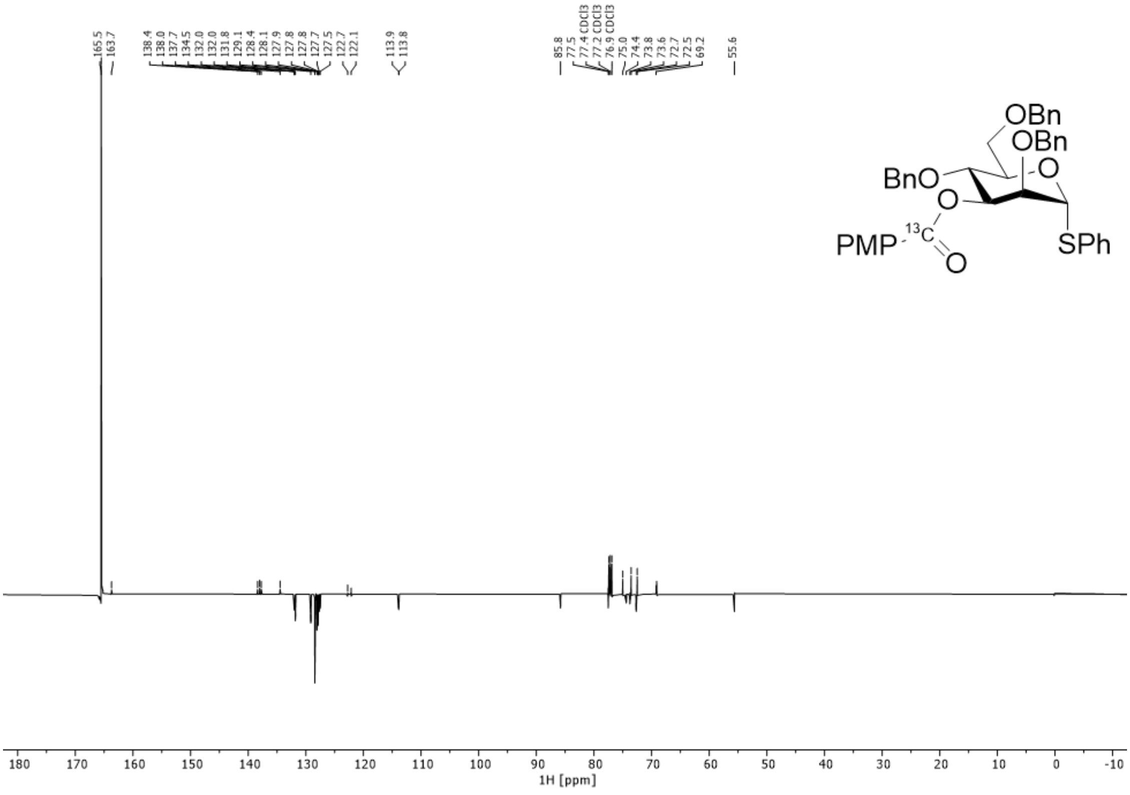

Figure S145: ^{13}C NMR of phenyl 3-*O*-(4-methoxybenzoyl-α-^{13}C)-2,4,6-tri-*O*-benzyl-1-thio-α-D-mannopyranoside.

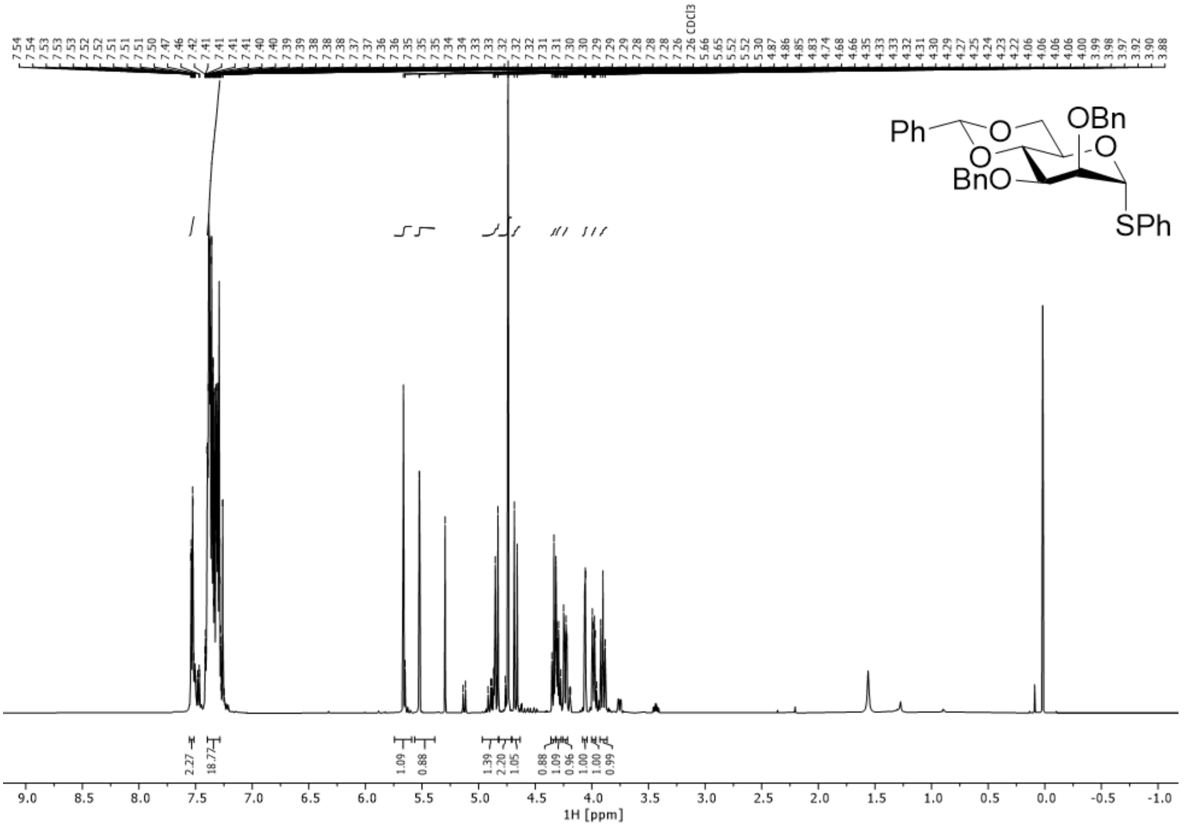

Figure S146: ^{1}H NMR of phenyl 2,3-di-*O*-benzyl-4,6-*O*-benzylidene-1-thio-α-D-mannopyranoside.

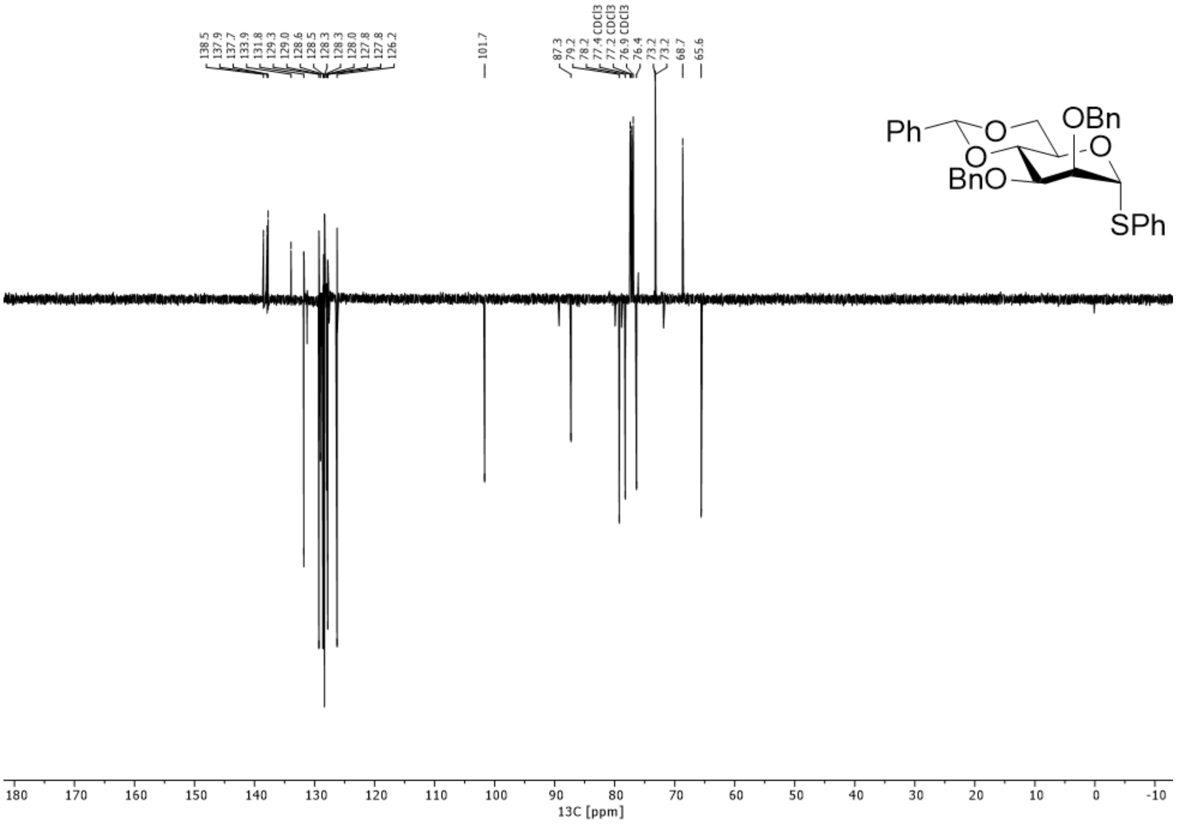

Figure S147: ^{13}C NMR of phenyl 2,3-di-*O*-benzyl-4,6-*O*-benzylidene-1-thio-α-D-mannopyranoside.

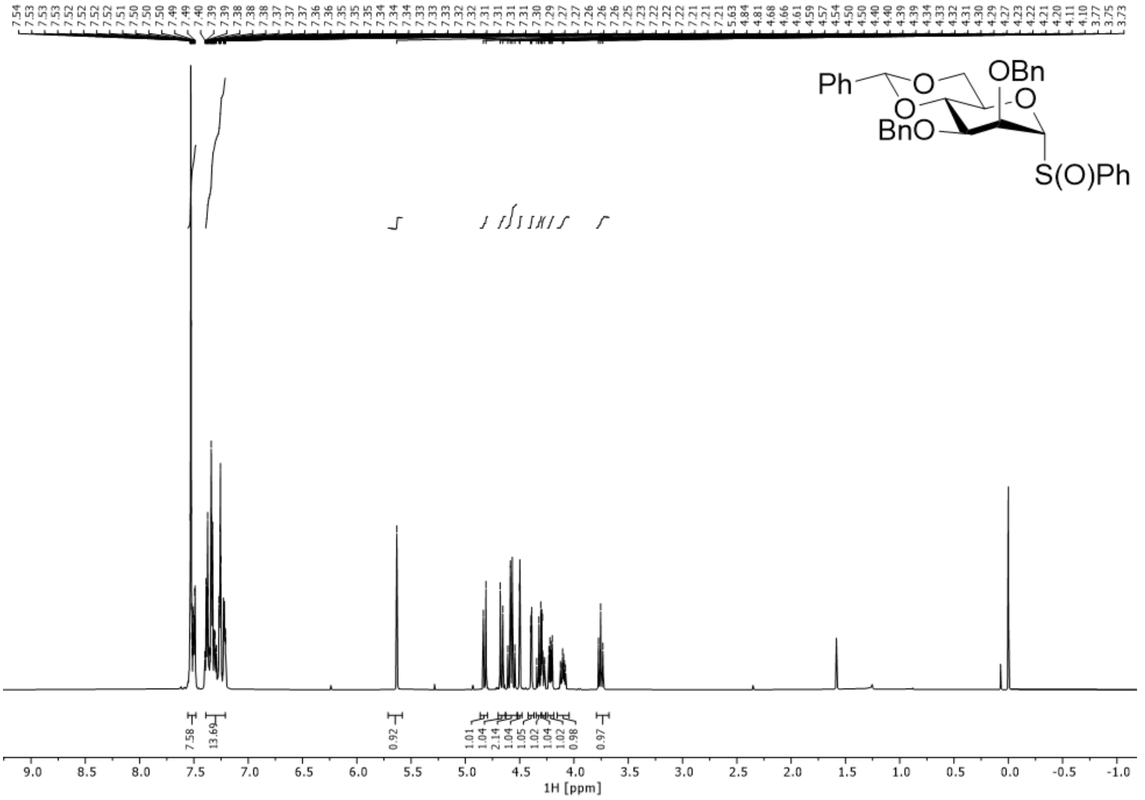

Figure S148: ^{1}H NMR of phenyl 2,3-di-*O*-benzyl-4,6-*O*-benzylidene-1-thiosulfinyl-α-D-manno-pyranoside.

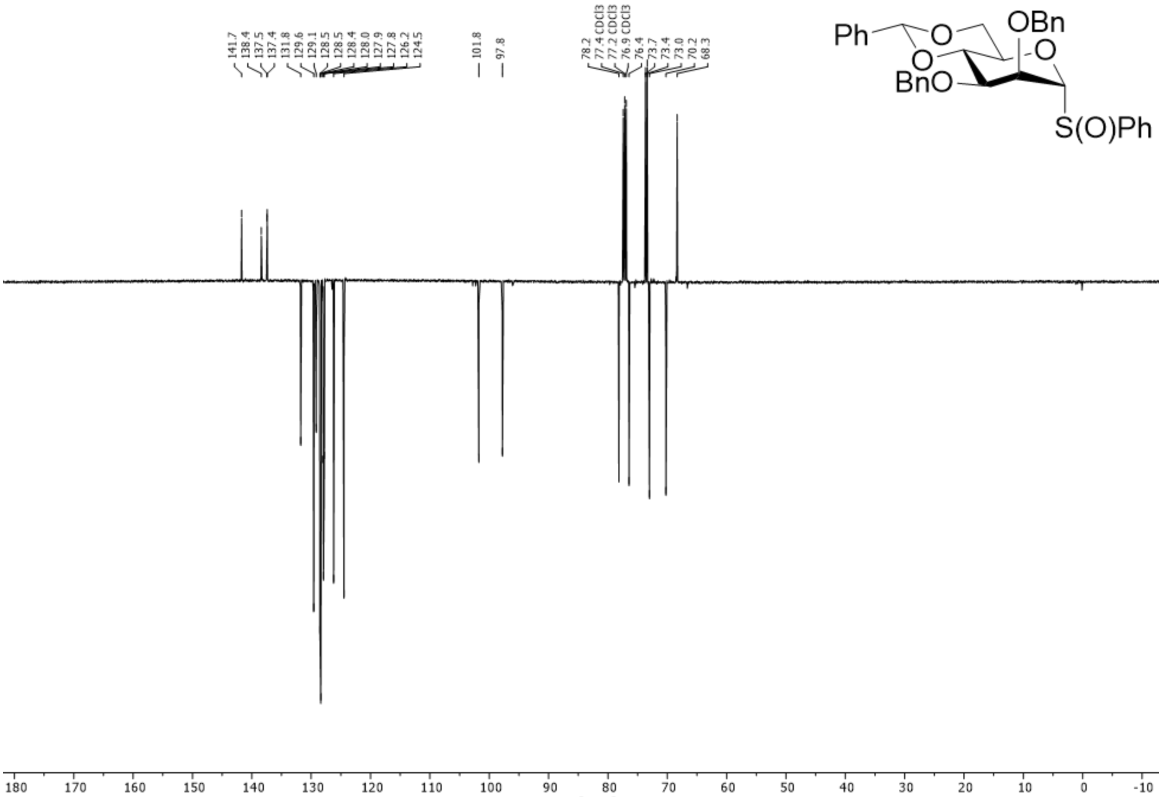

Figure S149: ^{13}C NMR of phenyl 2,3-di-O-benzyl-4,6-O-benzylidene-1-thiosulfinyl-α-D-mannopyranoside.

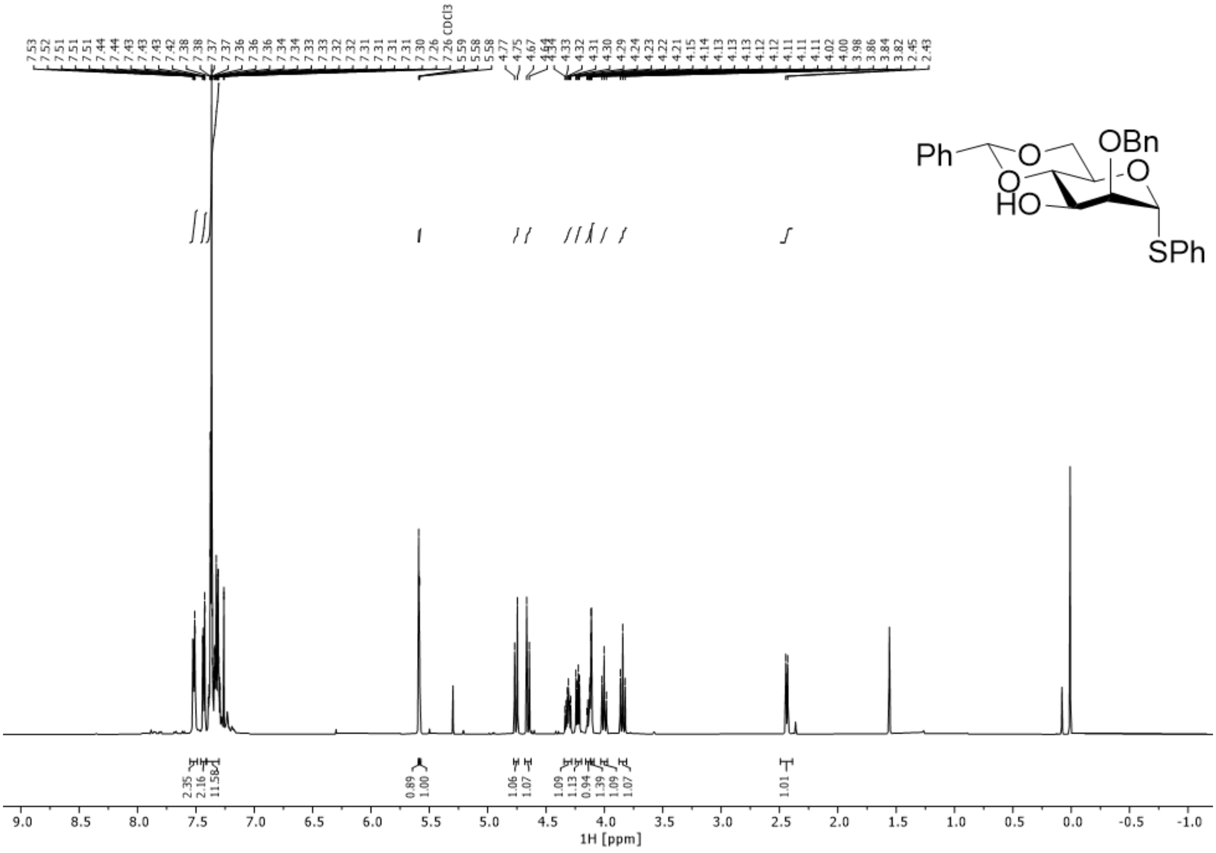

Figure S150: ^{1}H NMR of phenyl 2-*O*-benzyl-4,6-*O*-benzylidene-1-thio-α-D-mannopyranoside.

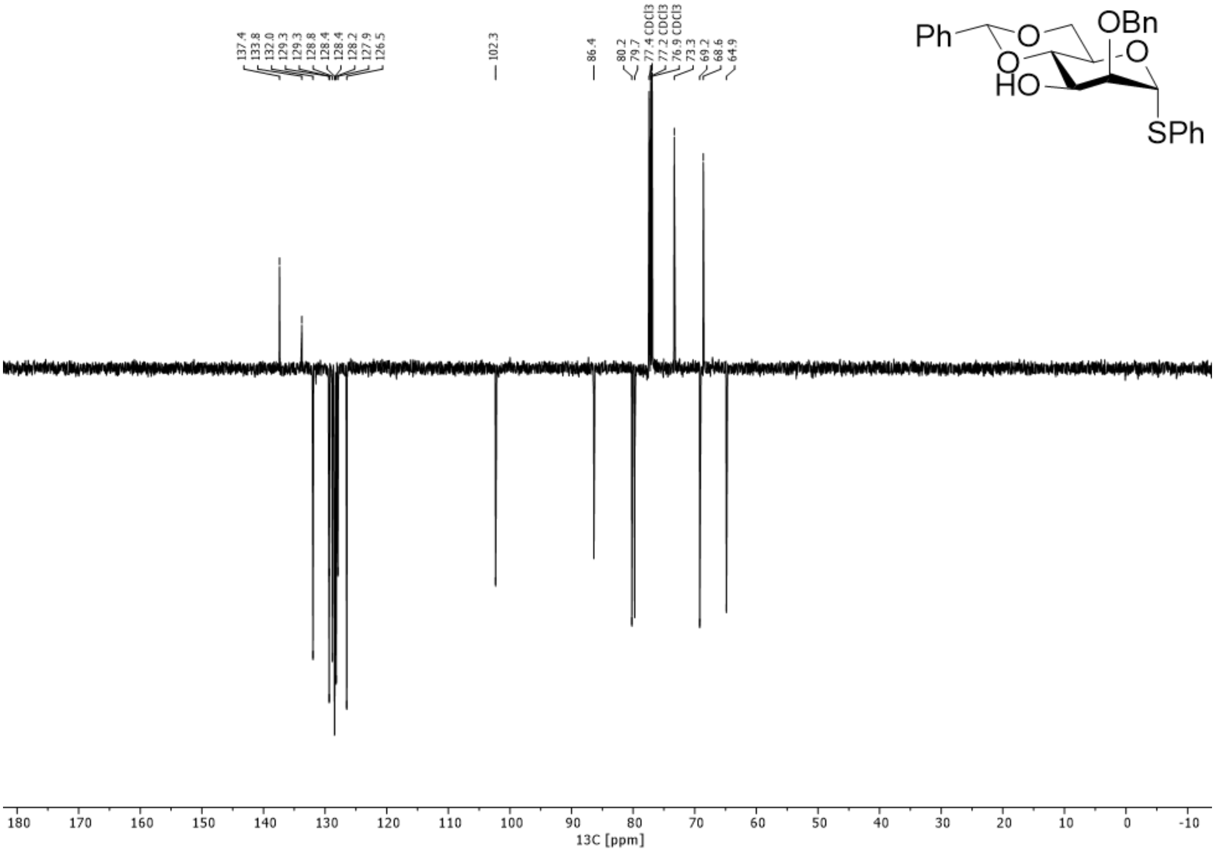

Figure S151: ^{13}C NMR of phenyl 2-*O*-benzyl-4,6-*O*-benzylidene-1-thio-α-D-mannopyranoside.

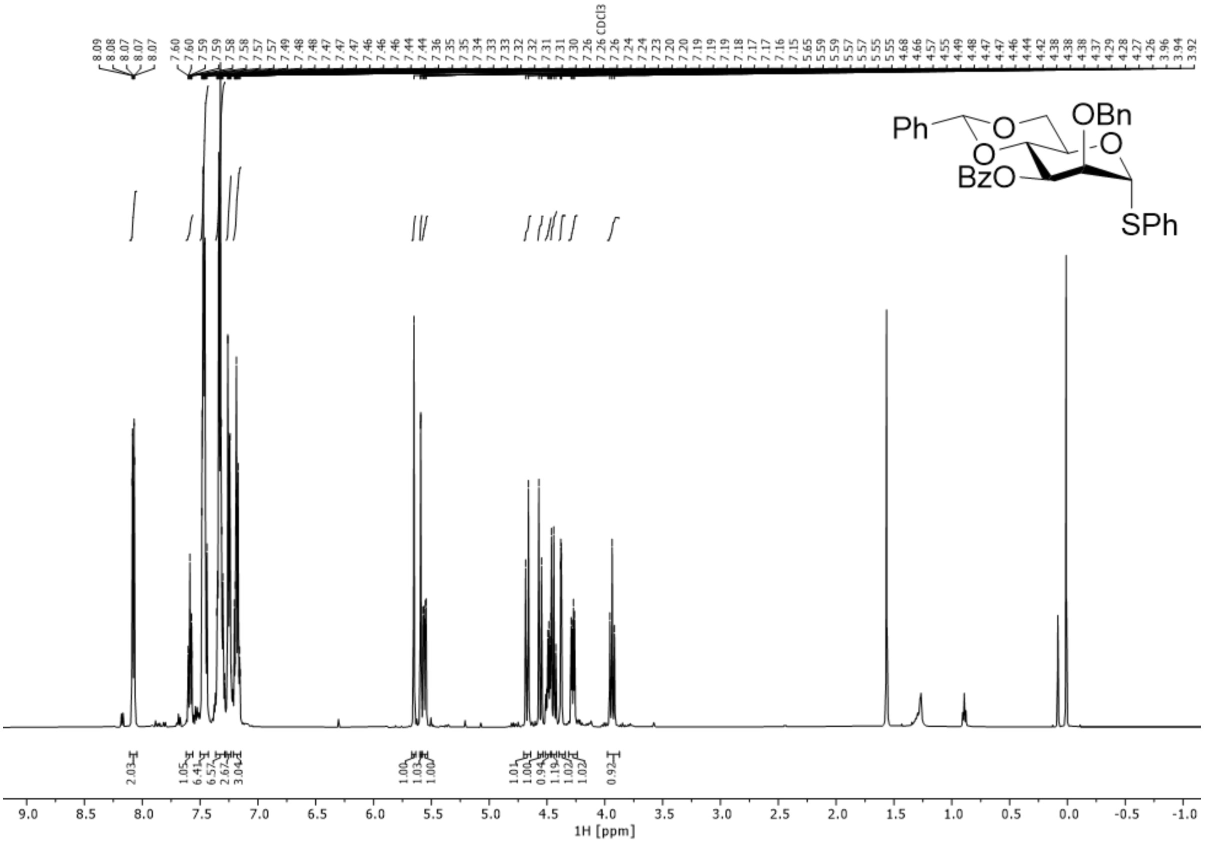

Figure S152: ^{1}H NMR of phenyl 3-O-benzoyl-2-O-benzyl-4,6-O-benzylidene-1-thio-α-D-mannopyranoside.

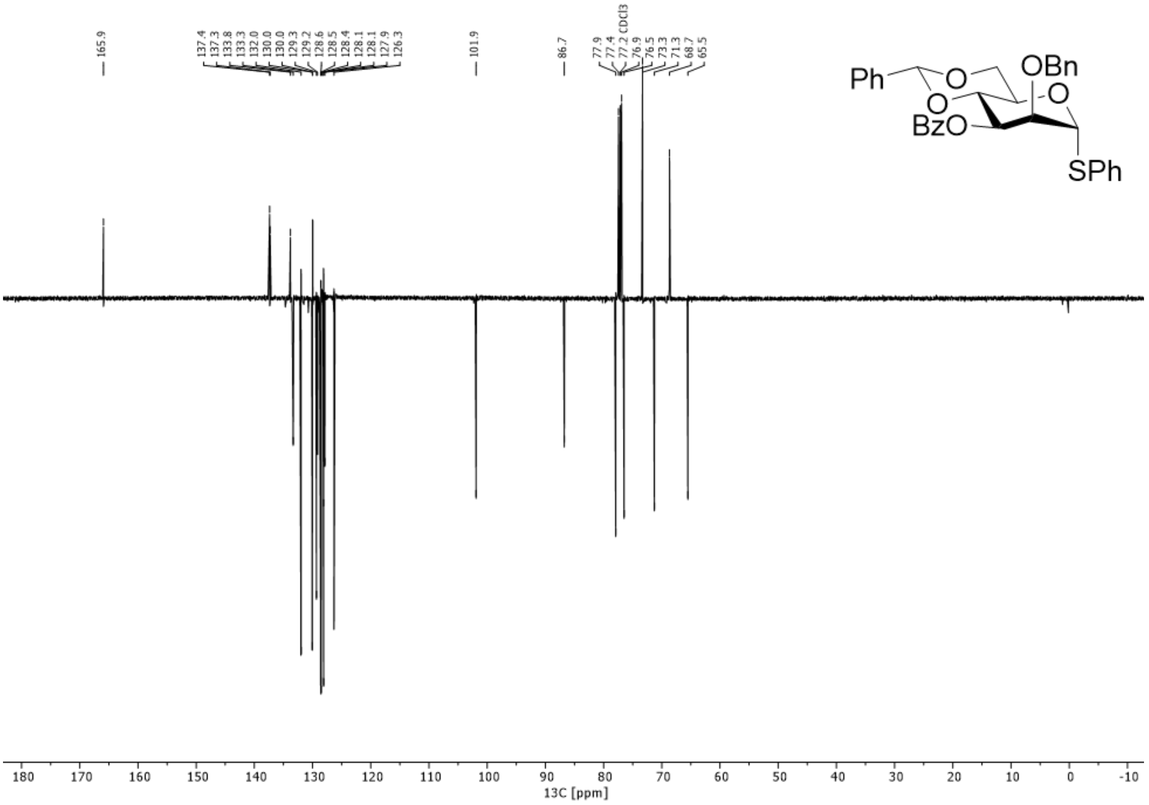

Figure S153: ^{13}C NMR of phenyl 3-O-benzoyl-2-O-benzyl-4,6-O-benzylidene-1-thio-α-D-mannopyranoside.

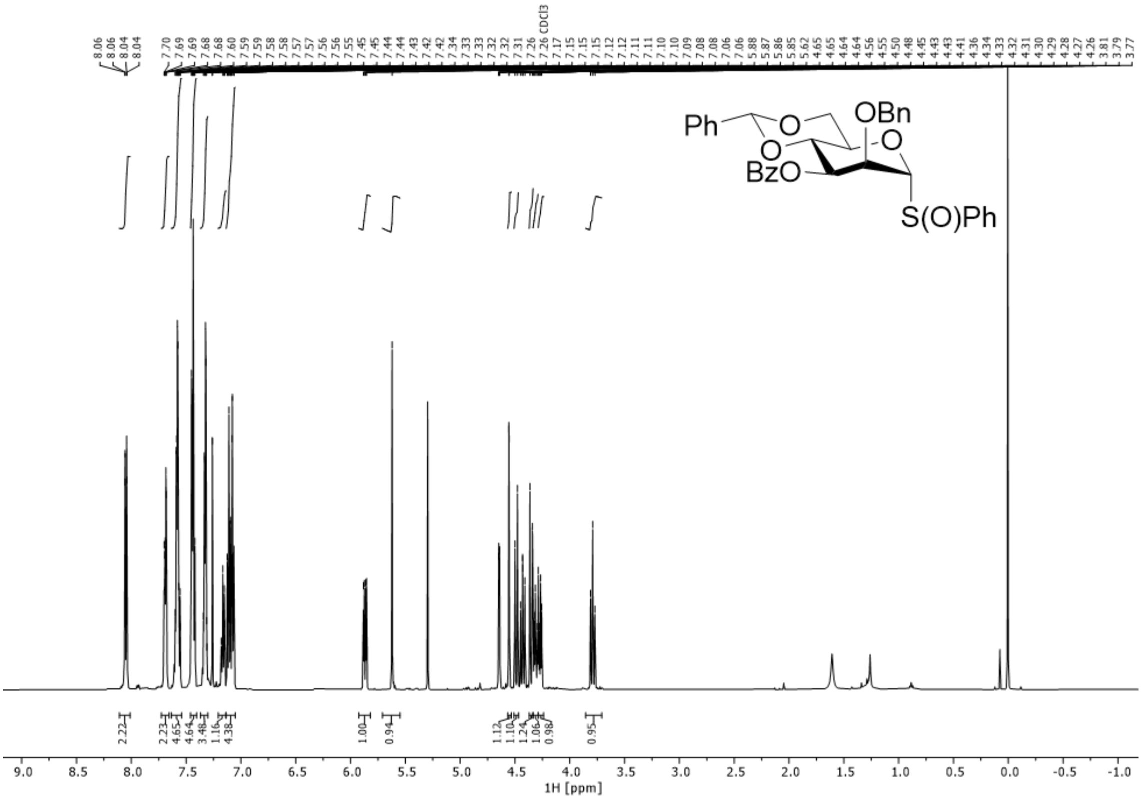

Figure S154: ^{1}H NMR of phenyl 3-O-benzoyl-2-O-benzyl-4,6-O-benzylidene-1-thiosulfinyl-α-D-mannopyranoside.

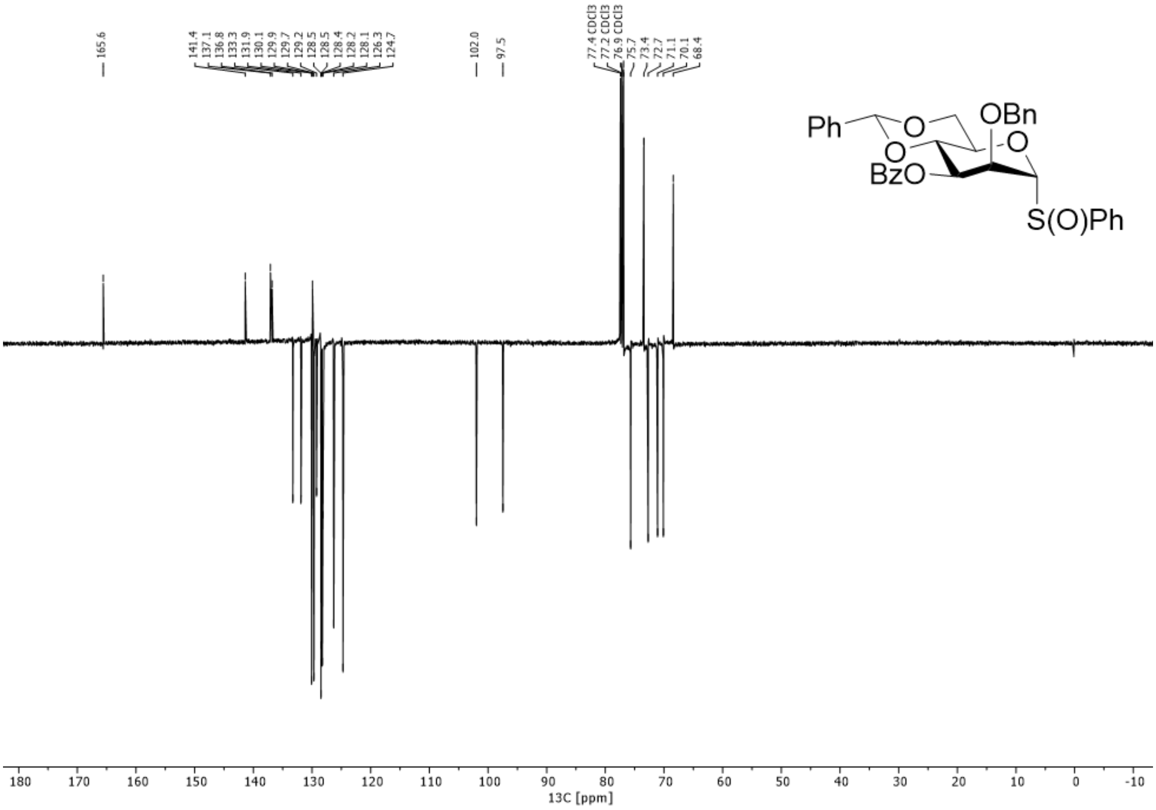

Figure S155: ^{13}C NMR of phenyl 3-O-benzoyl-2-O-benzyl-4,6-O-benzylidene-1-thiosulfinyl-α-D-mannopyranoside.

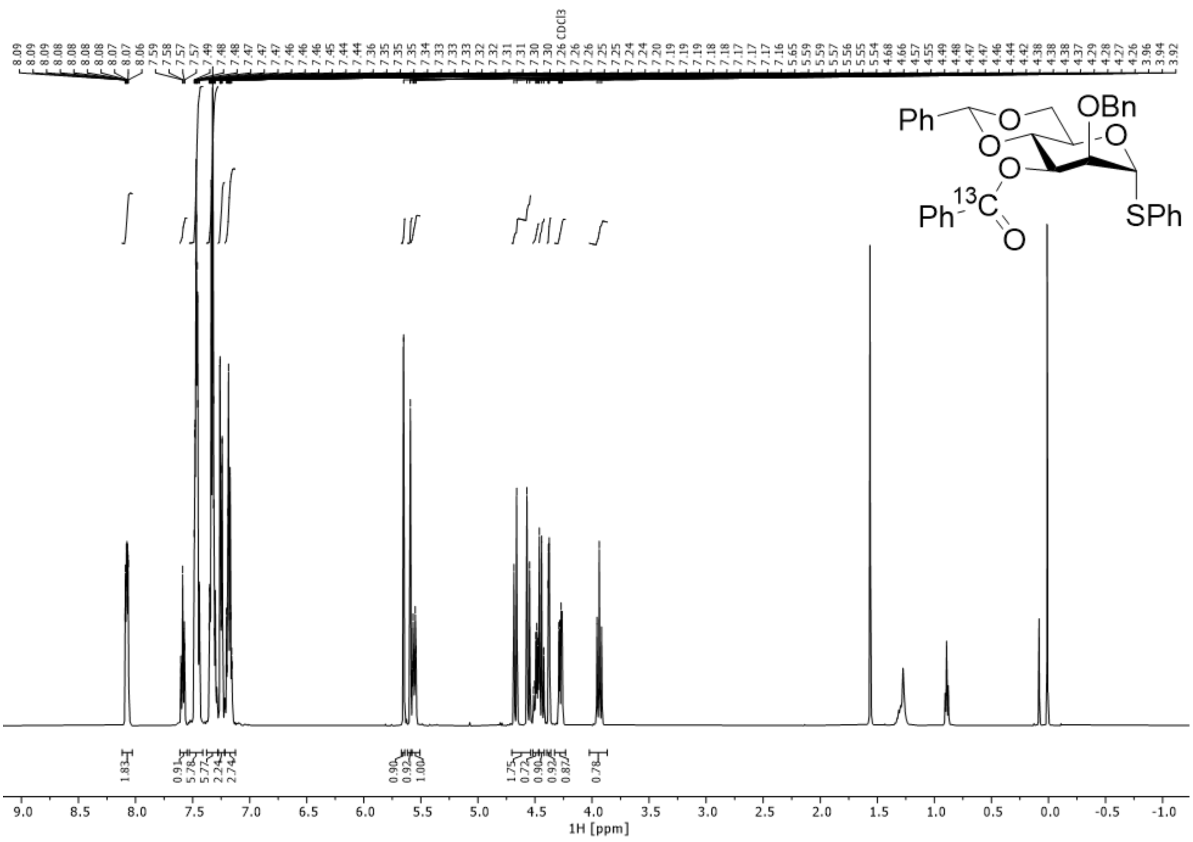

Figure S156: ^{1}H NMR of phenyl 3-*O*-(benzoyl-α- ^{13}C)-2-*O*-benzyl-4,6-*O*-benzylidene-1-thio-α-D-mannopyranoside.

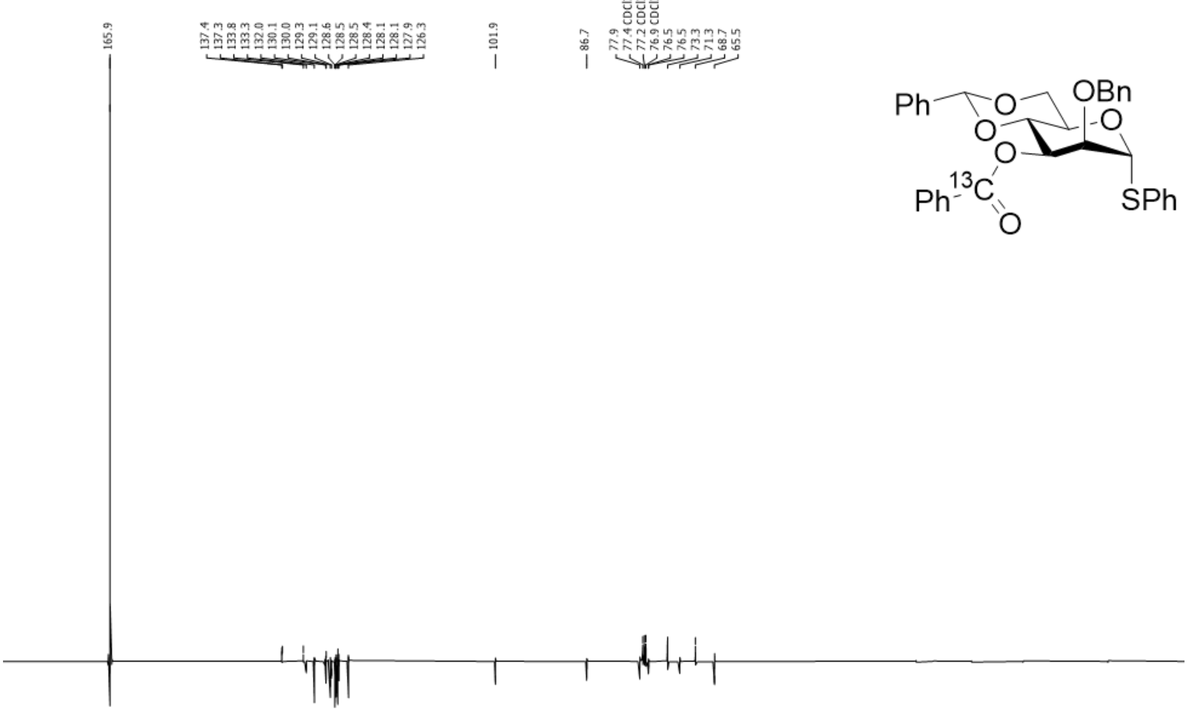

Figure S157: ^{13}C NMR of phenyl 3-*O*-(benzoyl-α- ^{13}C)-2-*O*-benzyl-4,6-*O*-benzylidene-1-thio-α-D-mannopyranoside.

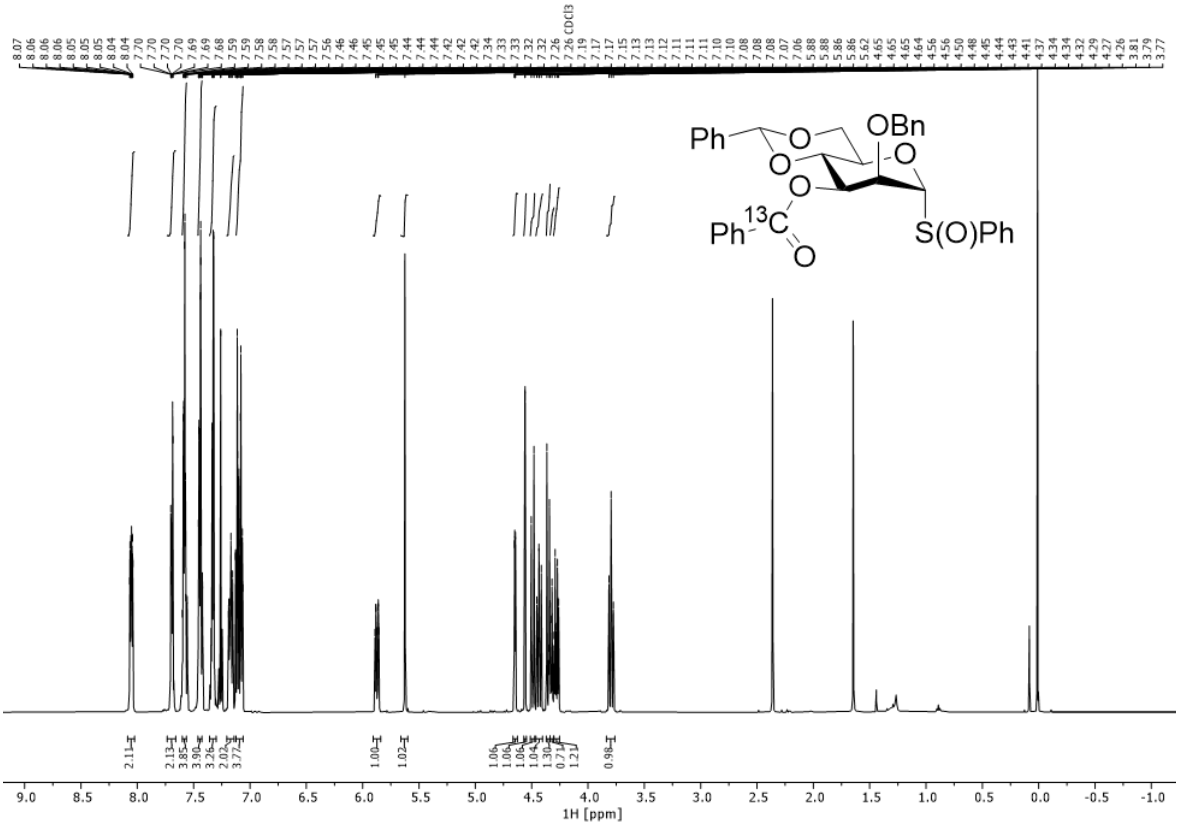

Figure S158: ^{1}H NMR of phenyl 3-*O*-(benzoyl-α-^{13}C)-2-*O*-benzyl-4,6-*O*-benzylidene-1-thiosulfinyl-α-D-mannopyranoside.

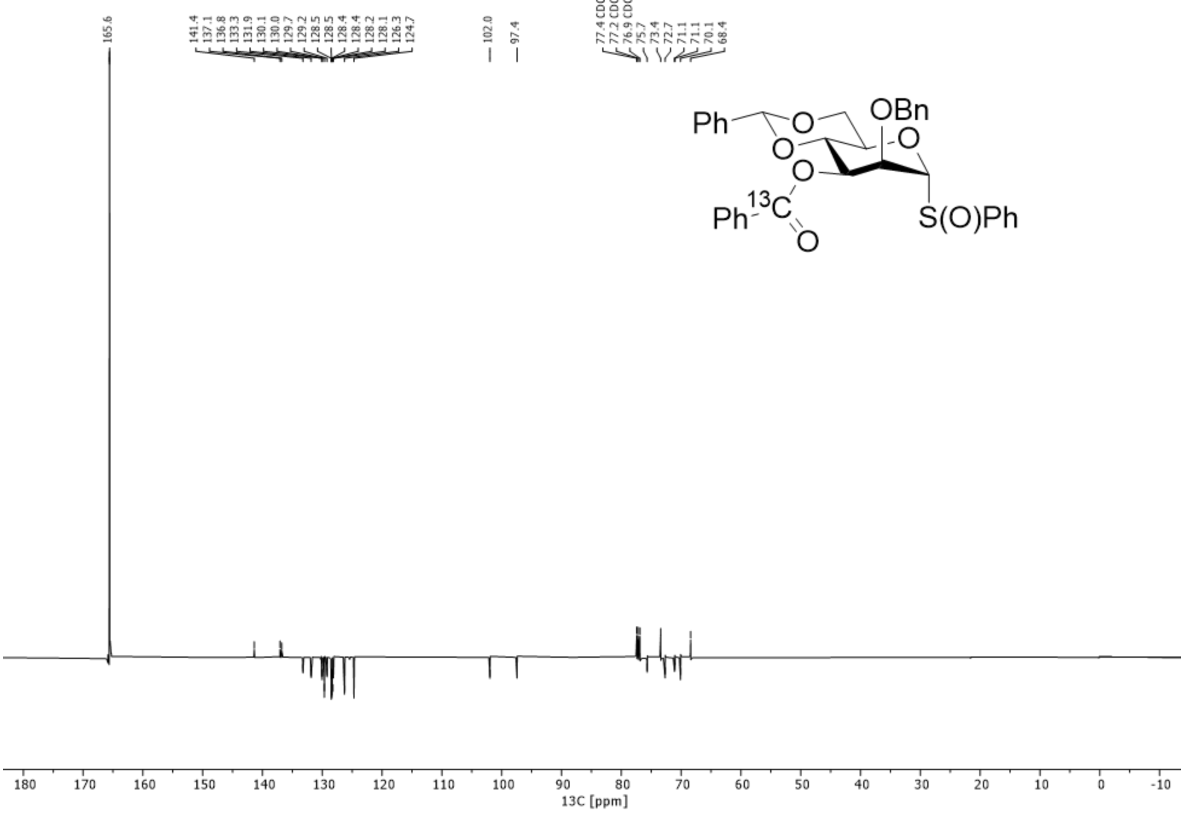

Figure S159: ^{13}C NMR of phenyl 3-*O*-(benzoyl-α-^{13}C)-2-*O*-benzyl-4,6-*O*-benzylidene-1-thiosulfinyl-α-D-mannopyranoside.

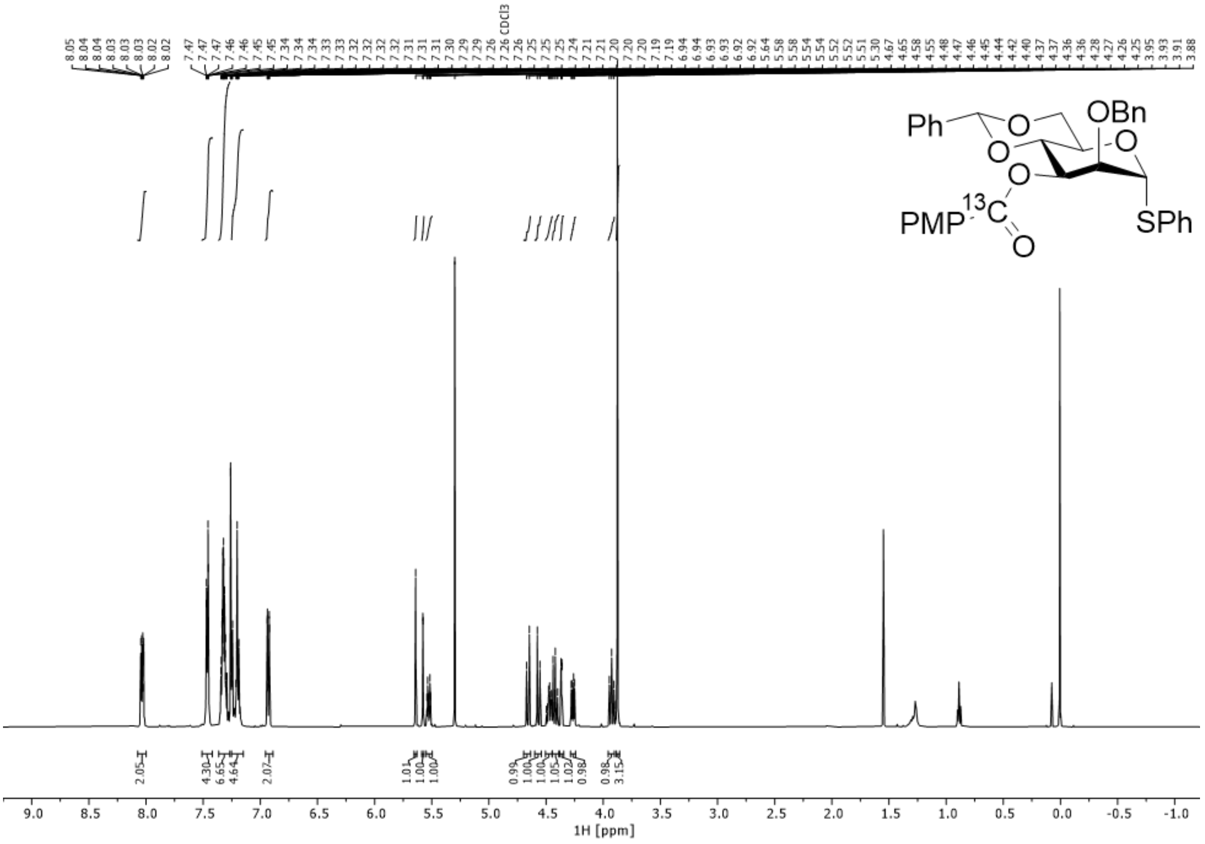

Figure S160: ^{1}H NMR of phenyl 3-*O*-(4-methoxybenzoyl -α- ^{13}C)-2-*O*-benzyl-4,6-*O*-benzylidene-1-thiosulfinyl-α-D-mannopyranoside.

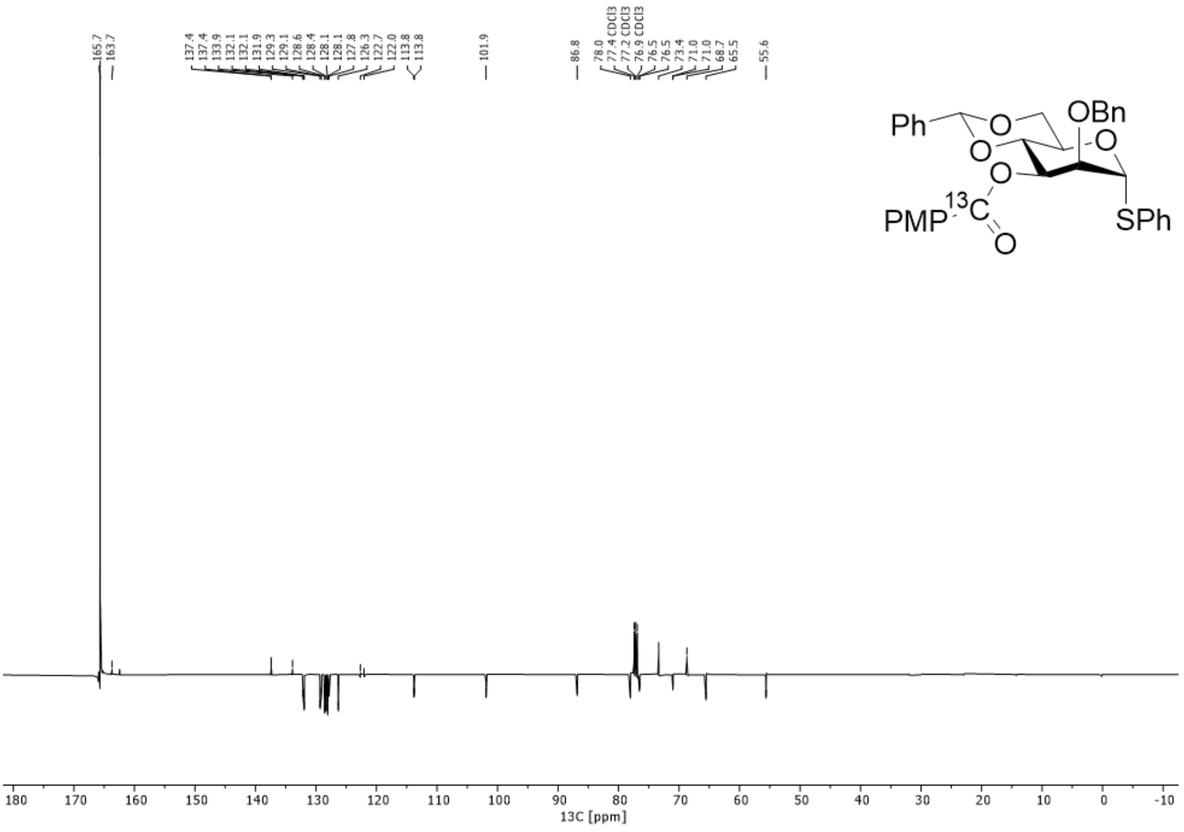

Figure S161: ^{13}C NMR of phenyl 3-*O*-(4-methoxybenzoyl -α- ^{13}C)-2-*O*-benzyl-4,6-*O*-benzylidene-1-thiosulfinyl-α-D-mannopyranoside.

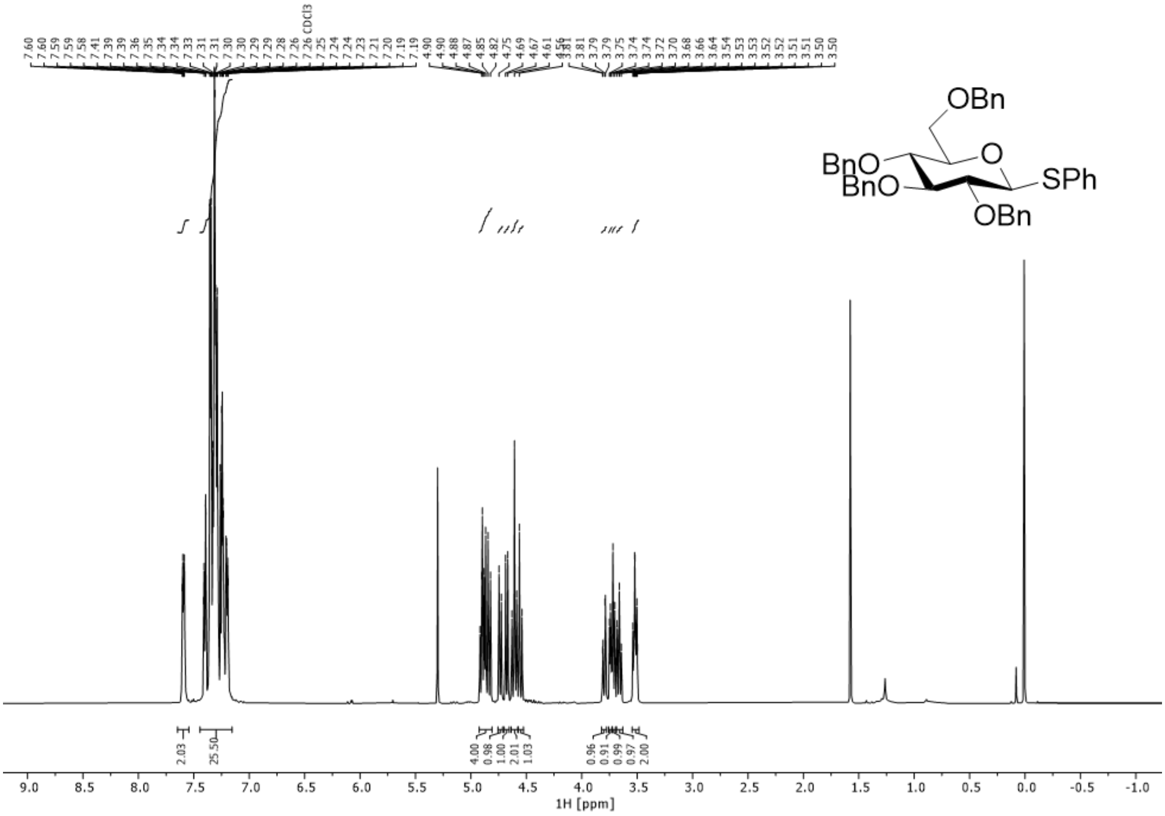

Figure S162: ^{1}H NMR of phenyl 2,3,4,6-tetra-*O*-benzyl-1-thio-β-D-glucopyranoside.

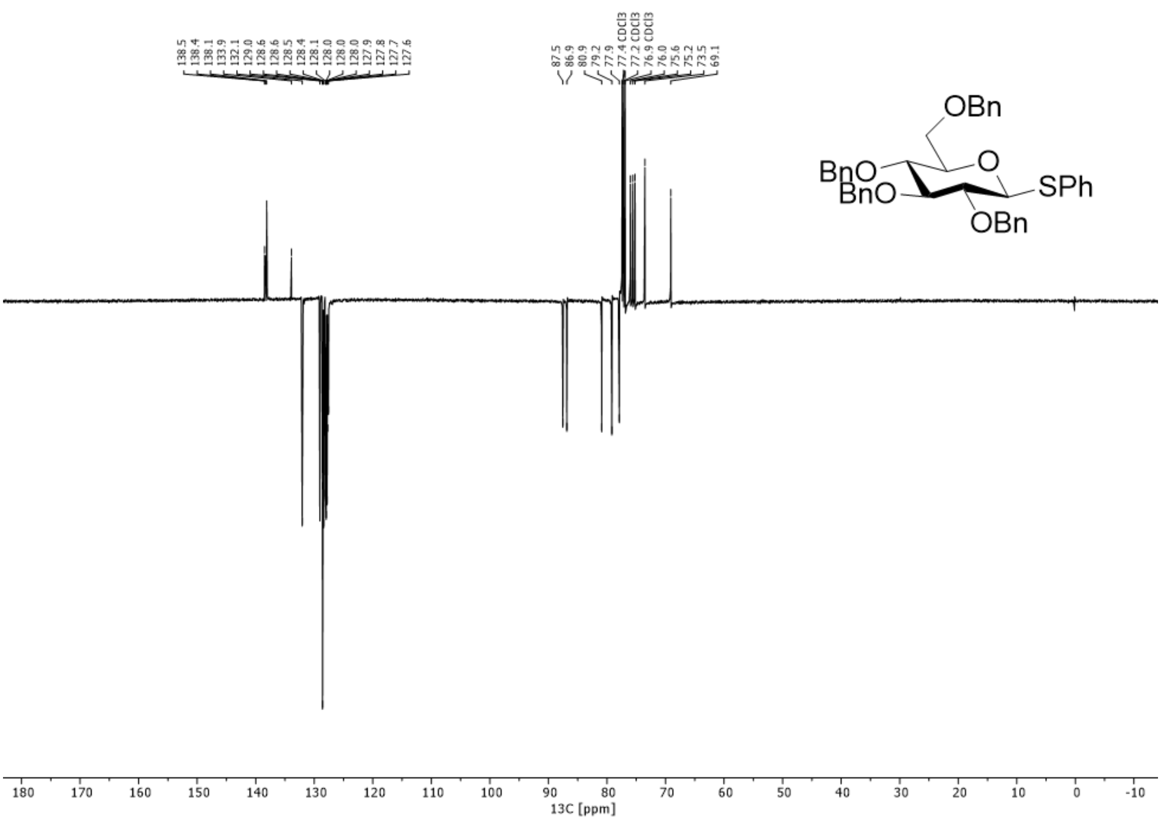

Figure S163: ^{13}C NMR of phenyl 2,3,4,6-tetra-*O*-benzyl-1-thio-β-D-glucopyranoside.

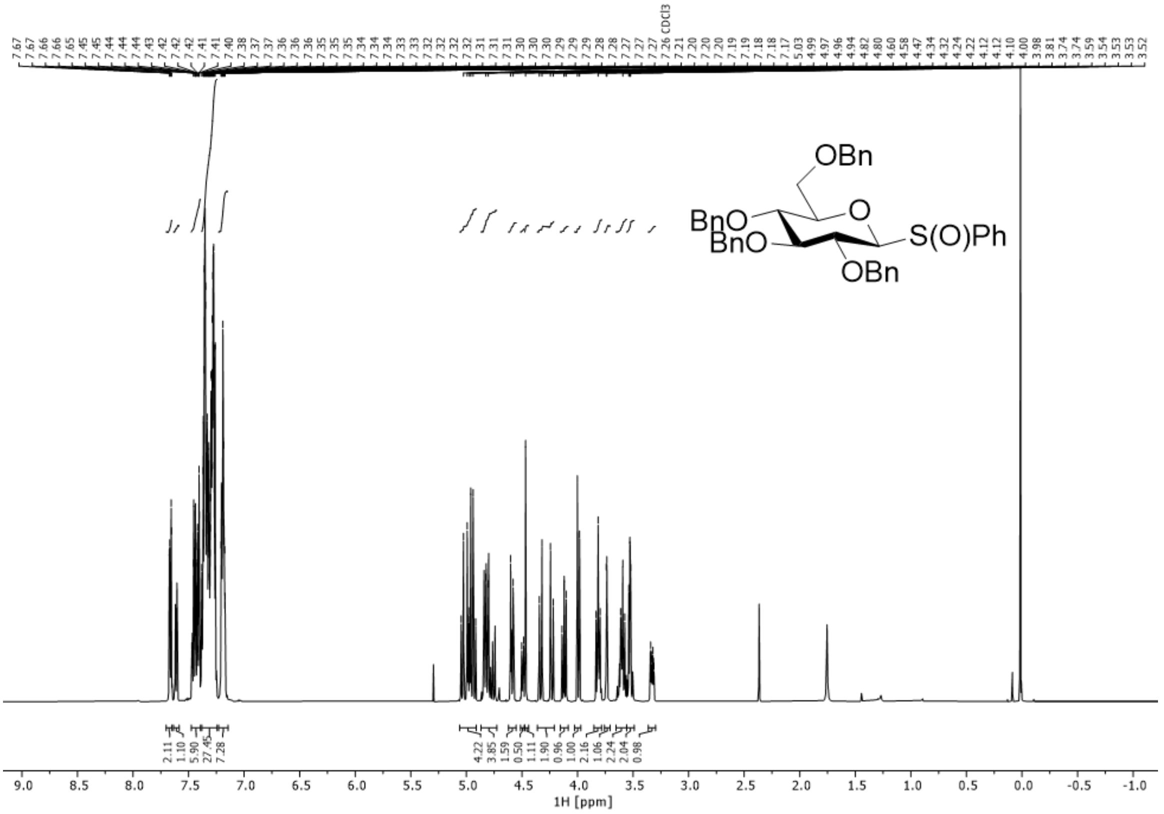

Figure S164: ^{1}H NMR of phenyl 2,3,4,6-tetra-*O*-benzyl-1-thiosulfinyl-β-D-glucopyranoside.

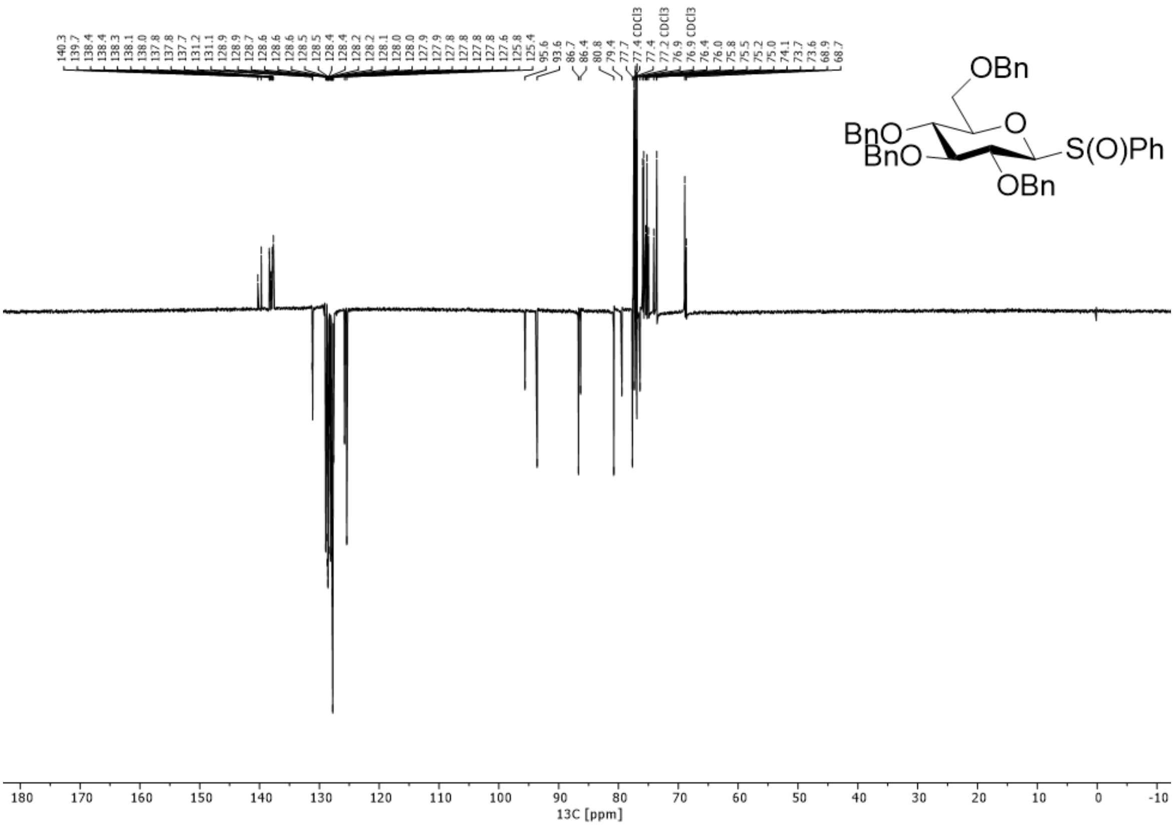

Figure S165: ^{13}C NMR of phenyl 2,3,4,6-tetra-*O*-benzyl-1-thiosulfinyl-β-D-glucopyranoside.

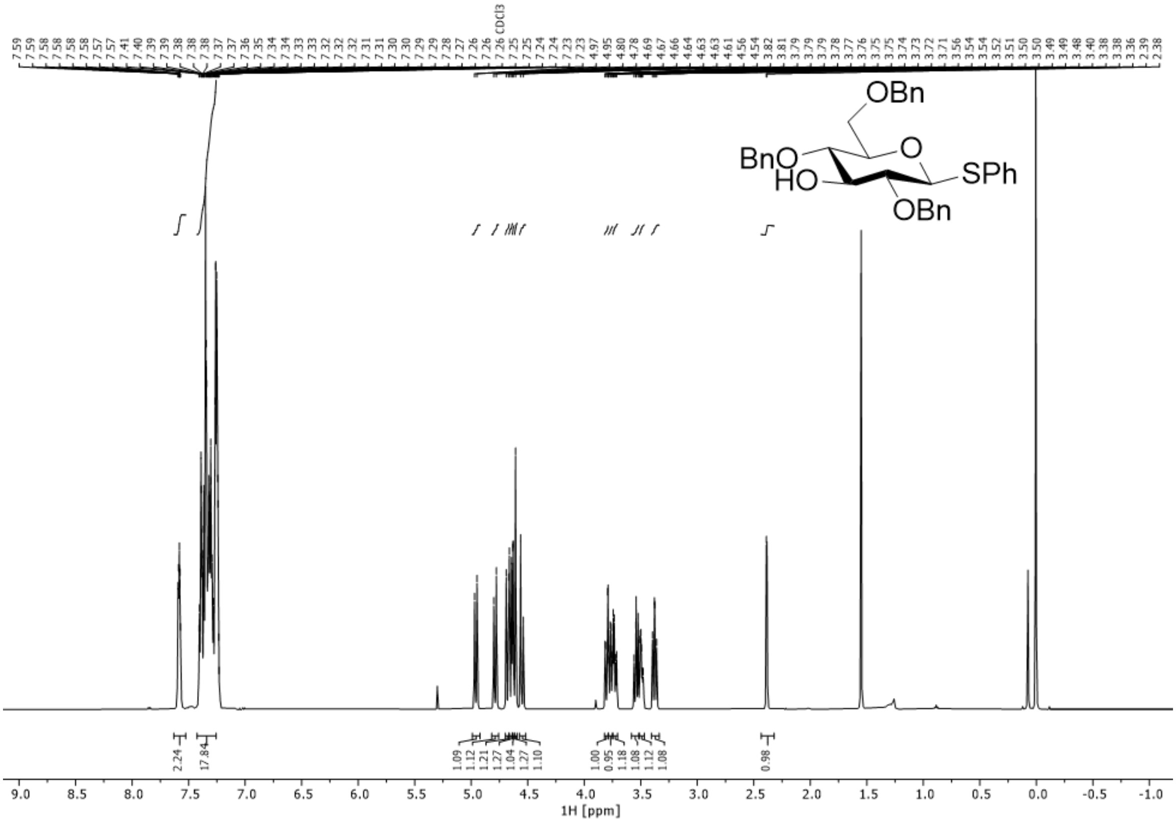

Figure S166: ^{1}H NMR of phenyl 2,4,6-tri-*O*-benzyl-1-thio- β-D-glucopyranoside.

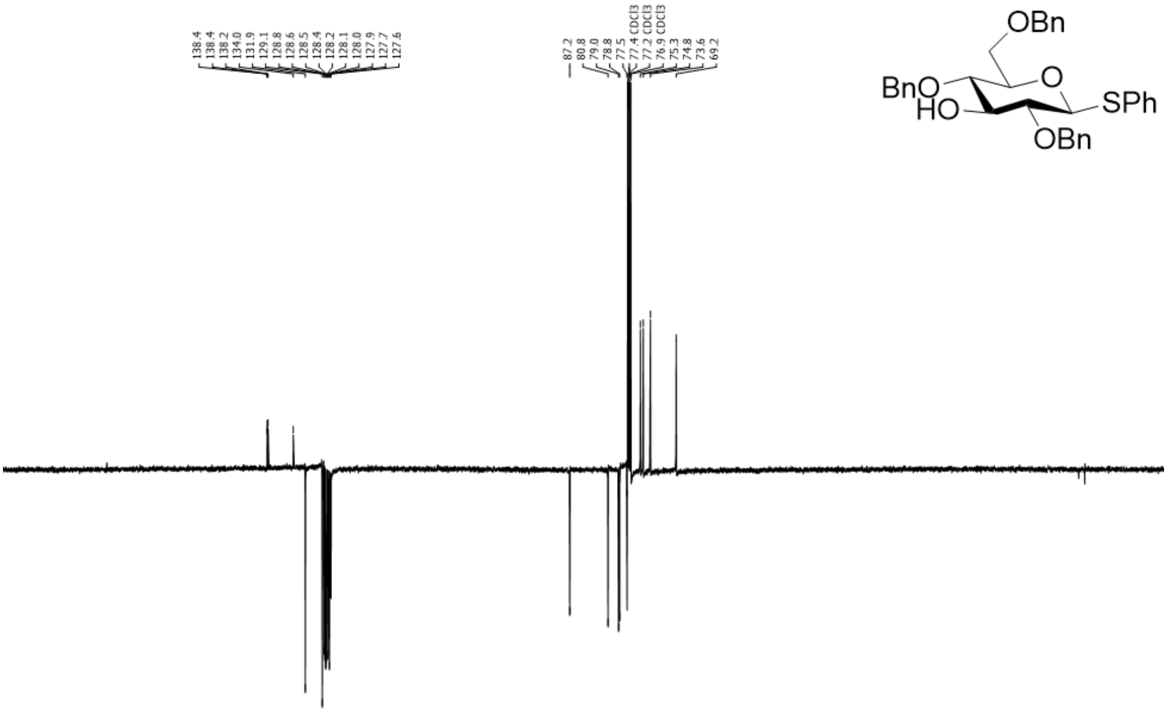

Figure S167: ^{13}C NMR of phenyl 2,4,6-tri-*O*-benzyl-1-thio- β-D-glucopyranoside.

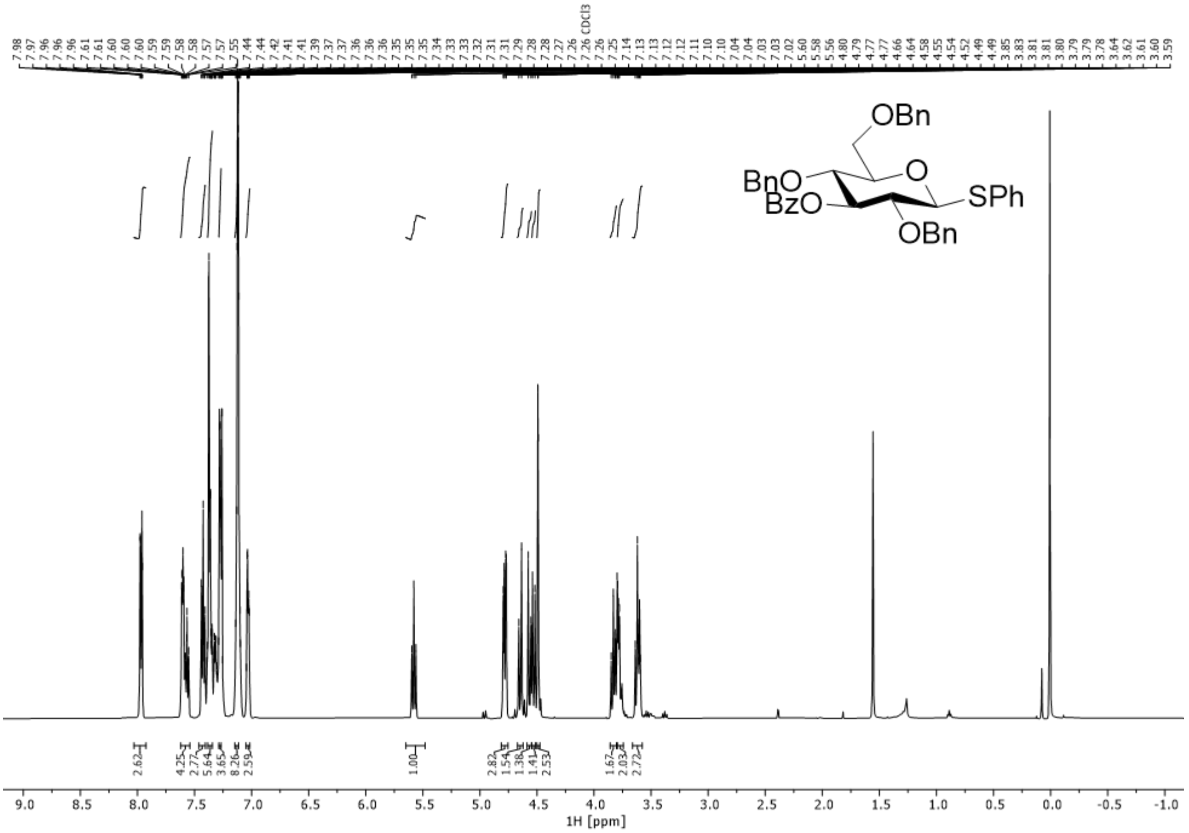

Figure S168: ^{1}H NMR of phenyl 3-*O*-benzoyl-2,4,6-tri-*O*-benzyl -1-thio- β-D-glucopyranoside.

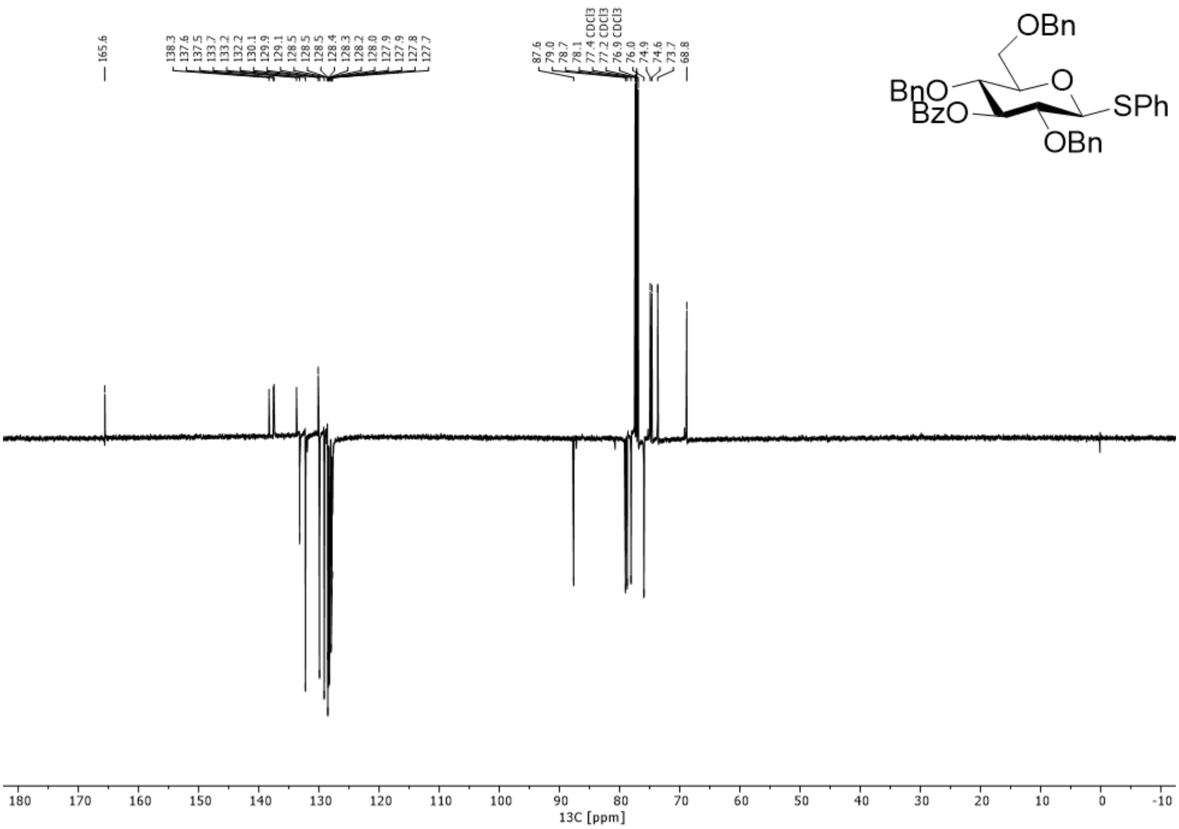

Figure S169: ^{13}C NMR of phenyl 3-*O*-benzoyl-2,4,6-tri-*O*-benzyl -1-thio- β-D-glucopyranoside.

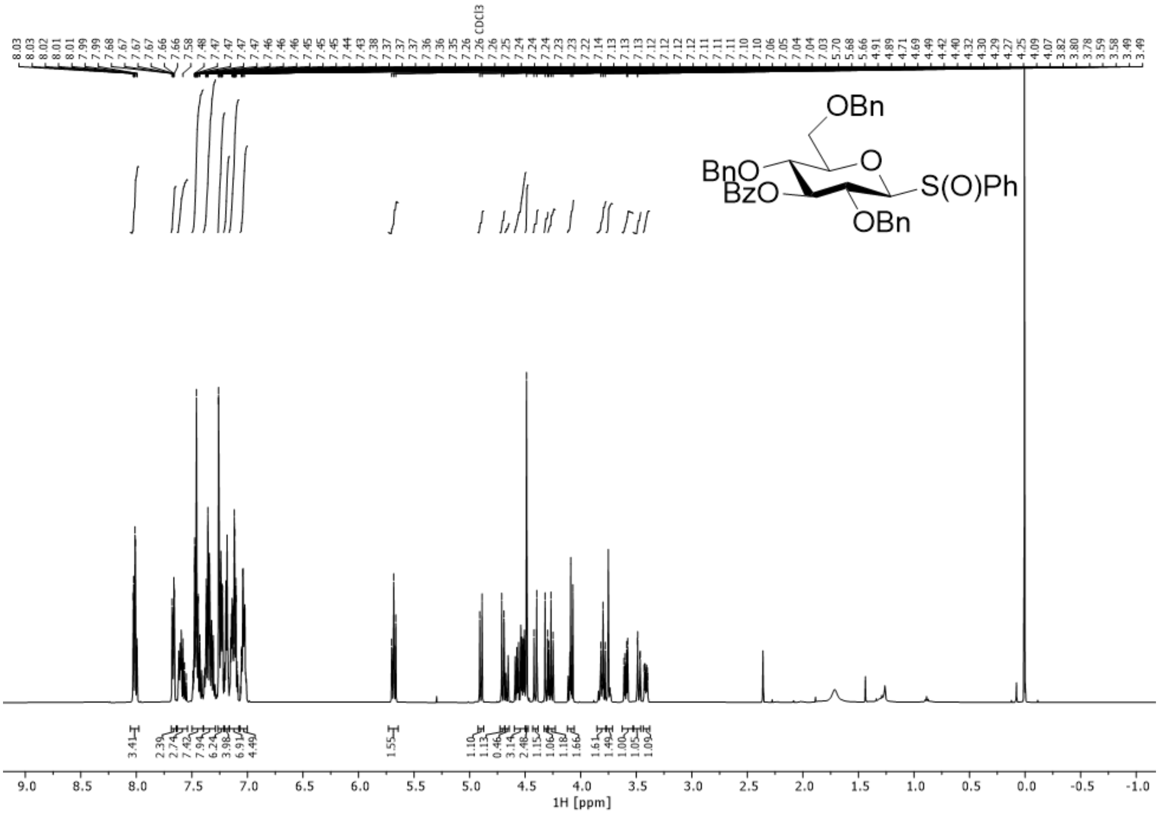

Figure S170: ^{1}H NMR of phenyl 3-*O*-benzoyl-2,4,6-tri-*O*-benzyl -1-thiosulfinyl- β-D-glucopyranoside.

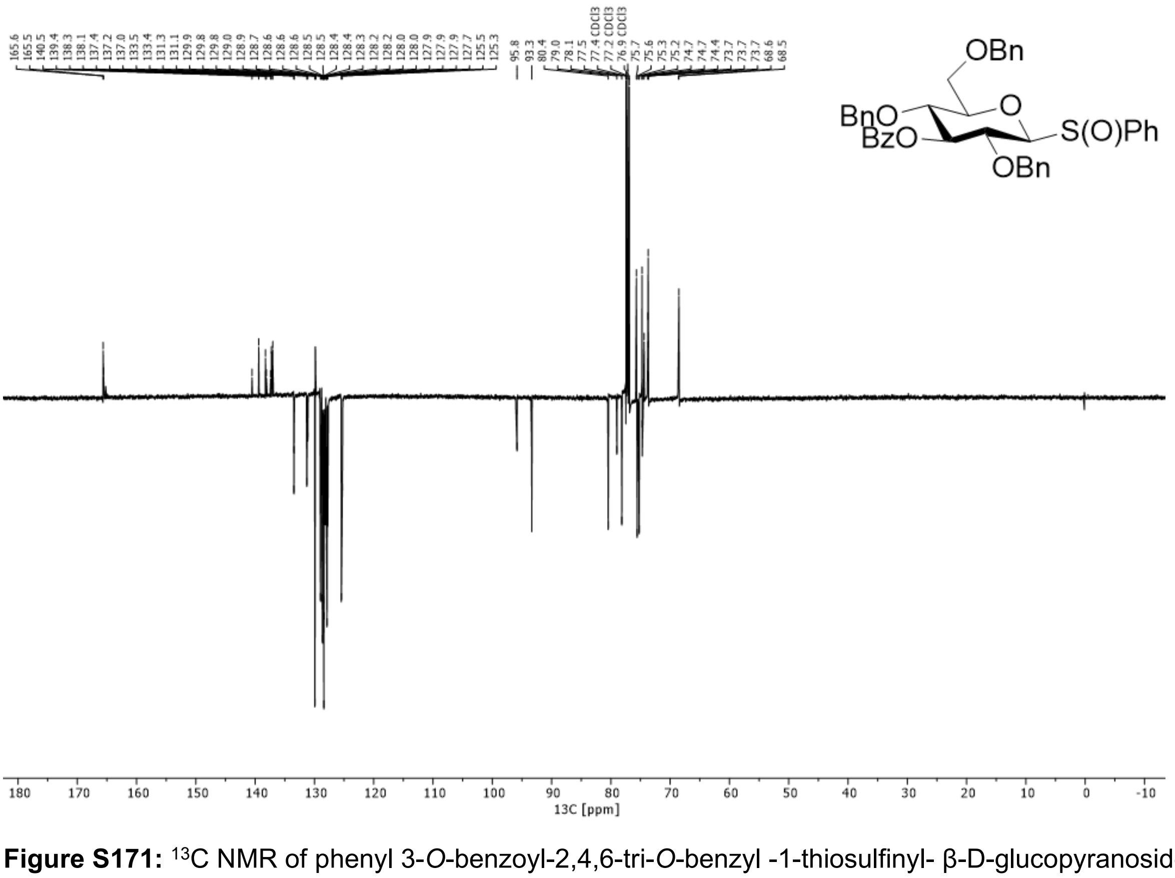

Figure S171: ^{13}C NMR of phenyl 3-*O*-benzoyl-2,4,6-tri-*O*-benzyl -1-thiosulfinyl- β-D-glucopyranoside.

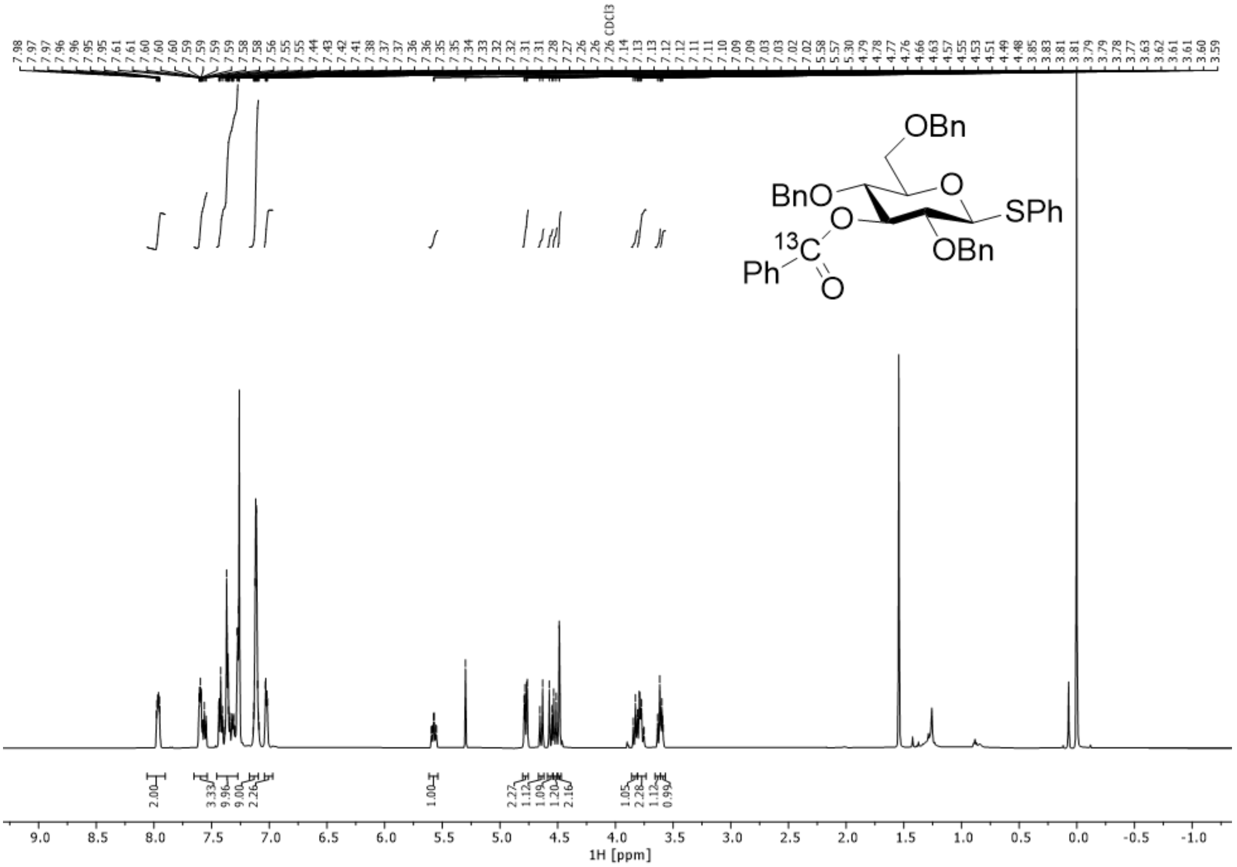

Figure S172: ^{1}H NMR of phenyl 3-*O*-(benzoyl-α- ^{13}C)-2,4,6-tri-*O*-benzyl -1-thio- β-D-glucopyranoside.

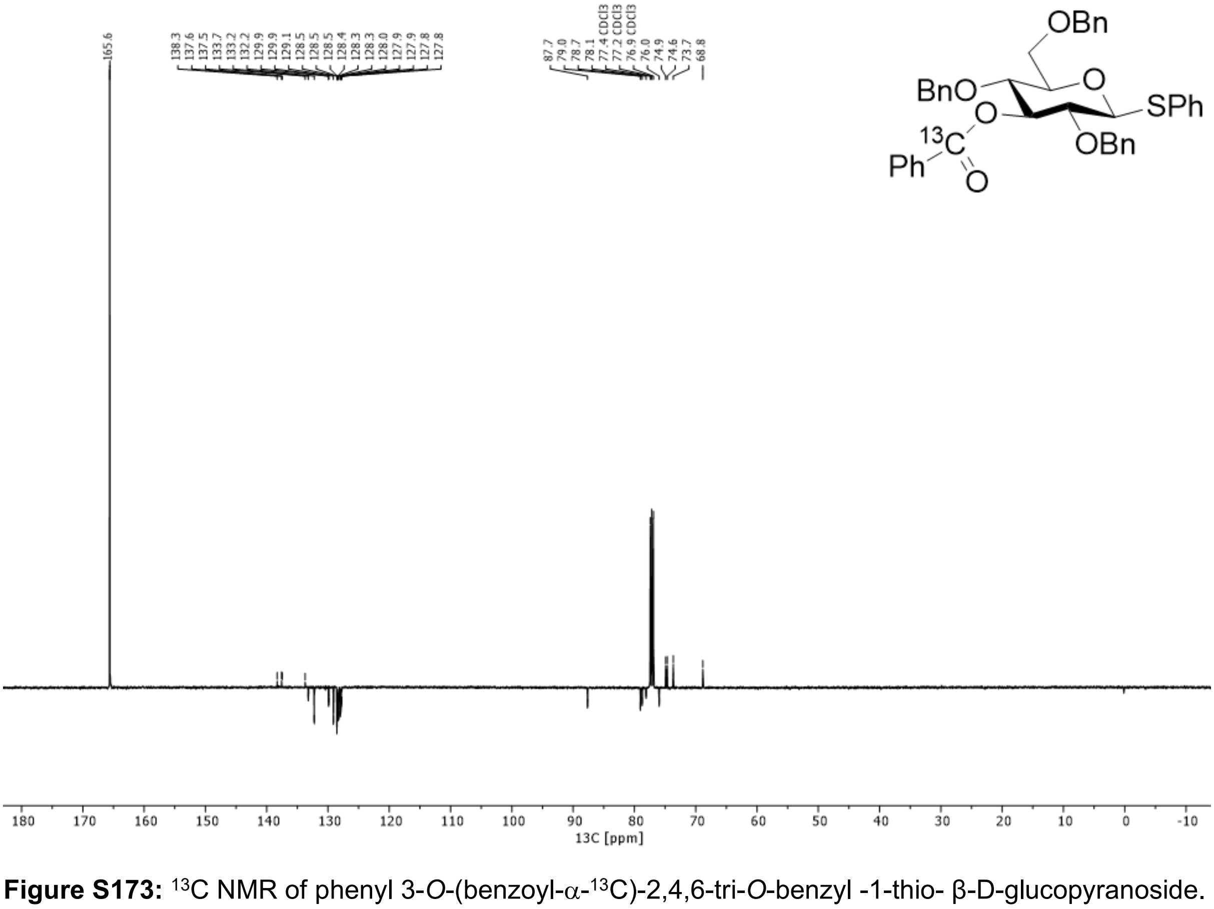

Figure S173: ^{13}C NMR of phenyl 3-*O*-(benzoyl-α-^{13}C)-2,4,6-tri-*O*-benzyl -1-thio- β-D-glucopyranoside.

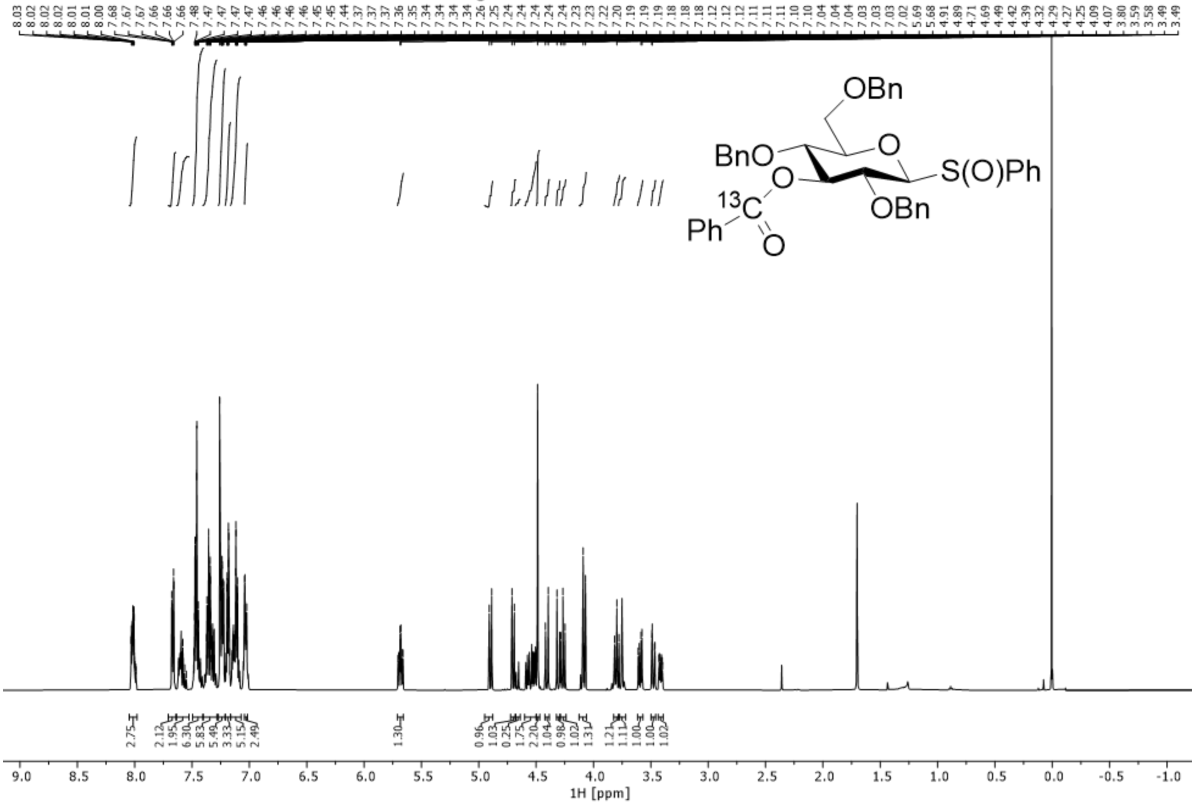

Figure S174: ^{1}H NMR of phenyl 3-*O*-(benzoyl-α- ^{13}C)-2,4,6-tri-*O*-benzyl -1-thiosulfinyl- β-D-glucopyranoside.

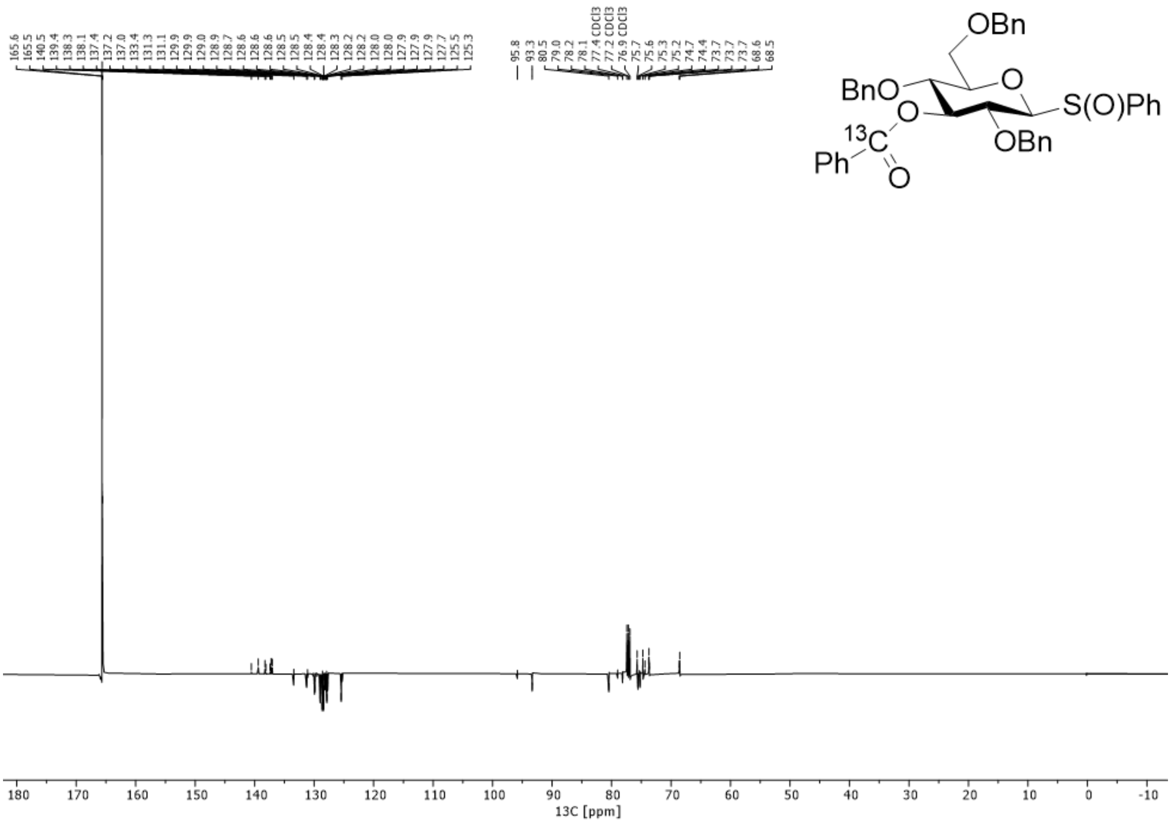

Figure S175: ^{13}C NMR of phenyl 3-*O*-(benzoyl-α- ^{13}C)-2,4,6-tri-*O*-benzyl -1-thiosulfinyl- β-D-glucopyranoside.

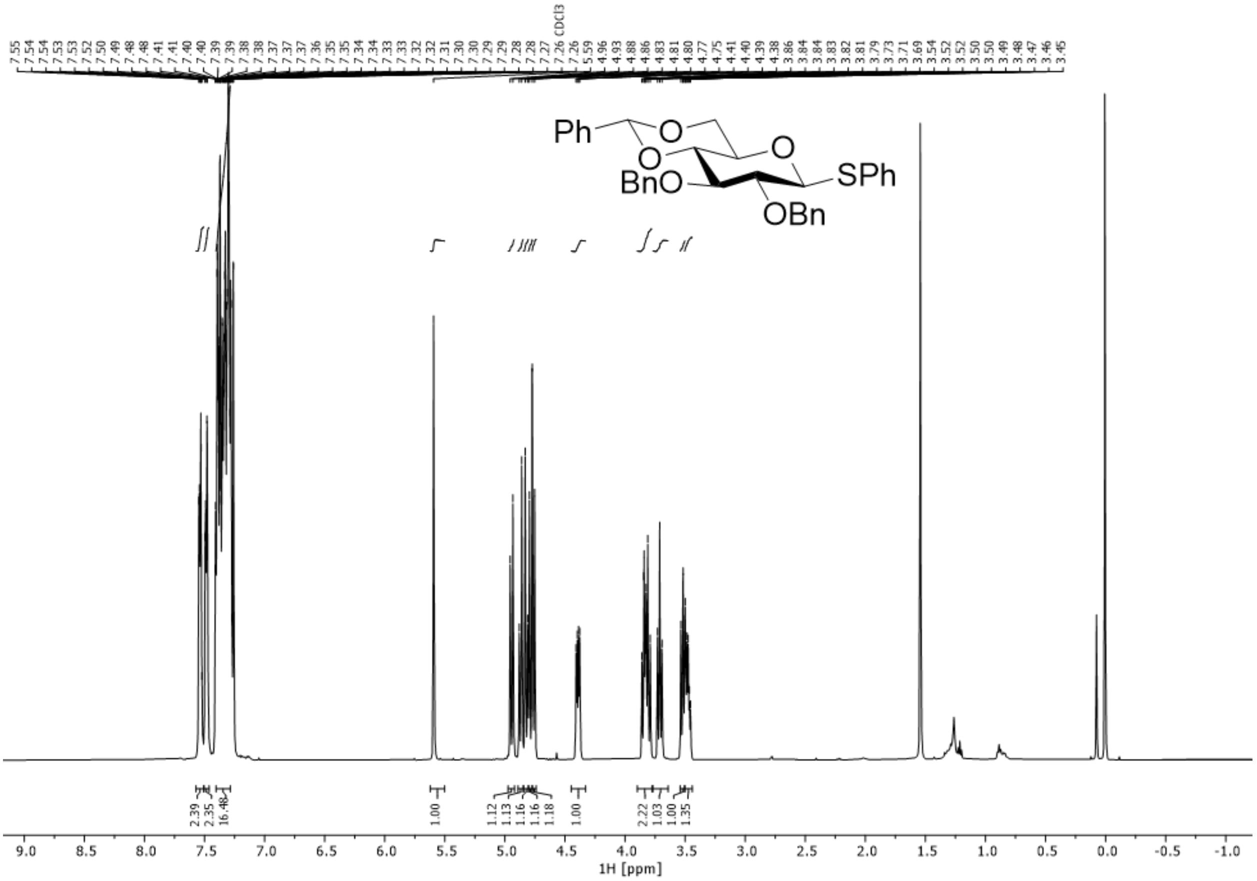

Figure S176: ^{1}H NMR of phenyl 2,3-di-*O*-benzyl-4,6-*O*-benzylidene-1-thio- β-D-glucopyranoside.

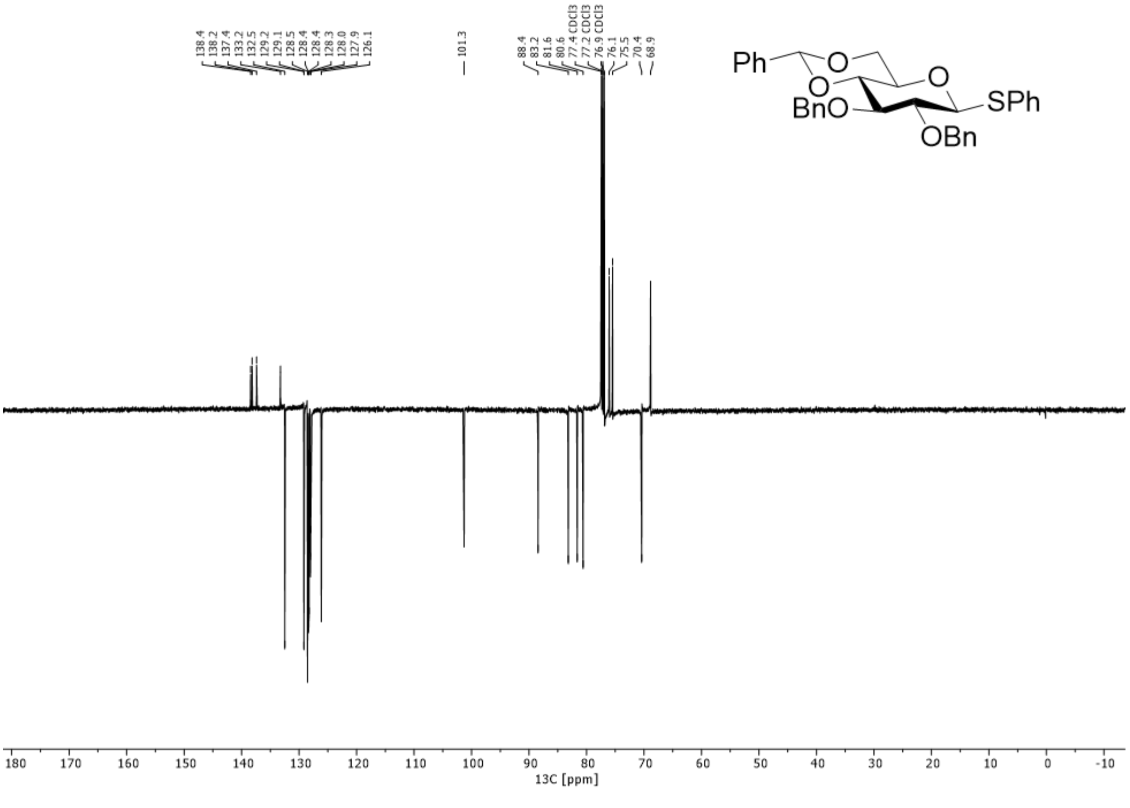

Figure S177: ^{13}C NMR of phenyl 2,3-di-*O*-benzyl-4,6-*O*-benzylidene-1-thio- β-D-glucopyranoside.

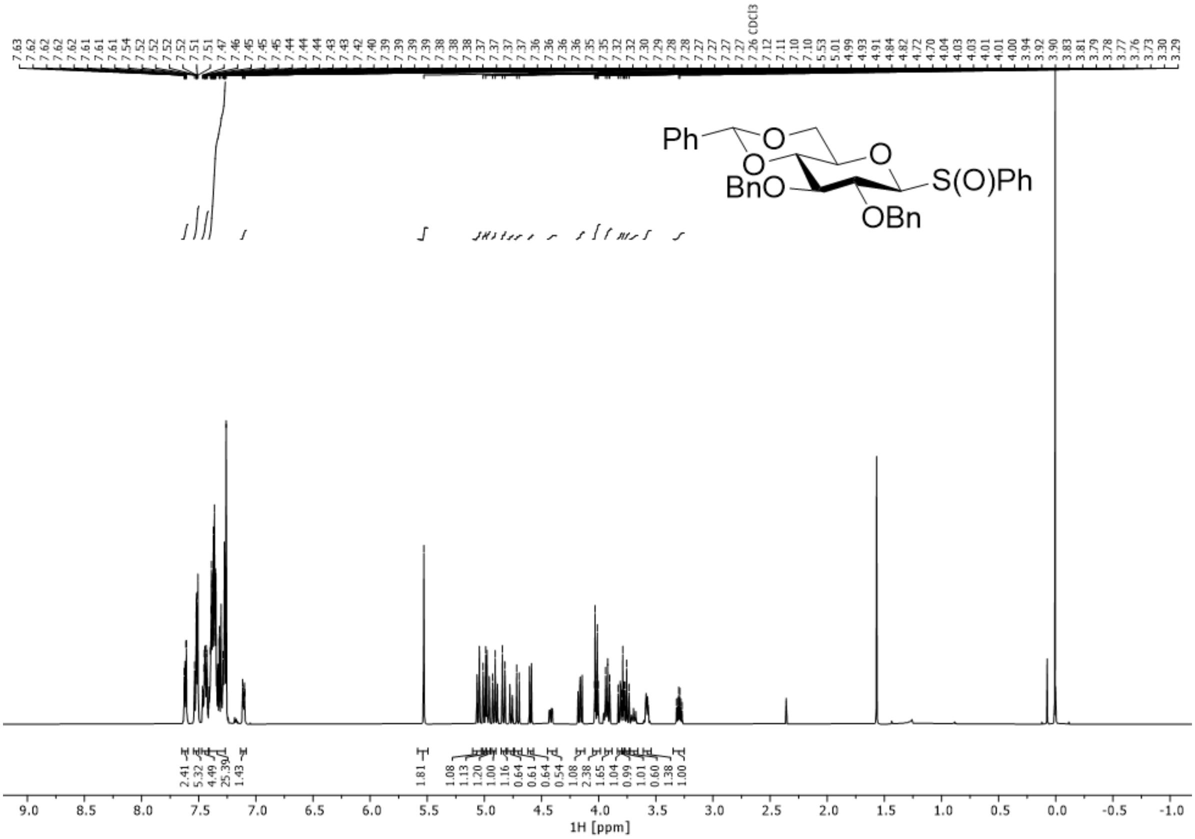

Figure S178: ^{1}H NMR of phenyl 2,3-di-*O*-benzyl-4,6-*O*-benzylidene-1-thiosulfinyl- β-D-glucopyranoside.

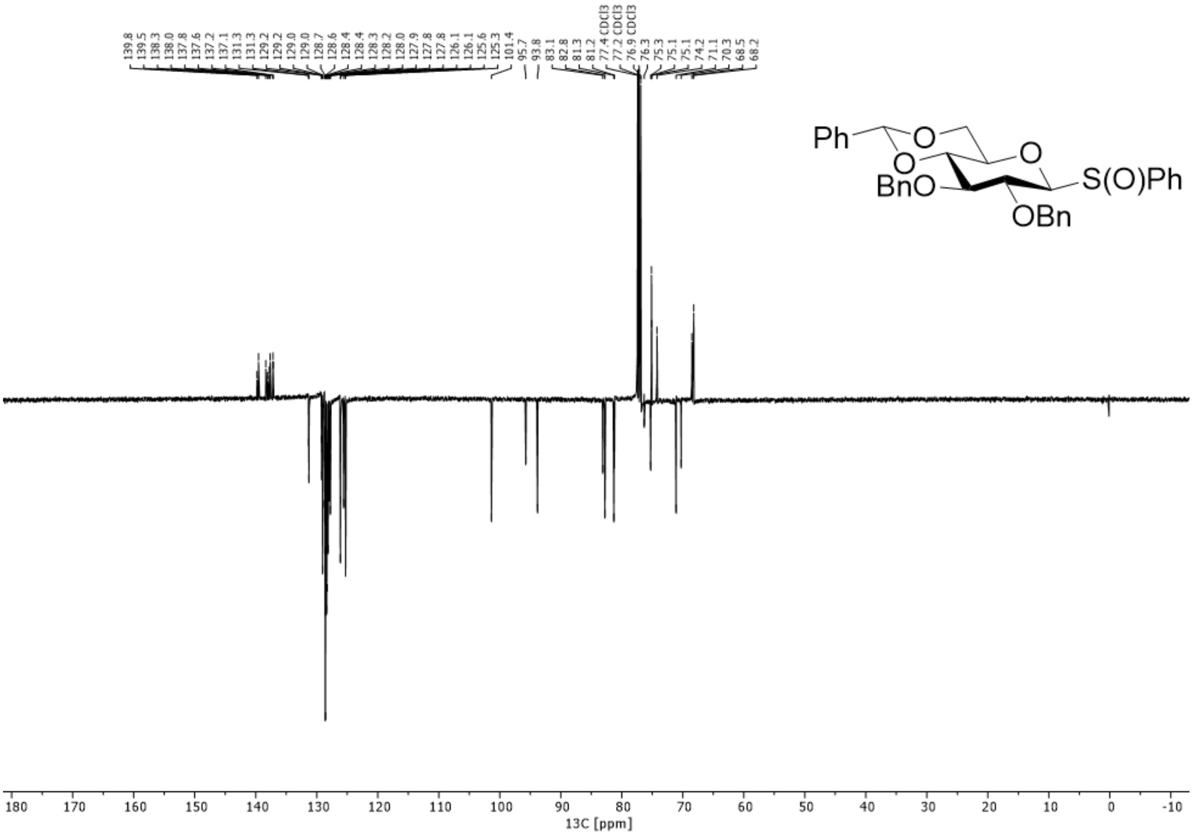

Figure S179: ^{13}C NMR of phenyl 2,3-di-*O*-benzyl-4,6-*O*-benzylidene-1-thiosulfinyl- β-D-glucopyranoside.

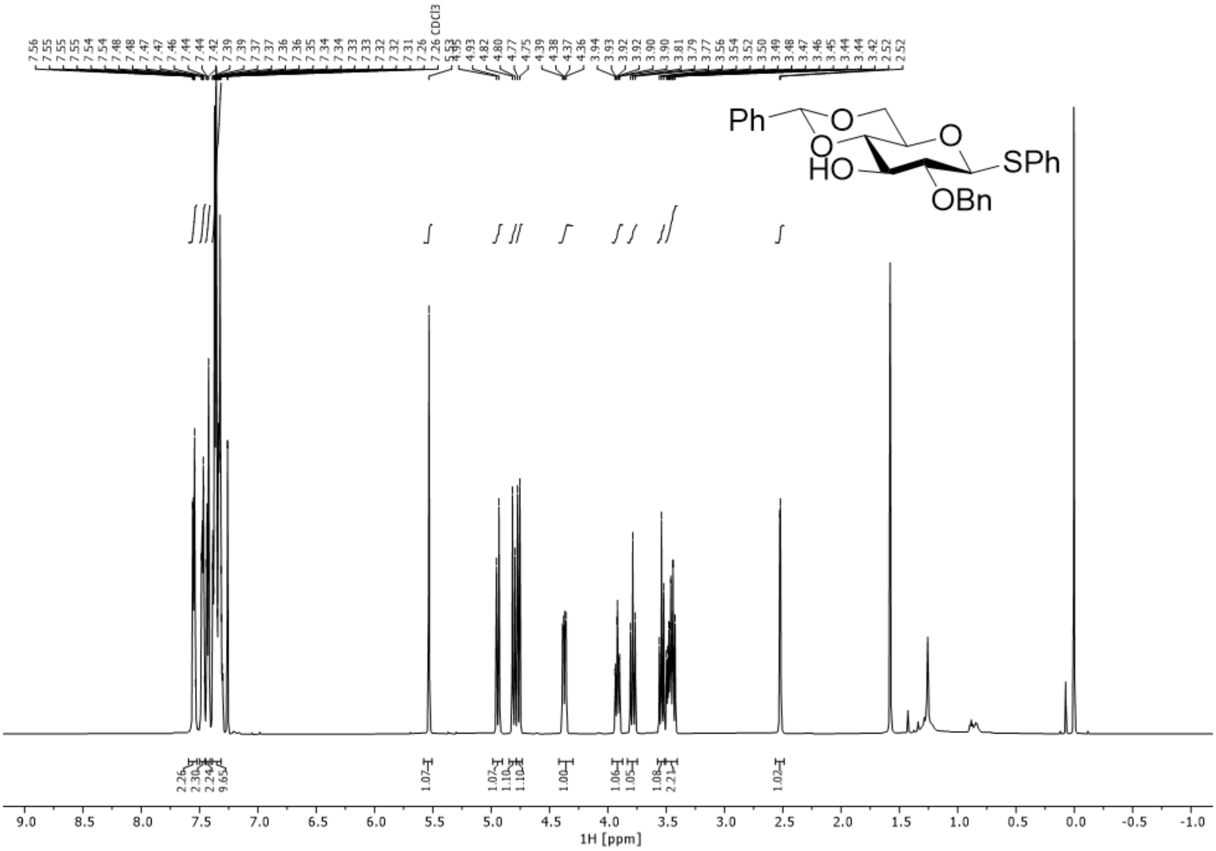

Figure S180: ^{1}H NMR of phenyl 2-*O*-benzyl-4,6-*O*-benzylidene-1-thio- β-D-glucopyranoside.

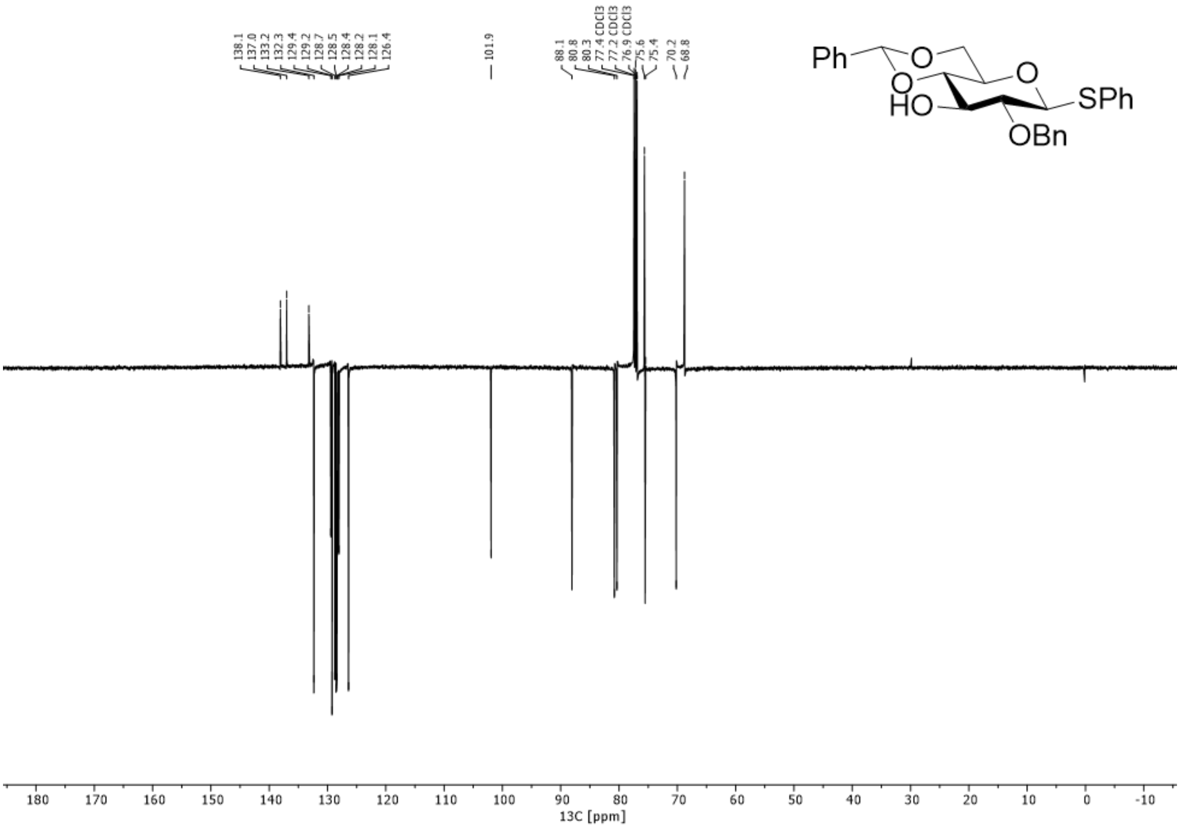

Figure S181: ^{13}C NMR of phenyl 2-*O*-benzyl-4,6-*O*-benzylidene-1-thio- β-D-glucopyranoside.

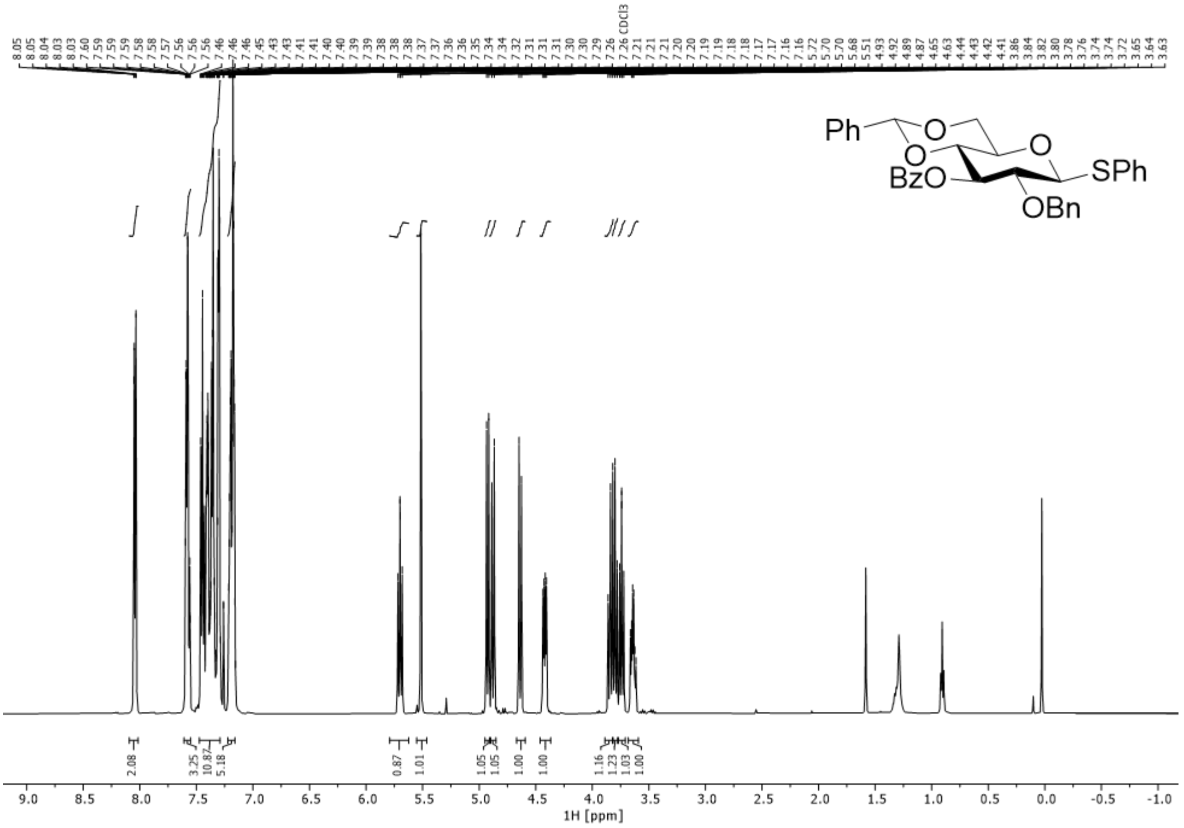

Figure S182: ^{1}H NMR of phenyl 3-*O*-benzoyl-2-*O*-benzyl-4,6-*O*-benzylidene-1-thio- β-D-glucopyranoside.

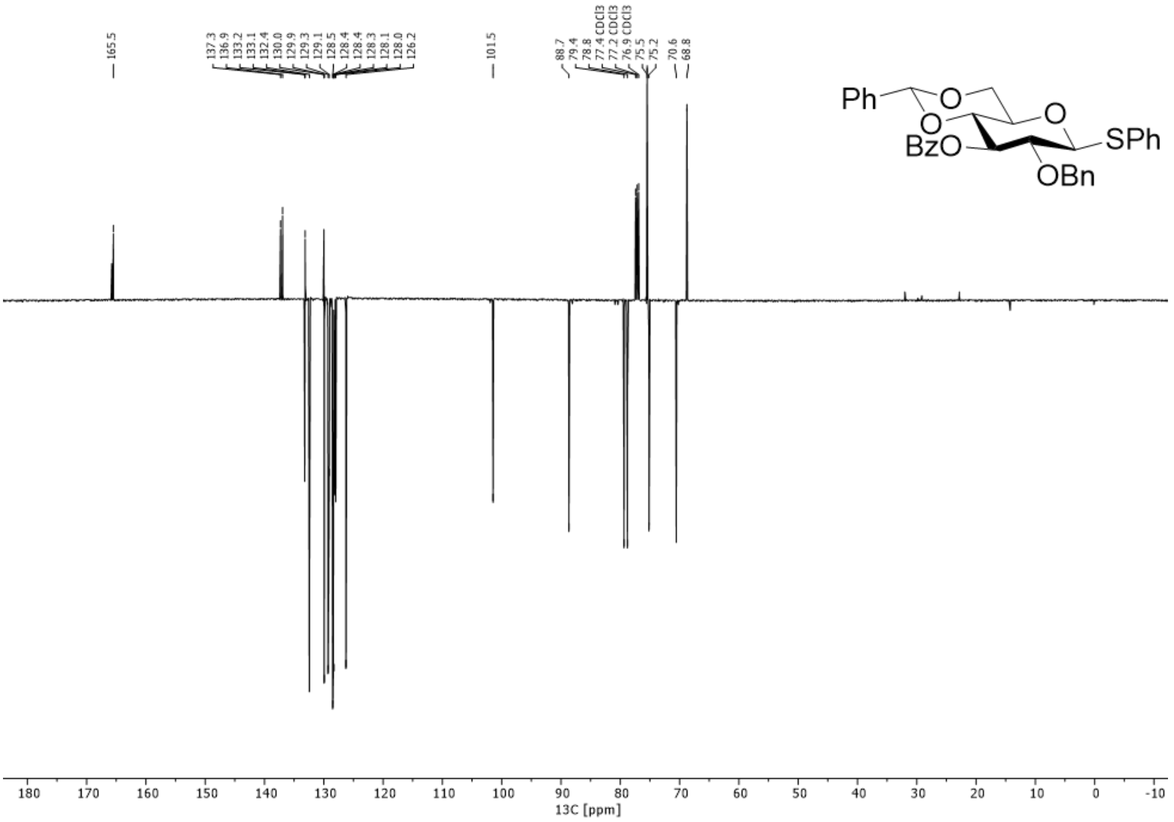

Figure S183: ^{13}C NMR of phenyl 3-*O*-benzoyl-2-*O*-benzyl-4,6-*O*-benzylidene-1-thio- β-D-glucopyranoside.

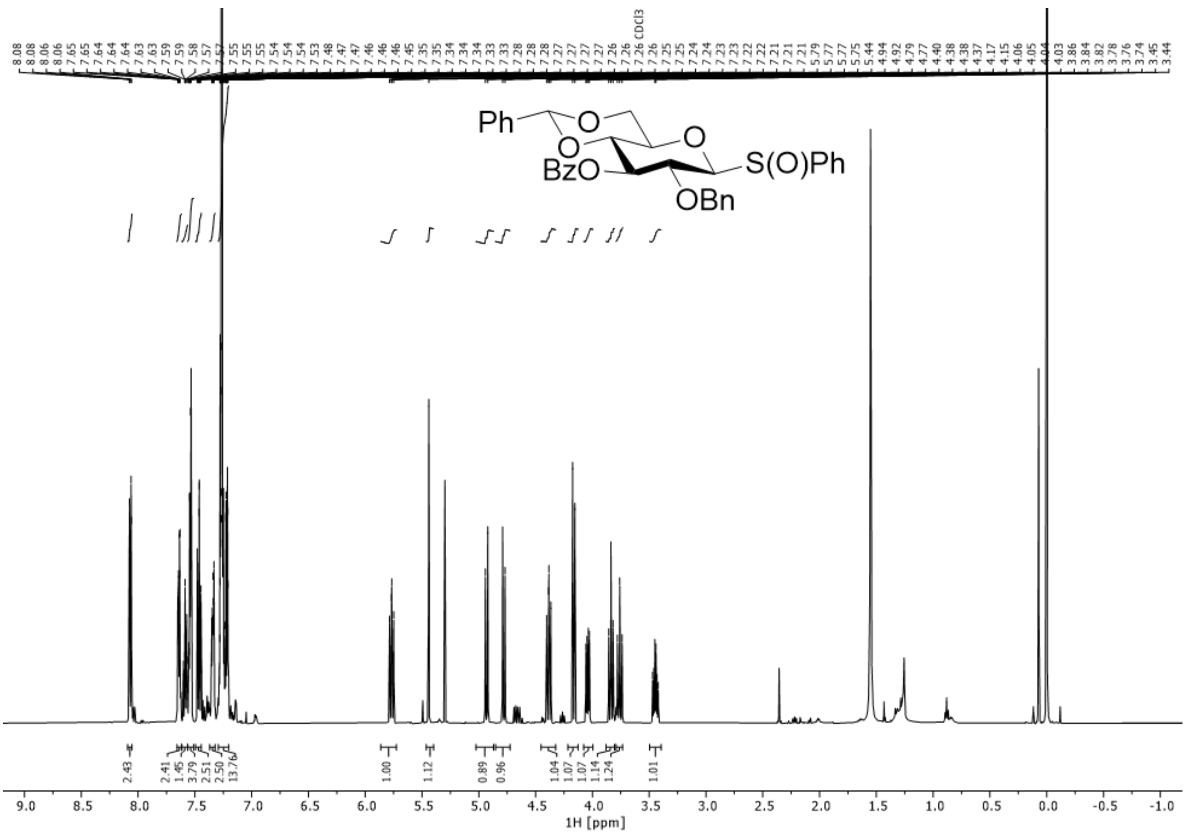

Figure S184: ^{1}H NMR of phenyl 3-*O*-benzoyl-2-*O*-benzyl-4,6-*O*-benzylidene-1-thiosulfinyl- β-D-glucopyranoside.

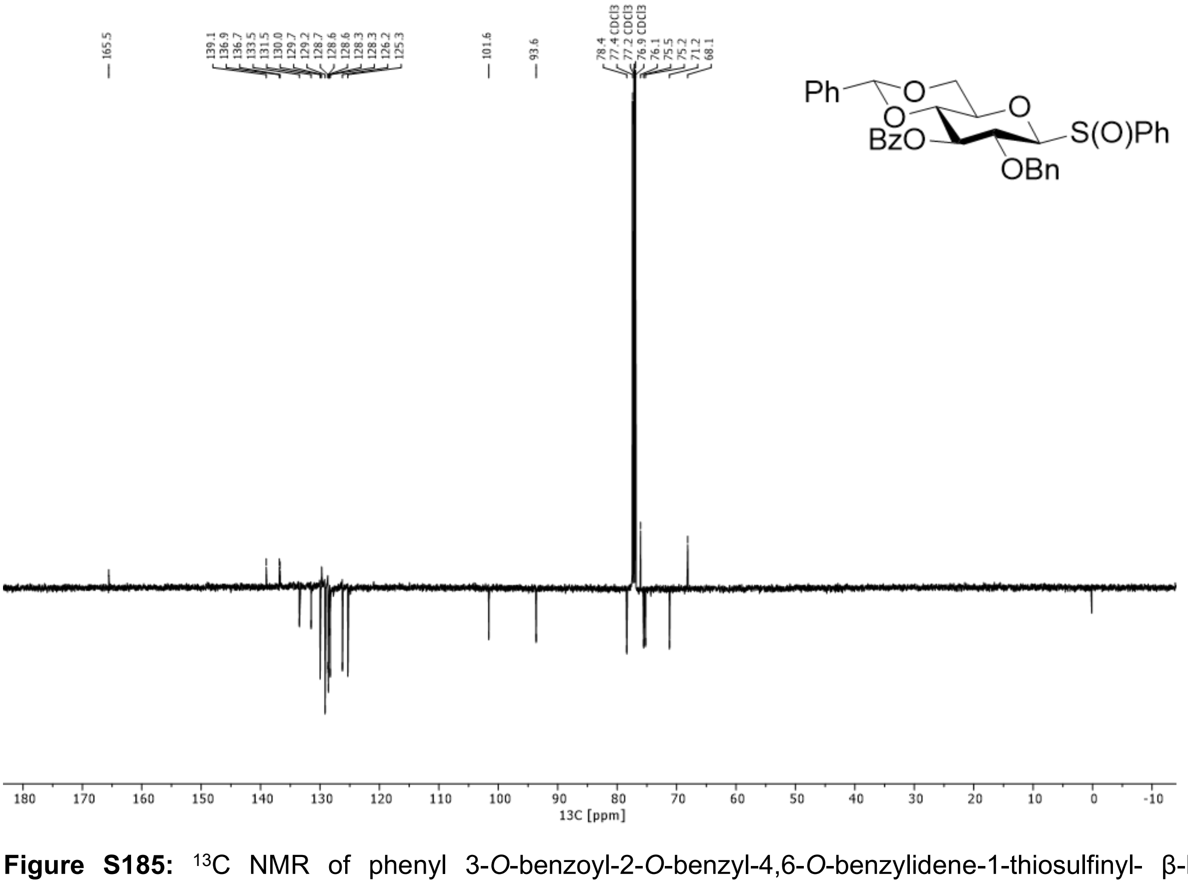

Figure S185: ^{13}C NMR of phenyl 3-*O*-benzoyl-2-*O*-benzyl-4,6-*O*-benzylidene-1-thiosulfinyl- β-D-glucopyranoside.

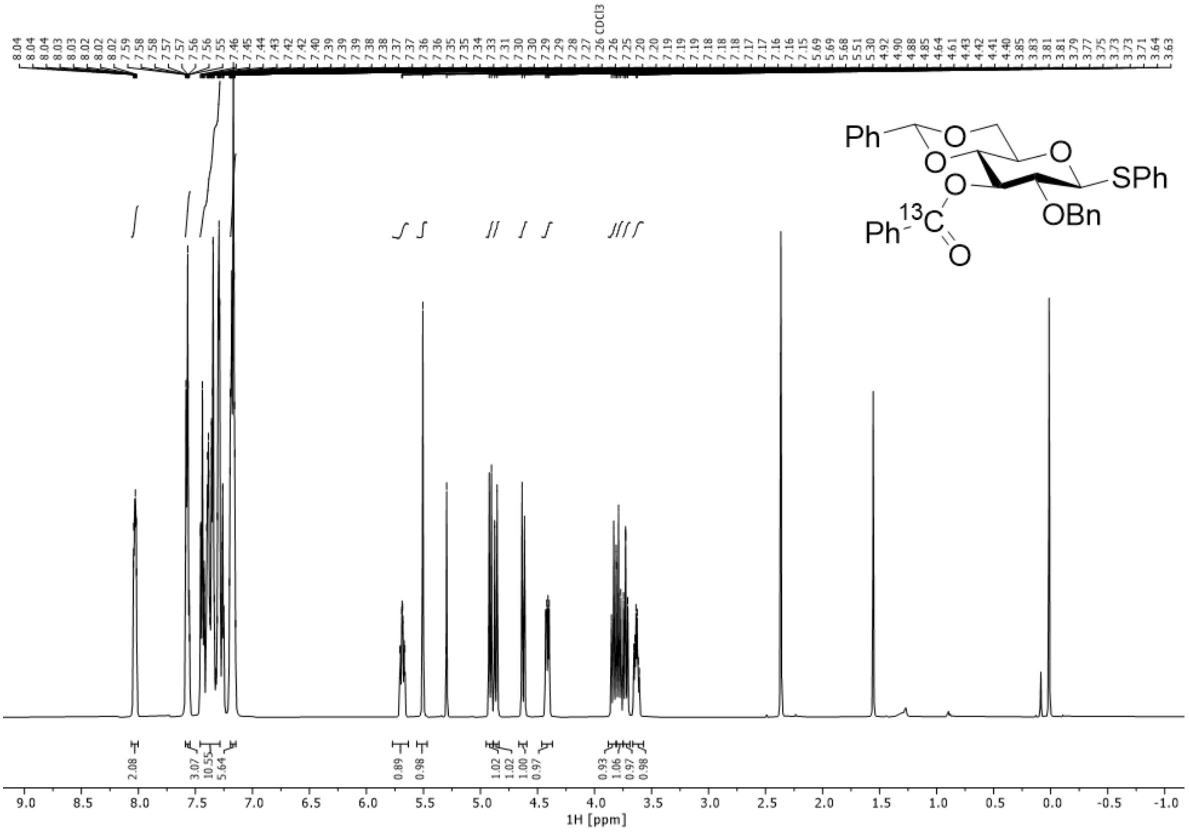

Figure S186: ^{1}H NMR of phenyl 3-*O*-(benzoyl-α- ^{13}C)-2-*O*-benzyl-4,6-*O*-benzylidene-1-thio- β-D-glucopyranoside.

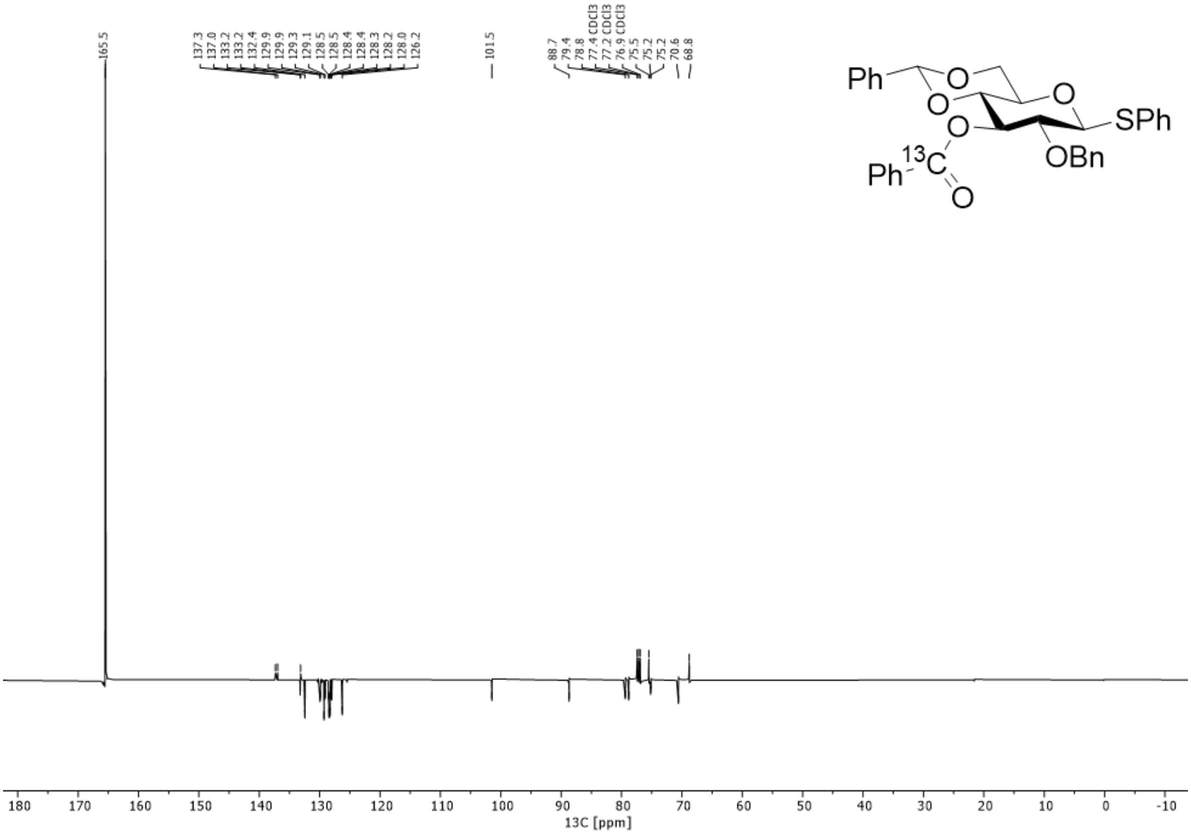

Figure S187: ^{13}C NMR of phenyl 3-*O*-(benzoyl-α- ^{13}C)-2-*O*-benzyl-4,6-*O*-benzylidene-1-thio- β-D-glucopyranoside.

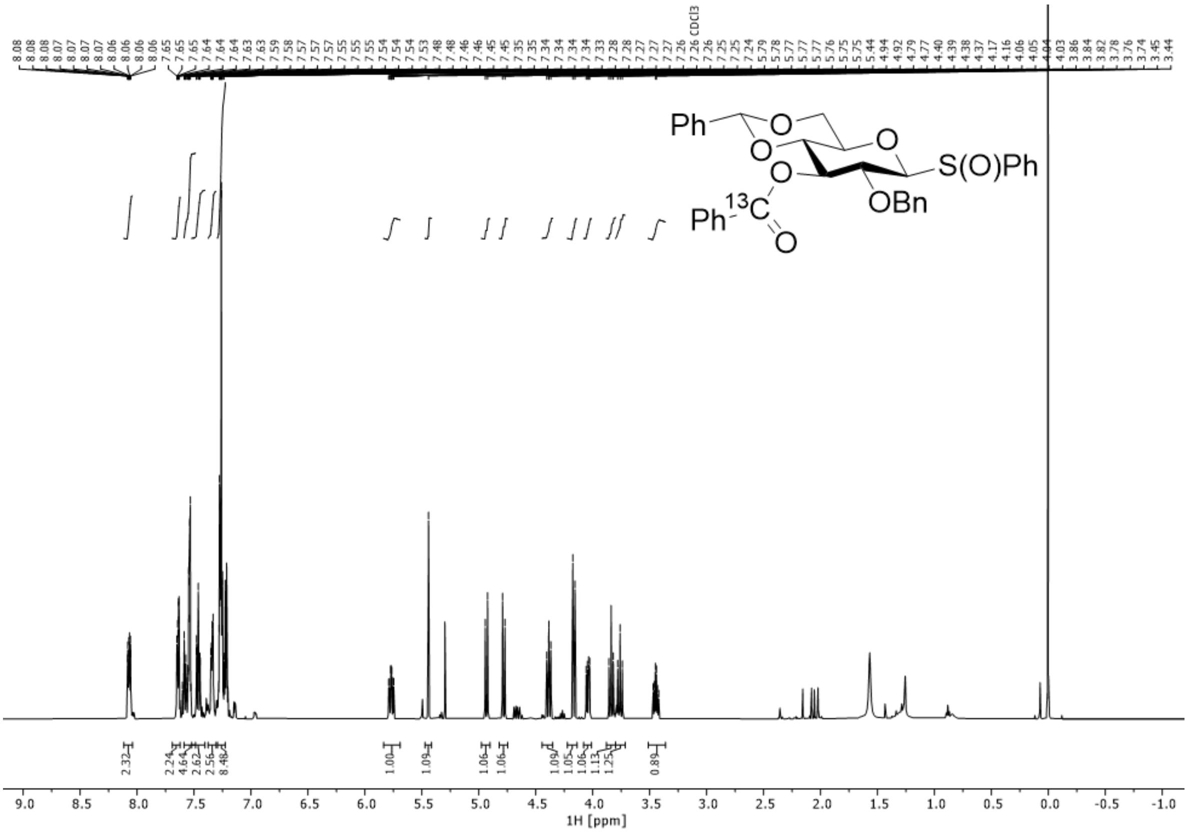

Figure S188: ^{1}H NMR of phenyl 3-*O*-(benzoyl-α- ^{13}C)-2-*O*-benzyl-4,6-*O*-benzylidene-1-thiosulfinyl- β-D-glucopyranoside.

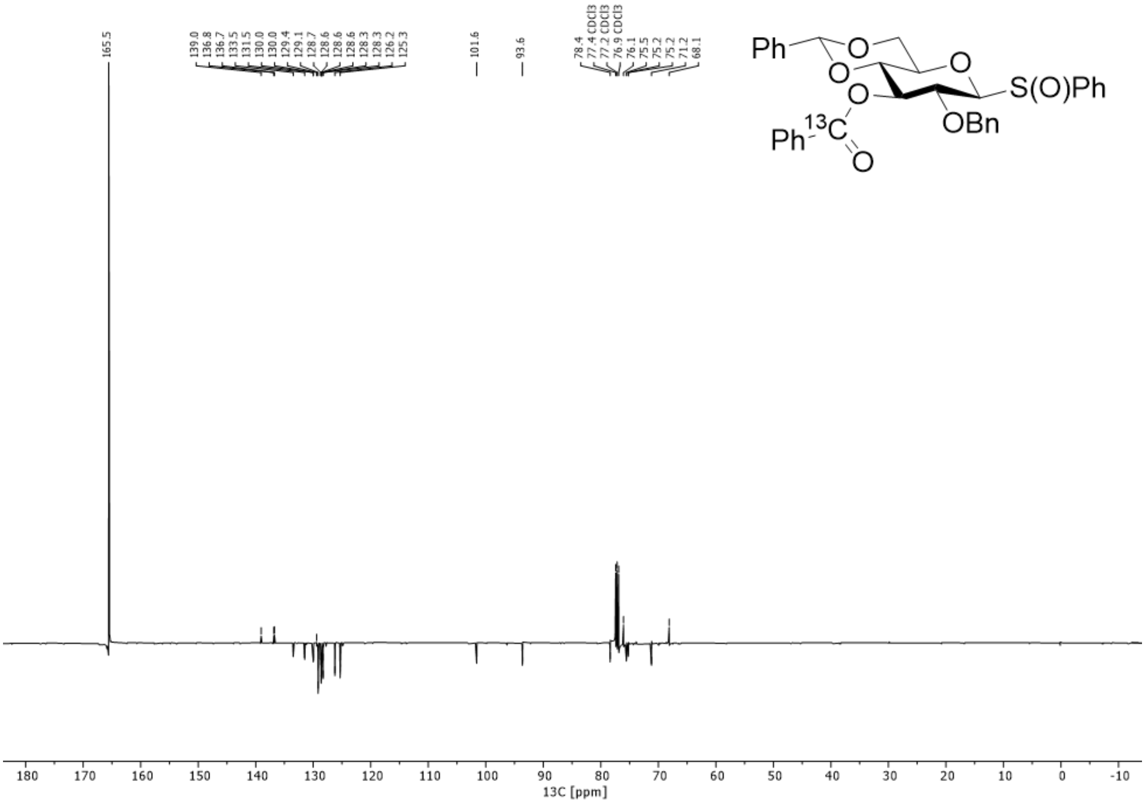

Figure S189: ^{13}C NMR of phenyl 3-*O*-(benzoyl-α- ^{13}C)-2-*O*-benzyl-4,6-*O*-benzylidene-1-thiosulfinyl- β-D-glucopyranoside.

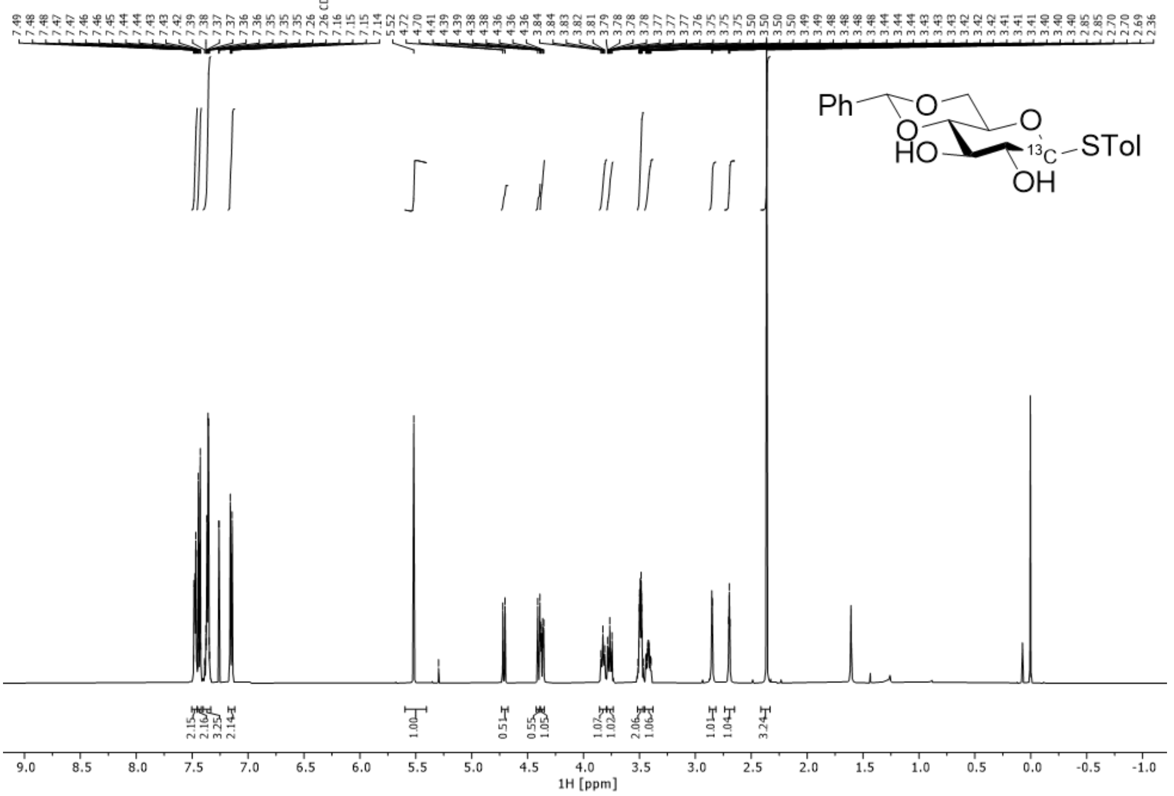

Figure S190: ^{1}H NMR of *p*-methylphenyl 4,6-*O*-benzylidene-1-^{13}C-thio- β-D-glucopyranoside.

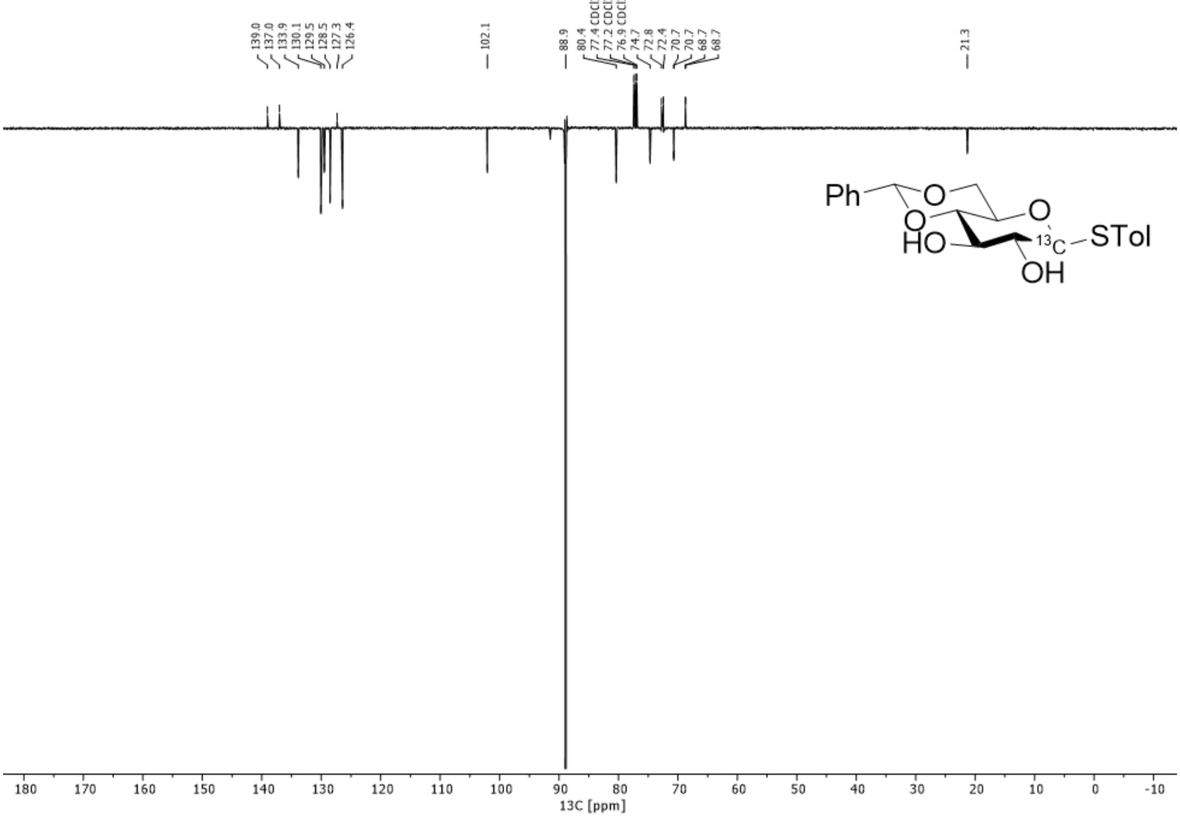

Figure S191: ^{13}C NMR of *p*-methylphenyl 4,6-*O*-benzylidene-1-^{13}C-thio- β-D-glucopyranoside.

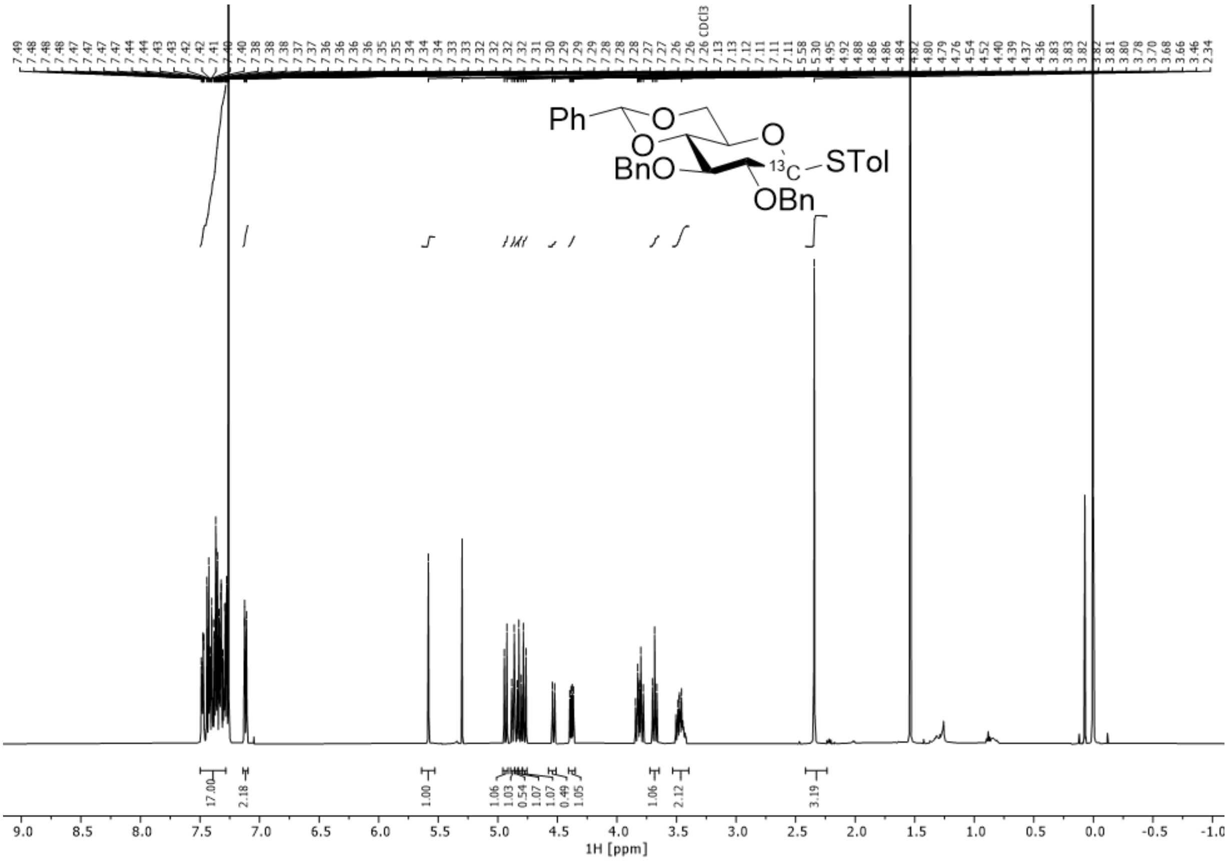

Figure S192: ^{1}H NMR of *p*-methylphenyl 2,3-di-*O*-benzyl-4,6-*O*-benzylidene-1-^{13}C -thio- β-D-glucopyranoside.

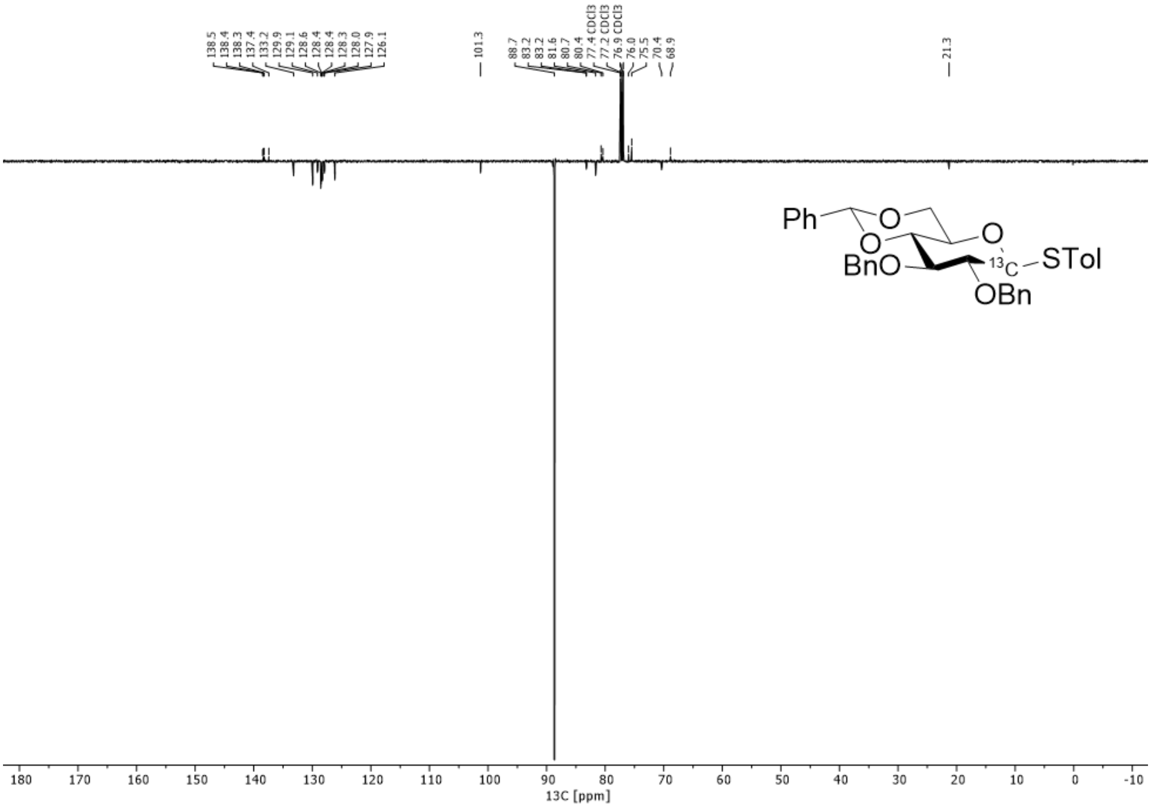

Figure S193: ^{13}C NMR of *p*-methylphenyl 2,3-di-*O*-benzyl-4,6-*O*-benzylidene-1-^{13}C -thio- β-D-glucopyranoside.

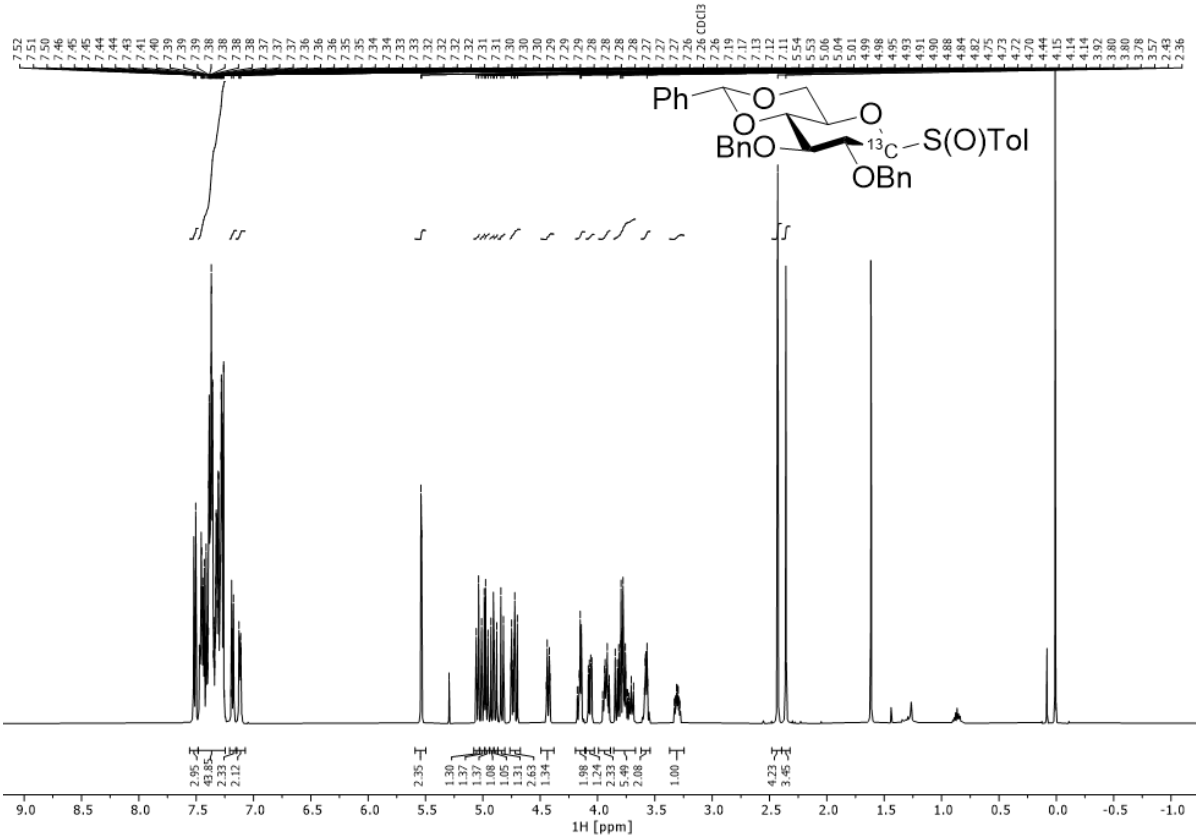

Figure S194: ^{1}H NMR of *p*-methylphenyl 2,3-di-*O*-benzyl-4,6-*O*-benzylidene-1-^{13}C -thiosulfinyl- β-D-glucopyranoside

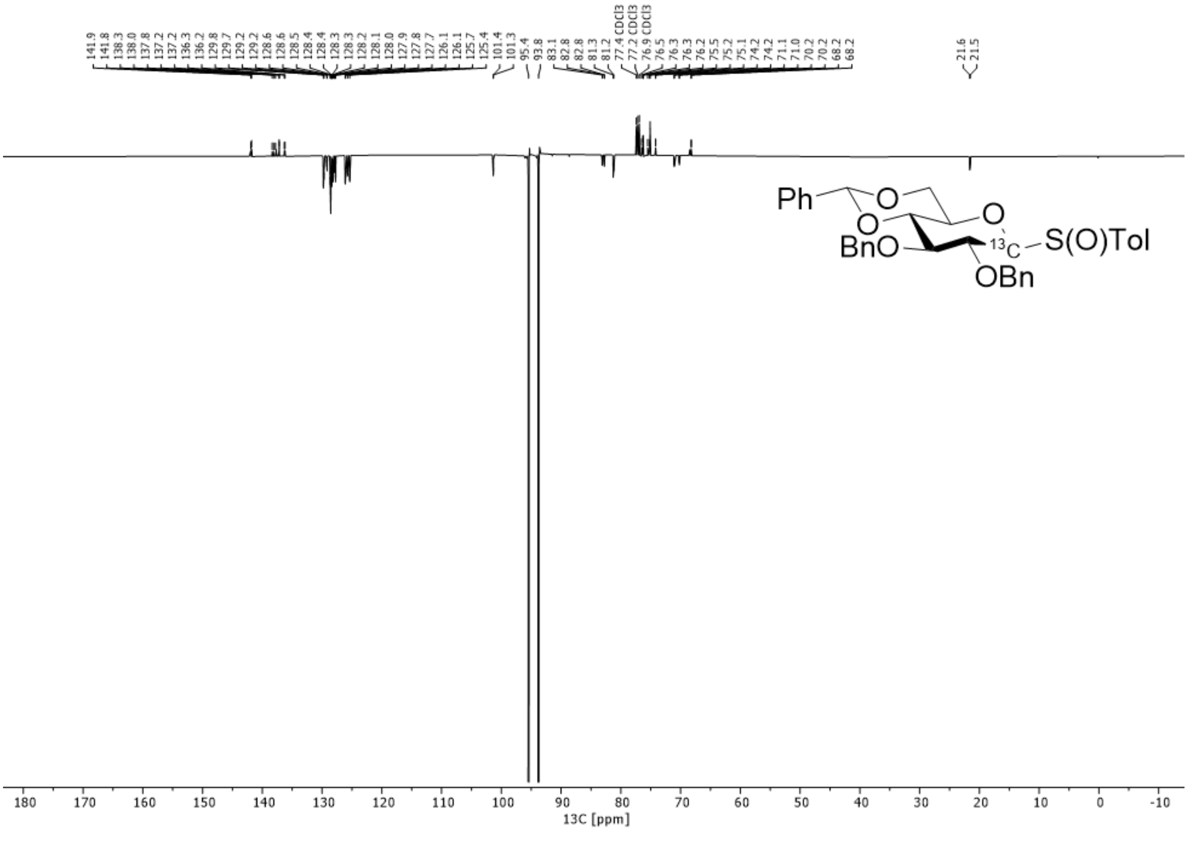

Figure S195: ^{13}C NMR of *p*-methylphenyl 2,3-di-*O*-benzyl-4,6-*O*-benzylidene-1-^{13}C -thiosulfinyl- β-D-glucopyranoside.

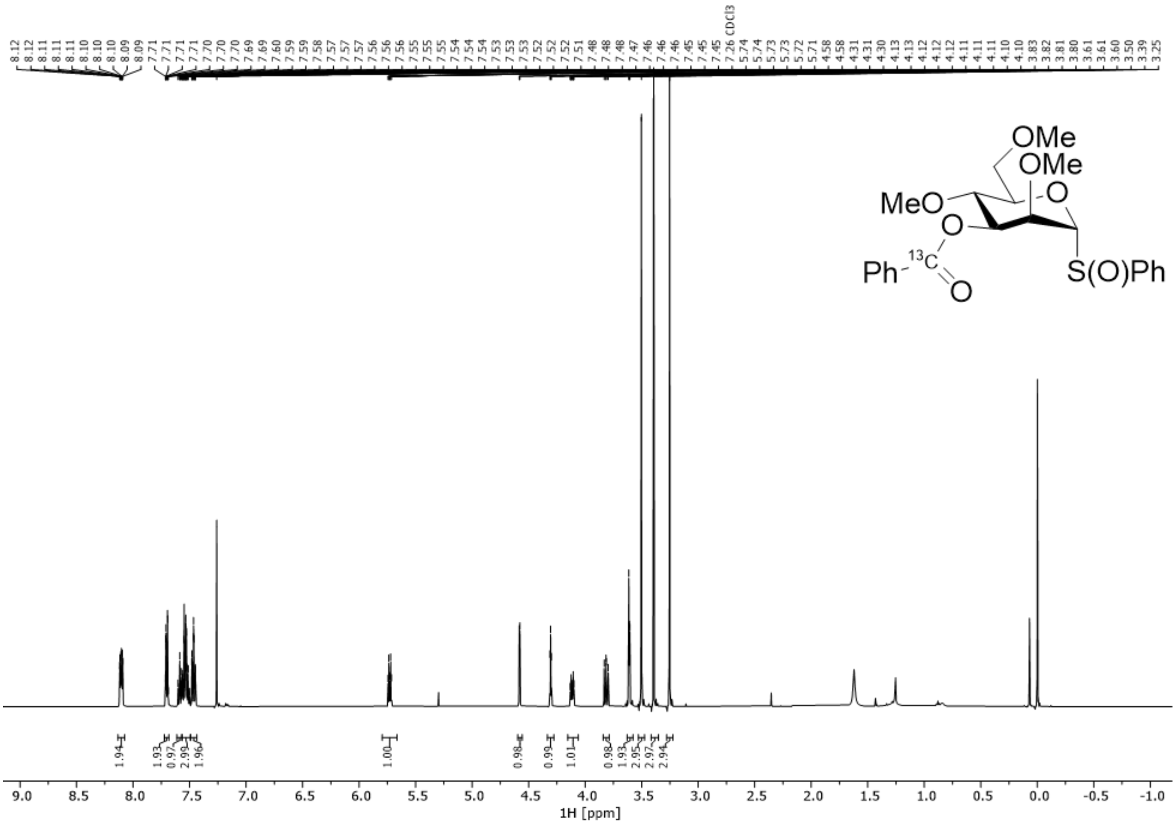

Figure S196: ^{1}H NMR of phenyl 3-*O*-(benzoyl-α-^{13}C)-2,4,6-tri-*O*-methyl-1-thiosulfinyl-α-D-mannopyranoside.

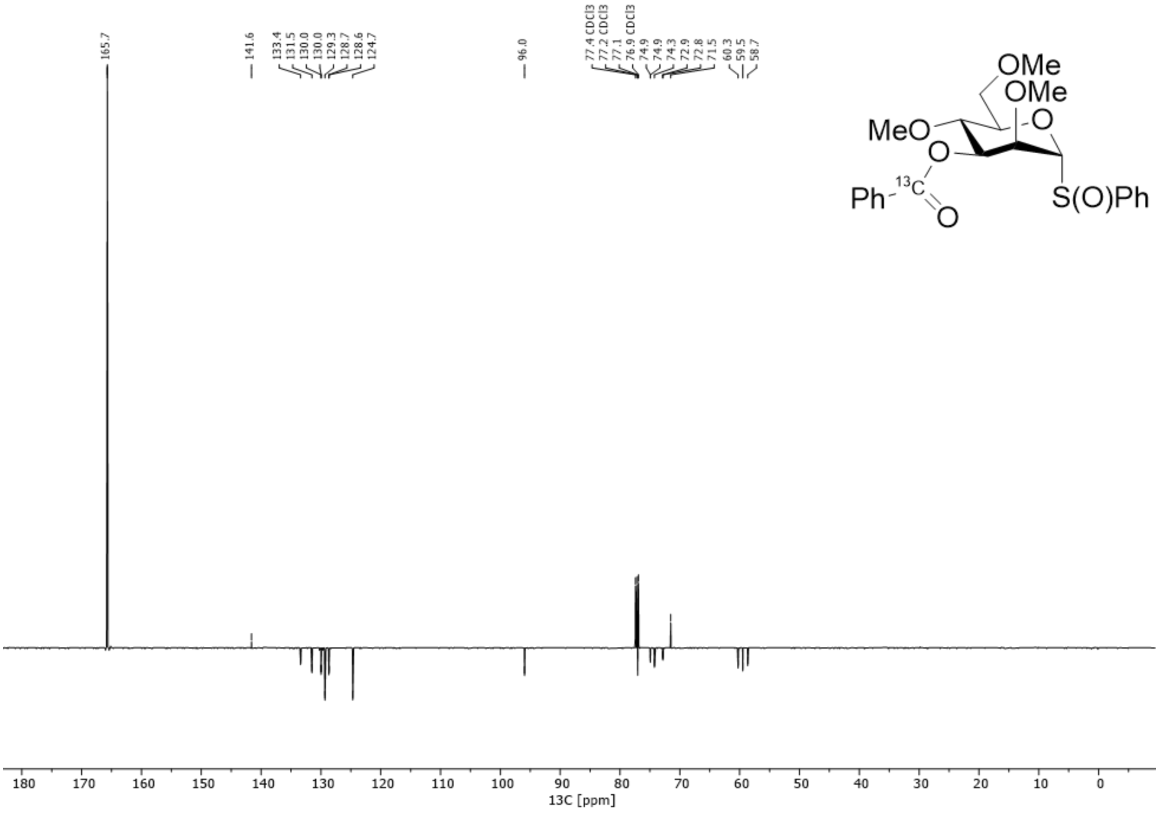

Figure S197: ^{13}C NMR of phenyl 3-*O*-(benzoyl-α-^{13}C)-2,4,6-tri-*O*-methyl-1-thiosulfinyl-α-D-mannopyranoside.

CEST profile data

See next pages

Table S8: *CEST profile data*

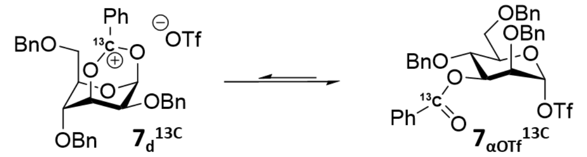 $7_d{}^{13C}$ ⇌ $7_{\alpha OTf}{}^{13C}$		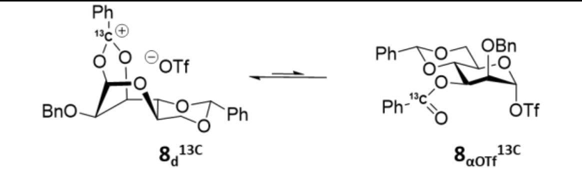 $8_d{}^{13C}$ ⇌ $8_{\alpha OTf}{}^{13C}$	
Saturation position [ppm]	Relative intensity [-]	Saturation position [ppm]	Relative intensity [-]
190	0.991565181	190	1
189.405	1	189.405	0.993368065
188.81	0.992691513	188.81	0.997167934
188.215	0.986782962	188.215	0.987483695
187.62	0.990756308	187.62	0.995651088
187.025	0.99372774	187.025	0.983525627
186.43	0.989424997	186.43	0.999830945
185.835	0.999080435	185.835	0.981037876
185.24	0.99999	185.24	1.000854583
184.645	0.988018199	184.645	0.965880275
184.05	0.992320942	184.05	0.980966532
183.455	0.984250726	183.455	0.979396953
182.86	0.991023943	182.86	0.973076762
182.265	0.977697106	182.265	0.974824702
181.67	0.988546606	181.67	0.964479751
181.075	0.977477508	181.075	0.97316982
180.48	0.972982617	180.48	0.961173089
179.885	0.948559233	179.885	0.985641147
179.29	0.963965386	179.29	0.963073024
178.695	0.907041538	178.695	0.963780265
178.1	0.818550518	178.1	0.970157842
177.505	0.659679113	177.505	0.965832195
176.91	0.668474688	176.91	0.9533314
176.315	0.808517647	176.315	0.94345329
175.72	0.8841622	175.72	0.986830738
175.125	0.90057713	175.125	0.965405679
174.53	0.932185478	174.53	0.939520038
173.935	0.941477206	173.935	0.946555225
173.34	0.95798821	173.34	0.931282851
172.745	0.954035451	172.745	0.936622831
172.15	0.930305172	172.15	0.947262466
171.555	0.93990571	171.555	0.931543414
170.96	0.937524447	170.96	0.910456465
170.365	0.938402838	170.365	0.924698996
169.77	0.923525092	169.77	0.912178039
169.175	0.919730169	169.175	0.891632377
168.58	0.897832159	168.58	0.842277502
167.985	0.839933846	167.985	0.787593504
167.39	0.776552453	167.39	0.695777802
166.795	0.623579306	166.795	0.489506932
166.2	0.325506276	166.2	0.224806478
165.605	0.014557476	165.605	-0.00133037
165.01	0.226159579	165.01	0.326211809
164.415	0.570647333	164.415	0.574748084
163.82	0.779125864	163.82	0.773380441
163.225	0.880216304	163.225	0.832360618
162.63	0.909045368	162.63	0.865137206
162.035	0.949286651	162.035	0.919630436

161.44<br>160.845<br>160.25	0.963375217<br>0.947598493<br>0.979296052	161.44<br>160.845<br>160.25	0.901079318<br>0.916387363<br>0.949463288
Temp = -50 °C; Saturation field = 80 Hz<br>Spectrometer frequency: 125.59 MHz		Temp = -50 °C; Saturation field = 80 Hz<br>Spectrometer frequency: 125.59 MHz	

Table S9: *CEST profile data*

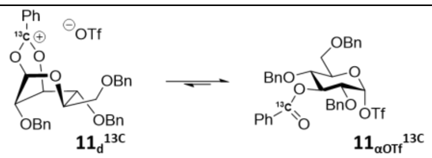		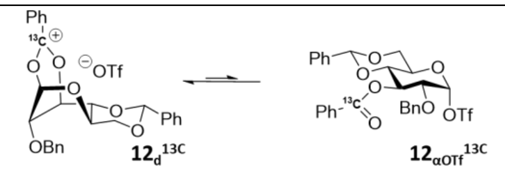	
Saturation position [ppm]	Relative intensity [-]	Saturation position [ppm]	Relative intensity [-]
190	1	189.9691611	0.948708706
189.405	0.959324256	188.9755204	0.99
188.81	0.99162984	187.9818797	0.978349517
188.215	0.952367572	186.988239	0.973896261
187.62	0.99	185.9945983	0.946005212
187.025	0.975085362	185.0009576	0.978109206
186.43	0.995163529	184.0073169	0.982014253
185.835	0.952819288	183.0136762	0.967467952
185.24	0.952708168	182.0200355	0.983606311
184.645	0.972752276	181.0263948	0.988254819
184.05	0.942371063	180.0327541	0.993902118
183.455	0.973041783	179.0391134	0.925541262
182.86	0.965029249	178.0454727	0.956736582
182.265	0.970359585	177.051832	0.95398803
181.67	0.958958115	176.0581913	0.95627849
181.075	0.954802837	175.0645506	0.974962639
180.48	0.965650837	174.0709099	0.923153175
179.885	0.964152213	173.0772692	0.940282816
179.29	0.955645814	172.0836285	0.949669948
178.695	0.95551809	171.0899878	0.965605545
178.1	0.9461602	170.0963471	0.909710801
177.505	0.974506348	169.1027064	0.910919864
176.91	0.96932928	168.1090657	0.873146041
176.315	0.950715679	167.115425	0.702317495
175.72	0.947011691	166.1217843	0.42361352
175.125	0.942881958	165.1281436	0.016712025
174.53	0.960524859	164.1345029	0.491701775
173.935	0.961103873	163.1408622	0.772200569
173.34	0.941255609	162.1472215	0.864690112
172.745	0.947394862	161.1535808	0.921215671
172.15	0.911981335	160.1599401	0.908306486
171.555	0.941664325	159.1662994	0.96010093
170.96	0.933694366		
170.365	0.955807597		
169.77	0.925698862		
169.175	0.917779992		
168.58	0.883073203		
167.985	0.830878484		
167.39	0.809602268		
166.795	0.669150467		
166.2	0.421228532		
165.605	0.013358027		
165.01	0.326555462		
164.415	0.616421011		
163.82	0.789618617		
163.225	0.866877837		

162.63<br>162.035<br>161.44<br>160.845<br>160.25	0.906361492<br>0.929964833<br>0.923825581<br>0.916613448<br>0.923144387		
Temp = -50 °C; Saturation field = 80 Hz<br>Spectrometer frequency: 125.59 MHz		Temp = -30 °C; Saturation field = 80 Hz<br>Spectrometer frequency: 75.48 MHz	

Table S10: *CEST profile data.*

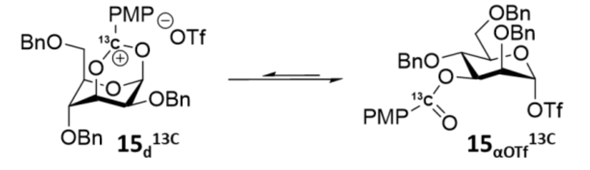		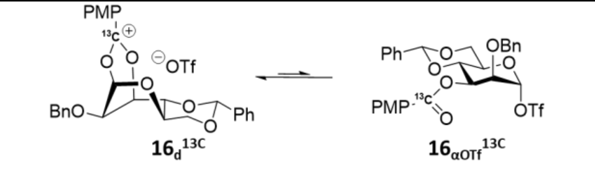	
Saturation position [ppm]	Relative intensity [-]	Saturation position [ppm]	Relative intensity [-]
190	0.971554741	190	0.961996264
189.405	0.995305543	189.405	0.980561419
188.81	0.965483064	188.81	0.963556725
188.215	0.988019915	188.215	0.99889684
187.62	0.972776376	187.62	0.978164377
187.025	1	187.025	1
186.43	0.989573849	186.43	0.99040533
185.835	0.9891724	185.835	0.95514107
185.24	0.993832283	185.24	0.988992245
184.645	0.971963873	184.645	0.984165652
184.05	0.989199292	184.05	0.967921682
183.455	0.957515549	183.455	0.968160086
182.86	0.969979678	182.86	0.987631176
182.265	0.982326637	182.265	0.991898605
181.67	0.9460003	181.67	1.006805345
181.075	0.946793594	181.075	0.973922957
180.48	0.949778531	180.48	0.977525022
179.885	0.902636118	179.885	1.007212799
179.29	0.944158244	179.29	0.998368017
178.695	0.925213306	178.695	0.935817357
178.1	0.937825337	178.1	0.972711432
177.505	0.846540815	177.505	0.968021378
176.91	0.799736465	176.91	0.928160259
176.315	0.616472089	176.315	0.931192323
175.72	0.217671826	175.72	0.804872974
175.125	0.080036265	175.125	0.598590817
174.53	0.473296915	174.53	0.389521502
173.935	0.830192811	173.935	0.640463197
173.34	0.881113454	173.34	0.765592694
172.745	0.89905573	172.745	0.829725489
172.15	0.885181728	172.15	0.832254737
171.555	0.930885454	171.555	0.881814557
170.96	0.944738328	170.96	0.871708402
170.365	0.937502641	170.365	0.845841587
169.77	0.949720906	169.77	0.865442716
169.175	0.982503352	169.175	0.807657964
168.58	0.936409701	168.58	0.803115288
167.985	0.928033053	167.985	0.695666685
167.39	0.976831203	167.39	0.609884656
166.795	0.910036995	166.795	0.396214147
166.2	0.83460875	166.2	0.143749052
165.605	0.768162208	165.605	0.052000642
165.01	0.781287096	165.01	0.139140923
164.415	0.878238004	164.415	0.390479452
163.82	0.912564779	163.82	0.57142145
163.225	0.938524511	163.225	0.696000451
162.63	0.959188573	162.63	0.779372434
162.035	0.98591855	162.035	0.852787808

161.44<br>160.845<br>160.25	0.949607579<br>0.987716427<br>0.961380216	161.44<br>160.845<br>160.25	0.872085513<br>0.870642087<br>0.899530128
Temp = -80 °C; Saturation field = 80 Hz<br>Spectrometer frequency: 125.59 MHz		Temp = -50 °C; Saturation field = 80 Hz<br>Spectrometer frequency: 125.59 MHz	

Table S11: *CEST profile data*

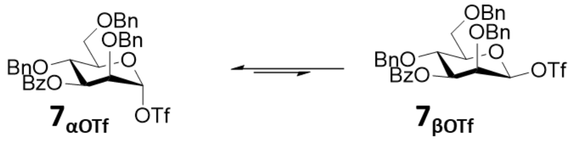		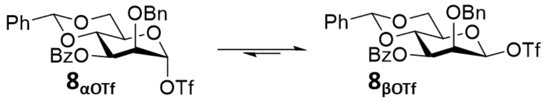	
Saturation position [ppm]	Relative intensity [-]	Saturation position [ppm]	Relative intensity [-]
-71	1	-71.01054657	1
-71.053	1.011532753	-71.11678639	0.97061316
-71.106	1.02394825	-71.22302621	1.002435902
-71.159	0.993755555	-71.32926603	0.971864795
-71.212	1.01826444	-71.43550584	0.968705568
-71.265	1.003698146	-71.54174566	0.986689406
-71.318	0.996155069	-71.64798548	1.013285774
-71.371	1.014517365	-71.7542253	0.972655488
-71.424	0.968843014	-71.86046512	0.986852509
-71.477	1.00841155	-71.96670494	0.978644192
-71.53	1.029937861	-72.07294476	0.994688527
-71.689	1.015956666	-72.17918457	0.988565087
-71.742	0.967672818	-72.28542439	0.983175608
-71.901	1.017412276	-72.39166421	0.979672447
-71.954	0.956335777	-72.49790403	0.981654499
-72.007	1.004976392	-72.60414385	0.995518223
-72.06	1.01028101	-72.71038367	0.979750453
-72.113	1.021285748	-72.81662349	0.978364081
-72.166	1.027780949	-72.92286331	0.992993678
-72.219	0.999439366	-73.02910312	0.988150239
-72.272	1.02293707	-73.13534294	0.990841432
-72.325	1.012209592	-73.24158276	0.986650404
-72.378	1.008607263	-73.34782258	0.988150239
-72.431	1.024594509	-73.4540624	0.985377494
-72.484	1.01995042	-73.56030222	0.983289071
-72.537	1.025257076	-73.66654204	0.984196773
-72.59	1.028011319	-73.77278185	0.990366307
-72.643	1.005989611	-73.87902167	0.977417376
-72.696	1.029585172	-73.98526149	0.981420482
-72.749	1.013634622	-74.09150131	0.988302704
-72.802	1.022698546	-74.19774113	0.988075779
-72.855	1.028967455	-74.30398095	0.980629789
-72.908	1.028111213	-74.41022077	0.990164202
-72.961	1.012658099	-74.51646059	0.973566736
-73.014	1.015351181	-74.6227004	0.970531608
-73.067	1.019648696	-74.72894022	0.966064014
-73.12	1.015267596	-74.83518004	0.979176048
-73.173	1.020774042	-74.94141986	0.97788541
-73.226	1.01829502	-75.04765968	0.983891842
-73.279	1.036475874	-75.1538995	0.978069787
-73.332	1.013811986	-75.26013932	0.975297042
-73.385	1.005789821	-75.36637914	0.971942801
-73.438	1.019624232	-75.47261895	0.922295776
-73.491	1.003537092	-75.57885877	0.909637593
-73.544	1.027964429	-75.68509859	0.850583801
-73.597	1.031342505	-75.79133841	0.708801515
-73.65	1.00493358	-75.89757823	0.791115161
-73.703	1.034822513	-76.00381805	0.849094603
-73.756	1.018352103	-76.11005787	0.935734015

-73.809	1.019365321	-76.21629768	0.967280193
-73.862	1.023726035	-76.3225375	0.97373693
-73.915	1.022898336	-76.42877732	0.96626612
-73.968	1.00697225	-76.53501714	0.977867681
-74.021	1.030117264	-76.64125696	0.974775823
-74.074	1.021697559	-76.74749678	0.968021246
-74.127	1.021505924	-76.8537366	0.972378923
-74.18	1.015387877	-76.95997642	0.976608954
-74.233	0.992126658	-77.06621623	0.978140701
-74.286	1.033462721	-77.17245605	0.988940932
-74.339	1.024360062	-77.27869587	0.976502583
-74.392	0.992681177	-77.38493569	0.978626463
-74.445	1.019232808	-77.49117551	0.984604529
-74.498	1.010071027	-77.59741533	0.958568384
-74.551	1.010205579	-77.70365515	0.980129844
-74.604	1.035224131	-77.80989496	0.983948573
-74.657	1.028050053	-77.91613478	0.977300368
-74.71	1.022270425	-78.0223746	0.946824994
-74.763	1.039798906	-78.12861442	0.952331481
-74.816	0.995718794	-78.23485424	0.920147076
-74.869	1.021825995	-78.34109406	0.930436725
-74.922	1.015522429	-78.44733388	0.909502856
-74.975	0.995415032	-78.5535737	0.837035645
-75.028	1.029152974	-78.65981351	0.849218703
-75.081	1.010505264	-78.76605333	0.613879325
-75.134	0.994067472	-78.87229315	0.375299169
-75.187	1.008853942	-78.97853297	0.004687038
-75.24	0.982255421	-79.08477279	0.351764168
-75.293	0.96750157	-79.19101261	0.545454223
-75.346	0.954364384	-79.29725243	0.824827767
-75.399	0.915609277	-79.40349225	0.926227968
-75.452	0.913160835	-79.50973206	0.955848825
-75.505	0.866357061	-79.61597188	0.940063326
-75.558	0.800296015	-79.7222117	0.923065195
-75.611	0.747529133	-79.82845152	0.961365949
-75.664	0.644763881	-79.93469134	0.993982931
-75.717	0.522639425	-80.04093116	0.986583035
-75.77	0.392831223	-80.14717098	0.972148452
-75.823	0.279943977	-80.25341079	0.975896267
-75.876	0.377816422	-80.35965061	0.971875432
-75.929	0.60662853	-80.46589043	0.99155412
-75.982	0.74673609	-80.57213025	0.993688637
-76.035	0.856516403	-80.67837007	0.974694271
-76.088	0.921615197	-80.78460989	0.978754109
-76.141	0.926770992	-80.89084971	0.983817382
-76.194	0.981876228	-80.99708953	0.995691963
-76.247	0.986400036		
-76.3	0.963905357		
-76.353	1.025754512		
-76.406	0.98662429		
-76.459	0.985592723		
-76.512	1.02952605		
-76.565	0.970145724		
-76.618	1.018189009		
-76.724	0.973509529		
-76.83	1.018502964		
-76.883	0.981164733		
-76.989	1.010401292		
-77.042	0.995490463		

-77.148	0.970584038		
-77.201	1.007899844		
-77.307	0.973505451		
-77.466	0.969525969		
-77.572	0.990691435		
-77.625	0.985019857		
-77.731	0.974773504		
-77.784	0.995851308		
-77.89	0.933205033		
-78.049	0.930723972		
-78.155	0.985087133		
-78.208	0.954250218		
-78.314	0.920785459		
-78.367	0.955026951		
-78.473	0.806573078		
-78.632	0.684799273		
-78.791	0.409248628		
-78.897	-0.012867878		
-78.95	0.180581469		
-79.003	0.15930041		
-79.109	0.658023388		
-79.268	0.819734728		
-79.321	0.992130736		
-79.427	0.833646609		
-79.586	0.93124791		
-79.692	0.95458456		
-79.745	0.951226871		
-79.851	0.95040121		
-80.01	0.949491964		
Temp = -60 °C; Saturation field = 30 Hz Spectrometer frequency: 469.96 MHz		Temp = -60 °C; Saturation field = 35 Hz Spectrometer frequency: 282.38	

Table S12: *CEST profile data*

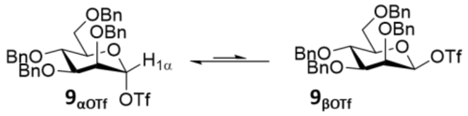 $\mathbf{9}_{\alpha OTf}$ ⇌ $\mathbf{9}_{\beta OTf}$		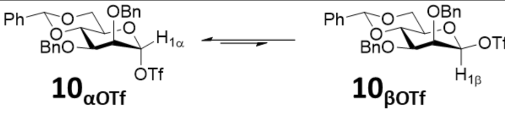 $\mathbf{10}_{\alpha OTf}$ ⇌ $\mathbf{10}_{\beta OTf}$	
Saturation position [ppm]	Relative intensity [-]	Saturation position [ppm]	Relative intensity [-]
-71.01104235	1	-71.01104235	1
-71.09957554	1.000984555	-71.09957554	0.989357607
-71.18810872	1.001727704	-71.18810872	0.990558401
-71.2766419	0.998917621	-71.2766419	0.989495429
-71.36517508	0.998952333	-71.36517508	0.992582959
-71.45370827	0.9989902	-71.45370827	0.993129494
-71.54224145	0.995227118	-71.54224145	0.991009887
-71.63077463	0.995698884	-71.63077463	0.992904543
-71.71930781	0.999637103	-71.71930781	0.99343999
-71.80784099	0.995126138	-71.80784099	0.991811472
-71.89637418	0.991030138	-71.89637418	0.991342561
-71.98490736	1.000178293	-71.98490736	0.995583359
-72.07344054	0.995806175	-72.07344054	0.993240385
-72.16197372	0.997849442	-72.16197372	0.996131479
-72.25050691	0.995282342	-72.25050691	0.993615831
-72.33904009	0.99605547	-72.33904009	0.995436032
-72.42757327	0.993253275	-72.42757327	0.99677148
-72.51610645	0.998895531	-72.51610645	0.996974253
-72.60463964	0.994177873	-72.60463964	0.994127515
-72.69317282	0.996752863	-72.69317282	0.998368314
-72.781706	0.994701707	-72.781706	0.996541777
-72.87023918	0.992409146	-72.87023918	0.996958411
-72.95877236	0.994446101	-72.95877236	0.999656237
-73.04730555	0.997333497	-73.04730555	0.999116038
-73.13583873	0.991258921	-73.13583873	0.998075244
-73.22437191	0.997819464	-73.22437191	1.001150101
-73.31290509	0.99464175	-73.31290509	0.99925386
-73.40143828	0.994916289	-73.40143828	0.998729503
-73.48997146	0.996623482	-73.48997146	1.001170695
-73.57850464	0.997849442	-73.57850464	1.001588913
-73.66703782	0.993586194	-73.66703782	0.997145342
-73.755571	0.997737417	-73.755571	1.001858221
-73.84410419	0.996367876	-73.84410419	0.998002373
-73.93263737	0.992712086	-73.93263737	0.998211482
-74.02117055	0.996183272	-74.02117055	0.997710887
-74.10970373	0.996718151	-74.10970373	1.000003168
-74.19823692	0.991962625	-74.19823692	0.995340983
-74.2867701	0.99628583	-74.2867701	0.993962763
-74.37530328	0.992073072	-74.37530328	0.990496619
-74.46383646	0.992260831	-74.46383646	0.987486713
-74.55236964	0.991132696	-74.55236964	0.978209866
-74.64090283	0.995771463	-74.64090283	0.971155598
-74.72943601	0.990187586	-74.72943601	0.946711995
-74.81796919	0.993267475	-74.81796919	0.900387961
-74.90650237	0.987226033	-74.90650237	0.891942799
-74.99503556	0.987503728	-74.99503556	0.921357499
-75.08356874	0.987462704	-75.08356874	0.920457696
-75.17210192	0.982901249	-75.17210192	0.898688156
-75.2606351	0.989078384	-75.2606351	0.852802935
-75.34916828	0.975815295	-75.34916828	0.744604744

-75.43770147	0.971700361	-75.43770147	0.642247354
-75.52623465	0.973620558	-75.52623465	0.638738438
-75.61476783	0.965963436	-75.61476783	0.531718862
-75.70330101	0.946065646	-75.70330101	0.430514741
-75.7918342	0.93013921	-75.7918342	0.511739425
-75.88036738	0.923032744	-75.88036738	0.602488083
-75.96890056	0.924519043	-75.96890056	0.669276308
-76.05743374	0.915394556	-76.05743374	0.770589736
-76.14596692	0.929979851	-76.14596692	0.841263907
-76.23450011	0.959888859	-76.23450011	0.885827938
-76.32303329	0.966867522	-76.32303329	0.905658464
-76.41156647	0.975886297	-76.41156647	0.922033936
-76.50009965	0.974595646	-76.50009965	0.933238706
-76.58863284	0.977899585	-76.58863284	0.93754604
-76.67716602	0.974799184	-76.67716602	0.943106445
-76.7656992	0.976212904	-76.7656992	0.953780521
-76.85423238	0.983298858	-76.85423238	0.957031219
-76.94276556	0.976908719	-76.94276556	0.951857349
-77.03129875	0.976171881	-77.03129875	0.956863298
-77.11983193	0.976189236	-77.11983193	0.960646274
-77.20836511	0.977683425	-77.20836511	0.959708451
-77.29689829	0.96745289	-77.29689829	0.95114923
-77.38543148	0.969089082	-77.38543148	0.954365076
-77.47396466	0.964955214	-77.47396466	0.942883078
-77.56249784	0.965816699	-77.56249784	0.94863675
-77.65103102	0.957405383	-77.65103102	0.94365298
-77.7395642	0.95505602	-77.7395642	0.943950802
-77.82809739	0.950407786	-77.82809739	0.941137332
-77.91663057	0.934355124	-77.91663057	0.916803037
-78.00516375	0.933055007	-78.00516375	0.923052551
-78.09369693	0.90553323	-78.09369693	0.89171468
-78.18223012	0.886069339	-78.18223012	0.868975634
-78.2707633	0.865101793	-78.2707633	0.84019143
-78.35929648	0.834560082	-78.35929648	0.808996133
-78.44782966	0.812869898	-78.44782966	0.786356889
-78.53636284	0.726563572	-78.53636284	0.669878289
-78.62489603	0.665910895	-78.62489603	0.591208857
-78.71342921	0.544473003	-78.71342921	0.442521097
-78.80196239	0.369073935	-78.80196239	0.237421366
-78.89049557	0.16172417	-78.89049557	0.04594637
-78.97902876	0.00332019	-78.97902876	0.082156645
-79.06756194	0.081451082	-79.06756194	0.237863347
-79.15609512	0.328580963	-79.15609512	0.51524042
-79.2446283	0.520501302	-79.2446283	0.693848228
-79.33316149	0.652059282	-79.33316149	0.799369187
-79.42169467	0.737825996	-79.42169467	0.860036214
-79.51022785	0.778566684	-79.51022785	0.881496842
-79.59876103	0.848465341	-79.59876103	0.949468435
-79.68729421	0.861630606	-79.68729421	0.948275562
-79.7758274	0.88112132	-79.7758274	0.958024488
-79.86436058	0.904212601	-79.86436058	0.975922338
-79.95289376	0.915080571	-79.95289376	0.989910479
-80.04142694	0.941322743	-80.04142694	1.010807146
-80.12996013	0.935262366	-80.12996013	0.997522372
-80.21849331	0.955450473	-80.21849331	1.016798442
-80.30702649	0.960736144	-80.30702649	1.02310974
-80.39555967	0.965595806	-80.39555967	1.021742609
-80.48409285	0.966989014	-80.48409285	1.025435288
-80.57262604	0.964780077	-80.57262604	1.01592557

-80.66115922<br>-80.7496924<br>-80.83822558	0.971227017<br>0.964691719<br>0.972937366	-80.66115922<br>-80.7496924<br>-80.83822558	1.029999255<br>1.019963596<br>1.026046774
Temp = -60 °C; Saturation field = 30 Hz<br>Spectrometer frequency: 282.38 MHz		Temp = -30 °C; Saturation field = 30 Hz<br>Spectrometer frequency: 282.38 MHz	

Table S13: *CEST profile data*

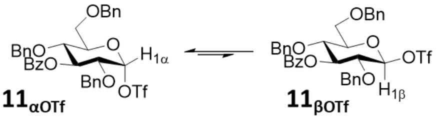 $\mathbf{11}_{\alpha OTf}$ ⇌ $\mathbf{11}_{\beta OTf}$		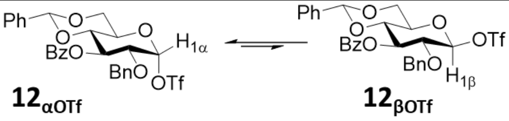 $\mathbf{12}_{\alpha OTf}$ ⇌ $\mathbf{12}_{\beta OTf}$	
Saturation position [ppm]	Relative intensity [-]	Saturation position [ppm]	Relative intensity [-]
-71.01054657	1	-71.01054657	1
-71.11678639	0.987466546	-71.11678639	0.98357458
-71.22302621	0.999214607	-71.22302621	0.988728588
-71.32926603	0.992332153	-71.32926603	0.977898608
-71.43550584	0.982986793	-71.43550584	0.98292138
-71.54174566	0.989286359	-71.54174566	0.981236187
-71.64798548	1.005462178	-71.64798548	0.997357377
-71.7542253	0.981410534	-71.7542253	0.978369866
-71.86046512	0.992274685	-71.86046512	0.979219919
-71.96670494	0.984171725	-71.96670494	0.974253817
-72.07294476	0.99379895	-72.07294476	0.991908087
-72.17918457	1.00208526	-72.17918457	0.992263021
-72.28542439	0.982264341	-72.28542439	0.9834851
-72.39166421	0.993905676	-72.39166421	0.983210697
-72.49790403	0.99215975	-72.49790403	0.986258959
-72.60414385	0.98987472	-72.60414385	0.984475338
-72.71038367	0.98943687	-72.71038367	0.984299362
-72.81662349	0.989995129	-72.81662349	0.98804258
-72.92286331	0.990148376	-72.92286331	0.991755972
-73.02910312	0.987212045	-73.02910312	0.988355758
-73.13534294	0.987712836	-73.13534294	0.986664599
-73.24158276	0.990944716	-73.24158276	0.983738625
-73.34782258	0.984193617	-73.34782258	0.987613079
-73.4540624	0.991423614	-73.4540624	0.982133962
-73.56030222	0.996436996	-73.56030222	0.988370671
-73.66654204	0.988205417	-73.66654204	0.990193067
-73.77278185	0.985682307	-73.77278185	0.985695239
-73.87902167	0.988889558	-73.87902167	0.983720729
-73.98526149	0.988722628	-73.98526149	0.981313735
-74.09150131	0.986393813	-74.09150131	0.98202957
-74.19774113	0.99257297	-74.19774113	0.985280652
-74.30398095	0.990703899	-74.30398095	0.97705452
-74.41022077	0.980124349	-74.41022077	0.977126103
-74.51646059	0.975236849	-74.51646059	0.972923558
-74.6227004	0.963382062	-74.6227004	0.974152407
-74.72894022	0.938914461	-74.72894022	0.965290972
-74.83518004	0.8789126	-74.83518004	0.960250303
-74.94141986	0.884139433	-74.94141986	0.940490287
-75.04765968	0.93700434	-75.04765968	0.9007734
-75.1538995	0.956472243	-75.1538995	0.821482792
-75.26013932	0.96850217	-75.26013932	0.785198927
-75.36637914	0.979273279	-75.36637914	0.840145195
-75.47261895	0.966597523	-75.47261895	0.882278024
-75.57885877	0.971044436	-75.57885877	0.894840921
-75.68509859	0.961192813	-75.68509859	0.869795659
-75.79133841	0.941771431	-75.79133841	0.778520788
-75.89757823	0.874348014	-75.89757823	0.825533222
-76.00381805	0.823973379	-76.00381805	0.862726793
-76.11005787	0.919517708	-76.11005787	0.933233514
-76.21629768	0.963721396	-76.21629768	0.951633445

-76.3225375	0.980337801	-76.3225375	0.961151062
-76.42877732	0.969413445	-76.42877732	0.948522547
-76.53501714	0.967073685	-76.53501714	0.952053998
-76.64125696	0.968154627	-76.64125696	0.94614538
-76.74749678	0.980439054	-76.74749678	0.95018388
-76.8537366	0.972705529	-76.8537366	0.946020109
-76.95997642	0.960210387	-76.95997642	0.933439317
-77.06621623	0.991927142	-77.06621623	0.960605238
-77.17245605	0.960114607	-77.17245605	0.933081399
-77.27869587	0.955733371	-77.27869587	0.920250065
-77.38493569	0.95621227	-77.38493569	0.91605945
-77.49117551	0.966827394	-77.49117551	0.920440954
-77.59741533	0.971413872	-77.59741533	0.914028269
-77.70365515	0.972341567	-77.70365515	0.904149753
-77.80989496	0.950044059	-77.80989496	0.879668211
-77.91613478	0.947852072	-77.91613478	0.868563827
-78.0223746	0.957129018	-78.0223746	0.863302443
-78.12861442	0.907381055	-78.12861442	0.79788113
-78.23485424	0.932740776	-78.23485424	0.798131672
-78.34109406	0.910407693	-78.34109406	0.745306064
-78.44733388	0.880144053	-78.44733388	0.676553137
-78.5535737	0.814190169	-78.5535737	0.566347424
-78.65981351	0.659131087	-78.65981351	0.38915451
-78.76605333	0.549810904	-78.76605333	0.235327927
-78.87229315	0.308366765	-78.87229315	0.079862977
-78.97853297	0.020304114	-78.97853297	0.018653008
-79.08477279	0.271397453	-79.08477279	0.184230761
-79.19101261	0.541576588	-79.19101261	0.358829968
-79.29725243	0.737881135	-79.29725243	0.541475156
-79.40349225	0.799103502	-79.40349225	0.634357673
-79.50973206	0.859056105	-79.50973206	0.716499688
-79.61597188	0.923381734	-79.61597188	0.800666919
-79.7222117	0.928589412	-79.7222117	0.816928294
-79.82845152	0.958691595	-79.82845152	0.874922824
-79.93469134	0.945318016	-79.93469134	0.878496032
-80.04093116	0.960612662	-80.04093116	0.898470798
-80.14717098	0.975110968	-80.14717098	0.923489216
-80.25341079	0.962265545	-80.25341079	0.925717251
-80.35965061	0.98692197	-80.35965061	0.943061326
-80.46589043	0.973526498	-80.46589043	0.941516317
-80.57213025	0.982228766	-80.57213025	0.957097649
-80.67837007	0.991803996	-80.67837007	0.968935763
-80.78460989	0.973778262	-80.78460989	0.954648898
-80.89084971	0.992269212	-80.89084971	0.977245409
-80.99708953	0.985602947	-80.99708953	0.972843026
Temp = -60 °C; Saturation field = 35 Hz<br>Spectrometer frequency: 282.38 MHz		Temp = -30 °C; Saturation field = 35 Hz<br>Spectrometer frequency: 282.38 MHz	

Table S14: *CEST profile data*

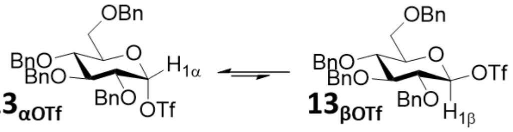 $\mathbf{13_{\alpha OTf}}$ ⇌ $\mathbf{13_{\beta OTf}}$		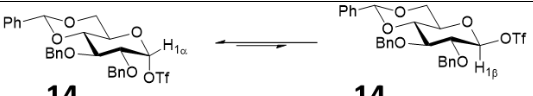 $\mathbf{14_{\alpha OTf}}$ ⇌ $\mathbf{14_{\beta OTf}}$	
Saturation position [ppm]	Relative intensity [-]	Saturation position [ppm]	Relative intensity [-]
-71.01104235	1	-71.01104235	0.921741111
-71.09957554	0.998426099	-71.09957554	0.928637067
-71.18810872	0.999036846	-71.18810872	0.923578985
-71.2766419	0.999911547	-71.2766419	0.931582378
-71.36517508	1.000318711	-71.36517508	0.934865419
-71.45370827	0.998809395	-71.45370827	0.930600608
-71.54224145	0.999194095	-71.54224145	0.929603129
-71.63077463	0.996751109	-71.63077463	0.928425005
-71.71930781	0.998522976	-71.71930781	0.9300194
-71.80784099	0.994011875	-71.80784099	0.93903598
-71.89637418	0.996268128	-71.89637418	0.934535544
-71.98490736	1.001234129	-71.98490736	0.940983813
-72.07344054	0.999855386	-72.07344054	0.928370025
-72.16197372	1.001766251	-72.16197372	0.930498504
-72.25050691	0.998416271	-72.25050691	0.938407647
-72.33904009	0.996730049	-72.33904009	0.932964711
-72.42757327	0.996567183	-72.42757327	0.939177355
-72.51610645	0.999802034	-72.51610645	0.938996709
-72.60463964	0.999205327	-72.60463964	0.939962771
-72.69317282	0.998774295	-72.69317282	0.934865419
-72.781706	0.99701366	-72.781706	0.938030647
-72.87023918	0.99707684	-72.87023918	0.938227001
-72.95877236	0.995627897	-72.95877236	0.938195584
-73.04730555	1.000474557	-73.04730555	0.941745666
-73.13583873	0.995967668	-73.13583873	0.940025605
-73.22437191	0.996415549	-73.22437191	0.941604292
-73.31290509	0.995800591	-73.31290509	0.943002333
-73.40143828	0.996543315	-73.40143828	0.939750709
-73.48997146	0.995925548	-73.48997146	0.943552124
-73.57850464	1.000268167	-73.57850464	0.943850582
-73.66703782	0.997187758	-73.66703782	0.941439354
-73.755571	0.999720601	-73.755571	0.947604873
-73.84410419	0.996067353	-73.84410419	0.943929124
-73.93263737	0.996588243	-73.93263737	0.946937269
-74.02117055	0.99523056	-74.02117055	0.940567542
-74.10970373	0.994086288	-74.10970373	0.944423936
-74.19823692	0.999376617	-74.19823692	0.943874145
-74.2867701	0.996482942	-74.2867701	0.943230103
-74.37530328	0.994037147	-74.37530328	0.940669646
-74.46383646	0.994066632	-74.46383646	0.938289834
-74.55236964	0.990984818	-74.55236964	0.934574815
-74.64090283	0.985322425	-74.64090283	0.923099881
-74.72943601	0.983321703	-74.72943601	0.908663928
-74.81796919	0.974146464	-74.81796919	0.881292167
-74.90650237	0.951680817	-74.90650237	0.830460018
-74.99503556	0.918863365	-74.99503556	0.728193307
-75.08356874	0.930028094	-75.08356874	0.559429316
-75.17210192	0.958003097	-75.17210192	0.585010328
-75.2606351	0.974972025	-75.2606351	0.703041132

-75.34916828	0.979601063	-75.34916828	0.731647568
-75.43770147	0.984482824	-75.43770147	0.747528687
-75.52623465	0.983707808	-75.52623465	0.812560379
-75.61476783	0.989808254	-75.61476783	0.83870689
-75.70330101	0.983480357	-75.70330101	0.832525663
-75.7918342	0.97927112	-75.7918342	0.769234455
-75.88036738	0.976067157	-75.88036738	0.613063831
-75.96890056	0.961075083	-75.96890056	0.407327935
-76.05743374	0.926439431	-76.05743374	0.599819354
-76.14596692	0.871564901	-76.14596692	0.753863071
-76.23450011	0.906602101	-76.23450011	0.855365572
-76.32303329	0.95472753	-76.32303329	0.891651809
-76.41156647	0.959544706	-76.41156647	0.914688072
-76.50009965	0.963897153	-76.50009965	0.91536353
-76.58863284	0.974496064	-76.58863284	0.93175517
-76.67716602	0.972903911	-76.67716602	0.934409877
-76.7656992	0.972513594	-76.7656992	0.936844668
-76.85423238	0.985117439	-76.85423238	0.942578208
-76.94276556	0.974223685	-76.94276556	0.941235146
-77.03129875	0.986011797	-77.03129875	0.942538937
-77.11983193	0.984172537	-77.11983193	0.944950165
-77.20836511	0.98245683	-77.20836511	0.950589455
-77.29689829	0.976192114	-77.29689829	0.948892956
-77.38543148	0.962538066	-77.38543148	0.953094933
-77.47396466	0.974820391	-77.47396466	0.949631247
-77.56249784	0.963992626	-77.56249784	0.951924663
-77.65103102	0.952113253	-77.65103102	0.944180457
-77.7395642	0.945025785	-77.7395642	0.946701644
-77.82809739	0.951079898	-77.82809739	0.932760503
-77.91663057	0.945001916	-77.91663057	0.930097941
-78.00516375	0.926160032	-78.00516375	0.933616607
-78.09369693	0.902978337	-78.09369693	0.909810636
-78.18223012	0.898040416	-78.18223012	0.907682158
-78.2707633	0.871517165	-78.2707633	0.893473975
-78.35929648	0.820637058	-78.35929648	0.864554944
-78.44782966	0.79663261	-78.44782966	0.831323976
-78.53636284	0.732429803	-78.53636284	0.782024175
-78.62489603	0.671300385	-78.62489603	0.676673133
-78.71342921	0.524902316	-78.71342921	0.523845242
-78.80196239	0.348584682	-78.80196239	0.242000141
-78.89049557	0.157885722	-78.89049557	0.0
-78.97902876	0.00254649	-78.97902876	0.203651401
-79.06756194	0.101240307	-79.06756194	0.513404702
-79.15609512	0.356264646	-79.15609512	0.669039671
-79.2446283	0.521375429	-79.2446283	0.814398253
-79.33316149	0.651370389	-79.33316149	0.852404552
-79.42169467	0.757530787	-79.42169467	0.892130913
-79.51022785	0.800270413	-79.51022785	0.931016879
-79.59876103	0.844401419	-79.59876103	0.945523519
-79.68729421	0.86243459	-79.68729421	0.956252307
-79.7758274	0.899027439	-79.7758274	0.967436637
-79.86436058	0.916798059	-79.86436058	0.969510136
-79.95289376	0.919198925	-79.95289376	0.970656844
-80.04142694	0.938696484	-80.04142694	0.986082422
-80.12996013	0.947961581	-80.12996013	0.993426065
-80.21849331	0.962156174	-80.21849331	0.995931543
-80.30702649	0.95700063	-80.30702649	0.985776109
-80.39555967	0.960114736	-80.39555967	0.993920877
-80.48409285	0.972585199	-80.48409285	0.987598275

-80.57262604	0.978383782	-80.57262604	0.988839233
-80.66115922	0.970458116	-80.66115922	0.995695918
-80.7496924	0.975175607	-80.7496924	1.000369146
-80.83822558	0.985132883	-80.83822558	1
Temp = -60 ℃; Saturation field = 35 Hz<br>Spectrometer frequency: 282.38 MHz		Temp = -20 ℃; Saturation field = 35 Hz<br>Spectrometer frequency: 282.38 MHz	

Table S15: *CEST profile data*

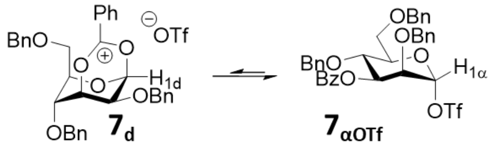 7_d ⇌ $7_{\alpha OTf}$		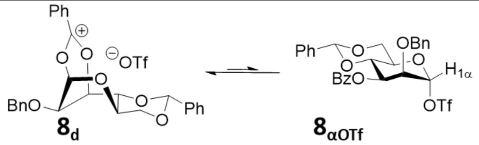 8_d ⇌ $8_{\alpha OTf}$	
Saturation position [ppm]	Relative intensity [-]	Saturation position [ppm]	Relative intensity [-]
7.02	0.986132432	7.000866291	0.905595632
6.984	0.961756757	6.950887949	0.93573675
6.948	0.984556757	6.900909606	0.945326841
6.912	0.944986486	6.850931263	0.9240217
6.876	0.941313514	6.80095292	0.921180625
6.84	0.981616216	6.750974578	0.920502508
6.804	0.920262162	6.700996235	0.907033707
6.768	0.9495	6.651017892	0.93754019
6.732	0.939151351	6.60103955	0.939428394
6.696	0.974608108	6.551061207	0.911172557
6.66	0.914678378	6.501082864	0.880376706
6.624	0.931	6.451104521	0.886131929
6.588	0.9438	6.401126179	0.843878243
6.552	0.88577027	6.351147836	0.777764787
6.516	0.853545946	6.301169493	0.676804317
6.48	0.845675676	6.251191151	0.393591213
6.444	0.724094595	6.201212808	0.061613625
6.408	0.576486486	6.151234465	0.280511744
6.372	0.581532432	6.101256122	0.643637979
6.336	0.543783784	6.05127778	0.762843297
6.3	0.429813514	6.001299437	0.831084636
6.264	0.368986486	5.951321094	0.865583823
6.228	0.275218919	5.901342751	0.890335083
6.192	0.198252973	5.851364409	0.897315009
6.156	0.136397027	5.801386066	0.908313945
6.12	0.236103243	5.751407723	0.941962563
6.084	0.405186486	5.701429381	0.911689914
6.048	0.549572973	5.651451038	0.917065158
6.012	0.684848649	5.601472695	0.923928166
5.976	0.813021622	5.551494352	0.924878114
5.94	0.825672973	5.50151601	0.931562825
5.904	0.841751351	5.451537667	0.935479534
5.868	0.912964865	5.401559324	0.939048415
5.832	0.907140541	5.351580982	0.937879248
5.796	0.921951351	5.301602639	0.930115981
5.76	0.964056757	5.251624296	0.947247197
5.724	0.964864865	5.201645953	0.935549684
5.688	0.962162162	5.151667611	0.937072523
5.652	0.945945946	5.101689268	0.941652734
5.616	0.972972973	5.051710925	0.942772211
5.58	0.972972973	5.001732583	0.948188376
5.544	0.945945946		
5.508	0.972972973		
5.472	0.972972973		
5.436	0.992763922		
5.4	0.972972973		
5.364	0.986374738		
5.328	0.945945946		
5.292	0.972972973		

5.256<br>5.22<br>5.184<br>5.148<br>5.112<br>5.076<br>5.04<br>5.004	0.945945946<br>0.972972973<br>0.999362836<br>0.983629472<br>0.972972973<br>0.918918919<br>0.945945946<br>0.918918919		
Temp = -60 °C; Saturation field = 20 Hz Spectrometer frequency: 499.46 MHz		Temp = -60 °C; Saturation field = 20 Hz Spectrometer frequency: 300.13 MHz	

Table S16: *CEST profile data*

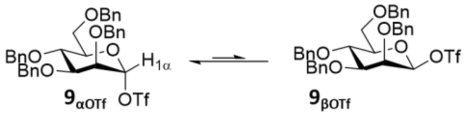		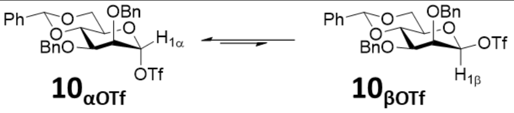	
Saturation position [ppm]	Relative intensity [-]	Saturation position [ppm]	Relative intensity [-]
7.02	0.988433579	7	1
6.984	0.999624134	6.966	0.9998
6.948	0.999624134	6.932	0.998
6.912	0.991662609	6.898	0.996598738
6.876	0.988433579	6.864	0.998711127
6.84	0.991662609	6.83	0.995039984
6.804	0.988433579	6.796	0.990801333
6.768	0.978208315	6.762	0.995526779
6.732	0.988433579	6.728	0.992246586
6.696	0.967735322	6.694	0.99031706
6.66	0.979882627	6.66	0.99230712
6.624	0.982103654	6.626	0.991385235
6.588	0.967735322	6.592	0.978248066
6.552	0.977593262	6.558	0.98518427
6.516	0.962071705	6.524	0.981442503
6.48	0.956621648	6.49	0.978345173
6.444	0.939297643	6.456	0.975459713
6.408	0.916310021	6.422	0.975130558
6.372	0.918992337	6.388	0.979629001
6.336	0.882678558	6.354	0.971207189
6.3	0.840036562	6.32	0.9732313
6.264	0.786250993	6.286	0.9615356
6.228	0.689258775	6.252	0.964941906
6.192	0.546744061	6.218	0.959643908
6.156	0.362034973	6.184	0.959081445
6.12	0.13308902	6.15	0.93432046
6.084	0.058266831	6.116	0.925211334
6.048	0.020800509	6.082	0.85285286
6.012	0.239471054	6.048	0.578374684
5.976	0.499662575	6.014	0.173565246
5.94	0.657797084	5.98	0.756239115
5.904	0.734958954	5.946	0.888898417
5.868	0.815937572	5.912	0.910893749
5.832	0.830658705	5.878	0.940555476
5.796	0.862843084	5.844	0.931849911
5.76	0.875801919	5.81	0.937722983
5.724	0.887496476	5.776	0.934647092
5.688	0.898994558	5.742	0.944282109
5.652	0.883831783	5.708	0.944477584
5.616	0.906136012	5.674	0.943449765
5.58	0.903163254	5.64	0.914616598
5.544	0.905572213	5.606	0.891744783
5.508	0.897063974	5.572	0.912839669
5.472	0.897610688	5.538	0.933793309
5.436	0.903761223	5.504	0.931564896
5.4	0.909800706	5.47	0.93372773
5.364	0.885685486	5.436	0.933967344
5.328	0.903462238	5.402	0.93077543
5.292	0.899541273	5.368	0.928477655
5.256	0.903974783	5.334	0.921948796

5.22<br>5.184<br>5.148<br>5.112<br>5.076<br>5.04<br>5.004	0.884455379<br>0.888854719<br>0.885480468<br>0.895885122<br>0.888154242<br>0.874640151<br>0.869395112	5.3<br>5.266<br>5.232<br>5.198<br>5.164<br>5.13<br>5.096<br>5.062<br>5.028	0.92444709<br>0.926773871<br>0.923486111<br>0.925568233<br>0.918937222<br>0.922050947<br>0.915230767<br>0.917309106<br>0.916660886
Temp = -50 °C; Saturation field = 20 Hz<br>Spectrometer frequency: 499.46 MHz		Temp = -30 °C; Saturation field = 20 Hz<br>Spectrometer frequency: 499.46 MHz	

Table S1: *CEST profile data*

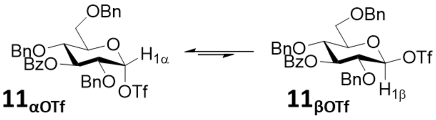 $11_{\alpha OTf}$ ⇌ $11_{\beta OTf}$		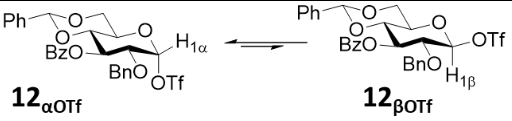 $12_{\alpha OTf}$ ⇌ $12_{\beta OTf}$	
Saturation position [ppm]	Relative intensity [-]	Saturation position [ppm]	Relative intensity [-]
7.02	1	7.02	0.988879642
6.984	0.990864594	6.984	0.978823215
6.948	1.000799121	6.948	0.978110417
6.912	0.988621607	6.912	0.993342533
6.876	0.986523915	6.876	0.98449049
6.84	0.976671116	6.84	0.97493267
6.804	0.987531897	6.804	0.992134638
6.768	0.982455662	6.768	0.983591591
6.732	0.974691476	6.732	0.984887269
6.696	0.966963613	6.696	0.979111144
6.66	0.963204112	6.66	0.970136204
6.624	0.960416269	6.624	0.978584446
6.588	0.950736009	6.588	0.964121309
6.552	0.947920923	6.552	0.960546783
6.516	0.94413418	6.516	0.968517485
6.48	0.927961061	6.48	0.967766062
6.444	0.919978932	6.444	0.964981583
6.408	0.909272527	6.408	0.953390708
6.372	0.891137022	6.372	0.9296015
6.336	0.874210187	6.336	0.91369521
6.3	0.822969279	6.3	0.889653187
6.264	0.705209724	6.264	0.79613263
6.228	0.444196838	6.228	0.608287423
6.192	0.046636609	6.192	0.170681864
6.156	0.207609811	6.156	0.104409167
6.12	0.547841011	6.12	0.51251962
6.084	0.710243278	6.084	0.728659061
6.048	0.785870996	6.048	0.828124287
6.012	0.819691975	6.012	0.867293087
5.976	0.851204584	5.976	0.888655971
5.94	0.852232544	5.94	0.900819191
5.904	0.869541686	5.904	0.907813745
5.868	0.854102306	5.868	0.830733199
5.832	0.862049019	5.832	0.794903667
5.796	0.880798394	5.796	0.868890738
5.76	0.86225334	5.76	0.928498945
5.724	0.854494601	5.724	0.959883143
5.688	0.838034526	5.688	0.9384711
5.652	0.815005312	5.652	0.966575723
5.616	0.741374488	5.616	0.966484429
5.58	0.681260613	5.58	0.967660722
5.544	0.716915938	5.544	0.966207035
5.508	0.800265163	5.508	0.981909668
5.472	0.847368803	5.472	0.983700442
5.436	0.861216298	5.436	0.967562405
5.4	0.872610129	5.4	0.957572693
5.364	0.860197419	5.364	0.967344703
5.328	0.862423153	5.328	0.966154365
5.292	0.878411021	5.292	0.972745117
5.256	0.868932356	5.256	0.963633235

5.22	0.870543311	5.22	0.979121678
5.184	0.879647842	5.184	0.970554052
5.148	0.876077224	5.148	0.961136685
5.112	0.855091218	5.112	0.967516758
5.076	0.861266243	5.076	0.967457065
5.04	0.847545881	5.04	0.975045033
5.004	0.859431898	5.004	0.965999867
Temp = -40 °C; Saturation field = 20 Hz Spectrometer frequency: 499.46 MHz		Temp = -30 °C; Saturation field = 20 Hz Spectrometer frequency: 499.46 MHz	

Table S2: *CEST profile data*

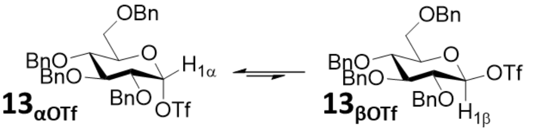 $13_{\alpha OTf}$ ⇌ $13_{\beta OTf}$		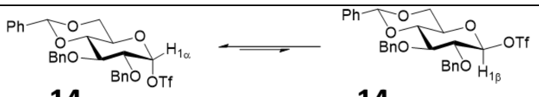 $14_{\alpha OTf}$ ⇌ $14_{\beta OTf}$	
Saturation position [ppm]	Relative intensity [-]	Saturation position [ppm]	Relative intensity [-]
7.02	0.948406289	7	1
6.984	0.950782007	6.968	0.997384294
6.948	0.963611403	6.936	0.998676876
6.912	0.930145565	6.904	0.999545477
6.876	0.931208386	6.872	0.997296047
6.84	0.944019548	6.84	0.994008972
6.804	0.929551635	6.808	0.997567624
6.768	0.918965104	6.776	0.998930043
6.732	0.941214013	6.744	0.998277461
6.696	0.92032489	6.712	0.994359159
6.66	0.929786081	6.68	0.996498135
6.624	0.920533286	6.648	0.976386076
6.588	0.922635484	6.616	0.96865358
6.552	0.931880464	6.584	0.972732779
6.516	0.918277396	6.552	0.982831399
6.48	0.909985829	6.52	0.980285449
6.444	0.911066885	6.488	0.957920839
6.408	0.908107658	6.456	0.955384353
6.372	0.896226464	6.424	0.96423366
6.336	0.87404268	6.392	0.950803536
6.3	0.839920393	6.36	0.935186167
6.264	0.79600869	6.328	0.926169339
6.228	0.695295975	6.296	0.920003218
6.192	0.52943337	6.264	0.921580003
6.156	0.227144398	6.232	0.880965239
6.12	0.041740995	6.2	0.842367828
6.084	0.236778298	6.168	0.768937515
6.048	0.494743204	6.136	0.608974238
6.012	0.650532453	6.104	0.359787238
5.976	0.734802701	6.072	0.063095364
5.94	0.786232299	6.04	0.118671563
5.904	0.815334841	6.008	0.369556494
5.868	0.830917673	5.976	0.574119328
5.832	0.842100739	5.944	0.683575309
5.796	0.863364975	5.912	0.746962842
5.76	0.861906201	5.88	0.80451078
5.724	0.851827635	5.848	0.826772227
5.688	0.855365162	5.816	0.825640273
5.652	0.854560232	5.784	0.814356698
5.616	0.846086318	5.752	0.803355165
5.58	0.810924654	5.72	0.765041928
5.544	0.813487929	5.688	0.714978516
5.508	0.774538663	5.656	0.589481156
5.472	0.750122433	5.624	0.486993886
5.436	0.756410791	5.592	0.531967291
5.4	0.803083744	5.56	0.689474531
5.364	0.843218264	5.528	0.847576142
5.328	0.85748299	5.496	0.881108861
5.292	0.878116827	5.464	0.916470121

5.256<br>5.22<br>5.184<br>5.148<br>5.112<br>5.076<br>5.04<br>5.004	0.897711288<br>0.89954257<br>0.889437955<br>0.906622834<br>0.923711329<br>0.904932219<br>0.908636463<br>0.901837534	5.432<br>5.4<br>5.368<br>5.336<br>5.304<br>5.272<br>5.24<br>5.208<br>5.176<br>5.144<br>5.112<br>5.08<br>5.048<br>5.016	0.939852165<br>0.943265063<br>0.972884211<br>0.952734294<br>0.950472278<br>0.94322058<br>0.938829052<br>0.954911128<br>0.952535539<br>0.960144996<br>0.939939238<br>0.945458934<br>0.963732041<br>0.948768669
Temp = -50 °C; Saturation field = 20 Hz Spectrometer frequency: 499.46 MHz		Temp = -30 °C; Saturation field = 20 Hz Spectrometer frequency: 499.46 MHz	

Table S3: *CEST profile data*

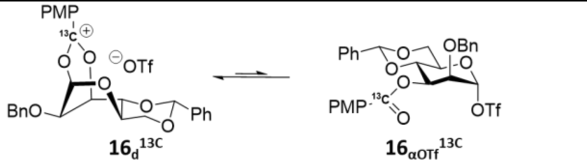

Saturation position [ppm]	Relative intensity [-]
7.02	0.948968969
6.984	0.978294408
6.948	0.933045681
6.912	0.97058676
6.876	0.960483406
6.84	0.959040965
6.804	0.952361839
6.768	0.896539397
6.732	0.894344379
6.696	0.86849334
6.66	0.847164037
6.624	0.788833003
6.588	0.623854201
6.552	0.585568698
6.516	0.587164162
6.48	0.693983142
6.444	0.766393648
6.408	0.797700875
6.372	0.777299752
6.336	0.756171136
6.3	0.731060131
6.264	0.695902215
6.228	0.564228106
6.192	0.401995585
6.156	0.079692948
6.12	0
6.084	0
6.048	0.076473798
6.012	0.41008015
5.976	0.57625116
5.94	0.573341821
5.904	0.587450142
5.868	0.706638989
5.832	0.819682412
5.796	0.87006121
5.76	0.878007175
5.724	0.863087324
5.688	0.861481825
5.652	0.923795249
5.616	0.906216291
5.58	0.920973083
5.544	0.890844894
5.508	0.890650478
5.472	0.926673858
5.436	0.909627976
5.4	0.888831749
5.364	0.931691042
5.328	0.891277626

5.292	0.907376515
5.256	0.899994983
5.22	0.908580639
5.184	0.944647919
5.148	0.901418609
5.112	0.910675313
5.076	0.918301432
5.04	0.898502371
5.004	0.903249881
Temp = -50 °C; Saturation field = 20 Hz<br>Spectrometer frequency: 499.46 MHz	

Table S20: *CEST profile data*

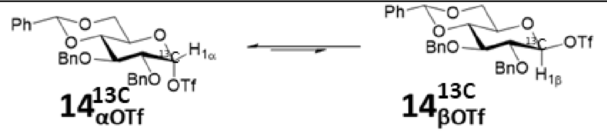

Saturation position [ppm]	Relative intensity [-]
110	1
109.8	0.99604706
109.6	0.977345651
109.4	0.98194282
109.2	0.806906387
109	0.964292192
108.8	0.926551623
108.6	0.962653474
108.4	0.950469412
108.2	0.943464201
108	0.897698914
107.8	0.914949243
107.6	0.917963986
107.4	0.899318868
107.2	0.862485223
107	0.789993808
106.8	0.739206034
106.6	0.543529875
106.4	0.129274898
106.2	0.089359586
106	0.42830918
105.8	0.600014386
105.6	0.654549321
105.4	0.713136645
105.2	0.699413939
105	0.667039861
104.8	0.596983381
104.6	0.498328132
104.4	0.560164748
104.2	0.718521901
104	0.787179215
103.8	0.802822099
103.6	0.820898043
103.4	0.783832976
103.2	0.819290597
103	0.826389627
102.8	0.829710847
102.6	0.819609585
102.4	0.817301618
102.2	0.816131998
102	0.785984576
101.8	0.809133043
101.6	0.806474816
101.4	0.796348534
101.2	0.794859927
101	0.7815125
100.8	0.769603643
100.6	0.786678842
100.4	0.777296865

100.2<br>100	0.77495137<br>0.777140498
Temp = -20 °C; Saturation field = 30 Hz<br>Spectrometer frequency: 499.46 MHz	

Optimized Computation Coordinates for 8_d, $8_{\alpha OTf}$, 16_d and $16_{\alpha OTf}$

Table S21: Optimized computation coordinates for 8_d

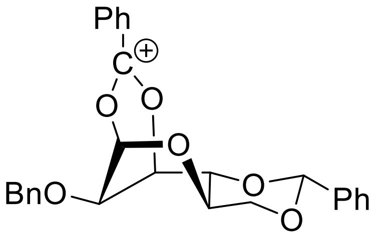

8_d

C	0.20052100	-0.43483100	0.19161600
C	-0.51039800	-0.90829600	1.48242900
C	-0.50979500	0.31735500	2.39192600
C	1.82535100	0.27264200	2.00895600
C	1.51565400	0.31767700	0.50584200
H	-1.20805900	0.25759500	3.22901000
H	0.06173400	-1.71862200	1.95943600
H	0.37193300	-1.22366300	-0.54351100
H	2.04816700	-0.76429800	2.29365300
H	1.38687400	1.37744000	0.21154100
O	0.70553400	0.77286400	2.79161600
C	3.09089600	1.10041500	2.24991500
H	3.41125900	1.06218300	3.29589500
H	2.91692800	2.15162000	1.96092100
C	3.80738600	0.47184300	0.08433600
H	3.62766300	1.51237500	-0.25697300
O	4.11446500	0.48305400	1.46601300
O	2.59598600	-0.27765800	-0.18421600
C	4.94074000	-0.18181600	-0.67099300
C	4.70225400	-0.75555700	-1.92516700
C	6.23533700	-0.16349300	-0.14160800
C	5.75842400	-1.31287600	-2.64636600
H	3.69090100	-0.77269400	-2.32260600
C	7.28883700	-0.72223100	-0.86718500
H	6.40499500	0.27865700	0.83568800
C	7.05279200	-1.29556500	-2.11910300
H	5.57207300	-1.76254400	-3.61938300
H	8.29489400	-0.71117500	-0.45340200
H	7.87578600	-1.72917600	-2.68325400
O	-1.85854300	-1.22387200	1.24867500
O	-0.74185400	0.48637600	-0.47487400
C	-1.37259200	1.35115000	0.27683000
C	-2.09051400	-2.59730500	0.82429500
H	-1.28692600	-2.91202600	0.13740600
H	-2.06773400	-3.26312200	1.70016900
C	-3.42954100	-2.64873900	0.12854000
C	-4.37000600	-3.63251800	0.44875400
C	-3.72158300	-1.71157300	-0.87250300
C	-5.58733900	-3.68971900	-0.23566200

H	-4.15342900	-4.35699100	1.23270200
C	-4.93801200	-1.76468100	-1.54997300
H	-2.98943500	-0.94279300	-1.11015800
C	-5.87256200	-2.75669800	-1.23388100
H	-6.31390400	-4.45893800	0.01648700
H	-5.16106400	-1.03558400	-2.32630300
H	-6.82132400	-2.79965000	-1.76434000
C	-2.25402100	2.29258900	-0.39014200
C	-2.32493300	2.31626400	-1.79863400
C	-3.02644700	3.18095600	0.38532800
C	-3.17095200	3.22563600	-2.42134400
H	-1.71647600	1.63182800	-2.38273300
C	-3.86918800	4.08431400	-0.25083700
H	-2.95777300	3.14692500	1.46864100
C	-3.94106300	4.10608000	-1.64959400
H	-3.23243200	3.25536700	-3.50624300
H	-4.47195200	4.77168800	0.33713700
H	-4.60179800	4.81581700	-2.14303200
O	-1.22263500	1.43719100	1.55261100

Table S22: Optimized computation coordinates for $8_{\alpha OTf}$

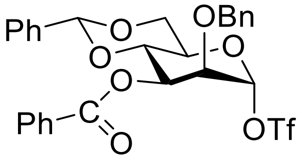

$8_{\alpha OTf}$

C	0.31964900	0.25508500	-0.10399100
C	-1.17071300	0.10223700	0.26743800
C	-1.72895700	-1.15959500	-0.42595200
C	0.36882800	-2.23139700	-0.33721500
C	1.02105000	-1.01534200	0.34889200
H	-2.79099200	-1.31525600	-0.22464100
H	-1.72237500	0.98320800	-0.07893800
H	0.40220600	0.42121300	-1.18158300
H	0.53669600	-2.14547400	-1.41848400
H	0.89296900	-1.09925200	1.44115600
O	-1.04600500	-2.31822600	-0.06059000
C	1.06905600	-3.49475700	0.16805300
H	0.70680500	-4.38956500	-0.34985200
H	0.88690600	-3.60785900	1.25217100
C	3.02051400	-2.20290400	0.46280500
H	2.87110000	-2.25808400	1.56293100
O	2.46455000	-3.36912500	-0.12805600
O	2.40302500	-1.00890400	-0.00951000
C	4.49417900	-2.14682400	0.11836600
C	5.14716900	-0.91172300	0.04904600
C	5.21255900	-3.33006900	-0.08093700
C	6.51662600	-0.86215100	-0.21608800
H	4.56833200	-0.00299000	0.18854200
C	6.58207600	-3.27703100	-0.34572700

H	4.68482700	-4.27871200	-0.04088500
C	7.23667300	-2.04402700	-0.41119600
H	7.02202500	0.10007500	-0.27504900
H	7.13863700	-4.19872800	-0.50539200
H	8.30461200	-2.00402900	-0.61770000
O	-1.22362900	-0.03993900	1.68299700
O	-1.53372300	-0.96437700	-1.87925600
O	0.91032100	1.36735800	0.60326600
C	0.68030800	2.58408000	0.03659300
C	-2.39310800	0.46406300	2.33102700
H	-2.04498400	0.91047100	3.27548700
H	-2.85067900	1.27314800	1.73776800
S	-2.76406300	-0.40454900	-2.77758700
O	-3.98013600	-0.36977300	-1.98158700
O	-2.68299100	-1.04623800	-4.07218700
C	-2.15050500	1.32740400	-2.97689200
F	-0.83385100	1.30754100	-3.16593500
F	-2.45140500	2.02239100	-1.87523800
F	-2.76081700	1.86953200	-4.02634700
C	-3.42792900	-0.61139200	2.63889500
C	-4.68935000	-0.24588400	3.12892900
C	-3.11627900	-1.96698700	2.49016400
C	-5.62761500	-1.22266400	3.46293900
H	-4.94081800	0.80849900	3.24672200
C	-4.06046900	-2.94401700	2.81897400
H	-2.14884700	-2.24304700	2.08104900
C	-5.31439700	-2.57691000	3.30963500
H	-6.60606700	-0.92809600	3.83803700
H	-3.81359000	-3.99645600	2.68996600
H	-6.04763500	-3.33912400	3.56591800
C	1.37617400	3.67500100	0.77906100
C	2.16828800	3.42777400	1.90824200
C	1.21274600	4.98214900	0.30273200
C	2.79362300	4.49191800	2.55762900
H	2.28371600	2.40733200	2.26124700
C	1.83930500	6.04248300	0.95337500
H	0.59191300	5.13169900	-0.57691500
C	2.62970500	5.79689100	2.08155600
H	3.41016300	4.30638300	3.43485500
H	1.71511600	7.05903300	0.58570100
H	3.11980000	6.62467100	2.59089400
O	-0.00959100	2.75248600	-0.94658400

Table S23: Optimized computation coordinates for 16_d

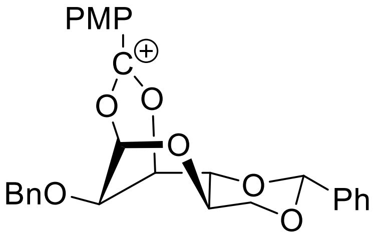

16_d

C	-0.66787300	0.71073000	0.18874300
C	-0.12703900	1.47862000	1.41779100
C	0.05024600	0.40499600	2.48900200
C	-2.23742400	-0.02492700	2.04386500
C	-1.84403100	-0.21883000	0.57241700
H	0.66790600	0.71920300	3.33309900
H	-0.86166200	2.22200600	1.76190500
H	-0.93928300	1.34765800	-0.65574700
H	-2.65895800	0.98226400	2.16525600
H	-1.51308400	-1.26685100	0.43812400
O	-1.09490900	-0.19946000	2.92249500
C	-3.34723700	-1.02960700	2.36586000
H	-3.72630900	-0.90664700	3.38541700
H	-2.97229100	-2.06036800	2.23954100
C	-4.04400600	-0.85024600	0.11236000
H	-3.66278400	-1.87682400	-0.06847200
O	-4.42141300	-0.72904800	1.47137900
O	-2.97566100	0.06581500	-0.22737400
C	-5.23379300	-0.53017600	-0.76247800
C	-5.03455500	-0.09314600	-2.07704100
C	-6.53082600	-0.72383200	-0.27594400
C	-6.13271400	0.15210700	-2.90184700
H	-4.02258700	0.06345700	-2.44089600
C	-7.62643900	-0.47731700	-1.10501700
H	-6.67079700	-1.05568600	0.74860400
C	-7.42953900	-0.04111400	-2.41756300
H	-5.97747100	0.49596700	-3.92235200
H	-8.63510200	-0.62431100	-0.72480000
H	-8.28501700	0.14915800	-3.06224900
O	1.15025100	2.01300000	1.17695000
O	0.45665100	-0.09971800	-0.30970100
C	1.18815500	-0.73040300	0.58128000
C	1.14324900	3.33697500	0.57609700
H	0.31107200	3.41264300	-0.14408100
H	0.98416000	4.09703700	1.35618700
C	2.46499100	3.53972700	-0.12663500
C	3.18838600	4.72610100	0.02870400
C	2.95647600	2.53507800	-0.97185900
C	4.38623800	4.91472800	-0.66634800
H	2.81733600	5.50598700	0.69243100
C	4.15399500	2.72097000	-1.65977100
H	2.39313400	1.61103400	-1.07966400

C	4.87009900	3.91339300	-1.50997400
H	4.94315300	5.84093000	-0.54274700
H	4.53184400	1.93881200	-2.31524700
H	5.80382100	4.05944300	-2.04878400
C	2.24511300	-1.56891400	0.09745800
C	2.42572000	-1.77262600	-1.29567700
C	3.11294200	-2.20454700	1.01315900
C	3.44301600	-2.58036500	-1.74564300
H	1.75338500	-1.29260500	-2.00102400
C	4.13749400	-3.01704700	0.56422700
H	2.97028400	-2.04521500	2.07821600
C	4.31668000	-3.21521500	-0.82439400
H	3.60518500	-2.75837500	-2.80497700
H	4.79717600	-3.49390400	1.28133000
O	0.97415300	-0.66232300	1.85878000
O	5.26654500	-3.97357400	-1.37487000
C	6.20123600	-4.66361800	-0.51685100
H	6.86249300	-5.20647700	-1.19558000
H	6.78586600	-3.95242200	0.08067600
H	5.68222400	-5.37242000	0.14103900

Table S24: Optimized computation coordinates for $16_{\alpha OTf}$

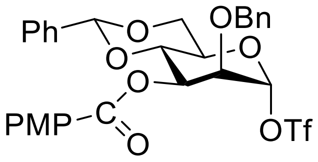

$16_{\alpha OTf}$

C	0.06763200	0.21035600	0.20586000
C	-1.28884000	-0.38656200	-0.22600400
C	-2.41842700	0.56405400	0.22573500
C	-1.02142400	2.45067300	0.00949900
C	0.17044300	1.58319500	-0.43960800
H	-3.41221200	0.18810900	-0.02690900
H	-1.42069300	-1.36425300	0.25031200
H	0.10244100	0.25502400	1.29795500
H	-0.94343200	2.60453500	1.09348200
H	0.13102000	1.44454500	-1.53307300
O	-2.29082100	1.84260800	-0.31438300
C	-0.91135300	3.80888400	-0.68685500
H	-1.68529700	4.50237900	-0.34000800
H	-1.01410800	3.67187500	-1.77864700
C	1.42989600	3.52344800	-0.70397900
H	1.38400000	3.34799500	-1.80070600
O	0.35549200	4.37817600	-0.33782600
O	1.36551100	2.26043600	-0.04899100
C	2.73055900	4.19607900	-0.31732300
C	3.85523600	3.41897900	-0.02083100
C	2.82150200	5.59157900	-0.30501200
C	5.06851300	4.03807500	0.28393500
H	3.75975000	2.33676100	-0.01758500

C	4.03637500	6.20760600	0.00015000
H	1.93294800	6.17818300	-0.52042000
C	5.16197600	5.43255300	0.29242200
H	5.94129500	3.43179800	0.51948200
H	4.10408100	7.29390600	0.01399000
H	6.10875300	5.91414500	0.53004300
O	-1.25709600	-0.48281400	-1.64643900
O	-2.30570500	0.68588800	1.69604600
O	1.15991400	-0.59558000	-0.28158000
C	1.43511000	-1.69437400	0.48094200
C	-2.00268400	-1.55231600	-2.22992800
H	-1.39649000	-1.91759800	-3.07354600
H	-2.10850600	-2.38871000	-1.51916700
S	-3.24656400	-0.23497800	2.64435200
O	-4.23874300	-0.92466500	1.83578500
O	-3.58456700	0.54952000	3.81270100
C	-1.95809000	-1.45883500	3.15102200
F	-0.81074700	-0.82319700	3.37063100
F	-1.81155600	-2.36076800	2.17551400
F	-2.37092700	-2.06557000	4.26021400
C	-3.37152500	-1.12634600	-2.74703500
C	-4.27631100	-2.09376000	-3.20571700
C	-3.71857300	0.22683400	-2.82071200
C	-5.51272600	-1.71360600	-3.72800300
H	-4.01412500	-3.15070300	-3.15088200
C	-4.96087100	0.60521200	-3.33816000
H	-3.02603700	0.96968900	-2.43577300
C	-5.85828100	-0.36029800	-3.79675600
H	-6.21005200	-2.47313000	-4.07692900
H	-5.22731500	1.65989100	-3.38187300
H	-6.82448300	-0.06345800	-4.20007200
C	2.61468100	-2.43294300	-0.03335400
C	3.34339600	-2.01640800	-1.16134800
C	3.00606100	-3.58903900	0.64897900
C	4.43822400	-2.74831200	-1.59079600
H	3.03712900	-1.11706500	-1.68743900
C	4.10548000	-4.33227400	0.22713600
H	2.42668800	-3.88776700	1.51913300
C	4.83071200	-3.91336800	-0.90100500
H	5.01977800	-2.44925500	-2.45920200
H	4.39158700	-5.22583700	0.77312700
O	0.78379400	-2.01680300	1.45354400
O	5.91827300	-4.55078200	-1.41015300
C	6.36423000	-5.73643600	-0.74869200
H	5.59552700	-6.52348800	-0.75710100
H	7.23639700	-6.07851300	-1.31370900
H	6.66153000	-5.53738500	0.29164400
